# Brief Digital Interventions for Psychological Distress: An AI-Enhanced Response-Adaptive Randomized Clinical Trial

**DOI:** 10.1001/jamanetworkopen.2025.40502

**Published:** 2025-10-31

**Authors:** Jill Newby, Sunil Gupta, Leonard Hoon, WuYi Zheng, Alexis E. Whitton, Kit Huckvale, Eileen Stech, Andrew Mackinnon, Manisha Senadeera, Artur Shvetcov, Julian Berk, Aimy Slade, Michael J. Spoelma, Jin Han, Joanne R. Beames, Rena Logothetis, Omar Dabash, Stefanus Kurniawan, Rajesh Vasa, Kon Mouzakis, Stuart Cameron, Akash Agarwal, Joshua Asbury, Joost Funke Kupper, Aliza Werner-Seidler, Simon Rosenbaum, Henry Cutler, Svetha Venkatesh, Helen Christensen

**Affiliations:** 1 Black Dog Institute, University of New South Wales Sydney, New South Wales, Sydney, Australia; 2 School of Psychology, University of New South Wales Sydney, New South Wales, Sydney, Australia; 3 Applied Artificial Intelligence Initiative, Deakin University, Victoria, Melbourne, Australia; 4 Centre for Digital Transformation of Health, The University of Melbourne, Victoria, Melbourne, Australia; 5 Discipline of Psychiatry and Mental Health, University of New South Wales Sydney, New South Wales, Sydney, Australia; 6 Department of Psychological Medicine, Sydney Children’s Hospitals Network, Sydney, New South Wales, Australia; 7 Division of Arts and Sciences and Centre for Global Health Equity, New York University Shanghai, Shanghai, People’s Republic of China; 8 Centre for Contextual Psychiatry, Department of Neuroscience, KU Leuven, Leuven, Belgium; 9 Centre for the Health Economy, Macquarie University, New South Wales, Sydney, Australia

## Abstract

**Importance:**

A large proportion of college students report experiencing psychological distress. Smartphone app–based interventions may alleviate distress, but their effectiveness across severity levels is unclear. Artificial intelligence (AI)–enhanced response-adaptive randomized clinical trials may offer an efficient method to evaluate competing interventions.

**Objective:**

To compare the effectiveness of 3 brief, 2-week self-guided smartphone application–based interventions (physical activity, mindfulness, sleep hygiene) or an active control (ecological momentary assessment [EMA]) for reducing psychological distress among college students with mild, moderate, or severe distress.

**Design, Setting, and Participants:**

This population-based AI-enhanced response-adaptive randomized clinical trial included 1282 participants with distress scores of 20 or more on the 10-item Kessler Psychological Distress Scale in 12 minitrials from November 9, 2021, with final follow-up on February 17, 2023. Participants’ distress was categorized as mild, moderate, or severe based on their normalized 21-item Depression Anxiety Stress Scale (DASS-21) scores at screening.

**Interventions:**

After a 2-week onboarding period of daily EMA, participants were assigned by a contextual multi-armed bandit algorithm to 1 of 4 two-week self-guided app interventions: physical activity, mindfulness, sleep hygiene, or a control that continued EMA.

**Main Outcomes and Measures:**

The primary outcome was change in psychological distress (DASS-21 total score) from week 2 (before intervention) to week 4 (after intervention). The primary end point was after the intervention (4 weeks). Secondary outcomes included DASS-21 subscale scores, self-reported physical activity, mindfulness, sleep quality, and app engagement, usability, and satisfaction. Analysis was performed on an intention-to-treat basis.

**Results:**

A total of 1282 individuals (mean [SD] age, 23.5 [5.2] years; 950 women [74.1%]) participated: physical activity (n = 305), mindfulness (n = 453), sleep hygiene (n = 431), or a control that continued EMA (n = 93). Among 349 participants with severe distress, physical activity (n = 79) and mindfulness (n = 180) were significantly more effective than the EMA control (n = 29) in reducing DASS-21 total scores (physical activity vs control: standardized mean difference [SMD], 0.62 [95% CI, 0.23-1.02]; mindfulness vs control: SMD, 0.53 [95% CI, 0.19-0.87]) and sleep hygiene (n = 61) (physical activity vs sleep hygiene: SMD, 0.50 [95% CI, 0.16-0.84]; mindfulness vs sleep hygiene: SMD, 0.41 [95% CI, 0.13-0.69]). Among 494 participants with mild distress, physical activity (n = 161) and sleep hygiene (n = 224) were significantly more effective than control (n = 37) in reducing DASS-21 total scores (physical activity vs control: SMD, 0.58 [95% CI, 0.30-0.86]; sleep hygiene vs control: SMD, 0.47 [95% CI, 0.20-0.73]). No significant group differences were observed among participants with moderate distress (n = 439).

**Conclusions and Relevance:**

In this AI-enhanced response-adaptive randomized clinical trial among college students, physical activity and mindfulness were most effective for severe distress, while physical activity and sleep hygiene were most effective for mild distress. These findings can guide personalized mental health interventions for college students. This trial improved efficiency by minimizing control group allocation but had reduced power to detect significant group differences.

**Trial Registration:**

http://anzctr.org.au Identifier: ACTRN12621001223820

## Key Points

**Question:**

Which brief digital intervention most effectively reduces mild, moderate, and severe levels of psychological distress among college students?

**Findings:**

This artificial intelligence–enhanced response-adaptive randomized clinical trial involving 1282 distressed college students found that smartphone-based physical activity and mindfulness were most effective for severe distress and that physical activity and sleep hygiene were most effective for mild distress; no significant differences were observed for moderate distress. The trial’s novel methods improved efficiency by reducing control group allocation.

**Meaning:**

These findings support using brief smartphone-based physical activity for mild and severe distress, mindfulness for severe distress, and sleep hygiene for mild distress.

## Teaser

This randomized clinical trial compares the effectiveness of 3 brief smartphone app–based interventions (physical activity, mindfulness, and sleep hygiene) or an active control (ecological momentary assessment) for reducing mild, moderate, and severe psychological distress among college students.

## Introduction

More than 1 in 5 college students experience psychological distress,^1^ which affects academic performance, employment, relationships, and quality of life.^1,2^ Brief smartphone application–based interventions targeting cognitive, emotional, and lifestyle factors offer a scalable means to improve college students’ mental health.^3^

Mindfulness, physical activity, and sleep hygiene are promising interventions for distress. Mindfulness reduces distress via improved attention, concentration, self-compassion, and present-focused awareness.^4,5^ Physical activity reduces distress by improving self-confidence and increasing cardiorespiratory fitness levels.^6^ Sleep hygiene reduces distress by promoting positive sleep habits that improve sleep quality.^7^ Despite the growing number of interventions for distress, the limited direct comparisons make it difficult to determine their relative effectiveness. There is limited understanding of how intervention effectiveness varies by distress severity. This lack of evidence hinders the development of tailored or stratified treatment approaches and personalized treatment recommendations for college students with distress.

Artificial intelligence (AI)–enhanced bandit-based response-adaptive trials may offer a more efficient approach than traditional randomized clinical trials (RCTs) for identifying the most effective interventions and evaluating effects in different participant subgroups. Unlike traditional RCTs that allocate participants equally across trial arms, in bandit-based response-adaptive designs, data from previous trial participants are processed by an AI algorithm that dynamically adjusts the probability that new participants will be allocated to different trial arms, in favor of better-performing interventions. This method may optimize resource allocation by directing more participants toward interventions that show promise, thereby enhancing statistical power to discern differences in treatment effects between conditions and within participant subgroups,^8^ and improving overall participant outcomes.

Although AI-enhanced response-adaptive trials have been used in other fields of medicine,^9,10,11^ they have never been used to compare the effectiveness of mental health interventions, to our knowledge. The primary goal of this contextual bandit-based AI-enhanced response-adaptive trial was to determine the relative effectiveness of brief smartphone app–based mindfulness, physical activity, and sleep hygiene and an active control for reducing psychological distress in subgroups of college students with mild, moderate, or severe levels of distress. As a secondary goal, we report on the feasibility and outcomes of this new method for detecting differences in intervention effects across distress subgroups.

## Methods

### Study Design

This 4-arm AI-enhanced response-adaptive randomized clinical trial compared mindfulness, physical activity, sleep hygiene, and an active control (ecological momentary assessment; EMA) in reducing psychological distress among college students (trial protocol^12^ in Supplement 1). This study is reported in line with the Consolidated Standards of Reporting Trials (CONSORT) reporting guideline.^13,14,15^ The trial ran across 12 minitrials from 2021 to 2023 (recruitment from October 29, 2021; first participant enrolled November 9, 2021; final follow-up on February 17, 2023; total duration, 16 months) and was approved by the University of New South Wales Sydney Human Research Ethics Committee. Participants were recruited via social and traditional media and college organizations and provided electronic informed consent to participate.

Each minitrial lasted 4 weeks (eFigure 1 in Supplement 2). Participants completed baseline assessments on their smartphone (week 0) followed by a 2-week onboarding period of daily EMA to facilitate smartphone-based passive sensing data collection (for a secondary study) and to familiarize participants with the app. Two weeks after baseline (week 2), they completed preintervention assessments, were assigned to an intervention or control for 2 weeks, and then completed a postintervention assessment (week 4). All interventions were then made available to all participants, who completed a week 12 follow-up assessment. The trial continued until a significant difference was found between the most effective and second-most effective interventions within each distress severity group (mild, moderate, and severe), or up to 12 minitrials.

### Eligible Participants and Enrollment

Inclusion criteria were age 18 years or older; Australian resident; enrolled at a higher education institution; advanced, fluent, or native English speaker; owned an eligible smartphone (iPhone 6S or Android 5 or later) with an active mobile number and internet access; and a self-rated psychological distress score of 20 or more on the 10-item Kessler Psychological Distress Scale.^16,17^ Exclusion criteria were high self-rated suicidal ideation on the Suicide Ideation Attributes Scale (scores ≥21)^18^; self-reported psychosis or bipolar disorder; previous participation in the trial; circumstances that would prevent adequate trial participation in the next 2 months (eg, travel); and/or inability to safely undertake a physical activity intervention. Participants could undertake other concurrent treatments but were discouraged from starting new treatments while participating.

### Randomization and Allocation

Each participant took part in 1 minitrial only. In the first minitrial, participants were equally assigned to 1 of 4 interventions, independent of distress severity, in a round-robin sequence (sleep hygiene, mindfulness, EMA, and physical activity) based on the order in which the participants completed their first baseline assessment task. In subsequent minitrials, allocation was determined by a contextual multi-armed bandit AI algorithm,^19^ which updated an underlying model of the effectiveness of each condition within mild, moderate, and severe distress groups (severity based on the participant’s normalized baseline scores on the 21-item Depression, Anxiety, Stress Scale [DASS-21]^20^) (eMethods 1 in Supplement 2).

The contextual multi-armed bandit AI algorithm^19^ (eMethods 2, eFigure 2, and eFigure 3 in Supplement 2) aimed to identify the most effective intervention for reducing distress as quickly as possible for the 3 distress subgroups. The algorithm explored intervention outcomes (preintervention to postintervention changes in distress) to ensure that 1 or more trial arms were not discarded from the trial until the best-performing intervention emerged, and to ensure the minitrials maximized statistical power for key outcome comparisons while controlling for false detection rates. There was no minimum or maximum allocation to trial arms.

Participants were allocated after they completed their baseline assessment. Their allocation was revealed to them within the app, after they completed the preintervention assessment (ie, after the initial 2-week EMA period). Participants and operational staff were unblinded. All other investigators, trial staff, and statisticians were blinded until the final analysis was completed.

### Interventions

All interventions were brief, self directed, standardized, accessible anytime, and delivered via a smartphone app. See the trial protocol^12^ in Supplement 1 (to access and view intervention content, contact the corresponding author) and eTable 1 in Supplement 2 for details. Each intervention intentionally involved a similar time commitment and effort over the 2-week period. A daily reminder encouraged engagement. Participants could not delay their intervention.

#### Mindfulness

After a brief introductory video, guided audio meditations of less than 5 minutes were released sequentially, every second day. Content included instructions to enhance mindfulness during everyday activities and adopt nonjudgmental, accepting, and self-compassionate responses and present-focused awareness.

#### Physical Activity

After a brief introductory infographic about the guidelines and benefits of physical activity, participants set a physical activity goal (eg, increasing steps). The intervention also included an evidence-based, 7-minute, high-intensity interval training guided video.^21^

#### Sleep Hygiene

This intervention included infographics on topics designed to shift behaviors and daily habits to improve sleep quality and daytime alertness (eg, sleep environments and stimulus control).^22^

#### EMA Control

This condition included twice-daily surveys of mood (state emotions, adapted from the International Positive and Negative Affect Short-Form^23^) and associated behavioral responses. EMA was an active comparator designed to control for various factors such as app engagement and repeated assessment.

### Primary Outcome

The primary outcome was self-rated psychological distress, assessed using the DASS-21 total score^20^ at the postintervention assessment (week 4). Scores range from 0 to 126, with higher scores indicating higher levels of distress; DASS-21 scores are multiplied by 2 to derive the full DASS-42 total scores.

### Secondary Outcomes

Secondary outcomes were scores on the DASS-21 depression, anxiety, and stress subscales^20^; the Physical Activity Vital Sign (PAVS), which measured the number of minutes engaged in moderate-to-strenuous physical activity^24^; item 6 of the Modified Pittsburgh Sleep Quality Index, which measured sleep quality (range, 0-3 [higher scores indicated better quality sleep])^25^; and a bespoke single item that measured mindfulness (range, 0-4 [higher scores indicate more mindfulness]).

Two items from the Credibility Expectancy Questionnaire^26^ measured participants’ perceived credibility and expected intervention benefit. App engagement metrics included the self-reported daily log of time spent engaging in the intervention, module access, completion, and time spent on pages (collected within the app).^27^ App usability and satisfaction were assessed with items from the System Usability Scale^28^ and mHealth App Usability Questionnaire.^29^ Additional self-report questionnaires and smartphone sensor data for digital phenotyping were collected and will be reported separately.^12^

### Sample Size Calculation

Sample size calculation was based on fixed trial estimates. We aimed to recruit at least 120 participants for each of the 12 minitrials, with 80 expected to start (20 per arm in minitrial 1). Allowing for 20% attrition, 64 participants were expected to complete postintervention assessments. Traditional power estimation tools are not well suited to adaptive allocation schemes. Assuming recruitment of a minimum of 192 participants in each of the 2 best-performing arms, it would have 80% power (2-tailed α = .05) to detect a 0.29 (small) between-groups effect size.

### Statistical Analysis

Refer to eMethods 3 and eFigure 4 in Supplement 2 and Huckvale et al^12^ for the statistical analysis plan. Analyses were conducted in R, version 2023.09.0+463 (R Project for Statistical Computing). Because the adaptive trial design results in unequal probabilities of allocation across interventions, we used the Horvitz-Thompson estimator with inverse propensity weighting. This method reweights each observation by the inverse of its intervention allocation probability, allowing for estimation of marginal intervention effects as if all arms had been equally sampled. This shifts the estimand from a potentially biased sample mean to an unbiased population-level mean effect.

Mixed-model repeated-measures analyses of variance with an unstructured covariance structure were used with all available data from the intention-to-treat sample to assess intervention (and control) effectiveness in reducing distress in each severity group (mild, moderate, and severe). Planned contrasts between each trial arm compared the differences in preintervention with postintervention changes in distress (DASS-21 total score) and secondary outcomes (eg, sleep quality). These contrasts included fixed effects for intervention, time, and their interactions as covariates. The unstructured covariance matrix allowed for the modeling of within-individual correlations across time points to allow for estimation in the change in outcome scores before and after the intervention. All tests were 2-sided. To control for type I error across multiple comparisons and repeated interim analyses, we combined the Benjamini-Hochberg^30^ procedure with an α-spending approach (eTable 2 in Supplement 2). Interim analyses were planned after minitrials 4 and 8, with the final analysis after minitrial 12. At each testing point, a portion of the overall α (.05) was allocated using an O’Brien-Fleming–type α-spending function. Within each time point, the Benjamini-Hochberg procedure was applied to adjust significance thresholds across intervention comparisons, allowing greater sensitivity while controlling the false discovery rate.

Planned interim analyses were conducted after minitrials 4 and 8 (eTable 3 and eTable 4 in Supplement 2). If any condition was significantly more effective in reducing distress than all other conditions within a specific distress severity group, it could be removed from subsequent minitrials so that the effect of other interventions could be estimated with greater precision. One-sided Welch *t* tests with Satterthwaite-adjusted degrees of freedom were used. All 4 conditions were retained in all minitrials because, at both interim analyses, no condition was more effective than all other conditions in any distress group (eTable 5 in Supplement 2). Secondary outcomes were analyzed at the study’s conclusion.

## Results

### Enrollment and Participant Characteristics

The sample of 1282 individuals (mean [SD] age, 23.5 [5.2] years; 950 women [74.1%], 280 men [21.8%], and 48 nonbinary or gender-neutral individuals [3.7%]) was predominantly undergraduate students (1004 [78.3%]), with 467 (36.4%) identifying as sexually diverse (Table 1). eFigure 5 in Supplement 2 shows DASS-21 subscale severity ranges by distress group. The Figure shows the participant flow diagram. A total of 1394 individuals were allocated to a condition, 1282 (92.0%) completed the postintervention assessment, and 701 (50.3%) completed the follow-up assessment.

**Table 1.** Sample Demographic Characteristics

Charactaristic	Total sample (N = 1282)	Mindfulness (n = 453)	Physical activity (n = 305)	Sleep hygiene (n = 431)	Ecological momentary assessment (n = 93)
Age, mean (SD), y	23.5 (5.2)	23.3 (5.0)	23.5 (5.2)	24.0 (5.6)	22.9 (4.1)
Student type, No. (%)					
Undergraduate or TAFE student	1004 (78.3)	361 (79.7)	227 (74.4)	340 (78.9)	76 (81.7)
Postgraduate coursework	218 (17.0)	75 (16.6)	56 (18.4)	71 (16.5)	16 (17.2)
Postgraduate research	60 (4.7)	17 (3.8)	22 (7.2)	20 (4.6)	1 (1.1)
International student status, No. (%)					
Domestic (Australian)	1208 (94.2)	431 (95.1)	285 (93.4)	407 (94.4)	85 (91.4)
International	74 (5.8)	22 (4.9)	20 (6.6)	24 (5.6)	8 (8.6)
Gender, No. (%)					
Woman or female	950 (74.1)	347 (76.6)	217 (71.1)	312 (72.4)	74 (79.6)
Man or male	280 (21.8)	91 (20.1)	72 (23.6)	105 (24.4)	12 (12.9)
Nonbinary or gender neutral	48 (3.7)	14 (3.1)	15 (4.9)	12 (2.8)	7 (7.5)
Did not disclose	4 (0.3)	1 (0.2)	1 (0.3)	2 (0.5)	0
Sex, No. (%)					
Female	1005 (78.4)	366 (80.8)	234 (76.7)	325 (75.4)	80 (86.0)
Male	275 (21.5)	87 (19.2)	70 (23.0)	105 (24.4)	13 (14.0)
Did not disclose	2 (0.2)	0	1 (0.3)	1 (0.2)	0
Sexual orientation, No. (%)					
Heterosexual	740 (57.7)	245 (54.1)	185 (60.7)	269 (62.4)	41 (44.1)
Homosexual	79 (6.2)	28 (6.2)	21 (6.9)	21 (4.9)	9 (9.7)
Bisexual	315 (24.6)	120 (26.5)	71 (23.3)	100 (23.2)	24 (25.8)
Other nonheterosexual^a^	73 (5.7)	29 (6.4)	17 (5.6)	19 (4.4)	8 (8.6)
Not known	64 (5.0)	29 (6.4)	8 (2.6)	18 (4.2)	9 (9.7)
Did not disclose	11 (0.9)	2 (0.4)	3 (1.0)	4 (0.9)	2 (2.2)
Indigenous status, No. (%)					
Not Aboriginal or Torres Strait Islander	1245 (97.1)	438 (96.7)	301 (98.7)	417 (96.8)	89 (95.7)
Aboriginal	22 (1.7)	9 (2.0)	4 (1.3)	9 (2.1)	0
Torres Strait Islander	1 (0.1)	1 (0.2)	0	0	0
Unknown	9 (0.7)	3 (0.7)	0	3 (0.7)	3 (3.2)
Did not disclose	5 (0.4)	2 (0.4)	0	2 (0.5)	1 (1.1)
Primary language, No. (%)					
Arabic	1 (0.1)	1 (0.2)	0	0	0
Cantonese	16 (1.2)	7 (1.5)	0	7 (1.6)	2 (2.2)
English	1176 (91.7)	418 (92.3)	281 (92.1)	396 (91.9)	81 (87.0)
Hindi	7 (0.5)	2 (0.4)	2 (0.7)	2 (0.5)	1 (1.1)
Italian	1 (0.1)	1 (0.2)	0	0	0
Mandarin	13 (1.0)	2 (0.4)	6 (2.0)	3 (0.7)	2 (2.2)
Punjabi	2 (0.2)	0	1 (0.3)	1 (0.2)	0
Spanish	4 (0.3)	3 (0.7)	0	1 (0.2)	0
Vietnamese	10 (0.8)	4 (0.9)	2 (0.7)	3 (0.7)	1 (1.1)
Other^b^	50 (3.9)	15 (3.3)	12 (3.9)	17 (3.9)	6 (6.5)
Did not disclose	2 (0.2)	0	1 (0.3)	1 (0.2)	0
Ancestry, No. (%)					
Australian	851 (66.4)	310 (68.4)	199 (65.2)	287 (66.6)	55 (59.1)
Chinese or Cantonese	107 (8.3)	33 (7.3)	28 (9.2)	36 (8.4)	10 (10.8)
Dutch	35 (2.7)	9 (2.0)	8 (2.6)	13 (3.0)	5 (5.4)
English	278 (21.7)	101 (22.3)	68 (22.3)	93 (21.6)	16 (17.2)
German	47 (3.7)	24 (5.3)	7 (2.3)	14 (3.2)	2 (2.2)
Greek	25 (2.0)	10 (2.2)	5 (1.6)	9 (2.1)	1 (1.1)
Indian	50 (3.9)	14 (3.1)	7 (2.3)	25 (5.8)	4 (4.3)
Irish	91 (7.1)	41 (9.1)	17 (5.6)	26 (6.0)	7 (7.5)
Italian	39 (3.0)	14 (3.1)	10 (3.3)	11 (2.6)	4 (4.3)
Scottish	76 (5.9)	24 (5.3)	14 (4.6)	27 (6.3)	11 (11.8)
Vietnamese	20 (1.6)	5 (1.1)	5 (1.6)	8 (1.9)	2 (2.2)
Other^b^	210 (16.4)	70 (15.5)	60 (19.7)	63 (14.6)	17 (18.3)
Did not disclose	3 (0.2)	1 (0.2)	1 (0.3)	1 (0.2)	0 (0.0)

Abbreviation: TAFE, technical and further education (ie, vocational education).

^a^ Encompasses labels referring to abrosexuality, asexuality, bicuriousity, demisexuality, omnisexuality, pansexuality, and queerness.

^b^ Encompasses all languages or ancestries that are not listed; participants were not required to specify what these were.

**Figure.**
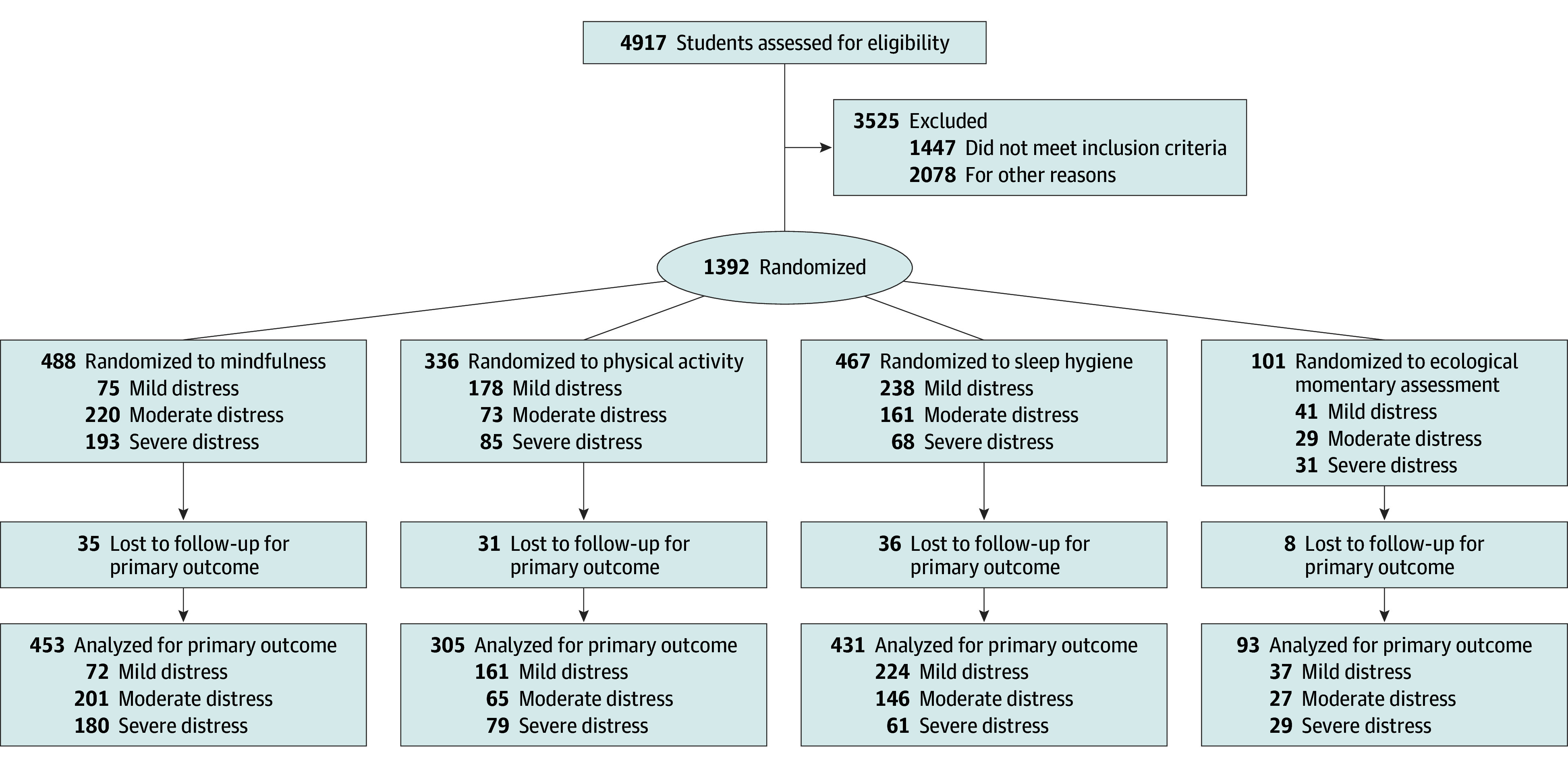
Flow Diagram

### Primary Outcome

Table 2 presents the observed mean change scores on the primary outcome, DASS-21 total score for each trial arm and severity group, pooled across minitrials. Table 3 presents the standardized mean differences (SMDs), 95% CIs, and significance tests for bias-corrected and log-transformed DASS-21 total scores. eFigure 6 and eTables 6, 7, and 8 in Supplement 2 show observed DASS-21 total scores and the participant allocation per minitrial for each trial arm and severity group.

**Table 2.** Unweighted Observed Mean Values for DASS-21 Total Scores, Before and After Intervention, and Change by Severity and Intervention Group

Severity group	Observed DASS-21 score, mean (SD)		Pre-post change
	Preintervention	Postintervention	
Mild			
Physical activity	33.6 (12.6)	24.5 (13.4)	−8.2 (13.5)
Mindfulness	30.1 (13.0)	23.5 (12.1)	−6.1 (12.2)
Sleep hygiene	31.7 (13.7)	24.9 (14.3)	−7.3 (14.7)
EMA	27.4 (14.0)	26.6 (14.5)	−0.6 (7.6)
Moderate			
Physical activity	46.4 (11.8)	34.8 (15.4)	−10.8 (16.1)
Mindfulness	50.1 (13.2)	38.8 (16.8)	−12.1 (17.9)
Sleep hygiene	45.4 (12.9)	36.3 (15.3)	−9.8 (14.8)
EMA	47.3 (11.9)	44.3 (18.0)	−3.0 (17.8)
Severe			
Physical activity	66.4 (18.7)	50.4 (24.8)	−16.6 (23.2)
Mindfulness	63.2 (17.7)	48.9 (21.9)	−14.5 (20.6)
Sleep hygiene	65.7 (20.0)	56.7 (21.4)	−7.6 (19.8)
EMA	69.4 (19.4)	65.4 (22.4)	−4.4 (23.3)

Abbreviations: DASS-21; 21-item Depression Anxiety Stress Scale (scores range from 0 to 126, with higher scores indicating higher distress); EMA, ecological momentary assessment.

**Table 3.** Group Comparisons of the Before and After Changes in Bias-Corrected and Log-Transformed DASS-21 Total Scores

Comparison	Mild distress			Moderate distress			Severe distress		
	SMD (95% CI)	*P* value	BH-adjusted *P* value	SMD (95% CI)	*P* value	BH-adjusted *P* value	SMD (95% CI)	*P* value	BH-adjusted *P* value
Mindfulness vs control	0.34 (0.01 to 0.67)	.06	.03	0.47 (0.09 to 0.84)	.02	.008	0.53 (0.19 to 0.87)^a^	.005	.03
Physical activity vs control	0.58 (0.30 to 0.86)^a^	.001	.007	0.45 (0.02 to 0.88)	.03	.02	0.62 (0.23 to 1.02)^a^	.003	.01
Sleep hygiene vs control	0.47 (0.20 to 0.73)^a^	.006	.01	0.39 (0.01 to 0.76)	.04	.02	0.12 (−0.26 to 0.50)	.30	.04
Physical activity vs mindfulness	0.24 (−0.03 to 0.51)	.06	.02	−0.01 (−0.34 to −0.31)	.46	.05	0.09 (−0.22 to 0.40)	.26	.04
Sleep hygiene vs mindfulness	0.13 (−0.12 to 0.39)	.18	.04	−0.08 (−0.31 to 0.15)	.24	.03	−0.41 (−0.69 to −0.13)^a^	.005	.02
Physical activity vs sleep hygiene	0.11 (−0.10 to 0.31)	.16	.04	0.07 (−0.25 to 0.38)	.34	.04	0.50 (0.16 to 0.84)^a^	.003	.007

Abbreviations: BH, Benjamini-Hochberg; DASS-21; 21-item Depression Anxiety Stress Scale (scores range from 0 to 126, with higher scores indicating higher distress); SMD, standardized mean difference.

^a^ *P* value lower than BH-adjusted critical *P* value.

For participants with mild distress, physical activity (n = 161) and sleep hygiene (n = 224) were significantly more effective in reducing DASS-21 total scores than the control (n = 37) (physical activity vs control: SMD, 0.58 [95%CI, 0.30-0.86]; sleep hygiene vs control: SMD, 0.47 [95% CI, 0.20-0.73]) (Table 3). For participants with moderate distress, group differences were not statistically significant. For participants with severe distress, physical activity (n = 79) and mindfulness (n = 180) were significantly more effective than control (n = 29) in reducing DASS-21 total scores (physical activity vs control: SMD, 0.62 [95% CI, 0.23-1.02]; mindfulness vs control: SMD, 0.53 [95% CI, 0.19-0.87]). Both physical activity (n = 79) and mindfulness (n = 180) were also significantly more effective than sleep hygiene (n = 61) in reducing DASS-21 total scores for participants with severe distress (physical activity vs sleep hygiene: SMD, 0.50 [95% CI, 0.16-0.84]; mindfulness vs sleep hygiene: SMD, 0.41 [95% CI, 0.13-0.69]). When we repeated the analyses controlling for preintervention DASS-21 total scores, the same pattern of results was observed.

### Secondary Outcomes

Secondary outcomes for DASS-21 depression, anxiety, and stress subscales are available in eTables 9, 10, and 11 in Supplement 2 with significance tests in eTables 12, 13, and 14 in Supplement 2. For participants with severe distress, both physical activity (n = 79) and mindfulness (n = 180) were superior in reducing DASS-21 depression scores compared with the control (n = 29) (physical activity vs control: SMD, 0.61 [95% CI, 0.18-1.04]; mindfulness vs control: SMD, 0.61 [95% CI, 0.23-1.00]) and compared with sleep hygiene (n = 61) (physical activity vs sleep hygiene: SMD, 0.50 [95% CI, 0.15-0.85]; mindfulness vs sleep hygiene: SMD, 0.50 [95% CI, 0.22-0.78]) (eTable 12 in Supplement 2).

For participants with mild distress, physical activity (n = 161) was superior to mindfulness (n = 72) in reducing DASS-21 anxiety scores (physical activity vs mindfulness: SMD, 0.35 [95% CI, 0.04-0.66]) (eTable 13 in Supplement 2). For participants with mild distress, all active interventions were better than the control in reducing DASS-21 stress scores (physical activity [n = 161] vs control [n = 37]: SMD, 0.65 [95% CI, 0.36-0.93]; mindfulness [n = 72] vs control [n = 37]: SMD, 0.57 [95% CI, 0.23-0.90]; sleep hygiene [n = 224] vs control [n = 37]: SMD, 0.47 [95% CI, 0.20-0.74]) (eTable 14 in Supplement 2). For the group with severe distress, physical activity (n = 79) was superior to both control (n = 29) (physical activity vs control: SMD, 0.61 [95% CI, 0.23-1.00]) and sleep hygiene (n = 61) (physical activity vs sleep hygiene: SMD, 0.55 [95% CI, 0.23-0.87]) in reducing DASS-21 stress scores.

Outcomes for sleep quality, mindfulness, and PAVS (physical activity levels) are shown in eTables 15, 16, and 17 in Supplement 2, and significance testing results are presented in eTables 18, 19, and 20 in Supplement 2. In all severity subgroups, there were no differences between trial arms in changes in sleep quality. At all levels of distress severity, the mindfulness intervention was associated with greater reported mindfulness relative to all other arms (SMDs ranged from 0.49 [95% CI, 0.18-0.80] to 0.87 [95% CI, 0.44-1.29]) (eTable 19 in Supplement 2).

For participants with mild distress, changes in self-reported physical activity (PAVS scores) were greater in the physical activity condition (n = 161) compared with the control (n = 37) (SMD, 0.53 [95% CI, 0.06-1.00]) (eTable 20 in Supplement 2). For participants with severe distress, changes in physical activity (n = 79) were greater in the physical activity arm compared with both mindfulness (n = 180) (SMD, 0.32 [95% CI, 0.07-0.57]) and sleep hygiene (n = 61) (SMD, 0.56 [95% CI, 0.20-0.92]).

### Intervention Engagement

eTable 21 in Supplement 2 displays intervention expectancy, engagement, and usability ratings. Of the participants allocated to an intervention, 83.2% (1066 of 1282) accessed it, 29.3% (375 of 1282) accessed all content, and 61.7% (791 of 1282) accessed half of all content. Nearly two-thirds of participants (68.8% [818 of 1189]) attained our a priori criterion of acceptable engagement. The interventions were acceptable to participants.

### Comparison of Sample Size With Fixed Allocation RCT

We calculated the sample size needed for a fixed allocation RCT to detect the smallest significant effect (SMD, 0.41) observed in this trial. Allowing for within-group correction for multiple testing (.05/6), a sample of 146 participants per intervention, per severity subgroup would be required, totaling 1752 participants.

## Discussion

In this bandit-based AI-enhanced response-adaptive RCT, we compared the effectiveness of brief smartphone app–based mindfulness, physical activity, and sleep hygiene interventions as well as an EMA active control for reducing psychological distress among college students and showed that the effectiveness of these interventions varied across distress severity subgroups. For students with severe distress, physical activity and mindfulness were more effective than sleep hygiene and EMA. For students with moderate distress, no differences were found. For those with mild distress, physical activity and sleep hygiene were more effective than EMA. Changes in the target behaviors were observed in the desired directions.

Despite being brief, 2-week interventions, the between-group effect sizes relative to EMA control were comparable with meta-analyses of longer app interventions with active controls for depression (Hedges *g* = 0.19)^31^ and anxiety (Hedges *g* = 0.22).^32,33^ User-centered design features (eg, notifications and evolving character)^12^ may have contributed to usability, satisfaction, and engagement relative to past research.^34,35^

These results support the use of brief smartphone app–based interventions to provide on-demand, anonymous, low-intensity mental health support to college students with distress. These results may optimize outcomes through improved treatment precision, by selecting the most effective treatment for an individual based on their distress levels. Physical activity, a nonstigmatizing intervention with mental and physical health benefits,^36^ was the most effective and recommended intervention for mild and severe distress. Results also support using mindfulness for severe distress (in line with meta-analyses of samples of individuals with depression and anxiety^37,38^), but not mild distress. In contrast, sleep hygiene was effective only for mild but not severe distress, suggesting more intensive insomnia treatments may be needed for students with severe distress.^7^ Given that no clear intervention recommendations can be made for participants with moderate distress, future trials should focus on this subgroup and explore the reasons for the lack of group differences (eg, power and symptom heterogeneity).

### Implications for AI-Enhanced Adaptive Trials

Adaptive trials have advantages over standard RCTs including efficiency (less time and resources) as well as ethical and health benefits.^8,39^ Our study investigated multiple subgroups, demonstrating the feasibility and capability of this method to investigate personalized intervention recommendations. Although future comparisons with standard RCTs are needed (eg, in stroke research),^40^ the algorithm varied intervention allocation across minitrials, and more participants were allocated to better-performing interventions (n = 305-453) than the control (n = 93). Overall, participants experienced more health benefits compared with a standard RCT because fewer participants were allocated to less-effective conditions. However, the smaller control group, coupled with strict significance thresholds, reduced the power to detect group differences.

Future research should identify the ideal use case for the AI-enhanced adaptive-response trial design, including the ideal interventions, type and timing of outcomes, and participant features used in the contextual multi-armed bandit algorithm. We used baseline self-reported psychological distress because it is strongly associated with digital intervention outcomes,^41^ is easily assessed, and is expected to change with brief interventions. However, psychological distress may reflect varied factors (eg, acute stress related to college or mental disorders). Future research should explore using other participant characteristics in the algorithm (eg, treatment preferences, history, and sociodemographic characteristics) to help inform more personalized treatment recommendations.

### Limitations

This study has some limitations. First, participants were not blinded to their intervention. Second, self-reported outcomes were measured immediately after the intervention, with no long-term follow-up data. Third, the interventions were brief. Longer interventions may be needed, especially for severe symptoms. Fourth, the sample, primarily English-speaking, female, domestic students recruited via social media, may limit generalizability. Fifth, the findings from smartphone app–based interventions may not apply to in-person interventions.

## Conclusions

This AI-enhanced response-adaptive RCT, conducted with college students experiencing elevated psychological distress, revealed differences in the effectiveness of brief smartphone app–based interventions for mild and severe distress, but not moderate distress. Physical activity was the most effective intervention for mild and severe distress, mindfulness was effective for severe distress, and sleep hygiene was effective for mild distress.

This AI-enhanced response-adaptive trial was feasible and offered some advantages over traditional RCTs, such as reduced allocation to the underperforming control and detecting varying treatment effects based on distress severity. This finding shows the potential for treatment personalization at scale. Future research should compare the efficiency and utility of AI-enhanced response-adaptive trials with conventional RCTs in mental health.

## Notes

Supplement 1.
Trial Protocol and Statistical Analysis Plan

Supplement 2. **eFigure 1.** Trial Schematic **eMethods 1.** Method of Calculating Severity Scores **eMethods 2.** Contextual Multi-Arm Bandit Algorithme **Figure 2.** Overview of the Contextual Multi-Arm Bandit (MAB) Algorithm Framework **eFigure 3.** Illustration of How the Contextual MAB Algorithm Allocates Participants to an Interventione **Table 1.** Intervention Content **eMethods 3.** Statistical Analysis Plan **eFigure 4.** Hypothesis Testing Procedure and Processing of DASS-21 Scores Including Bias Adjustment and Log Transformation **eTable 2.** Maximum *P* Value for Each Distress Severity Group at Each Interim Analysis and Final Analysis **eTable 3.** Detailed Breakdown Used to Calculate Final Maximum *P* Values for Each Distress Severity Group **eTable 4.** Number of Participants Per Distress Severity Group Included in the Interim Analyses and Final Analysis **eTable 5.** Interim Hypothesis Tests After Mini-Trials 4 and 8 for DASS-21 Total Scores
eResults.
**eFigure 5.** Number of Participants in the Mild, Moderate, and Severely Distressed Subgroups With Scores in the Normal/Mild, Moderate, and Severe/Extremely Severe Ranges on the DASS-21 Depression, Anxiety and Stress Subscales at Baseline **eFigure 6.** Mean Change in DASS-21 Total Score From Pre- to Post-Intervention (Top), and the Number of Participants (Bottom) Allocated Across Each of the 12 Mini-Trials, for Mild (a), Moderate (b), and Severe (c) Distress Groups **eTable 6.** Observed DASS-21 Total Scores for the Mild Distress Group by Intervention Group Across Each of the Twelve Mini-Trials **eTable 7.** Observed DASS-21 Total Scores for the Moderate Distress Group by Intervention Group Across Each of the Twelve Mini-Trials **eTable 8.** Observed DASS-21 Total Scores for the Severe Distress Group by Intervention Group Across Each of the Twelve Mini-Trials **eTable 9.** Unweighted Observed Means and Standard Deviations for DASS-21 Depression Subscale Scores Pre- and Post-Intervention, and Change by Severity and Intervention Group **eTable 10.** Unweighted Observed Means and Standard Deviations for DASS-21 Anxiety Subscale Scores Pre- and Post-Intervention, and Change by Severity and Intervention Group **eTable 11.** Unweighted Observed Means and Standard Deviations for DASS-21 Stress Subscale Scores Pre- and Post-Intervention, and Change by Severity and Intervention Group **eTable 12.** Group Comparisons of the Pre- to Post-Changes in Bias Corrected and Log Transformed DASS-21 Depression Subscale Scores **eTable 13.** Group Comparisons of the Pre- to Post-Changes in Bias Corrected and Log Transformed DASS-21 Anxiety Subscale Scores **eTable 14.** Group Comparisons of the Pre- to Post-Changes in Bias Corrected and Log Transformed for DASS-21 Stress Subscale Scores **eTable 15.** Unweighted Observed Means and Standard Deviations for Sleep Quality Scores Pre- and Post-Intervention, and Change by Severity and Intervention Group **eTable 16.** Unweighted Observed Means and Standard Deviations for Mindfulness Scores Pre- and Post-Intervention, and Change by Severity and Intervention Group **eTable 17.** Unweighted Observed Means and Standard Deviations for Physical Activity Vital Sign Scores Pre- and Post-Intervention, and Change by Severity and Intervention Group **eTable 18.** Group Comparisons of the Pre- to Post-Changes in Bias Corrected and Log Transformed Sleep Quality Scores **eTable 19.** Group Comparisons of the Pre- to Post-Changes in Bias Corrected and Log Transformed Mindfulness Scores **eTable 20.** Group Comparisons of the Pre- to Post-Changes in Bias Corrected and Log Transformed Physical Activity Vital Sign Scores **eTable 21.** Descriptive Statistics for the App User Engagement Metrics Across Groups
eReferences.

Supplement 3.
Data Sharing Statement

## References

[1] Auerbach RP, Alonso J, Axinn WG, . Mental disorders among college students in the World Health Organization World Mental Health Surveys. Psychol Med. 2016;46(14):2955-2970. doi:10.1017/S0033291716001665 27484622 PMC5129654

[2] Stallman HM. Psychological distress in university students: a comparison with general population data. Aust Psychol. 2010;45(4):249-257. doi:10.1080/00050067.2010.482109

[3] Oliveira C, Pereira A, Vagos P, Nóbrega C, Gonçalves J, Afonso B. Effectiveness of mobile app-based psychological interventions for college students: a systematic review of the literature. Front Psychol. 2021:12:647606. doi:10.3389/fpsyg.2021.647606 34045994 PMC8144454

[4] Dawson AF, Brown WW, Anderson J, . Mindfulness-based interventions for university students: a systematic review and meta-analysis of randomised controlled trials. Appl Psychol Health Well Being. 2020;12(2):384-410. doi:10.1111/aphw.12188 31743957

[5] Chen B, Yang T, Xiao L, Xu C, Zhu C. Effects of mobile mindfulness meditation on the mental health of university students: systematic review and meta-analysis. J Med Internet Res. 2023;25:e39128. doi:10.2196/39128 36596239 PMC9856434

[6] Singh B, Olds T, Curtis R, . Effectiveness of physical activity interventions for improving depression, anxiety and distress: an overview of systematic reviews. Br J Sports Med. 2023;57(18):1203-1209. doi:10.1136/bjsports-2022-106195 36796860 PMC10579187

[7] Friedrich A, Schlarb AA. Let’s talk about sleep: a systematic review of psychological interventions to improve sleep in college students. J Sleep Res. 2018;27(1):4-22. doi:10.1111/jsr.12568 28618185

[8] Pallmann P, Bedding AW, Choodari-Oskooei B, . Adaptive designs in clinical trials: why use them, and how to run and report them. BMC Med. 2018;16(1):29. doi:10.1186/s12916-018-1017-7 29490655 PMC5830330

[9] Burnett T, Mozgunov P, Pallmann P, Villar SS, Wheeler GM, Jaki T. Adding flexibility to clinical trial designs: an example-based guide to the practical use of adaptive designs. BMC Med. 2020;18(1):352. doi:10.1186/s12916-020-01808-2 33208155 PMC7677786

[10] Bothwell LE, Avorn J, Khan NF, Kesselheim AS. Adaptive design clinical trials: a review of the literature and ClinicalTrials.gov. BMJ Open. 2018;8(2):e018320. doi:10.1136/bmjopen-2017-018320 29440155 PMC5829673

[11] Allender S, Hayward J, Gupta S, . Bayesian strategy selection identifies optimal solutions to complex problems using an example from GP prescribing. NPJ Digit Med. 2020;3(1):7. doi:10.1038/s41746-019-0205-y 31993505 PMC6971230

[12] Huckvale K, Hoon L, Stech E, . Protocol for a bandit-based response adaptive trial to evaluate the effectiveness of brief self-guided digital interventions for reducing psychological distress in university students: the Vibe Up study. BMJ Open. 2023;13(4):e066249. doi:10.1136/bmjopen-2022-066249 37116996 PMC10151864

[13] Hopewell S, Chan AW, Collins GS, . CONSORT 2025 statement: updated guideline for reporting randomised trials. BMJ. 2025;389:e081123. doi:10.1136/bmj-2024-081123 40228833 PMC11995449

[14] Dimairo M, Pallmann P, Wason J, ; ACE Consensus Group. The Adaptive Designs CONSORT Extension (ACE) statement: a checklist with explanation and elaboration guideline for reporting randomised trials that use an adaptive design. BMJ. 2020;369:m115. doi:10.1136/bmj.m115 32554564 PMC7298567

[15] Liu X, Rivera SC, Moher D, Calvert MJ, Denniston AK; SPIRIT-AI and CONSORT-AI Working Group. Reporting guidelines for clinical trial reports for interventions involving artificial intelligence: the CONSORT-AI Extension. BMJ. 2020;370:m3164. doi:10.1136/bmj.m3164 32909959 PMC7490784

[16] Kessler RC, Andrews G, Colpe LJ, . Short screening scales to monitor population prevalences and trends in non-specific psychological distress. Psychol Med. 2002;32(6):959-976. doi:10.1017/S0033291702006074 12214795

[17] Andrews G, Slade T. Interpreting scores on the Kessler Psychological Distress Scale (K10). Aust N Z J Public Health. 2001;25(6):494-497. doi:10.1111/j.1467-842X.2001.tb00310.x 11824981

[18] van Spijker BAJ, Batterham PJ, Calear AL, . The Suicidal Ideation Attributes Scale (SIDAS): community-based validation study of a new scale for the measurement of suicidal ideation. Suicide Life Threat Behav. 2014;44(4):408-419. doi:10.1111/sltb.12084 24612048

[19] Auer P, Cesa-Bianchi N, Fischer PJML. Finite-time analysis of the multiarmed bandit problem. Machine Learning. 2002;47:235-256. doi:10.1023/A:1013689704352

[20] Lovibond SH, Lovibond PF. Manual for the Depression Anxiety Stress Scales. 2nd ed. Psychology Foundation of Australia; 1995.

[21] Klika B, Jordan C. High-intensity circuit training using body weight: maximum results with minimal investment. ACSM’s Health & Fitnest Journal. 2013;17(3):8-13. doi:10.1249/FIT.0b013e31828cb1e8

[22] Chung KF, Lee CT, Yeung WF, Chan MS, Chung EW, Lin WL. Sleep hygiene education as a treatment of insomnia: a systematic review and meta-analysis. Fam Pract. 2018;35(4):365-375. doi:10.1093/fampra/cmx122 29194467

[23] Thompson ER. Development and validation of an internationally reliable short-form of the Positive and Negative Affect Schedule (PANAS). J Cross Cult Psychol. 2007;38(2):227-242. doi:10.1177/0022022106297301

[24] Greenwood JLJ, Joy EA, Stanford JB. The Physical Activity Vital Sign: a primary care tool to guide counseling for obesity. J Phys ActHealth. 2010;7(5):571-576. doi:10.1123/jpah.7.5.571 20864751

[25] Buysse DJ, Reynolds CF III, Monk TH, Berman SR, Kupfer DJ. The Pittsburgh Sleep Quality Index: a new instrument for psychiatric practice and research. Psychiatry Res. 1989;28(2):193-213. doi:10.1016/0165-1781(89)90047-4 2748771

[26] Devilly GJ, Borkovec TD. Psychometric properties of the credibility/expectancy questionnaire. J Behav Ther Exp Psychiatry. 2000;31(2):73-86. doi:10.1016/S0005-7916(00)00012-4 11132119

[27] Barnett S, Huckvale K, Christensen H, Venkatesh S, Mouzakis K, Vasa R. Intelligent Sensing to Inform and Learn (InSTIL): a scalable and governance-aware platform for universal, smartphone-based digital phenotyping for research and clinical applications. J Med Internet Res. 2019;21(11):e16399. doi:10.2196/16399 31692450 PMC6868504

[28] Brooke J. SUS: a quick and dirty usability scale. In: Jordan PW, Thomas B, Weerdmeester BA, McClelland IL, eds. *Usability Evaluation in Industry*. CRC Press; 1995:189.

[29] Zhou L, Bao J, Setiawan IMA, Saptono A, Parmanto B. The mHealth App Usability Questionnaire (MAUQ): development and validation study. JMIR Mhealth Uhealth. 2019;7(4):e11500. doi:10.2196/11500 30973342 PMC6482399

[30] Chen SY, Feng Z, Yi X. A general introduction to adjustment for multiple comparisons. J Thorac Dis. 2017;9(6):1725-1729. doi:10.21037/jtd.2017.05.34 28740688 PMC5506159

[31] Firth J, Torous J, Nicholas J, . The efficacy of smartphone-based mental health interventions for depressive symptoms: a meta-analysis of randomized controlled trials. World Psychiatry. 2017;16(3):287-298. doi:10.1002/wps.20472 28941113 PMC5608852

[32] Firth J, Torous J, Nicholas J, Carney R, Rosenbaum S, Sarris J. Can smartphone mental health interventions reduce symptoms of anxiety? a meta-analysis of randomized controlled trials. J Affect Disord. 2017;218:15-22. doi:10.1016/j.jad.2017.04.046 28456072

[33] Linardon J, Cuijpers P, Carlbring P, Messer M, Fuller-Tyszkiewicz M. The efficacy of app-supported smartphone interventions for mental health problems: a meta-analysis of randomized controlled trials. World Psychiatry. 2019;18(3):325-336. doi:10.1002/wps.20673 31496095 PMC6732686

[34] Baumel A, Muench F, Edan S, Kane JM. Objective user engagement with mental health apps: systematic search and panel-based usage analysis. J Med Internet Res. 2019;21(9):e14567. doi:10.2196/14567 31573916 PMC6785720

[35] Torous J, Nicholas J, Larsen ME, Firth J, Christensen H. Clinical review of user engagement with mental health smartphone apps: evidence, theory and improvements. Evid Based Ment Health. 2018;21(3):116-119. doi:10.1136/eb-2018-102891 29871870 PMC10270395

[36] Hutchesson MJ, Whatnall MC, Yazin N, . Health behavior interventions for university students measuring mental health outcomes: a scoping review. Front Public Health. 2022;10:1063429. doi:10.3389/fpubh.2022.1063429 36568797 PMC9771454

[37] Spijkerman MP, Pots WT, Bohlmeijer ET. Effectiveness of online mindfulness-based interventions in improving mental health: a review and meta-analysis of randomised controlled trials. Clin Psychol Rev. 2016;45:102-114. doi:10.1016/j.cpr.2016.03.009 27111302

[38] Baer R, Crane C, Miller E, Kuyken W. Doing no harm in mindfulness-based programs: conceptual issues and empirical findings. Clin Psychol Rev. 2019;71:101-114. doi:10.1016/j.cpr.2019.01.001 30638824 PMC6575147

[39] Harrer S, Shah P, Antony B, Hu J. Artificial intelligence for clinical trial design. Trends Pharmacol Sci. 2019;40(8):577-591. doi:10.1016/j.tips.2019.05.005 31326235

[40] Broglio K, Meurer WJ, Durkalski V, . Comparison of bayesian vs frequentist adaptive trial design in the Stroke Hyperglycemia Insulin Network Effort trial. JAMA Netw Open. 2022;5(5):e2211616. doi:10.1001/jamanetworkopen.2022.11616 35544137 PMC9096598

[41] Haller K, Becker P, Niemeyer H, Boettcher J. Who benefits from guided internet-based interventions? a systematic review of predictors and moderators of treatment outcome. Internet Interv. 2023;33:100635. doi:10.1016/j.invent.2023.100635 37449052 PMC10336165

