# Supplementary material for: Brief Digital Interventions for Psychological Distress: An AI-Enhanced Response-Adaptive Randomized Clinical Trial
Source: JAMA Netw Open. 2025 Oct 31;8(10):e2540502. doi: 10.1001/jamanetworkopen.2025.40502 (PMC12579342; doi:10.1001/jamanetworkopen.2025.40502)
Supplement: Supplement 1. — Trial Protocol and Statistical Analysis Plan [file jamanetwopen-e2540502-s001.pdf]

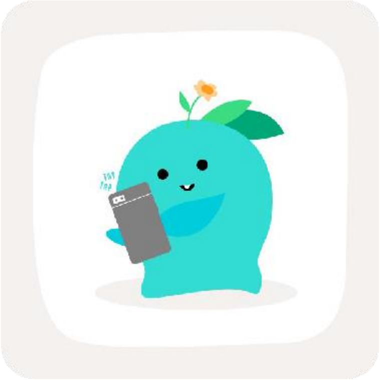

Clinical Trial Protocol

The Vibe Up Study:
An AI-response-adaptive randomised controlled trial of smartphone app-delivered self-care strategies for psychological distress in university students

**Protocol Id**	HC200466_CTP
**Version dated**	23 Jun 2022
**Principal investigator**	Professor Helen Christensen
**Grant number**	MRFAI000028
**RGO number**	RG200942
**UNSW HREC number**	HC200466
**Deakin University HREC number**	2021-098
**BDI project number**	187
**UNSW project finance code**	RG200942+RE939+BDI
**BDI project finance code**	UDOP
**Administrative contact**	Dr WuYi Zheng<br>Research Project Manager<br>wuyi.zheng@blackdog.org.au<br>+61 422 510 718

Contents

1 General information ... 6
2 Background information ... 10
2.1 Investigational products (ICH GCP 6.2.1) ... 10
2.2 Theoretical background (ICH GCP 6.2.2) ... 10
2.3 Risk(s) and benefit(s) (ICH GCP 6.2.3) ... 12
2.4 Route of administration, dosage, dosage regimen and treatment period(s) (ICH GCP 6.2.4) ... 13
2.5 Statement of compliance (ICH GCP 6.2.5) ... 13
2.6 Study population (ICH GCP 6.2.6) ... 14
3 Trial objectives and purpose (ICH GCP 6.3) ... 14
3.1 Overview ... 14
3.2 Research questions ... 14
4 Trial design ... 15
4.1 Design summary ... 15
4.2 Primary endpoint (ICH GCP 6.4.1) ... 15
4.3 Mini-trial architecture, sequence and duration (ICH GCP 6.4.5) ... 16
4.4 Randomisation procedures (ICH GCP 6.4.3) ... 19
4.5 Trial treatments (ICP GCH 6.4.4) ... 20
4.6 Stopping rules (ICH GCP 6.4.6) ... 21
4.7 Use of Case Report Forms (ICH GCP 6.4.9) ... 21
5 Selection and withdrawal of subjects ... 22
5.1 Subject inclusion criteria (ICH GCP 6.5.1) ... 22
5.2 Subject exclusion criteria (ICH GCP 6.5.2) ... 22
5.3 Trial sites ... 22
5.4 Subject recruitment procedures ... 22
5.5 Eligibility assessment method ... 23
5.6 Consent ... 24
5.7 Subject withdrawal ... 24
6 Treatment of subjects ... 26
6.1 Treatments to be administered (ICH GCP 6.6.1) ... 26
6.2 Management of known and potential risks ... 26
6.3 Medication(s)/treatment(s) permitted and not permitted before and/or during the trial (ICH GCP 6.6.2) 27
6.4 Procedures for monitoring subject compliance (ICH GCP 6.6.3) ... 27
6.5 Participant incentives ... 28
7 Assessment ... 28

7.1 Overview 28
7.2 Assessment of efficacy (ICH GCP 6.7) 28
7.3 Self-report questionnaires....28
7.4 Methods and timing for assessing, recording and analysis of study parameters 29
7.5 App-generated data 30
7.6 Assessment of safety (ICH GCP 6.8)....30
8 Statistics....33
8.1 Analysis of primary endpoint 33
8.2 Interim analyses 34
8.3 Additional analyses 34
8.4 Significance level 34
8.5 Planned sample size 34
8.6 Selection of subjects for analysis....35
8.7 Missing data 35
8.8 Confounding....35
8.9 Spurious data....36
9 Sub-studies 36
9.1 EMA sub-study 36
9.2 Digital phenotyping sub-study....37
10 Direct access to source data and documents (ICH GCP 6.10) 37
11 Quality assurance and quality control (ICH GCP 6.11) 38
11.1 Quality assurance 38
11.2 Quality control....38
12 Ethics (ICH GCP 6.12) 39
13 Data handling and record keeping (ICH GCP 6.13)....39
13.1 Applicable policies and procedures....39
13.2 Data Roles 40
13.3 Data Safety Monitoring Board....40
13.4 Data Collection and Storage Platforms....40
13.5 Data Retention and Disposal 41
13.6 Data Sharing....41
14 Financing and insurance (ICH GCP 6.14) 42
14.1 Funding details 42
14.2 Insurance....42
14.3 Clinical Trial Research Agreements 42
15 Publications policy (ICH GCP 6.15)....43

16 Essential documents 44
16.1 Trial documentation 44
16.2 Legal agreements and regulatory information 44
16.3 Standard Operating Procedures 44
16.4 Technology Requirements, Specifications and Procedures 45
17 Signatures 45
Appendices 46
Appendix 1 UNSW Human Research Ethics Committee Approval 46
Appendix 2 Deakin University Human Research Ethics Committee Approval 48
Appendix 3 UNSW Confirmation of Insurance Cover 49
Appendix 4 UNSW Medicine Letter of Support 50
Appendix 5 Deakin University Applied Artificial Intelligence Institute Letter of Support 51
Appendix 6 Recruitment Material 53
Appendix 7 Participant Information Statement and Consent Form 64
Appendix 8 Study Questionnaires 65
Appendix 9 Study Reminders 105
Appendix 10 Vibe Up Intervention Specifications 108
Appendix 11 Significant Safety Issue Notification Form 112
Appendix 12 Unexpected and Serious Adverse Event Notification Form 115
Appendix 13 Safety Monitoring Register 119
Appendix 14 Pre-Trial/Trial Initiation Monitoring and Reporting Procedure 120
Appendix 15 Delegations Log 125
Appendix 16 Training Policy 135
Appendix 17 Publications Policy 139
Appendix 18 Recruitment Strategy 164
Appendix 19 Mini-Trial Conduct and Monitoring Procedure 169
Appendix 20 Protocol Deviation Identification and Reporting Procedure 173
Appendix 21 Data Verification and Analysis Procedure 176
Appendix 22 Participant Psychological Safety Response Procedure 180
Appendix 23 Research Data Management Plan 191
Appendix 24 Data Safety Monitoring Board Terms of Reference and Procedures 198
Appendix 25 Vibe Up Algorithm Specification 204
Appendix 26 Optimise UNSW System Classification 211
Appendix 27 Optimise UNSW System Classification Approval 215
Appendix 28 Vibe Up App Software Requirements 216
Appendix 29 Vibe Up App Testing Plan 242

Appendix 30 Screening and Self-Report Questionnaire Testing Plan 248
Appendix 31 Systems Incident Identification and Response Protocol 253
Appendix 32 Case Report Form....271
Appendix 33 Research Partner Collaborative Research Agreement 315
Appendix 34 Commonwealth Standard Grant Agreement 328
References (ICP GCP 6.2.7)....369

1 General information

**Protocol title** (ICH GCP 6.1.1)	
**The Vibe Up Study: an AI-response-adaptive randomised controlled trial of smartphone app-delivered self-care strategies for psychological distress in university students**	
**Protocol Id**	HC200466_CTP
**Version dated**	23 Jun 2022
**Amendment history**	
Version date	Summary of changes
01 Dec 2020	Document created.
01 Mar 2021	SOPs finalised and inserted for sponsor review.
**Clinical trial sponsor** (ICH GCP 6.1.2)	
**Sponsor name**	UNSW Sydney
**Sponsor contact**	**Dr Ted Rohr**, Director of Research Ethics & Compliance Support, UNSW Sydney
**Telephone**	+61 417 844 054
**Email**	ted.rohr@unsw.edu.au
**Address**	Research Ethics and Compliance Support Unit, Level 3 Rupert Myers Building South Wing, UNSW Sydney, Kensington NSW 2052
**Personnel authorised to sign the protocol and the protocol amendment(s) for the Sponsor** (ICH GCP 6.1.3)	
**Name**	**Professor Helen Christensen**
**Telephone**	+61 2 9382 3717
**Email**	h.christensen@blackdog.org.au
**Address**	Black Dog Institute, Hospital Road, Randwick NSW 2031
**Human Research Ethics Committees (HREC)** (ICH GCP 6.12)	
Lead HREC	
**HREC name**	**UNSW HREC A**
**HREC contact**	Leonne Thompson, Human Research Manager, UNSW Sydney
**Telephone**	humanethics@unsw.edu.au
**Email**	+61 2 9385 6222
**Address**	Research Ethics and Compliance Support Unit, Level 3 Rupert Myers Building South Wing, UNSW Sydney, Kensington NSW 2052
Secondary HRECs	
**HREC name**	**Deakin University Human Research Ethics Committee** (DUHREC)
**HREC contact**	The Manager, Research Integrity, Deakin University
**Telephone**	+61 3 9251 7123
**Email**	research-ethics@deakin.edu.au
**Address**	Deakin Research Integrity, Deakin University, 221 Burwood Highway, Burwood VIC 3125
**Study Qualified Medical Expert** (ICH GCP 6.1.6)	
**Name**	**Dr Jill Newby**, Associate Professor and Clinical Psychologist, Black Dog Institute
**Telephone**	+61 2 9382 4217; +61 403 759 122
**Email**	j.newby@unsw.edu.au
**Address**	Black Dog Institute, Hospital Road, Randwick NSW 2031
**Sponsor's Independent Medical Expert** (ICH GCP 6.1.4)	
**Name**	**Prof Jennie Hudson**, Professor of Clinical Psychology, Black Dog Institute
**Telephone**	+61 408 660 098
**Email**	jennie.hudson@blackdog.org.au
**Address**	Black Dog Institute, Hospital Road, Randwick NSW 2031

**Clinical trial investigators** (ICH GCP 6.1.5)	
Principal Investigator (ICH GCP 1.34)	
**Name**	**Prof Helen Christensen**, Director and Chief Scientist, Black Dog Institute
**Telephone**	+61 2 9382 3717
**Email**	h.christensen@blackdog.org.au
**Address**	Black Dog Institute, Hospital Road, Randwick NSW 2031
**Trial site**	Black Dog Institute, UNSW Sydney
Subinvestigators (ICH GCP 1.56)	
**Name**	**Prof Svetha Venkatesh**, Alfred Deakin Laureate Professor and Co-Director of the Applied Artificial Intelligence Institute (A2I2), Deakin University
**Telephone**	+61 3 5227 2905
**Email**	svetha.venkatesh@deakin.edu.au
**Address**	KA Building Level 5, Deakin University, 75 Pigdons Road, Waurn Ponds VIC 3216
**Trial site**	Black Dog Institute, UNSW Sydney
**Name**	**Dr Kit Huckvale**, Research Fellow in Digital Health, Centre for the Digital Transformation of Health, The University of Melbourne
**Telephone**	+61 415 637 853
**Email**	kit.huckvale@unimelb.edu.au
**Address**	Level 13, Victorian Comprehensive Cancer Centre, The University of Melbourne. 305 Grattan Street, Melbourne VIC 3000
**Trial site**	Black Dog Institute, UNSW Sydney
**Name**	**Dr Jill Newby**, Associate Professor and Clinical Psychologist, Black Dog Institute
**Telephone**	+61 2 9382 4217; +61 403 759 122
**Email**	j.newby@unsw.edu.au
**Address**	Black Dog Institute, Hospital Road, Randwick NSW 2031
**Trial site**	Black Dog Institute, UNSW Sydney
**Name**	**Dr Jin Han**, Research Fellow, Black Dog Institute
**Telephone**	+61 2 9382 8509
**Email**	j.han@blackdog.org.au
**Address**	Black Dog Institute, Hospital Road, Randwick NSW 2031
**Trial site**	Black Dog Institute, UNSW Sydney
**Name**	**Dr WuYi Zheng**, Optimise Research Project Manager, Black Dog Institute
**Telephone**	+61 422 510 718
**Email**	wuyi.zheng@blackdog.org.au
**Address**	Black Dog Institute, Hospital Road, Randwick NSW 2031
**Trial site**	Black Dog Institute, UNSW Sydney
**Name**	**Prof Andrew Mackinnon**, Visiting Professor of Biostatistics, Black Dog Institute
**Telephone**	+61 403 923 129
**Email**	andrew.mackinnon@biostats.com.au
**Address**	Black Dog Institute, Hospital Road, Randwick NSW 2031
**Trial site**	Black Dog Institute, UNSW Sydney
**Name**	**Dr Sunil Gupta**, Associate Professor, Applied Artificial Intelligence Institute (A2I2), Deakin University
**Telephone**	+61 3 522 73109
**Email**	sunil.gupta@deakin.edu.au
**Address**	KA Building Level 5, Deakin University, 75 Pigdons Road, Waurn Ponds VIC 3216

**Trial site**	Black Dog Institute, UNSW Sydney
**Name**	**Ms Manisha Senadeera**, Associate Research Fellow, Applied Artificial Intelligence Institute (A2I2), Deakin University
**Telephone**	+61 3 522 78902
**Email**	manisha.senadeera@deakin.edu.au
**Address**	KA Building Level 5, Deakin University, 75 Pigdons Road, Waurn Ponds VIC 3216
**Trial site**	Black Dog Institute, UNSW Sydney
**Name**	**Dr Truyen Tran**, Associate Professor, Applied Artificial Intelligence Institute (A2I2), Deakin University
**Telephone**	+61 3 522 78744
**Email**	truyen.tran@deakin.edu.au
**Address**	KA Building Level 5, Deakin University, 75 Pigdons Road, Waurn Ponds VIC 3216
**Trial site**	Black Dog Institute, UNSW Sydney
**Name**	**Prof Kon Mouzakis**, Co-Director, Applied Artificial Intelligence Institute (A2I2), Deakin University
**Telephone**	+61 419 308 603
**Email**	kon.mouzakis@deakin.edu.au
**Address**	BC Building Level 7, Deakin University, 221 Burwood Highway, Burwood VIC 3125
**Trial site**	Black Dog Institute, UNSW Sydney
**Name**	**Prof Rajesh Vasa**, Head of Translational Research, Applied Artificial Intelligence Institute (A2I2), Deakin University
**Telephone**	+61 435 016 006
**Email**	rajesh.vasa@deakin.edu.au
**Address**	BC Building Level 7, Deakin University, 221 Burwood Highway, Burwood VIC 3125
**Trial site**	Black Dog Institute, UNSW Sydney
**Name**	**Dr Scott Barnett**, Senior Research Fellow, Applied Artificial Intelligence Institute (A2I2), Deakin University
**Telephone**	+61 3 924 45018
**Email**	scott.barnett@deakin.edu.au
**Address**	BC Building Level 7, Deakin University, 221 Burwood Highway, Burwood VIC 3125
**Trial site**	Black Dog Institute, UNSW Sydney
**Name**	**Dr Leonard Hoon**, Senior Research Fellow, Applied Artificial Intelligence Institute (A2I2), Deakin University
**Telephone**	+61 3 924 68012
**Email**	leonard.hoon@deakin.edu.au
**Address**	BC Building Level 7, Deakin University, 221 Burwood Highway, Burwood VIC 3125
**Trial site**	Black Dog Institute, UNSW Sydney
**Name**	**Dr Rena Logothetis,** Associate Research Fellow, Applied Artificial Intelligence Institute (A2I2), Deakin University
**Telephone**	+61 403 560 034
**Email**	rena.logothetis@deakin.edu.au
**Address**	BC Building Level 7, Deakin University, 221 Burwood Highway, Burwood VIC 3125
**Trial site**	Black Dog Institute, UNSW Sydney
**Name**	**Mr Stuart Cameron,** Principal Software Engineer, Applied Artificial Intelligence Institute (A2I2), Deakin University
**Telephone**	+61 3 9918 9002
**Email**	stuart.cameron@deakin.edu.au
**Address**	BC Building Level 7, Deakin University, 221 Burwood Highway, Burwood VIC 3125

**Trial site**	Black Dog Institute, UNSW Sydney
**Name**	**Prof Henry Cutler**, Director, Macquarie University Centre for the Health Economy (MUCHE), Macquarie University
**Telephone**	+61 2 9850 2998
**Email**	henry.cutler@mq.edu.au
**Address**	Suite 3, EMC2 Building Level 1, Sydney NSW 2109
**Trial site**	Black Dog Institute, UNSW Sydney
**Name**	**Dr Liz Schroeder**, Associate Professor, Macquarie University Centre for the Health Economy (MUCHE), Macquarie University
**Telephone**	+61 2 9850 2468
**Email**	liz.schroeder@mq.edu.au
**Address**	Suite 3, EMC2 Building Level 1, Sydney NSW 2109
**Trial site**	Black Dog Institute, UNSW Sydney
Administrative contact	
**Name**	**Dr WuYi Zheng**, Optimise Research Project Manager, Black Dog Institute
**Telephone**	+61 422 510 718
**Email**	wuyi.zheng@blackdog.org.au
**Address**	Black Dog Institute, Hospital Road, Randwick NSW 2031
**Trial site**	Black Dog Institute, UNSW Sydney
**Pharmacy and/or clinical laboratory and other medical and/or technical departments involved in the Trial** (ICH GCP 6.1.7)	
(Not applicable to this trial.)	

2 Background information

This section additionally fulfills the function of the Investigator's Brochure (ICH GCP 7).

2.1 Investigational products (ICH GCP 6.2.1)

Investigational product	Approval status in Australia
Vibe Up Mindfulness (Self-guided, app-delivered intervention)	Not regulated*
Vibe Up Physical Activity (Self-guided, app-delivered intervention)	Not regulated*
Vibe Up Sleep Hygiene (Self-guided, app-delivered intervention)	Not regulated*

* The primary purpose of the Vibe Up investigational digital products is to alleviate psychological distress. However, because psychological distress is not a disease, injury or disability, none qualify as Software as a Medical Device (SaMD). They are therefore not regulated by the Therapeutic Goods Administration and this clinical trial is not notifiable under the Clinical Trial Notification (CTN) or Approval (CTA) schemes.

The content of the Vibe Up interventions is specified in **Appendix 10**.

2.2 Theoretical background (ICH GCP 6.2.2)

2.2.1 Vibe Up Mindfulness

Vibe Up Mindfulness is a self-guided mindfulness-based training intervention (MBI). Mindfulness is the "awareness that emerges through paying attention on purpose, in the present moment, and non-judgementally to the unfolding of experience moment by moment." MBI training is intended to promote habitual mindfulness practice and can be instructor-facilitated or self-guided (e.g. using a course of audio recordings to learn mindfulness techniques) [1]. It appears to work by producing sustained changes in attentional control, emotion regulation, sensory awareness and self-awareness[2]. The mechanisms of MBIs are distinct from relaxation training[3].

In a 2019 meta-analysis of 40 randomized controlled trials[4], MBIs were associated with significant, small-medium improvements in psychological distress compared to active control (SMD = 0.37 favouring MBI, 95% CI 0.18-0.56, pooled n = 437 students) and, consistent with two earlier meta-analyses[5, 6], significant improvements in state anxiety compared to active control (SMD of 5.95 favouring MBI on the State-Trait Anxiety Inventory State Subscale, 95% CI 2.41-9.49, pooled n = 105). Although existing randomized controlled trials were of poor-moderate quality, most findings were robust to sensitivity analyses excluding low quality studies[4]. Effects appear to be sustained for at least three months, are seen when MBIs are offered as stand-alone interventions[6] (rather than combined with other treatment approaches), and do not appear to be contingent on intervention duration, suggesting that even brief MBIs (e.g. consisting of brief daily practice lasting fewer than 2 weeks[4]) can produce sustained improvements in distress symptoms.

Self-help MBIs based on audio-visual training content are acceptable, with approximately two thirds of participants completing follow-up measures in intervention studies based on these techniques [1]. Web-based delivery of digital self-guided MBIs is now an established method [7]. Evidence supporting the use of apps is still developing. A 2019 RCT of n=88 university students found significant moderately sized improvements in stress, mindfulness and self-compassion using a self-guided MBI smartphone app for 8 weeks [8]. Post-intervention, the majority of participants were satisfied with using an app and were prepared to recommend it to peers.

2.2.2 Vibe Up Physical Activity

A combination of trial meta-analytic and observational data supports the thesis that physical exercise can have positive impacts on the mental health of young adults including, specifically, reductions in psychological distress. In non-clinical adult populations, a 2015 meta-meta-analysis of 92 studies found a consistent moderate effect of physical exercise in reducing depressive symptoms (SMD of 0.50 favouring physical activity, 95% CI 0.05 to 0.93) and a small consistent effect on anxiety (SMD of 0.38 favouring physical activity, 95% CI 0.1 to 0.66 [9].) In teenagers, a 2006 meta-analysis of 16 heterogenous randomized controlled trials of structured physical activity interventions found a moderate effect on depressive symptoms (SMD of 0.66 favouring physical activity, 95% CI 0.08-1.25), regardless of exercise intensity[10]. A 2020 cross-sectional study of 1095 university students found higher levels of physical activity were inversely correlated with psychological distress (and positively correlated with resilience and emotion regulation[11].) The effects of physical activity on mental health are theorised to arise from a combination of modification of hormonal stress responses, reduced sympathetic nervous and immune system activation and direct changes in the brain[12]. Observed increases in neurotransmitter and neurotrophic factor production may directly improve brain function and consequently mood[13].

The Vibe Up Physical Activity intervention is based around a brief, evidence-based seven-minute high-intensity circuit training (HICT) protocol using bodyweight resistance[14]. HICT delivered as 3-4 six- or seven-minute sessions per week appears to prompt modest improvements in muscular endurance[15, 16], although benefits to physiological parameters, such as blood pressure, and body composition are unclear[16-18]. No studies have directly assessed the impact of HICT on mood or psychological distress in university students. Supporting the concept of a *brief* exercise intervention targeting mental health are the meta-analytic findings that short-duration exercise (e.g. 10-30 minutes) can positively impact mood, and that there is no additional benefit of increasing exercise duration beyond 30 minutes[12]. A 2018 RCT of 24 untrained adults aged 18-40 found that four weeks of daily HICT resulted in significant improvements in self-rated quality of life measured using the SF-36 Health Survey[19], which incorporates aspects of mental health[20]. The Vibe Up Physical Activity intervention also includes educational information on increasing incidental exercise and step count, to cater to participants of varying fitness levels.

App-delivered, self-guided exercise interventions appear to be modestly effective in increasing physical activity, such as average steps taken per day, particularly in the short-term[21]. Apps targeting physical activity alone are more effective than those combining exercise with other strategies, e.g. diet modification[21].

2.2.3 Vibe Up Sleep Hygiene

Sleep hygiene refers to the set of daily living activities that are necessary to maintain good quality sleep and full daytime alertness[22]. Although there are recognised common determinants of poor sleep relating to arousal (e.g. caffeine ingestion) and sleep organisation (e.g. excessive bedtime variation), the activities that influence sleep either positively or negatively substantially vary from individual to individual[22]. The purpose of sleep hygiene education (SHE) is to help individuals identify the specific behaviours and habits that promote their own sleep and implement these, while eliminating/reducing those that disturb sleep[23]. Vibe Up Sleep Hygiene is a brief, self-guided SHE which additionally incorporates elements of stimulus control which seeks to reduce, for those with sleep problems, anxiety or conditioned arousal associated with going to bed[24]. Although well-theorised, there is some evidence that SHE may not be substantially more effective than psychological placebos in improving subjective sleep quality (the impact of placebos on other outcomes is currently unclear)[25].

While the relationship between sleep and mental health is likely bidirectional[26], a combination of meta-analytic, trial and observational evidence supports the thesis that insomnia contributes to psychological distress in young adults, that this can be modified in the short-term, and that SHE is an effective intervention. Serial observational studies in Canadian students have confirmed that sleep quality is an independent predictor of improved positive affect, reduced negative affect and reduced stress on the following day[27]. A 2019

prospective study of 1638 Norwegian adults over 1 year found that nicotine use, disturbance and sleep schedule were significantly associated with persistent (but not new onset) insomnia[28]. In a 2018 cross-sectional study of 4823 Chinese teenagers[29], after accounting for sleep problems, sleep schedule was independently associated with mental health problems measured using the Strengths and Difficulties Questionnaire[30].

A 2018 meta-analysis of sleep interventions for college students identified small-moderate effects (SMD of 0.40 favouring SHE) on mental health of SHE in the small number of heterogeneous studies (n=3) reporting a relevant outcome[31]. Structural equation modelling of the impact of SHE in n=218 US college students identified both significant direct and indirect effects of SHE on depression and subjective well-being (mediated by sleep quality [26].) Unmanaged insomnia is a known significant predictor of subsequent depression (OR 2.83, 95% CI 1.55-5.17), alcohol abuse (OR 1.35, 95% CI 1.08-1.67) and psychosis (OR 1.28, 95% CI 1.03-1.59) [32]. Brief sleep hygiene appears to be effective in promoting modest improvements in self-rated insomnia. A 2018 meta-analysis of SHE in adults found an average improvement of 4.79% in self-rated sleep efficiency (95% CI 2.66-6.78) and 1.75 points on the Pittsburgh Sleep Quality Index[33] (scale range 0-21, 95% CI 1.05-2.45) across the 9 studies that reported this[23].

The use of apps to delivery insomnia interventions, including SHE, is now established. A 2017 systematic review identified 35 apps offering self-guided insomnia support[34].

2.3 Risk(s) and benefit(s) (ICH GCP 6.2.3)

Controls for known risks are provided in **Table 6.2**.

**Vibe Up Mindfulness**	
**Known risk(s)**	• Moderate risk (in 17% of people undertaking focussed attention-type meditation [13]) of transient anxiety symptoms or unusual perceptions (e.g. heart rate awareness, depersonalisation) during mindfulness meditation.
**Potential risk(s)**	• Risk of inconvenience/contribution to psychological distress if intervention is ineffective in alleviating psychological distress symptoms.
**Potential benefit(s)**	• Reduction in psychological distress [4].<br>• Reduction in state anxiety [4].
**Vibe Up Physical Activity**	
**Known risk(s)**	• Moderate risk of bronchoconstriction (up to 20% of those without known asthma [35]) in those with uncontrolled asthma/undiagnosed exercise-induced bronchoconstriction.<br>• Small risk (approximately 1.5 per 1000 'keep fit' type sessions similar to the included 7-minute workout [36]) of acute musculoskeletal injury or other acute exercise-related morbidity associated with any physical exercise.<br>• Very small risk (<1 event per 500,000 hours of exercise [37]) of an acute cardiac event associated with any unaccustomed vigorous physical exercise.
**Potential risk(s)**	• Risk of inconvenience/contribution to psychological distress if intervention is ineffective in alleviating psychological distress symptoms.
**Potential benefit(s)**	• Reduction in psychological distress.<br>• Improvement to exercise tolerance.<br>• (If sustained) improvements to cardiovascular health and reduced risk of cardiovascular/metabolic disease.
**Vibe Up Sleep Hygiene**	
**Known risk(s)**	• None known.
**Potential risk(s)**	• Risk of inconvenience/contribution to psychological distress if intervention is ineffective in alleviating psychological distress symptoms.
**Potential benefit(s)**	• Reduction in psychological distress.

	• Improvement to subjective sleep quality.<br>• Improvements to concentration and alertness.

2.4 Route of administration, dosage, dosage regimen and treatment period(s) (ICH GCP 6.2.4)

**Vibe Up Mindfulness**	
**Route of administration**	App-delivered self-guided informational/behavioural intervention
**Nominated dosage**	3 to 5-minute audio-recorded mindfulness exercise. Five available modules.
**Expected dosage regimen**	Minimum one mindfulness exercise completed daily at participant's convenience. All modules completed at least once by end of treatment period.
**Treatment period**	2 weeks
**Justification**	Self-help delivery methods are not unusual for MBIs: n=11/40 (22%) intervention RCTs in a recent meta-analysis used this approach[4]. Nor is a brief intervention duration: in the same meta-analysis n=13/40 (32%) were delivered for 2 weeks or less[4]. Importantly, treatment duration does not appear to influence MBI effectiveness. Web-based digital delivery of MBIs is an established technique[7]. Further, limited evidence suggests that app-delivered MBIs can be effective and acceptable[8]. The total expected practice time of approximately 70 minutes (5 x 14) lies within the range observed in existing RCTs (10 – 1650 minutes) [4].
**Vibe Up Physical Activity**	
**Route of administration**	App-delivered self-guided informational/behavioural intervention
**Nominated dosage**	7-minute structured workout or participant-selected goal-focussed physical activity (e.g. increasing daily step count, duration unspecified.)
**Expected dosage regimen**	Physical activity completed daily at participant's convenience, including 7-minute work out three times weekly or, alternatively, increasing steps by 10% weekly.
**Treatment period**	2 weeks
**Justification**	App-delivery of self-guided physical activity interventions is an established administration technique [21]. Brief physical exercise (e.g. 10-30 minutes) is clearly effective in improving mood, with no additional benefits of longer intervention durations [12], suggesting that a 7-minute workout will have at least some positive impacts on mood. A thrice-weekly HICT protocol is a standard approach[15, 18], allowing time for physiological recovery between sessions. For exercise apps, brief treatment periods (less than 3 months) appear to be more effective than longer durations [21].
**Vibe Up Sleep Hygiene**	
**Route of administration**	App-delivered self-guided informational/behavioural intervention
**Nominated dosage**	Participant-structured sleep hygiene activity (5-30 minutes). Three available education modules (3-5 minutes).
**Expected dosage regimen**	Minimum one sleep hygiene activity completed daily before bedtime. All modules completed at least once by end of the treatment period.
**Treatment period**	2 weeks
**Justification**	App-delivery of self-guided sleep support, including SHE, is an established administration technique[34]. Individual self-help is a common approach, seen in n=5/15 (33%) of a recent meta-analysis of SHE[23]. The number of available education modules matches the median number of sessions (n=3) identified in the same meta-analysis[23]. Brief treatment periods (two weeks or less) are not unusual in SHE: used in n=4/15 (27%) studies [23].

2.5 Statement of compliance (ICH GCP 6.2.5)

The clinical trial will be conducted in compliance with the following guidelines and documentation:

- ICH Guidelines for Good Clinical Practice (GCP)

- National Statement on Ethical Conduct in Human Research (National Statement)
- This Clinical Trial Protocol, as approved by:
  - The Human Research Ethics Committees (HRECs) responsible for monitoring the conduct of the trial; and
  - The Clinical Trial Sponsor.
- Standard Operating Procedures for trial conduct detailed in **Table 16.3** and as approved by the Clinical Trial Sponsor.
- The Clinical Trial Sponsor's on-site monitoring procedures, as defined/required by the Sponsor (ICH GCP 5.18).

The trial will be registered with the Australian and New Zealand Clinical Trials Registry (ANZCTR).

2.6 Study population (ICH GCP 6.2.6)

Adult university students aged 18 or older; resident in Australia; currently enrolled at an accredited Australian higher education institution; and experiencing elevated psychological distress (self-rated Kessler Psychological Distress score of ≥20 at the time of study entry.)

3 Trial objectives and purpose (ICH GCP 6.3)

3.1 Overview

The Vibe Up study is a pragmatically-oriented, decentralised AI-adaptive group sequential randomised controlled trial (RCT) comparing the effectiveness of receiving one of three brief, two week digital self-guided interventions (physical activity, mindfulness or sleep hygiene) or active control in reducing self-reported psychological distress (DASS-21 normalised total score) in Australian university students.

The primary purpose of the study is to determine whether intervention effect sizes can be identified (efficiently) using a multi-arm contextual bandit-based AI-adaptive trial method (see **RQ1** and **RQ3**), and whether estimates are consistent with estimates derived from prior research using traditional trial methods (see **RQ2**.) In addition, the study will explore whether baseline severity is associated with differences in intervention effect by incorporating baseline severity into the model of intervention effectiveness created by the AI algorithm.

The study also incorporates sub-studies focussing on assessing resilience to negative affect using ecological momentary assessment (EMA, see **RQ6-8**) and exploring the potential for smartphone-based passive sensing of activity (digital phenotyping, see **RQ9**) to predict changes in distress symptoms.

3.2 Research questions

- RQ1: Can an AI-adaptive sequential randomized controlled trial method comparing one of three alternative brief digital self-help intervention strategies (mindfulness, physical exercise or sleep hygiene) and an active control identify the most effective strategy(ies) for reducing self-reported mild or more severe psychological distress (K-10 score ≥ 20 at screening) amongst Australian university students?
- RQ2: Can this method rediscover an expected, *a priori* defined, binary partition of the selected interventions into 'likely more effective' (physical activity and mindfulness) and 'likely less effective' (sleep hygiene and active control) for reducing self-reported psychological distress?
- RQ3: How does the total number of involved participants and overall trial duration compare with a hypothetical (*a priori* defined) traditional four-arm randomized controlled trial using the same interventions?

- RQ4: Can a context-vector based variant of this method identify differences in intervention effectiveness according to baseline distress symptoms (measured using the DASS-21)?
- RQ5: Are any specific demographics, self-reported psychological factors, or personality traits (or combinations of these) associated with intervention response amongst university students?
- RQ6: Are individual differences in self-reported likelihood to respond to feelings (and proposed behaviours), measured using EMA, associated with self-reported psychological distress and baseline suicidality amongst university students?
- RQ7: Are any specific patterns of positive/negative affect (PA/NA) rhythms, measured using EMA, associated in university students with:
    - Psychological distress, exercise or sleep quality; and/or
    - Intervention response.
- RQ8: How does a one-week versus three-week EMA protocol (in the active control condition) compare on compliance and reactivity?
- RQ9: Are any digital phenotyping-derived behavioural and cognitive markers associated with:
    - Within-individual changes in self-reported psychological distress, comparing mid and post assessments in the mindfulness, sleep hygiene, physical activity and active control conditions; and/or
    - Within-individual changes in self-reported psychological affect collected using EMA; and/or
    - Intervention compliance based on self-report and/or app instrumentation data?

4 Trial design

4.1 Design summary

A pragmatically-oriented, decentralised AI-adaptive group sequential randomised controlled trial (RCT) comparing the effectiveness of receiving one of three brief, two week digital self-guided interventions (physical activity, mindfulness or sleep hygiene) or active control in reducing self-reported psychological distress (DASS-21 normalised total score) in Australian university students.

Comparison will be made between those reporting none/mild, moderate and severe/extremely severe overall symptom burden at baseline.

Statistical details including planned sample size and multiple-testing adjustment are provided in **8**.

4.2 Primary endpoint (ICH GCP 6.4.1)

The primary endpoint is the change in *total score* on the self-reported Depression, Anxiety and Stress Scale - 21 item version[38] (DASS-21) between the mid (pre-intervention) and the post (post-intervention) timepoints. Lower overall scores represent reduced symptom burden.

Per the DASS Manual[39], Z-score normalisation is the recommended procedure when wishing to use the DASS total score rather than its component subscale scores. The normalised total score is calculated as the arithmetic mean of subscale z-scores. Each subscale z-score is calculated by subtracting from the participant raw subscale total score the population mean and dividing the result by the population standard deviation. To do this, we will use the population values provided in the DASS manual.

4.3 Mini-trial architecture, sequence and duration (ICH GCP 6.4.5)

4.3.1 Conceptual overview

In an AI-adaptive group sequential RCT, the study proceeds as a series of discrete "mini-trials", each recruiting a restricted sample of participants who are allocated to study intervention/control arms under the control of an AI algorithm. In a *contextual* AI-adaptive study, learning is conditioned on a pre-specified set of characteristics (e.g. demographic properties) such that trial analyses can explore questions of the form "What works for whom?"

Adaptive allocation provides a mechanism for the study to be adjusted 'on the fly' to maximise the chance that one or more pre-specified optimisation goals can be satisfied (see **4.3.2**). The selection of appropriate optimisation goals is problem and domain-specific. Examples can include minimising the overall number of participants allocated to ineffective interventions, or, alternatively, maximising the chance that desired statistical comparisons can be made with appropriate statistical power. In Vibe Up, optimisation is directed towards the latter goal for the primary planned analyses of the trial (see **4.3.2**).

After each mini-trial, the algorithm uses the observed sample data to update an underlying model of effects attributable to each intervention and determine how the study will proceed. The trial can then either:

- Stop, if interim analyses (see **8.2**) confirm that all optimisation goals have been satisfied.
- Continue with another mini-trial. The updated model is used to allocate participants to each subsequent trial consistent with the pre-specified goals.

In the Vibe Up Study, the algorithm under evaluation is a contextual, Gaussian process-based multi-arm bandit[40] (cMAB, see **4.3.4**).

Mini-trials and cMAB operation will be conducted according to the **Mini-Trial Conduct and Monitoring Procedure** (see **Appendix 19**).

4.3.2 Optimisation goals

For each clinical severity group, after blindly rank-ordering all intervention arms including active control by their observed mean primary endpoint scores (given that lower is better, see **4.2**) such that $aa_0 < aa_1 < aa_2 < aa_3$:

- Goal 1: Find the best. Identify with the smallest number of mini-trials/participants possible the best performing intervention arm, such that for all pairwise comparisons $aa_0 < aa_1$, $aa_0 < aa_2$ and $aa_0 < aa_3$ the null hypothesis of no difference can be rejected.
- Goal 2: Find the next best. Should Goal 1 be satisfied (best-performing intervention aa_0 having been identified and fixed), find with the smallest number of mini-trials/participants possible the next best intervention arm, such that for both pairwise comparisons $aa_1 < aa_2$ and $aa_1 < aa_3$ the null hypothesis of no difference can be rejected.
- Goal 3: Find the third best. Should Goal 2 be satisfied (second best-performing intervention having been identified and fixed), and further mini-trials remain, trial will continue to find with the smallest number of mini-trials/participants possible the next best intervention arm, such that for the pairwise comparison $aa_2 < aa_3$ the null hypothesis of no difference can be rejected.

Optimisation goals will be formally evaluated as part of planned interim analyses (see **8.2**).

To identify robust estimates of the effect sizes of the most effective intervention(s) as quickly as possible while simultaneously minimising the numbers of participants allocated to likely-ineffective interventions (consistent with the pragmatic effectiveness orientation of the trial).

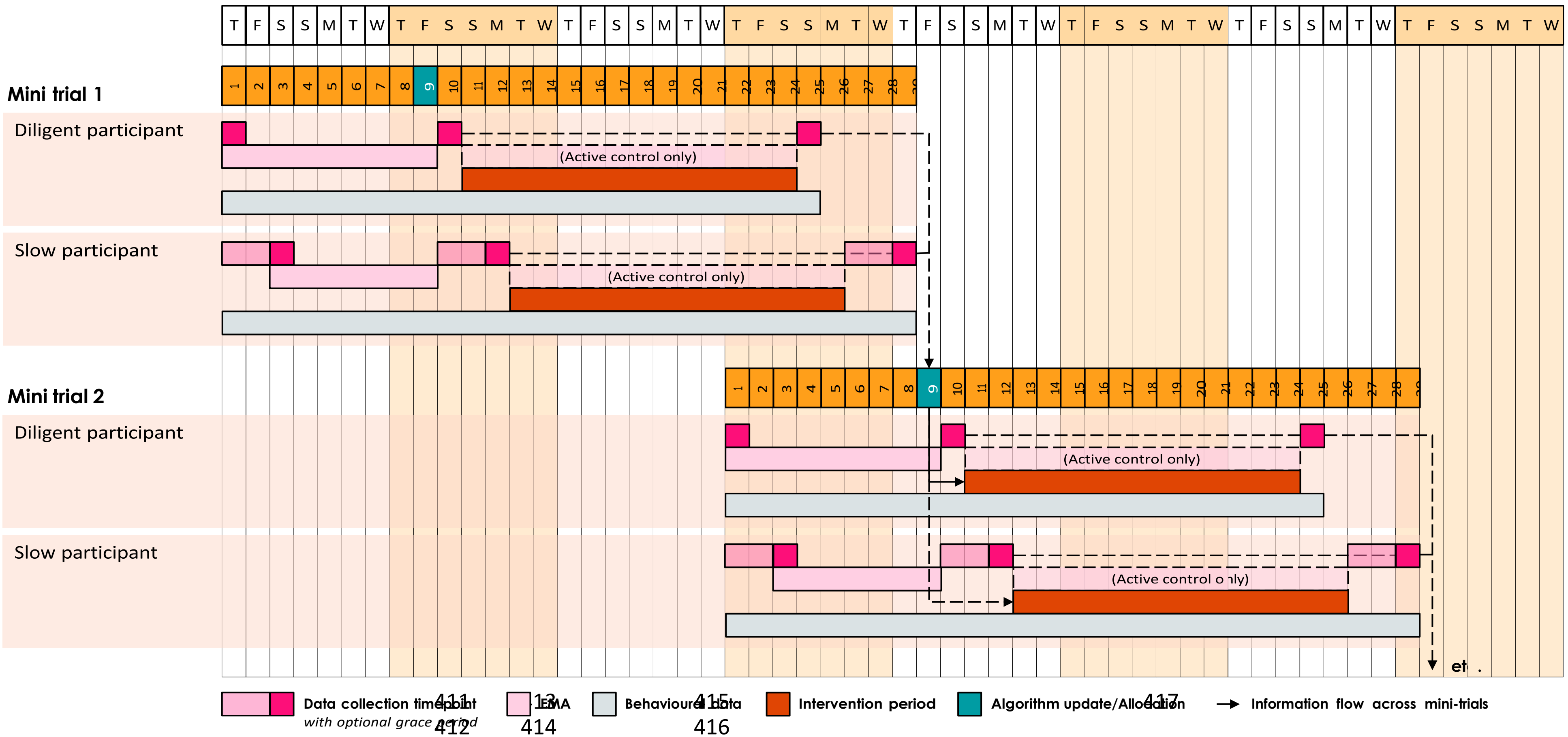

Figure 1 - Mini-trial sequencing

4.3.3 Sequence and duration

Up to 12 mini-trials will be conducted (3 pilot, 9 roll-out). Every participant completes exactly one mini-trial, once.

Each mini-trial will last exactly 4 weeks and 1 day, starting and ending on a Thursday. Mini-trials can occur with a maximum frequency of one every 3 weeks, but spacing can be arbitrarily increased if necessary (e.g. for technical troubleshooting). Under 3-week spacing, algorithm update plus allocation *must* occur on Day 30/Day 9 of each mini-trial pair (see **Figure 1**) to avoid disrupting the timeline/sequencing of participants.

Each mini-trial consists of two periods:

- A 10-day *baseline measurement period* incorporating questionnaire batteries on Day 1 (baseline) and Day 10 (mid) plus EMA (Days 1-9); and
- A 14-day *intervention period* (Days 11-26; extra days allowing for the 72 hour flexibility to complete questionnaires) plus post questionnaire (Days 25-29).
- Invitations to app download/baseline surveys sent via SMS and email to all participants on Day 1 of the mini-trial, with up to 72 hours to complete app installation and baseline survey.
- Up to 72 hours is allowed to complete each of the baseline, mid and post assessments, meaning that each participant may have slightly different timings for start/finish even though the overall mini-trial start/finish dates are fixed. **Figure 1** provides examples of the extremes for participants who complete tasks as soon as asked (diligent participant) and those who do not respond until the last minute to complete tasks (slow participant).
- The initial EMA period ends on Day 9 for *all participants* in a given mini-trial regardless of whether they start on time or not (e.g. early starting participants may do more days of EMA). This is to provide a fixed point in time for algorithm update allocation to occur (on Day 9 Wednesday), allowing mini-trials to be chained together efficiently.

The mini-trial flow for individual participants is illustrated in **Figure 2** (see page 25.) As soon as a participant completes the baseline questionnaire battery they immediately advance to the next step of the study (i.e. EMA). After a participant completed the mid questionnaire battery, they advanced to the next step of the study (i.e. intervention) the following day:

- In the intervention phase, participants are allocated to one of 3 self-guided app-based interventions or active control.

Eight weeks after the mini-trial ends, participants who completed the mid questionnaires will be invited to complete a follow-up questionnaire battery (follow-up) online, via Qualtrics (see **4.5.3**).

4.3.4 Contextual multi-arm bandit algorithm

The multi-arm bandit algorithm used in Vibe Up has the following principal characteristics:

- R*eward distributions* (i.e. models of intervention effects) are modelled on a per-arm basis using Gaussian Processes (GP) with a zero mean function and squared exponential covariance function/kernel.
- *Per-participant reward* is calculated as the within-individual mid (pre-intervention) to post-intervention difference in normalised total DASS score (see **4.2**). This normalisation step is intended to account for baseline differences. Participants with missing data from either the mid or post DASS surveys will be omitted from the optimisation process (but not from planned interim and trial analyses.)
- In mini-trial 1, participants will be allocated at random to each of the four arms with a uniform probability of 0.25 (i.e. an allocation ratio of 1:1:1:1).

- In subsequent trials, an Upper Confidence Bound (UCB) *acquisition function* is used to deterministically identify the intervention to which participants will be allocated. UCB balances exploitation and exploration by selecting for evaluation arms as a combination of high mean performance and/or high uncertainty (reflected by wider confidence bounds.)
- For Vibe Up, the MAB *context vector* will consist simply of the normalised total DASS score at baseline, treating this as a continuous real-valued number. No other demographic characteristics will be used to define context.

Assessment of whether optimisation goals have been satisfied (including any decision to reconfigure the algorithm to focus on Optimisation Goal 2) will be made offline as part of interim analyses (see **8.2**).

All MAB algorithm input variables are collected using the study app from the baseline, mid and post questionnaires (see **7.3**).

4.4 Randomisation procedures (ICH GCP 6.4.3)

4.4.1 Allocation

Computerised allocation will be performed automatically by the Optimise platform on Day 30/Day 9 of each mini-trial pair.

An initial allocation ratio (mini-trial 1) of 1:1:1:1 (three interventions plus active control) will be used. Subsequent allocation will be determined on a per-individual basis, conditioned on the context offered by baseline DASS scores, by the MAB algorithm as part of its designed function (see **Appendix 25**).

There will be no minimum per-arm allocation (One of the consequences of variable allocation under the MAB scheme is that allocation to any given arm may tend to zero.)

4.4.2 Sequence generation

Sequence generation for mini-trial 1 (e.g. for random assignment into blocks for stratification and subsequent random sampling) will be based on computer-generated random numbers.

4.4.3 Blinding

The following groups will be unblinded (because the nature of the interventions mean that they cannot easily be concealed):

- Participants.
- Operational staff involved in day-to-day participant interactions.

The following groups will be blinded:

- Investigators and other trial staff involved in algorithm operations/data analysis because of the risk of bias to trial outcomes if either are affected by knowledge of allocation/intervention performance.
- All other trial staff unless necessary for trial operations since there is no good requirement to know allocation otherwise.

All study assessments are via electronic self-report. There are no outcome assessors.

4.4.4 Concealment mechanism for blinded study staff

Allocation concealment will be guaranteed by:

- Preventing access by blinded study staff to the Optimise computer system holding randomisation information; and
- Breaking randomization codes only once primary data analysis is complete (see **4.4.5**).

4.4.5 Maintenance of trial treatment randomization codes and procedures for breaking codes (ICH GCP 6.4.8)

Randomization codes will be generated and retained automatically by the Optimise platform.

Responsibility for holding and securing the randomisation list and performing any code break will be assigned in the **Delegations Log** (see **Appendix 15**). The list holder cannot be the Principal Investigator or any other staff member involved in:

- day-to-day trial operations; or
- primary data analysis.

Because individual participants will be aware of, and able to disclose, the intervention to which they were allocated, codes may be broken only in the following situations:

- On completion of the primary data analysis, certified as complete by the Principal Investigator; or
- If required by the trial **Data Safety Monitoring Board** (see **13.2**) in writing to the Principal Investigator.

Any code break shall be recorded in the **Safety Monitoring Register** (see **Appendix 13**) and reported to the Sponsor per **7.6.5**.

4.5 Trial treatments (ICP GCH 6.4.4)

4.5.1 Digital self-guided interventions

All three Vibe Up treatments are brief, app-delivered, self-guided digital informational/behavioural interventions. Each intervention is entirely separate but consists of a combination of:

- Brief modular information covering key concepts, delivered e.g., as infographics.
- Structured activities, e.g. practising mindfulness with guided audio.

The interventions are fully described in the **Vibe Up Intervention Specifications** (**Appendix 10**).

All interventions are self-guided and delivered via the Vibe Up app installed on participants' smartphones. The study app also delivers questionnaires and EMA. Further details of the treatments to be administered, dosing schedules, routes of administration and treatment period are specified in **Tables 2.2** and **2.4**.

After completion of the mid questionnaire, participants will be invited automatically by the study app to undertake one of three self-guided interventions (or active control) targeting one of: physical activity, mindfulness or sleep hygiene. Each participant will be allocated to receive exactly one intervention or active control, once.

Interventions are designed (assuming compliance) to be loosely matched on dose and required effort over a 14-day period. Each will incorporate information indicating minimum expected effort (e.g. how many practice activities to undertake each week).

Completion of information and activities will be recorded and presented to participants as a motivational element. In addition, participants will be able to record completion of relevant activities (e.g. physical exercise) undertaken outside the app.

There are no blended intervention elements (i.e., there is no clinician support during the interventions).

4.5.2 Active control

The active control condition consists of two weeks of once daily EMA measurement following the protocol described in **9.1**.

On Day 30, when the mini-trial concludes, all available interventions will be unlocked for both control and intervention participants.

4.5.3 Treatment follow-up

There is one follow-up measurement occasion, eight weeks after the post-intervention measurement occasion (see **7.4**). To balance intention-to-treat principles and minimising undue participant burden, only participants who completed the mid (pre-intervention) questionnaires will be invited to complete the follow-up questionnaires.

4.5.4 Accountability procedures:

The following accountability procedures will apply:

- Software version control ensuring only approved/tested version of each intervention is used in the trial.

4.6 Stopping rules (ICH GCP 6.4.6)

There are no discontinuation criteria for individual participants.

Consistent with the aim of the study to identify estimates of intervention effect as efficiently as possible, the overall trial will be stopped should any of the following pre-defined stopping rule(s) be satisfied.

Stopping rule
Interim analyses conclude that Optimisation Goals 1, 2 and 3 have been satisfied (see **4.3.2** and **8.2**)

Any mini-trial underway at the time of a trial-stopping rule being satisfied will complete as planned and the trial shall stop immediately after.

If stopping rules are not triggered, the trial will conclude once 12 mini-trials have been conducted.

4.7 Use of Case Report Forms (ICH GCP 6.4.9)

Case Report Forms will not be used in the Vibe Up study unless:

- Required for the assessment of a (suspected) adverse event by the Sponsor or their Independent Medical Expert (see **7.6.4**).
- Required as part of the examination of study data by the Data Safety Monitoring Board (see **13.2**).

Responsibility for generating Case Report Forms will be assigned in the **Delegations Log** (see **Appendix 15**.) A Case Report Form template is provided as **Appendix 32**.

Participant-level data generated by self-report assessments and study activities described in **7** will be captured and stored automatically using the schemas defined by the electronic database systems described in **13.4.** Manual assessment and/or data entry is not permitted.

Participant-level data created for the purposes of safety monitoring (see **7.6.4**) and participant safety responses (see **6.2**) will be stored in the registers defined for these purposes.

5 Selection and withdrawal of subjects

5.1 Subject inclusion criteria (ICH GCP 6.5.1)

To be eligible for inclusion, participants must satisfy *all* of the following criteria at screening:

- Adults aged 18 or older;
- Currently residing in Australia and planning to be resident throughout their mini-trial period;
- Currently enrolled at an Australian University (registered by TEQSA;)
- Advanced, fluent or native English speaker;
- Own an eligible personal smartphone (iPhone 6S/Android 5 or later) with active mobile number and internet access;
- Self-rated psychological distress on the Kessler Psychological Distress Scale, 10 item version[41] (K-10) scoring ≥ 20 (Clinical Research Unit for Anxiety & Depression scoring of "likely to have a mild (or more serious) mental disorder"[42]) at screening.

Eligibility screening questions are provided in **Appendix 8** (p66 onwards).

5.2 Subject exclusion criteria (ICH GCP 6.5.2)

Participants will be excluded at screening if they satisfy *any* of the following criteria:

- Self-rated suicidal ideation on the Suicide Ideation Attributes Scale[43] (SIDAS) ≥ 21 ("high ideation") at screening. Participants satisfying this criterion will trigger the Psychological Safety Response Procedure (see **Appendix 22**);
- Current self-reported active diagnosis of psychosis or bipolar disorder at screening;
- Already recruited to a previous mini-trial (having screened eligible and consented);
- Indicate limited availability to participate in a digital intervention during either the current or subsequent mini-trial periods (approximately 2 months);
- Indicate that any aspect of their personal circumstances would prevent them from taking part in a physical activity intervention, if allocated to receive this treatment.

5.3 Trial sites

The Vibe Up study is a single site decentralised randomised controlled trial based at Black Dog Institute.

It is a *single site* study because, although involving subinvestigators from multiple academic institutions, all study activities are coordinated through a trial operations team based at Black Dog Institute and there is no duplication of study roles.

It is *decentralised[44]* because electronic intervention delivery/assessment and support mechanisms mean that eligible participants can take part in the study regardless of where they are physically located within Australia.

5.4 Subject recruitment procedures

Trial recruitment will be based on online advertising via social media, supplemented with advertising via other avenues including university organisations, university societies, university staff contacts and releases to traditional media outlets. All advertisements will provide direct click-through to web-based study information and, for interested participants, self-directed screening/electronic consent.

Target sample sizes for recruitment are described in **8.1**.

Self-directed screening will be opened approximately 2 weeks prior to each block of mini-trials. Inclusion for a mini-trial will close at midnight three days before the mini-trial starts. For later mini-trials, a maximum n of 'screened eligible and consented' participants (n=maximum) may be set to enhance efficiency of the trial methodology. In this case, inclusion for a mini-trial will close at midnight three days before the mini-trial starts, or when the n=maximum is reached (whichever is sooner). If recruitment targets are not being reached, the following groups may be considered for inclusion:

- Invitation non-respondents, defined as those who did not respond to the download invitation and/or those who downloaded the app but did not complete the baseline questionnaire within a 72-hour eligibility period.

Forward referral may occur once per participant (i.e. each participant may be given up to two chances to take part in a mini-trial.) As a result, each participant may receive up to two invitations via email/SMS to join the study. Should a participant not respond to either of these they will be withdrawn from the study.

If we anticipate a significant time delay until the next mini-trial starts, online screening will be closed. When screening is closed, the link to the self-directed screening/electronic consent on the study information webpage will be replaced with an expression of interest form, where potential participants can enter their preferred name, email address, and current higher education institution. The study team will inform registered persons via email when study screening re-opens.

Participant flow after receiving study information is shown in **Figure 2** (p25). Upon completing the online eligibility screening (see **5.5**) and consent process (see **5.6**), eligible participants will receive an onscreen message and email confirming the start date of the mini-trial.

The online screening and consent platform will be hosted and managed by the Black Dog Institute. Black Dog Institute staff will collate the preferred name, mobile number, email address and sensor data collection consent status of 'screened eligible and consented' participants for each upcoming mini-trial into a spreadsheet that is identified by mini-trial number.

Black Dog Institute will send the password-protected spreadsheet for the upcoming mini-trial to Deakin University via UNSW OneDrive, by 12 noon two days prior to the mini-trial start date (i.e. 12 noon on Tuesday). Deakin University will be responsible for identifying any invitation non-respondent participants (per the definition above) from the previous mini-trial, and add them to the participant group for the upcoming mini-trial if required. Deakin University will then send an SMS to the mini-trial participants at 6pm on the day prior to the mini-trial start day, prompting participants that the mini-trial commences the next day.

A participant is considered 'enrolled' if they install the app and complete the baseline questionnaires.

The trial **Recruitment Strategy** is provided as **Appendix 18**.

5.5 Eligibility assessment method

Eligibility will be assessed via automated web-based self-screening completed at a potential participant's convenience. Screening will be based on the following self-report questionnaires (provided in **Appendix 8** at the page numbers shown below):

Id	Self-report questionnaire	Page
ELY	Basic eligibility questionnaire*	66
Consent will be taken at this point, assuming the participant satisfies basic eligibility (ELY).		
MED	Physical and mental health questionnaire*	71

KTEN	Kessler Psychological Distress Scale, 10-item version [41]	73
SIDAS	Extended Suicidal Ideation Attributes Scale [42]	75

* Study-specific screening tools

All ineligible participants will receive an automated thank you message and web links to psychological support information. Ineligible participants with elevated suicidal ideation will be managed according to the Psychological Safety Response Procedure (see **Appendix 22**).

5.6 Consent

Electronic consent will be incorporated into online participant information provision and screening. Consent will be taken after basic eligibility tests but *before* instrument/personal history exclusions (see **Table 5.5**).

An opt-in consent model will be used for the digital phenotyping passive sensing sub-study (see **9.2**) and the model consent from provided in **Appendix 7**.

Consent will *not* be repeated on app download, but app-specific permissions must be granted by participants to allow notifications and sensor-based data collection (if opted-in).

5.7 Subject withdrawal

5.7.1 Withdrawal criteria (ICH GCP 6.5.3)

Participants may be withdrawn from the study if:

- They experience a serious adverse event (see **7.6**) that:
  - is attributable to a study intervention or procedure; or
  - means they can no longer participate in the study; or
- They withdraw consent to take part (see below).

5.7.2 Withdrawal procedures

Participants can self-withdraw at any time by submitting to the study team the UNSW standard withdrawal form appended to the Participant Information Statement (see **Appendix 7).**

No mechanisms for automated withdrawal (e.g. via the study app) will be provided. To ensure that data belonging to a participant who withdraws can be uniquely identified, their mobile number will be solicited on the withdrawal of participation form.

Excluding self-withdrawals, the decision to withdraw a participant will be made by the Principal Investigator.

Handling of participant data after withdrawal will depend on when a participant decided to withdraw from the study. Participants will have their data removed from the study if they:

- Do not engage in the study after invitations to two mini-trials (see **5.4**); or
- Withdraw or are withdrawn prior to completion of the baseline assessment. Reflecting the intention-to-treat principles informing the planned analyses of the trial (see **8**), data from participants who withdraw after this point will not be discarded.

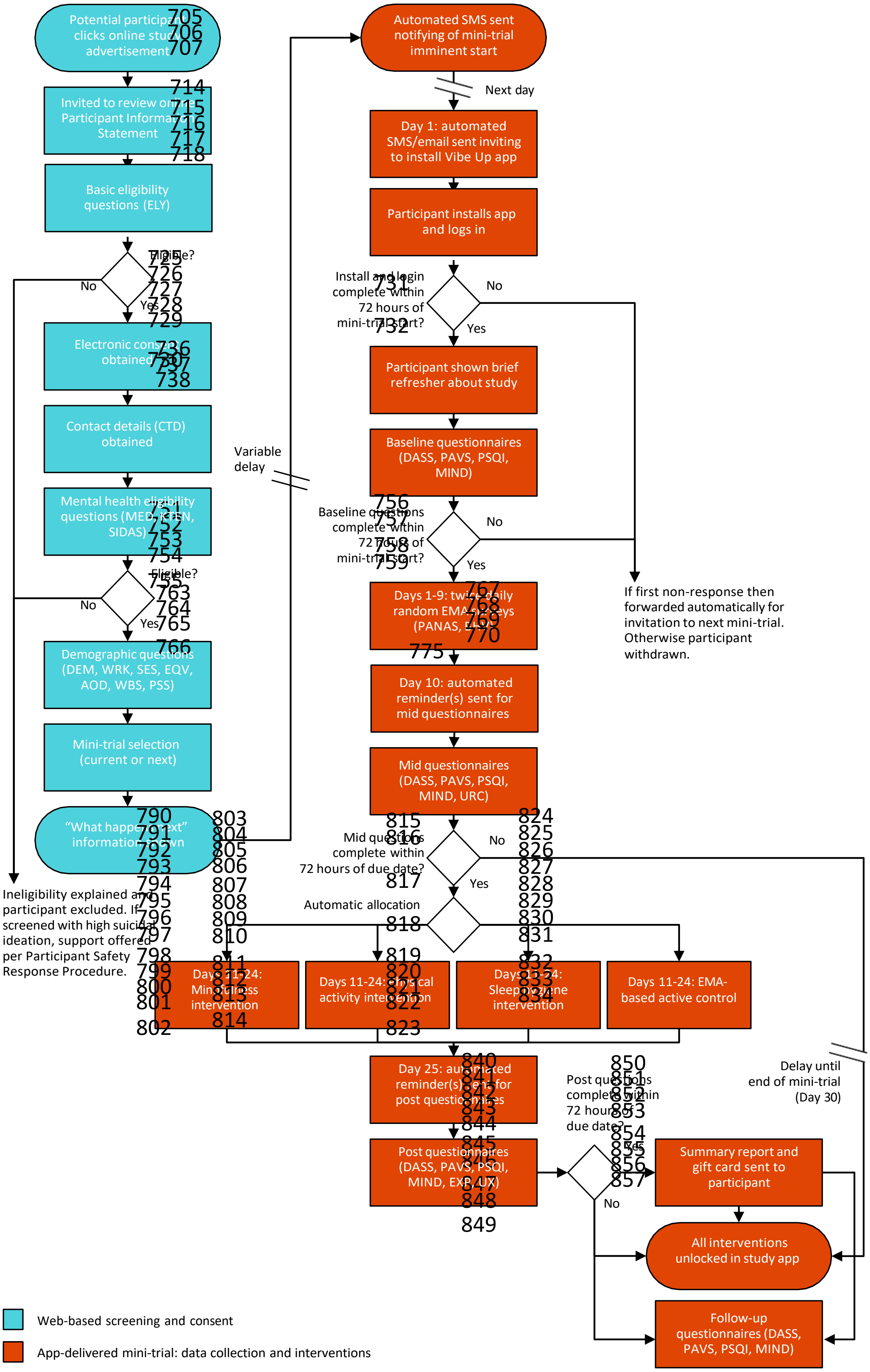

Figure 2 - Participant flow

For those who install the app and complete baseline, subsequent non-response (rather than active withdrawal) will *not* be treated as withdrawal but handled as missing data (see **8.6**).

Responsibility for confirming successful withdrawal of data will be assigned in the **Delegations Log** (see **Appendix 15**).

Non-identifiable data about withdrawals enabling aggregate reporting (e.g. counts by intervention assignment) will be retained in the **Withdrawals Log** which is part of the **Safety Monitoring Register** (see **Appendix 13**). In addition, any participant withdrawal linked to an adverse event will be recorded to enable reporting of safety-related statistics.

Participants who withdraw from the study will not be replaced and will not be followed up except per adverse event follow-up protocol, if applicable (see **7.6.6**).

6 Treatment of subjects

6.1 Treatments to be administered (ICH GCP 6.6.1)

See **4.5**.

6.2 Management of known and potential risks

The following controls are in place for the known and potential risks identified in **Table 2.3**:

Known risk	Control(s)
Moderate risk of transient anxiety symptoms or increased awareness of physical sensations (e.g. heart rate awareness, depersonalisation) during mindfulness meditation.	• Safety advice provided in 'Frequently asked questions' section for Vibe Up mindfulness intervention. Balancing risk of sensitising individuals who would not otherwise experience symptoms. The guidance will not be provided up-front (e.g. as a disclaimer.)
Moderate risk of bronchoconstriction in those with uncontrolled asthma/undiagnosed exercise-induced bronchoconstriction.	• Safety advice (e.g. inhaler use; discontinuation on respiratory symptoms) provided in pre-exercise briefing for 7-minute workout and in 'Frequently asked questions' section for Vibe Up physical activity intervention.<br>• Participant asked to self-determine appropriate level of exertion for all exercises.
Small risk of acute musculoskeletal injury or other acute exercise-related morbidity associated with any physical exercise.	• Safety advice (e.g. appropriate footwear and exercise setting) provided in pre-exercise briefing for 7-minute workout.<br>• Recovery advice provided in 'Frequently asked questions' section for Vibe Up physical activity intervention.<br>• Participant asked to self-determine appropriate level of exertion for all exercises.
Very small risk of acute cardiac event associated with vigorous physical exercise in deconditioned individuals.	• Exclusion of participants with known cardiac problems during screening (see **Appendix 8**, p66).<br>• Safety advice (e.g. immediate discontinuation on chest pain) provided in pre-exercise briefing for 7-minute workout.<br>• Participant asked to self-determine appropriate level of exertion for all exercises.

Known risk	Control(s)
**Potential risk**	**Control(s)**
Risk of inconvenience/contribution to psychological distress due to self-reflection as a result of assessment content or if an assigned Vibe Up intervention is ineffective in alleviating psychological distress symptoms.	• Additional support avenues (e.g., telephone support lines) displayed prominently in Vibe Up Study app, study website and print materials (listed as appendix to the **Psychological Safety Response Procedure**, see **Appendix 22.**)
Disclosure/identification of acute psychological risk to research team (including identification of suicidality during screening, see **5.2**)	• Management per defined **Psychological Safety Response Procedure**, see **Appendix 22.**

(Suspected) adverse events relating to known risks will be managed using the common process applicable to all adverse events (see **7.6.4**).

6.3 Medication(s)/treatment(s) permitted and not permitted before and/or during the trial (ICH GCP 6.6.2)

6.3.1 Before the trial

There are no restrictions on medication(s) or treatment(s) received by participants prior to the trial.

However, if at the time of screening medication or treatment is being received for bipolar disorder and/or psychosis *and the condition(s) is/are identified by the individual as currently active*, a potential participant will not be eligible to take part and will be excluded (see **5.2**).

6.3.2 During the trial

There are no restrictions on medication(s) or treatment(s) received by participants during the trial, whether:
- For physical or mental health related issues; and/or
- Received as part of routine/urgent healthcare or self-initiated.

Nevertheless, participants will be discouraged in trial materials from undertaking new psychological therapies during the four-week trial period.

Relevant exposures and explored as potential confounders via the Within-Study Exposures self-report questionnaire at post-intervention (see **Appendix 8,** page 103).

6.4 Procedures for monitoring subject compliance (ICH GCP 6.6.3)

Assessment of participant compliance during the trial and its subsequent analysis will be based on a combination of the following self-reported and automatically collected data:
- Self-reported compliance assessed using the following questionnaires provided in **Appendix 8:**

Id	Self-report questionnaire	Page
LOG	Daily log of engagement with intervention	104
UX	User experience (UX) questionnaire (questions UX4a and UX4b)	106

- App-generated data (see **7.5**).

Significant participant compliance issues will be identified and managed using the **Protocol Deviation Identification and Reporting Procedure** (see **Appendix 20**.)

6.5 Participant incentives

Participants who complete the post questionnaire will:

- be offered a $30 electronic gift token in recognition of their contribution to the study;
- receive a personalised summary report.

All interventions will become available to all participants via the app whichever is the sooner of:

- Full completion of the post-questionnaire; or
- Day 30.

Participants who complete the follow-up questionnaire will:

- be offered to go into a draw to win one of three $50 electronic gift token per mini-trial in recognition of their contribution to the study.

7 Assessment

7.1 Overview

Assessment of intervention effects will be based on data collected via validated self-report questionnaires (see **Table 7.3**) A combination of validated, adapted and bespoke self-report questionnaires, and automatically-collected app-generated data (see **7.5**) will be used to generate covariates and explanatory variables for primary and secondary data analyses.

7.2 Assessment of efficacy (ICH GCP 6.7)

The primary efficacy endpoint is the change in *total score* on the self-reported Depression, Anxiety and Stress Scale - 21 item version[38] (DASS-21) between the mid (pre-intervention) and post (post-intervention) timepoints (see **4.2**).

In addition, three brief self-report questionnaires will assess intervention-specific effects of, respectively, the Physical Activity, Sleep and Mindfulness interventions:

Id	Self-report questionnaire	Page
PAVS	Modified Physical Activity Vital Sign [45]	98
PSQI	Abridged Pittsburgh Sleep Quality Index [46]	98
MIND	Mindfulness single item questionnaire *	98

* Study-specific questionnaire

Methods and timing for assessing, recording and analysis of these parameters are described in **7.4** and illustrated in the participant flow shown in **Figure 2** (p25).

7.3 Self-report questionnaires

Validated standard instruments are shown in **bold**. All instruments are provided in **Appendix 8** (see page numbers referenced below.)

Id	Self-report questionnaire	Item count	Screening	Baseline	EMA	Mid	Intervention	Post	Follow-up	Page
ELY	Basic eligibility	11	●							66
RTP	Recruitment pathway	2	●							69
CTD	Contact details	4	●							70
MED	Physical and mental health	8	●							71
KTEN	**Kessler Psychological Distress Scale, 10-item version**	10	●							73
SIDAS	**Extended Suicidal Ideation Attributes Scale**	6	●							75
DEM	Demographic details	10	●							78
WRK	Study and employment	4	●							80
PCQ	**Productivity Costs Questionnaire**	13	●						●	81
MHS	**Use of Mental Health Care Services**	13	●						●	85
PUWS	**Prior use of wellbeing strategies**	9	●							88
EQV	**EQ-5D-5L**	6	●						●	89
ReQoL	**Recovering Quality of Life**	11	●						●	91
SES	**Subjective Socioeconomic Status Scale**	1	●							92
AOD	**Abridged NIDA-Modified ASSIST Drug Screening Tool**	4	●							93
WBS	**Short Warwick Edinburgh Mental Wellbeing Scale**	7	●							94
PSS	**Multidimensional Scale of Perceived Social Support**	12	●							95
DASS	**Depression, Anxiety and Stress Scale, 21-item version**	21		●		●		●	●	96
PAVS	**Modified Physical Activity Vital Sign**	2		●		●		●	●	98
PSQI	**Abridged Pittsburgh Sleep Quality Index**	1		●		●		●	●	98
MIND	Mindfulness single item questionnaire	1		●		●		●	●	98
PANAS	**Modified Positive and Negative Affect Schedule, Short Form**	13			●					99
BHV	Behavioural intentions	2			●					101
CEQ	**Abridged Credibility and Expectancy Questionnaire**	2				●				102
URC	**Abridged Revised University of Rhode Island Change Assessment Scale**	6				●				103
LOG	Daily log of engagement with intervention	1					●			104
EXP	Within-study exposures questionnaire	4						●		105
UX	UX questionnaire	9						●		106

7.4 Methods and timing for assessing, recording and analysis of study parameters

Screening questionnaires, which include for eligible participants a set of fixed demographic/trait penrsonality related measures, will be delivered via a web-based environment.

All subsequent questionnaires, including intervention pre-post and EMA measures, will be administered electronically by the Vibe Up app. There are three measurement occasions:

- Baseline (Day 1)
- Mid (Day 10)
- Post (Day 29)

The timing of each questionnaire is shown in **Table 7.3**. The expected question burden is shown below.

Timepoint	Expected number of questions		Remarks
	Min	Max	
Screening	2	131	Branched based on eligibility, active mental health diagnoses, K10, and SIDAS responses.

Timepoint	Expected number of questions		Remarks
	Min	Max	
Baseline	25	26	Branched based on response to PAVS question 1.
EMA	1	15	Branched based on responses to PANAS question 1 and BHV question 1.
Mid	31	32	Branched based on response to PAVS question 1.
Intervention	1	14	1 question per day, repeated for 14 days
Post	31	39	Branched based on allocation and response to PAVS/UX questions.
Follow-up	49	68	-

Participants will have 72 hours to complete each questionnaire battery. App-generated reminders will be sent to complete each questionnaire battery (with the exception of follow-up, when the initial prompt will be sent via SMS), followed by an SMS reminder at 36 hours and then an email reminder at 48 hours if no response. Although participants will be encouraged to activate app-generated reminders, they may opt-out of these and this is why SMS and email reminders are additionally required for non-respondents. Details of reminders are provided in **Appendix 9**.

All questionnaire data will be recorded automatically by the Vibe Up app.

Standard scoring procedures, where applicable, are specified for each instrument in **Appendix 8.**

Study parameters will be analysed:

- Automatically at the conclusion of each mini-trial according to the Multi-Arm Bandit algorithm procedures described in **4.3**;
- At the conclusion of the study according to the statistical analysis plans described in **8**.

7.5 App-generated data

The following instrumentation events will be collected automatically by the Vibe Up app with start (and, if applicable, end) timestamps to assess intervention compliance/dose and task engagement:

Data item
Initial user registration (with platform, OS, app version identifiers)
App activated/suspended
App navigation event (with details of destination location in app, e.g. module/page)
Questionnaire started/abandoned/completed (with questionnaire and timepoint identifiers)
Intervention module/FAQ started/abandoned/completed (with intervention and module identifiers)
Intervention interactivity events (e.g. video started/paused/completed; goal selected for the physical activity intervention)
EMA prompt generation and user responses*

* EMA questionnaire start/completion events are captured by questionnaire events.

7.6 Assessment of safety (ICH GCP 6.8)

7.6.1 Definitions

The Vibe Up trial adopts the definitions of key safety concepts specified by UNSW Clinical Trials Research Governance 'Safety Monitoring – Other Clinical Trials' guidelines[47]:

- An *adverse event* is any untoward occurrence (medical or other) in a clinical trial participant administered one or more of the trial interventions. It does not necessarily have a causal relationship with this treatment. Further:

- An adverse event is *serious* if it results in death, is life-threatening, requires hospitalisation or prolongation of existing hospitalisation, results in persistent or significant disability or incapacity.
- An adverse event is *expected* if it defined as a known or potential risk in **Table 2.3**. All other adverse events are *unexpected*.

- A *significant safety issue* is any issue that could adversely affect the safety of participants or materially impact on the continued ethical acceptability or conduct of the trial.
- An *urgent safety measure* is any action or procedure required to be taken to eliminate an immediate hazard to a participant's health or safety.

In addition the trial defines:

- A *protocol deviation* as an unplanned excursion from the protocol that is not implemented or intended as a systematic change[48].
    - A protocol deviation is *serious* if it (a) reduces the quality or completeness of the data, (b) makes the Consent Form inaccurate, or (c) impacts a subject's safety, rights, or welfare[48].

7.6.2 Safety parameters

Id	Self-report questionnaire	Signal	Page
DASS	Depression, Anxiety and Stress Scale, 21-item version	Increase (deterioration) in DASS-21 total score comparing pre- and post-intervention for each arm.	96
EXP	Within-study exposures questionnaire *	Participant-reported negative mental health event potentially attributable to intervention exposure reported at post-intervention.	105
UX	UX questionnaire *	Participant-reported experiences using the Vibe Up interventions (and app more generally) that may include safety-relevant information.	106

* Study-specific questionnaire

Additional parameters may be specified by the Data Safety Monitoring Board.

7.6.3 Methods and timing for assessing, recording and analysis of safety parameters

Methods and timing for collection of safety parameters will follow those already defined in **7.4** and **Table 7.3**.

Analysis and evaluation of safety parameters will be performed by the trial **Data Safety Monitoring Board** according to the Terms of Reference and Procedures defined in **Appendix 24.**

7.6.4 Procedures for eliciting, recording and assessing adverse events

Adverse event and/or protocol deviation reports will be elicited through:

- Participant self-reports provided via an email-based reporting mechanism provided in the Vibe Up app (or any other route); and/or
- Ad hoc reporting by any member of study staff at any time during the trial; and/or
- Proactive identification of protocol deviations under the **Protocol Deviation Identification and Reporting Procedure** (see **Appendix 20**); and/or
- Analysis of collected safety parameters by the Data Safety Monitoring Board.

All adverse events and protocol deviations will be recorded within 24 hours of their notification to trial staff in the **Safety Monitoring Register** (see **Appendix 13**).

For all events identified as serious and/or unexpected, the **Unexpected and Serious Adverse Event Notification Form** (see **Appendix 12**) must be completed within 72 hours and reviewed/approved by the Study's Qualified Medical Expert (named in **Table 1**) and the Principal Investigator.

For all other adverse events, the entry in the Safety Monitoring Register will be its report.

It is the responsibility of the Principal Investigator to consider whether an adverse event represents a significant safety issue. If a new significant safety issue is identified, the **Significant Safety Issue Notification Form** must be completed (see **Appendix 11**) and reviewed/approved by the Principal Investigator.

Any other remedial actions arising from an adverse event not meeting the threshold of significant safety issue should be recorded in the Unexpected and Serious Adverse Event Notification Form, if one exists, or the Safety Monitoring Register, otherwise.

Responsibility for event recording and the completion of notification forms will be assigned in the trial **Delegations Log** (see **Appendix 15**.)

All adverse events and protocol deviation reports, whether serious, unexpected or otherwise, and any accompanying Significant Safety Issue notification, must be reviewed within 72 hours by the Sponsor's Independent Medical Expert (named in **Table 1**). They will decide what action(s) are required, if any. These may include, but are not restricted to:

- A request to review the complete Case Report Form for the affected individual(s) (see **4.7**).
- A reclassification of the adverse event e.g. into a serious/unexpected event requiring additional actions (such as the completion of the Unexpected and Serious Adverse Event Notification Form.)
- The collection of additional information to better understand the impact/risks associated with the event.
- The identification of a new significant safety issue, urgent safety measure or recommendation relating to the suspension or termination of the trial which should be captured using the Significant Safety Issue Notification Form.

The Safety Monitoring Register will be periodically assessed by the Data Safety Monitoring Board according to its standard operating procedures (see **Appendix 24**).

7.6.5 Procedures for reporting adverse events

Per the UNSW Clinical Trials Research Governance 'Safety Monitoring – Other Clinical Trials' protocol:

- Single case reports of adverse events, whether serious or otherwise, will not be reported to the UNSW Sponsor's Delegate or the HREC but will be recorded in the **Safety Monitoring Register** (see **Appendix 13**), reviewed by the trial Data Safety Monitoring Board and reported to the UNSW Sponsor's Delegate annually.
- Significant safety issues will be reported immediately (within 7 days) of their occurrence to UNSW HREC.
- Urgent safety measures will be reported immediately (within 7 days) of their introduction to UNSW HREC.

In addition:

- Code breaks performed by the study team prior to the conclusion of primary data analysis will be recorded in the Safety Monitoring Register**.** Any decision to unblind all participants prior to the

conclusion of primary data analysis will be reported immediately (within 7 days) of its occurrence to the Sponsor.

It is the responsibility of the Principal Investigator (or their delegate) to ensure that events are reported as required.

7.6.6 Subject follow-up after adverse events

Follow up of subjects with *serious* adverse events will be decided by the Principal Investigator on a case-by-case basis, taking advice from UNSW HREC. *Non-serious* adverse events will not be followed up once recorded.

8 Statistics

8.1 Analysis of primary endpoint

For analysis/reporting, participants will be discretised into one of three clinically-relevant severity groups according to *baseline* normalised DASS-21 total score/DASS manual scoring categories:

- Normal or mild symptoms
- Moderate symptoms
- Severe or extremely severe symptoms

This approach will allow exploration of whether the most effective intervention(s) differ according to severity (consistent with **RQ4**.)

For each group, planned contrasts will compare intervention arms using the DASS-21 total score at pre-intervention (mid) and post intervention (post). After blindly rank-ordering all intervention arms including active control by their observed mean primary endpoint scores (given that lower is better, see **4.2**) such that $aa_0 < aa_1 < aa_2 < aa_3$, we will compare:

- The best performing intervention versus the other lower-performing interventions; aa_0 $vvvv$ aa_1, aa_0 $vvvv$ aa_2 and aa_0 $vvvv$ aa_3.
- The second-best performing intervention versus the other lower-performing interventions; aa_1 $vvvv$ aa_2 and aa_1 $vvvv$ aa_3.
- The third-best performing intervention versus the other lower-performing intervention; aa_2 $vvvv$ aa_3.

Contrasts are intended to be explicitly aligned with the cMAB optimisation goals (see **4.3.2**).

We will use a mixed-effects repeated measures (MMRM) model adjusted for multiple comparisons (see **8.4**). Pre-intervention (mid) scores will be incorporated as a predictor variable in all models. An unconstrained variance–covariance matrix will model within-individual dependencies. Transformation of scores, including categorisation, may be undertaken to satisfy distributional assumptions and accommodate outliers.

Analysis of the primary endpoint (see **4.2**) will be based on an intention-to-treat analysis strategy, analysing all participants starting the study regardless of the intervention received, under the assumption that missing data are Missing At Random (see **8.7**.)

Procedures for the analysis of all trial data are described in the **Data Verification and Analysis Procedure** (see **Appendix 21**).

8.2 Interim analyses

We will conduct interim analyses three times during the trial period, after mini-trials 4, 8 and 12 (evenly spaced). Interim analyses will be used to:

- Decide for each context-related clinical severity group defined in **8.1** , whether Optimisation Goal 1 (see **4.3.2**) has been satisfied and therefore whether the cMAB can be reconfigured for that group to focus on Optimisation Goal 2.
- Decide whether Optimization Goal 2 has been satisfied for all clinical severity groups, in which case the trial will seek to achieve Goal 3 (see **4.6**).

Interim analyses will follow the methods described in **8.1.**

8.3 Additional analyses

Mediation analyses will be explored using structural equation modelling.

We will use mixed effect logistic and Poisson regression models to assess if self-reported psychological distress and suicidality at the baseline are associated with the likelihood to respond to feelings measured using EMA (see **9.1**). We will assess the effect of time-varying responses to feelings, momentary affect, and changes in self-reported psychological distress and suicidality using mixed effects regression models. The relationship between momentary affect, psychological distress, exercise, and sleep quality at a given time interval will be assessed by mixed effect regression models. Descriptive analyses will be used to examine compliance and reactivity of the EMA.

Machine learning will be used to analyse digital phenotyping data (see **9.2**) to:

- explore whether any novel behavioural factors predict the study primary endpoint; and
- investigate within-individual behavioural signals that predict individual changes in self-reported distress or affect measured using EMA.

8.4 Significance level

The study pre-defined significance level for individual comparisons, alpha, is 0.05.

The risk of Type 1 error inflation associated with repeated planned comparisons that will be performed for the interim and trial analyses will be managed by applying a Benjamini-Hochberg adjustment[49] to each set of comparisons in order to control the False Discovery Rate for each clinical severity group at 5%.

8.5 Planned sample size

Up to 1200 participants across 12 mini-trials.

To allow for attrition between screening and mini-trial commencement, recruitment for each mini-trial will continue until 120 individuals have been screened eligible.

Assuming that up to a third do not respond to the subsequent invitation to install the study app and complete baseline questionnaires, this will yield at least 80 participants starting the mini-trial (i.e. 20 per arm, assuming the mini-trial 1 allocation ratio of 1:1:1:1)

Attrition after baseline is assumed at 20%, resulting in expected completion (i.e. having mid and post assessments) of 64 (i.e. n=16 per arm, assuming the initial allocation ratio of 1:1:1:1).

Assumptions will be updated based on observed attrition. Recruitment procedures may be adjusted to recruit more/fewer participants to ensure sufficient numbers of participants completing the mid and post assessments.

8.6 Selection of subjects for analysis

All randomized subjects who download the study app and complete the baseline questionnaire will be analysed.

8.7 Missing data

We intend to analyse data from all participants who start the study. To qualify as having started the study, participants must install the app and complete the baseline questionnaires within the 72-hour window. Participants who do not do this will be excluded from all study analyses (although may be given another chance to take part, see **5.3**).

To reduce bias in the assessment of a treatment's effectiveness, intention to treat based analysis of the data will be conducted. For this, all data from participants who had been recommended a treatment by the optimiser (following their completed onboarding DASS survey), who had also completed their mid-DASS survey but failed to complete the post-DASS survey, are included in the analysis.

To accommodate the results of participants with incomplete data, 'last observation carried forward' (LOCF) is avoided as this has been shown to produce potentially biased estimates. Instead, an observation-wise ANOVA-type model of group by time effects will be fitted (mixed-model repeated measures), allowing the variance of observations to vary between occasions of measurement (mid-DASS and post-DASS time points) and the residuals of individuals to correlate freely over occasions. This approach assumes that missing assessments are missing at random, an assumption that allows missingness to be dependent on observed information (intervention assignment and previous scores) but not on the unobserved values themselves.

As our analysis would be conducted separately for each cohort, the amount of data points will be divided and may lead to a small number of samples for a particular treatment within a cohort's analysis. To accommodate for this, an adjustment to the degrees of freedom of the mixed-model would be applied as per the Satterthwaite method.

To minimise missing data:

- Electronic reminders will be sent when data collections are due (see **7.4**).
- Incentives will be offered to participants (see **6.5**).
- An engagement strategy based on UX-led co-design of a reward/feedback mechanism is being developed.

There are no specific mechanisms for identifying, or responding to, excessive missingness. Statistics reporting proportions of missing data and study withdrawals will be published with the main trial report.

8.8 Confounding

Although RCTs are not in general susceptible to confounding (because randomisation means that confounders are expected to be equally distributed across arms) the Vibe Up study design *may* be susceptible to confounding relating to the combination of:

- *Temporal factors.* Should external contingencies affect the response of different mini-trial samples, For example, should early estimates be conditioned on students all of whom are undergoing examinations. Strategies to manage this risk include pooling participants from multiple institutions in each mini-trial

and the exclusion of participants indicating at screening significant expected disruption/inability to participate during the proposed mini-trial period.
- *UCB allocation.* The UCB allocation mechanism means that all participants with a given context (i.e. a given level of baseline distress severity) will be allocated to the *same single arm* for a given mini-trial.

The possibility of confounding will be assessed by looking for differences between mini-trials/arms across a range of measured characteristics/behaviour (e.g. engagement/compliance data, self-reported life events collected at post, and intervention compliance measured automatically by the study app.)

8.9 Spurious data

Potentially spurious and/or erroneous data (e.g. unusual response distributions) will be identified as part of planned data cleaning/verification steps described in the **Data Verification and Analysis Procedure** (see **Appendix 21**).

9 Sub-studies

9.1 EMA sub-study

9.1.1 Overview

EMA-based data collection will support exploratory analyses of the relationship between:
- Response styles to negative affect and psychological distress/baseline suicidality (see **RQ6-8**); and separately
- Whether passive sensing data can be correlated with within-individual changes in psychological distress (**RQ9**).

9.1.2 EMA data collection protocol

EMA will run from Days 1-3 to 9 (depending on when the participant installs the app and completes baseline) in the intervention arms and Days 1-3 to 28 in the active control arm.

EMA questionnaires will not be presented/prompted in any condition while the user has uncompleted questionnaires from the baseline/mid/post assessments. If applicable, however, EMA prompts will be activated as soon as these items are complete.

EMA run on a blended signal-contingent/event-based protocol consisting of:
- Signal-contingent: Two daily random prompts to the user generated by the study app at a random time within two windows: morning (08:00-10:00) and evening (19:00-21:00). Participants will have up to 60 minutes to respond to this prompt, with a reminder sent after 30 minutes to those not having responded to the initial prompt
- Event-contingent: Participants will be able to log EMA measurements at any time (e.g. in response to self-identified exposures to negative stressors, although participants will be allowed to log any feelings.)
    - If a participant initiates an event-contingent recording within the 08:00-10:00 or 19:00-21:00 windows, then no signal contingent prompt will be generated within that window, regardless of whether or not they complete their self-initiated EMA response.

Each EMA measurement will consist of:

- An initial filter question to elicit whether participants are experiencing any of the emotions drawn from an extended Short Form (12-item) version of the internationally reliable form of the Positive and Negative Affect Schedule[51] (I-PANAS), see **Appendix 8**, page 97.
- If any emotions are selected in the first question:
  - The I-PANAS items for the selected emotions (up to 12); and
  - Two follow-on questions exploring behavioural intentions as a result of measured feelings, (see **Appendix 8**, page 99):

    1. How likely are you to do something specifically as a result of how you are feeling right now? (4 item Likert: highly unlikely, unlikely, likely, highly likely).

    If the response to 1) is 'likely' or 'highly likely':

    2. What is it that are you likely to do?

- Recognising that participants may disclose risk information in their response, question 2 will include an annotation noting that "We don't actively monitor responses to this question, but help is always available if you need it." plus a link to support options.

9.2 Digital phenotyping sub-study

9.2.1 Overview

Digital phenotyping will be used to explore whether response to interventions and within individual changes in psychological distress are correlated with passively collected behavioural data (**RQ9**).

9.2.2 Digital-phenotyping data collection protocol

Digital phenotyping will run (for participants who do not opt-out at consent or disable the required permissions on their smartphone) continuously throughout the mini-trial period (from initial app installation until Day 30) and consist of:

- GPS location data, sampled on the basis of significant location change (as defined by the underlying operating system);
- Gravity-adjusted accelerometery (Android devices and iPhone 7 or later) and gyroscope (Android devices only) data, sampled with best-effort at 50Hz for 1 minute every 5 minutes.
- Operating system-generated activity label plus confidence (e.g. in vehicle, walking, stationary with low/medium/high confidence), step count and total distance travelled sampled with best-effort once every 5 minutes.
- Device power state and battery percentage, sampled with best effort once every 5 minutes.
- Device model, make, platform and app software versions, submitted with every sensor payload.

Recognising that, even after compression, passive sensing data payloads may be large, participants will be given mechanisms to control whether upload of passive sensing data (and other study data) occurs via a cellular connection or is restricted to Wi-Fi.

10 Direct access to source data and documents (ICH GCP 6.10)

The Principal Investigator, UNSW Sydney and Deakin University will permit, as required, the following oversight activities involving direct access to source data/documents:

- Trial-related monitoring;

- Sponsor or HREC initiated audits;
- HREC review; and
- Regulatory inspections.

11 Quality assurance and quality control (ICH GCP 6.11)

11.1 Quality assurance

Definition: *"Planned and systematic actions that are established to ensure that the trial is performed and the data are generated, documented (recorded), and reported in compliance with Good Clinical Practice (GCP) and the applicable regulatory requirement(s)"* ICH GCP 1.46

Quality assurance in this trial is supported and enabled by the following documentation (and the procedures they contain):

- The specification of the trial defined in this clinical trial protocol;
- The Standard Operating Procedures, including staff training procedures, based on this protocol that are specified in **Table 16.3**;
- The technology requirements, specifications and procedures specified in **Table 16.4**. As a principle, automated verification (e.g. data entry checks) will be applied wherever possible to assure data quality and completeness;
- The **Delegations Log** specified in **Appendix 15** and the formal allocation of significant study-related duties that this entails.

The Optimise technology platform used in this study has completed the UNSW Data Governance/Research Infrastructure System Classification assurance process (see **Appendix 26** and **Appendix 27)** and is classified as suitable for handling Sensitive Data (per the UNSW Data Standard definition.)

11.2 Quality control

Definition: *"Operational techniques and activities undertaken within the quality assurance system to verify that the requirements for quality of the trial-related activities have been fulfilled."* ICH GCP 1.47

The following quality control activities will be undertaken:

- Formal end-to-end testing, supervised by a professional Software Test Lead, of all software and digital self-report instruments used in the study and any updates subsequently applied. This process will verify/validate automated intervention delivery/fidelity and data collection.
- Formal documented version control/release mechanism for software products used in the study.
- Proactive monitoring, validation of completeness/correctness of data and evaluation of the performance of the technology and AI-Adaptive algorithm at key stages within each mini-trial, as defined in the **Mini-Trial Conduct and Monitoring Procedure** (see **Appendix 19**).
- Proactive identification of, and standard responses to, protocol deviations, as defined in the **Protocol Deviation Identification and Reporting Procedure** (see **Appendix 20**).
- Standardised data validation and cleaning procedures prior to any analysis, as defined in the **Data Verification and Analysis Procedure** (see **Appendix 21)**.

12 Ethics (ICH GCP 6.12)

Human Research Ethics approval will be sought from the following Human Research Ethics Committees (HRECs):

Lead HREC	
**HREC name**	**UNSW HREC A**
**HREC contact**	Leonne Thompson, Human Research Manager, UNSW Sydney
**Telephone**	+61 2 9385 6222
**Email**	humanethics@unsw.edu.au
**Address**	Research Ethics and Compliance Support Unit, Level 3 Rupert Myers Building South Wing, UNSW Sydney, Kensington NSW 2052
**HREC number**	HC200466
**Record of approval**	**Appendix 1**
Secondary HRECs	
**HREC name**	**Deakin University Human Research Ethics Committee** (DUHREC)
**HREC contact**	The Manager, Research Integrity, Deakin University
**Telephone**	+61 3 9251 7123
**Email**	research-ethics@deakin.edu.au
**Address**	Deakin Research Integrity, Deakin University, 221 Burwood Highway, Burwood VIC 3125
**HREC number**	2021-098
**Record of approval**	**Appendix 2**

Before commencing recruitment and/or data collection, the Principal Investigator (or their delegates) will arrange, and ensure completion of, a process of study start up training for all personnel involved in the trial, using the most recent approved versions of the clinical trial protocol, standard operating procedures and/or relevant essential documents, following the procedures specified in the **Training Policy** (see **Appendix 16**.)

Delivery and completion of training will be recorded in the Training Log (see Appendix 1 of the Training Policy.)

13 Data handling and record keeping (ICH GCP 6.13)

13.1 Applicable policies and procedures

The UNSW Research Data Governance & Materials Handling Policy provides the overarching policy framework for data handling and record keeping in the trial. The following trial-specific standard operating procedures will be used to manage data on a day-to-day basis.

Trial-related procedure	Document	Appendix
Data handling	Research Data Management Plan	Appendix 23
Data verification*	*Before the trial*<br>Screening and Self-Report Questionnaire Testing Plan	Appendix 30
	During the trial<br>Data Verification and Analysis Procedure	Appendix 21
Statistical analysis	Data Verification and Analysis Procedure	Appendix 21
Preparation of trial reports	Publications Policy	Appendix 17

* Data verification is the confirmation, through provision of objective evidence, that specified data collection requirements have been fulfilled (e.g. that electronic versions of self-report questionnaires meet the specifications defined in Appendix 8

and that electronic data is captured and persisted with fidelity.) In the Vibe Up Study, this will be done via through a combination of software testing (prior the study) and review (during the study).

13.2 Data Roles

Role	Named holder
Data Custodian<br>*Ultimate owner and retainer of data generated by the study.*	UNSW
Research Data Owner<br>*Accountable for ensuring effective local protocols are in place to guide the appropriate use of their data and materials.*	Professor Helen Christensen, Principal Investigator
Research Data Steward<br>*Responsible for the quality, integrity, implementation and enforcement of data management within their research project. Research Data Stewards will classify and approve user access to the data and materials, under delegation from a Research Data Owner, based upon the appropriateness of the User's role and the intended use.*	Dr Artur Shvetcov, Postdoctoral Research Fellow, Black Dog Institute

13.3 Data Safety Monitoring Board

The Vibe Up Data Safety Monitoring Board (DSMB) will be responsible for the following trial procedures:

- Assessment of all clinical trial safety data and critical efficacy endpoints at regular intervals;
- Generating recommendations to the Principal Investigator/Sponsor on whether to continue, modify, or stop a trial; and
- Review of the final study report at the conclusion of the study.

Terms of reference and procedures for the DSMB are defined in **Appendix 24**. These specify the frequency for which the above activities will occur and detail how written records of meetings will be retained and maintained throughout the life cycle of the research.

13.4 Data Collection and Storage Platforms

All trial data will be stored electronically.

The following electronic platforms will be used to collect and store trial data:

**Data collection**	
**Platform name**	Qualtrics
**Type**	Cloud-based survey platform
**Purpose**	Participant self-screening and electronic consent collection.
**Conforms to UNSW data classification standard?**	Yes, for *sensitive* data. UNSW-endorsed service[52].
**Platform name**	Optimise
**Type**	Cloud-based trial orchestration platform
**Purpose**	Self-report questionnaire, app usage data and sensor data collection and mini-trial orchestration
**Conforms to UNSW data classification standard?**	Yes, for *sensitive* data. System classification completed, see **Appendix 26**, and approved, see **Appendix 27**
**Data storage**	
**Platform name**	UNSW OneDrive

**Type**	Cloud-based file hosting service
**Purpose**	Secure storage and backup of all trial-related data and analytical work products (e.g. data analysis files.)
**Conforms to UNSW data classification standard?**	Yes, for *highly sensitive* data. UNSW supported platform[52].

13.5 Data Retention and Disposal

Trial data will be retained for 15 years after the conclusion of the study.

Provisions for trial data retention and disposal are documented in the Research Data Management Plan (see **Appendix 23**).

13.6 Data Sharing

Data from this study will be stored in a databank for future use. Research proposals from applicants will be reviewed and approved by the Vibe Up Study Publications Committee before access to study data can be granted.

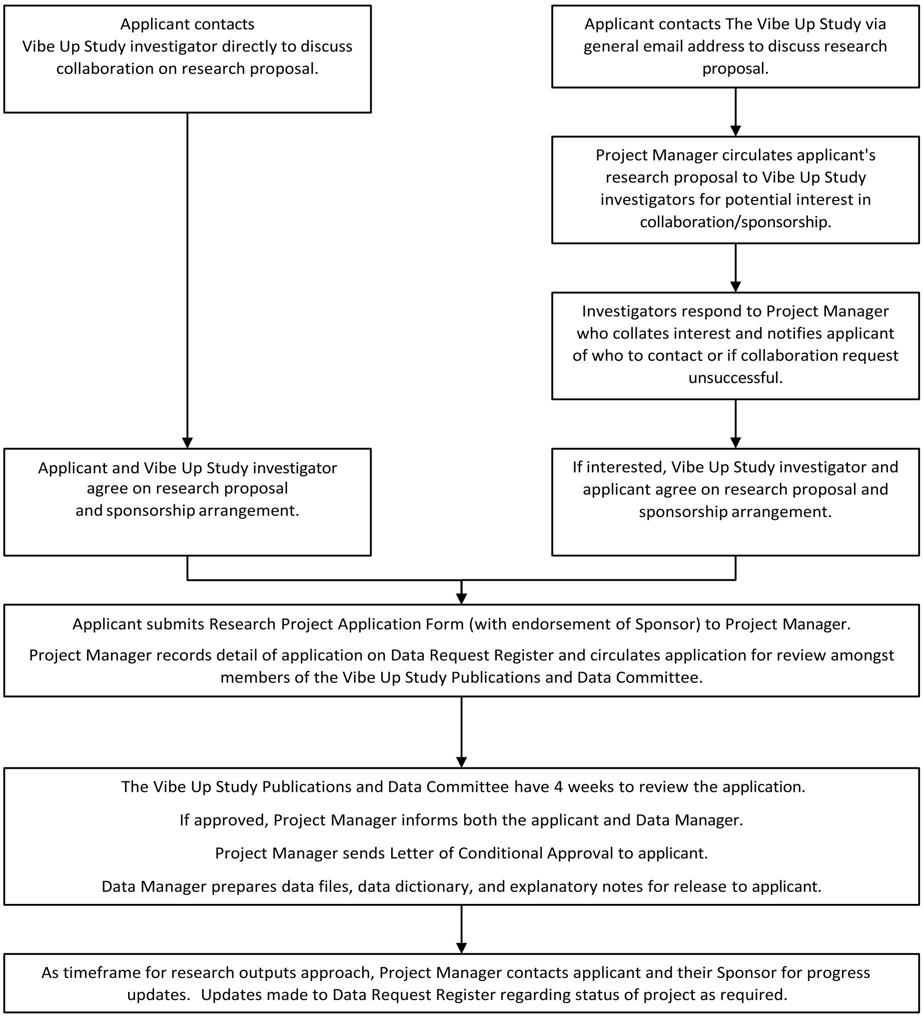

Figure 3 - Data request application and review process

The processes required for gaining access to the Vibe Up Study data are as follows (see **Figure 3**):

- Research proposals from applicants are circulated to study investigators to identify a sponsor for the application. Only applications with a sponsor will proceed to the next stage.
- The applicant submits a Data Request Application form (see **Appendix 17**1Appendix 17), with evidence of sponsorship, to the Project Manager who records application details in the Data Request Register and circulates the application to members of the Vibe Up Study Publications Committee for review.
- The Committee will either approve or reject the application within 4 weeks. Successful applicants will receive a letter of conditional approval from the Project Manager.
- Data Manager will prepare data files, data dictionary, and explanatory notes for release to successful applicants upon receiving applicable ethical approvals.

14 Financing and insurance (ICH GCP 6.14)

14.1 Funding details

**Grant 1 – Optimising treatments in mental health using AI**	
Grant details	
**Reference number**	MRFAI000028
**Activity start date**	30 June 2020
**Activity end date**	29 June 2023
Funder	
**Funder name**	Commonwealth of Australia, Department of Health
**Funder contact**	Masha Somi, Chief Executive Officer, Health and Medical Research Office, Department of Health
**Telephone**	+61 2 6289 7705
**Email**	masha.somi@health.gov.au
**Address**	Sirius Building, Furzer Street, Woden Town Centre ACT 2606
Contracts	
Funding agreement	Appendix 34

The UNSW finance project code for the Optimise project is **RG200942+RE939+BDI**. The Black Dog Institute code is **UDOP**.

14.2 Insurance

UNSW insurance cover for this trial is provided by Newline Australia Insurance Pty Ltd. Original confirmation of cover is provided as **Appendix 3**.

Insurance cover will be reviewed annually and renewed as required. Routine renewal of insurance cover will not be treated as a notifiable protocol change.

14.3 Clinical Trial Research Agreements

This decentralised trial operates at a single site hosted by the Black Dog Institute. As a result, there are no Clinical Trial Research Agreements for this study and no site-specific assessments (SSAs) will be performed.

The Collaborative Research Agreement that governs research partners involved in this study is provided as **Appendix 32**.

15 Publications policy (ICH GCP 6.15)

The Vibe Up Study is subject to the publications policy of the MRFAI Optimise Project within which it sits.

This policy is provided as **Appendix 17**. As long as this policy remains in place and is agreed by all project partners, revisions will not be treated as a notifiable protocol change.

16 Essential documents

16.1 Trial documentation

Document	Appendix	Date version
UNSW Human Research Ethics Application Form	(External document)	20210114
UNSW Human Research Ethics Project Description	(External document)	20201201
UNSW Human Research Ethics Committee Approval	Appendix 1	20210202
Deakin University Human Research Ethics Committee Approval	Appendix 2	20210315
UNSW Medicine Letter of Support	Appendix 4	20210113
Deakin University A2I2 Letter of Support	Appendix 5	20201215
Recruitment Material	Appendix 6	20210406
Participant Information Statement and Consent Form	Appendix 7	20210406
Self-Report Questionnaires	Appendix 8	20210406
Study Reminders	Appendix 9	20210121
Vibe Up Intervention Specifications	Appendix 10	20210115
Significant Safety Issue Notification Form	Appendix 11	20201201
Unexpected and Serious Adverse Event Notification Form	Appendix 12	20201201

16.2 Legal agreements and regulatory information

Document	Appendix	Date version
Funding Agreement	Appendix 34	20200619
Research Partner Collaborative Research Agreement	Appendix 32	20210107
UNSW Confirmation of Insurance Cover	Appendix 3	20201217

16.3 Standard Operating Procedures

Procedure	Appendix	Date version
Safety Monitoring Register	Appendix 13	20201201
Pre-Trial and Trial Initiation Monitoring Procedure	Appendix 14	20201201
Delegations Log	Appendix 15	20210115
Training Policy	Appendix 16	20210315
Publications Policy	Appendix 17	20210406
Recruitment Strategy	Appendix 18	20210310
Mini-Trial Conduct and Monitoring Procedure	Appendix 19	20201201
Protocol Deviation Identification and Reporting Procedure	Appendix 20	20201201
Data Verification and Analysis Procedure	Appendix 21	20201201
Psychological Safety Response Procedure	Appendix 22	20210119
Research Data Management Plan	Appendix 23	20210315
Data Safety Monitoring Board Terms of Reference and Procedures	Appendix 24	20210309
Case Report Form	Appendix 32	20210406

16.4 Technology Requirements, Specifications and Procedures

Document	Appendix	Date version
Vibe Up Algorithm Specification	Appendix 25	20210406
Optimise UNSW System Classification	Appendix 26	20201214
Optimise UNSW System Classification Approval	Appendix 27	20201214
Vibe Up App Software Requirements	Appendix 28	20210301
Vibe Up App Testing Plan	Appendix 29	20210310
Screening and Self-Report Questionnaire Testing Plan	Appendix 30	20210312
Systems Incident Identification and Response Protocol	Appendix 31	20210208

17 Signatures

Signature of person authorised to sign the protocol by the Sponsor.

Name (please print)	Helen Christensen
Signature	
Date	Date

Appendices

Appendix 1 UNSW Human Research Ethics Committee Approval

01-Feb-2021

Dear Scientia Professor Helen Christensen,

**Project Title**	Optimise study: Using Artificial Intelligence to identify the optimal self-care strategies for psychological distress
**HC No**	HC200466
**Re**	HC200466 Notification of Ethics Approval
**Approval Period**	29-Jan-2021 - 28-Jan-2026

Thank you for submitting the above research project to the **HREC Executive** for ethical review. This project was considered by the **HREC Executive** at its meeting on **28-Jan-2021**.

I am pleased to advise you that the **HREC Executive** has granted ethical approval of this research project. The following condition(s) must be met before data collection commences:

Conditions of Approval:
Conditions of Approval
1. Registration of the clinical trial on the Australian New Zealand Clinical Trials Registry must be completed before recruitment, and data collection commences. The registration number must be provided to the human ethics team for record-keeping purposes.
2. Confirmation of sponsor related responsibilities is to be obtained before recruitment and data collection commences.

Conditions of Approval - All Projects:

- The Chief Investigator will immediately report anything that might warrant review of ethical approval of the project.
- The Chief Investigator will seek approval from the **HREC Executive** for any modifications to the protocol or other project documents.
- The Chief Investigator will notify the **HREC Executive** immediately of any protocol deviation or adverse events or safety events related to the project.
- The Chief Investigator will report to the **HREC Executive** annually in the specified format and notify the **HREC Executive** when the project is completed at all sites.

- The Chief Investigator will notify the **HREC Executive** if the project is discontinued before the expected completion date, with reasons provided.
- The Chief Investigator will notify the **HREC Executive** of his or her inability to continue as Coordinating Chief Investigator including the name of and contact information for a replacement.

The **HREC Executive** Terms of Reference, Standard Operating Procedures, membership and standard forms are available from https://research.unsw.edu.au/research-ethics-and-compliance-support-recs.

If you would like any assistance, or further information, please contact the ethics office on:
P: +61 2 9385 6222, + 61 2 9385 7257 or + 61 2 9385 7007
E: humanethics@unsw.edu.au

Kind Regards,

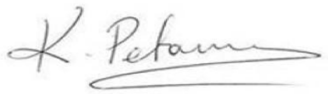

Associate Professor Kathy Petoumenos
Human Research Ethics Presiding Member

This HREC is constituted and operates in accordance with the National Health and Medical Research Council's (NHMRC) *National Statement on Ethical Conduct in Human Research (2007)*. The processes used by this HREC to review multi-centre research proposals have been certified by the National Health and Medical Research Council.

Appendix 2 Deakin University Human Research Ethics Committee Approval

DEAKIN UNIVERSITY

Memorandum

**To:**	Prof Rajesh Vasa<br>A2I2D<br><br>B	**cc:**
**From:**	Deakin University Human Research Ethics Committee (DUHREC)	
**Date:**	15 March, 2021	
**Subject:**	2021-098<br>Optimise study: Using Artificial Intelligence to identify the optimal self-care strategies for psychological distress<br>Please quote this project number in all future communications	

Approval granted by University of NSW HREC for this project will be noted at the DUHREC meeting to be held on 19/04/2021.

It will be noted that approval has been granted for Prof Rajesh Vasa, A2I2D, to undertake this project as stipulated in the University of NSW HREC approval documentation.

The approval noted by the Deakin University Human Research Ethics Committee is given only for the project and for the period as stated in the memo. It is your responsibility to contact the HREC should the project be discontinued before the expected date of completion. You are reminded that:

- The Deakin logo should be on any participant documents, including the Plain Language Statement, or where that is not possible, ensure Deakin University's involvement in the project is clearly written in the documentation
- The Deakin Human Research Ethics Office needs to be notified immediately if any complaints are received
- An annual/progress report must be submitted to the approving HREC and at the conclusion of the project, a final report must be submitted to the Deakin HREC.

DUHREC may need to audit this project as part of the requirements for monitoring set out in the National Statement on Ethical Conduct in Human Research 2007 (Updated 2018).

Human Research Ethics Unit
research-ethics@deakin.edu.au
Telephone: 03 9251 7123

Human Ethics - Deakin Research Integrity
Tel 03 9251 7123
research-ethics@deakin.edu.au

Melbourne Burwood Campus,
221 Burwood Highway, Burwood, VIC 3125
deakin.edu.au

1

Deakin University CRICOS Provider Code: 00113B

Appendix 3 UNSW Confirmation of Insurance Cover

Routine renewal of insurance cover will not be treated as a notifiable protocol change.

AON
Empower Results®

Certificate of Currency

Date of Issue: 29 October 2021

To whom it may concern,

Contact: Samir Sofat
t: 02 9253 7233
e: samir.sofat@aon.com

We hereby certify that the under mentioned insurance policy is current as at the date of this certificate, please refer to the important notices below.

**Policy Type**	Clinical Trials Insurance
**Insured**	University of New South Wales<br><br>Additional Insureds:<br>Kirby Institute;<br>UNSW Counselling Service and Compass Programs (UNSW Psychology Clinic);<br>UNSW Health Service (University of New South Wales Medical Centre); UNSW (Medicine) Lifestyle Clinic;<br>University of New South Wales Optometry Clinic<br>UNSW Sport and Recreation (UNSW Lifestyle Centre);<br>UNSW Psychology Clinic;<br>UNSW Global Pty Ltd;<br>Scientia Clinical Research Limited;<br>UNSW School of Psychology
**Insurer**	Newline Australia Insurance Pty Ltd
**Policy Number(s)**	AUS21888283A
**Period of Insurance**	From: 4.00 pm 01st November 2021 Local Standard Time<br>To: 4.00 pm 01st November 2022 Local Standard Time
**Limits of Liability**	**Coverage Section 3A: No Fault Compensation for Clinical Trials**<br>AUD 20,000,000 any **One Claim** and in the aggregate during the **Period of Insurance**<br>**Coverage Section 3B: Clinical Trials: Legal Liability**<br>AUD 20,000,000 any **One Claim** and in the aggregate during the **Period of Insurance**<br>**Coverage Section 4A: Medical Malpractice**<br>AUD 20,000,000 any **One Claim** and in the aggregate during the **Period of Insurance**
**Jurisdictional Limit**	Commonwealth of Australia
**Retroactive Date**	28 November 2008 in respect of a $5,000,000 Limit of Liability<br>20 December 2016 in respect of a $10,000,000 Limit of Liability

Further Information
Should you have any queries, please contact us on the details set out at the top of the page.

Important notes
- Aon does not guarantee that the insurance outlined in this Certificate will continue to remain in force for the period referred to as the Policy may be cancelled or altered by either party to the contract, at any time, in accordance with the terms of the Policy and the Insurance Contracts Act 1984 (Cth).
- Aon accepts no responsibility or liability to advise any party who may be relying on this Certificate of such alteration to or cancellation of the Policy.
- Subject to full payment of premium
- This certificate does not:
  - represent an insurance contract or confer rights to the recipient; or
  - amend, extend or alter the Policy
  - contain the full policy terms and conditions

Appendix 4 UNSW Medicine Letter of Support

13 January 2021

Professor Sean Emery
Acting Dean of Medicine
UNSW Medicine and Health

Professor Helen Christensen
Black Dog Institute
Hospital Road
Randwick NSW 2031

Re: The Vibe Up Study - MRFAI000028 (Funding reference); RG200942 (UNSW RGO); HC200466 (UNSW HREC)

The above-named research project is being submitted for consideration by Professor Helen Christensen, Chief Investigator.

I have seen the application and I agree that the required academic expertise and resources are available to complete this proposed research.

Should you have any queries please do not hesitate to contact me.

Thank you for your assistance.

Yours sincerely,

Professor Sean Emery
Acting Dean
UNSW Medicine and Health

LEVEL 4 | LOWY BUILDING | GATE 9 HIGH STREET |
UNSW AUSTRALIA | UNSW SYDNEY NSW 2052 AUSTRALIA
T +61(2) 9385 1007 | F +61 (2) 9385 1289 | ABN 57 195 873 179 | CRICOS Provider Code 00098G
SYDNEY | CANBERRA | AUSTRALIA

Appendix 5 Deakin University Applied Artificial Intelligence Institute Letter of Support

A2I2 APPLIED ARTIFICIAL INTELLIGENCE INSTITUTE

DEAKIN UNIVERSITY

15/12/2020

Prof Helen Christensen
Black Dog Institute
Hospital Road
Randwick 2031

Dear Helen,

Re: **The Vibe Up Study: a decentralised AI-response-adaptive randomised controlled trial of smartphone app-delivered self-care strategies for psychological distress in adult university students**
Letter of Support

This letter is to confirm that the Applied Artificial Intelligence Institute (A2I2) at Deakin University is aware of this proposed research study.

As a principal partner to the MRFAI "Optimising treatments in mental health using artificial intelligence" grant, we acknowledge and agree the involvement of A2I2 in the completion of this trial.

Our involvement will include:

- Devising, implementing and supporting the multi-arm bandit optimisation process throughout the trial.
- Developing and supporting the trial app and back-end technology infrastructure throughout the trial and ensuring its compliance with UNSW (and, if applicable, Deakin University) Data Governance principles.
- Supporting the recruitment process by promoting the study to Deakin university students.
- Supporting principal and exploratory machine learning-based data analyses.
- Once approved by UNSW, arranging reciprocal ethics approval via the Deakin University Human Research Ethics Committee, so that the above activities can be carried out.
- Ensuring that all Deakin trial staff receive training on the study protocol and that appropriate insurance is in place.

A2I2 also undertakes to allow and facilitate, if required, the following oversight activities involving direct access to source data/documents:

- Trial-related monitoring;
- Sponsor or HREC initiated audits;
- HREC review; and
- Regulatory inspections.

Applied Artificial Intelligence Institute

Geelong Waurn Ponds Campus,
75 Pigdons Road, Waurn Ponds, VIC 3216
http://a2i2.deakin.edu.au

Deakin University CRICOS Provider Code: 00113B

APPLIED ARTIFICIAL INTELLIGENCE INSTITUTE

DEAKIN UNIVERSITY

As the Co-Director of A2I2, I have read through your research proposal and support our involvement in this study, subject to receiving appropriate ethical approval and participant consent.

Yours sincerely, Yours sincerely,

Prof Svetha Venkatesh
Co-Director, Alfred Deakin Professor, ARC Australian Laureate Fellow
Applied Artificial Intelligence Institute, Deakin University

Appendix 6 Recruitment Material

Vibe Up Study Recruitment Material

Version dated: 26 Apr 2021

1. Purpose

The purpose of this document is to provide the material that shall be used for creating advertisements and sharing about the study to promote the Vibe Up Study to potential participants.

2. Recruitment advertisement material

The below copy and visual assets will be flexibly combined to create study advertisements. This flexibility will allow for the best performing combinations to be continued, to optimise recruitment.

2.1 Advertisement copy

Headline variants:

Participate in mental health research	Higher education students | Participate in mental health research
Test a new wellbeing app & be reimbursed for your time	Seeking higher education students who want to improve their wellbeing
Take part in a world-first wellbeing study for uni students	Higher education students | Test a new wellbeing app
Improve your wellbeing and be reimbursed for your time	Build better mental health in 30 days by taking part in Vibe Up
Build resilience to stress by testing a new app	Uni getting you down? Get your vibe up.

Copy variants:

The Black Dog Institute is seeking higher education students to test a new wellbeing app over 30 days. You'll learn strategies taught by leading doctors and psychologists, and be reimbursed for your time.	We're excited to be launching a world-first study looking at how AI can be used to deliver mental health interventions to higher education students at scale.<br><br>The app, Vibe Up, teaches strategies in sleep hygiene, mindfulness and physical activity to boost mood, and reduce stress through completing engaging, daily tasks over 30 days. Get your students on board today.

More than 1 in 4 university students experience high-stress levels that negatively impact their studies, relationships, and daily lives. Vibe up is a world-first study aiming to change this at scale.<br><br>If you decide to take part, you'll be invited to test a new wellbeing app, developed by doctors and psychologists, that will teach you strategies to combat stress and boost mood - and be reimbursed for your time.	*Calling all higher education students* Join a world-first study using AI to explore different methods of mental health training, that can boost your wellbeing reduce uni-related stress - all in the palm of your hand.<br><br>At the end of the 30 days, you'll also receive a personalised report and tailored suggestions for staying well.
Take steps to improve your wellbeing and increase your resilience to university-related stress. Participate in world-first research, using AI to explore different methods of mental health training in the palm of your hand.	We're seeking higher education organisations to help us spread the word about Vibe Up - a new study aiming to reduce stress and improve wellbeing in your students. Everything you need to share the news can be found here.
Leading mental health organisation, Black Dog Institute, is currently seeking higher education students to take part in a world-first study looking at how AI can be used to build better mental health at scale.	We're looking for Australian uni, TAFE and college students who want to reduce stress and improve wellbeing, to test a new app. You'll learn strategies taught by leading doctors and psychologists, in the palm of your hand, and be reimbursed for your time.
Improve your wellbeing and increase your resilience to university-related stress by taking part in a world-first study. You'll be guided through engaging daily tasks that teach you strategies (taught by doctors & psychologists) to improve your mental health.	More than 1 in 4 university students experience high-stress levels that negatively impact their studies, relationships, and daily lives. Get involved in the vibe up study and learn strategies to combat this - all in the palm of your hand.

2.2 Advertisement visual assets

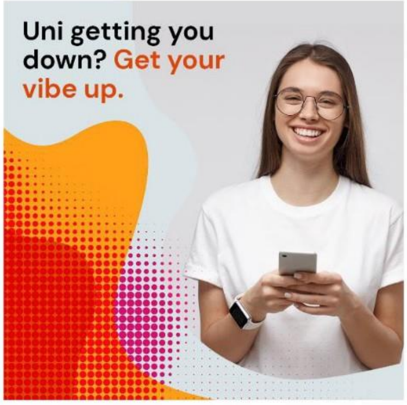

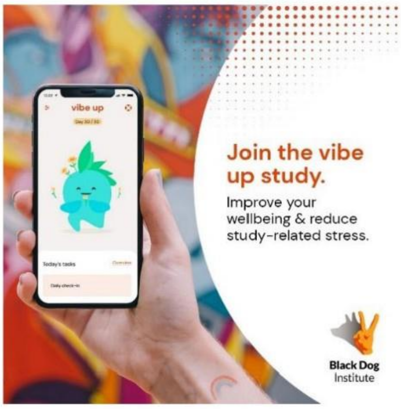

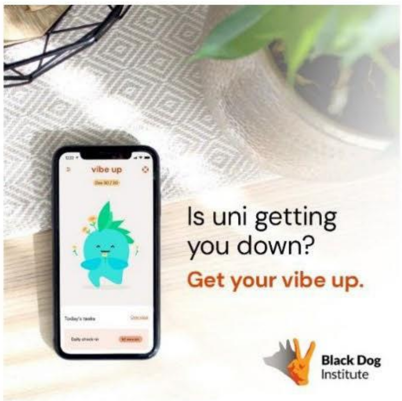

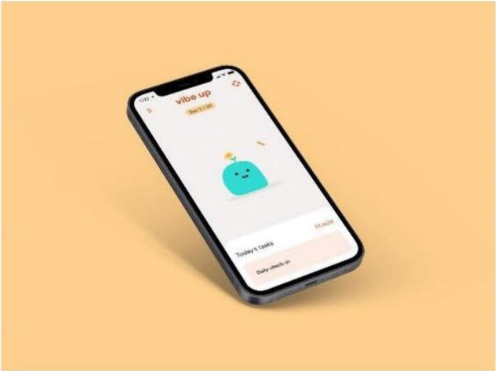

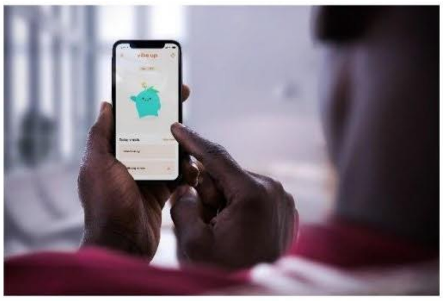

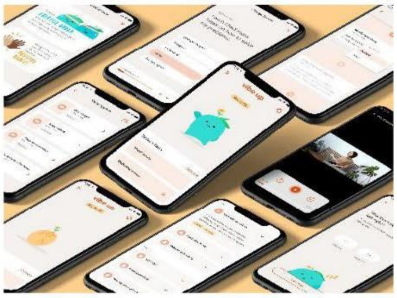

The below image is a screenshot of a visual asset that contains stable text, with a repeating animation of the character bouncing up and down:

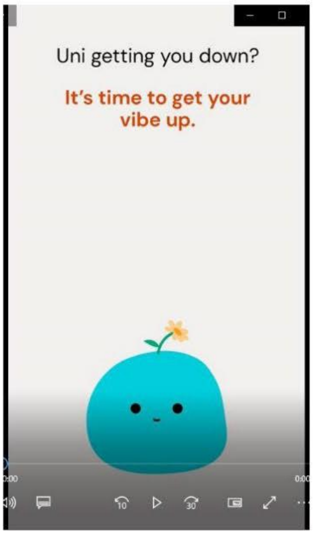

Page 3 of 11
HC Number: HC200466
Version dated: 26 Apr 2021

The below images will be purchased for use without watermark if ethics approval is received:

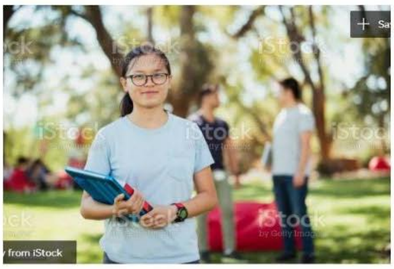

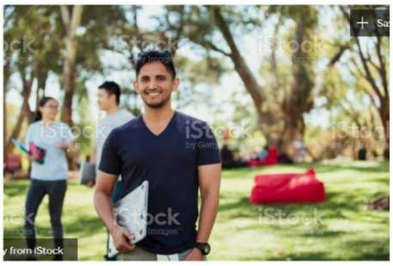

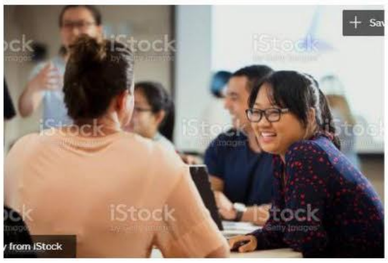

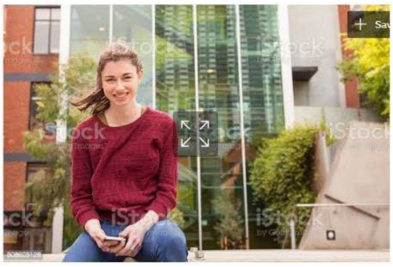

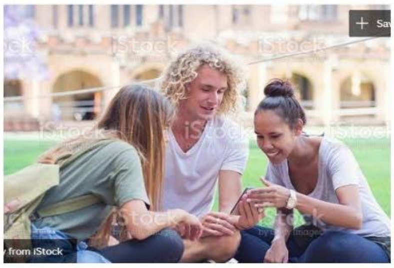

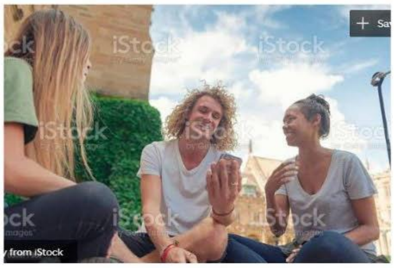

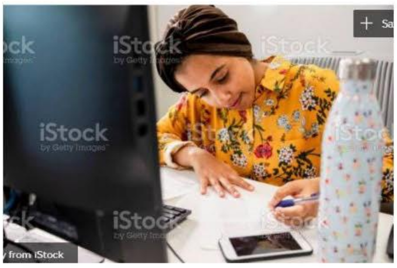

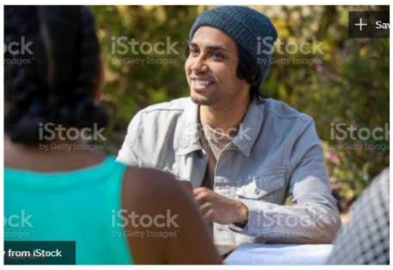

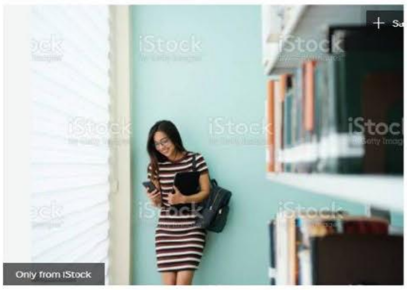

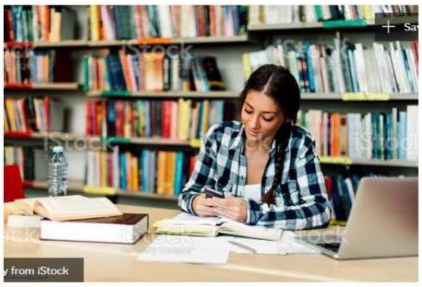

2.3 Advertisement posters

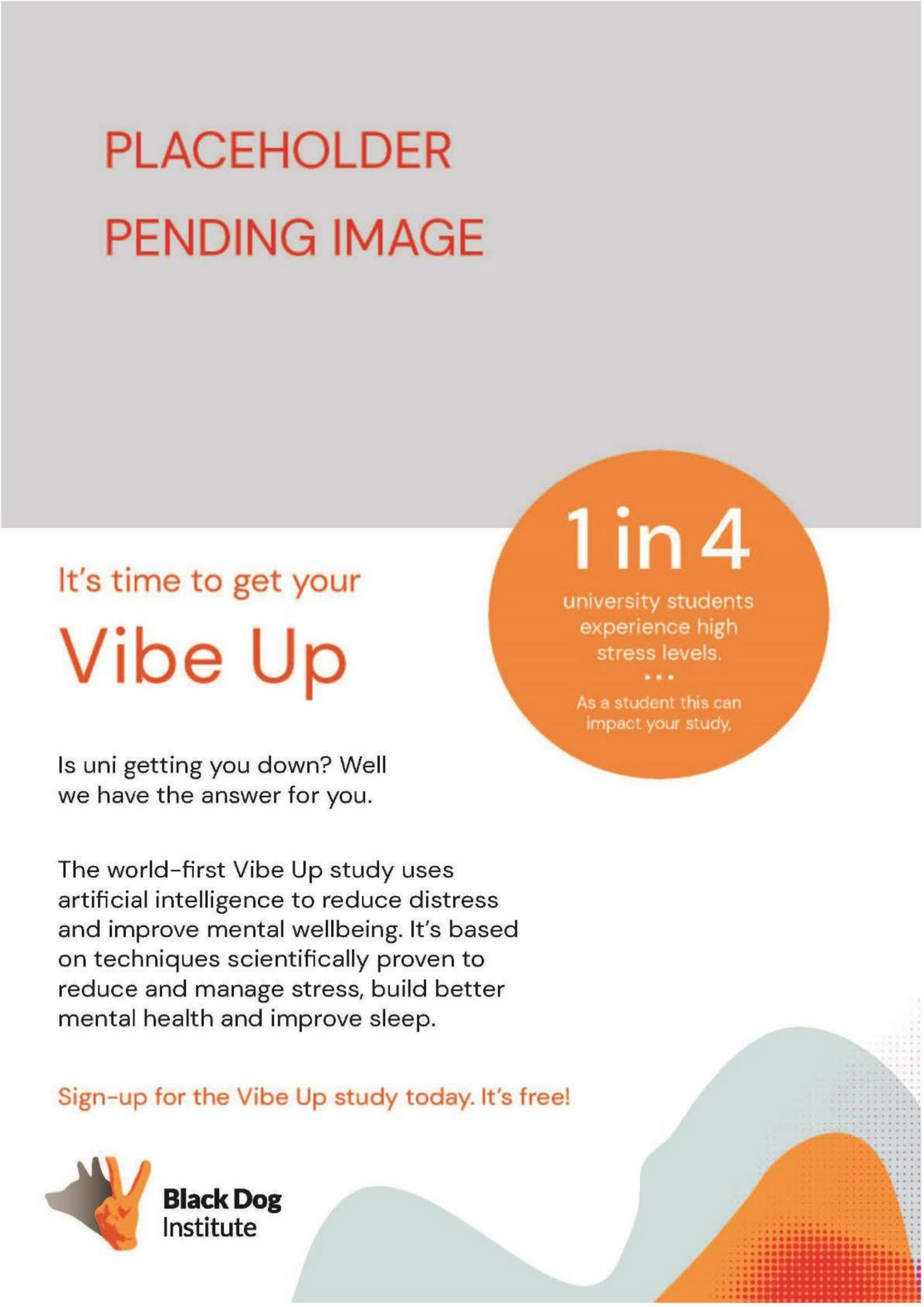

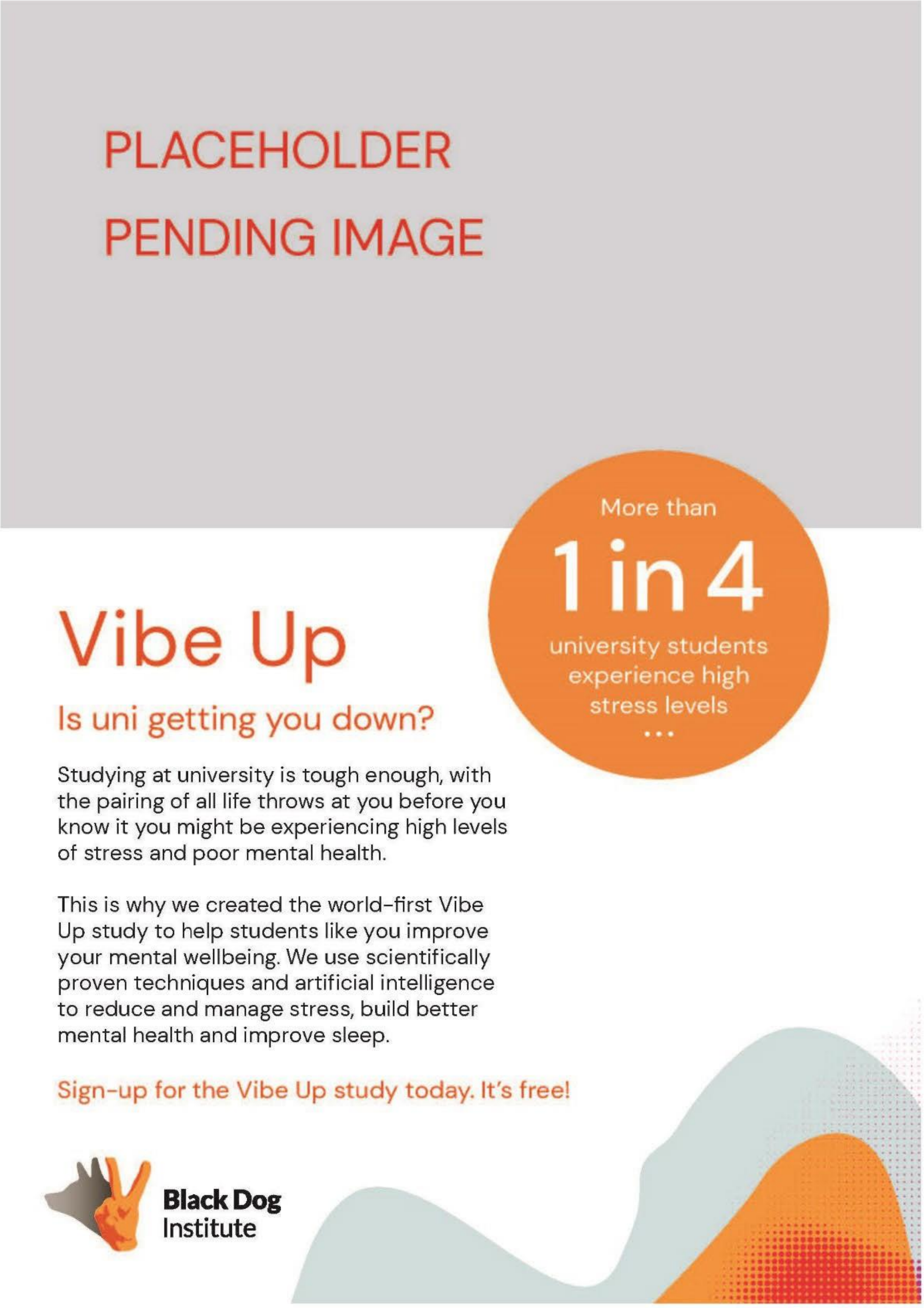

Page 6 of 11

HC Number: HC200466 **Version dated:** 26 Apr 2021

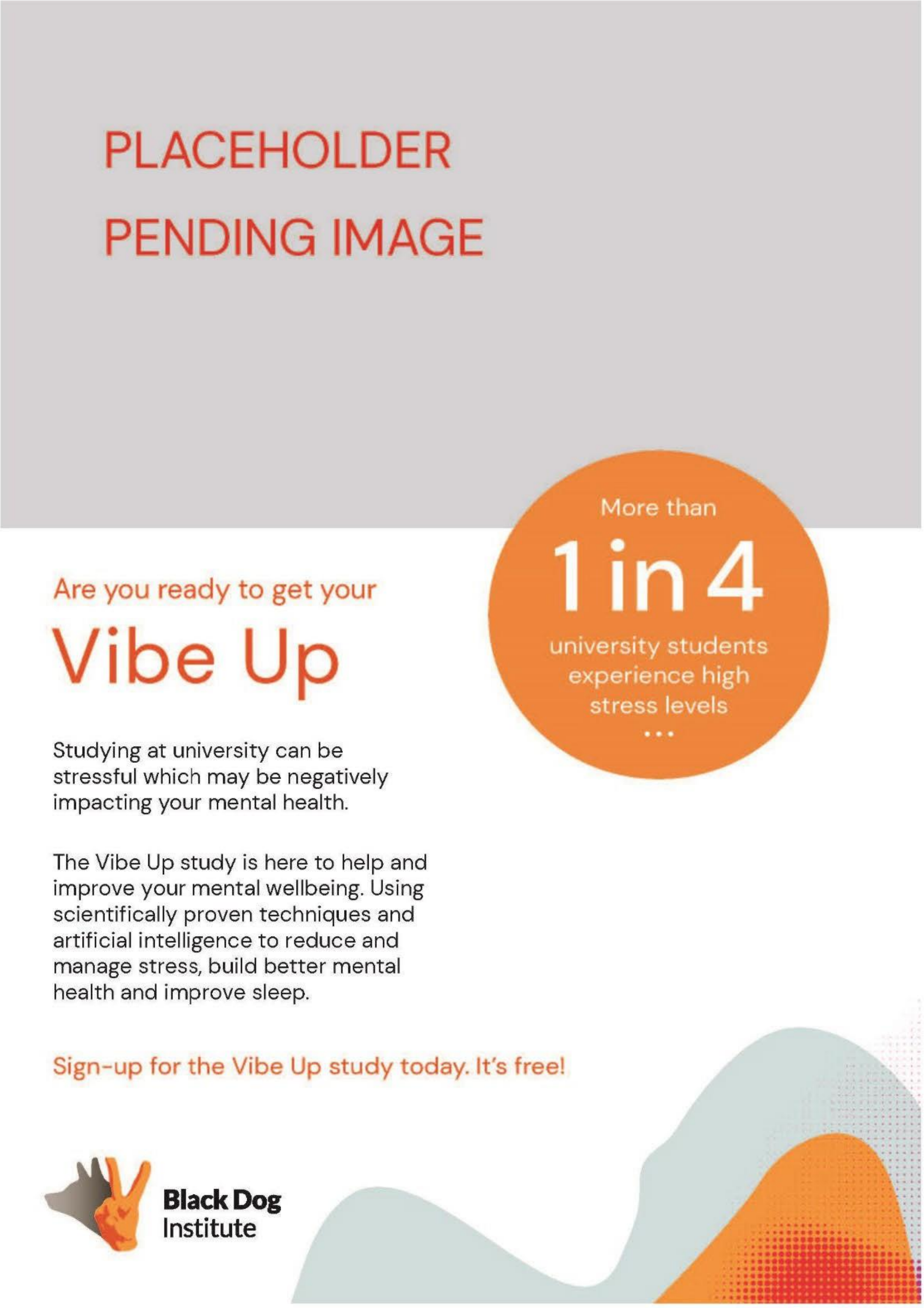

HC Number: HC200466

Page 7 of 11
Version dated: 26 Apr 2021

3. Email to potential partners

> Hi <<name>>,
>
> We don't have to tell you that studying at university can be a stressful time (even before the challenges of COVID19 and remote learning). **More than 1 in 4 university students experience high stress levels that negatively impacts their studies, relationships, and daily lives.** Your students may need help as these stressors can cause future mental health issues and educational institutions have a duty of care.
>
> The good news is that your students CAN feel and do better… with a little help.
>
> Black Dog Institute is launching a world-first research study to improve psychological distress and mental wellbeing in Australian university, TAFE and higher education students by identifying the best smartphone app self-help interventions using artificial intelligence (AI).
>
> Black Dog Institute is working with Deakin University in collaboration with:
>
> - *Macquarie University*
> - *UNSW*
> - *the Association for Psychological Science (APS)*
> - *the Australian Medical Association (AMA)*
> - *the Australian Government (funded by a $5M grant for Medical Research Future Fund for Artificial Intelligence in Health Scheme)*
>
> We'd like to work together with <<name of organisation>> too. The Vibe Up study runs from May 2021 to June 2022 and will engage 1,200 higher education students across Australia.
>
> Now is the perfect time to help your students get their Vibe Up. Please share assets from our Communications Pack <<insert link>> with your students via your networks and digital channels.
>
> If you'd like to learn more about the study, please contact vibeup@blackdog.org.au.
>
> All the very best,
>
> <<name>>

4. Vibe Up partner communications pack

The materials in **2 (Recruitment Materials)2.1** will be hosted on Black Dog Institute's website, to enable partners/advocates to share material to promote the study to potential participants. Below is a template for this webpage:

> Thanks for sharing vibe up. Over the next year, we're looking to recruit 1200 students currently studying at an Australian university, TAFE, college or other higher education institutions. To make it as easy as possible for our partners, we've put the below assets together so you can share the world-first study across social, email and on-campus.

> We hope that by participating in the Vibe Uup study, your students will learn strategies to combat stress, and improve wellbeing as they are guided through various engaging daily tasks over 30 days.
>
> Questions? Feel free to email us at vibeup@blackdog.org.au.
>
> 1. Social media tiles and copy
> 2. Email text for partner newsletters
> 3. Posters for campus

5. Recruitment landing page

Each recruitment advertisement will contain a link to a recruitment landing page, hosted on Black Dog Institute website. The landing page will contain brief information about the study and a direct link to commence the screening questionnaire and consent process. Below is the copy that will appear on the page:

> More than 1 in 4 university students experience high-stress levels that negatively impact their studies, relationships, and daily lives. Vibe up is a world-first study aiming to change this at scale.
>
> Through various engaging daily tasks (delivered using AI technology), you'll be guided through techniques taught by doctors and psychologists to help build better mental health. Spending just 15 minutes in the morning and night can help you better manage stress, worry, and anxiety, boost your mood, and keep you performing at your best.
>
> Once you've completed the study, you'll receive a $30 gift card to say thank you. We'll also send you a confidential, personalised report about your wellbeing with tailored suggestions for staying well.
>
> Who can participate?
>
> You will likely be eligible for this study if you are:
>
> - 18 years or older
> - Living in Australia
> - Fluent in English
> - Currently studying at an Australian university, TAFE, or other higher education institution
> - Experiencing feelings like elevated anxiety, stress, or low mood
> - The owner of an up-to-date smartphone, with an active phone number and internet access
>
> *Unfortunately, we are not able to take on participants who have experienced significant thoughts of suicide in the past month, have a diagnosis of psychosis or bipolar disorder, or who can't safely undertake physical activity.*

What does it involve?

The study is made up of a series of mini-trials over the next year. You will only be required to take part for 30 days.

1. Download the Vibe Up app

You'll be notified via SMS when the study commences and asked to download the app. The first group will begin on Thursday 20th May. We'll be running more mini-trials later in the year, until mid-2022.

2. Complete mood and wellbeing surveys (10 days)

You'll be prompted to complete short surveys at the start, during and end of the study to check-in on your mood. The first will need to be completed within 72 hours of invitation and each survey takes only 3-5 minutes to complete.

3. Complete daily activities (14 days)

You'll be prompted to complete daily activities developed by doctors and psychologists to either monitor, or improve your mood over the next 2 weeks depending on the group you are allocated to. Participants will unlock all activities at the end of the 30 days.

Are you ready to get your Vibe Up?

If you decide to take part in this study, you will first be required to complete a 10-minute survey to determine your eligibility and provide your consent.

If eligible, it might be 4 weeks until you can download the app, depending on when the next mini-trial starts. *See study schedule > [link]*

SEE IF I'M ELIGIBLE [link to screening survey]

6. Expression of interest

Enrolment into the study will be closed if there is a significant time delay until the next mini-trial. When enrolment is closed, the link to the self-directed screening/consent will be replaced by an expression of interest (EOI) form, where potential participants submit their preferred name, email address and current educational institution (to encourage EOIs only from current students, per study aims). People who complete the EOI will be sent the following email when study enrolments next open:

Hi <<name>>,

We're just touching base to let you know that the Vibe Up study has begun.

You can now register for the study, and will first be required to complete a 10-minute survey to determine your eligibility and provide your consent.

SEE IF I'M ELIGIBLE [link to screening survey]

Page 10 of 11
HC Number: HC200466
Version dated: 26 Apr 2021

Any questions? Reach us at vibeup@blackdog.org.au<br><br>All the best,<br><br>The Vibe Up team<br><br>Black Dog Institute<br><br>www.blackdog.org.au<br><br>vibeup@blackdog.org.au

Version history

Date	Summary of changes
01 Dec 2020	Document created.
06 April 2021	Material developed by BDI Marketing and Communications Team included.

Appendix 7 Participant Information Statement and Consent Form

Is provided as a separate document.

Appendix 8 Study Questionnaires

Version dated 22 Nov 2021. Validated standard instruments are shown in **bold**.

Id	Self-report questionnaire	Item count	Screening	Baseline	EMA	MId	Intervention	Post	Follow-up	Page
ELY	Basic eligibility	11	●							2
RTP	Recruitment pathway	2	●							5
CTD	Contact details	4	●							6
MED	Physical and mental health	8	●							7
KTEN	**Kessler Psychological Distress Scale, 10-item version**	10	●							9
SIDAS	**Extended Suicidal Ideation Attributes Scale**	6	●							11
DEM	Demographic details	10	●							14
WRK	Study and employment	4	●							16
PCQ	**Productivity Costs Questionnaire**	13	●						●	17
MHS	**Use of Mental Health Care Services**	13	●						●	19
PUWS	Prior use of wellbeing strategies	9	●							22
EQV	**EQ-5D-5L**	6	●						●	23
ReQoL	**Recovering Quality of Life**	11	●						●	25
SES	**Subjective Socioeconomic Status Scale**	1	●							26
AOD	**Abridged NIDA-Modified ASSIST Drug Screening Tool**	4	●							27
WBS	**Short Warwick Edinburgh Mental Wellbeing Scale**	7	●							28
PSS	**Multidimensional Scale of Perceived Social Support**	12	●							29
DASS	**Depression, Anxiety and Stress Scale, 21-item version**	21		●		●		●	●	30
PAVS	**Modified Physical Activity Vital Sign**	2		●		●		●	●	32
PSQI	**Abridged Pittsburgh Sleep Quality Index**	1		●		●		●	●	32
MIND	Mindfulness single item questionnaire	1		●		●		●	●	32
PANAS	**Modified Positive and Negative Affect Schedule, Short Form**	13			●					33
BHV	Behavioural intentions	2			●					35
CEQ	**Abridged Credibility and Expectancy Questionnaire**	2				●				36
URC	**Abridged Revised University of Rhode Island Change Assessment Scale**	6				●				37
LOG	Daily log of engagement with intervention	1					●			38
EXP	Within-study exposures questionnaire	4						●		39
UX	UX questionnaire	9						●		40

Response coding

Questionnaires and questions should always be identified by the unique identifier codes specified in this document. For closed-response questions, items must be coded using the scheme described in the presentation column for that item.

Basic eligibility (ELY)

☒ Screening	☐ Baseline	☐ EMA	☐ Mid	☐ Intervention	☐ Post	☐ Follow-up
						Up to 11 questions

Source: Bespoke questions.

Id	Question	Presentation
ELY1	How old are you?	Dropdown list with closed choice options: 'under 18', then numeric integers from 18 to 120.
ELY2a	Are you **currently registered** as a **student** at an Australian university, TAFE or other higher education institution?	2-item closed choice: No (0), Yes (1). User sees: words only.

*If the response to ELY1 is <18 or response to ELY2a is 0 (No) then participant is **ineligible**.*

ELY2b	Which of the following best describes you?	4-item closed choice: Undergraduate or TAFE student (0), Postgraduate coursework student (1), Postgraduate research student (2), User sees: words only.
ELY2c	Which academic institution are you **currently** registered at?<br><br>If you are registered at multiple institutions, pick the one where you spend most of your time.	Free text, max 128 characters. Ideally with autocomplete from the TEQSA national register. Validation: non-null.
ELY3a	Where do you currently live?	2-item closed choice: Australia (0), Somewhere else (1). User sees: words only.

*If the response to ELY3a is 1 (Somewhere else) then participant is **ineligible**.*

ELY3b	In the **next 2 months**, are you planning to travel outside Australia?	2-item closed choice: No (0), Yes (1). User sees: words only.

*If the response to ELY3b is 1 (Yes) then participant is **ineligible**.*

ELY4	How do you rate your English ability?	3-item closed choice: Basic (0), Advanced (1), Fluent/Native speaker (2). User sees: words only.

*If the response to EL4 is 1 (Basic) then participant is **ineligible**.*

ELY5a	Do you have your own smartphone with an active mobile number and internet access?	2-item closed choice: No (0), Yes, Android smartphone (1), Yes, iPhone (2), Yes, Other

		smartphone (3). User sees: words only.

*If the response to EL5a is 0 (No) or 3 (Other smartphone) then participant is **ineligible**.*

If the response to EL5a is 1 (Android smartphone):

ELY5b	Does your smartphone run **Android 5.0** (Lollipop) or higher?<br><br>If you bought your smartphone new within the past 2 years, you can assume it does.	2-item closed choice: No (0), Yes (1). User sees: words only.

If the response to EL5a is 2 (iPhone):

ELY5b	Do you have an **iPhone 6S or higher**?	2-item closed choice: No (0), Yes (1). User sees: words only.

*If the response to either version of EL5b is 0 (No) then participant is **ineligible**.*

ELY6a	Have you previously registered to take part in this study?	2-item closed choice: No (0), Yes (1). User sees: words only.
ELY6b	In the **next 2 months**, do you expect any major events or disruptions coming up that might make it difficult to take part (or continue taking part) in this study?	2-item closed choice: No (0), Yes (1). User sees: words only.
ELY7	If you were allocated to the physical activity treatment, is there anything that would prevent you from safely taking part?<br><br>One of the brief treatments for distress in this study involves exercise. This is self-guided so you can control the intensity and type of activity involved.<br><br>If you have a heart or lung problem that interferes with exercise, you should talk to your GP or specialist before agreeing to take part in this study.	2-item closed choice: No (0), Yes (1). User sees: words only.

*If either response to ELY6a, ELY6b or ELY7 is 1 (Yes) then participant is **ineligible**.*

Branching: See conditions triggering ineligibility, above. Participants triggering these conditions will be shown the ineligibility message below and screening will stop.

ELY ineligibility message

> **Unfortunately, it looks like you aren’t eligible to take part in the Vibe Up study**
>
> We’re looking for Australian university students aged 18 or older who are fluent in English, have an up-to-date personal smartphone (Android 5/iPhone 6S or higher) and who can commit to taking part in the study at some point over the next 2 months.

To join the Vibe Up study you also have to be able to undertake physical activity safely.

We're sorry you aren't able to take part. Thank you for your interest in Vibe Up!

If you are concerned about your mental health, help is available.

**Organisation**	Lifeline<br>*Crisis support and suicide prevention*
**Telephone**	13 11 14 available 24/7
**Online chat**	www.lifeline.org.au/crisis-chat available 19:00 – 23:59 AEDT/AEST
**Web**	www.lifeline.org.au/get-help

**Organisation**	Suicide Call Back Service<br>*Counselling and support for people affected by suicide*
**Telephone**	1300 659 467 available 24/7
**Online chat**	www.suicidecallbackservice.org.au/phone-and-online-counselling/suicide-call-back-service-online-counselling available 24/7
**Web**	www.suicidecallbackservice.org.au

**Organisation**	Beyond Blue<br>*Suport and information for people experiencing depression or anxiety*
**Telephone**	1300 22 4636 available 24/7
**Online chat**	online.beyondblue.org.au available 11:00 – 23:59 AEDT/AEST
**Web**	www.beyondblue.org.au

For information on other online mental health services that are available, including support services tailored to specific communities (e.g. LGBTIQ+, Aboriginal and Torres Strait Islander peoples, carers), and services specific to the state you live in, visit: www.headtohealth.gov.au

---

If you have any questions about your application, please contact vibeup@blackdog.org.au.

For information about other ways to get involved with Black Dog Institute, please visit: www.blackdoginstitute.org.au/get-involved

Recruitment pathway (RTP)

☒ Screening	☐ Baseline	☐ EMA	☐ Mid	☐ Intervention	☐ Post	☐ Follow-up
						2 questions

Source: Bespoke questions.

Id	Question	Presentation
RTP1a	How did you hear about this study?<br><br>This information will help us get the word out to more students.	12-item multiple choice: Facebook ad (0), Facebook post from a person or group that I follow (1), Instagram ad (2), Instagram post from a person or group that I follow (3), Other social media (4), Email (5), Poster/flyer (6), University/College class or event (7), Press/media (8), Health professional (9), Black Dog Institute website (10), Other (11). User sees: words only. Validation: maximum one selection.

If response to RTP1a is 11 (Other):

RTP1b	Please specify:	Free text, max 128 characters.

Contact details (CTD)

☒ Screening	☐ Baseline	☐ EMA	☐ Mid	☐ Intervention	☐ Post	☐ Follow-up
						4 questions

Source: Bespoke questions.

Id	Question	Presentation
In case we need to contact you about anything to do with the study…		
CTD1	What is your preferred name?	Free text, max 128 characters. Validation: non-null.
CTD2a	What is your mobile number?<br>Your mobile number will be your login for the study app. We'll also send instructions via SMS.	Numeric integer input, max 12 characters. Validation: valid Australian mobile number, matched values in CTD2a/b
CTD2b	Confirm your mobile number by typing it again.	
CTD3	What is your email address?	Free text, max 128 characters. Validation: valid email address.

Physical and mental health (MED)

☒ Screening	☐ Baseline	☐ EMA	☐ Mid	☐ Intervention	☐ Post	☐ Follow-up
						Up to 8 questions

Source: Bespoke questions.

Id	Question	Presentation
MED1	Have you ever been diagnosed by a professional (e.g., a doctor) with a **chronic physical health condition**?<br>Chronic physical health conditions are things like asthma, diabetes, cerebral palsy or sickle cell anaemia that can be managed but not cured.	4-item closed choice; No (0), Yes (1), Unsure (2), Prefer not to say (3). User sees: words only.
MED2	Have you ever been diagnosed by a professional (e.g., a doctor or psychologist) with a **mental health** condition?	

If the response to MED2 is 1 (Yes):

MED3	Which of the following condition(s) have you **ever been** diagnosed with? Pick all that apply.	Multiple choice (e.g. checkbox):<br>Major Depression (1), Bipolar Disorder (2), Social Anxiety Disorder or Social Phobia (3), Generalised Anxiety Disorder (4), Obsessive Compulsive Disorder (5), Panic Disorder (6), Eating Disorder (7), Alcohol Use Disorder (8), Substance Use Disorder (9), Attention Deficit Hyperactivity Disorder (ADHD) (10), Post-Traumatic Stress Disorder (PTSD) (11), Schizophrenia or Psychosis (12), Another condition not listed here (13). User sees: words only.
MED5	When were you **first diagnosed** with a mental health condition?	Month and year picker. Validation: less than current date.

If the response to MED2 is 1 (Yes) ***and*** *if participant selects either 2 (Bipolar Disorder) or 12 (Schizophrenia or Psychosis) to MED3:*

MED4	Is your mental health condition currently **active**?<br><br>By active we mean that you are experiencing symptoms, receiving treatment and/or seeing a healthcare professional regularly for this condition.	2-item closed choice: No (0), Yes (1). User sees: words only.

Branching: If participant responds 1 (Yes) to MED4 then they are **ineligible** on risk grounds, will be shown the ineligibility message below and screening will stop.

MED ineligibility message

Unfortunately, it looks like you aren't eligible to take part in the Vibe Up study

The brief self-help treatments like those in the Vibe Up app are not the best option for people currently experiencing bipolar disorder, schizophrenia or psychosis.

If you haven't already, we suggest you seek support and advice from a GP (General Practitioner), who can also connect you with local services in your area.

We're sorry you aren't able to take part. Thank you for your interest in Vibe Up!

If you are concerned about your mental health, help is available.

**Organisation**	Lifeline<br>*Crisis support and suicide prevention*
**Telephone**	13 11 14 available 24/7
**Online chat**	www.lifeline.org.au/crisis-chat available 19:00 – 23:59 AEDT/AEST
**Web**	www.lifeline.org.au/get-help

**Organisation**	Suicide Call Back Service<br>*Counselling and support for people affected by suicide*
**Telephone**	1300 659 467 available 24/7
**Online chat**	www.suicidecallbackservice.org.au/phone-and-online-counselling/suicide-call-back-service-online-counselling available 24/7
**Web**	www.suicidecallbackservice.org.au

**Organisation**	Beyond Blue<br>*Support and information for people experiencing depression or anxiety*
**Telephone**	1300 22 4636 available 24/7
**Online chat**	online.beyondblue.org.au available 11:00 – 23:59 AEDT/AEST
**Web**	www.beyondblue.org.au

For information on other online mental health services that are available, including support services tailored to specific communities (e.g. LGBTIQ+, Aboriginal and Torres Strait Islander peoples, carers), and services specific to the state you live in, visit: www.headtohealth.gov.au

---

If you have any questions about your application please contact vibeup@blackdog.org.au.

For information about other ways to get involved with Black Dog Institute, please visit: www.blackdoginstitute.org.au/get-involved

Kessler Psychological Distress Scale, 10-item version (KTEN)

☒ Screening	☐ Baseline	☐ EMA	☐ Mid	☐ Intervention	☐ Post	☐ Follow-up
						Up to 10 questions

Source: [53] with scoring cut-offs based on [42].

Id	Question	Presentation
In the **past 4 weeks**…		
KTEN1	About how often did you feel tired out for no good reason?	5-level rating scale; None of the time (1) – A little of the time (2) – Some of the time (3) – Most of the time (4) – All of the time (5). User sees: words only.
KTEN2	About how often did you feel nervous?	

If response to KTEN2 is <u>not</u> 1 (None of the time):

KTEN3	About how often did you feel so nervous that nothing could calm you down?	As above.

For all participants:

KTEN4	About how often did you feel hopeless?	As above.
KTEN5	About how often did you feel restless or fidgety?	

If response to KTEN5 is <u>not</u> 1 (None of the time):

KTEN6	About how often did you feel so restless you could not sit still?	As above.

For all participants:

KTEN7	About how often did you feel depressed?	As above.
KTEN8	About how often did you feel that everything was an effort?	
KTEN9	About how often did you feel so sad that nothing could cheer you up?	
KTEN10	About how often did you feel worthless?	

Scoring:

- Total score: simple sum of item scores.
- If not shown, KTEN3 and KTEN6 are automatically scored as 1 (None of the time).
- Possible range: 10 – 50 inclusive.
- Interpretation (CRUfAD ranges from [42]):

Total score	Level of distress
10-19	Likely to be well
20-24	Likely to have a mild mental disorder
25-29	Likely to have a moderate mental disorder

30-50	Likely to have a severe mental disorder

Branching: If KTEN total score is < 20 (Likely to be well) then participant is unlikely to have psychological distress, and is therefore **ineligible**. They will be shown the ineligibility message below and screening will stop

KTEN ineligibility message

Unfortunately, it looks like you aren't eligible to take part in the Vibe Up study

The Vibe Up study is aimed at people experiencing moderate or higher levels of psychological distress.

Your responses to the screening questions suggest that you aren't currently experiencing distress at this level.

We're sorry you aren't able to take part. Thank you for your interest in Vibe Up!

If you are concerned about your mental health, help is available.

**Organisation**	Lifeline<br>*Crisis support and suicide prevention*
**Telephone**	13 11 14 available 24/7
**Online chat**	www.lifeline.org.au/crisis-chat available 19:00 – 23:59 AEDT/AEST
**Web**	www.lifeline.org.au/get-help

**Organisation**	Suicide Call Back Service<br>*Counselling and support for people affected by suicide*
**Telephone**	1300 659 467 available 24/7
**Online chat**	www.suicidecallbackservice.org.au/phone-and-online-counselling/suicide-call-back-service-online-counselling available 24/7
**Web**	www.suicidecallbackservice.org.au

**Organisation**	Beyond Blue<br>*Support and information for people experiencing depression or anxiety*
**Telephone**	1300 22 4636 available 24/7
**Online chat**	online.beyondblue.org.au available 11:00 – 23:59 AEDT/AEST
**Web**	www.beyondblue.org.au

For information on other online mental health services that are available, including support services tailored to specific communities (e.g. LGBTIQ+, Aboriginal and Torres Strait Islander peoples, carers), and services specific to the state you live in, visit: www.headtohealth.gov.au

---

If you have any questions about your application please contact vibeup@blackdog.org.au.

For information about other ways to get involved with Black Dog Institute, please visit: www.blackdoginstitute.org.au/get-involved

Extended Suicidal Ideation Attributes Scale (SIDAS)

☒ Screening	☐ Baseline	☐ EMA	☐ Mid	☐ Intervention	☐ Post	☐ Follow-up
						Up to 6 questions

Source: [43]

Id	Question	Presentation
SIDAS1	In the **past month**, how often have you had thoughts about suicide?	11-level rating scale; Never (0) – (1) – (2) – […] – (8) – (9) – Always (10). User sees: words and numbers.

If response to SIDAS is <u>*not*</u> *0 (Never):*

SIDAS2	In the **past month**, how much control have you had over these thoughts?	11-level rating scale; No control (0) – (1) – (2) – […] – (8) – (9) – Full control (10). User sees: words and numbers.
SIDAS3	In the **past month**, how close have you come to making a suicide attempt?	11-level rating scale; Not close at all (0) – (1) – (2) – […] – (8) – (9) – Made an attempt (10). User sees: words and numbers.
SIDAS4	In the **past month**, to what extent have you felt tormented by thoughts about suicide?	11-level rating scale; Not at all (0) – (1) – (2) – […] – (8) – (9) – Extremely (10). User sees: words and numbers.
SIDAS5	In the **past month**, how much have thoughts about suicide interfered with your ability to carry out daily activities, such as school, chores or social activities?	

For all participants:

SAT	Have you ever made a suicide attempt?	3-item closed choice: No, never (0), Yes, once (1), Yes, more than once (2). User sees: words only.

Scoring:

- Total score: sum of item scores for SIDAS1 and SIDAS3-5 plus reversed item score for SIDAS2.
- If not shown, SIDAS2 is assigned a (reversed) score of 0 (Full control) and SIDAS 3-5 are assigned a score of 0 (Not close at all/Never). Question SAT is not part of the SIDAS as is **not** included in scoring.
- Possible range: 0 – 50 inclusive.
- Interpretation: scores ≥ 21 indicate a high risk of suicide behaviour[43].

Branching: If SIDAS total score is ≥ 21 then participant is **ineligible** on risk grounds, will be shown the ineligibility message below and screening will stop. Offered support options will include opt-in psychologist call-back.

SIDAS ineligibility message including opt-in offer of psychologist call-back

Support for people experiencing suicidal thoughts

The brief treatments we are testing in Vibe Up aren't the best option for people who are experiencing suicidal thoughts.

It's really important to seek immediate help for thoughts of death or harming yourself. With help, you can overcome these thoughts and stay safe.

If your life or someone else's life is in danger, call 000 now.

The services listed below offer 24/7 crisis support. You can speak to someone via phone or online chat.

You could also consider seeking support and advice from a GP (General Practitioner).

If you'd like to speak to a trained clinician from Black Dog Institute, we can call you back. To choose this option, tick the box below. We'll respond within one working day (please contact one of the below crisis support services if you need immediate help).

☐ Yes, I would like to receive a callback from a trained clinician from Black Dog Institute.

We're sorry that you aren't eligible to take part in the study. Thank you for your interest in Vibe Up!

Get help right now (24/7 crisis support)

**Organisation**	Lifeline<br>*Crisis support and suicide prevention*
**Telephone**	13 11 14 available 24/7
**Online chat**	www.lifeline.org.au/crisis-chat available 19:00 – 23:59 AEDT/AEST
**Web**	www.lifeline.org.au/get-help

**Organisation**	Suicide Call Back Service<br>*Counselling and support for people affected by suicide*
**Telephone**	1300 659 467 available 24/7
**Online chat**	www.suicidecallbackservice.org.au/phone-and-online-counselling/suicide-call-back-service-online-counselling available 24/7
**Web**	www.suicidecallbackservice.org.au

**Organisation**	Beyond Blue<br>*Support and information for people experiencing depression or anxiety*
**Telephone**	1300 22 4636 available 24/7
**Online chat**	online.beyondblue.org.au available 11:00 – 23:59 AEDT/AEST
**Web**	www.beyondblue.org.au

For information on other online mental health services that are available, including support services tailored to specific communities (e.g. LGBTIQ+, Aboriginal and Torres Strait Islander peoples, carers), and services specific to the state you live in, visit: www.headtohealth.gov.au

---

If you have any questions about your application please contact vibeup@blackdog.org.au.

For information about other ways to get involved with Black Dog Institute, please visit: www.blackdoginstitute.org.au/get-involved

Demographic details (DEM)

☒ Screening	☐ Baseline	☐ EMA	☐ Mid	☐ Intervention	☐ Post	☐ Follow-up
						Up to 10 questions

Source: ABS Draft Standard for Sex, Gender, Variation of Sex Characteristics and Sexual Orientation Variables, 2020 (Unpublished), [54] and [55].

Remarks: DEM1b, DEM2b and DEM3b are included principally to afford (potentially marginalised) individuals the right to assert their identity.

Id	Question	Presentation
DEM1a	How do you describe your gender?<br>This information will help our research understand if different groups of people find different treatments more useful.	5-item closed choice: Woman or female (0), Man or male (1), Non-binary (2), I use a different term (3), Prefer not to say (4). User sees: words only.

If response to DEM1a is 3 (I use a different term):

DEM1b	Please specify:	Free text, max 64 characters.

For all participants:

DEM2a	What was your sex recorded at birth?	3-item closed choice: Female (0), Male (1), Another term (2), Prefer not to say (3). User sees: words only.

If response to DEM2a is 2 (Another term):

DEM2b	Please specify:	Free text, max 64 characters.

For all participants:

DEM3a	How do you describe your sexual orientation?<br>This information will help our research understand if different groups of people find different treatments more useful.	6-item closed choice: Straight (heterosexual) (0), Gay or lesbian (1), Bisexual (2), I use a different term (3), Don't know (4), Prefer not to say (5). User sees: words only.

If response to DEM3a is 3 (I use a different term):

DEM3b	Please specify:	Free text, max 64 characters.

For all participants:

DEM4	Are you of Aboriginal or Torres Strait Islander origin?	5-item multiple choice: No (0), Yes, Aboriginal (1), Yes, Torres

	If you are of both Aboriginal and Torres Strait Islander origin, mark both 'Yes' boxes.	Strait Islander (2), Don’t know (3), Prefer not to say (4). User sees: words only. Validation: If options 0, 3 or 4 are selected then no other option can be chosen.

DEM5	What is your ancestry? Pick up to two options.	12-item multiple choice: Australian (0), Chinese or Cantonese (1), Dutch (2), English (3), German (4), Greek (5), Indian (6), Irish (7), Italian (8), Scottish (9), Vietnamese (10), Another ancestry (11), Prefer not to say (12). User sees: words only. Validation: Maximum two concurrent selections. If item 11 is chosen, no other items can be selected.
DEM6	What is the language that you mostly speak at home?	11-item closed choice: Arabic (0), Cantonese (1), English (2), Greek (3), Hindi (4), Italian (5), Mandarin (6), Punjabi (7), Spanish (8), Vietnamese (9), Another language (10), Prefer not to say (11). User sees: words only.
DEM7	What is your postcode?	Numeric integer input, max 4 characters

Study and employment (WRK)

☒ Screening	☐ Baseline	☐ EMA	☐ Mid	☐ Intervention	☐ Post	☐ Follow-up
						Up to 3 questions

Source: Bespoke questions.

Id	Question	Presentation
WRK1	Are you studying in Australia as an international student?	2-item closed choice: No (0), Yes (1). User sees: words only.
WRK2a	What is your Weighted Average Mark (WAM) or Grade Point Average (GPA) for your **current** degree or qualification?<br><br>The WAM or GPA provides an indication of your overall academic performance. Please select the option that your institution uses.	6-item closed choice with options: WAM (0), GPA – 4.0 scale (1), GPA – 7.0 scale (2), Don't know (3), Don't have one (4), Prefer not to say (5). User sees: numbers/words only.

If response to WRK2a is 0 (WAM):

Id	Question	Presentation
WRK2a	What is your Weighted Average Mark (WAM) for your **current** degree or qualification?	11-item closed choice: Below 50 (0), 50-54 (1), 55-59 (2), 60-64 (3), 65-69 (4), 70-74 (5), 75-79 (6), 80-84 (7), 85-89 (8), 90-94 (9), 95 or higher (10), User sees: words only.

If response to WRK2a is 1 (GPA – 4.0 scale):

Id	Question	Presentation
WRK2a	What is your Grade Point Average (GPA – 4 point scale) for your **current** degree or qualification?	8-item closed choice: Below 1.0 (0), 1.0-1.4 (1), 1.5-1.9 (2), 2.0-2.4 (3), 2.5-2.9 (4), 3.0-3.4 (5), 3.5-3.9 (5), 4.0 (7). User sees: numbers/words only.

If response to WRK2a is 2 (GPA – 7.0 scale):

Id	Question	Presentation
WRK2a	What is your Grade Point Average (GPA – 7 point scale) for your **current** degree or qualification?	8-item closed choice: Below 4.0 (0), 4.0-4.4 (1), 4.5-4.9 (2) 5.0-5.4 (3), 5.5-5.9 (4), 6.0-6.4 (5), 6.5-6.9 (6), 7.0 (7). User sees: numbers/words only.

Productivity Costs Questionnaire (PCQ)

☒ Screening	☐ Baseline	☐ EMA	☐ Mid	☐ Intervention	☐ Post	☒ Follow-up
						Up to 13 questions

Source: [56].

Note: at follow-up, the phrasing of the questionnaire is adapted to reference "the 12 weeks after starting the Vibe Up app" rather than "the last 12 weeks".

Id	Question	Presentation
The purpose of the following questions is to collect information on your capacity to undertake paid and unpaid work. The questions refer to you circumstances in the **last 12 weeks**.		
PCQ1	What did you do? (select all that apply)	5-item multiple choice: I went to university (0), I was employed (1), I was self employed (2), I was unemployed (3), I was unable to work (4). User sees: words only.
PCQ2	Did you have a paying job?	2-item closed choice: No (0), Yes (1). User sees: words only.

If response to PCQ2 is 1 (Yes):

The following questions refer to your work. That is, work that you get paid for.		
PCQ3	What was your occupation over the last 12 weeks?	Free text, max 128 characters.
PCQ4	How many hours a week (on average) did you work over the last 12 weeks?<br>Count only the hours that you get paid.	Numeric fill-in/pick 'X hours.<br>Validation: X is integral in the range 0 ≤ X ≤ 100.
PCQ5	How many days a week did you work (on average) over the last 12 weeks? Count only the days that you get paid	Numeric fill-in/pick 'X days'.<br>Validation: X is integral in the range 0 ≤ X ≤ 7.
PCQ6	Have you missed work in the last 12 weeks because of psychological problems?<br><br>If *1 (Yes)* is selected, numeric fill-in with 'X days (only count the missed work days in the last 12 weeks)'. (PCQ6B)	2-item closed choice: No (0), Yes (1). User sees: words only.<br><br>Numeric fill-in/pick.<br>Validation: X is integral in the range 0 ≤ X ≤ 28.

If response to PCQ2 and PCQ6 is 1 (Yes):

PCQ7	Did you miss work earlier than the last 12 weeks because of psychological problems?<br><br>This is referring to <u>one whole uninterrupted period of missed work</u> from psychological problems.	2-item closed choice: No (0), Yes (1). User sees: words only.

For all participants:

PCQ8	During the last 12 weeks have there been days when you worked but during this time you were bothered by psychological problems?	2-item closed choice: No (0), Yes (1). User sees: words only.

If response to PCQ8 is 1 (Yes):

PCQ9	How many days at work were you bothered by psychological problems? (Only count the days at work in the last 12 weeks)	Numeric fill-in/pick 'X work days'. Validation: X is integral in the range 0 ≤ X ≤ 28.
PCQ10	On days that you were bothered by psychological problems, was it more difficult to get as much work finished as you normally do? On these days how much work could you do on average?	11-item likert: On these days I could not do anything, 0 (0), 1 (1), 2 (2), 3 (3), 4 (4), I was able to do half as much as I normally do, 5 (5), 6 (6), 7 (7), 8 (8), 9 (9), I was able to do just as much as I normally do (10). User sees: numbers and word anchors only.

For all participants:

<table>
<tr><td colspan="3">Even for unpaid work, you can be bothered by psychological problems. Sometimes as a result you (might) do less. For example you do less voluntary work, or are unable to help family or friends with their tasks. The following questions refer to this.</td></tr>
<tr><td>PCQ11</td><td>Were there days in which you did less unpaid work because of psychological problems? Only days in the last four weeks.</td><td>2-item closed choice: No (0), Yes (1). User sees: words only.</td></tr>
</table>

If response to PCQ10 is 1 (Yes):

PCQ12	How many days did this happen? Only count the days in the last 12 weeks.	Numeric fill-in/pick 'X days'. Validation: X is integral in the range 0 ≤ X ≤ 28.
PCQ13	How many hours of unpaid work (in total) did you miss because of psychological problems? Only count the hours in the last 12 weeks.	Numeric fill-in/pick 'X hours. Validation: X is integral in the range 0 ≤ X ≤ 100.

Use of Mental Health Care Services (MHS)

☒ Screening	☐ Baseline	☐ EMA	☐ Mid	☐ Intervention	☐ Post	☒ Follow-up
						13 questions

Source: provided by collaborators at Macquarie University Centre for the Health Economy, based on [57].

Note: at follow-up, the phrasing of the questionnaire is adapted to reference "the 12 weeks after starting the Vibe Up app" instead of "the 12 weeks prior to applying to take part in this study".

Id	Question	Presentation
The purpose of this survey is to collect information on your use of health and community care services. This includes hospital services, medical services, allied health services, community based services, and diagnostic services.<br><br>We would like to ask you some questions on the number of times you have used hospital services in the 12 weeks prior to applying to take part in this study.		
MHS1	In the last 12 weeks, how many times did you visit the Emergency Department of a hospital for mental health concerns?	7-item closed choice: 0 (0), 1 (1), 2 (2), 3 (3), 4 (4), 5 (5), 'More than 5. If so how many?' (6). User sees: words/numbers only.<br>If user selects 6: Numeric fill-in/pick 'X times'. Validation: X is integral in the range 5 < X ≤ 31.
MHS2	In the last 12 weeks, how many times were you admitted to a hospital where you stayed overnight for at least one night for mental health services?	
MHS3	In the last 12 weeks, how many times did you visit a hospital outpatient clinic for mental health services?	
We would now like to ask you some questions on the number of times you have used specialist services for your mental health in the 12 weeks prior to applying to take part in this study.		
MHS4	In the last 12 weeks, how many times did you visit a GP for mental health concerns?	7-item closed choice: 0 (0), 1 (1), 2 (2), 3 (3), 4 (4), 5 (5), 'More than 5. If so how many?' (6). User sees: words/numbers only.<br>If user selects 6: Numeric fill-in/pick 'X times'. Validation: X is integral in the range 5 < X ≤ 31.
MHS5	In the last 12 weeks, how many times did you visit a psychologist?	
MHS6	In the last 12 weeks, how many times did you visit a psychiatrist?	
MHS7	In the last 12 weeks, how many times did you visit an occupational therapist?	
We would now like to ask you some questions on the number of times you have used an online self-help service for your mental health prior to applying to take part in this study. There are many common online services, such as myCompass, Mind Spot and This Way Up. There may also be an online wellbeing program offered by your University.		
MHS8	In the last 12 weeks, how many times did you undertake a lesson from an online self-help service for your mental health?	7-item closed choice: 0 (0), 1 (1), 2 (2), 3 (3), 4 (4), 5 (5), 'More than 5. If so how many?' (6). User sees: words/numbers only.<br>If user selects 6: Numeric fill-in/pick 'X times'. Validation: X is integral in the range 5 < X ≤ 100.
MHS9	In the last 12 weeks, how many hours did you spend on reviewing content and undertaking activities associated with an online self-help service for your mental health?	7-item closed choice: 0 (0), 1-3 hours (1), 4-6 hours (2), 7-9 hours (3), 10-12 hours (4), 13-15 hours (5), More than 15 hours (6).
MHS10	In the last 12 weeks, how much did you spend on an online self-help service for your mental health?	7-item closed choice: 0 (0), $1-$49 (1), $50-$99 (2), $100-

1762

		$149 (3), $150-$199 (4), $200-$249 (5), $250 or more (6).
We would now like to ask you some questions on the number of times you have used community services for your mental health in the 12 weeks prior to applying to take part in this study.		
MHS11	In the last 12 weeks, how many times did you participate in a group support service for your mental health?	7-item closed choice: 0 (0), 1 (1), 2 (2), 3 (3), 4 (4), 5 (5), 'More than 5. If so how many?' (6). User sees: words/numbers only.<br>If user selects 6: Numeric fill-in/pick 'X times'. Validation: X is integral in the range 5 < X ≤ 31.
MHS12	In the last 12 weeks, how many times did you use a counselling service offered by your University for your mental health?	
We would now like to ask you a question on your use of medicines for your mental health in the 12 weeks prior to applying to take part in this study.		
MHS13	In the last 12 weeks, how many weeks did you use prescribed medicines for your mental health?	7-item closed choice: 0 weeks (0), 1-2 weeks (1), 3-4 weeks (2), 5-6 weeks (3), 7-8 weeks (4), 9-10 weeks (5), 11-12 weeks (6).

Prior use of wellbeing strategies (PUWS)

☒ Screening	☐ Baseline	☐ EMA	☐ Mid	☐ Intervention	☐ Post	☐ Follow-up
						9 questions

Source: Bespoke questions.

We would like to know more about your previous use of different wellbeing strategies.

Id	Question	Presentation
PUWS1	Have you ever used a smartphone app related to the following:<br>a. Mindfulness<br>b. Physical activity/exercise<br>c. Tips to improve your sleep	2-item closed choice: No (0), Yes (1). User sees: words only.
PUWS2	Have you ever taken part in a group program, online course or individual appointments related to the following:<br>a. Mindfulness<br>b. Physical activity/exercise<br>c. Tips to improve your sleep	2-item closed choice: No (0), Yes (1). User sees: words only.
PUWS3	How many days in the past week, have you:<br>a. Practised mindfulness<br>b. Engaged in physical activity/exercise<br>c. Used strategies to improve sleep	Numeric fill-in/pick 'X day(s)'. Validation: X is integral in the range $0 \le X \le 7$.

EQ-5D-5L (EQV)

☒ Screening	☐ Baseline	☐ EMA	☐ Mid	☐ Intervention	☐ Post	☒ Follow-up
						6 questions

Source: [58] including the EQ-VAS question.

Id	Question	Presentation
The following sentence is displayed above each question label:<br>Please tick the ONE box that best describes your health TODAY.		
EQV1	**MOBILITY**	5-item closed choice: I have no problems in walking about (1), I have slight problems in walking about (2), I have moderate problems in walking about (3), I have severe problems in walking about (4), I am unable to walk about (5). User sees: words only.
EQV2	**SELF-CARE**	I have no problems washing or dressing myself (1), I have slight problems washing or dressing myself (2), I have moderate problems washing or dressing myself (3), I have severe problems washing or dressing myself (4), I am unable to wash or dress myself (5). User sees: words only.
EQV3	**USUAL ACTIVITIES** (e.g. work, study, housework, family or leisure activities)	5-item closed choice: I have no problems doing my usual activities (1), I have slight problems doing my usual activities (2), I have moderate problems doing my usual activities (3), I have severe problems doing my usual activities (4), I am unable to do my usual activities (5). User sees: words only.
EQV4	**PAIN / DISCOMFORT**	5-item closed choice: I have no pain or discomfort (1), I have slight pain or discomfort (2), I have moderate pain or discomfort (3), I have severe pain or discomfort (4), I have extreme pain or discomfort (5). User sees: words only.
EQV5	**ANXIETY / DEPRESSION**	5-item closed choice: I am not anxious or depressed(1), I am

		slightly anxious or depressed (2), I am moderately anxious or depressed (3), I am severely anxious or depressed (4), I am extremely anxious or depressed (5). User sees: words only.
EQV6	We would like to know how good or bad your health is **today.**<br>100 means the **best** health you can imagine.<br>0 means the **worst** health you can imagine.<br>Mark the scale to indicate how your health is **today**.	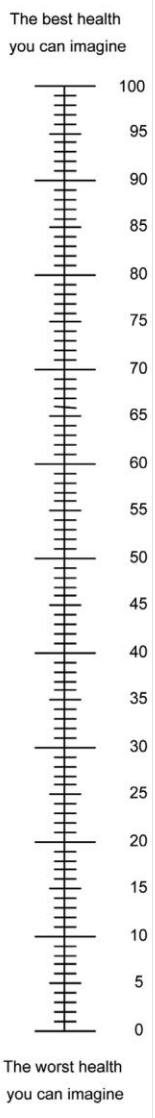<br>Vertical visual analogue scale; integral range from: The worst health you can imagine (0) –The best health you can imagine (100) inclusive. User sees: vertical visual representation of scale with text anchors and mark indicating their selection.

Recovering Quality of Life (ReQoL)

☒ Screening	☐ Baseline	☐ EMA	☐ Mid	☐ Intervention	☐ Post	☒ Follow-up
						11 questions

Source: The University of Sheffield (Via MUCHE)

Id	Question	Presentation
For each of the following statements, please tick one box that best describes your thoughts, feelings and activities over the last week.		
ReQoL1	I found it difficult to get started with everyday tasks	5-item closed choice; None of the time, Only occasionally, Sometimes, Often, and Most or all of the time. User sees: words only.
ReQoL2	I felt able to trust others	
ReQoL3	I felt unable to cope	
ReQoL4	I could do the things I wanted to do	
ReQoL5	I felt happy	
ReQoL6	I thought my life was not worth living	
ReQoL7	I enjoyed what I did	
ReQoL8	I felt hopeful about my future	
ReQoL9	I felt lonely	
ReQoL10	I felt confident in myself	

ReQoL11	Please describe your **physical health** (problems with pain, mobility, difficulties caring for yourself or feeling physically unwell) **over the last week**	5-item closed choice: No problems (4), Slight problems (3), Moderate problems (2), Severe problems (1), Very severe problems (0). User sees: words only.

Scoring:

- For ReQoL1, ReQoL3, ReQoL6, and ReQoL9, 'None of the time' scores 4, 'Only occasionally' scores 3, 'Sometimes' scores 2, 'Often' scores 1, and 'Most or all of the time' scores 0.
- For ReQoL2, ReQoL4, ReQoL5, ReQoL7, ReQoL8, and ReQoL10, 'None of the time' scores 0, 'Only occasionally' scores 1, 'Sometimes' scores 2, 'Often' scores 3, and 'Most or all of the time' scores 4.
- A total score is derived from summing all items.

Subjective Socioeconomic Status (SES)

☒ Screening	☐ Baseline	☐ EMA	☐ Mid	☐ Intervention	☐ Post	☐ Follow-up
						1 question

Source: [59]

Remarks: Text can be refined, layout can be amended as needed for mobile presentation/touch interface. Ladder will need to be re-rendered for mobile.

Id	Question	Presentation
SES	Think of this ladder as representing where people stand in Australia.<br><br>At the top are the people who are the best off – they have the most money, the most education and the most respected jobs.<br><br>At the bottom are the people who are the worst off – who have the least money, least education and the least respected jobs.<br><br>**Where would you place yourself on this ladder?**	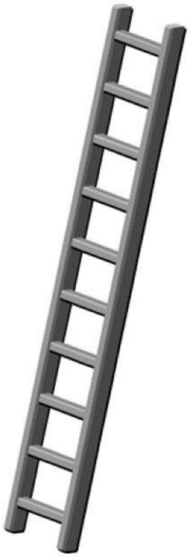<br>Visual representation of 10-rung ladder where any 1 rung can be selected. Scoring: 1-10 inclusive. User sees: ladder with mark indicating their selection.

Abridged NIDA-Modified ASSIST Drug Screening Tool (AOD)

☒ Screening	☐ Baseline	☐ EMA	☐ Mid	☐ Intervention	☐ Post	☐ Follow-up
						4 questions

Source: [60] including only question 1.

Id	Question	Presentation
In the **past year**, how often have you used the following?		

If participant response to question DEM2a is 1 (Male):

AOD1	Five or more alcoholic drinks in a day.	5-level Likert; Never (0) – Once or twice (1) – Monthly (2) – Weekly (3) – Daily or almost daily (4). User sees: words only.

Otherwise:

AOD1	Four or more alcoholic drinks in a day.	As above.

For all participants:

AOD2	Tobacco products.	As above.
AOD3	Prescription drugs for non-medical reasons.	As above.
AOD4	Illegal drugs.	As above.

Scoring: Report individual items only.

Short Warwick Edinburgh Mental Wellbeing Scale (WBS)

☒ Screening	☐ Baseline	☐ EMA	☐ Mid	☐ Intervention	☐ Post	☐ Follow-up
						7 questions

Source: [61]

Id	Question	Presentation
Below are some statements about feelings and thoughts. Please choose the option that best describes your experience of each over the **past two weeks**.		
WBS1	I've been feeling optimistic about the future.	5-item Likert; None of the time (1) – Rarely (2) – Some of the time (3) – Often (4) – All of the time (5). User sees: words only.
WBS2	I've been feeling useful.	
WBS3	I've been feeling relaxed.	
WBS4	I've been dealing with problems well.	
WBS5	I've been thinking clearly.	
WBS6	I've been feeling close to other people.	
WBS7	I've been able to make up my own mind about things.	

Scoring:

- Total score: Sum of item scores, transformed using the lookup table from [61] below into a metric score.

Raw score	Transformed score
7	7.00
8	9.51
9	11.25
10	12.40
11	13.33
12	14.08
13	14.75
14	15.32
15	15.84
16	16.36
17	16.88
18	17.43
19	17.98
20	18.59
21	19.25

Raw score	Transformed score
22	19.98
23	20.73
24	21.54
25	22.35
26	23.21
27	24.11
28	25.03
29	26.02
30	27.03
31	28.13
32	29.31
33	30.70
34	32.55
35	35.00

- Possible range (both raw and transformed): 7 – 35 inclusive.
- Interpretation: higher scores indicate higher positive mental wellbeing. No standardised cut-offs.

Multidimensional Scale of Perceived Social Support (PSS)

☒ Screening	☐ Baseline	☐ EMA	☐ Mid	☐ Intervention	☐ Post	☐ Follow-up
						12 questions

Source: [62]

Id	Question	Presentation
We are interested in how you feel about the following statements. Read each statement carefully. Indicate how you feel about each statement.		
PSS1	There is a special person who is around when I am in need.	7-level Likert; Very strongly disagree (1) – Strongly disagree (2) – Mildly disagree (3) – Neutral (4) – Mildly agree (5) – Strongly agree (6) – Very strongly agree (7). User sees: words only.
PSS2	There is a special person with whom I can share joys and sorrows.	
PSS3	My family really tries to help me.	
PSS4	I get the emotional help and support I need from my family.	
PSS5	I have a special person who is a real source of comfort to me.	
PSS6	My friends really try to help me.	
PSS7	I can count on my friends when things go wrong.	
PSS8	I can talk about my problems with my family.	
PSS9	I have friends with whom I can share my joys and sorrows.	
PSS10	There is a special person in my life who cares about my feelings.	
PSS11	My family is willing to help me make decisions.	
PSS12	I can talk about my problems with my friends.	

Scoring:

- Total score: sum of item scores, divided by 12.
- Significant other subscale score: sum of item scores for PSS1, 2, 5 and 10, divided by 4.
- Family subscale score: sum of item scores for PSS3, 4, 8 and 11, divided by 4.
- Friends subscale score: sum of item scores for PSS6, 7, 9 and 12, divided by 4.
- Possible range (total or subscale): 1 – 7 inclusive.
- Interpretation: higher total/subscale scores indicated greater levels of perceived support. No standardised cut-offs.

Depression, Anxiety and Stress Scale, 21-item version (DASS)

☐ Screening	☒ Baseline	☐ EMA	☒ Mid	☐ Intervention	☒ Post	☒ Follow-up
						21 questions

Source: [38] adapted for presentation on a mobile device (no separate key).

Remarks: Questions must be presented in order shown. The introductory prompt could be shortened if necessary.

<table>
<tr><th>Id</th><th>Question</th><th>Presentation</th></tr>
<tr><td colspan="3">Please read each statement and choose the option which indicates how much the statement applied to you over the past week. There are no right or wrong answers. Do not spend too much time on any one question.</td></tr>
<tr><td>DASS1</td><td>I found it hard to wind down.</td><td rowspan="21">4 item rating scale; Did not apply to me at all (0), Applied to me some degree, or some of the time (1), Applied to me a considerable degree, or a good part of the time (2) Applied to me very much, or most of the time (3), User sees: words only.</td></tr>
<tr><td>DASS2</td><td>I was aware of dryness of my mouth.</td></tr>
<tr><td>DASS3</td><td>I couldn't seem to experience any positive feeling at all.</td></tr>
<tr><td>DASS4</td><td>I experienced breathing difficulty (e.g., excessively rapid breathing, breathlessness in the absence of physical exertion.)</td></tr>
<tr><td>DASS5</td><td>I found it difficult to work up the initiative to do things.</td></tr>
<tr><td>DASS6</td><td>I tended to over-react to situations.</td></tr>
<tr><td>DASS7</td><td>I experienced trembling (e.g., in the hands.)</td></tr>
<tr><td>DASS8</td><td>I felt that I was using a lot of nervous energy.</td></tr>
<tr><td>DASS9</td><td>I was worried about situations in which I might panic and make a fool of myself.</td></tr>
<tr><td>DASS10</td><td>I felt that I had nothing to look forward to.</td></tr>
<tr><td>DASS11</td><td>I found myself getting agitated.</td></tr>
<tr><td>DASS12</td><td>I found it difficult to relax.</td></tr>
<tr><td>DASS13</td><td>I felt downhearted and blue.</td></tr>
<tr><td>DASS14</td><td>I was intolerant of anything that kept me from getting on with what I was doing.</td></tr>
<tr><td>DASS15</td><td>I felt I was close to panic.</td></tr>
<tr><td>DASS16</td><td>I was unable to become enthusiastic about anything.</td></tr>
<tr><td>DASS17</td><td>I felt I wasn't worth much as a person.</td></tr>
<tr><td>DASS18</td><td>I felt that I was rather touchy.</td></tr>
<tr><td>DASS19</td><td>I was aware of the action of my heart in the absence of physical exertion (e.g., sense of heart rate increase, heart missing a beat.)</td></tr>
<tr><td>DASS20</td><td>I felt scared without any good reason.</td></tr>
<tr><td>DASS21</td><td>I felt that life was meaningless.</td></tr>
</table>

Scoring:

- Total score: sum of item scores; multiplied by two.
- Depression subscale: sum of item scores for DASS 3, 5, 10, 13, 16, 17, 21; multiplied by two.
- Anxiety subscale: sum of item scores for DASS 2, 4, 7, 9, 15, 19, 20; multiplied by two.
- Stress subscale: sum of item scores for DASS 1, 6, 8, 11, 12, 14, 18; multiplied by two.
- Possible range (total): 0 – 126 inclusive.
- Possible range (subscale): 0 – 42 inclusive.

- Interpretation: per [39]:

Severity	Depression	Anxiety	Stress	Z score
Normal	0 - 9	0 - 7	0 - 14	< 0.5
Mild	10 - 13	8 - 9	15 - 18	0.5-1.0
Moderate	14 - 20	10 - 14	19 - 25	1.0 – 2.0
Severe	21 - 27	15 - 19	26 - 33	2.0 – 3.0
Extremely Severe	28 +	20 +	34 +	> 3.0

- Normalisation of the total score: per [39], calculate the arithmetic mean of the Z-scores for each of the three DASS subscales (depression, anxiety, stress). The population reference values are (for 20-29 year olds):
    - Depression subscale: mean = 6.35, SD = 6.85
    - Anxiety subscale: mean = 4.77, SD = 4.79
    - Stress subscale: mean = 11.19, SD = 8.25
- Discretisation of the total score will be simplified to the following bounds for Vibe Up:
    - None or mild: < 1.0
    - Moderate: 1.0 ≤ x < 2.0
    - Severe or extremely severe: ≥ 2.0

Modified Physical Activity Vital Sign (PAVS)

☐ Screening	☒ Baseline	☐ EMA	☒ Mid	☐ Intervention	☒ Post	☒ Follow-up
						Up to 2 questions

Source: [45] modified to consider only the most recent week.

Id	Question	Presentation
PAVS1	In the past **week**, on how many **days** did you engage in moderate to strenuous exercise?<br><br>Moderate exercise means that you are breathing hard enough that, while you might be able to talk, singing would not be possible.<br><br>Strenuous exercise means being out of breath enough that you can't talk.	Numeric fill-in/pick 'X day(s)'. Validation: X is integral in the range 0 ≤ X ≤ 7.

If response to PAVS1 > 0:

PAVS2	On average, how many **minutes** did each session of moderate to strenuous exercise last for?	Numeric fill-in/pick 'X minute(s)'. Validation: X is integral in the range 1 ≤ X ≤ 240.

Abridged Pittsburgh Sleep Quality Index (PSQI)

☐ Screening	☒ Baseline	☐ EMA	☒ Mid	☐ Intervention	☒ Post	☒ Follow-up
						1 question

Source: [46] including only question 6.

Id	Question	Presentation
PSQI6	During the past **week**, how would you rate your sleep quality overall?	4-level Likert; Very bad (0), Fairly bad (1), Fairly good (2), Very good (3), User sees: words only.

Mindfulness single item questionnaire (MIND)

☐ Screening	☒ Baseline	☐ EMA	☒ Mid	☐ Intervention	☒ Post	☒ Follow-up
						1 question

Source: Bespoke question.

Id	Question	Presentation
MIND1	Mindfulness is a practice where you intentionally focus your attention on what you're experiencing in the present moment, with an attitude of openness and non-judgment.<br><br>During the past **week**, how mindful have you been?	5-level Likert; Not at all mindful (0) – (1) – (2) – (3) – Extremely mindful (4). User sees: words and numbers.

Modified Positive and Negative Affect Schedule, Short Form (PANAS)

☐ Screening	☐ Baseline	☒ EMA	☐ Mid	☐ Intervention	☐ Post	☐ Follow-up
						12 questions

Source: [51] modified to consider current mood state and with the addition of 2 items from the original PANAS focussed on distress, specifically (‘hopeless’ and ‘calm’).

Id	Question	Presentation
You will be asked a few questions about your current feelings and behaviours. The survey will take 1-2 minutes. Please complete the questions as soon as possible. This helps us to get more accurate data to understand how young people like you have been experiencing in daily life. Incomplete surveys will expire in one hour.		
PANAS0	How do you feel **right now**? (Please select all that apply)	Multiple choice with options: Upset (1), Hostile (2), Alert (3), Ashamed (4), Inspired (5), Nervous (6), Determined (7), Attentive (8), Afraid (9), Active (10), Hopeless (11), Calm (12). User sees: words only.

If response to PANAS0 includes 1 (Upset):

PANAS1	To what extent do you feel **upset** right now?	5-level Likert; Very slightly or not at all (1) – A little (2) – Moderately (3) – Quite a bit (4) – Extremely (5). User sees: words only.

If response to PANAS0 includes 2 (Hostile):

PANAS2	To what extent do you feel **hostile** right now?	As above.

If response to PANAS0 includes 3 (Alert):

PANAS3	To what extent do you feel **alert** right now?	As above.

If response to PANAS0 includes 4 (Ashamed):

PANAS4	To what extent do you feel **ashamed** right now?	As above.

If response to PANAS0 includes 5 (Inspired):

PANAS5	To what extent do you feel **inspired** right now?	As above.

If response to PANAS0 includes 6 (Nervous):

PANAS6	To what extent do you feel **nervous** right now?	As above.

If response to PANAS0 includes 7 (Determined):

PANAS7	To what extent do you feel **determined** right now?	As above.

If response to PANAS0 includes 8 (Attentive):

PANAS8	To what extent do you feel **attentive** right now?	As above.

If response to PANAS0 includes 9 (Afraid):

PANAS9	To what extent do you feel **afraid** right now?	As above.

If response to PANAS0 includes 10 (Active):

PANAS10	To what extent do you feel **active** right now?	As above.

If response to PANAS0 includes 11 (Hopeless):

PANAS11	To what extent do you feel **hopeless** right now?	As above.

If response to PANAS0 includes 12 (Calm):

PANAS12	To what extent do you feel **calm** right now?	As above.

Scoring:

- Positive affect subscale: sum of item scores for PANAS3, 5, 7, 8, 10.
- Negative affect subscale: sum of item scores for PANAS1, 2, 4, 6, 9.
- If item not shown, assume score is 1 (Very slightly or not at all).
- Possible score range (both subscales): 5 – 25 inclusive.
- Interpretation: higher scores indicate higher levels of positive/negative affect, respectively.
- (There is no total score. Items PANAS11 and PANAS12 are not used for scoring.)

Behavioural intentions (BHV)

☐ Screening	☐ Baseline	☒ EMA	☐ Mid	☐ Intervention	☐ Post	☐ Follow-up
						Up to 2 questions

Source: Bespoke questions

If at least one item selected in response to PANAS0:

Id	Question	Presentation
BHV1	How likely are you to do something specifically as a result of how you feel **right now**?	4-level Likert; Highly unlikely (0) – Unlikely (1) – Likely (2) – Highly likely (3). User sees: words only.

If response to BHV1 ≥ 2 (Likely or Highly likely):

BHV2	What is it that are you likely to do?<br><br>We don't actively monitor responses to this question, but help is always available if you need it. [Link to support options]	Free text, Max 512 characters. Validation: none.

Scoring: Report individual items only.

Abridged Credibility and Expectancy Questionnaire (CEQ)

☐ Screening	☐ Baseline	☐ EMA	☒ Mid	☐ Intervention	☐ Post	☐ Follow-up
						2 questions

Source: [63] including only questions 1 (Set 1, question 1) and 6 (Set 2, question 2), selected as single items with highest distinct loadings on credibility/expectancy factors.

Id	Question	Presentation	Purpose
In the next two questions we would like you to indicate how much you believe, right now, that this self-help app will help improve your feelings of distress.			
CEQ1	Right now, how logical does the idea of using this app to improve your distress seem to you?	9-level Likert; Not at all logical (1) – (2) – (3) – (4) – Somewhat logical (5) – (6) – (7) – (8) – Very logical (9). User sees: words and numbers.	Credibility factor
CEQ6	Close your eyes for a few moments and try to identify what you really **feel** about this app and its likely success.<br>By the end of the study, how much improvement in your distress do you really **feel** will occur?	11-level numeric rating scale: 0% to 100% inclusive in 10% increments. User sees: percentages.	Expectancy factor

Scoring: Report individual items only.

Abridged Revised University of Rhode Island Change Assessment Scale (URC)

☐ Screening	☐ Baseline	☐ EMA	☒ Mid	☐ Intervention	☐ Post	☐ Follow-up
						6 questions

Source: [64] abridged to select the two questions accounting for the highest proportion of variance explained for each of the ambivalence, seeking assistance and action factors (3 × 2).

Id	Question	Presentation	Purpose
Each statement describes what a person might think when starting a new self-help programme.<br>Please indicate the extent to which you agree or disagree with each statement in terms of what you think **right now**.			
URC19	I wish I had more ideas on how to solve my problems.	5-level Likert; Strongly disagree (1) – Disagree (2) – Undecided (3) – Agree (4) – Strongly agree (5). User sees: words only.	Seeking assistance factor
URC24	I hope that this app will contain some good advice for me.		Seeking assistance factor
URC25	Anyone can talk about changing; I'm actually doing something about it.		Action factor
URC26	All this talk about psychology is boring. Why can't people just forget their problems?		Ambivalence towards change factor
URC29	I have worries but so does the next person. Why spend time thinking about them?		Ambivalence towards change factor
URC30	I am actively working on my problem.		Action factor

Scoring:

- Total: Sum item scores of URC19, 24, 25 and 30 and **reversed** scores of URC26 and 29.
- Seeking assistance subscale: Sum item scores of URC19 and 24.
- Action subscale: Sum item scores of URC25 and 30.
- Ambivalence towards change subscale: sum **reversed** item scores of URC26 and 29.
- Possible total score range: 6 – 30 inclusive, subscales: 2 – 10 inclusive.
- Interpretation: higher total scores indicate greater change readiness.

Daily log of engagement with intervention (LOG)

☐ Screening	☐ Baseline	☐ EMA	☐ Mid	☒ Intervention	☐ Post	☐ Follow-up
						1 question

Source: Bespoke questions.

Remarks: Participants will be encouraged to log their engagement with the intervention once per day during the intervention period. Participants will only see the option corresponding to the intervention they are allocated to (LOGa for Mindfulness, LOGb for Physical Activity, LOGc for Sleep Hygiene); the active control (EMA) condition will not see any question. Completion of the question is not compulsory.

Id	Question	Presentation
LOGa	How much time did you spend practicing mindfulness yesterday?	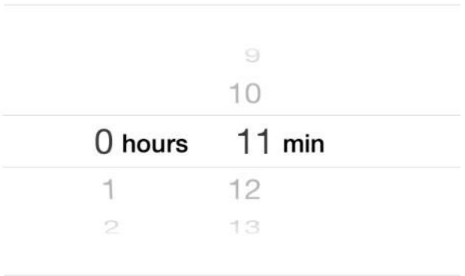<br>Timespan in hours and minutes.<br>Hours: 0 – 23 (coded 0 – 23).<br>Mins: 0 – 59 (coded 0 – 59)<br>Default: 0 hours and 0 minutes.
LOGb	How much time did you spend being physically active yesterday?<br><br>Please include any changes you made to increase your physical activity throughout the day (not just exercise workouts).	
LOGc	How much sleep did you get last night?	

Scoring: Report individual items only.

Within-study exposures questionnaire (EXP)

☐ Screening	☐ Baseline	☐ EMA	☐ Mid	☐ Intervention	☒ Post	☐ Follow-up
						4 questions

Source: Bespoke questions.

Remarks: Rather than trying to enumerate exposures (which nevertheless may be evaluated differently by different people), the approach adopted here is to elicit *perception of perceived impact* on daily life, mental health or app use of any recent happening, regardless of cause.

Id	Question	Measurement	Purpose
EXP1	In the past **two weeks**, my life or routine was disrupted for some reason (ignoring anything to do with this app.)	5-level Likert, Strongly disagree (1) – Disagree (2) – Undecided (3) – Agree (4) – Strongly agree (5), User sees: words only.	Life event confound
EXP2a	In the past **two weeks**, something **negatively** affected my mental health (ignoring anything to do with this app.)		Mental health change confound
EXP2b	In the past **two weeks**, something **positively** affected my mental health (ignoring anything to do with this app.)		Mental health change confound
EXP3	In the past **two weeks**, something interfered with my ability to use this app.		(Attitudinal) non-engagement confound

Scoring: Report individual items only.

UX questionnaire (UX)

☐ Screening	☐ Baseline	☐ EMA	☐ Mid	☐ Intervention	☒ Post	☐ Follow-up
						Up to 9 questions

Source: Bespoke questions, based on the System Usability Scale[65] and mHealth App Usability Questionnaire[66].

Id	Question	Measurement	Purpose
UX1	I found the app easy to use.	5-level Likert, Strongly disagree (1) – Disagree (2) – Undecided (3) – Agree (4) – Strongly agree (5), User sees: words only.	Usability: ease of use construct
UX2	I found the app useful for my mental health.		Usability: usefulness construct
UX3	Overall, I am satisfied with the app.		Usability: satisfaction construct
UX4a	I had no problems using the app.		Technology barriers

If response to UX4A is 'Strongly Disagree' or 'Disagree'…

UX4b	Can you tell us what problem(s) you encountered?<br><br>Letting us know will help us improve the experience for others. Thank you!	Free text, Max 256 characters.<br>Validation: non-null.	Technology barriers

For participants in an intervention arm (mindfulness, physical exercise, sleep hygiene) only…

UX5	I trusted the information given by the app.	5-level Likert, Strongly disagree (1) – Disagree (2) – Undecided (3) – Agree (4) – Strongly agree (5), User sees: words only.	Post-hoc credibility
UX6	Activities or actions suggested by the app **were new** for me.		Behaviour-change confound
UX7	**In the past two weeks**, I have put into practice activities or actions suggested by the app.		Subjective compliance
UX8	I **intend in the future** to put into practice activities or actions suggested by the app.		Post-hoc intention

Scoring: Report individual items only.

Appendix 9 Study Reminders

Vibe Up Study Reminders

Version dated: 15 Mar 2021

1. Purpose

The purpose of this document is to provide the templates that will be used for sending notifications/reminders to participants in the Vibe Up study.

2. SMS reminders

Example 1: Alerting participants that their mini-trial is about to start.

> Get ready to start! Vibe Up begins tomorrow 🥳

Example 2: Inviting participants to start their mini-trial.

> Vibe Up starts today! Download the app now 📲 [URL LINK]
> This invitation expires at midnight on Thursday - see you there!

Example 3: Reminding participants to complete baseline questionnaires.

> 📢 Answer the kick-off questions before midnight tomorrow to stay in the study ⏱️👩‍🎓

Example 4: Reminding participants to complete post questionnaires.

> 📢 Answer the final survey to finish the study and get your gift card 🎁
> The survey expires at midnight tomorrow!

3. Email reminders

Example 1: Inviting participants to install study app.

> **Welcome to the Vibe Up Study – Get Started Today**
>
> Hi [first name],
>
> Thanks for joining Vibe Up - a ground-breaking study using AI to test new ways to improve mental health in uni students.
>
> Vibe Up starts today!
>
> **Ready to start? Download the study app now:**

1986

LINK BUTTON

The app will get you started in just a few minutes.

This link expires at midnight on Thursday, so download the app today, or you might miss out!

Any questions or tech issues? Contact vibeup@blackdog.org.au

Best wishes,

The Vibe Up team.

Black Dog Institute
www.blackdog.org.au
vibeup@blackdog.org.au

Example 2: reminding participants to complete post questionnaires

Final survey for Vibe Up is due now!

Hi [first name],

Thanks for being part of the Vibe Up study over the past few weeks.

Heads up that you have **one survey left to finish the study**. Jump into the Vibe Up app to do it now – it should only take about 5 minutes.

The survey will expire at midnight tonight.

If you finish the study, we'll send you a **personalized report** on your wellbeing based on the info you have provided throughout, with tailored suggestions for staying well. You'll also receive a **$30 gift card** to say thanks for your contribution.

Thanks again for your contribution so far,

The Vibe Up team.

Black Dog Institute
www.blackdog.org.au
vibeup@blackdog.org.au

4. App-generated notifications

Example 1: Prompting participants to complete baseline questionnaires.

Get started by answering your kick-off questions

Page 2 of 3
HC Number: HC200466 **Version dated:** 15 Mar 2021

1987

Example 2: Prompting participants to complete post questionnaires.

Almost there! Do your final survey to tell us how you're going 📋

Example 3: Prompting participants to start assigned treatment. Participants will be sent one of the following notifications, depending on the treatment they are assigned to.

Get started with a 3 min 📺 on the what, why and how of mindfulness
Get started with the what, why and how of physical activity! 🤓
Get started with the benefits of boosting your sleep 🤓

Version history

Date	Summary of changes
01 Dec 2020	Document created.
19 Jan 2021	Document populated with example templates of app generated notifications, and SMS and email reminders.
20 Jan 2021	'Let's start' email template example created.
21 Jan 2021	Addition of pre-study activation SMS. Minor edits.
15 Mar 2021	Updated copy for invitation emails.

Appendix 10 Vibe Up Intervention Specifications

Vibe Up Study Interventions Specifications

Version dated: 15 Jan 2021

1. Purpose

To purpose of this document is to summarise the structure and content of the three interventions in the Vibe Up study:

- Vibe Up Mindfulness
- Vibe Up Physical Activity
- Vibe Up Sleep Hygiene

2. Structure of the interventions

All three interventions are self-guided and delivered via the Vibe Up app installed on participants' smartphones. The interventions are designed (assuming compliance) to be loosely matched on dose and required effort over a 14-day period.

Each intervention consists of a combination of:

- Brief modular information covering key concepts, delivered e.g. as infographics.
- Structured activities, e.g. practising mindfulness with guided audio.
- 'Frequently asked questions' section including tips, safety advice and answers to common questions associated with that intervention.

Each intervention also indicates the expected effort (e.g. how many practice activities to undertake each week).

3. Vibe Up Mindfulness

The Vibe Up Mindfulness intervention starts with an introductory video (3 minutes), followed by five guided mindfulness practices delivered as audio recordings (3-5 minutes in length).

Module	Summary of content	Format
Introduction	• Explanation of mindfulness and its benefits for students.<br>• Formal vs informal mindfulness practice.<br>• Tuning into the five senses to bring mindful awareness to daily activities.<br>• Reducing judgment and increasing self-compassion.	Video
1 - Mindful breathing	Guided mindfulness practice based on mindful awareness of breathing.	Audio, with male/female voice options
2 - Unhooking from thoughts	Guided mindfulness practice teaching non-judgement towards thoughts, using 'leaves on a stream' imagery.	Audio, with male/female voice options
3 - Body scan	Guided mindfulness practice based on mindful awareness of body sensations and releasing of muscle tension.	Audio, with male/female voice options

4 - Mindful eating	Guided mindfulness practice encouraging the use of all five senses to bring curiosity to everyday activities (eating).	Audio, with male/female voice options
5 - Mindful walking	Guided mindfulness practice blending awareness of surrounding environment and bodily sensations.	Audio, with male/female voice options

The modules are made available to participants as follows:

- The introductory video is available to participants immediately after allocation to the intervention.
- The first mindfulness audio guide becomes available immediately after the introductory video has been completed.
- The subsequent mindfulness audio guides are sequentially released, at one day intervals (regardless of participant engagement).

Expected effort: minimum one mindfulness audio completed daily at participant's convenience. All modules completed at least once by end of treatment period. Participants are also encouraged to practice bringing mindful awareness to activities throughout their day ('informal' mindfulness).

4. Vibe Up Physical Activity

The Vibe Up Physical Activity intervention starts with an introductory infographic, then prompts participants each day to choose a goal to increase their physical activity that day. An evidence-based seven-minute high-intensity circuit training (HICT) protocol is provided as one option for increasing physical activity.

Module	Summary of content	Format
Introduction	• Benefits of physical activity for cognition, mental health and physical health.<br>• Australian guidelines for physical activity.<br>• Setting realistic goals and benefits of even small change.<br>• Tips to increase physical activity:<br>○ Choosing enjoyable activities<br>○ Setting goals and tracking progress<br>○ Being social<br>○ Increasing step count	Infographic (reading time: <5 minutes)
Daily goal setting	Participants choose from the following options:<br>• Increasing step count<br>• 7-minute HICT workout<br>• Other activity (social sport, gardening, yoga, bike riding, dancing, etc)<br>• Rest day	Interactive app cards, with informational text tailored to the option selected.
HICT workout	HICT protocol consisting of the following 12 exercises (each performed for 30 seconds, with 10 second interim rest periods):<br>1. Jumping jacks<br>2. Wall sit<br>3. Push up<br>4. Abdominal crunch<br>5. Step up onto a chair<br>6. Squat<br>7. Tricep dip on chair	Video (7 minutes)

Page 2 of 4
HC Number: HC200466 **Version dated:** 15 Jan 2021

	8. Plank<br>9. High knees<br>10. Lunge<br>11. Pushup and rotation<br>12. Side plank	

The modules are made available to participants as follows:

- The introductory infographic is available to participants immediately after allocation to the intervention.
- The Daily goal setting and 7-minute workout becomes available immediately after a participant has completed the introductory infographic.

Expected effort: physical activity completed daily at participant's convenience, including 7-minute work out three times weekly or, alternatively, increasing steps by 10% weekly

5. Vibe Up Sleep Hygiene

The Vibe Up Sleep Hygiene intervention centres around four brief, sequential modules covering key sleep hygiene concepts, which are delivered via infographics. Each module takes up to five minutes to read.

Module	Summary of content	Format
1 – Why sleep?	• Recommended hours of sleep per night.<br>• Impact of lack of sleep on cognition and emotion.<br>• Benefit of sleep for mental health and physical health.<br>• Introduction to sleep hygiene.	Infographic
2 – Sleep habits	• Establishing a regular bedtime and wake time.<br>• Eliminating (or limiting) naps.<br>• Establishing a wind down routine.	Infographic
3 – Sleep environment	• Reducing light, noise and temperature disturbance, and ensuring bedding is comfortable.<br>• Limiting use of bed to sleep and sex.<br>• The impact of electronic devices on sleep; avoiding screens/blue light prior to bedtime, and not sleeping with a phone.<br>• Get out of bed when unable to sleep for 20 minutes or more, do something relaxing and return to bed when sleepy.	Infographic
4 – Daily activities for sleep	• Reducing caffeine, alcohol, and nicotine intake; especially close to bedtime.<br>• Eating a healthy diet and considering timing of food/fluid intake.<br>• Increasing physical activity and avoiding exercise close to bedtime.	Infographic

The modules are made available to participants as follows:

- Module 1 is available to participants immediately after allocation to the intervention.
- Module 2 becomes available immediately after a participant has completed Module 1.
- Module 3 becomes available two days after Module 2 is made available (regardless of participant engagement).

- Module 4 becomes available two days after Module 3 is made available (regardless of participant engagement).

Expected effort: All modules completed at least once by end of the treatment period. Participants are instructed to chose one strategy from each of module 2 – 4 and implement it daily (total of 3 strategies by end of treatment period).

Version history

Date	Summary of changes
01 Dec 2020	Document created.
15 Jan 2020	Details of structure and content of three interventions added.

Appendix 11 Significant Safety Issue Notification Form

Vibe Up Study Significant Safety Issue Notification Form

Version dated: 01 Dec 2020

Refer to the Trial Protocol, **Section 7.6** for details on how safety issues must be managed and reported.

Applicable definitions:

- A *significant safety issue* is any issue that could adversely affect the safety of participants or materially impact on the continued ethical acceptability or conduct of the trial.
- An *urgent safety measure* is any action or procedure required to be taken to eliminate an immediate hazard to a participant's health or safety.

**Protocol Id**	HC200466_CTP	**Applicable date version**	Enter date version of protocol applicable at time of issue.
**HREC number**	HC200466		
**Site**	Black Dog Institute (Single site trial)		
**Principal Investigator**	Prof Helen Christensen, Director and Chief Scientist, Black Dog Institute<br>+61 2 9382 3717<br>h.christensen@blackdog.org.au		
**Date of report**	Enter the report date.		
**Completed by**	Enter your name.<br>Enter your telephone number.<br>Enter your email address.		
**Issue title**	Enter a brief descriptive title for the Significant Safety Issue.		
**Issue date**	Enter the date on which the issue was identified.		
**Descriptive summary**	Enter a summary of the issue.		
**Action(s) taken**<br>Pick all that apply.	☐Implementation of an Urgent Safety Measure. Complete **Section 1**.<br>☐Application to amend approved protocol. Complete **Section 2**.<br>☐Temporary halt of the trial for safety reasons. Complete **Section 3**.<br>☐Early termination of the trial for safety reasons. Complete **Section 4**.		

1. Implementation of an Urgent Safety Measure

**Description of measure**	Enter a description of the Urgent Safety Measure.
**Introduction date**	Enter the actual/planned date on which the measure will be implemented.
**Provide a justification for the measure and how it will address the identified safety issue. If the introduction is to be delayed, please explain why**	
Enter text.	

2. Application to amend approved protocol

**Is the amendment being submitted with this report?**	Choose an item.

Page 1 of 3

HC Number: HC200466 **Version dated:** 01 Dec 2020

2000

**If No, describe the nature of the planned amendment(s) (e.g. revised protocol or PISCF) and the likely timeframe for submission of the Notification of an Amendment to the HREC.**
Enter text.

3. Temporary halt of the trial for safety reasons

**Is this a recommendation of the DSMB?**	Choose an item.	If Yes, please attach the written recommendation to this report.
**Describe the scope of the halt - e.g. suspension of recruitment or cessation/interruption of trial treatment/intervention.**		
Enter text.		
**Provide details of the number of participants still receiving treatment in Australia at the time of the temporary halt and their proposed management.**		
Enter text.		

4. Early termination of the trial for safety reasons

**Is this a recommendation of the DSMB?**	Choose an item.	If Yes, please attach the written recommendation to this report.
**Provide details of the number of participants still receiving treatment in Australia at the time of termination and their proposed management.**		
Enter text.		
**Please also comment on the consequences of early termination for the evaluation of the study results and provide the anticipated date when the final progress report will be provided to the HREC, if not provided with this notification.**		
Enter text.		

Principal Investigator declaration

I have reviewed and approved this safety issue notification and any recommendations for action that it contains.

I declare that the information provided above is, to the best of my knowledge, true and accurate.

Name of Researcher (please print)	Helen Christensen
Signature of Researcher	
Date	Date

Page 2 of 3

HC Number: HC200466 **Version dated:** 01 Dec 2020

2001

2002

Template version history

Date	Summary of changes
01 Dec 2020	Template created.

References

1. Family Planning NSW, *Appendix G - Significant Safety Issue Form*. 2019, Family Planning NSW: Sydney, NSW, Australia.

2003

Appendix 12 Unexpected and Serious Adverse Event Notification Form

Vibe Up Study Unexpected and Serious Adverse Event Notification Form

Version dated: 01 Dec 2020

Refer to the Trial Protocol, **Section 7.6** for details on how adverse events must be managed and reported.

Applicable definitions:

- An *adverse event* is any untoward occurrence (medical or other) in a clinical trial participant administered one or more of the trial interventions. It does not necessarily have a causal relationship with this treatment. Further:
- An adverse event is *serious* if it results in death, is life-threatening, requires hospitalisation or prolongation of existing hospitalisation, results in persistent or significant disability or incapacity.
- An adverse event is *expected* if it defined as a known or potential risk (see **Table 2.3** in the Trial Protocol). All other adverse events are *unexpected*.

**Protocol Id**	HC200466_CTP	**Applicable date version**	Enter date version of protocol applicable at time of event.
**HREC number**	HC200466		
**Site**	Black Dog Institute (Single site trial)		
**Principal Investigator**	Prof Helen Christensen, Director and Chief Scientist, Black Dog Institute<br>+61 2 9382 3717<br>h.christensen@blackdog.org.au		
**Report type**	Choose an item.		
**Date of report**	Enter the report date.		
**Completed by**	Enter your name.<br>Enter your telephone number.<br>Enter your email address.		
**Participant Id**	Enter the affected participant identifier.		
**Sex**	Choose an item.	**Age at entry**	Enter age at study entry in years.
**Event title**	Enter a brief descriptive title for the event.		
**Event type**	Choose an item.		
**Event intensity**	Choose an item.		
**SAE category**	If applicable, choose an item. Otherwise choose 'Not applicable'.		
**Event outcome**	Choose the outcome status applicable at time of report.		
**Causal link to study**	Choose an item.		
**Study consequence**	Choose an item.		
**Linked Significant Safety Issue?**	Choose an item.	If Yes, the Significant Safety Issue Notification form must be completed and attached to this form.	
**Last study action date**	Enter the most recent date of any study action/assessment prior to the event.<br>Enter a brief description of the action/assessment, if applicable.		
**Event date**	Enter the event date or event onset date.		

Page 1 of 4

HC Number: HC200466 **Version dated:** 01 Dec 2020

**Study awareness date**	Enter the event date or event onset date.
**Registered date**	Enter the event date or event onset date.
**First report date**	Enter the event date or event onset date.
**Descriptive summary**	Enter a summary of the event, actions taken and outcome.
**Relevant medical history**	Enter any relevant medical history or context, if applicable, including existing treatments/conditions.
**Actions taken**	Enter a summary of actions taken by the study.

Study Qualified Medical Expert declaration

I have reviewed and approved the adverse event description provided above.

I declare that the information provided above is, to the best of my knowledge, true and accurate.

Name of Researcher (please print)	Jill Newby
Signature of Researcher	
Date	Date

Principal Investigator declaration

I have reviewed and approved the adverse event description provided above.

I declare that the information provided above is, to the best of my knowledge, true and accurate.

Name of Researcher (please print)	Helen Christensen
Signature of Researcher	
Date	Date

Sponsor's Independent Medical Expert review and declaration

**Decision**<br>(Pick one only.)	☐ The adverse incident is adequately characterised, remedial actions (if any) are acceptable and no further action is required.<br>☐ Further action is required. Please describe below.
**Required actions**	Describe further <u>required</u> actions, if any.
**Recommended actions**	Describe further <u>recommended</u> actions, if any.

I have reviewed this adverse incident report.

Name of Researcher (please print)	Jennie Hudson

Page 2 of 4

HC Number: HC200466 **Version dated:** 01 Dec 2020

Signature of Researcher	
Date	Date

Template version history

Date	Summary of changes
01 Dec 2020	Template created.

References

1. Morrison, J., *Appendix A - Example SAE Report Form*. 2020, Black Dog Institute: Sydney, NSW, Australia.

Appendix 13 Safety Monitoring Register

Headings for a single register entry are shown.

Vibe Up Study Safety Monitoring Register

Version dated: 01 Dec 2020

Trial site	Participant Id	Date of event	Summary of event details	Date recorded in register	Recorded by	Date notified to Principal Investigator
Select trial site...						

INITIAL ASSESSMENT				
Assessed by	Name	Assessed event type	Unexpected and Serious Adverse Event Notification Form completed?	Significant Safety Issue identified?
Select person responsible for assessing event...		Select event type...	Select...	Select...

SPONSOR'S INDEPENDENT MEDICAL EXPERT				
Name	Date notified	Date review completed	Summary of corrective or follow up steps	Date resolved

Reported to	Date reported
Select the body event was reported to...	

HC Number: HC200466

Page 1 of 1
Version dated: 01 Dec 2020

Appendix 14 Pre-Trial/Trial Initiation Monitoring and Reporting Procedure

Vibe Up Study Pre-Trial and Trial Initiation Monitoring Procedure

Version dated: 22 Oct 2021

1. Purpose

The International Council for Harmonisation (ICH) Good Clinical Practice (GCP)[1] requires that a process of assessment and documentation be completed to confirm that the study site is suitable and ready to run the trial and that the trial procedures have been reviewed with the Principal Investigator and trial staff. (ICH GCP 8.2.19/8.2.20)

This procedure describes the steps that shall be completed before the Vibe Up study commences and the steps required to initiate the study. It also specifies how these steps shall be recorded to produce the pre-trial and trial initiation monitoring reports.

2. Scope

The procedure applies to all staff involved in the preparation and commencement of the Vibe Up study.

Because the Vibe Up study is a decentralised study coordinated via a single site (Black Dog Institute) this procedure will be completed only once.

3. Responsibilities

Responsibility for ensuring the completion of, and appropriate documentation of, the pre-trial and trial initiation monitoring procedures is delegated by the Principal Investigator to the Research Project Manager (WuYi Zheng.)

It is the responsibility of those staff listed in **Table A2** to ensure that they review study procedures when requested by the PI or their delegate to enable timely completion of the Trial Initiation Monitoring Procedure.

It is the responsibility of all study staff to support this process as required to ensure that the study can start as planned.

4. Pre-Trial Monitoring Procedure

The PI or their delegate will arrange completion the **Pre-trial Monitoring Report** (see **Appendix 1**) which will confirm that all the resources required to run the study are established and available.

Confirmation of readiness for each required pre-trial action or procedure should be notified to the PI or their delegate by the relevant accountable person in writing (e.g. by email.)

The Pre-Trial Monitoring Report and any assessments that inform its completion must be completed no earlier than 4 weeks prior to the start of the study. If there is any delay in starting the study, or should the study be abandoned and then restarted, then this procedure must be repeated.

5. Trial Initiation Monitoring Procedure

The PI or their delegate will arrange completion the **Trial Initiation Monitoring Report** (see **Appendix 2**), which will confirm that trial procedures have been reviewed by the PI and all relevant trial staff.

Each staff member named in **Table A2** must send in writing (e.g. by email) to the PI or their delegate confirmation that they have reviewed the relevant procedures.

The Trial-Initiation Monitoring Report must be completed no earlier than 72 hours prior to the start of the study. If there is any delay in starting the study, or should the study be abandoned and then restarted, then this procedure must be repeated.

Version history

Date	Summary of changes
01 Dec 2020	Document created.
22 Oct 2022	Commenced completion of pre-trial reports

References

1. International Council for Harmonisation of Technical Requirements for Pharmaceuticals for Human Use (ICH), *Integrated Addendum to ICH E6(R1): Guideline for Good Clinical Practice*. 2016.

Appendices

Appendix 1 – Vibe Up Study Pre-trial Monitoring Report

Table A1 – Required pre-trial actions and procedures

Required pre-trial action or procedure	Status	Accountable person*	Assessed on
Completion of, and confirmation of fitness for purpose of, all Standard Operating Procedures by study operational team.	Done	WuYi Zheng	22/10/2021
All active, current protocol and procedure versions notified to, and accepted by, UNSW and Deakin HREC.	Done	WuYi Zheng	22/10/2021
Delegations Log complete and up-to-date.	Done	WuYi Zheng	22/10/2021
All required staff training complete per Training Procedure.	Done	WuYi Zheng	22/10/2021
Trial Insurance confirmed as up-to-date and providing cover for the planned trial period.	Done	WuYi Zheng	02/11/2021
DSMB established and Terms of Reference and Procedures agreed by committee.	Done	WuYi Zheng	01/11/2021
Successful testing (per Vibe Up App Testing Plan) and deployment of Vibe Up Study App.	Done	WuYi Zheng	22/10/2021
Vibe Up App and data collection platform version-locked.	Done	Leonard Hoon	01/11/2021
Successful testing of screening (per Questionnaire Testing Plan) and app-based data collection instruments, including psychological safety triggers.	Done	WuYi Zheng	01/11/2021
Trial algorithm version-locked and Algorithm Specification documentation up-to-date.	Done	Sunil Gupta	28/10/2021
Confirmation of readiness to start trial obtained from study operational team (BDI).	Done	WuYi Zheng	01/11/2021
Confirmation of readiness to start trial obtained from study operational team (Deakin).	Done	Leonard Hoon	01/11/2021
Confirmation of readiness to implement Psychological Safety Response Procedure, if needed, from clinical staff members assigned to this procedure.	Done	Jill Newby	22/10/2021
Confirmation of readiness to start trial obtained from algorithm team.	Done	Sunil Gupta	28/10/2021
Confirmation of readiness to start trial and execute Systems Incident Identification and Response Protocol, if required, obtained from software team.	Done	Leonard Hoon	01/11/2021

Principal Investigator declaration

I confirm that the required pre-trial actions and procedures listed in **Table A1**, above, which confirm the suitability of the Black Dog Institute site to run the Vibe Up Study have been adequately completed.

Name of Researcher (please print)	Helen Christensen
Signature of Researcher	Helen Christensen
Date	23/11/2021

Appendix 2 – Vibe Up Study Trial Initiation Monitoring Report

Table A2 – Staff who must review trial procedures prior to study start

Staff member	Confirmation of review received?
Svetha Venkatesh	Yes
Jill Newby	Yes
Kit Huckvale	Yes
Eileen Stech	Yes
WuYi Zheng	Yes
Jin Han	Yes
Andrew Mackinnon	Yes
Sunil Gupta	Yes
Manisha Senadeera	Yes
Truyen Tran	Yes
Kon Mouzakis	Yes
Rajesh Vasa	Yes
Leonard Hoon	Yes
Stuart Cameron	Yes
Rena Logothetis	Yes

Principal Investigator declaration

I confirm that:

- I have reviewed all trial procedures; and that
- All trial procedures have been reviewed by the relevant trial staff listed in **Table A2**, above.

Name of Researcher (please print)	Helen Christensen
Signature of Researcher	
Date	23/11/2021

Appendix 15 Delegations Log

The assignment/revision of delegated responsibilities will not be treated as a notifiable protocol change.

Vibe Up Study Delegations Log

Version dated: 15 Mar 2022

1. Purpose

The purpose of the Delegations Log is to record significant study-related duties delegated to specific staff by the Principal Investigator (PI).

For the purposes of this document, a 'significant' study-related duty is any activity, procedure or process identified by the PI as important to the operation of the Vibe Up study, its ethical conduct, participant safety, and/or the satisfaction of any clinical or data governance requirements, including those laid out by the International Council for Harmonisation (ICH) Good Clinical Practice (GCP) Guideline[1].

2. Scope

It applies to all staff involved in the Vibe Up Study, regardless of their institution.

3. Responsibilities

It is the responsibility of the PI (Helen Christensen) to identify and assign (with the agreement of the assigned party) significant study-related duties.

Responsibility for administration of the Delegations Log is assigned to the Research Project Manager (WuYi Zheng). This responsibility entails: capturing details of duties identified/delegated by the PI; notifying delegated staff; securing authorised signatures; flagging delegated or delegable duties not captured by the Log for review by the PI; and maintaining secure, version-controlled copies of the Log.

It is the responsibility of named staff to promptly review their delegated duty or duties and either accept by signing or flag promptly to the PI any duties that they are unable to undertake or agree to.

Version history

Date	Summary of changes
01 Dec 2020	Document created.
13 Jan 2021	Minor edits to purpose and responsibilities, and edits to delegated duties.
15 Jan 2021	Edits to delegated duties
28 July 2021	Duties added for Dr Joanne Beames, Dr Artur Shvetcov, Ms Jodie Rosenberg & Dr Priya Rani<br>Duties removed for Dr Kit Huckvale<br>Duties updated for Dr Eileen Stech & A/Prof. Jill Newby
12 Oct 2021	Duties updated for Dr Jin Han, Dr Joanne Beames, & Dr Artur Shvetcov<br>Duties removed for Dr Eileen Stech
19 Jan 2022	Duties added for Dr Omar Ibrahim
15 Mar 2022	Duties added for Dr Aimy Slade

References

1. International Council for Harmonisation of Technical Requirements for Pharmaceuticals for Human Use (ICH), *Integrated Addendum to ICH E6(R1): Guideline for Good Clinical Practice*. 2016.
2. National Institute of Dental and Craniofacial Research, *Delegation of Responsibilities Log*. 2010, National Institutes of Health: Bethseda, MD, USA.

Vibe Up Study Delegations Log

Version dated: 15 Mar 2022

Staff to whom the Principal Investigator (PI) has delegated significant study-related duties. Digital signatures are acceptable.

Full name / Position	Responsibilities	Start date	End date	Staff signature and date	PI signature and date
**Dr WuYi Zheng**<br>Research Project Manager<br>Black Dog Institute	• Administration of the Delegations Log<br>• Day-to-day trial management per Clinical Trial Protocol<br>• Participant recruitment<br>• Mini-trial conduct and monitoring<br>• Verification and analysis of screening and trial data<br>• Completion and documentation of Pre-trial and Trial Initiation Monitoring Procedure<br>• Active monitoring of the study email address for participant risk disclosures (must be delegated during absence)<br>• Ensure timely follow-up of all participant risk disclosures per the Psychological Safety Response Procedure.<br>• Maintenance of Safety Monitoring Register and	01/01/2021	31/12/2022		

Full name / Position	Responsibilities	Start date	End date	Staff signature and date	PI signature and date
	ensuring timely completion of Unexpected and Serious Adverse Event and Significant Safety Issue notification Forms<br>• Staff training delivery and record keeping per Training Policy<br>• Mandatory record keeping and reporting per Clinical Trial Protocol and Grant Funding Agreement<br>• Trial data backup per Research Data Management Plan<br>• Setup, validation and day-to-day operation of the participant screening process<br>• Administration of the Optimise Publications Committee<br>• Data steward				
**Associate Professor Jill Newby**<br>Associate Professor<br>Black Dog Institute	• Study Qualified Medical Expert<br>• Oversight of day-to-day trial operations<br>• Chair of Trial Operations Working Group<br>• Oversight of mandatory reporting per Clinical Trial	01/01/2021	31/12/2022		

Full name / Position	Responsibilities	Start date	End date	Staff signature and date	PI signature and date
	Protocol and Grant Funding Agreement<br>• Intervention content and programme design<br>• Provision of psychological support per Psychological Safety Response Procedure<br>• Review and approval of Unexpected and Serious Adverse Event Notification Forms.<br>• Chair of Internal Management Committee<br>• Chair of Knowledge Translation Working Group				
**Dr Jin Han**<br>Research Fellow<br>Black Dog Institute	• EMA study design and data analysis plan<br>• Oversight of EMA operations<br>• DSMB study investigator	01/01/2021	31/12/2022		
**Dr Joanne Beames**<br>Postdoctoral Research Fellow & Clinical Psychologist<br>Black Dog Institute	• Provision of psychological support per Psychological Safety Response Procedure<br>• Assist in the maintenance of the Safety Monitoring Register	30/06/2021	31/12/2022		
**Dr Artur Shvetcov**	• Verification and analysis of screening and trial data	02/08/2021	31/12/2022		

Full name / Position	Responsibilities	Start date	End date	Staff signature and date	PI signature and date
Postdoctoral Research Fellow<br>Black Dog Institute	• Trial data backup per Research Data Management Plan<br>• Management of trial data<br>• Data steward				
**Dr Omar Ibrahim**<br>Postdoctoral Research Fellow<br>Black Dog Institute	• Assist with participant recruitment<br>• Assist in the day-to-day operation of the participant screening process<br>• Assist with mini-trial conduct and monitoring	19/01/2022	31/12/2022		
**Ms Jodie Rosenberg**<br>Research Assistant<br>Black Dog Institute	• Participant recruitment<br>• Mini-trial conduct and monitoring<br>• Verification and analysis of screening and trial data<br>• Completion and documentation of Pre-trial and Trial Initiation Monitoring Procedure<br>• Active monitoring of the study email address for participant risk disclosures<br>• Maintenance of Safety Monitoring Register	12/07/2021	12/07/2022		
**Dr Aimy Slade**<br>Research Assistant	• Participant recruitment<br>• Day-to-day trial operations	21/03/2022	30/06/2023		

Full name / Position	Responsibilities	Start date	End date	Staff signature and date	PI signature and date
Black Dog Institute	• Mini-trial conduct and monitoring<br>• Active monitoring of the study email address for participant risk disclosures<br>• Assist in the maintenance of Safety Monitoring Register				
**Prof Svetha Venkatesh**<br>Co-Director<br>Applied Artificial Intelligence Institute, Deakin University	• Overall supervision of all staff based at Deakin University<br>• Oversight of algorithm and software development activities	01/01/2021	31/12/2022		
**Dr Sunil Gupta**<br>Associate Professor<br>Applied Artificial Intelligence Institute, Deakin University	• Implementation and validation of multi-armed bandit algorithm	01/01/2021	31/12/2022		
**Dr Manisha Senadeera**<br>Associate Research Fellow<br>Applied Artificial Intelligence Institute, Deakin University	• Implementation and validation of multi-armed bandit algorithm	01/01/2021	31/12/2022		
**Prof Rajesh Vasa**<br>Head of Translational Research	• Implementation, validation and operation of cloud data collection platform	01/01/2021	31/12/2022		

Full name / Position	Responsibilities	Start date	End date	Staff signature and date	PI signature and date
Applied Artificial Intelligence Institute, Deakin University	• Development of study smartphone app<br>• Maintenance of secured randomisation list<br>• Code breaking on study completion or if required by Data Safety Monitoring Board<br>• Data steward				
**Dr Leonard Hoon**<br>Senior Research Fellow<br>Applied Artificial Intelligence Institute, Deakin University	• Implementation, validation and operation of cloud data collection platform<br>• Development of study smartphone app<br>• Verification and analysis of screening and trial data<br>• Completion and documentation of Pre-trial and Trial Initiation Monitoring Procedure<br>• Day-to-day operation of trial software systems	01/01/2021	31/12/2022		
**Dr Rena Logothetis**<br>Associate Research Fellow<br>Applied Artificial Intelligence Institute, Deakin University	• Assist with day-to-day trial management per Clinical Trial Protocol (Deakin University)<br>• Assist with participant recruitment (Deakin University)	01/01/2021	31/12/2022		

Full name / Position	Responsibilities	Start date	End date	Staff signature and date	PI signature and date
	• Assist with mini-trial conduct and monitoring (Deakin University)<br>• Assist with maintenance of Safety Monitoring Register (Deakin University)<br>• Assist with staff training delivery and record keeping per Training Policy (Deakin University)<br>• Assist with mandatory record keeping per Clinical Trial Protocol and Grant Funding Agreement (Deakin University)<br>• Assist with verification and analysis of screening and trial data<br>• Assist with the completion and documentation of Pre-trial and Trial Initiation Monitoring Procedure				
**Dr Priya Rani**<br>Postdoctoral Fellow<br>Applied Artificial Intelligence Institute, Deakin University	• Assist with day-to-day trial operations per Clinical Trial Protocol (Deakin University)<br>• Assist with participant recruitment (Deakin University)<br>• Assist with mini-trial conduct and monitoring (Deakin University)	30/06/2021	31/12/2022		

Full name / Position	Responsibilities	Start date	End date	Staff signature and date	PI signature and date
	• Assist with staff training delivery and record keeping per Training Policy (Deakin University)<br>• Assist with verification and analysis of screening and trial data<br>• Assist with the completion and documentation of Pre-trial and Trial Initiation Monitoring Procedure				
**Mr Stuart Cameron**<br>Principal Software Engineer<br>Applied Artificial Intelligence Institute, Deakin University	• Implementation, validation and operation of cloud data collection platform<br>• Development of study smartphone app<br>• Day-to-day operation of trial software systems	01/01/2021	31/12/2022		

Appendix 16 Training Policy

The creation of records in the Training Log will not be treated as a notifiable protocol change.

Vibe Up Study Training Policy

Version dated: 15 Mar 2021

1. Introduction

The International Council for Harmonisation (ICH) Good Clinical Practice (GCP)[1] stipulates that each member of the clinical trials team must be 'qualified by education, training and experience' to discharge his/her role in the study (ICH GCP 2.8). The Principal Investigator (PI) is responsible for supervising any individual or party to whom they have delegated tasks at the clinical trial site.

To provide evidence of compliance with this requirement to regulatory authorities, records of clinical trials team experience, education and training are required. The maintenance of up-to-date training records provides a means of demonstrating the adequate training and experience of staff involved in the conduct of clinical trials. Training can be obtained through multiple sources, including university-run or accredited third party clinical trials training.

2. Purpose

The purpose of this policy is to state the training requirements and associated record-keeping procedures for staff involved in the Vibe Up clinical trial.

3. Scope

It applies to:

- The Principal investigator (PI) (ICH GCP 1.34) and Subinvestigators (ICH GCP 1.56) named in the Clinical Trial Protocol and;
- All staff with assigned responsibilities relating to the conduct of this clinical trial during the recruitment and mini-trial phases, including trial monitoring and interim analyses.

Staff involved in the secondary analysis of data after the trial completes are not covered by these requirements.

4. Responsibilities

It is the responsibility of the Study Sponsor (UNSW Sydney) to ensure that the Principal Investigator has the expertise and experience necessary to successfully conduct the proposed clinical trial.

It is the responsibility of the PI (Helen Christensen) to ensure that staff members, including Subinvestigators, who work on their clinical trials have the expertise and experience necessary to successfully conduct the proposed clinical trial. This responsibility includes managing the training and development of the clinical trial team, supervising their role in the trial, and ensuring that this is documented. This responsibility applies regardless of which institution a staff member is based at.

In the Vibe Up Study, the following PI responsibilities (from those described above) are delegated:

- Responsibility for the supervision of staff based at Deakin University is delegated to Prof Svetha Venkatesh (and her delegates, as required).
- Responsibility for managing CVs, and managing and documenting the completion of appropriate trial-related training (regardless of institution) is delegated to the Research Project Manager (WuYi Zheng.)

- Responsibility for the development and delivery of Vibe Up study-specific training is also delegated to the Research Project Manager.
- Responsibility for the coordinating the process of procedure review required by at Trial Initiation (ICP GCH 8.2.20) is also delegated to the Research Project Manager.

It is the responsibility of all staff covered by this policy to:

- Provide an up-to-date copy of their CV, if required (see **Curriculum vitae (CV) requirements**, below).
- Ensure specific training required by this policy has been completed (see **Training requirements**, below);
- Read and agree by signing the **Data Access Principles** described in the **Vibe Up Research Data Management Plan** (Appendix 23 to the Clinical Trial Protocol).
- Read and understand the **Optimise Publications Policy** (Appendix 17 to the Protocol).
- Identify any skills gaps relevant to their role in the trial and ensure that these are addressed in good time before their role in the study starts;
- Provide evidence of completed training on request; and
- Maintain their own records of training received.

5. Curriculum vitae (CV) requirements

The PI and all Subinvestigators must provide a copy to hold on file of their up-to-date CV documenting their qualifications, skills and experience relevant to the study and supporting their role in this clinical trial (ICH GCP 4.1.1).

6. Training requirements

The following table defines the training required for different staff types under this policy.

Staff type	Required training	Provider	Recency
PI, Research Project Manager, BDI/Deakin Trial Leads	ICH GCP Training	Accredited GCP provider	Within past 3 years
Research Project Manager, BDI/Deakin Trial Leads	Research Data Management Online Training (RDMoT)	UNSW	Within 3 months of starting role in study/policy effective date, whichever is later
All staff in Scope	Research Integrity Training	UNSW/Deakin	Within past 3 years
All staff in Scope	Vibe Up study-specific training (See **Section 7,** below)	Research Project Manager	Within 3 months of starting role in study/policy effective date, whichever is later
All staff in Scope	Vibe Up RDMP Data Access Principles	Self-guided	Within 3 months of starting role in study/policy effective date, whichever is later
All staff in Scope	Optimise Publications Policy	Self-guided	Within 3 months of starting role in study/policy effective date, whichever is later

2092

7. Vibe Up study-specific training

This training is intended to ensure that all staff understand:

- The basic purpose, design and principles of operation of the Vibe Up trial;
- Where critical study information can be found;
- Standard Operating Procedures applicable to their role; and
- Their responsibilities concerning research good practice, confidentiality, participant safety and compliance with audit/oversight.

Training will be developed and delivered by the Research Project Manager.

8. Record keeping

Details of completed training will be maintained by the Research Project Manager in the **Training Log** (template in **Appendix 1**).

This log will be maintained by the Research Project Manager and detail the training status of all applicable staff including training received/not received. For completed training, it will detail the date, provider (if applicable) and evidence of completion.

At the start of each quarter, the Research Project Manager will provide a training status update to the PI (via the zzOptimise IMC), flagging any staff that have failed to complete their training requirements.

Version history

Date	Summary of changes
01 Dec 2020	Document created.
15 Mar 2021	Updated to include review of Data Access Principles and Publications Policy as training requirements.

References

1. International Council for Harmonisation of Technical Requirements for Pharmaceuticals for Human Use (ICH), *Integrated Addendum to ICH E6(R1): Guideline for Good Clinical Practice*. 2016.
2. Dobell-Brown, K., *Documentation of Training and Clinical Trial Handover*. 2020, South Wester Sydney Local Health District: Sydney, NSW, Australia.

2093

Appendix 1 – Vibe Up Study Training Log

Staff member	Project role	Training type	Provider	Completed date	Expires date	Evidence of completion	Logged by	Logged date

HC Number: HC200466

Page 4 of 4
Version dated: 15 Mar 2021

Appendix 17 Publications Policy

Revisions to the publications policy will not be treated as a notifiable protocol change.

Optimise project publications policy

Version/effective from date: 23 Sep 2021

1. Overview

The purpose of this document is to lay out the principles and procedures that govern the creation, approval and authorship of publishable research outputs arising from the MRFAI "Optimising treatments in mental health using AI" project and its sub-studies.

2. Scope

This policy applies to all publishable research outputs that are:

- Based on, or principally informed by, data generated by the Optimise project and its sub-studies; and/or
- Proposed by a named investigator, partner or other staff member acting in their capacity as a contributor working towards the specific aims of the project.

A publishable research output is any authored output intended for dissemination in a public forum, whether peer reviewed or otherwise. It includes, but is not restricted to journal articles, commentaries, reviews, conference presentations, online content, posters and reports.

3. Policy statement

The Optimise project involves researchers from multiple research groups spread across three universities plus external partners. The principal aim of this policy is to ensure quality, transparency and fairness in the publications process for all project collaborators.

To do this, the Optimise Executive has established a set of common **Publication principles** that apply to all publishable outputs. These are described in **Section 4**, below.

The principles are enforced by the Optimise **Publications committee**, which serves to review and approve all proposed publications and their authorship. The membership and terms of reference for this committee are presented in Appendix 1.

The **Publication process**, described in **Section 7** below, explains the steps involved in proposing and securing approval for a proposed publication.

The policy also sets clear expectations about when it is appropriate to involve external collaborators as authors (see **Section 6**).

Approval for publication is *separate* from governance processes concerning access to data, for example data required to complete the principal analysis informing an intended publication. Access procedures are governed by the **Research Data Management Plan**.

4. Publication principles

The following principles apply to all publishable research outputs. They are intended to guide the decisions of the Publications Committee. They are informed by the Guidelines on Collaborative Research and Authorship published by the University of Sydney Centre for Values, Ethics and the Law in Medicine.

4.1 Responsibility to publish

Researchers have a responsibility to publish. This responsibility extends to the dissemination of negative findings. It also means ensuring that selected publication venues/formats are appropriate and accessible to a wide audience.

4.2 Inclusiveness

All people registered as involved in the project (i.e. named investigators and research team members who may have been recruited subsequent to the initial funding application) have default 'rights' to propose their inclusion as an author on any publication linked to the project.

The Optimise publication tier assigned to each paper (see **Section 5**) establishes clear expectations about who should be included as authors by default. All papers must have a defined and agreed publication tier.

4.3 Warrant for authorship

Notwithstanding the principle of inclusiveness, there are no automatic or 'gift' authorships. Academic integrity and transparency are paramount. All authors must fulfil the International Committee of Medical Journal Editors (ICMJE) requirements of authorship:

http://www.icmje.org/recommendations/browse/roles-and-responsibilities/defining-the-role-of-authors-and-contributors.html

4.4 Recognition of effort

Authorship should reflect principally the effort of manuscript creation. That is, those who assume the *Principal Author* role and/or write the paper should have first authorship options. Ideally, the Principal Author role should be designated before writing commences.

However, in large scale projects, such as Optimise, authorship must also recognise the foundational effort that has enabled a paper to be written. This means, in particular, considering the contributions of those that designed and implemented the study and its systems, as well as those involved in data collection, per:

https://jamanetwork.com/journals/jama/article-abstract/2673143

If contribution is not sufficient to recognise authorship, contributors should (with consent) be recognised in an Acknowledgement section of the manuscript or output.

4.5 Recognition of intellectual leadership

Research papers should appropriately reflect, through authorship, the intellectual leadership of core components of the research project, as reflected in the **MRFAI Optimise - Team Structure**, accessible via Teams or on request from the Research Project Manager, WuYi Zheng.

Members of the wider team contribute intellectual leadership of the project across a range of areas including study operations, software and algorithm development.

4.6 Fairness and accountability when deciding authorship

The following general principles shall guide authorship selection and roles:

4.6.1 Authorship will be explicitly negotiated for all publications arising from the project. It is appropriate to begin negotiations before writing commences, and to revisit negotiations again after writing finishes.

4.6.2 All publications must have at least one *Principal Author*. For some interdisciplinary works, it may be appropriate to designate a Co-Principal Author who will be listed second in the author list with the designation "contributed equally".

4.6.3 The Principal Author is responsible for the delivery of the manuscript and all practical administration tasks, for example, identifying an appropriate venue for publication; identifying and ensuring compliance with requirements of the selected publication venue, ensuring timely submission and coordinating any required revisions or resubmissions.

4.6.4 Principal Authorships should be equitably allocated across team members (after taking into account the skills/knowledge necessary to produce the output.)

4.6.5 The default assumption is that the Principal Author will be the lead (first) author. The Principal Author is responsible for the publication venue and list of authors for review by the Publications Committee and negotiating the ordering of the approved author list with the agreed co-authors.

4.6.6 All publications must have a single *Senior Author*. They are accountable for the delivery of the manuscript and its quality. They are responsible for deciding the publication venue and resolving any issues relating to authorship ordering or intellectual content that cannot be solved by the Principal Author.

4.6.7 The default assumption is that the Senior Author will be the last (supervising) author and also be the designated corresponding author.

4.7 Open access publication

Wherever possible, publication should target venues that support open access. If a selected publication supports open access (e.g. for an additional fee), then this option must be selected and budgeted for.

Financial support for Open Access publication will be made available, if required, for all publications agreed by the Publications Committee.

4.8 Academic integrity

All authors involved in publications agreed under this Publications Policy agree to be bound by the expectations of conduct and integrity described in the following UNSW policies:

- UNSW Code of Conduct
- UNSW Plagiarism Policy Statement

Concerns about integrity may be identified by any staff member and must be reported without delay to the Principal Investigator, Prof Helen Christensen. Any concerns that involve the Principal Investigator should be notified to Prof Sean Emery, Vice Dean (Research and Operations), UNSW Medicine.

Substantiated concerns will be handled according to the UNSW Allegations of Research Misconduct Procedure.

5. Publication tiers

All publications covered by this protocol shall have an assigned publication tier, agreed by the Publications Committee. Each tier defines a set of requirements that any publication in that tier should follow.

Tier 1

A restricted set of *Key Project Papers* that present either (a) the vision of the project, (b) the initial findings of the project, or (c) the principal findings of the project. All relevant members from all groups will serve as co-authors for this paper. There may be multiple Tier 1 papers, all of which should receive high priority.

Tier 2

These consist of a series of papers that make use of the cross-disciplinary skills of two or more teams, as well as the resources, data, or knowledge that derive from our larger collaboration. The Tier 2 papers will together form a cohesive body of work.

All Tier 2 papers must cite the Key Project Paper. All relevant members from all groups (decided by the team leaders) will serve as co-authors for these papers. Each group should have the opportunity to lead a Tier 2 paper.

Example(s): All papers presenting an analysis of the Future Proofing Project, the Deakin Mental Health Project, the ARC HUB Project, or the Uniting Agewell Project.

Tier 3

These consist of a series of papers that make use of the intra-disciplinary skills of one team, but rely on resources, data, or knowledge that derive from our larger collaboration. This includes sub-projects that might spin-off as PhD chapters. These can only be scheduled after Tier 1 and Tier 2 projects are already planned and scheduled.

All Tier 3 papers must cite the relevant Key Project Paper, and any relevant Tier 2 papers. Early career researchers should be encouraged to lead Tier 3 papers as Principal and Senior Authors.

Example(s): Machine learning conference papers making use of collected data. A perspective piece on adaptive clinical trials in mental health.

Tier 4

These consist of any papers that involve an external group as the lead investigator but make use of the resources, data, or knowledge that derive from our larger collaboration. Tier 4 projects should begin only after approval from the **Optimise Project Executive**. If involving project-generated data, then data approvals under the **Research Data Management Plan** must also be secured.

All Tier 4 papers must cite the relevant Key Project Paper, and any relevant Tier 2 papers. At least 1 relevant member from the team will serve as a co-author on this paper, as selected by the Project Executive. Their role is to ensure that the resources, data, or knowledge is interpreted and used according to best practices.

Example(s): An external collaborator wants to analyse our data with respect to a clinical demographic we exclude from our analysis. They apply for data access and lead the project. Members from our team maintain close contact with their team and support their work.

6. Including external collaborators as authors

A cross-disciplinary collaboration consists of several groups. Each group brings with them their own expertise. For this collaboration, the groups and expertise are:

- A2I2-1 (team leader: Svetha Venkatesh): Machine learning and data analytics;
- A2I2-2 (team leader: Kon Mouzakis): Software engineering
- MUCHE (team leader: Henry Cutler): Health economics
- Black Dog Institute (team leader: Helen Christensen): Mental health and trial biostatistics

For all activities that fall within the project scope, each group will be the **exclusive provider of their respective expertise**. This means that no group will collaborate with an external group for this project if the external group's expertise overlaps with the expertise of any group within the team. A group may recruit external collaborators with different expertise than those within the team but should keep this recruitment procedure transparent.

If all team leaders agree, an external group with overlapping expertise may be recruited to perform clearly defined duties (e.g., to form an Expert Advisory Group).

7. Publication process – required steps

The Optimise publications process is intended to be simple while ensuring fairness, transparency and appropriate review of proposed publications. There are five required steps:

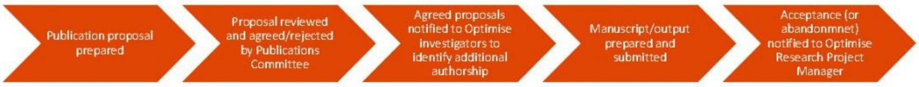

Step 1 – Publication proposal prepared and submitted for review by Publications Committee
For all new publication proposals, the template provided in **Appendix 2** should be completed and submitted to Research Project Manager, WuYi Zheng, who will arrange review by the Publications Committee.

Incomplete proposals will be returned to the proposing author.

Step 2 – Review by Publications Committee
The publication proposal will be reviewed by the Publications Committee at its next scheduled meeting. There are three possible outcomes:

- Agreed;
- Agreed with required modifications (e.g. to proposed tier, title/scope or authorship); or
- Rejected with reason(s).

Agreed proposals will be added to the **Optimise Publications Register** by the Research Project Manager. Rejected proposals cannot be resubmitted.

Step 3 – Notification of proposed publication to Optimise investigators
Agreed publication proposals will be notified by email to all Optimise investigators and team members by the Research Project Manager. Following principle **4.2**, all investigators will be given the opportunity to propose their inclusion on the author list for the proposed publication.

Individuals who wish to propose their inclusion on the author list must do this by responding by email, incorporating a brief written justification, to the Principal Author within 7 working days.

Per **4.6.5**, it is the responsibility of the Principal Author (with the support of the Senior Author, if required) to negotiate whether to accept each proposed inclusion and, subsequently, to finalize the authorship ordering.

Step 4 – Output prepared and submitted

Step 5 – Notification of publication changes, acceptance or abandonment
The following changes/outcomes must be promptly notified to the Research Project Manager by the Principal or Senior Author:

- Changes to the intended venue or format of the publication;
- Acceptance for publication; in which case the details of the publication venue – including the final authorship list/ordering – must be provided;
- Abandonment.

8. Conferences

This section refers to any conference presentation associated with the Optimise project, including poster presentations, and oral presentations.

Authorship on a conference abstract and presentation should reflect the team that contributed to the research, and take accountability for its conduct, analysis and interpretation of findings. Criteria for authorship is generally the same as for publications.

Number of authors included on a submission will depend on whether a limit is imposed by the conference.

- In cases where a limit is imposed, the first author will determine the authorship list, taking into account expertise of the team and the level of contribution to the abstract. Where possible, the first author shall strive for an even distribution of authors from BDI and Deakin University.

- Where no limit is imposed, the first author shall include all interested project team members on the submission. An email shall be sent to Wu Yi Zheng (BDI) & Rena Logothetis (Deakin University) in the first instance, who will gauge interest within their team and respond to the first author with names of interested team members to include on the submission within 3 working days. Where possible, the first author shall strive for an even distribution of authors from BDI and Deakin University. The first author, in consultation with the project team, will determine the order of authorship.

All conference presentations (including poster presentations) should list the entire project team to acknowledge their contributions, along with the source(s) of funding, any conflicts of interest, ethics approval numbers, and trial registration numbers (i.e. ANZCTR). A copy of the slide, project team list, and funding source(s) will be made available to the project team and accessed via Wu Yi Zheng.

The project manager will maintain a list of all accepted conference abstracts, but it is the responsibility of the first author to send the citation to all co-authors immediately after the conference (within 3 working days).

9. Dispute resolution

Any disagreements about authorship or publication scope that cannot be resolved through discussion by the involved parties will be decided by the Principal Investigator.

Version history

Date	Summary of changes
01 Dec 2020	Document created.
20 Jan 2021	Edits proposed by A2I2 (SV/TQ).
22 Jan 2021	Edits accepted/integrated.
06 Apr 2021	Data request process added.
20 Jul 2021	Section on conferences added.
23 Sept 2021	Updated list of available data and glossary

Appendices

Appendix 1 – Optimise Publications Committee Terms of Reference

Purpose

The purpose of the Optimise Publications Committee is to:

1. Review and agree proposals for publication arising from the Optimise project by applying the Optimise Publication Principles to ensure quality, fairness and transparency in the publications process;
2. Ensure that the contributions of all project stakeholders, across all participating institutions, are fairly and equitably recognised; and
3. Ensure effort is appropriately directed to high-quality outputs and avoid duplicate publications.

Term

These Terms of Reference are effective from the Version Date (23 Sep 2021) until the conclusion of the Optimise Project (29 Jun 2023) or until terminated by the Principal Investigator, whichever is sooner.

Membership

The Optimise Publications Committee will comprise:

- Members of the Optimise Executive, as defined in the **MRFAI Optimise - Team Structure.** Membership is automatic for as long as an individual is a named member of the Executive.

Chair

The Publications Committee is chaired by the Optimise Principal Investigator, Prof Helen Christensen.

Quorum

To convene, the Publications Committee requires two representatives each from Black Dog Institute (which may include the chair) and Deakin University Applied Artificial Intelligence Institute to be present.

Committee Responsibilities

The Committee is responsible for:

1. Reviewing each proposed publication and deciding whether it should proceed by:
   - Fairly applying the criteria specified in the **Optimise Publication Principles**, and
   - Attending to the project objectives and existing/expected publications proposals to ensure high-quality outputs commensurate with the project and grant objectives and to avoid duplication of effort/outputs.

   There are three possible review outcomes from which the committee must decide:

   - Agreed;
   - Agreed with required modifications (e.g. to proposed tier, title/scope or authorship); or
   - Rejected with reason(s).

   Decisions not receiving unanimous support and which cannot be resolved through discussion shall be made through majority open vote, moved by the Chair. Any ties will be broken by the Chair.
2. Identifying any required modifications (e.g. missing authors) for any proposal it has decided to agree.

3. Providing at least one substantive reason to be communicated to proposing authors for proposal it has decided to reject.

Administration

The Publications Committee will be administered by the Research Project Manager, WuYi Zheng.

They will:

1. Collate, validate and provide to the committee for review all new publication proposals.
2. Record as minutes the decisions of the committee including:
    a. Any required modifications to a proposed publication;
    b. If rejected, the reason(s) for this to be communicated to the proposing authors.
3. Promptly communicate committee decisions to proposing authors;
4. Arrange notification of newly accepted proposals to Optimise investigators and team members (who may wish to propose their inclusion as authors);
5. Maintain a register of proposed and accepted publications.

Modification

These Terms of Reference may be amended, varied or modified by the Principal Investigator or the quorate Committee at any time.

Appendix 2 – Optimise Publication Proposal Template

All fields must be completed. Please submit completed forms to the Optimise Research Project Manager, WuYi Zheng (wuyi.zheng@blackdog.org.au).

**Output type**	Choose an item.
**Publication tier** (See **Section 5**)	Choose an item.
**Draft title**	Enter a draft title for the proposed publication. This title can be revised later, as required.
**Principal Author** (See **Section 4.6**)	Type a name.
**Senior Author** (See **Section 4.6**)	Type a name.
**Other Author(s)** (Assumed unordered)	Type a name. Use the plus button to add additional authors.
**Brief description/justification**	Enter a brief description (2-3) sentences of the purpose/focus of the intended publication and how it aligns with the Optimise project objectives.
**Target publication venue(s)**	Enter the intended journal, conference or publication venue name. If more than one venue is being considered, use the plus button to add additional rows, listing in decreasing order of preference.
**Intended submission date** (Assumed provisional)	Enter the intended submission date.

Appendix 3 – Optimise Data Request Application Form

Purpose and scope

This form is to be completed and submitted to the Optimise Publications Committee by applicants when requesting access to data collected during the Optimise Project. Approval of this application and evidence of other applicable approvals (e.g. ethics) are required before study data can be granted, regardless of whether the applicant is internal or external to Black Dog Institute, UNSW or Applied Artificial Intelligence Institute (A2I2), Deakin University.

By submitting this application, you are agreeing to the terms and conditions specified by the Optimise Publications Committee. These are provided at the end of this application form.

Requirement for Sponsor

Before submitting this form, each applicant must identify a 'Sponsor' drawn from the Optimise Project investigator teams (e.g. Vibe Up subinvestigators, etc.). The Sponsor will be a co-investigator on the applicant's project and will act as the liaison between the applicant and the Optimise Project.

If you require assistance finding a Sponsor, please email a brief outline of your research proposal to the Project Manager, Wu Yi Zheng. Further details about sponsorship can be found on page 17.

Summary of the application process

Once an applicant has discussed the details of their research project with a Optimise Project Sponsor, they must complete and submit this form. While the information in the application does not need to be extensive, it does need to be specific about the aims and context of the proposal so that these can be checked for potential overlap with other proposals. The applicant must specify the intended output(s) of their project; listing intended publications, presentations, and/or student theses, and include an estimated completion date for each.

Completed applications will undergo review by the Optimise Project to ascertain whether the data requested are available and the proposal is consistent with the study's scientific objectives. We aim for the entire review process to be completed within 4 weeks from the date of submission. If the application is incomplete when submitted, or if further information is required to assess the application, the review process may take longer. These details will be communicated to the applicant as soon as possible and applications will be expedited wherever possible.

The delivery date of the requested data will be discussed and negotiated during the application and review process. If approved, the applicant will be asked to sign a Research Agreement / Memorandum of Understanding / Student Deed Poll before any data will be released.

Timelines for planned output

For each planned output item (e.g., publication, student thesis, etc.), the applicant will be contacted after the nominated due date for the item and asked to provide a progress update. If there has been no output as proposed or no satisfactory update provided 12 months after the nominated due date, the Optimise Project reserves the right to revoke approval for the application and you may be required to delete all data provided to you.

Contact information

If you require further information about this form or the application process, please contact the Project Manager, Wu Yi Zheng, by email Vibeup@blackdog.org.au.

Office use only:

**Submission date**	
**Application ID**	

Section 1: Applicant information

1.1 Lead investigator's details:

**Name**	
**Position**	
**Institution**	
**Address**	
**Email**	
**Phone**	

1.2 Co-investigators' details:

Please ensure that this list contains <u>all</u> people who will have access to Optimise data, should this request be granted.

a) Researchers listed in the Subinvestigator list of the Vibe Up Study Protocol

**Name**	**Position**	**Email**

b) Researchers <u>not</u> listed in the Subinvestigator list of the Vibe Up Study Protocol

**Name**	**Institution**	**Email**

c) If applicable, please describe the collaboration between listed subinvestigators and other researchers (NB. The Optimise Project recommends that researchers from different institutions complete a Memorandum of Understanding to outline their expectations and responsibilities before undertaking collaborative research).

d) Does this project form part of or contribute to any aspect of a student project? **YES / NO**

If Yes, please provide details (incl degree, institution, supervisor, etc):

1.3 Optimise Project sponsor/s

Name	Role on Vibe Up Study

Section 2: Project description

**Project name**	

**Brief outline of proposed research (500 words max.)**<br><br>*You may attach a detailed proposal at the end of this application.*	

**Summary of data being requested**	
**Please explain why these data are required**	
**Last date after which data will no longer be required?**<br><br>(If known)	

**What kind of output(s) will your project produce?**<br><br>(e.g. publications, conference	

presentations, new datasets, etc)<br>*Please provide a general description of all likely project IP. Specific descriptions should be included in Section 3 below.*	
**Will your project yield any *new* variable(s) or dataset(s)?**<br>(A new variable would be generated, for example, if Optimise data were transformed to generate a participant-specific risk score not present in the original data.)<br>*If so, please describe.*	

Section 3: Planned outputs

**Output type**<br>(e.g. publications, student theses, new datasets, etc)	**Description**	**Authors/contributors**	**Due date**

Section 4: Other information

**Ethics approval number for your project**<br>Note: Your project must have ethics approval for your data request to be approved.	

**Name of ethics committee**	

**Data Custodian**<br><br>Lead organisational unit(s) (e.g. university) establishing policies/procedures that govern data handling by the team proposing data.	Name:
**Data Owner**<br><br>Responsible for ensuring effective local protocols are in place to guide the appropriate use of their data asset. Access to, and use of, institutional data will generally be administered by the appropriate Data Owner. Data Owners (or a delegated Data Steward) are also responsible for ensuring that all legal, regulatory, and policy requirements are met in relation to the specific data or information asset.	Name:<br><br>Job title:<br><br>Email:<br><br>Telephone:

**Please list any known or potential overlaps/conflicts with other research using these data**<br><br>If you are declaring conflicts of interest, please explain how you will mitigate/address these.	

**If you have any further comments regarding this application, please provide them here**	

Applicant acknowledgement

By submitting this application, I/we the investigators listed on this application agree that:

i. Any data released by the Optimise Project in response to this application shall be used only as specified in this application unless an amendment or modification to the application is submitted and approved by the Optimise Project (this includes changes to project staff.)

ii. Any data released by the Optimise Project in response to this application shall not be passed on to anyone not named in this application.

iii. I/we declare that I/we will not attempt any procedure that either as its intended purpose, or as a side effect, may result in the identity of any Optimise participant becoming known.

iv. We will inform the Optimise Project of any changes to the planned output of this application and understand that the Optimise Project may nominate investigators and potential co-authors to be included on any such additions.

v. Any changes to the ethics approval for this project which occur after the submission of this application shall be communicated immediately to the Optimise Project.

vi. The Optimise Project may revoke their approval of this application and request the immediate return of all data if I/we are found to be in breach of items (i) to (iv), or if the changes specified in (iv) are deemed to be inconsistent with the Optimise Project's own compliance requirements.

vii. If this application is approved:

- In the case of an applicant that is not a Black Dog Institute, UNSW or A2I2 employee or student, your research organisation will be required to sign a Research Agreement (in the form set out in the attachment to this Application) before any data are released.
- In the case of an applicant that is a Black Dog Institute, UNSW or A2I2 employee, you may be required to sign an acknowledgement of any special conditions that apply to your use of the data (such as in relation to the costs that will need to be borne by your Faculty/School).
- In the case that the applicant is a UNSW or A2I2 student, they may be required to sign a deed poll before any data are released.

viii. I/we will adhere to all terms and conditions for accessing, using, securely storing and destroying data as specified in the following sections of this application.

Terms and Conditions

General research practice and ethics

The guidelines in this document, as well as the general research practice of the Optimise Project, are based on the following UNSW and NHMRC documents:

A. National Statement on Ethical Conduct in Human Research (NHMRC)
https://www.nhmrc.gov.au/about-us/publications/national-statement-ethical-conduct-human-research-2007-updated-2018

B. Australian Code for the Responsible Conduct of Research (NHMRC)
https://www.nhmrc.gov.au/about-us/publications/australian-code-responsible-conduct-research-2018

C. Research Code of Conduct (UNSW)
https://www.gs.unsw.edu.au/policy/researchcode.html

D. Intellectual Property Policy (UNSW)
https://www.gs.unsw.edu.au/policy/ippolicy.html

E. Research - Authorship and Resolving Disputes Between Authors Procedure (UNSW)
https://www.gs.unsw.edu.au/policy/researchauthorproc.html

F. Research - Handling Research Material & Data Procedure (UNSW)
https://www.gs.unsw.edu.au/policy/researchdataproc.html

These documents provide the principles and foundations for the research conduct and practise adopted by the Optimise Project. The following sections in this document are included to either highlight sections of the reference guidelines that are particularly relevant to the Optimise Project and/or to address issues not specifically discussed in the reference documents. The process for addressing and resolving disputes should be guided by documents (C) and (E) above.

Applying to access data

Parties interested in accessing Optimise Project data are required to complete the application form in this document. All applicants are required to agree to the terms and conditions specified in this document. Review of these applications will go through a two-stage review process:

i. Before submitting a research project application to the Optimise Project, the applicant is required to find a 'Sponsor' for their project, drawn from the Optimise Project investigator team. The Sponsor will be a co-investigator on the applicant's project and will act as the liaison between the applicant and the Optimise Project. It is recommended that the applicant and their team agree on the nature of the Sponsor's role in writing to clarify the terms of the collaboration. Applicants must also confirm that they have appropriate ethics approval from their organisation to access and use Optimise Project data and must provide evidence of this approval in their application. Should an applicant's organisation not require them to obtain ethics approval for their project, then the applicant must explicitly state this in their application. However, under no circumstances may an applicant knowingly or intentionally contravene the right to privacy or confidentiality granted to research participants under Australian law and/or the policies and documents listed in *1. General research practice and ethics*.
ii. Completed applications will then undergo review by the Optimise Project to ascertain whether the data requested are available and the proposal is consistent with the study's scientific objectives. The application will be assessed for basic feasibility, i.e., are the requested data available, how long would it take to respond to the application, and within what time frame could data be provided to the applicant if the application is approved. We aim for the entire review process to be completed within 4 weeks. All applications must include details of the ethics approval for the proposed project. Projects are not covered by the ethics approval of the Optimise Project unless all investigators named on the application and who will have access to participant-level data released to this project are named personnel on the Optimise Project.

Once an application is approved, the Optimise Project may require the applicant to sign a Research Agreement stipulating any specific conditions required by the Optimise Project in addition to the terms and conditions described in this document.

Before approving an application, the Optimise Project may request modifications to the project. These will be made on a case-by-case basis but should adhere to the principles of good research practice (see the Australian Code for the Responsible Conduct of Research (NHMRC, 2007)). As part of the review and feedback process, the Optimise Project may also ask the project team to consider the inclusion of additional investigators and/or potential co-authors on the project. Should the applicant not wish to include the requested modifications, additional investigators or potential co-authors, then this will be resolved by negotiation or, if this is not possible, the decision of the Optimise Project will prevail.

IMPORTANT NOTE: Data released by the Optimise Project can only be used for the purposes and planned output stated in the approved project application. Any additional use must first be approved by the Optimise Project and under no circumstances can data be shared between projects or researchers. Doing so represents a breach of these terms and conditions and, depending on the circumstances, may be in breach of privacy legislation under Australian State and Commonwealth law.

Approval

Once an application is approved, you will receive written conditional approval which will confirm the data you will receive, conditions of approval including the data retention date and required standards for handling data securely. It is a requirement of approval that you comply with these conditions. Data files will be provided via secure electronic transfer, accompanied by a data dictionary and explanatory notes. Updates to projects

After a project has been approved and data have been released to the applicant:

i. The addition of new investigators to the project must be recorded with the Optimise Project with justification. The Optimise Project may query and/or deny the addition of new investigators.

ii. Requests for additional data must be submitted to the Optimise Project and should be accompanied with justification. These requests will be reviewed by the Optimise Project as per the procedures for new applications in *2. Applying to Access Data*.

iii. Should the project require substantial changes to its aims, these should be discussed with the Optimise Project by the Sponsor. The applicant may be asked to submit a formal request to update the project. Data may not be used for a project with aims other than are stated on their approved application without additional approval from the Optimise Project.

iv. Should the planned output of the project change (e.g., additional publications, modifications to planned manuscripts, etc.), these must be submitted to the Optimise Project for review.

v. If a project computes/produces a new variable or dataset based on Optimise Project data, this variable and/or dataset must be made available to the Optimise Project. These new variables will then be available (pending application and approval) to other projects. The Sponsor should keep the Optimise Project updated of the development of any new variables. Any concerns or alterations relating to these requirements should be resolved and stated clearly in the Research Agreement.

In summary, any deviation of (or addition to) the project from the original approved application requires the approval of the Optimise Project. In cases where the applicant has previously signed a formal contract with the Optimise Project regarding the approved application, substantive updates to the project may necessitate a formal variation to the contract. The Optimise Project shall defer to the guidance of UNSW Legal in all cases.

Publishing and releasing findings

The Optimise Project must be notified in advance of all publications and presentations using Optimise Project data. As discussed above, failure to gain the approval of the Optimise Project before publishing or otherwise disseminating Optimise Project data represents a breach of our terms and conditions and will be dealt with accordingly.

All planned output items (i.e., publications, theses, etc.) must be listed on the project application. Should an output item be planned after an application has been approved, the applicant must submit an updated application to the Optimise Project (see *4. Updates to projects*) and must gain approval for this update before proceeding. Once a manuscript is accepted for publication, a copy must be sent to the Optimise Project for archiving.

Review of progress

Projects are required to produce output items by the date specified in their approved application. If 12 months have elapsed since the nominated deadline and output items are still not produced, the Optimise Project reserves the right to revoke its approval for the project to use its data.

Should a project be facing difficulties reaching its specified deadlines, the Sponsor should inform the Optimise Project as soon as possible. Depending on the circumstances of the individual case, the Optimise Project may allow for the project's nominated deadlines to be revised.

If a project is unable to proceed for reasons such as a lack of significant findings, staff turnover, etc., the Sponsor should discuss the matter promptly with the Optimise Project for resolution. All outcomes must be reported to the Optimise Project.

Requests to access identifying data or cohort details

The Optimise Project will not release any identifying information under any circumstances.

No project or user may undertake any activity that, either as its intended purpose or as a side-effect, results in the identity of any Optimise participant becoming known.

Participant confidentiality and data security

General guidelines

The principles regarding data security and participant confidentiality are outlined in *1. General research practice and ethics.*

In addition, the Optimise Project requires that:

- Applicants will respect the guarantee of confidentiality given to research participants whose data they use.
- Applicants will not present information in any form that would enable others to identify research participants.
- Only individuals explicitly named in this application may be granted access to Optimise Project data. No data (including copies) are to be given to parties not named in this application.

Release of data for publications

Some journals require that the data analysed and reported on in publications must be made available for review or validation of statistical analyses. Researchers using data from the Optimise Project may make data available at their discretion so long as the data is provided in a summarised or aggregated form.

If publishers require that participant-level data be made available, then researchers should state that data can be accessed by making an application to the Optimise Project. If the publisher does not accept this then Optimise Project data may be made available if the following conditions are met:

i. Participant-level data should only be made available if publishers expressly require the researcher to do so.

ii. Researchers who are required to make participant-level data available in order for their manuscripts to be accepted for publication must notify the Optimise Project as soon as possible. The Optimise Project can advise researchers how any necessary privacy requirements pertaining to participant-level data can be upheld so as to not impact the likelihood of acceptance of the manuscript.

iii. The researcher takes all responsibility for ensuring they have permission from the Optimise Project to make data available and, once made available, for responding to all requests to access data used in their publications.

Criteria for authorship

Right to authorship should adhere to best research practices and be based on the contribution of an individual to a given manuscript/project.

The Optimise Publications Committee Terms of Reference provide authoritative guidance on how authorship shall be assigned for all Optimise investigators/staff.

Nominating authors

Although the ways in which a research project or publication is realised can vary greatly, any project using Optimise Project data shall allow the following opportunities for others to nominate authors:

i. Initial submission and review of project application: All applications must include a list of investigators. However, during the review of the application, the Optimise Project may nominate additional people who the applicant must consider for inclusion as investigators and

potential co-authorship. The applicant is required to contact and discuss potential collaboration with any such nominees. The Sponsor for each project is responsible for managing these discussions and their outcomes.

ii. At completion of the manuscript outline: A copy of this outline should be forwarded to the Optimise Project for review. During this review, the Optimise Project may again nominate additional people who should be considered for involvement and/or authorship. Again, the Sponsor is responsible for managing these discussions.

iii. Before submitting manuscripts for review/publication: Each potential co-author should have had the opportunity to review and revise the final draft manuscript and should be provided with a copy of the submitted manuscript. Co-authors must acknowledge this via Statement of Authorship. Note also, before submitting any revisions to the draft manuscript, the corresponding author must forward reviewer responses and the updated manuscript to all co-authors.

- Note: These rules also apply to conference abstracts.

Criteria and Statement of Authorship

All research projects are required to complete a Statement of Authorship declaration before publishing any research output in any format. Criteria for authorship should accord with the 'Vancouver Protocol' and be guided by the Australian Code for the Responsible Conduct of Research (NHMRC, 2007). As such, authorship is substantial participation where all the following conditions are met:

i. Conception and design of the research project OR analysis and interpretation of data; AND

ii. Drafting the article OR revising it critically for important intellectual content; AND

iii. Final approval of the version to be published.

In addition, the following guidelines should be considered:

- Participation solely in the acquisition of funding or the collection of data does not justify authorship.
- General supervision of the research group is not sufficient for authorship.
- Any part of an article critical to its main conclusion must be the responsibility of at least one author.
- An author's role in a research output must be sufficient for that person to take public responsibility for at least that part of the output in that person's area of expertise.
- No person who is an author, consistent with this definition, must be excluded as an author without their permission in writing.
- No person must be listed as an author without his or her knowledge. This also applies for conference abstracts.

Required acknowledgements

All applicants will be advised of any required acknowledgements following review of their application. Required acknowledgements will be stipulated in the Research Agreement and are a condition of access to and use of Optimise Project data.

Please indicate all data being requested by placing an **X** in the appropriate box(es) and specifying the relevant mini-trial numbers. A glossary of all self-report measures can be found on pages 14-15. Add additional comments as appropriate.

**SELF-REPORT DATA**	**Timepoint**							**Mini-trial**		**Comments**
	Screening	Baseline	EMA	Mid	Intervention	Post	Follow-up	All available	Specified:	
Basic eligibility (ELY)										
Recruitment pathway (RTP)										
Physical and mental health (MED)										
Kessler Psychological Distress Scale, 10-item version (KTEN)										
Extended Suicidal Ideation Attributes Scale (SIDAS)										
Demographic details (DEM)										
Study and employment (WRK)										
Productivity Costs Questionnaire (PCQ)										
Use of Mental Health Care Services (MHS)										
EQ-5D-5L (EQV)										
Subjective Socioeconomic Status Scale (SES)										
Abridged NIDA-Modified ASSIST Drug Screening Tool (AOD)										
Short Warwick Edinburgh Mental Wellbeing Scale (WBS)										

SELF-REPORT DATA	Timepoint							Mini-trial		Comments
	Screening	Baseline	EMA	Mid	Intervention	Post	Follow-up	All available	Specified:	
Multidimensional Scale of Perceived Social Support (PSS)										
Depression, Anxiety and Stress Scale, 21-item version (DASS)										
Modified Physical Activity Vital Sign (PAVS)										
Abridged Pittsburgh Sleep Quality Index (PSQI)										
Mindfulness single item questionnaire (MIND)										
Modified Positive and Negative Affect Schedule, Short Form (PANAS)										
Behavioural intentions (BHV)										
Abridged Credibility and Expectancy Questionnaire (CEQ)										
Abridged Revised University of Rhode Island Change Assessment Scale (URC)										
Daily log of engagement with intervention (LOG)										
Within-study exposures questionnaire (EXP)										
UX questionnaire (UX)										

SMARTPHONE DATA	Mini-trial		Comments
	All available	Specified:	
Accelerometer			
Activity			
Battery			
Distance			
Gyroscope			
Location			
Step count			

Please note: Some of the datasets listed above consist of multiple mini-trials. In order to expedite the data request you may be supplied with data from all mini-trials. Please note that if you are issued more data than requested then you are only authorised to use/publish/present the data discussed in your research outline (i.e., being issued surplus data does not mean the applicant is authorised to publish or present this data).

Glossary

Self-report data	Description
Abridged Credibility and Expectancy Questionnaire	Validated measure of intervention credibility and expectancy, including only questions 1 (Set 1, question 1) and 6 (Set 2, question 2), selected as single items with highest distinct loadings on credibility/expectancy factors.
Abridged NIDA-Modified ASSIST Drug Screening Tool	Validated measure of alcohol, tobacco, prescription drugs for non-medical reasons and illegal drugs over the past year.
Abridged Pittsburgh Sleep Quality Index	Validated measure of sleep quality, including only question 6 (measuring overall sleep quality).
Abridged Revised University of Rhode Island Change Assessment Scale	Validated measure of readiness for change, abridged to select the two questions accounting for the highest proportion of variance explained for each of the ambivalence, seeking assistance and action factors (3 × 2).
Basic eligibility	Age, student status, residential status, English language ability, smartphone access, expected disruptions, and ability to participant in physical activity.
Behavioural intentions	Two bespoke questions assessing behavioral intentions in response to current mood state (measured by the Modified Positive and Negative Affect Schedule, Short Form).
Daily log of engagement with intervention	Single item question measuring time spent engaging with intervention specific activities.
Demographic details	Gender identity, sex recorded at birth, sexual orientation, Aboriginal and Torres Strait Islander origin, ancestry, language spoken at home.
Depression, Anxiety and Stress Scale, 21-item version	Validated measure of depression, anxiety symptoms and stress.
EQ-5D-5L (EQV)	Health-related quality of life measures, which may be used in clinical and health economic research.
Extended Suicidal Ideation Attributes Scale	Validated measure of suicidal ideation, plus additional question on history of suicide attempt(s).
Kessler Psychological Distress Scale, 10-item version	Validated measure of psychological distress.
Mindfulness single item questionnaire	Bespoke questions measuring subjective rating of overall mindfulness during past week.
Modified Physical Activity Vital Sign	Validated measure of physical activity, modified to consider only the most recent week.
Modified Positive and Negative Affect Schedule, Short Form	Validated measure, modified to consider current mood state and with the addition of 2 items from the original PANAS focused on distress, specifically ('hopeless' and 'calm').
Multidimensional Scale of Perceived Social Support	Validated measure of perceived social support (12 items).
Physical and mental health	Diagnosed chronic physical health condition and/or mental health condition.

Productivity Costs Questionnaire (PCQ)	The PCQ collects information on an individual's capacity to undertake paid and unpaid work.
Recruitment pathway	Where the participant heard about the study.
Short Warwick Edinburgh Mental Wellbeing Scale	Validated measure of mental wellbeing (7 items).
Study and employment	International student status, academic performance, work status.
Subjective Socioeconomic Status Scale	Single item, validated, visual analogue scale measuring subjective socioeconomic status.
Use of Mental Health Care Services (MHS)	The MHS collects information on an individual's use of health and community care services. This includes hospital services, medical services, allied health services, community based services, and diagnostic services.
UX questionnaire	Bespoke questions, based on the System Usability Scale and mHealth App Usability Questionnaire (8 items).
Within-study exposures questionnaire	Bespoke questions assessing perceived impact of events/disruptions on daily life, mental health and app use during the study period.

Appendix 18 Recruitment Strategy

Vibe Up Study Recruitment Strategy

Version dated: 10 Mar 2021

1. Purpose

The objective of the Study Recruitment Strategy is to outline proposed activities to ensure adequate recruitment across the 12 months of mini-trials within Vibe Up. The Study Recruitment Strategy is intended as a guide only and additional recruitment procedures are likely to be required.

2. Study eligibility criteria

Inclusion criteria:

- 18 years or older;
- Living in Australia;
- Currently studying at an Australian university, TAFE or other higher education institution;
- Fluent in English;
- Own an up-to-date smartphone, with active mobile number and internet access; and
- Are experiencing psychological distress (e.g., anxiety, stress or low mood).

Exclusion criteria

- People who have experienced significant thoughts of suicide in the past month.
- People with a current diagnosis of psychosis or bipolar disorder.
- People who have previously participated in this study.
- People who have limited availability to participate in the next 2 months.
- People who can't safely undertake physical activity for any reason.

Please note, although participants will be discouraged from undertaking new mental health interventions during the four-week mini-study period, there are no restrictions on the medication(s) or treatment(s) that participants can receive prior to or during the trial.

3. Recruitment considerations

- The study needs a sample of 1200 participants, over 12 mini-trials across 12 months. Tapping into fresh pools of potential participants will be necessary to sustain high recruitment numbers.

- Allowing for attrition (i.e., eligible participants not starting, or not finishing, the study), approximately 120 participants need to be recruited per mini-trial. The upper limit on participants per trial is flexible and can be increased in the early stages of the study (i.e., during 2021) to capitalise on higher initial interest, if this occurs.

- Participants cannot start the app experience immediately upon screening as eligible; they need to wait until the mini-trial start date. Mini-trials will always start on a Thursday. Recruitment for an upcoming mini-trial will close at midnight on the Monday prior to the mini-trial start date, to allow time for data to be transferred from UNSW to Deakin and amalgamated into the study platforms.

- We need to ensure that participants who screen eligible to participate in the study re-engage when their allocated mini-trial starts.

- Eligible participants who do not respond to the app download invitation and/or do not complete the baseline questionnaire with a 72-hour period will be automatically referred forward to the next mini-trial. Re-engaging this sub-group may be particularly challenging.
- Eligible participants can also elect to delay their start until the following mini-trial (if another mini-trial is due to commence soon).

- We need to aim for a balance of gender amongst participants, which will require variation in copy and visual assets for advertisements.

4. Recruitment timeline and targets

The below dates and recruitment targets are provisional and may be adjusted based on early recruitment outcomes and/or study results.

Study	Study start date (Thursday)	Recruitment dates	Recruitment target
Pilot 1	20/05/21	23/04/21 – 17/05/21	120 participants
Pilot 2: mini-trial 1	22/07/21	Early Jul 2021 – 16/08/21	Total of 240 participants (n = 120 per mini-trial)
Pilot 2: mini-trial 2	19/08/21		
Main study: mini-trial 1	28/10/21	Early Oct 2021 – 15/11/21	Total of 240 participants (n = 120 per mini-trial)
Main study: mini-trial 2	18/11/21		
UNSW/BDI shutdown	*Exact dates unknown.*		
Main study: mini-trial 3	03/02/22	Mid Jan 2022 – Jun 2022	Up to approximately 600 participants in the first half of 2022 (provided adequate recruitment in 2021).
Main study: mini-trial 4	24/02/22		
Main study: mini-trial 5	3 weeks from above		
Mini-trials will continue, starting every 3 weeks (with 2 weeks of recruitment prior), until the research stopping criteria is met or 9 mini-trials in the main study period have been completed (whichever is sooner).			
Main study: mini-trial 9	09/06/22*		

*NB: this date reflect the most efficient possible completion of 9 mini-trials (commencing every 3 weeks, starting in late January 2022). Mini-trials may be spaced out further if necessary (e.g. for technical troubleshooting).

5. Proposed recruitment avenues

Social media

Based on previous experience and the literature on recruitment for digital health interventions (Darmawan et al., 2020; Sanchez et al., 2020), **targeted paid social media advertising** is likely to be the most fruitful recruitment avenue.

Free social media will also be used to compliment paid social media advertising campaigns. For example, posts can be made or shared by BDI, UNSW, Deakin or affiliated stakeholders or advocates for the study (see list below).

Platforms

Based on user testing for Vibe Up and the research literature (Ford et al., 2019; Guillory et al., 2018), **Facebook and Instagram** are likely to be the most fruitful platforms for recruitment via social media. Instagram advertising is run through the Facebook advertising platform, making simultaneous campaigns easy to coordinate and

2221

monitor. Facebook and Instagram allow for more specificity in targeting than other platforms, and have been used most widely in the research literature; although there are mixed findings as to whether Facebook has higher conversion rates than other platforms such as Google Ads. Instagram and Snapchat ads are a more recent development, so there is less published research on using those platforms for recruitment. Based on user testing for Vibe up, LinkedIn and Youtube may be helpful platforms, secondary to Instagram and Facebook.

Budget

Based on similar previous campaigns at BDI, a budget of $6.00 per enrolled (eligible) participant has been set, for a total budget of approximately $7500.

Other avenues

- BDI networks
  - Give a talk to the Discovery Portfolio in June, to seek assistance from other researchers.
  - Fundraising email list (EDM)
  - Fundraising contacts, particularly from *Mullets for Mental Health* campaign
  - BDI Volunteer Research Registry
  - Education Teams' network of presenters with lived experience
  - Volunteer network from the Future Proofing study.
  - Education Team's database of university contacts from previous education presentations
  - Teams' contacts at UNSW
- Deakin networks
  - Student Services, via contact through Rena Logothesis.
- Media releases through BDI, UNSW and Deakin
- Student organisations
  - Student minds at UNSW (driven by students and supported by UNSW's Health Promotion Unit): https://student.unsw.edu.au/studentminds
- University societies
  - Coordinated by Arc @ UNSW and Deakin University Student Association (DUSA).
- University counselling and psychological services
- University psychology training clinics (e.g. http://www.clinic.psy.unsw.edu.au/)
- University health services
- University wellbeing coordinators (program level)
- University academics/teaching staff
- University colleges/residential coordinators
- Posters on university campuses
- University volunteering pages
- Research registries
- Posting on Gumtree

6. Evidence-driven adaption

The study team will meet regularly to review and adapt the recruitment strategy.

A multiple-choice question will be included in the eligibility screening questionnaire for the study, to allow the team to monitor the effectiveness of recruitment methods. The question will be early in the screening, to capture information from participants who do not complete the questionnaire or are ineligible. The question will enable the team to analyse patterns of interest in the study and conversion to enrolment and focus more resources on the most effective strategies.

Continuous monitoring of performance and refinement of our paid social media campaign will be critical. BDI's Digital and Social Media Officer will monitor advertisements each business day and hold fortnightly meetings

2222

with the BDI study team (specifically, the Research Project Manager and Clinical Postdoctoral Fellow) when campaigns are active. By starting with several ads, we will monitor which is most effective in real time and continue with these, and then revise ads over time to sustain engagement.

7. Recruitment material

BDI's Marketing and Communications Team will develop a collection of images and copy to be submitted for ethics approval, in consultation with the study's User Experience (UX) Designer and the research team.

The collection of images and copy will be flexibly combined to create various advertisements, to aid evidence-driven adaption of recruitment material (Lattie et al., 2018; Watson et al., 2018).

References

Darmawan, I., Bakker, C., Brockman, T. A., Patten, C. A., & Eder, M. (2020, Oct 26). The Role of Social Media in Enhancing Clinical Trial Recruitment: Scoping Review. *J Med Internet Res, 22*(10), e22810. https://doi.org/10.2196/22810

Ford, K. L., Albritton, T., Dunn, T. A., Crawford, K., Neuwirth, J., & Bull, S. (2019, Oct 9). Youth Study Recruitment Using Paid Advertising on Instagram, Snapchat, and Facebook: Cross-Sectional Survey Study. *JMIR Public Health Surveill, 5*(4), e14080. https://doi.org/10.2196/14080

Guillory, J., Wiant, K. F., Farrelly, M., Fiacco, L., Alam, I., Hoffman, L., Crankshaw, E., Delahanty, J., & Alexander, T. N. (2018, Jun 18). Recruiting Hard-to-Reach Populations for Survey Research: Using Facebook and Instagram Advertisements and In-Person Intercept in LGBT Bars and Nightclubs to Recruit LGBT Young Adults. *J Med Internet Res, 20*(6), e197. https://doi.org/10.2196/jmir.9461

Lattie, E. G., Kaiser, S. M., Alam, N., Tomasino, K. N., Sargent, E., Rubanovich, C. K., Palac, H. L., & Mohr, D. C. (2018, Nov 29). A Practical Do-It-Yourself Recruitment Framework for Concurrent eHealth Clinical Trials: Identification of Efficient and Cost-Effective Methods for Decision Making (Part 2). *J Med Internet Res, 20*(11), e11050. https://doi.org/10.2196/11050

Sanchez, C., Grzenda, A., Varias, A., Widge, A. S., Carpenter, L. L., McDonald, W. M., Nemeroff, C. B., Kalin, N. H., Martin, G., Tohen, M., Filippou-Frye, M., Ramsey, D., Linos, E., Mangurian, C., & Rodriguez, C. I. (2020, Nov). Social media recruitment for mental health research: A systematic review. *Compr Psychiatry, 103*, 152197. https://doi.org/10.1016/j.comppsych.2020.152197

Watson, N. L., Mull, K. E., Heffner, J. L., McClure, J. B., & Bricker, J. B. (2018, Aug 24). Participant Recruitment and Retention in Remote eHealth Intervention Trials: Methods and Lessons Learned From a Large Randomized Controlled Trial of Two Web-Based Smoking Interventions. *J Med Internet Res, 20*(8), e10351. https://doi.org/10.2196/10351

Appendix 19 Mini-Trial Conduct and Monitoring Procedure

Vibe Up Mini-Trial Conduct and Monitoring Procedure

Version dated: 18 Oct 2021

1. Purpose

Monitoring is necessary to protect the rights and safety of study participants involved in the Vibe Up trial, and to ensure the quality and integrity of the submitted data.

The objectives of the conduct and monitoring procedure are:
- To ensure that the study is being carried out in accordance with the approved trial protocol;
- To identify any problems and suggest/seek solutions [1].

2. Scope

This Conduct and Monitoring Procedure serves as a Standard Operating Procedure and is applicable to the investigators, study management group, and the study team.

3. Mini-trial conduct and monitoring

Monitoring will focus on the following key processes of the study to ensure protection of rights and well-being of study participants and integrity of data:

1) Informed consent process: each participant has provided informed consent;

2) Study eligibility criteria met for all participants: each participant meets the inclusion criteria of the trial;

3) Timely completion of the Unexpected and Serious Adverse Event Form and/or the Significant Safety Issue Notification Form;

4) Review of data management procedure i.e. data entry, handling of data discrepancies and data backup;

5) Reporting of adverse events and protocol violations: to ensure privacy and safety of participants.

3.1 Screening

Participant screening is hosted by BDI on the Vibe Up study website, and screening questionnaires will be administered using Qualtrics. Data collected during the online screening process are transferred from BDI to Deakin University prior to the commencement of a mini-trial, as the study app and the optimisation platform are hosted by Deakin University. Responsibility for ensuring the safe and secure exchange/transfer of Vibe Up study data rests upon the following BDI and Deakin University Staff members: Wu Yi Zheng (BDI research project manager), Eileen Stech (BDI clinical postdoctoral research fellow), Leonard Hoon (Deakin project manager), Rena Logothetis (Deakin research manager) and Scott Barnett (Deakin senior research fellow).

3.1.1 Exchange/Transfer of information from BDI to Deakin University

Data transfer will involve the following steps:

1. Export the latest completion data from Qualtrics by using UNSW username and password;
2. Filter exported data by selected/allocated mini-trial start date, and identify and exclude those who have received two invitations, to display participants for the upcoming mini-trial only;

3. Collate the preferred name, Qualtrics ID, mobile number, email address, and sensor data collection consent status of 'screened eligible and consented' participants into a spreadsheet that is identified by mini-trial number;
4. Name each spreadsheet according to the agreed naming protocol (e.g. Screening data – mt# - YYYYMMDD);
5. Password-protect each spreadsheet before transfer to the Deakin team, with passwords accessible to authorised staff members only. Random passwords will be generated using https://www.guidgenerator.com and will not be re-used;
6. Transfer mini-trial spreadsheets to Deakin University via UNSW OneDrive, by 12 noon on the Tuesday prior to the mini-trial start day (Thursday).
7. Transferred data will be retrieved by authorised Deakin staff (Leonard Hoon, Rena Logothetis, or Scott Barnett), assigned with UNSW zIDs, from the designated UNSW OneDrive folder;
8. Receipt/successful processing of data will be confirmed by email to the BDI research manager (Wu Yi Zheng).

3.1.2 Exchange/Transfer of information from Deakin University to BDI

The team at Deakin will identify participants who were non-responders and need to be automatically referred forward to the next mini-trial. Participants will be given a maximum of two opportunities to participate in a mini-trial. Data transfer will involve the following steps:

1. Export experimental data from the most recently completed mini-trial in the database; this includes:
    - Participants who failed to download the app and/or complete the baseline questionnaires within 72 hours;
    - Participants who have been invited twice;
    - Active and passive data for the most recent trial (post hoc);
    - Participants who had failed to complete the mid-point surveys within the allotted time;
    - Participants who had failed to complete the end-point surveys within the allotted time.

2. Name each file according to the agreed naming protocol (e.g. Experimental Data – mt# - YYYYMMDD);
3. Password-protect the files before transfer to the BDI team, with passwords accessible to authorised staff members only. Random passwords will be generated and will not be re-used;
4. Transfer mini-trial files to BDI via UNSW OneDrive within 5 working days of the mini-trial ending;
5. Transferred data will be retrieved by authorised BDI staff from the designated UNSW OneDrive folder. Microsoft Teams file sharing is the backup option if OneDrive fails;
6. Receipt/successful processing of users by the BDI team will be confirmed by email to the Deakin research manager (Rena Logothetis).

Any problems identified during data transfer shall be reported to the BDI research manager and the Deakin Research manager.

3.2 Mini-trial

Conduct of mini-trials shall be as per the design outlined in the approved clinical trial protocol. Monitoring shall follow the Protocol Deviation Identification and Reporting Procedure, and the Data Verification and Analysis Procedure.

3.3 Post-trial

After the completion of each mini-trial, it is the responsibility of the BDI research manager to ensure that gift tokens are sent to eligible participants. Data (preferred name and email address) on participants who had completed the study will be transferred from Deakin University to BDI using password-protected files via OneDrive.

It is the responsibility of the BDI research manager to liaise with his counterpart at Deakin University to ensure that required data for the personalised participant report are securely transferred to BDI. The research team will endeavour to send this report to participants within 7 days of study completion.

It is the responsibility of the Deakin University research manager to ensure that content from all three interventions are made available to all participants after each mini-trial.

3.4 Contact outside of planned data collection

The research team can be contacted by participants, potential participants, or other interested parties via an email address provided within the app and on public facing communications about the study (e.g. BDI website). Access to the Vibe Up study email account (Vibeup@blackdog.org.au) is restricted to authorised study personnel only (BDI and Deakin Staff members with access: Kit Huckvale, Jill Newby, Eileen Stech, Wu Yi Zheng, Joanne Beames, Leonard Hoon, & Rena Logothetis). To ensure that each incoming participant email is assessed and assigned to an appropriately qualified team member to address the participant contact, the following steps must be followed:

1. All emails shall remain in the primary inbox unless it has been assessed and allocated to an appropriate member within the research team. This means that all emails in the inbox require action to be taken;
2. Allocated emails should be moved to the 'action in progress' folder;
3. For urgent matters that require immediate action, emails shall be moved to the 'urgent' folder;
4. It is the responsibility of the BDI research manager (Wu Yi Zheng) to follow up on each email in the 'action in progress' folder on a weekly basis; and emails in the 'urgent' folder every 48 hours;
5. Resolved emails are moved to the 'action complete' folder by the BDI research manager (Wu Yi Zheng)

To maintain accurate trial records and ensure data privacy is upheld, assigned team members will respond to email enquiries from within the Vibe Up email account (emails will not be forwarded to team members' individual accounts).

Participant emails shall be allocated to the following staff members:

1. Compliments/complaints (Wu Yi Zheng)
2. Technical problems (Rena Logothetis/Leonard Hoon)
3. Disclosure of risk or heightened distress (Wu Yi Zheng will coordinate response from a BDI clinician)
4. Interest in deferring their intake into another mini-trial (Wu Yi Zheng)

3.4.1 Participant complaints

Participant complaints shall be allocated to the BDI research manager (Wu Yi Zheng) in the first instance. The participant shall be responded to within 48 hours. If a resolution is required, the complaint will be tabled, discussed, and resolved at the next Trial Operations Committee meeting. The BDI research manager will communicate the resolution to the participant in question within 24 hours of the meeting.

3.4.2 Technical problems

Technical problems shall be allocated to the Deakin Research Manager (Rena Logothetis) in the first instance. A ticket will be created in Jira (Issue & Project Tracking Software) and an engineer will be assigned to resolve the issue. The Deakin Project Manager (Leonard Hoon) will include the ticket into the active sprint in the Project Roadmap.

3.4.3 Disclosure of risk or heightened distress

Emails that suggest heightened distress or contain disclosure of risk will be allocated to the BDI research manager (Wu Yi Zheng) in the first instance. The BDI Research Manager is responsible for arranging a BDI clinician to reply to the contact within 48 hours, per the Psychological Safety Response Procedure.

Version history

Date	Summary of changes
01 Dec 2020	Document created.
18 Oct 2021	Version finalised for Pre-trial report

References

1. The Global Health Network, *Monitoring Plan and Standard Operating Procedure*. 2021.

Appendix 20 Protocol Deviation Identification and Reporting Procedure

Vibe Up Protocol Deviation Identification and Reporting Procedure

Version dated: 18 Oct 2021

1. Purpose

This document describes the process for identifying and reporting deviation(s) from the clinical trial protocol approved by the governing ethics committees. A protocol deviation is defined as any breach, divergence or departure from the requirements of Good Clinical Practice or the clinical trial protocol [1]. These may be the result of human error when the deviation is investigator or trial management related or they could be participant-related, where participants misunderstood or ignored guidance given to them whilst they are in the study [2].

2. Scope

This document applies to all BDI and Deakin University staff involved in the conduct of the Vibe Up trial.

3. Identification of protocol deviations

Protocol deviations will be identified by:

- Proactive identification by performing random data checks;
- Ad hoc reporting by any member of study team at any time during the trial;
- Analysis of collected safety parameters by the Data Safety Monitoring Board; and
- Participant self-reports provided via an email-based reporting mechanism.

In the majority of instances, protocol deviations do not result in harm to trial participants or significantly affect the scientific value of the reported results of the trial [3]. A serious deviation is likely to affect to a significant degree:

(a) The safety or rights of the trial participant;

(b) The reliability and robustness of the data generated in the clinical trial.

Any BDI or Deakin University staff member involved in the conduct, management or monitoring of this clinical trial can identify suspected protocol deviations. Suspected deviations should be recorded in the Protocol Deviation Tracking Log within 24 hours of identification (**Appendix 1**).

4. Reporting of protocol deviations

Members of the trial team are made aware of the process for reporting protocol deviations during training and induction.

The research project manager at BDI (Wu Yi Zheng) and his counterpart at Deakin University (Leonard Hoon) will ensure all identified protocol deviations (minor or major) are recorded, and reported to the study Principal Investigator (PI). For all events identified as serious and/or unexpected, the **Unexpected and Serious Adverse Event Notification Form** must be completed within 72 hours and reviewed/approved by the Study's Qualified Medical Expert (Prof. Jennie Hudson) and the Principal Investigator.

Where a protocol deviation has been assessed by the PI and the Study's Qualified Medical Expert as possibly meeting the definition of a serious breach (as defined in 3. Identification of protocol deviations), this will be reported to the trial sponsor (UNSW) within 72 hours of the site becoming aware of the deviation. If the sponsor confirms, after investigation, that a serious breach has occurred, this is reported to UNSW HREC and Deakin University HREC within 72 hours of this confirmation.

Version history

Date	Summary of changes
01 Dec 2020	Document created.
18 Oct 2021	Version finalised for pre-trial report

References

1. Johns Hopkins Medicine. *Reporting Protocol Deviations*. 2016 [cited 2021 03 February]; Available from: https://www.hopkinsmedicine.org/institutional_review_board/guidelines_policies/guidelines/protocol_deviations.html.
2. Queensland Health. *Australian ICH GCP (Including Teletrials) SOP 50 Appendix 1: Example Protocol Deviation Log*. 2019 [cited 2021 03 February]; Available from: https://www.health.qld.gov.au/__data/assets/pdf_file/0032/836195/AUSTRALIAN-ICH-GCP-SOP-50_Appendix-1_V4.0-Watermarked.pdf.
3. NSW Health. *Clinical Trial Toolkit: supporting clinical trials at site*. 2020 [cited 2021 03 February]; Available from: https://www.medicalresearch.nsw.gov.au/clinical-trial-toolkit/.

Appendix 1: Protocol Deviation Tracking Log (adapted from [2])

Protocol/HREC Number:					Principal Investigator:			
Protocol Title (Abbreviated):					Site Name:			
Record number	Participant ID	Reported by (full name)	Date of deviation	Date identified	Description of protocol deviation	Consequence of deviation	Did participant continue in the study (yes/no)?	Reported to Sponsor/Research office (yes/no)?/date of report

Staff signature: _______________

Investigator signature: _________________

Date: _________________

Appendix 21 Data Verification and Analysis Procedure

Vibe Up Data Verification and Analysis Procedure

Version dated: 04 Nov 2021

1. Purpose

The purpose of this document is to outline the procedures and identify staff members responsible for performing any data verification that is not performed by the system and analysis. Data verification is the process of evaluating the completeness and correctness of a data set [1]. These steps are undertaken to ensure data integrity and validity.

2. Scope

This document applies to all staff with assigned responsibilities relating to the conduct of this trial, from participant screening to the running of mini trials, including trial monitoring and interim analysis.

3. Verification and analysis of data

Data verification shall occur at every stage of data collection, with each verification episode recorded in the data verification log (**Appendix 1**). Identified problems (incomplete or incorrect data) shall be reported to the Research Project Manager at BDI (Wu Yi Zheng) or to his counterpart at Deakin University (Rena Logothetis) within 24 hours of detection. These will be discussed and resolved by the Trial Operations Committee.

3.1 Participant screening phase

In addition to testing of the Qualtrics survey prior to deployment (as per the Screening and self-report Questionnaire Testing Plan), screening data downloaded from Qualtrics shall be randomly checked on a weekly basis by designated BDI staff members. Particular attention shall be focused on items that determine exclusion of a potential participant (e.g. level of psychological distress), and the item that triggers duty of care actions from BDI clinicians (i.e. level of suicidality). Please refer to **Appendix 2** for the full trial exclusion criteria. In addition, data shall be checked for completeness, with missing data flagged and investigated to determine if the detected problem is widespread (e.g. data on participant gender missing for all participants).

Basic analysis will be carried out to determine demographic breakdown of recruited participants at the start of each mini-trial. For example, age (ELY1), type of smart phone (ELY5a), etc.

3.2 App data collection phase

A technical testing strategy is in place for the App prior to deployment. The solution platform under test is an orchestrated active (survey) and passive (sensor) data collection ingestion engine that extracts features collected from participant users. Synthetic data blocks will be generated to cover both good clean data as well as erroneous and incomplete data. This test is to ensure that the App verifies the data collected in 'real-time' during the trial.

If the data is incorrect or missing, the App will notify the user. For passive data, missing data can be caused by a drop-out in sensor data, in which case the app will prompt the user to reactivate the sensor. For active data, the app will notify the user that the data is incorrect or missing , requiring the user to input the correct data to continue to the next phase. Data validation will also be assessed by the Deakin staff applying the Bayesian Optimisation technique during the mini-trials.

Focus of test:

- Data quality: missing values, valid blank values, sparse vectors, changed data types, additional fields, and missing fields;
- Input validation: only appropriate data formats are persisted;
- Data completeness: all data entered in a given survey is persisted with no missing fields;
- Data format validity: participant generated data can be successfully retrieved for analysis with no mutation to the data;
- Accuracy of mapping: The data entered for each user is the one that is persisted in the database (that is, identity mapping is accurate);
- Protocol adherence: The data is captured to the schedule and structure of the experiment protocol;
- Passive data collection: Data expected within the experiment protocol for passive data is collected and reflects the underlying ground truth.

3.3 Mini-trial phase

Designated BDI staff will randomly select 10 participants during each mini trial to validate the data and ensure that it is complete and correct (e.g. questionnaire scores accurately reflects the scoring protocol) at the following stages:

- following the completion of the baseline survey and prior to the mid survey
- following the completion of the mid survey and prior to the post survey
- following the completion of the post survey

Passive data is automatically validated and verified.

On day 9 of the trial, data will be extracted from the baseline surveys by Deakin University staff and randomly checked (10 participants) for completeness and correctness. Scoring protocols for pre, mid and post surveys are applied to random participant raw data to check that scores are calculated correctly using automation. The data will then be passed through the algorithm (as described below) to allocate participants to the recommended intervention group.

As data collected during the mini trial is via selection (i.e., multiple choice, drop down), to ensure completeness of data, participants cannot submit the surveys or move to the next module unless all data points have been collected. This is verified automatically via the system.

From the active and passive data collected during the baseline phase and findings from previous mini trials, a Bayesian optimisation technique (multi-armed bandit) will be used for partitioning participants into mutually exclusive groups.

Please refer to *Appendix 25 Algorithm Specifications*, for information on hypothesis testing.

3.4 Post-trial phase

On day 30 of the trial, data will be extracted and shared with trial investigators at BDI using OneDrive. Deakin University will run a script to extract details of participants that have completed the study. Data from this group of participants will be checked to assess if study completion criteria are satisfied (i.e. completed all post-surveys). Any problems (e.g. incorrect completion status assigned to a participant) shall be reported to the BDI Trial manager (Wu Yi Zheng), and resolved by the Trial Operations Committee.

Version history

Date	Summary of changes
01 Dec 2020	Document created.
18 Oct 2021	Version finalised for pre-trial report
04 Nov 2021	Details added for random checking

References

1. U.S. Environmental Protection Agency, *Quality assurance handbook for air pollution measurement systems*. 2017.

Appendix 1: Data Verification Log

Time/Date	Type of data verified (e.g. Screening data, mini-trial data, etc.)	Data verified by (full name):	State of verified data (i.e. complete/accurate, incomplete/accurate, complete/inaccurate, or incomplete/inaccurate)	Description of problem (if any)	Action taken (i.e. no action required, attempted to resolve issue, notified research project manager)	Problem resolved?
5/11/2021	Screening, main trial, 1 iteration	Artur Shvetcov	complete/accurate	NA	NA	NA
05/11/21	Screening, main trial, 1 iteration	Rena Logothetis	complete/accurate	NA	NA	NA

Appendix 2: Participant Exclusion Criteria

- Younger than 18 years of age (ELY1 < 18)
- Not currently registered as a student at an Australian university, TAFE or other higher education institution (ELY2 = 0)
- Currently not living in Australia (ELY3a = 1)
- Have plans to travel outside Australia in the next 2 months (ELY3b = 1)
- Have basic English ability (ELY4 = 0)
- Do not own a smartphone with an active mobile number and internet access (ELY5a = 0)
- Own a smartphone that is not Android or iPhone (ELY5a = 3)
- Own a smartphone that does not run Android 5.0 (Lollipop) or higher (ELY5b_Android = 0)
- Own a smartphone that is not iPhone 6S or higher (ELY5b_iPhone = 0)
- Previously registered to take part in the study (ELY6a = 1)
- Expecting major events or disruptions in the next 2 months that will make it difficult to take part in the study (ELY6b = 1)
- Have currently active (MED4 = 1) bipolar disorder (MED3 = 2), or Schizophrenia or psychosis (MED3 = 12)
- Scored less than 20 on the KTEN (likely to be well)
- Scored 21 or more on the SIDAS (high suicidal ideation) – ***trigger duty of care actions***

Appendix 22 Participant Psychological Safety Response Procedure

Vibe Up Psychological Safety Response Procedure

Version dated: 19 Jan 2021

1. Purpose

The purpose of this policy is to state the clinical duty of care procedures related to participants' psychological safety during the Vibe Up clinical trial.

2. Scope

It applies to:

- The Principal investigator (PI) (ICH GCP 1.34) and Subinvestigators (ICH GCP 1.56) named in the Clinical Trial Protocol and;
- All staff with assigned responsibilities relating to the conduct of this clinical trial during the recruitment and mini-trial phases.

Staff from the Black Dog Institute (including the BDI Clinicians listed in **Appendix 1** with letters of support provided in **Appendix 2**), with appropriate clinical mental health qualifications (Psychologist or Psychiatrist currently registered with the Australian Health Practitioner Regulation Agency), will be responsible for providing the clinical follow-up outlined in this document.

3. Elevated risk of suicide at eligibility screening

The Extended Suicidal Ideation Attributes Scale (SIDAS) [1] will be used to assess risk of suicide during the eligibility screening. The SIDAS assesses frequency, controllability, closeness to attempt, level of distress and impact on daily functioning associated with thoughts of suicide. In addition, participants will be asked one question to assess history of suicide attempts: *"Have you ever made a suicide attempt"* (3-item closed choice: No, never; Yes, once; Yes, more than once).

Participants who responses met the criterion for 'high suicidal ideation' (SIDAS total score ≥ 21) are not eligible to participate in the study. For participants reporting high suicidal ideation at screening, an onscreen message will be displayed with:

- Contact information for emergency services and 24/7 crisis support services (e.g., Lifeline)
- Recommendations for online self-help materials for psychological distress.
- Recommendation to seek support and advice from a General Practitioner (GP).
- The option to request a call back from a BDI Clinician during business hours.

Therefore, if a participant requests a clinician call back on a weekend or public holiday, they will be given immediate self-help options and a list of crisis support services (e.g., Lifeline) on screen, and the call back will be attempted the following business day.

When participants indicate a preference for a clinician call back, an automated email will be sent to the research team to alert the Research Project Manager and on-call BDI Clinician. The Research Project Manager will be responsible for ensuring that all alerts are followed up and arranging appropriate Clinician/monitoring cover.

A BDI Clinician will attempt to call the participant within 24 hours, or the next business day if the alert is received on a weekend or public holiday. If the participant does not answer the call, a voicemail will be left (if option to do so is available) and an email will be sent advising the participant that contact was attempted and inviting them to reply with convenient times to reach them. Up to three attempts to call the participant will be made by

2285

BDI Clinicians.

During a requested call back, the BDI Clinician will seek to provide support, assess imminent risk of harm, and make referrals to appropriate services. If the clinician deems there is imminent risk of harm, referral to emergency services and/or acute community mental health services will be made. The clinician will have access to the participant's screening responses to aid their assessment and management, which includes the participant's score on the SIDAS; Kessler Psychological Distress Scale, 10-item version (K10)[2]; and history of mental health conditions.

Contacts will be documented in the **Psychological Safety Response Register** (see **Section 6**).

4. In-app safety measures

Contact information for telephone/online crisis support services (e.g., Lifeline) will be displayed prominently in the app.

The standard list of services that shall be used across the Vibe Up study for example, in the app and in the Participant Information Statement, is provided as **Appendix 4**.

There will be no active monitoring of responses to questionnaires completed in the study app. Any open-ended questions will be annotated to clarify that disclosures will not be actively monitored by the study team and will include a link to the app screen with contact information for telephone/online crisis support services.

5. Disclosure of significant risk outside of planned data collection

Any information indicating significant risk of harm to self or others that is disclosed by a participant or applicant outside the planned data collections (e.g., email to the study team) will be reviewed by a BDI Clinician, and an appropriate course of action decided by the BDI Clinician, in line with our duty of care requirements and as required by law.

To ensure that any request for support is actioned, the study email address (vibeup@blackdog.org.au) will be actively monitored by the Research Project Manager or, in their absence, a named delegate and notified to:

- A BDI Clinician who will triage the disclosure; and
- The Study Qualified Medical Expert (named in the Clinical Trial Protocol.)

All disclosures of risk will be followed up within 48 business hours. It is the responsibility of the Research Project Manager (or their delegate) to ensure that this occurs.

If deemed necessary, an email with options for telephone follow-up and/or self-help options may be sent to the participant.

6. Record keeping

All clinician review of contacts and any subsequent management will be documented in the **Psychological Safety Response Register** (see **Appendix 3**).

This will be securely stored on UNSW OneDrive with access limited to the staff roles named above.

Responsibility for documentation in the register lies with the BDI Clinician(s) responding to an at-risk disclosure.

7. Other supports

The following steps will also be taken to ensure participants are supported throughout the study:

- Contact information for telephone/online crisis support (e.g., Lifeline) will be displayed prominently in the online screening/consent environment, study collateral and within the app (see **Appendix 4**)

2286

- Advice will be provided within the app to help participants address any transient increases in discomfort during the interventions (e.g., helpful responses if transient anxiety sensations are experienced during mindfulness).
- All ineligible participants will be provided with links to alternate online self-help options, and telephone/online counselling services.

There are no restrictions on medication(s) or treatment(s) that can be received by participants prior to or during the trial.

8. Data Safety Monitoring Board (DSMB)

An independent Data Safety Monitoring Board (DSMB) will be established to assess the clinical trial progress; to assess the clinical trial safety data at regular intervals; and make recommendations to the Sponsor on whether to continue, modify, or stop a trial.

Refer to the **DSMB Terms of Reference and Procedure** for further information.

Version history

Date	Summary of changes
01 Dec 2020	Document created.
11 Jan 2021	Documented amended by Eileen Stech and Jill Newby to provide detailed information on procedures.
13 Jan 2021	Link to Psychological Safety Response Register included.
19 Jan 2021	Additions of Clinician Support Letters and support service details.

2289

Appendix 1 – BDI Clinicians

The following BDI clinicians have agreed to support the Vibe Up study. They will be the first port of call for responding to participants identified at potential risk.

Name	Clinical qualification	Registration number	Telephone	Email
Ms Eileen Stech	MPsych (Clin)	PSY0002031247	02 9065 9088	eileen.stech@unsw.edu.au
Dr Jill Newby	MPsych (Clin)	PSY0001292310	0403759122	j.newby@unsw.edu.au
Dr Joanne Beames	MPsych (Clin)	PSY0001960782	02 9382 8776	j.beames@blackdog.org.au

In the event that none of the clinicians named above are available (e.g. due to unexpected absence), support will be sought from other staff with appropriate clinical mental health qualifications employed by Black Dog Institute.

2290

Appendix 2 – Clinician Letters of Support

Black Dog Institute

Prof Helen Christensen
Director and Chief Scientist
Black Dog Institute
Hospital Road
Randwick NSW 2031

18 January 2020

Dear Helen,

Psychologist support for the Vibe Up Study

I am writing to confirm that I am happy to act as a psychologist supporting the Vibe Up Study. I have read the Participant Safety Response Procedure and I am aware that this role will involve responding promptly to any individuals identified at elevated risk of suicide during screening, as well as reviewing contact from, and, if necessary following up with, any participant who contacts the study team with concerns about their mental health at any stage during the study.

I further confirm that:

- I hold an appropriate qualification in clinical psychology and a current registration with the Psychology Board of Australia.
- I am covered by professional indemnity insurance that satisfies the standards established by the Australian Health Practitioner Regulation Agency (AHPRA).

With best wishes,

eileen stech

Ms Eileen Stech

Postdoctoral Fellow and Clinical Psychologist Registrar
Bachelor of Psychology (Honours), Master of Psychology (Clinical) / PhD Candidate
Registration number: PSY0002031247
(02) 9065 9088
eileen.stech@unsw.edu.au

Black Dog Institute

Prof Helen Christensen
Director and Chief Scientist
Black Dog Institute
Hospital Road
Randwick NSW 2031

18 January 2020

Dear Helen,

Psychologist support for the Vibe Up Study

I am writing to confirm that I am happy to act as a psychologist supporting the Vibe Up Study. I have read the Participant Safety Response Procedure and I am aware that this role will involve responding promptly to any individuals identified at elevated risk of suicide during screening, as well as reviewing contact from, and, if necessary following up with, any participant who contacts the study team with concerns about their mental health at any stage during the study.

I further confirm that:

- I hold an appropriate qualification in clinical psychology and a current registration with the Psychology Board of Australia.
- I am covered by professional indemnity insurance that satisfies the standards established by the Australian Health Practitioner Regulation Agency (AHPRA).

With best wishes,

Dr Jill Newby

Assoicate Professor and Clinical Psychologist
PhD/M.Psych(Clinical), B.Psych(Hons 1)
Registration number: PSY0001292310
0403 759 122
j.newby@unsw.edu.au

Black Dog Institute

Prof Helen Christensen
Director and Chief Scientist
Black Dog Institute
Hospital Road
Randwick NSW 2031

19 January 2020

Dear Helen,

Clinical psychologist support for the Vibe Up Study

I am writing to confirm that I am happy to act as a clinical psychologist supporting the Vibe Up Study. I have read the Participant Safety Response Procedure and I am aware that this role will involve responding promptly to any individuals identified at risk of suicide during screening, as well as reviewing and, if necessary, following up any participant who contacts the study team with concerns about their mental health at any stage during the study.

I further confirm that:

- I hold an appropriate qualification in clinical psychology and a current registration with the Psychology Board of Australia.
- I am covered by professional indemnity insurance that satisfies the standards established by the Australian Health Practitioner Regulation Agency (AHPRA).

With best wishes,

Joanne Beames

Dr Joanne Beames

Postdoctoral Research Fellow and Clinical Psychology Registrar
Registration number: PSY0001960782
BPsych (Hons), PhD, MPsychol
02 9382 8776
j.beames@blackdog.org.au

Appendix 3 – Psychological Safety Response Register

The register is a separate Excel spreadsheet. Headings for a single entry are shown below.

Vibe Up Study Psychological Safety Response Register

Version dated: 19 Jan 2021

Trial site	Participant Id	First name	Gender	Age at entry	Screening data		
					K-10 total score	SIDAS score	Mental health history
Select trial site...							

Event type

Elevated risk of suicide at screening: Call-back attempt 1					
Date	Time	Clinical notes	Follow-up email sent?	Clinician	Date recorded in register

Elevated risk of suicide at screening: Call-back attempt 2				
Date	Time	Clinical notes	Clinician	Date recorded in register

Elevated risk of suicide at screening: Call-back attempt 3				
Date	Time	Clinical notes	Clinician	Date recorded in register

HC Number: HC200466

Page 1 of 2
Version dated: 19 Jan 2021

Disclosure of risk outside of planned data collection				
Date	Time	Clinical notes	Clinician	Date recorded in register

Appendix 4 – Standard Support Services

If at any stage during the study, you become distressed or require additional support from someone not involved in the research please call:

**Organisation**	Lifeline<br>*Crisis support and suicide prevention*
**Telephone**	13 11 14 available 24/7
**Online chat**	www.lifeline.org.au/crisis-chat available 19:00 – 23:59 AEDT/AEST
**Web**	www.lifeline.org.au/get-help

**Organisation**	Suicide Call Back Service<br>*Counselling and support for people affected by suicide*
**Telephone**	1300 659 467 available 24/7
**Online chat**	www.suicidecallbackservice.org.au/phone-and-online-counselling/suicide-call-back-service-online-counselling available 24/7
**Web**	www.suicidecallbackservice.org.au

**Organisation**	Beyond Blue<br>*Suport and information for people experiencing depression or anxiety*
**Telephone**	1300 22 4636 available 24/7
**Online chat**	online.beyondblue.org.au available 11:00 – 23:59 AEDT/AEST
**Web**	www.beyondblue.org.au

For information on other online mental health services that are available, including support services tailored to specific communities (e.g. LGBTIQ+, Aboriginal and Torres Strait Islander peoples, carers), and services specific to the state you live in, visit: www.headtohealth.gov.au

2303

References

1. van Spijker, B.A.J., et al., *The suicidal ideation attributes scale (SIDAS): Community-based validation study of a new scale for the measurement of suicidal ideation.* Suicide and Life-Threatening Behavior, 2014. **44**(4): p. 408-419.
2. Kessler, R. and D. Mroczek, *An update of the development of mental health screening scales for the US National Health Interview Study.* Ann Arbor: University of Michigan, Survey Research Center of the Institute for Social Research, 1992.

2304

2305 # Appendix 23 Research Data Management Plan
2306

Vibe Up Research Data Management Plan

Version dated: 15 Mar 2021

1. Purpose

The Vibe Up Research Data Management Plan (RDMP) describes the data that is expected to be acquired or generated during the course of the trial, how these data will be managed, analysed and stored, and the mechanisms that must be used to store, share and preserve data. It is intended to be read in conjunction with the Vibe Up Clinical Trial Protocol.

2. Scope

This RDMP applies to all data acquired or generated during the course of the Vibe Up study, regardless of where it was generated.

3. Governing and related regulations and policies

This RDMP is governed by the following regulations and policies:

- The Australian Code for the Responsible Conduct of Research and its supplementary guides,
- The UNSW Research Data Governance & Materials Handling Policy, Procedure and Data Classification Standard.

4. Guiding principles

Reflecting the personal and potentially stigmatizing nature of mental health status and disclosures, the following core principles guide this RDMP:

1) All data pertaining to participants will be treated as if it were Sensitive Data, per the UNSW Data Classification Standard.
2) Data will always be transported over a restricted set of pre-approved secure channels and stored in a restricted set of pre-approved secure locations.
3) Access to data shall always be limited by purpose/need. This must always be justified.

5. Roles and responsibilities

Key data-related roles, such as the Data Custodian, Research Data Owner and Research Data Steward are specified in **Section 13.2** (Data Roles) of the Clinical Trial Protocol.

The Research Project Manager is responsible for:

- Maintaining a register of the names of people belonging to each the user groups described in **Section 8.2**.
- Arranging periodic backup of essential trial data, according to the schedule/procedure defined in **Section 7.1**.

It is the responsibility of all Vibe Up investigators and trial staff to familiarise themselves with, and comply with the requirements of this RDMP. All staff must read, and agree by signing, the Optimise Data Access Principles stated in **Section 8.1**.

6. Data sources and types

6.1 Data sources

Data collection and storage platforms are specified, with approvals information, in **Section 13.4** of the Clinical Trial Protocol. The data types arising from these systems are listed below.

Data source	Data type(s)	Specification
Qualtrics	Consent, screening questionnaires (ELY, MED, KTEN, SIDAS), contact details (CTD), demographics questionnaire (DEM, WRK, SES, EQV, AOD, WBS and PSS)	**Section 7.3** and **Appendix 8** to Clinical Trial Protocol
Vibe Up App (collated via Optimise system)	Baseline, mid and post questionnaires (DASS, PAVS, PSQI, MIND, CEQ, URC, LOG, EXP and UX)	**Section 7.3** and **Appendix 8** to Clinical Trial Protocol
Vibe Up App (collated via Optimise system)	EMA questionnaires (PANAS, BHV)	**Section 7.3** and **Appendix 8** to Clinical Trial Protocol
Vibe Up App (collated via Optimise system)	Instrumentation data	**Section 7.5** of Clinical Trial Protocol
Vibe Up App (collated via Optimise system)	Digital phenotyping data	**Section 9.2** of Clinical Trial Protocol
Optimise system	Enrolment data, allocation, algorithm models	-
Software repositories	Vibe Up algorithm implementation/codebase	**Appendix 25** to Clinical Trial Protocol
Software repositories	Optimise system codebase	-
Outlook 365	Project emails	-
Teams/OneDrive	Project records	-

6.2 Data classification

All data pertaining to participants will be treated as if it were Sensitive Data, per the UNSW Data Classification Standard. Sensitive designated data is deemed high importance to the Universities capabilities, obligations and strategies.

6.3 Dataset types

6.3.1 Identifiable datasets

For the purposes of Vibe Up, an identifiable dataset is one that contains any of the following fields or data types, in any combination:

- Full or partial name (e.g. initials plus surname);
- Date of birth;
- Personal or other email addresses;
- Mobile or other telephone numbers; and/or
- GPS traces.

A de-identified dataset is, therefore, one that does not contain any of the fields or data types described above.

6.3.2 Participant-level contact details

For the purposes of Vibe Up, participant-level contact details are defined as a) those relating to either a participant and b) consisting of the following fields or data types:

- Study identifier (e.g. Unique identifier, Enrolment Id, etc.);
- Full or partial name (e.g. initials plus surname);
- Date of birth;
- Personal or other email addresses; and/or
- Mobile or other telephone numbers.

Any fields or data types not listed here are not valid participant-level contact details.

6.3.3 Participant safety/clinical support data

For the purposes of Vibe Up, participant safety and clinical support data are defined as:

- Any participant-level data generated in the process of executing the **Psychological Safety Response Procedure** (see **Appendix 22** to the Clinical Trial Protocol) including, but not limited to, clinical records, communications to/from participants and entries in the **Psychological Safety Response Register**.
- Any participant-level data relating to the management of adverse events (see **Section 7.6.4** of the Clinical Trial Protocol), including entries in the **Safety Monitoring Register**.

7. Data management

7.1 Backup

Data will be backed up by transfer to UNSW OneDrive which is pre-approved for the storage of (Highly) Sensitive Data and provides resilient, multiply redundant off-site backup.

Periodic backups will be arranged by the Research Project Manager according to the following minimum schedule:

Dataset/type	Minimum backup frequency
Consent and screening data	At the conclusion of each mini-trial
Participant questionnaires, instrumentation, digital phenotyping data	At the conclusion of each mini-trial
Enrolment data, allocation, algorithm models	At the conclusion of each mini-trial
Analytical products (e.g. SPSS data files, scripts, etc)	At the conclusion of each analysis
Software code	Every six months
Project emails	Every six months
Project files	Every six months

7.2 Retention

Trial data will be retained for 15 years after the conclusion of the study.

Data will be processed for retention (and ultimately disposed of) following the then-current UNSW Research Data Governance & Materials Handling policy/procedures.

8. Data access and handling

8.1 Vibe Up Data Access Principles

The following principles govern the access to and day-to-day handling of data within the Vibe Up Study. All staff are required to read and sign to indicate their understanding of, and agreement to, these principles.

1) Everyone in the project has a responsibility to ensure that data remain protected. If you see anything that concerns you, you should report it promptly to WuYi Zheng (Research Project Manager) or Helen Christensen (Principal Investigator).
2) There is no presumptive right of access to any Vibe Up data. This includes, in particular:

- a. Data that are captured, generated or stored by Qualtrics, the Vibe Up App, the Optimise cloud system and/or any associated monitoring infrastructure; and
- b. Data generated through primary and secondary analyses and business intelligence activities.

3) Access to individual-level (whether identifiable or otherwise) and aggregate data is permissible only where there is a specific, valid purpose.

   In this context, specific and valid means that the purpose:
   - a. Can be clearly stated;
   - b. Is necessary to advance one or more goals of the Vibe Up;
   - c. Is compatible with the specific permissions given by participants during informed consent and with the law;
   - d. Is consistent with the duties defined for your role;
   - e. Cannot reasonably be achieved by another means that either requires a smaller volume or level of detail of data; or does not require access to the data at all;
   - f. Is likely to be successfully achieved.

4) Some data is accessible only to specific team members. Access rights are governed by the **Vibe Up Data Access Rights Matrix** (see **Section 8.2**, below). It is your responsibility to understand what data you can and cannot access under these requirements, and to follow them in performing your role in the study.

5) The storage and exchange of data creates specific risks relating to unauthorized access. A public data breach of participant-level data would not only jeopardise the privacy of the individuals involved but would likely result in the trial being terminated, with significant reputational damage to the Institutes and the Universities more broadly.

6) As a result, there are project-specific guidelines that govern how data should be saved and transferred.
   - a. The **Vibe Up Secure Data Storage Matrix** (see **Section 8.3,** below) lists the ways in which data of different types can be safely saved, as well as methods that must NOT be used.
   - b. The **Vibe Up Secure Data Transfer Matrix** (see **Section 8.4**, below) lists the ways in which data may be safely exchanged with others, including those outside the Institute, as well as methods that must NOT be used.

7) If you use either a laptop or personal device to store any form of Vibe Up data, whether identifiable or otherwise, then password-protected, whole disk encryption must be enabled on that device.

8) Every team member has a responsibility to report any event that might jeopardise or violate these requirements (even if accidental, unintentional or relating to the actions of others.)

   Things that are notifiable include, but are not restricted to:
   - a. The loss of a computer or other device on which Vibe Up data had been stored, regardless of whether that device was protected by encryption.
   - b. Accidentally sending participant-level data to the wrong recipient by email.
   - c. Evidence that participant-level data is being stored on assets without adequate access control, such as the main BDI network drive.

9) It is your responsibility to understand and follow these requirements. If you have any questions or do not understand this in full, please speak to WuYi Zheng.

10) Compliance with these requirements will be subject to periodic audit.

I have read, understand and agree to these principles.

Name (please print)	Name
Signature	

Page 4 of 7

HC Number: HC200466 **Version dated:** 15 Mar 2021

Date: Date

8.2 Data Access Rights Matrix

This rights matrix specifies which data can be accessed by which staff members/groups. A register of group membership is maintained by the Research Project Manager.

Data classification level / User rights group	Sensitive: Participant-level identifiable and/or unblinded data, see **Section 6.3.1**. *e.g. raw data files*	Sensitive: Participant-level contact details, see **Section 6.3.2**. *e.g. register of screened eligible participants*	Sensitive: Participant safety/clinical support data, see **Section** Error! Reference source not found. *e.g. Psychological Safety Response Register*	Sensitive: Deidentified participant-level datasets *e.g. anonymised questionnaire responses*	Private: Aggregate data and analytical products *e.g. study reports, counts data*	Private: Other study files *e.g. protocols, minutes, progress reports, software code*	Public: Content <u>intended</u> for external audiences *e.g. presentations, journal articles*
Vibe Up Data Owner/Steward	✓	✓	✓	✓	✓	✓	✔
BDI/ A^2I^2 Vibe Up Operations team	🔒	✓	✓	✓	✓	✓	✔
BDI/A^2I^2 Other Optimise investigators/staff	🔒	✓	✖	✓	✓	✓	✔
BDI/ A^2I^2 IT staff and contractors	🔒	🔒	✖	🔒	✓	✓	✔
Other Optimise project partners	✖	✖	✖	🔒	✓	✓	✔
Vibe Up DSMC members	🔒	🔒	🔒	🔒	🔒	🔒	✔
External IT contractors	✖	✖	✖	🔒	🔒	🔒	✔
Others	✖	✖	✖	✖	✖	✖	✔

✔ Permitted without restriction.
✓ Permitted for a specific, valid purpose.
🔒 Permitted only with permission of the Vibe Up Data Steward and only for a specific, valid purpose.
✖ Not permitted

8.3 Secure Data Storage Matrix

This matrix specifies what data storage methods are permitted according to the type and sensitivity of the data. If more than one type is combined in a dataset, then the <u>least</u> permissive rule shall apply.

Page 5 of 7
HC Number: HC200466 **Version dated:** 15 Mar 2021

Data Storage Location / Data classification level	Sensitive: Participant-level identifiable and/or unblinded data, see **Section 6.3.1**. *e.g. raw data files*	Sensitive: Participant-level contact details, see **Section 6.3.2**. *e.g. register of screened eligible participants*	Sensitive: Participant safety/clinical support data, see **Section** Error! Reference source not found. *e.g. Psychological Safety Response Register*	Sensitive: Deidentified participant-level datasets *e.g. anonymised questionnaire responses*	Private: Aggregate data and analytical products *e.g. study reports, counts data*	Private: Other study files *e.g. protocols, minutes, progress reports, software code*	Public: Content intended for external audiences *e.g. presentations, journal articles*
UNSW OneDrive for Business	(✓)	(✓)	(✓)	(✓)	✓	✓	✔
Vibe Up Shared Teams Folder	(✓)	(✓)	(✓)	(✓)	✓	✓	✔
Departmental network drive (e.g. BDI J:\ drive)	✗	✗	✗	✗	✗	✗	✔
Personal network drive (e.g. UNSW H:\ drive)	✗	✗	✗	✗	✗	✗	✔
Personal cloud storage	✗	✗	✗	✗	✗	✗	✔
Local hard drive	✗	✗	✗	✗	✗	✗	✔
USB sticks and other removable media	✗	✗	✗	✗	✗	✗	✔
Locations not described here	✗	✗	✗	✗	✗	✗	✔

✔ Permitted without restriction.
✓ Permitted for a specific, valid purpose.
(✓) Permitted for a specific, valid purpose and with access control restricted to named users only.
✗ Not permitted

8.4 Secure Data Transfer Matrix

This matrix specifies what data transfer methods are permitted according to the type and sensitivity of the data. If more than one type is being exchanged in a single package, then the least permissive rule shall apply.

Page 6 of 7
HC Number: HC200466 **Version dated:** 15 Mar 2021

Data classification level	Sensitive	Sensitive	Sensitive	Sensitive	Private	Private	Public
**Data Transfer Method**	Participant-level identifiable and/or unblinded data, see **Section 6.3.1**. *e.g. raw data files*	Participant-level contact details, see **Section 6.3.2**. *e.g. register of screened eligible participants*	Participant safety/clinical support data, see **Section** Error! Reference source not found. *e.g. Psychological Safety Response Register*	Deidentified participant-level datasets *e.g. anonymised questionnaire responses*	Aggregate data and analytical products *e.g. study reports, counts data*	Other study files *e.g. protocols, minutes, progress reports, software code*	Content intended for external audiences *e.g. presentations, journal articles*
UNSW OneDrive for Business file sharing	🔒	🔒	🔒	(✓)	✓	✓	✅
Print outs	✖	🔒	🔒	🔒	✓	✓	✅
Email	✖	✖	🔒	✖	✓	✓	✅
Teams chat	✖	✖	✖	✖	✓	✓	✅
Slack, Trello, Jira, etc.	✖	✖	✖	✖	✓	✓	✅
USB sticks and other removable media	✖	✖	✖	✖	✖	✖	✅
Methods not described here	✖	✖	✖	✖	✖	✖	✅

✅ Permitted without restriction.

✓ Permitted for a specific, valid purpose.

(✓) Permitted for a specific, valid purpose to named individuals only.

🔒 Permitted only with the permission of the Vibe Up Data Steward (for participant-level identifiable data) or a member of the Vibe Up Operations Team (for participant-level contact details or safety/clinical support data.)

✖ Not permitted

Version history

Date	Summary of changes
15 Mar 2021	Document created.

Page 7 of 7

HC Number: HC200466 **Version dated:** 15 Mar 2021

Appendix 24 Data Safety Monitoring Board Terms of Reference and Procedures

Vibe Up Data Safety Monitoring Board Terms of Reference and Procedures

Version dated: 12 Oct 2021

1. Purpose

This document specifies the composition, roles, responsibilities and functions of the Data Safety Monitoring Board (DSMB) for the Vibe Up trial.

The purpose of the DSMB is to safeguard the interests of study participants, assess the safety and efficacy of the Vibe Up interventions, monitor the overall conduct of the trial and provide recommendations about whether to continue, modify or stop the trial.

2. Scope

This document governs the constitution and operation of the DSMB within the Vibe Up study from the commencement of the trial until the final study report is produced.

3. Governing policies and principles

The Vibe Up DSMB will function in accordance with the principles of the following documents: Good Clinical Practice (GCP) Guidelines, Declaration of Helsinki 2000, NHMRC National Statement on Ethical Conduct in Human Research, NHMRC Guidance Safety and Monitoring of Clinical Trials involving a Therapeutic Good, and the University of New South Wales HREC guidelines.

4. Limitation of scope

The sole function of a Data Safety Monitoring Board is to identify based on reported data whether there are grounds to modify or stop the trial on either safety or ethical grounds.

The DSMB does this by reviewing available data, requesting clarification if necessary, and providing written recommendation(s) to the investigator team, which the team may either accept or rebut.

For the purposes of the Vibe Up DSMB, reported data includes standard reports produced by the Data Steward (see **Appendix 1**) plus the details of any adverse events reported to the Board.

The DSMB is not empowered to critique or request changes to the design of the study or its day-to-day conduct except where the Board believes, based on the data it has reviewed, that there are aspects of the study that significantly affect its safety or ethical conduct.

5. Composition

The Vibe Up DSMB will consist of a fixed chair (A/Prof. Fiona Shand) and 2 members (Professor Andrew Mackinnon & Dr Megan Kalucy) one of whom will be the trial biostatistician. The DSMB shall be established before the trial commences.

All DSMB members must:

- Offer relevant expertise in clinical studies, biostatistics and/or digital mental health interventions.
- Have a degree of independence from the Black Dog Institute, University of New South Wales Sydney, and the investigator team, as required by the NHMRC for non-commercial trials. No member of the DSMB should have direct involvement in the day-to-day conduct of the study.
- Be free of any significant or potential conflicts of interest, according to the guidelines set out in the NHMRC National Statement on Ethical Conduct in Human Research. No member should have financial, proprietary, professional, or other interests that may affect impartial, independent decision-making by the DSMB.
- Agree to the confidentiality principles that govern these Terms (see **10 Record keeping and confidentiality**).

6. Roles and responsibilities

6.1 DSMB members and chair

Per **Section 13.3** of the Clinical Trial Protocol, the Vibe Up Data Safety Monitoring Board (DSMB) will be responsible for the following procedures:

- Assessment of all clinical trial safety data, critical efficacy endpoints and trial performance data (see **Appendix 1**) at regular intervals;
- Generating recommendations to the Principal Investigator/Sponsor on whether to continue, modify, or stop a trial; and
- Review of the final study report at the conclusion of the study.

DSMB members are expected to:

- Read and agree to these Terms of Reference;
- Attend meetings of the committee according to an agreed schedule;
- Review aggregate and individual subject data related to safety, efficacy and overall conduct of the trial;
- Identify consensus recommendations to continue, modify or terminate the trial;
- Review the final study report and issue a closeout decision; and
- Comply with conflict of interest and confidentiality guidelines stated in these Terms.

The DSMB Chair is, in addition, expected to:

- Facilitate both open and closed sessions of the DSMB;
- Act as arbiter (and decide if necessary) should there be any disagreement regarding any recommendation being proposed by the Board; and
- Generate a written recommendation detailing with justifications the recommendation of the DSMB and communicate this promptly to the investigators.

6.2 Vibe Up team

In general, the Vibe Up Principal Investigator and their delegates are expected to assure the proper conduct of the study, including the collection of accurate and timely data.

The Vibe Up Research Project Manager (Dr Wu Yi Zheng) will:

- Liaise with members to schedule meetings of the DSMB;
- Attend the open component of each meeting to answer trial management-related questions;
- Promptly report any safety concerns to the DSMB and make the trial **Safety Monitoring Register** available for review by the Board at each scheduled meeting;
- Communicate any recommendation arising from the DSMB to the broader investigator team;
- Ensure that a written response is provided to the DSMB within 20 working days of receipt of any recommendation; and

- Communicate with overseeing authorities (e.g. UNSW HREC) as needed.

The Vibe Up Data Steward (Dr Artur Shvetcov) will:

- Extract, prepare and report the data described in **Appendix 1** to the DSMB in an unblinded format at least 5 working days before scheduled meetings;
- Attend both the open and closed components of each meeting to answer data-related questions; and
- Take minutes in each DSMB meeting on behalf of the Chair.

In addition, a named Vibe Up DSMB Investigator (Dr Jin Han) will be assigned in the Delegations Log and nominated to:

- Attend the open component of each meeting to answer trial-related questions.

Neither the Vibe Up Research Project Manager nor the named Investigator will have access to blinded data.

7. Meetings and quorum

The DSMB will meet after the conclusion of the third and twelfth mini-trials and at study closeout (see **9 Study closeout**). It may also meet as required by its membership.

Each meeting will be split into an open and closed sessions:

- The open session will consider only blinded trial data. It will be attended by the Research Project Manager and a named Investigator to answer any questions relating to trial management or day-to-day conduct.
- The closed session will consider blinded data not accessible to the investigator team.

A quorum of two DMSC members must be present for each closed session and for any recommendation to be issued by the Board.

The trial Data Steward will be available throughout both components to answer data-related questions.

8. DSMB recommendations

8.1 Recommendations

At the conclusion of each meeting, the DSMB is required to provide within 10 working days a written report outlining their recommendation to the Principal Investigator.

The Board may either:

- Request clarification or additional data (see below);
- Recommend to continue the study without modification;
- *Provisionally* recommend to modify the study with reasons (see below); or
- *Provisionally* recommend to stop the study with reasons (see below).

8.2 Requests for additional data

Requests for additional data can concern any research or trial administrative data sources/types specified in the Clinical Trial Protocol including unblinded aggregate data; Case Report Forms for specific participants (e.g. those experiencing safety outcomes or adverse events), and data relating to adverse events such as safety monitoring reports. However, the DSMB cannot request new data to be collected.

8.3 Recommendations to modify or stop the study

Modification or cessation of a trial is a significant event. Such recommendations are therefore provisional until the investigator team has been able to review and respond. In addition, when making such a recommendation, the DSMB is required, to articulate in its written report:

1. The reasoning behind its concerns, based on the data it has reviewed.
2. Justification as to why the concern identified are sufficiently serious in terms of either safety or ethical impact to necessitate changes to the study. Justification may be on the basis of either (or both) of:
   - Severity: the risk presented to participants, even if rare, is sufficiently severe that even small numbers experiencing this outcome in the future would be unacceptable.
   - Likelihood: the risk occurs sufficiently often that the aggregate burden/effect on participants (or other aspects of the study, such as the viability of analysis) is unacceptable.

8.4 Investigator response and final decision

The Vibe Up investigators will review and respond to the DSMB recommendation. If the recommendation requests action, the investigators will provide, within 20 working days, a written response stating whether the recommendation will be followed and the plan for addressing any issues raised.

The DSMB must then consider the investigators' response and issue a final decision stating whether the recommendation is sustained and, if so, whether the actions proposed by the investigator team are satisfactory.

If required, the Research Project Manager will disseminate the final decision and any action taken to the appropriate regulatory agencies within an appropriate timeframe.

9. Study closeout

At the conclusion of the trial, the DSMB will review the final study report.

The DSMB is required to issue a closeout decision stating whether the report accurately reflects the data they have reviewed over the course of the study and any recommendation(s) that have been issued by the Board.

10. Record keeping and confidentiality

The Data Steward will be responsible for keeping complete and accurate minutes of all DSMB meetings.

All data provided to the DSMB will be considered privileged and confidential. The DSMB agrees to use these data to accomplish the responsibilities of the DSMB and will not use it for any other purpose without written consent from the Principal Investigator.

All DSMB documentation will be retained for a period of 15 years after completion of the trial. Access to archived data will be controlled by the Sponsor who will release information only as specified in these Terms of Reference or as required by law.

Signatures

Chair's signature

I agree to these Terms of Reference and Procedures.

Name (please print) Fiona Shand

Signature	
Date	25/10/2021

Member's signature

I agree to these Terms of Reference and Procedures.

Name (please print)	Andrew Mackinnon
Signature	
Date	Date 27/10/2021

Member's signature

I agree to these Terms of Reference and Procedures.

Name (please print)	Megan Kalucy
Signature	
Date	Date 26 Oct 2021

Version history

Date	Summary of changes
09 Mar 2021	Document created.
12 Oct 2021	Updated name of Data Steward and Vibe Up DSMB Investigator

Appendix 1 – Data to be routinely supplied to DSMB

The following data will be reported to the DSMB in aggregate form (counts, proportions by arm, by mini-trial):

1. **Safety parameters** defined in **Section 7.6.2** (Safety parameters) of the Clinical Trial Protocol:

Id	Self-report questionnaire	Signal
DASS	Depression, Anxiety and Stress Scale, 21-item version	Increase (deterioration) in DASS-21 total score comparing pre- and post- intervention for each arm.
EXP	Within-study exposures questionnaire	Participant-reported negative mental health event potentially attributable to intervention exposure reported at post-intervention.
UX	UX questionnaire	Participant-reported experiences using the Vibe Up interventions (and app more generally) that may include safety-relevant information. See also compliance-related data, below.

2. **Compliance-related data** defined in **Section 6.4** (Procedures for monitoring subject compliance) of the Clinical Trial Protocol:

Id	Self-report questionnaire	Signal
LOG	Daily log of engagement with intervention	Poor (differential) compliance between study arms.
UX	User experience (UX) questionnaire (questions UX4a and UX4b)	Significant technical challenges interfering with the conduct of the study.

Plus data concerning completion of/attrition from key components of the study, e.g. between recruitment/study start, for each questionnaire, etc.

3. **The Trial Safety Monitoring Register**

The purpose and function of the register and the procedures for managing adverse events are described in **Section 7.6.4** (Procedures for eliciting, recording and assessing adverse events) of the Clinical Trial Protocol.

Appendix 25 Vibe Up Algorithm Specification

Vibe Up Algorithm Specification

Version dated: 18 Nov 2021

1. Purpose

The purpose of this document is to provide information on the Algorithm that is integrated into the 'Vibe Up' study.

2. Scope

The algorithm applied in this study is a Multi-Arm Bandit (MAB) algorithm with contextual information. The MAB is a sample efficient method of determining the best possible treatment to prescribe to a participant. In contextual MABs, a participant's characteristics (e.g. age, gender, weight etc.) – referred to as their *features* – are used to incorporate more information about an individual in the selection of the treatment recommendation [1].

An overview of the algorithmic framework in the Vibe Up study, and the information flow to and from the participant is illustrated in Figure 1.

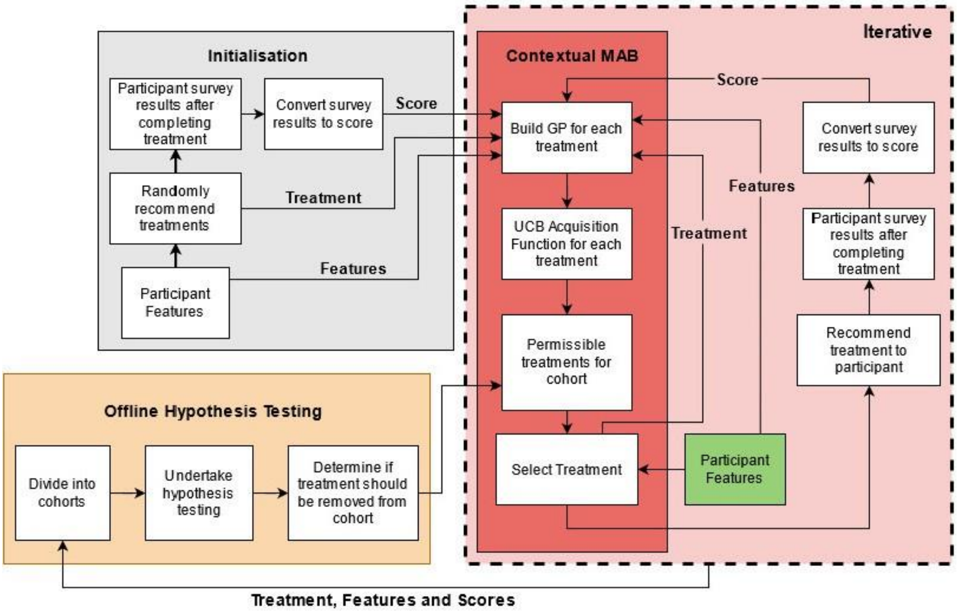

Figure 1: Contextual MAB Framework – When starting a trial, initial group of participants are randomly assigned to treatments. The survey results of these treatments are converted to a score which is fed into the Contextual MAB algorithm. The Contextual MAB provides recommended treatments based on participant features. Intermittently, offline hypothesis testing is conducted to determine the significance of each treatment within cohort, with any changes influencing the available treatments in further trials of the study.

At a high level, the framework is initialised with the first set of participants. During the onboarding period, participants are required to complete a **baseline** DASS survey. In the initial phase of the Vibe Up study, these

Page 1 of 7

HC Number: HC200466 **Version dated:** 18 Nov 2021

baseline survey results will be normalised and used to represent a participant's features. Participants will also be categorized into various cohorts based on these baseline survey results, with these cohorts reflecting the severity of their mental health. Further information on this normalisation procedure and cohort allocation is described below.

As there is no prior information about each of the treatment options, participants are randomly recommended to an intervention to try for the first treatment period. Prior to beginning their treatment period, participants undertake another **mid-point** DASS survey (mid-point survey). At the end of the intervention period, the participant completes a **post** survey. The responses of these surveys are converted into a normalised total DASS score. A comparison between the results of the mid-point total DASS score and the post total DASS score (after treatment) is performed, and a single numerical *score* is derived to describe the change in survey results – this is to reflect the impact of the treatment. This information – the score, treatment, and participant features – are used to initialise the Contextual MAB algorithm. The Contextual MAB (discussed in more detail below), is used to recommend the best treatment for the next round of participants, based on their individual features. The overall goal of the algorithm is to recommend treatments that will ultimately lead to the greatest reduction in the post DASS score compared to the mid-point DASS score (indicating a treatment had a positive impact on a participant). The treatments to be selected from, for this next round of participants, is based on the cohort. The outcome of this recommendation is fed back to the Contextual MAB and the process is repeated.

Throughout the experimental period, there will be intermittent hypothesis tests using the results obtained so far to examine whether any treatments exhibit a within-cohort significant effect on a participant's score. These tests will be conducted for each cohort of participants, and the treatments available to them. Once a particular treatment is deemed significant within that cohort, it will be removed from the list of available treatments for that cohort.

3. Normalised total DASS survey scores

Once a participant has completed their DASS survey, these results are converted to a single feature value according to the formula below:

Normalised total DASS score:

$$S = \left(\frac{DASS_{depression} - 6.35}{6.85} + \frac{DASS_{anxiety} - 4.77}{4.79} + \frac{DASS_{stress} - 11.19}{8.25} \right) \div 3$$

Where $DASS_{depression}$, $DASS_{anxiety}$, and $DASS_{stress}$ are a participant's cumulative depression, anxiety and stress scores from the DASS survey. These values are normalised and averaged to calculate the normalised total DASS score [2].

This normalisation is done to DASS scores at baseline, mid-point and post. The baseline normalised total DASS score will go on to represent a participants context feature, whilst the difference in the mid-point and post normalised total DASS score will represent a participants score for the completed treatment.

4. Cohorts

The baseline normalised total DASS score is further used to categorise a participant as either mild, moderate, or severe according to the following metric:

Mild: $S < 1$

Moderate: $1 \leq S < 2$

Severe: $S \geq 2$

5. Contextual MAB

The contextual MAB works by building a probabilistic model for each of the treatments using the participants features and scores. A commonly used probabilistic model is a Gaussian Process (GP) which is fully defined by a mean and covariance function [3]. The smoothness of the model is defined through the covariance function i.e. how varied output values are for nearby points. As the features (inputs to the GP) are all real-valued numbers (e.g. DASS scores), a Squared Exponential (Gaussian) covariance (= kernel) function is used.

Once fitted with points from participants who have completed the treatment, the GP builds a probabilistic model which returns a prediction (mean) and confidence (standard deviation) for what score could be expected from a participant who has not conducted the treatment, based on their features.

The mean and standard deviation, with respect to the features, from each GP is then used to construct individual acquisition functions for each treatment. In the case of this study, the Upper Confidence Bound (UCB) acquisition function [4] was used. The UCB acquisition function balances the trade-off between acting exploitatively (recommending treatments which are known to produce good scores given the participants features) and exploratively (recommending treatments which have not been tested for the participant's features and have a high level of uncertainty). For each participant in the next round, the value of each treatment UCB function value is determined based on the participant's features. The treatment with the highest UCB function value is recommended to the participant in the next iteration.

Participants go through the recommended treatment and return a completed survey after the treatment period. The survey results are converted to a score, and once again the score, together with the participant's features and the recommended treatment are fed back to the GP and the process is repeated.

Figure 2 illustrates more detail into the Contextual MAB algorithm. In this diagram, for participant 1, treatment 1 returned the highest UCB function value whilst for participant 2, treatment 2 returned the highest value. In the following iteration, scores from these participants, after they have completed the recommended treatment, is integrated back into the GP model and the GP is refitted.

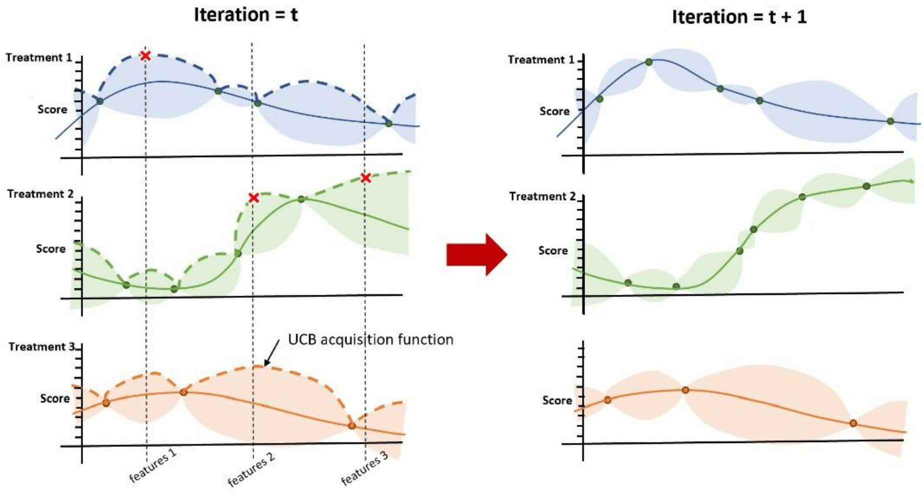

Figure 2: An iteration of the Contextual MAB algorithm. In iteration = t, participant scores are fit to the individual GP's. The UCB acquisition function is derived for each treatment GP (dashed line) using the mean (solid line) and the standard deviation (shaded area). For each participant, their features (feature 1 = features for participant 1) are used to select the treatment for which the UCB

function, at that feature value, returns the highest value (marked by red cross). These treatments are then recommended to the participant and the resulting scores are used to update the GP's as shown in iteration = t + 1

6. Intermittent Hypothesis Testing

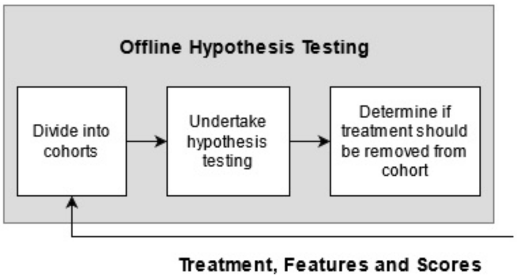

Figure 3: High level schematic of offline hypothesis testing. The full data from the experiment gathered so far is divided into cohorts. Of the treatments that are currently active, hypothesis testing is conducted between them to determine whether a treatment within the cohort should be removed. This process is done for each cohort.

Background

Intermittently during the experimental period, hypothesis testing will be conducted offline using the full available data to determine whether a particular treatment has resulted in a significantly greater improvement between the mid and post DASS scores, compared to other available treatments, within each cohort. The aim of this process is to determine, for a given cohort, which treatment is most effective with statistical confidence. To capture the intention to treat, missing data from participants who were unsuccessful in completing the trial will also be included in this analysis.

Once a treatment has been found to be significant, it will be removed from the list of treatments available for recommendation, for that cohort, in proceeding mini-trials. This allows the optimiser to focus its allocations in proceeding mini-trials to the remainder of the treatments. The intention being that, at a future hypothesis testing time point, comparison can be done to find the next most effective treatment (and the process is repeated). Figure 3 illustrates this process at a high level.

During the trial period, hypothesis testing will be conducted in order to determine, within each cohort, the order of effectiveness of the treatments. I.e. For each cohort, determine which treatment was most effective in improving the cohort's DASS score, then the second and third most effective.

Though attempts will be made to rank the effectiveness of the treatments (within cohort), there is no guarantee that a full rank will be distinguished by the end of the trial period.

As there are 4 treatments available for each cohort, 3 intermittent tests will be conducted during the experimentation period. This will give the best chance to determine the first, second and third most effective treatment (within cohort).

Additionally, due to the inflation of Type 1 errors that arise from the increased number of tests (both across time and across treatments), the number of intermittent tests is limited to 3. This also imposes stricter requirements on the p-values to claim significance.

Timing

Across the experimentation period, hypothesis testing will be conducted after approximately 33%, 66% and 100% of the study period is completed.

Missing Data

To reduce bias in the assessment of a treatment's effectiveness, intention to treat based analysis of the data will be conducted [7]. For this, all data from participants who had been recommended a treatment by the optimiser (following their completed onboarding DASS survey), who had also completed their mid-DASS survey but failed to complete the post-DASS survey, are included in the analysis.

To accommodate the results of participants with incomplete data, 'last observation carried forward' (LOCF) is avoided as this has been shown to produce potentially biased estimates. Instead, an observation-wise ANOVA-type model of group by time effects will be fitted (mixed-model repeated measures), allowing the variance of observations to vary between occasions of measurement (mid-DASS and post-DASS time points) and the residuals of individuals to correlate freely over occasions [8]. This approach assumes that missing assessments are missing at random, an assumption that allows missingness to be dependent on observed information (intervention assignment and previous scores) but not on the unobserved values themselves.

As our analysis would be conducted separately for each cohort, the amount of data points will be divided and may lead to a small number of samples for a particular treatment within a cohort's analysis. To accommodate for this, an adjustment to the degrees of freedom of the mixed-model would be applied as per the Satterthwaite method [9].

Controlling for Type 1 errors

Comparison of multiple treatments, at multiple time points, can lead to an inflation in Type 1 (false positive) errors, which needs to be corrected for.

Controlling for Type 1 errors from Sequential hypothesis testing

To control for Type 1 errors that arise from sequential analysis, an alpha spending function is applied to distribute the Type 1 error across the 3 intermittent hypothesis tests. Various spending functions exist such as Pocock, O'Brien-Fleming, Demets & Lan [10].

In our study, we will conduct the first hypothesis test at about 33% of the way during the experiment period (after about mini-trial 3 or 4), the second at about 66% of the way (after mini-trial 7 or 8) and the third after the final mini-trial (100% of the way through). The information fraction for the alpha spending function is the fraction of participants' data so far, compared to the expected total number of participants over the experimental period.

Our study is amendable to an extension by a few mini-trials (if extra power is needed). Appropriate adjustments to the critical alpha spending p-value will be made to ensure the cumulative Type 1 error is maintained at 0.05.

Controlling for Type 1 errors from multiple hypothesis tests

To control the increased Type-I errors due to multiple hypothesis tests, we apply the Benjamini-Hochberg Correction in the critical p-values [5]. This means that the alpha spending p-value is adjusted for each of the multiple tests. For example, in the first intermittent test, there will be 4 treatments to compare for each cohort. This totals 6 comparisons (done via t-tests). As such the alpha spending p-value for this test is adjusted according to the Benjamini-Hochberg method.

Testing for significance

At each intermittent testing point, a multiple hypothesis test is conducted (separately for each cohort) to determine whether, for the currently active treatments, any of them is significantly better at improving the DASS score.

For a treatment to be removed from the list of available treatments for the cohort (and be deemed the most effective treatment option), it must emerge significantly better (one-sided test) in pairwise comparisons between it and every other active treatment, within the cohort. Significance is specified as returning a p-value less than the critical alpha spending p-value (with the Benjamini-Hochberg p-value adjustment).

Let:

n – number of active treatments for the cohort

$k = \frac{n(n-1)}{2}$ – number of comparisons to conduct

α – alpha spending p-value for the current intermittent test

To undertake the test, the following steps are taken:

1. For each pair of treatments:
    a. Conduct a one-sided Welch t-test (with Satterthwaite adjusted degrees of freedom)
    b. Note the p-value of the test
2. Sort each test according to p-value and apply a Benjamini-Hochberg correction to the α value to determine the critical value for each test
3. Determine which of the tests produced significant results (p-values less than the adjusted α value).
4. For each active treatment, test whether that treatment was significant in all comparisons with other active treatments
    a. If treatment is significant in all comparisons: **Remove treatment from active treatments in future mini-trials. This treatment is the most significant treatment for available treatments.**
    b. If treatment is not significant in all comparisons: Treatment remains available for recommendation by optimiser in future mini-trials.

Communication Protocol

During the Vibe Up study, after each intermittent hypothesis test, one week is permitted for the offline hypothesis testing to be conducted and changes to the active treatment state file (***treatment_status.txt)*** to be updated and placed back into the Conductor platform.

If a change is required to the active treatment state file, a notification with the updated treatment_status.txt file will be sent to A/Prof. Sunil Gupta who will forward this to Prof. Raj Vasa. Prof. Vasa will then notify his team to replace the file for future mini-trial optimiser recommendations.

Contingency Plan

The primary controller of the offline hypothesis testing is Ms Manisha Senadeera. If she is unable to conduct the offline tests, this responsibility will fall upon Stephan Jacobs and Julian Berk, both of whom are made aware of the protocol.

7. Data Checking

After each mini-trial, data checking will be conducted online to monitor and verify the behavior of the Optimiser. These include confirmation of the data preprocessing, general trends in treatment performance and verification of appropriate treatment allocations.

Version history

Date	Summary of changes
01 Dec 2020	Document created.
18 Oct 2021	Document revised.
18 Nov 2021	Document revised (section 6).

References

[1] Lu, T., Pál, D., & Pál, M. (2010, March). Contextual multi-armed bandits. In *Proceedings of the Thirteenth international conference on Artificial Intelligence and Statistics* (pp. 485-492). JMLR Workshop and Conference Proceedings.

[2] Lovibond, S.H. and P.F. Lovibond, Manual for the depression anxiety stress scales (2nd edition). 1995, Psychology Foundation: Sydney, NSW, Australia

[3] Brochu, E., Cora, V. M. & Freitas, N. d. (2010) A Tutorial on Bayesian Optimization of Expensive Cost Functions, with Application to Active User Modeling and Hierarchical Reinforcement Learning. *CoRR* **abs/1012.2599**.

[4] Srinivas, N., Krause, A., Kakade, S. & Seeger, M. Gaussian Process Optimization in the Bandit

[5] Chen, S. Y., Feng, Z., & Yi, X. (2017). A general introduction to adjustment for multiple comparisons. *Journal of thoracic disease*, *9*(6), 1725–1729.

[6] Albers, C. (2019). The problem with unadjusted multiple and sequential statistical testing. *Nat Commun* **10,** 1921.

[7] McCoy C. E. (2017). Understanding the Intention-to-treat Principle in Randomized Controlled Trials. The western journal of emergency medicine, 18(6), 1075–1078. https://doi.org/10.5811/westjem.2017.8.35985

[8] Carpenter, J. R., & Kenward, M. G. (2007). Missing data in randomised controlled trials — a practical guide. London, UK: Medical Statistics Unit, London School of Hygiene & Tropical Medicine

[9] Satterthwaite, F.E. (1946) An Approximate Distribution of Estimates of Variance Components. Biometrics Bulletin, 2, 110-114.
http://dx.doi.org/10.2307/3002019

[10] Albers, C. (2019). The problem with unadjusted multiple and sequential statistical testing. Nat Commun 10, 1921.

Appendix 26 Optimise UNSW System Classification

System Classification_Optimise

**System Name**	Optimise		
**Data Executive**	Helen Christensen (Director, Chief Scientist, Black Dog Institute)		
**Data Owner**	WuYi Zheng (Research Project Manager, MRFAI Optimise Project)		
**System Owner**	Black Dog Institute, Deakin University Applied Artificial Intelligence Institute (A2I2) 50:50		
**Service Provider**	Deakin University Applied Artificial Intelligence Institute (A2I2)	**Data Type**	**Data Type (Please specify in case of Other)**
**Data Classification**	Sensitive	Identifiable Human Subject Research	NB The system will not be used to collect medical data.
	To what populations does the data pertain? (Example: Student, Faculty, Staff, Research Subjects, Patients, Affiliates, Public, etc.)	Research subjects (Healthy adults inclu	
	What population will be consuming this data? (Examples: Student, Faculty, Staff, Research, Vendors, Affiliates, Public)	Researchers	
	What population will be providing data for this software/service? (Examples: Student, Faculty, Staff, Research, Vendors, Affiliates, Public)	Research subjects (Healthy adults inclu	

Standard	Question	Response<br>*(Select Compliant, Non-Compliant)*	Comments
**1. Data Security**			
1.1	Do you use cryptography in data encryption, hashing, and/or masking?	Compliant	Public key cytography-based transport (HTTPS) and at-rest (Google Cloud Storage) encryption.
1.2	Are data backups encrypted?	Compliant	Data will be regularly transferred to UNSW OneDrive for long-term storage, which includes UNSW-compliant standards of encryption by default. (We will leverage backup/DR/retention support offered by this service, per schedule specified in **Data Management Plan**, currently
1.3	Does the database support encryption of specified data elements in storage?	Compliant	Google Cloud Storage buckets (used for data retention) provide default layers of encryption. See: https://cloud.google.com/storage/docs/encryption/default-keys
1.4	Is data encrypted in storage (e.g. disk encryption, at-rest)?	Compliant	All data is stored via the Google Cloud Platform. Default encryption is provided by this platform on all data. Refer to https://cloud.google.com/security/encryption-at-rest/default-encryption
1.5	Do you currently use encryption in your database(s)?	Compliant	Selected entities, e.g. those containing PID are encrypted. Passwords are stored as hashes.
1.6	Do you perform ongoing vulnerability identification activities such as vulnerability scanning, configuration reviews, and penetration testing?	Compliant	All data is stored via the Google Cloud Platform. Regular vulnerability scanning is performed. See: https://cloud.google.com/container-registry/docs/vulnerability-scanning
1.7	Do you deploy adequate technologies such as Anti-Virus (AV), Intrusion Defence Systems (IDS)/Intrusion Prevention Systems (IPS), email filtering, to prevent malicious code from infiltrating the environment?	Compliant	Google Cloud Platform is used for orchestrating the service. Google is responsible for controlling access, logging and monitoring of the systems and infrastructure underlying cloud platform architecture, and thus, our system. See: https://cloud.google.com/files/GCP_Client_Facing_Responsibility_Matrix_PCI_2018.pdf

1.8	Do you monitor information security controls and re-assess them in light of changing threats?	Non-Compliant	Discussions underway between Deakin eSolutions and Google to implement project level settings for information security controls (currently it is organisation level.)
1.9	Is data encrypted in transport?	Compliant	All transport is over HTTPS.
1.10	Do you have systems and processes for analysing security event log data to identify suspected and detected breaches?	Non-Compliant	**Monitoring, Incident Identification and Response Protocol** is being prepared for review/approval by the Project Executive. Responsibility for implementation will sit with A2I2.
1.11	Are there procedures in place for service providers to inform UNSW about incidents and the effectiveness of security controls?	Non-Compliant	Will be specified by **Monitoring, Incident Identification and Response Protocol**, see above.
1.12	Are operating system patches deployed and up-to-date, and is an Anti-Virus (AV) product installed with up-to-date signatures?	Compliant	Google Cloud Platform is used to orchestrate the service. Google is responsible for maintenance/updates to underlying infrastructure (see: https://cloud.google.com/files/GCP_Client_Facing_Responsibility_Matrix_PCI_2018.pdf) Optimise architecture is containerised. Individual containers and application code leveraging SDKs/frameworks will be updated with best effort but may not always be up to date if patches introduce breaking changes. Risk is managed by ensuring that: 1) no container is public facing unless necessary and 2) any public facing container (e.g. those hosting ingest endpoints) have write-only access to sensitive data.
1.13	Do you use an intrusion prevention system and, if so, do you formally review its policy at least annually?	Non-Compliant	Google provides intrusion prevention at the stack level, however application-level intrusion prevention is a client concern. An external Cyber Security provider will be engaged to review the system periodically according to a timeline specified in the Incident Identification and Response Protocol.
1.14	Do you use security controls to protect security event logs from unauthorised modification or deletion?	Compliant	Google Cloud Stackdriver audit logs cannot be modified/delete by users. See: https://cloud.google.com/logging/docs/audit/#audit_log_retention
**2. Data Residency**			
2.1	Does your system store data in Australia? (Please specify the location in comment section)	Compliant	All selected Google Cloud services use servers based on Australia (Zone: australia-southeast1-a, Sydney).
2.2	Does the data centre of your system operate inside of the Australia?	Compliant	See above.
2.3	Will any institution data leave the Australia? (if yes please select Non-Compliant)	Compliant	Data remains within Australia. The only possible reason for data to be outside of Australia is if a participant is overseas (i.e., the source of participant data). However, once that data is securely transported to our servers, it will remain in Australia. (Study protocols will exclude participants at screening who declare that they are resident outside Australia.)
2.4	Are data backups stored for more than 6 months?	Compliant	Data will be regularly transferred to UNSW OneDrive for long-term storage, which includes UNSW-compliant standards of encryption by default. (We will leverage backup/DR/retention support offered by this service, per schedule specified in **Data Management Plan**, currently

2.5	Are backup copies made according to pre-defined schedules and securely stored and protected?	Compliant	Backup to UNSW One Drive will be per regular schedule specified in **Data Management Plan**, currently under development.
**3. Data Access**			
3.1	Does your system use multi-factor authentication for administrators? (e.g. OTP, SMS or App based etc.)	Compliant	Google Cloud access is via Deakin University SSO which mandates 2FA.
3.2	Does your system use multi-factor authentication for users? (e.g. OTP, SMS or App based etc.)	Not Applicable	System does not currently support the concept of 'user' in relation to data access.
3.3	Do you ensure remote connection to your system is approved and provided via Virtual Private Network (VPN), and remote privileged access uses two-factor authentication?	Non-Compliant	Data Owner accountable for approving and auditing users granted acess to the system and their roles, per procedures specified in Data Management Plan, currently under development. 2FA is as above for all users. VPN based tunnelling to Google Cloud is not a planned feature (HTTPS-based transport encryption used instead.)
3.4	Do you implement secure account management based on zIDs, with defined parameters for invalid password attempts, lockout duration, screen lockout, etc aligned to UNSW security standard?	Compliant	Deakin University SSO used for authentication defines password complexity, reset and lockout processes. Gcloud does not support screen lockout (see 5.7). Deakin procedures here: IT H Reset your forgotten Deakin Staff password.p
3.5	Do you establish access controls so that system data can't be modified?	Compliant	Role-based security used to define administrator/user rights and limitations.
3.6	Do backups containing the UNSW's data ever leave the Australia either physically or via network routing?	Compliant	Planned manual backup to UNSW OneDrive. Data stored on UNSW OneDrive will not leave Australia.
**4. Data Processing**			
4.1	At the completion of system's contract, will data be returned to the UNSW?	Compliant	Data ownership is shared between UNSW and Deakin University (per signed partner agreement prepared/approved by RGO for MRFAI000028). On termination of service each entity will maintain a copy of the data.
4.2	Is the service hosted in a high availability environment?	Compliant	All services are hosted using Google Kubernetes Engine (GKE) which guarantees a highly available environment.
4.3	Are audit logs available that include at least all of the following; login, logout, actions performed, and	Compliant	Google Cloud Stackdriver audit logs.
4.4	Are ownership rights to all data, inputs, outputs, and metadata retained by the UNSW?	Compliant	Data ownership is shared between UNSW and Deakin University (per signed partner agreement prepared/approved by RGO for MRFAI000028). On termination of service each entity will maintain a copy of the data.
4.5	Do you log data relating to activity and security events?	Compliant	Yes, activity and security audit data are logged automatically by Google Cloud.
**5. Data Management**			

5.1	Does your system have a Disaster Recovery Plan (DRP)?	Not Applicable	Backup/DR not a design requirement of this prototype research system. Data will be regularly transferred to UNSW OneDrive for long-term storage (and leverage backup/DR procedures of this service, per schedule specified in **Data Management Plan**, currently under development.) Codebase will be stored in the Deakin University Applied Artificial Intelligence Insitute (A2I2) private repositories on GitHub corporate plan (and leverage backup/DR procedures of this service.) Named UNSW/BDI staff will be granted acess to this codebase.
5.2	Does system administrator review the access controls in every 6 months?	Non-Compliant	Will be specified by **Monitoring, Incident Identification and Response Protocol**, currently under development.
5.3	Do you document the ownership and technical purpose of system accounts, and maintain that	Non-Compliant	As above.
5.4	Does system administrator review data leakage in every 6 months?	Non-Compliant	Protocol/procedures will be specified by **Data Management Plan**, currently under development.
5.5	Do you make users aware of the need to protect their portable hardware and storage devices with respect to this system?	Non-Compliant	User responsiblities (and communications to users on this topic) will be specified by **Data Management Plan**, currently under development. We expect to require that all users accessing the system/data will have whole disk encryption on any portable/storage device.
5.6	On termination of service, is UNSW data returned or transferred securely to UNSW, and removed from the service provider's systems and media?	Compliant	Yes, data will be transferred securely to UNSW/Deakin per the shared ownership agreement and removed completely from Google Cloud.
5.7	Does your system have automatic locking/logout and password protection of systems after 15 minutes	Non-Compliant	Not supported by Google Cloud.
**6. Data Retention, Backup & Disposal**			
6.1	Do you store security logs for more than 1 year?	Compliant	Google Cloud retention of security events exceeds one year. See: https://cloud.google.com/logging/quotas#logs_retention_periods
6.2	Does system trigger notifications in case of backup job fails?	Not Applicable	Manual backup procedure to UNSW OneDrive.
6.3	Do procedures exist to ensure that retention and destruction of data meets established regulatory requirements?	Compliant	Planned regular backup to UNSW OneDrive ensures compliance with retention policies. Data destruction will be managed via procedures specified in **Data Management Plan**, currently under development.
6.4	Does system have data backup mechanisms? (e.g. full, incremental)	Compliant	Full manual backup will be performed on a scheduled basis per **Data Management Plan**, currently under development.
6.5	Can system be successfully entirely restored from backup media?	Not Applicable	DR not a design requirement of this prototype research system. Data will be regularly transferred to UNSW OneDrive for long-term storage (and leverage backup/DR procedures of this service, per schedule specified in **Data Management Plan**, currently under development.)
6.6	Do you or system administrator secure backup media in multiple geographic locations?	Compliant	UNSW OneDrive provides distributed mirroring by default. See: https://docs.microsoft.com/en-us/compliance/assurance/assurance-data-resiliency-
6.7	Can the institution extract a full backup of data?	Compliant	Full manual backup will be performed on a scheduled basis per Data Management Plan, currently under development. This means that all data can be extracted/recovered per the last backup.

Appendix 27 Optimise UNSW System Classification Approval

Kit Huckvale

From: David Jung
Sent: Monday, 14 December 2020 5:14 PM
To: Kit Huckvale
Subject: Re: SCT Review Request for Optimise (previously 'Instil')

Dear Kit,

Amazing – looks great. I'll keep this copy as the final version.

I can confirm that you have completed the UNSW System Classification Tool. As per the requirements of the UNSW Data Handling Guidelines and based on the information you have provided in this Tool, the system, Optimise, is deemed suitable for the Sensitive classification of data.

Hope the rest of the ethics application process goes well.

Best regards,
David
--
Dr. David S.R. Jung – PhD, BSci (Hons)
Research Data Support Officer

UNSW Research Infrastructure
Research Technology Services
UNSW Sydney NSW 2052 Australia
P: (02) 9065 3736
E: david.jung@unsw.edu.au
W: research.unsw.edu.au
CRICOS Provider Code 00098G

1

2394
Appendix 28 Vibe Up App Software Requirements
2395

Vibe Up Software Requirements

The purpose of this requirements document is to describe the requirements that the Conductor platform has to meet in order to conduct the Vibe Up study on the platform.

Table of Contents

Table of Contents 1
Glossary 2
Motivation 3
Study Design 3
  User Flow 4
  Mini-Trial Orchestration 4
  Web Enrollment 5
  Experiment Lifecycle 6
Key Components 11
  Mobile app 11
  Infrastructure 12
  Backlog 12
  Survey/Questionnaire Codes 13
Key Dates 14
Roadmap 19
Appendix A - Web Based Enrollment Requirements 24
Appendix B - Internal Test Users 26

Glossary

Participant Candidate - A user that clicks on the URL in the study advertisement and enrols online, accepting the Plain Language Statement (PLS; Deakin) / Participant Information Statement (PIS; BDI), Informed Consent, is eligible to participate and completes the relevant battery of onboarding questionnaires and surveys.

Participant - A Participant Candidate that has received their SMS to download the app, and has downloaded, installed, and completed the SMS verification process to start Active and Passive data collection for the experiment.

Mini-Trial – A highly structured, repeatable sub-experiment within the trial that is built around the end-to-end installation/use of the Vibe Up App by a sample of Participant Candidates over a period of exactly 29 days. Multiple mini-trials are chained together to build the trial.

Mobile App - A mobile app that uses the existing platform SDK to capture user data and display interventions to participants.

Web Enrolment App - A web front end that prospective participants land on to read the PLS/PIS, provide Informed Consent, undergo Eligibility Screening, provide Registration Details, and complete their Onboarding Survey. Out of scope in these requirements.

Infrastructure Compliance - The infrastructure must largely comply with the UNSW data and governance compliance spreadsheet.

Plain Language Statement (Deakin) / Participant Information Statement (BDI) - A document (typically created through MS Word) that describes the experiment, data collected and participation expectations in plain language to be read by prospective participants to inform their decision on whether or not to participate in the study.

Informed Consent Form - A form that confirms a participant candidate's willingness to take part in the study, their receipt of appropriate study information and opt-in/out of any optional study components. Providing informed written consent transitions a person from prospective Participant Candidate into a Participant.

Eligibility Screening Survey - A web-based survey composed of numerical input, multiple choice and open-ended questions (free text) that automatically determines if a Participant Candidate is eligible to take part in the study.

Registration Details - A form that captures at minimum and with appropriate validation to minimise the risk of data-entry errors, the participant's preferred name, mobile phone number and email address.

Onboarding Survey - A survey conducted after the consent form, eligibility screening survey, and registration process, in that order, to gather data about the participant candidate.

Ecological Momentary Assessment (EMA) Survey – A brief survey intended to capture 'in the moment' experience of daily life. To be fired twice a day and open throughout the Baseline phase for Participants to complete, with a similar schedule for the Active Control group in the Intervention Exposure Phase.

Last revised: 01 Mar 2021, Rena

Page 2 of 26

Motivation

This clinical trial has been designed to help university students experiencing psychological distress by offering one of three very brief (2-week) self-guided digital interventions targeting either sleep, mindfulness or exercise.

The platform will need to enable a university student to join and participate in the study anonymously (to the researcher) with their smartphone. It will need to support an engaging experience for participants, recognising that non-participation attrition is a significant risk in this target population that jeopardises the viability of the Mini-Trial approach.

It will need to support the process of response-adaptive random allocation to an intervention based on output of the optimisation algorithm maintained by the algorithm team. It will need to preserve blinding so that researchers conducting the analysis remain unaware of the specific intervention offered to each participant until stopping criteria are met and analysis is complete.

Study Design

The definitive account of the study design is provided by the Vibe Up Clinical Trial Protocol.

The study design encapsulates multiple components and consists of multiple overlapping mini-trials. The condition to cease mini-trials is contingent on reaching sufficient sample size and effect, determined by the research team.

There are three components to the facilitation of the experiment. A web-based enrolment component that involves Participant Candidates, depicted in Figure 1, a mobile app component facilitating the experiment for Participants, depicted in Figure 2, and a Mini-trial Orchestrator, encapsulating the web enrolment and mobile app lifecycles, depicted in Figure 3.

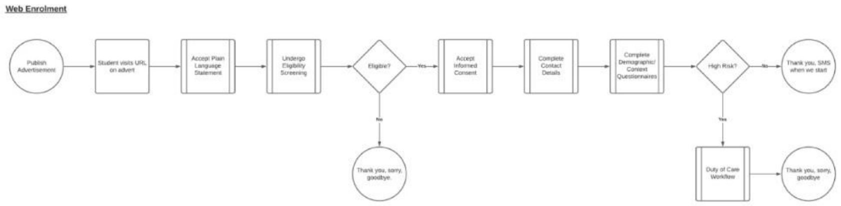

Figure 1: The web enrolment flow for Participant Candidates (link here)

User Flow

Within the broader study design, every user follows a pre-planned, stereotyped flow that ensures key study tasks, such as consent, are completed, required data are collected and interventions are delivered as planned. The broad flow is depicted in Figure 2, while the complete end-to-end journey is described by the User Flow Diagram: Figma Link.

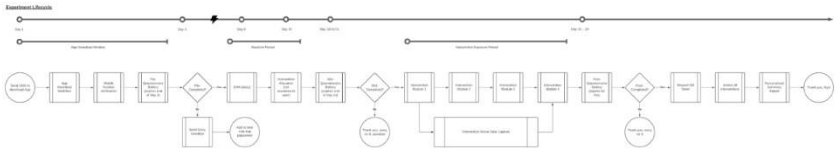

Figure 2: The app experiment lifecycle for Participants (link here)

Mini-Trial Orchestration

Multiple Mini-Trials will be conducted within this study, each starting and ending on a Tuesday and lasting exactly 29 days.

Last revised: 01 Mar 2021, Rena

Page 4 of 26

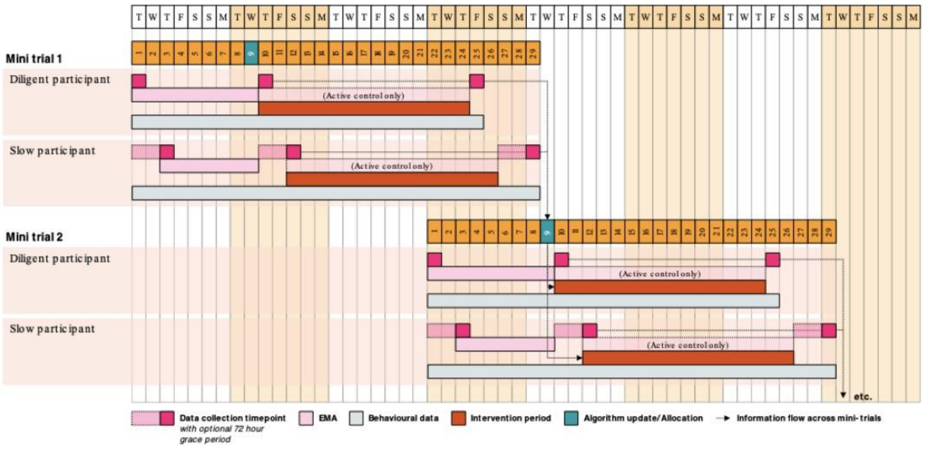

Figure 3: The timeline of how the mini-trials within the experiment will work

Figure 3 shows the high-level design of each mini-trial including the main data collection points and intervention periods. To show how this design can accommodate all user types, it represents the journey for two participants within each mini-trial, typifying extremes of behaviour: 1) diligent participants who complete requested tasks immediately and can therefore move on immediately to the next phase of their mini-trial and 2) slow participants who operate on a 'just in time' basis at every stage, only completing tasks at the last minute.

Commencement of a Mini-Trial can occur no earlier than Day 22 of the previous Mini-Trial (dependency is shown as the connecting line between the two Mini-Trials shown in Figure 3), so as to ensure that all data from the Post- Questionnaire Battery will be collected for the Optimiser to produce intervention program recommendations at Day 9 of the most recent Mini-Trial.

Although it cannot occur before Day 22, any Mini-Trial can always be arbitrarily delayed if required (e.g. to allow time for technical activity, recruitment etc). Any delayed Mini-Trial must nevertheless start on a Tuesday to ensure that contingent activities (e.g. any review of the Optimiser output) occur within the working week.

Web Enrollment

Web Enrolment is out of scope for this requirements document, will be described separately and will be built and deployed by BDI (please refer to the Vibe Up Clinical Trial Protocol).

2404

Experiment Lifecycle

The abbreviated character code per instrument must be preserved to ensure effective data transfer between organisations. All Survey codes will be suffixed with `-mt#`, where mt# refers to the mini-trial number. All Survey Within the Experiment Lifecycle, there are several phases to note, namely:

Phase 1 - Web Registration / Screening: Contingent on when the study is advertised, approximately 2 weeks before the start of the experiment, Participant Candidates can register for the study online.

Phase 1.5 - App Download: At 0800 hours on Day 1 of the experiment, selected Participant Candidates will be sent an SMS containing a link to download the app and commence their involvement in the study. They will have a window ending at 2359 hours of Day 3 of the experiment to download the app **and** complete the Pre- Questionnaire Battery within the app. This phase involves:

1. Downloading and installing the app from the App Store/Google Play store
2. Successfully completing the SMS verification workflow, which generates a participant id for the user and transitions the user from a Participant Candidate into a Participant.
3. Completing a 'Get started' onboarding flow which serves as a brief activation intervention by communicating purpose/value of using the app and prepares the user for the activities they will need to do and contextualizes the need to turn on sensing permissions.
4. Providing permissions for Passive data collection. Because permissions are optional and can be arbitrarily revoked by a user at any time, failure to complete permissions will not affect a user's ability to otherwise use the Mobile App. For users who grant permissions, all passive data collection **must cease automatically on Day 30**.

Phase 2 - Pre-Questionnaire Battery (Baseline): This is a series of surveys that are mandatory for a Participant to complete in order to proceed to the next phase of the experiment. App-generated reminders will be sent to complete each questionnaire battery, followed by an SMS reminder at 36 hours and then an email reminder at 48 hours if no response is received. The SMS and Email reminders are v2 features.

1. If the Participant has not completed the Pre- Questionnaire Battery by 2359 hours of Day 3 of the experiment, a notification will be sent to the user informing them that they have not completed a required component of the study, and will be moved into the pool of Participant Candidates for the next mini-trial. A user will only get 2 opportunities in total to take part in the study.
2. We will have to track via the participant mobile number against the list of mobile numbers we sent the SMS download link to, that the participant has not completed the Pre- Questionnaire Battery.

2405

2406

3. The instruments for this Battery are:
    a. Depression, Anxiety and Stress Scale, 21-item version (DASS, 21 items)
    b. Wellbeing Survey (WB, 4 items), consolidating the following into a single survey for streamlined user experience.
        i. Modified Physical Activity Vital Sign (PAVS, 2 items)
        ii. Abridged Pittsburgh Sleep Quality Index (PSQI, 1 item)
        iii. Mindfulness single item questionnaire (MIND, 1 item)

Phase 3 - EMA Phase: On completion of the Pre- Questionnaire Battery, a Participant will be able to start completing EMA surveys. The EMA surveys will be available to all Participants between Days 1 to 9 of the experiment. The Optimiser will ingest the vectors to generate recommendations per Participant on Day 9. Participants will be presented with their recommended intervention programs in the Intervention Exposure Phase, after completing the Mid- Questionnaire Battery. Each EMA measurement will consist of: 1) An extended Short Form (12 item version) of the Positive and Negative Affect Schedule (I-PANAS); and 2) Two follow-on questions exploring behavioural intentions as a result of measured feelings. EMA runs on a blended signal-contingent/event-based protocol consisting of:

1. Signal-contingent: One daily random prompt to the user generated by the study app at a random time in the morning (07:00-09:00). Participants will have up to 60 minutes to respond to this prompt.
2. Event-contingent: Participants will be able to log EMA measurements at any time (e.g. in response to self-identified exposures to negative stressors, although participants will be allowed to log any feelings.)
3. The EMA surveys consists of the following instruments:
    a. Modified Positive and Negative Affect Schedule, Short Form (PANAS; 12 items)
    b. Behavioural intentions
    c. If the participant triggers the conditional item in 3b - annotation should be included "We don't actively monitor responses to this question, but help is always available if you need it." plus a link to support options.

Phase 4 - Mid- Questionnaire Battery: This is a series of surveys that are mandatory for a Participant to complete to be eligible for their gift token and to move onto the next phase of the experiment. At 0800 hours on Day 10 of the experiment, Participants will be notified via the app that they have surveys to complete that are a mandatory aspect of their involvement in the study. They will have a window ending at 2359 hours of Day 12 of the experiment to complete this. App-generated reminders will be sent to complete each questionnaire battery, followed by an SMS reminder at 36 hours and then an email reminder at 48 hours if no response is received.

1. If the Participant has not completed the Mid- Questionnaire Battery by 2100 hours of Day 12 of the experiment, a notification will be sent to the user informing them that they have not completed a required component of the study, their app behaviour will be

2407

2408

locked (i.e. no surveys, no notifications, no passive data) till Day 30, and all intervention programs will be unlocked at the same time as the other participants in the Mini-trial.

2. The instruments for this Battery are:
    c. Depression, Anxiety and Stress Scale, 21-item version (DASS, 21 items)
    d. Wellbeing Survey (WB-baseline-mt#, 4 items), consolidating the following into a single survey for streamlined user experience.
        i. Modified Physical Activity Vital Sign (PAVS, 2 items)
        ii. Abridged Pittsburgh Sleep Quality Index (PSQI, 1 item)
        iii. Mindfulness single item questionnaire (MIND, 1 item)
    e. Abridged Credibility and Expectancy Questionnaire (CEQ, 2 items)
    f. Abridged Revised University of Rhode Island Change Assessment Scale (URC, 6 items)

Phase 5 - Intervention Exposure / Active Control: This phase lasts 14 days and commences once the Participant completes their Mid- Questionnaire Battery, which is available from 0800 hours on Day 10, through to 2200 hours of Day 12 of the experiment.

1. On commencing this phase, participants will:
    a. UPDATE (09 Dec 2020): Be provided an information card indicating their intervention assignment and communicating expected effort.
    b. Complete the Abridged Credibility and Expectancy Questionnaire (CEQ, 2 items)

2. Participant experience is **forked** in this phase. A subset of participants will serve as an Active Control group, while the rest of the participants will be exposed to intervention programs per the Optimiser's recommendations.

3. The arbitrary exposure period for an intervention program is 14 days. Participants that are not in the Active Control group will be allocated the intervention program of either Mindfulness, Physical Activity, or Sleep Hygiene. Each Intervention will have:
    a. An Introduction Module incorporating motivational, safety and effort information, which can be freely repeated by users, and **n** modules which are unlocked in sequence. The Introduction module and the first module will be accessible upon completion of the Mid- Questionnaire Battery. The first of 14 days of intervention exposure commences upon granting access to the module.
    b. A variable number of subsequent modules which are unlocked in sequence and can be freely repeated by users once unlocked and consist of infographics (for Sleep/Physical Activity but not Mindfulness interventions) and/or media (audio or video for Mindfulness/Physical Activity but not Sleep interventions). Multimedia content should be **distributed as part of the app bundle** to avoid negative UX relating to buffering/connectivity (front load the download burden);
    c. A set of text-based FAQs, which can be freely reviewed by users once unlocked (after completion of introductory module).

Last revised: 01 Mar 2021, Rena   Page 8 of 26

2409

d. An optional completion card showing a congratulatory message when all modules have been completed (Sleep/Mindfulness interventions only.)

4. Once the Introductory Module has been made accessible and completed, **FAQs and Module 2** will be made available. Each subsequent module will be made available as below:
    a. Mindfulness - after 24 hours
    b. Sleep Hygiene - after 48 hours
    c. Physical Activity - N/A

5. During this period, Participants will be able to complete an optional daily survey (log) that is associated with the intervention program. Completion of the survey (log) is **not** required for access to the next module. Concretely, the questions are:
    a. Mindfulness: Minutes spent practicing mindfulness.
    b. Physical Activity: Step count for the day
    c. Sleep Hygiene: Hours slept last night

6. The **Mindfulness Program** will consist of:
    a. Intro Module – Orientation video with brief text copy displayed below the video. Video/text will communicate purpose, value and expected effort.
    b. 5 audio modules consisting of a pop-up text caption (triggered by an 'info' icon on each audio option within the full list), audio-only video and debrief text card. These modules are:
        i. Audio 1 - Mindful breathing (audio - 3 min)
        ii. Audio 2 - Unhooking from your thoughts (audio - 3 min)
        iii. Audio 3 - Body scan (audio - 5 min)
        iv. Audio 4 - Mindful eating (audio - 3 min)
        v. Audio 5 - Mindful walking (audio - 3 min)
    c. FAQs
    d. Completion card shown if/when all modules completed.

7. The **Physical Activity Program** will consist of:
    a. Intro Module – Series of infographic-based cards navigable by the user.
    b. Daily Goal module – Cloned and repeated for each of the 14 days and consisting of:
        i. A simple interactive card to choose one of 4 daily goals: increase your steps; 7-minute workout; other activity; rest day. A pop-up text caption triggered by an 'info' icon on the 'Increase your steps' and 'Other activity' option will contain examples for each.
        ii. If the 'Increase your steps' option is chosen, a text card providing positive motivational message.

iii. If the '7-minute workout' option is chosen: a user-controllable video presenting a standardised 7-minute workout.
iv. If the 'Other activity' option is chosen, a text card providing positive motivational message.
v. If the 'Rest day' option is chosen, a text card providing tips on how to encourage physiological recovery, e.g. hydration, balanced food intake, etc.
vi. FAQs
vii. An optional completion card showing a congratulatory message when all modules have been completed.

8. The **Sleep Hygiene Program** will consist of:
   a. Intro Module (Sleep Module 1) – Infographic providing reading material about sleep and its importance.
   b. 3 infographic modules consisting of user-navigable cards. These modules are:
      i. Sleep Module 2 - Sleep habits
      ii. Sleep Module 3 - Sleep environment
      iii. Sleep Module 4 - Daily activities for sleep
   c. FAQs
   d. An optional completion card showing a congratulatory message when all modules have been completed (Sleep/Mindfulness interventions only.)

9. This Phase also encapsulates an Active Control group, where a subset of Participants will **not** be assigned an intervention program, but will instead continue to complete daily EMA surveys. Access to complete EMA surveys is only granted on completion of the Mid-Questionnaire Battery.
   a. The EMA surveys consists of the following instruments:
      i. Modified Positive and Negative Affect Schedule, Short Form (PANAS, 12 items)
      ii. Behavioural intentions (BHV, 1 item + 1 conditional item)
      iii. If the participant triggers the conditional item in aii - annotation should be included "We don't actively monitor responses to this question, but help is always available if you need it." plus a link to support options.

Phase 6 - Post- Questionnaire Battery: This is a series of surveys that are mandatory for a Participant to complete to be eligible for their gift token. At 0800 hours on the day **after** (Day 25 to 27 of the experiment) they have had access to their intervention program or EMA surveys (in the Active Control group), Participants will be notified via the app that they have surveys to complete that are a mandatory aspect of their involvement in the study. They will have a window ending at 2359 hours of Day 29, depending on when the Post-Questionnaire was made accessible, to complete this. App-generated reminders will be sent to complete each

2414

questionnaire battery, followed by an SMS reminder at 36 hours and then an email reminder at 48 hours if no response is received. On completion of the Post- Questionnaire Battery, Participants are transitioned into the End of Study Phase.

1. Hide all Intervention Program content until Post- Questionnaire Battery has been completed.
2. If the Participant has not completed the Post- Questionnaire Battery within the 72 hour window, no notification will be sent, but the participant will be automatically transitioned into the End of Study Phase.
3. The instruments for this Battery are:
    a. Depression, Anxiety and Stress Scale, 21-item version (DASS, 21 items)
    b. Wellbeing Survey (WB, 4 items), consolidating the following into a single survey for streamlined user experience.
        i. Modified Physical Activity Vital Sign (PAVS, 2 items)
        ii. Abridged Pittsburgh Sleep Quality Index (PSQI, 1 item)
        iii. Mindfulness single item questionnaire (MIND, 1 item)
    c. Within-study exposures questionnaire (EXP, 4 items)
    d. UPDATE (30 Nov 2020): UX questionnaire (UX, 9 items)

Phase 7 - End of Study Phase: On Day 30, regardless of whether or not the user has completed any/all of the previous phases, all participants will be migrated to the End of Study Phase.

1. In this phase, Active and Passive data collection will cease, and a notification will be sent via the app to inform the user of this.
2. All Intervention programs will now be made available to the user and they may elect to use the app freely.
3. Delivery of gift token is arranged (to be confirmed how this is orchestrated)

Key Components

This section describes additional functionality beyond what was described above to support facilitation of the study.

Mobile app

1. **Safety Information:** consisting of hotlines and assets for Participants to seek help and escalation
2. **Research Team Contact Details:** consisting of a monitored email address so Participants may contact the researchers.
3. **Issue Reporting:** Via Research team contact details; BDI will triage issues and send technical issues to A2I2. Crash reporting will still be viable as we will distribute the app using the A2I2 app store accounts rather than the Deakin account

Last revised: 01 Mar 2021, Rena Page 11 of 26

2415

4. **Map Screens:** to comply with app distribution channel requirements to visualise collected geospatial data to app users.
5. **Research Assets:** consisting of the Plain Language Statement and Informed Consent form so that Participants may access it at their convenience.
6. **Mini-Trial Timetable (nice to have):** A scheduled run sheet of the experiment and interaction points so that Participants can refer to it at their convenience.
7. **Survey Fields:** Unless otherwise stated, all survey fields are mandatory for the participants to complete.

Infrastructure

1. **UNSW Data Governance Compliance:** The system must largely comply with the requirements of the UNSW Data Governance Compliance spreadsheet. This also entails:
    a. An agreed System Operations Monitoring Protocol, and
    b. An agreed Incident Response Protocol.
2. **Information Exchange Protocol with Shared Index:** To maintain association of participant data collected via the web **and** mobile app.
    a. 1st version: First Name, Last Name, Mobile, Email
3. **Data Export:** To provide visibility into the progress of current and previous mini-trials. The date for delivery of this has not been finalised, as the facilitation of the core study protocol is of a higher priority. Data export refers to:
    a. Participant engagement, per mini-trial,
    b. Data Vectors to be provided to the Optimiser for post-hoc analysis, and
    c. Optimiser output for post-hoc analysis.

Backlog

1. Permutation list of permission settings for Android.
2. Master opt in for passive sensing that is derived from Web enrollment. If passive sensing is opted out of, do not ask for permissions for sensing.
3. "More" Screen addition of how we are using the data we are collecting, as a hyperlink to a BDI hosted site.
4. App settings to only upload data when on WiFi.
5. Onboarding flow that immediately follows TOTP to walk a user through the app.
6. Male and Female voice selector for mindfulness recordings
7. Survey attempt start analytics.
8. Dropdown list vs 2 column radio CEQ.
9. Send an SMS to participants at 1800 hours the day before to tell users the experiment starts at 0800 the next morning.
10. For Mindfulness and Sleep, if there are no more new modules, ensure the notification sent is appropriate (i.e. does not as the user to try the next module)

11. Start your <intervention> challenge today... {xx} days left!
12. EMA has a 60 minute and 30 minute notification <Post-Pilot>

Survey/Questionnaire Codes

Survey Code	Questionnaire	Item count	Phases
ELY	Basic eligibility	11	Web Screening
CTD	Contact details	4	Web Screening
MED	Physical and mental health	8	Web Screening
KTEN	Kessler Psychological Distress Scale, 10-item version	10	Web Screening
SIDAS	Extended Suicidal Ideation Attributes Scale	6	Web Screening
DEM	Demographic details	9	Web Screening
WRK	Study and employment	4	Web Screening
SES	Subjective Socioeconomic Status Scale	1	Web Screening
EQV	Abridged EQ-5D-5L	1	Web Screening
AOD	NIDA-Modified ASSIST Drug Screening Tool	4	Web Screening
WBS	Short Warwick Edinburgh Mental Wellbeing Scale	7	Web Screening
PSS	Multidimensional Scale of Perceived Social Support	12	Web Screening
DASS	Depression, Anxiety and Stress Scale, 21-item version	21	Baseline, Mid, Post
PAVS	Modified Physical Activity Vital Sign	2	Baseline, Mid, Post
PSQI	Abridged Pittsburgh Sleep Quality Index	1	Baseline, Mid, Post
MIND	Mindfulness single item questionnaire	1	Baseline, Mid, Post
PANAS	Modified Positive and Negative Affect Schedule, Short Form	13	EMA
BHV	Behavioural intentions	2	EMA
CEQ	Abridged Credibility and Expectancy Questionnaire	2	Mid
URC	Abridged Revised University of Rhode Island Change Assessment Scale	6	Mid
LOG	Daily log of engagement with intervention	1	Intervention
EXP	Within-study exposures questionnaire	4	Post
UX	UX questionnaire	9	Post

Last revised: 01 Mar 2021, Rena

Page 13 of 26

2420

Key Dates

Date	Milestone	Primary Lead	Notes
10 Dec 2020	v0.1 End to End of DASS on app, into vectorisor, into optimiser, into recommendation engine.	Deakin	
**08 Jan 2021**	Intervention content and structure finalised. All content provided either as placeholders or in the final format that will be used in the app.	**BDI**	Slipped to 08 Feb 2021; partially completed and undergoing revisions.
20 Jan 2021	v0.2 Addition of further functionality toward fuller coverage of the study protocol (refer to requirements document for details)	Deakin	
24 Jan 2021	Optimisation statistical aspects discussed with BDI biostatistician	Joint	
24 Jan 2021	App Release notes format established, and releases are tagged	Deakin	
24 Jan 2021	Technical Specs – all items added 09/10 Dec 2020 are considered in release planning and release dates updated	Deakin	
31 Jan 2021	Data exchange protocol for participant information between Deakin and BDI established	BDI	
31 Jan 2021	Ethics for study expected to be approved [Fingers crossed]	BDI	
**TESTING – Flow, information architecture and structure focused**			
**05 Feb 2021**	Delivery of final intervention assets (videos/audios)	**BDI**	Deakin requested audio only files, all videos received
06 Feb 2021	Release notes format finalised in collaboration with BDI	Deakin	
**08 Feb 2021**	Delivery of survey, intervention and notification content with flows and visual assets.	**BDI**	
10 Feb 2021	v0.3 (Fleshing out the survey dispatch/delivery) released for beta testing along with release notes	Deakin	Slipped due to pulling forward architectural work as we were waiting

Last revised: 01 Mar 2021, Rena Page 14 of 26

2421

			on UX content. Estimated resolution Mon 22nd Feb / Tue 23rd Feb
12 Feb 2021	Review of survey, intervention and notification content with flows and visual assets.	Deakin	
**15 Feb 2021**	Internal user testing starts for v0.3 (private deployments) – primary focus is on app flow and general structure of the overall app. (release used is not the one distributed via app stores)	**BDI**	Originally planned to start introducing survey and intervention content but will be done in v0.3.5 and v0.4 instead due to 08-Feb receipt of assets + Commencement on 23rd Feb, contingent on v0.3 release
17 Feb 2021	Deakin ethics application lodged (based on approved from UNSW)	Deakin	Joint Clarifications 18th Feb
17 Feb 2021	Cloud infrastructure monitoring and escalation procedures progress update – sent by Deakin and discussed jointly	Joint	Sent to BDI
17 Feb 2021	Testing strategy established (used by dev team)	Deakin	
24 Feb 2021	App Deployment Protocol to transmit apps to BDI for submission to App Stores finalised	Deakin	
24 Feb 2021	System to capture issues setup	Deakin	
**24 Feb 2021**	Data exchange protocol used to send user data (to test that component)	Joint	
03 Mar 2021	v0.3.5 – Inclusion of survey and notification content with flows and visual assets	Deakin	
**08 Mar 2021**	Internal user testing starts for v0.3.5 (private deployments) – primary focus is on survey and notification delivery and experience. (release used is not the one distributed via app stores)	Deakin	

22 Mar 2021	User testing of v0.3 completed – feedback sent to team for discussion	Deakin	
30 Mar 2021	Optimiser updated to address feedback from BDI / stats experts	Deakin	
**31 Mar 2021**	**v1.0 internal release. App with surveys and intervention programs to be tested end to end with all content and visuals. – Internal testing.**	**Deakin**	
05 Apr 2021	Transmit v1.0 app packages to BDI for submission (using agreed protocol)		
05 Apr 2021	Internal user testing starts for v1.0 (private deployments) – primary focus is on holistic experience and protocol adherence with surveys, interventions and notification delivery and experience. (release used is not the one distributed via app stores)	Deakin	
07 Apr 2021	v1.0 – Uploaded to App Stores	BDI	Moved from 19 Feb 2021; ensures submission reflects the design / content.
14 Apr 2021	Internal testing of v1.0 completed – feedback sent to team for discussion	Joint	
19 Apr 2021	Revisions for v1.1 discussed and planned	Joint	
**01 May 2021**	**Decision point: go/no-go for pilot phase.**	**Joint**	
**01 May 2021**	**Pilot Study 1 recruitment starts (UNSW-based)**	**BDI**	Moved from 22 Mar 2021
**05 May 2021**	**v1.1 internal release, implementation of v1.1 feedback**	**Joint**	
10 May 2021	Internal user testing starts for v1.1 (private deployments) – primary focus is on holistic experience and protocol adherence with surveys, interventions and notification delivery and experience, as well as optimiser updates from v1.0 (release used is not the one distributed via app stores)	Joint	

Last revised: 01 Mar 2021, Rena

Page 16 of 26

12 May 2021	Apps on App Store (Production v1)	Joint	
14 May 2021	Support for cloud infrastructure and escalation procedures are fully established	Deakin	
17 May 2021	Internal testing of v1.1 completed – feedback sent to team for discussion	Deakin	
**18 May 2021**	**Pilot Study 1 starts – target is v1 (One end-to-end mini-trial)**	**BDI**	Moved from 04 Apr 2021
20 May 2021	Transmit v1.1 app packages to BDI for submission (using agreed protocol)	Deakin	
24 May 2021	v1.1 – Uploaded to App Stores	**BDI**	
31 May 2021	***UNSW T2 Starts***	**-**	
04 Jun 2021	v1.1 on App Store	Joint	
**08 Jun 2021**	**Pilot Study 1 ends**	**BDI**	Moved from 04 May 2021
10 Jun 2021	Formative feedback collected and analysed	BDI	
14 Jun 2021	Pilot feedback/revisions to be incorporated are agreed	Joint	
30 Jun 2021	Issue escalation process & associated team (for end-users) established	BDI	
**30 Jun 2021**	**v1.2 Release, feedback from Pilot 1**	**Deakin**	
**01 Jul 2021**	**Go/No Go slippage decision re: Pilot 2 + Subsequent Trial**	**Joint**	
01 Jul 2021	**Pilot Study 2 recruitment starts (Deakin-based)**	**Joint**	
05 Jul 2021	Internal Testing of v1.2	Joint	
06 Jul 2021	Transmit v1.2 app packages to BDI for submission (using agreed protocol)	BDI	
08 Jul 2021	v1.2 – Uploaded to App Stores	BDI	
09 Jul 2021	End Internal Testing of v1.2	Joint	
12 Jul 2021	*Deakin T2 Starts*	-	
12 Jul 2021	v1.2 on App Store	Joint	
**20 Jul 2021**	**Pilot Study 2, Mini-trial 1 starts**	**Joint**	

Last revised: 01 Mar 2021, Rena

Page 17 of 26

2428

07 Aug 2021	*UNSW T2 Study Period*	-	
**17 Aug 2021**	**Pilot Study 2, Mini-trial 1 ends**	**Joint**	
22 Aug 2021	Incorporate procedural learning (anticipated learning around how to support and run these administratively)	Deakin	
**24 Aug 2021**	**Pilot Study 2, Mini-trial 2 starts**	**Joint**	
**21 Sep 2021**	**Pilot Study 2, Mini-trial 2 ends**	**Joint**	
27 Sep 2021	Stage 1 debrief/learnings/improvements completed	Joint	
04 Oct 2021	*Deakin T2 Study Period*	-	
**13 Oct 2021**	**Production v1.3 (Updates/improvements) released and published to app store – flexible overlapping mini-trials supported by this release**	**Joint**	

Last revised: 01 Mar 2021, Rena Page 18 of 26

2429

Roadmap

Release	Scope	Comments
v.0.1	**Goal:** End to End of DASS on app, into vectorisor, into optimiser, into recommendation engine.<br><br>Web App (BDI):<br>• First iteration of data exchange protocol to enable association between web-collected and app-collected data. This includes:<br>• CSV Data schema: First Name, Last Name, Mobile, Email<br>• Data exchange schedule<br>• Assignment of executors for protocol<br><br>Mobile App:<br>• Single page BTAS app with new name (Name: VibeUp, Icon: pick an up arrow from font awesome or something similar)<br>• Delivery of Optimise surveys at fixed schedule, where currently technically possible (i.e. finalised instruments, fixed schedules)<br>○ Day 1 DASS @ 1000 expires 2359<br>○ Day 1 EMA @ 1015 expires 1115<br>○ Run vectoriser at Day 2 0900<br>○ Day 2 DASS @ 1000 expires 2359<br>○ Day 2 EMA @ 0947 expires 1047<br>○ Day 2 intervention delivery @ 1400<br>○ Day 3 DASS @ 1000 expires 2359<br>○ Day 3 EMA @ 1105 expires 1205<br>• Ability to receive TEXT recommendation and show it to users.<br>○ Intervention Program will use existing BTAS Text block to show title and body text which repeats the title text.<br>• Safety (Get Help screen) content, where currently technically possible, click to call the number, leveraging OS pre-built friction for confirmation<br>• Add support for device battery level passive data collection<br>Back End:<br>• Implement feature extractor for the study<br>• Optimiser implemented for the study	Release Date: 10 Dec 2020

Last revised: 01 Mar 2021, Rena Page 19 of 26

	• Add support for a survey to be able to be submitted multiple times	
v.0.2	**Goal:** Addition of further functionality toward fuller coverage of the study protocol.<br><br>Web App (BDI):<br>• (Deakin) PCM Backend with support for csv data import using first name, last name, mobile and email.<br>• Import Support for allow_passive_data field to be captured during web enrolment<br><br>Mobile App:<br>• TOTP (only via SMS) that verifies against the imported mobile numbers<br>• Change from mandatory permissions to optional for passive data collection<br>• Dynamic EMA survey behaviour that hides/shows questions based on the multi-selected emotions in the first question.<br>• EMA has support for the user to complete and submit (all submissions get persisted) as many times as they like, which will be overridden if a time based EMA is available.<br>• Conditional survey feature, specifically to hide EMA till after DASS has been completed<br>• First draft of Protocol to detect users who have not completed their Questionnaire Battery within the 72 hour window<br>• Settings with links or placeholders to BDI Terms and Conditions/Privacy Policy<br><br>Back End:<br>• Estimates to support dynamic participant timeline around 72 hour windows to complete Questionnaire Batteries<br>• Estimates for overlapping mini-trial support	Release Date: 20 Jan 2021.
v0.3	**Goal:** Fleshing out the intervention delivery.<br><br>Web App: N/A<br><br>Mobile App:<br>• Support for allow_passive_data field to change	Release date: 10 Feb 2021.

	App sensor permissions screens<br>• Intervention Programs have different modules<br>• Conditional survey feature, specifically to hide EMA till after DASS has been completed<br>• When finalised<br>○ Intervention Programs have either media content or placeholders<br>○ Intervention Programs will have text content and infographics, or placeholders<br>○ Addition of surveys associated with Intervention Program<br>• Addition of other surveys described in the Experiment Lifecycle<br>• Finalise Technical Protocol to detect users who have not completed their Questionnaire Battery at Day 4 and Day 13<br><br>Back End:<br>• Draft data export requirements<br>• Custom Push notifications<br>• Support to cease passive data collection by stopping silent notifications<br>• Scheduled Timescale backups with dry run restoration	
v0.3.5	**Goal:** Building out support for critical Study Protocol components (functionality explicitly listed in v0.1 to v0.3 plus feedback from prior releases, if feasible). Refinements based on prior version feedback.<br><br>Web App:<br><br>Mobile App:<br>• When finalised, PARTIAL implementation of:<br>○ Intervention Programs have either media content or placeholders<br>○ Intervention Programs will have text content and infographics, or placeholders<br>○ Addition of surveys associated with Intervention Program<br><br>Back End:<br>• Finalise data export requirements	Release date: 24 Feb 2021. There are likely to be minor releases. Release date contingent on velocity from prior releases.

Last revised: 01 Mar 2021, Rena Page 21 of 26

v0.4	**Goal:** Full support for critical Study Protocol components (functionality explicitly listed in v0.1 to v0.3 plus feedback from prior releases, if feasible). Refinements based on prior version feedback. This is the targeted release that will be pushed to App Stores.<br><br>Web App:<br><br>Mobile App:<br>• UI Refinements to surveys, contingent on feedback of prior versions.<br>• When finalised, PARTIAL? implementation of:<br>○ Intervention Programs have either media content or placeholders<br>○ Intervention Programs will have text content and infographics, or placeholders<br>○ Addition of surveys associated with Intervention Program<br><br>Back End:<br>• Finalise data export requirements	Release date: 03 Mar 2021. There are likely to be minor releases. Release date contingent on velocity from prior releases.
v0.5	**Goal:** Refinements based on prior version feedback.<br><br>Web App:<br><br>Mobile App:<br><br>Back End:<br>• Implement Technical Protocol to detect users who have not completed their Questionnaire Battery at Day 4 and Day 13	Release date: TBD.
v0.6	**Goal:** Refinements based on prior version feedback.<br><br>Web App:<br><br>Mobile App:<br><br>Back End:<br>• Support Overlapping Mini-trials<br>• Support data export requirements	Release date: TBD.

Last revised: 01 Mar 2021, Rena   Page 22 of 26

2438

v1.0	**Goal:** Apps are available on App Store/Google Play. Apps need to be submitted 1 month in advance from the experiment start date.	Release date: TBD.

2439

Appendix A - Web Based Enrollment Requirements

Currently out of scope but retained in this document for record-keeping purposes. Within the web enrolment component, a user will have to complete phases, described below.

- **Plain Language Statement (PLS) / Participant Information Statement (PIS):** The PLS / PIS will be shown to the user, where they have the option to decline and leave, or agree and continue to the next phase.

- **Informed Consent:** The Informed Consent form will be shown to the user, where they have the option to decline and leave, or agree and continue to the next phase.
- **Eligibility Screening:** A series of surveys and questionnaires will be presented for the user to complete to determine their eligibility for the study. There are three possible outcomes from this:
    a. They are eligible and proceed to the next phase. Once eligibility is confirmed, we consider users as Participant Candidates.
    b. They are ineligible and are **not** viewed as at high risk. A thank you message is displayed and their journey terminates here.
    c. They are ineligible **and** are viewed as high risk. Users flagged as High Risk will proceed to the next phase where they are provided with the option to have a clinical psychologist follow up and we collect their contact details so that we may discharge our duty of care.

- **Contact/Registration Details:** This phase captures personally identifiable details of the Participant Candidate, including but not limited to preferred name, mobile phone number, and email address.
    a. Users flagged as High Risk terminate here, with a thank you message and an indication that they should expect an opt-in follow-up call (if participant has agreed).

- **Onboarding:** This phase provides a series of mandatory questionnaires and surveys for the Participant Candidate to complete. Completion of this will display a thank you message that indicates the Participant Candidate should expect an SMS to download the app, and the scheduled experiment commencement date.

The enrolment instruments for this phase are listed here. The abbreviated character code per instrument must be preserved to ensure effective data transfer between BDI and A²I².

- Basic eligibility (ELY, 11 items)
- Contact details (CTD, 4 items)
- Physical and mental health (MED, 8 items)
- Kessler Psychological Distress Scale, 10-item version (KTEN, 10 items)

Last revised: 01 Mar 2021, Rena

Page 24 of 26

- Extended Suicidal Ideation Attributes Scale (SIDAS, 6 items)
- Demographic details (DEM, 9 items)
- Study and employment (WRK, 4 items)
- Subjective Socioeconomic Status Scale (SES, 1 item)
- Abridged EQ-5D-5L (EQV, 1 item)
- NIDA-Modified ASSIST Drug Screening Tool (AOD, 4 items)
- Short Warwick Edinburgh Mental Wellbeing Scale (WBS, 7 items)
- Multidimensional Scale of Perceived Social Support (PSS, 12 items)

Appendix B - Internal Test Users

There will be a set of internal test users composed from the team at BDI and Deakin. These users will be recipients of our release notes for the minor versions leading up to the pilot release. Full details for the test users can be requested from Leonard Hoon.

The feedback mechanism for collecting issues and feedback for the pre-pilot builds will be via google forms, also available via the settings screen, in app.

2446
2447

Appendix 29 Vibe Up App Testing Plan

Tester:				Tester Profile:
Functional Cases:				
Download App			Pass	
TOTP			Pass	
Permissions			Fail	
Survey Notification				
Survey Discard				
Survey Expiry				
Survey Submission				

OVERVIEW

Phase	Passed Cases	Total Cases	% Passed	Comments
1.5		32	0.00%	
2	0	17	0.00%	
3		54	0.00%	
4		23	0.00%	
5		116	0.00%	
6		16	0.00%	
7		1	0.00%	
All		1	0.00%	

ID	Phase #	Phase Name	Component #	Component	Day	Time	Condition	User Action	View	Modality	Instrument	# Questions	Intervention	Module	Pass/Fail	Message	Comment	Back End Lifec	Info Exchange I	Questions/Comments	Payload
1	1.5	App Download	1	Splash	1		App downloaded/ opened		Splash Logo	App											
2	1.5	App Download	1	Splash	1		App downloaded/ opened		Splash Vibe Up	App											
3	1.5	App Download	2	OTP	1			Tap phone #	Get Started	App											
4	1.5	App Download	2	OTP	1			Enter phone #	Get Started	App											
5	1.5	App Download	2	OTP	1		Phone # entered	Phone #	Get Started	App											
6	1.5	App Download	2	OTP	1		Phone # validated			SMS Code						Verification SMS + Code					
7	1.5	App Download	2	OTP	1		Phone # not validated		Phone # not	App											
8	1.5	App Download	2	OTP	1		SMS sent?		Enter Code	App											
9	1.5	App Download		OTP			Didn't get code			SMS Code											
10	1.5	App Download		OTP			New code sent		Enter Code	App											
11	1.5	App Download	2	OTP	1			Tap on space	Enter Code +	App											
12	1.5	App Download	2	OTP	1		Code entered		Display Code +	App											
13	1.5	App Download		OTP			Code wrongly entered		Invalid Code +	App											
14	1.5	App Download		OTP	1		Code expired		Enter Code												
15	1.5	App Download		OTP			Code error		Enter Code												
16	1.5	App Download	2	OTP	1		Code entered	User clicks on	Onboarding	App											
17	1.5	App Download	3	Onboarding & Permissions	1		Code verified		Welcome	App											
18	1.5	App Download	3	Onboarding & Permissions	1		Next clicked		All Tasks	App											
19	1.5	App Download		Onboarding & Permissions			Next clicked		Motion	App											
20	1.5	App Download		Onboarding & Permissions	1		Motion 'enable' clicked		Location	App											
21	1.5	App Download		Onboarding & Permissions																	
22	1.5	App Download		Onboarding & Permissions	1		Location 'next' clicked		Access Location Pop	App											
23	1.5	App Download	3	Onboarding & Permissions	1		Next clicked		Task Reminders	App											
24	1.5	App Download	3	Onboarding & Permissions	1		Next clicked		Your Location	App											
25	1.5	App Download	3	Onboarding & Permissions	1		Next clicked		Vibe Up	App											
26	1.5	App Download	3	Onboarding & Permissions	1		Location Data Selected from Vibe Up		Health Data	App											
27	1.5	App Download	3	Onboarding & Permissions	1		Next clicked		Health	App											
28	1.5	App Download	4	Dashboard	1		Categories Turned On		Support	App											
29	1.5	App Download	4	Dashboard	1		Next clicked		Tasks Intro	App											
30	1.5	App Download	4	Dashboard	1		Next clicked		Vibey Intro	App											
31	1.5	App Download	4	Dashboard	1		Next clicked		Tasks	App											
32	1.5	App Download	4	Dashboard	1			All Tasks	All Tasks	App											
33	All	All	5	Get Help				User clicked on Services	Get Help	App											
34	2	Pre-Questionnaire Battery (Baseline)	6	Mood Survey	1			Mood Survey Selected -	Mood Survey	App											
35	2	Pre-Questionnaire Battery (Baseline)	6	Mood Survey	1	10:00	72 hours			App	DASS-21	21	N/A				Psychological di	Pre		Diligent participants/Incomplete in 72 hrs - Excluded	
36	2	Pre-Questionnaire Battery (Baseline)	6	Mood Survey	1		Close survey		Close the	App						Are you sure - progress not saved					
37	2	Pre-Questionnaire Battery (Baseline)	6	Mood Survey	1		Mood Survey Completed		Well Done/Home	App											
38	2	Pre-Questionnaire Battery (Baseline)	6	Mood Survey			Mood Survey Completed	Home Button Pressed	Tasks	App											
39	2	Pre-Questionnaire Battery (Baseline)	7	Well Being Survey	1			Well Being Survey Selected -	Well Being Survey	App											
40	2	Pre-Questionnaire Battery (Baseline)	7	Well Being Survey	1	10:00	72 hours / Mood Survey Completed?			App	PAVS	2	N/A				Physical Activity	Pre		Diligent participants/Incomplete in 72 hrs - Excluded	
41	2	Pre-Questionnaire Battery (Baseline)	7	Well Being Survey Survey	1	10:00	72 hours			App	PSQI	1	N/A				Sleep Quality Inc	Pre		Diligent participants/Incomplete in 72 hrs - Excluded	
42	2	Pre-Questionnaire Battery (Baseline)	7	Well Being Survey	1	10:00	72 hours			App	MIND	1	N/A				Mindfulness sing	Pre			
43	2	Pre-Questionnaire Battery (Baseline)	7	Well Being Survey	1		Wellbeing Survey Completed		Well Done/Home	App											
44	2	Pre-Questionnaire Battery (Baseline)	6	Well Being Survey			Wellbeing Survey Completed	Home Button Pressed	Tasks	App											
45	2	Pre-Questionnaire Battery (Baseline)	6	Mood Survey	1		120mins after completing Phase 1.5: App Download, if failed to complete Mood Survey questionnaire			App											
46	2	Pre-Questionnaire Battery (Baseline)	7	Well Being Survey			120mins after completing Phase 2: Baseline verify/permissions, if failed to complete Well Being questionnaire			App											
47	2	Pre-Questionnaire Battery (Baseline)	6	Mood Survey	2	2000	failed to complete Mood Survey - 36 hrs after app notification			SMS						Last chance! Answer the kick-off questions before midnight tomorrow to stay in the study					
48	2	Pre-Questionnaire Battery (Baseline)	7	Well Being Survey		2000	failed to complete Well Being - 48 hrs after app notification			SMS											
49	2	Pre-Questionnaire Battery (Baseline)	6	Mood Survey	3	800	failed to complete Mood Survey			Email						TBC					
50	2	Pre-Questionnaire Battery (Baseline)	7	Well Being Survey	3	800	failed to complete Well Being			Email											
51	3	EMA	8	Mood Check-In	1				Mood Check-In Intro	App											
52	3	EMA	8	Mood Check-In	1	Random bw 0700-1900	baseline questionnaire completed			App/Notification						How are you feeling right now? Let's check in					
53	3	EMA	8	Mood Check-In	1	0700-1900	60 min			App	PANAS	12	-				Positive and neg	Baseline Phase		Slow participants start on day 3	

2448

54	3	EMA	8	Mood Check-In	1	0700-1900	60 min			App	BHV	2	-		Coping behavior	Baseline Phase		* Conditional - if answer to Question 1 is 'unlikely' will not be asked second question
55	3	EMA	8	Mood Check-In	1				Well Done/Home	App								
56	3	EMA	8	Mood Check-In			EMA Completed	Home Button Pressed	Tasks	App								
57	3	EMA	8	Mood Check-In	2				Mood Check-In Intro	App								
58	3	EMA	8	Mood Check-In	2	Random bw 0700-1900	baseline questionnaire completed			App/Notification				Complete your daily check-in to keep XX healthy. ⌛ Available for 1 hour				
59	3	EMA	8	Mood Check-In	2		60 min			App	PANAS	12	-		Positive and neg	Baseline Phase		
60	3	EMA	8	Mood Check-In	2		60 min			App	BHV	2	-		Coping behavior	Baseline Phase		
61	3	EMA	8	Mood Check-In	2				Well Done/Home	App								
62	3	EMA	8	Mood Check-In	2		EMA Completed	Home Button Pressed	Tasks	App								
63	3	EMA	8	Mood Check-In	3				Mood Check-In Intro	App								
64	3	EMA	8	Mood Check-In	3	Random bw 0700-1900	baseline questionnaire completed			App/Notification				🌡 Take your emotional temperature. Time for your daily check-in (60 mins to go)				
65	3	EMA	8	Mood Check-In	3		60 min			App	PANAS	12	-		Positive and neg	Baseline Phase		
66	3	EMA	8	Mood Check-In	3		60 min			App	BHV	2	-		Coping behavior	Baseline Phase		
67	3	EMA	8	Mood Check-In	3				Well Done/Home Button	App								
68	3	EMA	8	Mood Check-In			EMA Completed	Home Button Pressed	Tasks	App								
69	3	EMA	8	Mood Check-In	4				Mood Check-In Intro	App								
70	3	EMA	8	Mood Check-In	4	Random bw 0700-1900	baseline questionnaire completed			App/Notification				How are you feeling right now? Let's check in				
71	3	EMA	8	Mood Check-In	4		60 min			App	PANAS	12	-		Positive and neg	Baseline Phase		
72	3	EMA	8	Mood Check-In	4		60 min			App	BHV	2	-		Coping behavior	Baseline Phase		
73	3	EMA	8	Mood Check-In	4				Well Done/Home Button	App								
74	3	EMA	8	Mood Check-In			EMA Completed	Home Button Pressed	Tasks	App								
75	3	EMA	8	Mood Check-In	5				Mood Check-In Intro	App								
76	3	EMA	8	Mood Check-In	5	Random bw 0700-1900	baseline questionnaire completed			App/Notification				Complete your daily check-in to keep XX healthy. ⌛ Available for 1 hour				
77	3	EMA	8	Mood Check-In	5		60 min			App	PANAS	12	-		Positive and neg	Baseline Phase		
78	3	EMA	8	Mood Check-In	5		60 min			App	BHV	2	-		Coping behavior	Baseline Phase		
79	3	EMA	8	Mood Check-In	5				Well Done/Home Button	App								
80	3	EMA	6	Mood Check-In			EMA Completed	Home Button Pressed	Tasks	App								
81	3	EMA	8	Mood Check-In	6				Mood Check-In Intro	App								
82	3	EMA	8	Mood Check-In	6	Random bw 0700-1900	baseline questionnaire completed			App/Notification				🌡 Take your emotional temperature. Time for your daily check-in (60 mins to go)				
83	3	EMA	8	Mood Check-In	6		60 min			App	PANAS	12	-		Positive and neg	Baseline Phase		
84	3	EMA	8	Mood Check-In	6		60 min			App	BHV	2	-		Coping behavior	Baseline Phase		
85	3	EMA	8	Mood Check-In	6				Well Done/Home Button	App								
86	3	EMA	8	Mood Check-In			EMA Completed	Home Button Pressed	Tasks	App								
87	3	EMA	8	Mood Check-In	7				Mood Check-In Intro	App								
88	3	EMA	8	Mood Check-In	7	Random bw 0700-1900	baseline questionnaire completed			App/Notification				How are you feeling right now? Let's check in				
89	3	EMA	8	Mood Check-In	7		60 min			App	PANAS	12	-		Positive and neg	Baseline Phase		
90	3	EMA	8	Mood Check-In	7		60 min			App	BHV	2	-		Coping behavior	Baseline Phase		
91	3	EMA	8	Mood Check-In	7				Well Done/Home Button	App								
92	3	EMA	8	Mood Check-In			EMA Completed	Home Button Pressed	Tasks	App								
93	3	EMA	8	Mood Check-In	8				Mood Check-In Intro	App								
94	3	EMA	8	Mood Check-In	8	Random bw 0700-1900	baseline questionnaire completed			App/Notification				Complete your daily check-in to keep XX healthy. ⌛ Available for 1 hour				
95	3	EMA	8	Mood Check-In	8		60 min			App	PANAS	12	-		Positive and neg	Baseline Phase		
96	3	EMA	8	Mood Check-In	8		60 min			App	BHV	2	-		Coping behavior	Baseline Phase		
97	3	EMA	8	Mood Check-In	8				Well Done/Home Button	App								
98	3	EMA	8	Mood Check-In			EMA Completed	Home Button Pressed	Tasks	App								
99	3	EMA	8	Mood Check-In	9				Mood Check-In Intro	App								
100	3	EMA	8	Mood Check-In	9	Random bw 0700-1900	baseline questionnaire completed			App/Notification				🌡 Take your emotional temperature. Time for your daily check-in (60 mins to go)				
101	3	EMA	8	Mood Check-In	9		60 min			App	PANAS	12	-		Positive and neg	Baseline Phase		
102	3	EMA	8	Mood Check-In	9		60 min			App	BHV	2	-		Coping behavior	Baseline Phase		
103	3	EMA	8	Mood Check-In	9				Well Done/Home Button	App								
104	3	EMA	8	Mid	10	800								Good work on getting halfway through the study 👏 Do a quick survey to enter Part 2 of Vibe Up!				
105	4	Mid- Questionnaire Battery	6	Mood Survey	10			Mood Survey Selected -	Mood Survey	App								
106	4	Mid- Questionnaire Battery	6	Mood Survey	10	800	72 hours			App	DASS-21	21	N/A		Psychological di	Mid Phase (Pre-I	5 Nov meeting. N	Next Mini-Trial commences Baseline/Incomplete in 72 hrs - Excluded
107	4	Mid- Questionnaire Battery	6	Mood Survey	1		Close survey		Close the	App								
108	4	Mid- Questionnaire Battery	6	Mood Survey	1		Mood Survey Completed		Well Done/Home Button	App								
109	4	Mid- Questionnaire Battery	7	Well Being Survey	1			Well Being Survey Selected -	Well Being Survey	App								
110	4	Mid- Questionnaire Battery	7	WB Survey	10	800	72 hours			App	PAVS	2	N/A		Physical Activity	Mid Phase (Pre-Intervention)		/Incomplete in 72 hrs - Excluded
111	4	Mid- Questionnaire Battery	7	WB Survey	10	800	72 hours			App	PSQI	1	N/A		Sleep	Mid Phase (Pre-Intervention)		/Incomplete in 72 hrs - Excluded
112	4	Mid- Questionnaire Battery	7	WB Survey	10	800	72 hours			App	MIND	1	N/A		Mindfulness sing	Mid Phase (Pre-Intervention)		/Incomplete in 72 hrs - Excluded
113	4	Mid- Questionnaire Battery	7	Well Being Survey	1		Wellbeing Survey Completed		Well Done/Home Button	App								
114	4	Mid- Questionnaire Battery	9	Change Survey	10	800		Change Survey Selected -	Change Survey - Start Button	App	URC							
115	4	Mid- Questionnaire Battery	9	Change Survey	10	800	72 hours			App	URC	6	N/A		Change Assessr	Mid Phase (Pre-Intervention)		/Incomplete in 72 hrs - Excluded

2449

2450

116	4	Mid- Questionnaire Battery	9	Change Survey	10	800	Change Survey Completed		Change Survey - Home Button	App	URC										
117	4	Mid- Questionnaire Battery	10	CEQ	10	800		CEQ Survey Selected -	CEQ Survey - Start Button	App	CEQ										
118	4	Mid- Questionnaire Battery	10	CEQ	10	800	72 hours			App	CEQ		2	N/A				Credibility and E	Mid Phase (Pre-Intervention)		/Incomplete in 72 hrs - Excluded
119	4	Mid- Questionnaire Battery	10	CEQ	10	800	CEQ Survey Completed		CEQ Survey- Home Button	App	CEQ										
120	4	Mid- Questionnaire Battery	6	Mid	11	20:00	failed to complete Mood Survey			SMS							Hey [firstname]! Finish a quick survey in Vibe Up by midnight tomorrow to stay in the study				
121	4	Mid- Questionnaire Battery	7	Mid	11	20:00	failed to complete Well Being			SMS											
122	4	Mid- Questionnaire Battery	9	Mid	11	20:00	failed to complete Change Survey			SMS											
123	4	Mid- Questionnaire Battery	10	Mid	11	20:00	failed to complete CEQ Survey			SMS											
124	4	Mid- Questionnaire Battery	6	Mid	12	800	failed to complete Mood Survey			Email							TBC				
125	4	Mid- Questionnaire Battery	7	Mid	12	800	failed to complete Well Being			Email											
126	4	Mid- Questionnaire Battery	9	Mid	12	800	failed to complete Change Survey			Email											
127	4	Mid- Questionnaire Battery	10	Mid	12	800	failed to complete CEQ Survey			Email											
128	5	Intervention Exposure / Active Control		Sleep Hygeine	11	10:00	Mid Survey Completed/Why Sleep Selected		Welcome to Next Phase	App	Intervention			Sleep Hygiene							
129	5	Intervention Exposure / Active Control		Sleep Hygeine	11	10:00			Today's Tasks	App	Intervention			Sleep Hygiene							
130	5	Intervention Exposure / Active Control		Sleep Hygeine	11	10:00			Sleep Hygeine	App	Intervention			Sleep Hygiene							
131	5	Intervention Exposure / Active Control		Sleep Hygeine	11	10:00			Sleep Hygeine FAQs	App	Intervention			Sleep Hygiene							
132	5	Intervention Exposure / Active Control		Sleep Hygeine	11	10:00	Why Sleep Selected		Why Sleep?	App	Intervention			Sleep Hygiene							
133	5	Intervention Exposure / Active Control		Sleep Hygeine	11	10:00	Why Sleep Completed - 48 hours - for next module		Why Sleep - Done	App	Intervention			Sleep Hygiene							
134	5	Intervention Exposure / Active Control		Sleep Hygeine	11	10:00			Log Sleep	App	Intervention		1	Sleep Hygiene							
135	5	Intervention Exposure / Active Control		Mindfullness	11	10:00	Mid Survey Completed/Mindfulness Selected		Welcome to Next Phase	App	Intervention			Mindfulness							
136	5	Intervention Exposure / Active Control		Mindfullness	11	10:00			Today's Tasks	App	Intervention			Mindfulness							
137	5	Intervention Exposure / Active Control		Mindfullness	11	10:00			Mindfulness	App	Intervention			Mindfulness							
138	5	Intervention Exposure / Active Control		Mindfullness	11	10:00			Mindfulness FAQs	App	Intervention			Mindfulness							
139	5	Intervention Exposure / Active Control		Mindfullness	11	10:00			Welcome to Mindfulness	App	Intervention			Mindfulness							
140	5	Intervention Exposure / Active Control		Mindfullness	11	10:00		Minfulness Video Play	Intro to Mindfulness Video	App	Intervention			Mindfulness							
141	5	Intervention Exposure / Active Control		Mindfullness	11	10:00			Mindfulness Log	App	Intervention		1	Mindfulness							
142	5	Intervention Exposure / Active Control		Physically Activity	11	10:00	Mid Survey Completed/Physical Activity Selected		Welcome to Next Phase	App	Intervention			Physical Activity							
143	5	Intervention Exposure / Active Control		Physically Activity	11	10:00			Physical Activity Program	App	Intervention			Physical Activity							
144	5	Intervention Exposure / Active Control		Physically Activity	11	10:00			Welcome to Physical Activity Program	App	Intervention			Physical Activity							
145	5	Intervention Exposure / Active Control		Physically Activity	11	10:00			Physical Activity FAQs	App	Intervention			Physical Activity							
146	5	Intervention Exposure / Active Control		Physically Activity	11	10:00		Welcome to Physical Activity	Today's Tasks	App	Intervention			Physical Activity							
147	5	Intervention Exposure / Active Control		Physically Activity	11	10:00		Increase Your Steps Selected	Increase Your Steps	App	Intervention			Physical Activity							
148	5	Intervention Exposure / Active Control		Physically Activity	11	10:00			Physical Activity Log	App	Intervention	1/2???		Physical Activity							
149	5	Intervention Exposure / Active Control	8	Control	11	10:00	72 hours			App	PANAS		12	EMA							
150	5	Intervention Exposure / Active Control	8	Control	11	10:00	72 hours			App	BHV		2	EMA							
151	5	Intervention Exposure / Active Control		Sleep Hygeine	12	10:00			Log Sleep	App	Intervention			Sleep Hygiene							
152	5	Intervention Exposure / Active Control		Mindfullness	12	10:00			Mindfulness Log	App	Intervention			Mindfulness							
153	5	Intervention Exposure / Active Control		Physically Activity	12	10:00			Physical Activity Log	App	Intervention			Physical Activity							
154	5	Intervention Exposure / Active Control	8	Control	12	10:00	60 min			App	PANAS		12	EMA				Positive and neg	Intervention Phase/Control		
155	5	Intervention Exposure / Active Control	8	Control	12	10:00	60 min			App	BHV		2	EMA				Coping behavior	Intervention Phase/Control + Int Group		
156	5	Intervention Exposure / Active Control		Sleep Hygeine	13	10:00			Log Sleep Hours	App	Intervention			Sleep Hygiene	2			Psychological di	Intervention Phase/Control		
157	5	Intervention Exposure / Active Control		Mindfullness	13	10:00			Mindfulness Log	App	Intervention			Mindfulness	2			General mental v	Intervention Phase/Control + Int Group		
158	5	Intervention Exposure / Active Control		Physically Activity	13	10:00			Physical Activity Log	App	Intervention			Physical Activity	2			Positive and neg	Intervention Phase/Control		Control group only
159	5	Intervention Exposure / Active Control	8	Control	13	10:00	60 min			App	PANAS		12	EMA				Response to affe	Intervention Phase/Control + Int G		Control group only
160	5	Intervention Exposure / Active Control	8	Control	13	10:00	60 min			App	BHV		2	EMA				Positive and neg	Intervention Phase/Control		
161	5	Intervention Exposure / Active Control		Intervention	13	20:00	failed to complete intervention intro module			SMS							11 days left to learn practical ways to improve your wellbeing. Great job on your progress so far, [firstname]. Open Vibe Up to keep going.				
162	5	Intervention Exposure / Active Control	8	Control	13	2000	failed to complete EMA			SMS							Time to start Part 2 of the study. Great job on your progress so far, [firstname]. Open Vibe Up to keep going! ☺				
163	5	Intervention Exposure / Active Control		Sleep Hygeine	14	10:00	Why Sleep Completed - accessible after 48 hours		Sleep Habits	App	Intervention			Sleep Hygiene							
164	5	Intervention Exposure / Active Control		Sleep Hygeine	14	10:00			Sleep Habits - Done	App	Intervention			Mindfulness							
165	5	Intervention Exposure / Active Control		Sleep Hygeine	14	10:00			Log Sleep	App	Intervention		1	Physical Activity							
166	5	Intervention Exposure / Active Control		Mindfullness	14	10:00	Mindfulness Video Watched - 24 hours - for next module		Welcome to Mindfulness - Start	App	Intervention										
167	5	Intervention Exposure / Active Control		Mindfullness	14	10:00	Welcome to Mindfulness - START		Mindful	App	Intervention										
168	5	Intervention Exposure / Active Control		Mindfullness	14	10:00			Mindfulness Log	App	Intervention		1								
169	5	Intervention Exposure / Active Control		Physically Activity	14	10:00		Welcome to Physical Activity	Today's Tasks	App	Intervention										
170	5	Intervention Exposure / Active Control		Physically Activity	14	10:00		7-minute Workout Selected	7-minute Workout	App	Intervention										
171	5	Intervention Exposure / Active Control		Physically Activity	14	10:00			Physical Activity Log	App	Intervention		1/2?								
172	5	Intervention Exposure / Active Control	8	Control	14	10:00	60 min			App				EMA				Positive and neg	Intervention Phase/Control		
173	5	Intervention Exposure / Active Control	8	Control	14	10:00	60 min			App				EMA				Response to affe	Intervention Phase/Control + Int Group		
174	5	Intervention Exposure / Active Control		Sleep Hygeine	15	10:00			Log Sleep Hours	App	Intervention		1								
175	5	Intervention Exposure / Active Control		Mindfullness	15	10:00			Mindfulness Log	App	Intervention		1								
176	5	Intervention Exposure / Active Control		Physically Activity	15	10:00			Physical Activity Log	App	Intervention		1-2								
177	5	Intervention Exposure / Active Control	8	Control	15	10:00	60 min			App				EMA				Response to affe	Intervention Phase/Control + Int Group		
178	5	Intervention Exposure / Active Control	8	Control	15	10:00	60 min			App				EMA				Positive and neg	Intervention Phase/Control		
179	5	Intervention Exposure / Active Control		Intervention	15	800	failed to complete intervention intro module			Email							TBC				
180	5	Intervention Exposure / Active Control	8	Control	15	800	failed to complete EMA			Email							TBC				
181	5	Intervention Exposure / Active Control		Intervention	15	20:00	failed to interact with app - 4 days after completion of intervention intro			SMS							We've missed you the past few days Jump back into the Vibe Up!				
182	5	Intervention Exposure / Active Control		Sleep Hygeine	16	10:00			Log Sleep Hours	App	Intervention		1								
183	5	Intervention Exposure / Active Control		Mindfullness	16	10:00			Mindfulness Log	App	Intervention		1								
184	5	Intervention Exposure / Active Control		Physically Activity	16	10:00			Physical Activity Log	App	Intervention		1-2								
185	5	Intervention Exposure / Active Control	8	Control	16	10:00	60 min			App				EMA				Positive and neg	Intervention Phase/Control		
186	5	Intervention Exposure / Active Control	8	Control	16	10:00	60 min			App				EMA				Response to affe	Intervention Phase/Control + Int Group		

2451

2452

187	5	Intervention Exposure / Active Control		Intervention	16	800	failed to interact with app after completion of intro			Email				TBC		
188	5	Intervention Exposure / Active Control		Sleep Hygeine	17	10:00	Why Sleep Completed - accessible after 48 hours		Sleep Environment	App	Intervention					
189	5	Intervention Exposure / Active Control		Sleep Hygeine	17	10:00			Sleep Environment - Done	App	Intervention					
190	5	Intervention Exposure / Active Control		Sleep Hygeine	17	10:00			Log Sleep	App	Intervention	1				
191	5	Intervention Exposure / Active Control		Mindfullness	17	10:00	Mindfulness Video Watched - 24 hours - for next module		Welcome to Mindfullness - Start	App	Intervention					
192	5	Intervention Exposure / Active Control		Mindfullness	17	10:00		Welcome to Mindfulness - START	Unhooking from Thoughts	App	Intervention					
193	5	Intervention Exposure / Active Control		Mindfullness	17	10:00			Mindfulness Log	App	Intervention	1				
194	5	Intervention Exposure / Active Control		Physically Activity	17	10:00		Welcome to Physical Activity	Today's Tasks	App	Intervention					
195	5	Intervention Exposure / Active Control		Physically Activity	17	10:00		Other Activity Selected	Other Activity	App	Intervention					
196	5	Intervention Exposure / Active Control		Physically Activity	17	10:00			Physical Activity Log	App	Intervention	1-2				
197	5	Intervention Exposure / Active Control	8	Control	17		60 min			App			EMA			
198	5	Intervention Exposure / Active Control	8	Control	17		60 min			App			EMA			
199	5	Intervention Exposure / Active Control		Sleep Hygeine	18	10:00			Log Sleep Hours	App	Intervention	1				
200	5	Intervention Exposure / Active Control		Mindfullness	18	10:00			Mindfulness Log	App	Intervention	1				
201	5	Intervention Exposure / Active Control		Physically Activity	18	10:00			Physical Activity Log	App	Intervention	1-2				
202	5	Intervention Exposure / Active Control	8	Control	18		60 min			App			EMA			
203	5	Intervention Exposure / Active Control	8	Control	18		60 min			App			EMA			
204	5	Intervention Exposure / Active Control		Sleep Hygeine	19	10:00			Log Sleep Hours	App	Intervention	1				
205	5	Intervention Exposure / Active Control		Mindfullness	19	10:00			Mindfulness Log	App	Intervention	1				
206	5	Intervention Exposure / Active Control		Physically Activity	19	10:00			Physical Activity Log	App	Intervention	1-2				
207	5	Intervention Exposure / Active Control	8	Control	19		60 min			App			EMA			
208	5	Intervention Exposure / Active Control	8	Control	19		60 min			App			EMA			
209	5	Intervention Exposure / Active Control		Sleep Hygeine	20	10:00				Log Sleep Hours	App	Intervention				
210	5	Intervention Exposure / Active Control		Mindfullness	20	10:00	Mindfulness Video Watched - 24 hours - for next module		Welcome to Mindfullness - Start	App	Intervention					
211	5	Intervention Exposure / Active Control		Mindfullness	20	10:00		Welcome to Mindfulness - START	Body Scan	App	Intervention					
212	5	Intervention Exposure / Active Control		Mindfullness	20	10:00			Mindfulness Log	App	Intervention	1				
213	5	Intervention Exposure / Active Control		Physically Activity	20	10:00		Welcome to Physical Activity	Today's Tasks	App	Intervention					
214	5	Intervention Exposure / Active Control		Physically Activity	20	10:00		Rest Day Selected	Rest Day	App	Intervention					
215	5	Intervention Exposure / Active Control		Physically Activity	20	10:00			Physical Activity Log	App	Intervention	1-2???				
216	5	Intervention Exposure / Active Control	8	Control	20		60 min			App			EMA			
217	5	Intervention Exposure / Active Control	8	Control	20		60 min			App			EMA			
218	5	Intervention Exposure / Active Control		Sleep Hygeine	21	10:00	Why Sleep Completed - 48 hours - for next module		Daily Activities for Sleep	App	Intervention					
219	5	Intervention Exposure / Active Control		Sleep Hygeine	21	10:00			Daily Activities for Sleep - Done	App	Intervention					
220	5	Intervention Exposure / Active Control		Sleep Hygeine	21	10:00			Log Sleep	App	Intervention	1				
221	5	Intervention Exposure / Active Control		Mindfullness	21	10:00			Mindfulness Log	App	Intervention	1				
222	5	Intervention Exposure / Active Control		Physically Activity	21	10:00			Physical Activity Log	App	Intervention	1-2?				
223	5	Intervention Exposure / Active Control	8	Control	21		60 min			App			EMA			
224	5	Intervention Exposure / Active Control	8	Control	21		60 min			App			EMA			
225	5	Intervention Exposure / Active Control		Sleep Hygeine	22	10:00			Log Sleep Hours	App	Intervention	1				
226	5	Intervention Exposure / Active Control		Mindfullness	22	10:00			Mindfulness Log	App	Intervention	1				
227	5	Intervention Exposure / Active Control		Physically Activity	22	10:00			Physical Activity Log	App	Intervention	1-2?				
228	5	Intervention Exposure / Active Control	8	Control	22		60 min			App		\	EMA			
229	5	Intervention Exposure / Active Control	8	Control	22		60 min			App			EMA			
230	5	Intervention Exposure / Active Control		Sleep Hygeine	23	10:00			Log Sleep Hours	App	Intervention	1				
231	5	Intervention Exposure / Active Control		Mindfullness	23	10:00	Mindfulness Video Watched - 24 hours - for next module		Welcome to Mindfullness - Start	App	Intervention					
232	5	Intervention Exposure / Active Control		Mindfullness	23	10:00		Welcome to Mindfulness - START	Daily Activities for Sleep	App	Intervention					
233	5	Intervention Exposure / Active Control		Mindfullness	23	10:00			Mindfulness Log	App	Intervention	1				
234	5	Intervention Exposure / Active Control		Physically Activity	23	10:00			Welcome to Mindfullness - Start	App	Intervention					
235	5	Intervention Exposure / Active Control		Physically Activity	23	10:00		Welcome to Physical Activity	Daily Activities for Sleep	App	Intervention					
236	5	Intervention Exposure / Active Control		Physically Activity	23	10:00			Physical Activity Log	App	Intervention	1-2?				
237	5	Intervention Exposure / Active Control	8	Control	23		60 min			App			EMA			
238	5	Intervention Exposure / Active Control	8	Control	23		60 min			App			EMA			
239	5	Intervention Exposure / Active Control		Sleep Hygeine	24	10:00			Log Sleep Hours	App	Intervention	1				
240	5	Intervention Exposure / Active Control		Mindfullness	24	10:00			Mindfulness Log	App	Intervention	1				
241	5	Intervention Exposure / Active Control		Physically Activity	24	10:00			Physical Activity Log	App	Intervention	1				
242	5	Intervention Exposure / Active Control	8	Control	24		60 min			App			EMA			
243	5	Intervention Exposure / Active Control	8	Control	24		60 min			App			EMA			
244				Post	25	800	72 hours			App				Almost there! Do your final survey to tell us how you're going 📋		
245	6	Post- Questionnaire Battery	6	Mood Survey	25			Mood Survey Selected -	Mood Survey	App						
246	6	Post- Questionnaire Battery			25	10:00	72 hours			App			DASS-21		Psychological di:	Exit Phase
247	6	Post- Questionnaire Battery	6	Mood Survey	25		Close survey		Close the	App						
248	6	Post- Questionnaire Battery	6	Mood Survey	25		Mood Survey Completed		Well Done/Home Button	App						
249	6	Post- Questionnaire Battery	7	Well Being Survey	25			Well Being Survey Selected -	Well Being Survey	App						
250	6	Post- Questionnaire Battery			25	10:00	72 hours			App			PAVS		General mental \	Exit Phase
251	6	Post- Questionnaire Battery			25	10:00	72 hours			App			PSQI		Physical wellbeir	Exit Phase
252	6	Post- Questionnaire Battery			25	10:00	72 hours			App			MIND		Mindfulness sing	Exit Phase
253	6	Pre-Questionnaire Battery (Baseline)	7	Well Being Survey	25		Wellbeing Survey Completed		Well Done/Home Button	App						
254	6	Post- Questionnaire Battery		Disruptions Report	25			Disruptions Survey Selected -	Disruptions Survey	App						
255	6	Post- Questionnaire Battery			25	10:00	72 hours			App		4	EXP		Within-study exp	Exit Phase
256	6	Post- Questionnaire Battery		Disruptions Report	25		Disruptions Survey Completed		Well Done/Home Button	App						
257	6	Post- Questionnaire Battery		Your Feedback	25			Your Feedback Selected -	Your Feedback	App						
258	6	Post- Questionnaire Battery			25	10:00	72 hours			App		9	UX		UX questionnair	Exit Phase

2453

2454

259	6	Post- Questionnaire Battery	Your Feedback	25		Your feedback Completed	Well Done/Home Button	App							
260	6	Post- Questionnaire Battery	Post	26	20:00	failed to complete questionnaire		SMS						📢 Answer the final survey to finish the study and get your gift card 🎁<br>The survey expires at midnight tomorrow!	
261	7	End of Study		26		Upon completion of post questionnaires		SMS			Finish			Congrats on finishing the study!	To complete Baseline, Mid, Post
262			Post	27	800	failed to complete questionnaire		Email						TBC	
263				27		72 hours									To complete Baseline, Mid, Post
264				28		72 hours									To complete Baseline, Mid, Post
265				29		72 hours									To complete Baseline, Mid, Post
266				30							All Interventions				

2455

Cell: A26
Note: Might want to make this =above+1, but the tradeoff is that the ids will no longer be fixed if rows are added in between
-Leonard Hoon

Cell: J54
Note: Not sure.
-Rena Logothetis

Cell: J74
Note: Is it repeatedly shown?
-Rena Logothetis

Cell: M238
Note: Do we collect if it is rest day?
-Rena Logothetis
Good question to raise during tomorrow's stand up
-Leonard Hoon

2458
2459
Appendix 30 Screening and Self-Report Questionnaire Testing Plan

Vibe Up Screening and Self-Report Questionnaire Testing Plan

Version dated: 12 Mar 2021

1. Purpose

To describe the principles that will guide testing of the self-report questionnaires incorporated across the Vibe Up Study.

Because self-report is the primary mechanism for collecting outcomes and experience data from participants, and is the sole mechanism for collecting the primary endpoint (DASS-21), it is particularly important that the questionnaire data collection process operates as intended.

2. Scope

The plan applies to all questionnaires used at web-based consent/screening and those embedded in the Vibe Up app. It addresses testing for standard instruments, bespoke questionnaires and ecological momentary assessments.

The following systems will need to be examined during testing:

System	Elements
Qualtrics	Consent, screening and demographic questionnaire implementation (user-facing)
Vibe Up App	Baseline, mid and post self-reported outcome questionnaire implementations (user-facing)
Optimise Cloud System	Persisted questionnaire responses and calculated scores (not user-facing)

3. Testing principles

3.1 Testing must be conducted according to a pre-specified test plan.

In order to ensure that all elements of the app are appropriately tested, a plan should be devised prior to commencing testing.

Users asked to take part in testing, whether internal or external, should be given clear, simple guidance about:

- What they need to do. If specific tasks must be completed or sequenced, these should be presented in a test script (see **Appendix 1**). A test script is particularly important if there are aspects of the product that may be neglected without specific pointers, where things must be done at specific times; or where there are multiple different testing permutations that must be explicitly assigned to different users in order to ensure coverage.
- What to look for; including any expected external effects of using the product (e.g. notifications/reminders); and
- How to report bugs effectively so that issues can be tracked down efficiently.

3.2 The test plan/scripts should explicitly address high-risk items.

A high-risk item is any feature or behaviour of a questionnaire that:

- Is critical to its operation; and/or
- Affects the conduct of the overall study; and/or
- would pose a safety, privacy or reputational risk should it fail.

2460

An example of a high risk item is the calculation of a summary score that is used to drive a key study or intervention element.

3.3 Testing must assess the data pipeline from end to end.

The testing process should provide assurance that the *right data* are being collected, in the *right form* and that these are *captured and persisted accurately*. Examples might include ensuring that data entered into a web form are correctly persisted when that page is refreshed, or that user responses to a questionnaire are stored correctly in a back-end database.

Data types that should be evaluated might include:
- Data supplied by a user (e.g. questionnaire responses);
- Data generated about a user (e.g. analytics data); and
- Metadata (e.g. timestamps generated when data are created.)

3.4 Testing should be conducted by trained users.

The process of comparing the design and function of the product against requirements should be performed by staff who have a deep understanding of how the product is expected to behave (e.g. the product or working group lead).

It may be appropriate to involve other staff, e.g. UX or marcomms to evaluate specific aspects of the product.

3.5 Testing should ensure that all aspects of the product are evaluated in real-world conditions.

The number of people required to test each product will depend on its complexity; the amount of testing each user can be expected to (or does) perform; the heterogeneity of the environment in which the product will run (see 3.7 below); and the surface area for risk.

If the product has multiple basic configuration states (for example, a set of functions that differ if a user is randomized to an intervention or opts in to some study component) then these should be explicitly mapped out, all the valid combinations identified and - if feasible - users assigned to test each of these.

3.6 Testing must capture a realistic spread of users and device types.

- The most popular desktop browsers by market share in Australia are: Chrome (62%), Safari (18%), Edge (9%) and Firefox (6%).
- The most popular mobile browsers by market share in Australia are: Mobile Safari (49%), Mobile Chrome (39%), and Samsung Internet (8%).
- The most popular desktop screen resolutions in Australia are: 1920×1080 (24%), 1366×768 (13%), 1440×900 (12%), 1536×864 (7%), 1280×720 (7%), and 1024×768 (6%).
- The Vibe Up App was designed to support the iPhone 6S running iOS 13 or later, and any device Android 5 or later.
- The most popular mobile screen resolutions in Australia are: 414×896 (14%), 375×812 (11%), 375×667 (11%), 414×736 (5%), 360×640 (5%) and 360×780 (5%).

3. Testing goals

Questionnaire testing in the Vibe Up study has the following goals:

2461

2462

1. To ensure that the wording and presentation, including ordering, of question/answer options is correct and free from spelling and other typographical errors, by comparing questions against their reference specification (see **Appendix 8** of the **Clinical Trial Protocol**).
2. To ensure that presentation is consistent and appropriate across different device types and screen layouts, including small form factor devices with limited resolution.
3. To ensure that users can navigate and interact with all questions appropriately, including being able to provide any valid input for any given question.
4. To verify that individual item responses are persisted correctly so that they can be extracted for subsequent analysis.
5. To verify that, if implemented for a specific questionnaire, any automatic calculation of score (e.g. the DASS-21 total score consumed by the Vibe Up algorithm) is correct.
6. To verify that the design of the questionnaires is consistent with the BDI brand and Vibe Up graphic design.

4. Testing approach

To avoid issues being missed, assessment should be structured as a series of test rounds focussed on specific goals. Rounds may run concurrently if not being performed by the same person.

Round	Focus	Related Goals
Round 1	Content and wording	1 and 2
Round 2	Functionality and reporting	3, 4 and 5
Round 3	Styling	6

For each round and questionnaire that is to be tested, a structured test script should be created based on the template provided in **Appendix 1**. The test script should provide a structured, repeatable series of steps to follow and allow identified issues to be captured/described clearly.

Issues identified through testing will be aggregated and reported to the software/design team as required.

Version history

Date	Summary of changes
12 Mar 2021	Document created.

2463

2464

Appendix 1 – Test script template

**Tester Id: (Mobile number)**	
**Questionnaire:**	
**Test Round/Focus:**	

**Device type:**	
**Device model (smartphones/tablets):**	
**Operating system and version:**	
**Web browser and version (if relevant):**	

Script prepared by test team				Steps completed by tester		
#	When	Instruction	Expected outcome	Pass/Fail	Remarks (including steps to trigger/reproduce any bug)	Screenshots (if needed)
0		Setup instructions here...				
1		First instruction here...				
2		Second instruction here...				
3		...				
4						
5						
6						
7						
8						
...						

What to do if you think you've found a bug...

- Use the Remarks column to describe what the bug is.
- Tell us what you were doing immediately before the bug happened.
- If possible, describe the steps that can trigger the bug.
- Take a screenshot, if possible/relevant.
- Make sure you've recorded your device type, operating system and web browser version.

How to take a screenshot:

- If you are using UserSnap, then a screenshot is taken automatically and you don't need to do anything.
- In Windows, click **Start** then type '**snip**' and choose the **Snipping Tool**. Once the tool has opened, click **New** to select an area of the screen to capture as a screenshot.

Page 4 of 5

HC Number: HC200466 **Version dated:** 12 Mar 2021

2465

- In Mac OS, simultaneously press the keys **Command + Shift + 3**. The screenshot will be saved on your desktop as a PNG file.
- On an iPhone with a home button, simultaneously press the **home button** and the **power button**. The screenshot will open in a popup in the bottom left of your screen. Tap this to save or send by email.
- On an iPhone without a home button, simultaneously press the **volume up button** and the **power button**. The screenshot will open in a popup in the bottom left of your screen. Tap this to save or send by email.
- On an Android phone, press and hold the **power button** for a few seconds. Then tap **Screenshot**. If that doesn't work, press and hold the **power button** and **volume down button** at the same time for a few seconds. The screenshot will appear in a popdown at the top of the screen. Swipe down to save or send by email.

How to determine your device's operating system, version and model:

- In Windows, press **Start** and type **'System Information'**. Click to run the **System Information** tool and copy the information shown in the **OS Name** and **Version** fields.
- In Mac OS, click the **Apple** icon in the top left corner of your screen and choose **About this Mac**. OS and Version information is shown in the middle of the window that appears.
- On an iPhone, open the **Settings** app, scroll down and tap **General** and then **About**. The version of iOS (the iPhone's operating system) is shown under **Version** and the device model is shown under the **Model** field.
- On an Android phone, open the **Settings** app, scroll down to the bottom and tap **About phone**. The version of Android is shown under **Android Version** and the device model is shown under the **Model** field.

How to determine your web browser version:

- You don't need to get the version if you're using a mobile browser.
- In Google Chrome for Desktop, type '**chrome://chrome**' in the address bar. The version number is shown in the top panel.
- In Microsoft Edge, choose the **...** icon and then **Settings**. Scroll down to the bottom. The version is listed under **About this app**.
- In Safari for Desktop, choose the **Safari** menu and then **About Safari**. The version number will be shown in the window that appears.
- In Microsoft Internet Explorer, choose the ⚙ **icon** and then **About Internet Explorer**. The version number will be shown in the window that appears.
- In Firefox for Desktop, choose ☰ and then **Help** and then **About Firefox**. The version number will be shown in the window that appears.

Appendix 31 Systems Incident Identification and Response Protocol

A²I²

Systems incident identification and response protocol

Date: 08 February 2021

Version: 1.1

Prepared by: Deakin University
Applied Artificial Intelligence Institute

a2i2.deakin.edu.au

Deakin University CRICOS Provider Code: 00113B

Commercial in Confidence

Document History

Version	Notes	Date
1.0	Initial version	08/02/2021

Commercial in Confidence

Table of Contents

1. Introduction **4**
- 1.1. Cyber incidents monitoring 4
- 1.2. Data Breach vs Security Vulnerability 4
  - 1.2.1. Data Breach Examples 4
  - 1.2.2. Security Vulnerability Examples 5
- 1.3. About the DBRP 5

2. Continuous monitoring **5**
- 2.1. Types of monitoring 5
- 2.2. Plan of action and milestones 5
  - 2.2.1. Security Assessment Report 6

3. Data Breach Identification/Assessment **7**
- 3.1. Data Breach Identification Process 7
- 3.2. Issue Assessment Process 8
- 3.3. Identification Vectors 8
  - 3.3.1. Security Vulnerabilities 8
  - 3.3.2. Data Breach 9

4. Communication Strategy **9**
- 4.1. Slack 9
- 4.2. Email 9
- 4.3. Google Drive 10

5. Response **10**
- 5.1. Escalation Procedure 10
- 5.2. Incident Response 11

6. Roles and Responsibilities **11**
- 6.1. Roles 11
  - 6.1.1. Engineering Cohort Steering Group (STG) 11
  - 6.1.2. Engineering Cohort Security Group (ECSG) 12
  - 6.1.3. Response Team 12
  - 6.1.4. General Manager Software Engineering 12
  - 6.1.5. Co-Director 12
  - 6.1.6. A^2I^2 Team Members 13
- 6.2. Responsibilities Matrix 13

7. Documentation **15**
- 7.1. Security Incident Log 15
- 7.2. Security Incident Report 15

Page 2

2474

8. Post-Incident Review **15**

9. Appendix **16**

9.1. Escalation Email Template 16

10. Terminology **17**

10.1. Cyber security events 17

10.2. Cyber security incidents 17

11. References **17**

2475

Commercial in Confidence

1. Introduction

Information present in this document has been heavily influenced by the referred sources. Modifications to the aforementioned guidelines include Conductor specific security measures.

Use of the pronouns "us", "our" and "we" anywhere in this document refers to A^2I^2.

The Data Breach Response Plan (DBRP) assists A^2I^2 to prepare for and respond to data breaches in/and related to the software it ships. According to the Australian Government's Office of the Australian Information Commissioner (OAIC), a data breach is, "unauthorised access or disclosure of personal information, or loss of personal information." Personal information is information about a known or identifiable individual and is attractive to criminals who use it for financial fraud, identity theft, and other crimes.

1.1. Cyber incidents monitoring

A continuous monitoring plan will assist us in proactively identifying, prioritising and responding to security vulnerabilities. Measures to monitor and manage security vulnerabilities in systems can also provide us with a wealth of valuable information about their exposure to cyber threats, as well as assisting them to determine security risks associated with the operation of our systems.

1.2. Data Breach vs Security Vulnerability

There is a critical distinction to be made between a security vulnerability and a data breach. A security vulnerability is a defect in the software, hardware, or business processes (e.g. social engineering) that an attacker can exploit to gain unauthorised access to a system and its data. A data breach is an example of a possible side effect that can arise from a security vulnerability.

Security vulnerabilities can occur in first-party (software developed in-house) and third-party (prerequisite companion software) applications.

Collectively data breaches and verified security vulnerabilities found in Institute software will be referred to as security incidents.

1.2.1. Data Breach Examples

Examples of data breaches include:

- "loss or theft of physical devices (such as laptops and storage devices) or paper records that contain personal information."[1]
- "unauthorised access to personal information by an employee."[2]

[1] https://www.oaic.gov.au/privacy/guidance-and-advice/data-breach-preparation-and-response/
[2] Ibid.

Page 4

- "inadvertent disclosure of personal information due to 'human error', for example[,] an email sent to the wrong person."[3]
- "[the] disclosure of an individual's personal information to a scammer, as a result of inadequate identity verification procedures."[4]

1.2.2. Security Vulnerability Examples

Examples of security vulnerabilities include:

- Poor business processes surrounding the protection of private information that can lead to attacks, E.g. social engineering (https://en.wikipedia.org/wiki/Social_engineering_(security)).
- A software defect that can be used to gain unauthorised access to a system, E.g. a zero-day exploit (https://en.wikipedia.org/wiki/Zero-day_(computing)).
- A hardware feature that can be exploited to gain unauthorised access to a system, E.g. "Row hammer" (https://en.wikipedia.org/wiki/Row_hammer).

1.3. About the DBRP

The DBRP is not an official/legally binding document. It is an internal tool that provides baseline guidance on steps to follow when team members receive communication about or identify a data breach. It does not replace Deakin University's INFORMATION AND COMMUNICATIONS TECHNOLOGY SECURITY POLICY or PRIVACY POLICY (see https://policy.deakin.edu.au).

2. Continuous monitoring

Undertaking continuous monitoring activities is important as cyber threats and the effectiveness of security controls will change over time.

2.1. Types of monitoring

Three types of continuous monitoring activities are vulnerability assessments, vulnerability scans and penetration tests. A vulnerability assessment typically consists of a review of a system's architecture or an in-depth hands-on assessment while a vulnerability scan involves using software tools to conduct automated scans. In each case, the goal is to identify as many security vulnerabilities as possible. A penetration test however is designed to exercise real-world targeted cyber intrusion scenarios in an attempt to achieve a specific goal, such as compromising critical system components or information.

2.2. Plan of action and milestones

[3] Ibid.
[4] Ibid.

Many potential cyber security incidents are noticed by personnel rather than software tools. As such, successful detection of cyber security incidents is often based around trained cyber security personnel with access to sufficient data sources complemented by tools supporting both manual and automated analysis.

The required cyber security personnel (internal to our organisation or a third party) will be provisioned by Deakin, who will have access to sufficient data sources and tools to ensure that any security alerts generated by systems are investigated and that systems and data sources are able to be searched for key indicators of compromise including but not limited to IP addresses, domains and file hashes.

The following activities will be conducted by the appointed cyber security personnel to ensure that there is no conflict of interest, perceived or otherwise, and that the activities are undertaken in an objective manner:

- Conducting vulnerability assessments or penetration tests for systems to produce a Security Assessment Report annually.
- Conducting vulnerability scans for systems monthly to be actioned and communicated if vulnerabilities are detected.
    - In the case that a vulnerability is detected, analysis will commence investigating their potential impact and appropriate mitigations based on effectiveness, cost and existing security controls, to produce recommendations for the Data Management Committee to prioritise using a risk-based approach for implementation.

2.2.1. Security Assessment Report

At the annual conclusion of a security assessment for a system, a security assessment report will be produced by the assessor. This will assist the system owner in performing any initial remediation actions as well as guiding the development of the system's plan of action and milestones.

This assessment report will cover:

- the scope of the security assessment
- the system's strengths and weaknesses
- security risks associated with the operation of the system
- the effectiveness of the implementation of security controls any recommended remediation actions.

Commercial in Confidence

3. Data Breach Identification/Assessment

If a team member identifies a potential security vulnerability in deployed software (software running in a Development, QA, or Production environment) or receives internal (Deakin)/external communication about a known data breach, they should follow these steps:

- Take note of the pertinent details and notify their immediate team lead or the most senior member of the project team. If a suitable individual is not available, they should post a message on Slack in the "engineering-cohort" channel for the attention of the Engineering Cohort Security Group (AKA. FA-DevSecOps) ("@fa-devsecops-steering-group").
- If they receive no response, use the same steps to follow up until they have an acknowledgement of the data breach from a senior team member.

3.1. Data Breach Identification Process

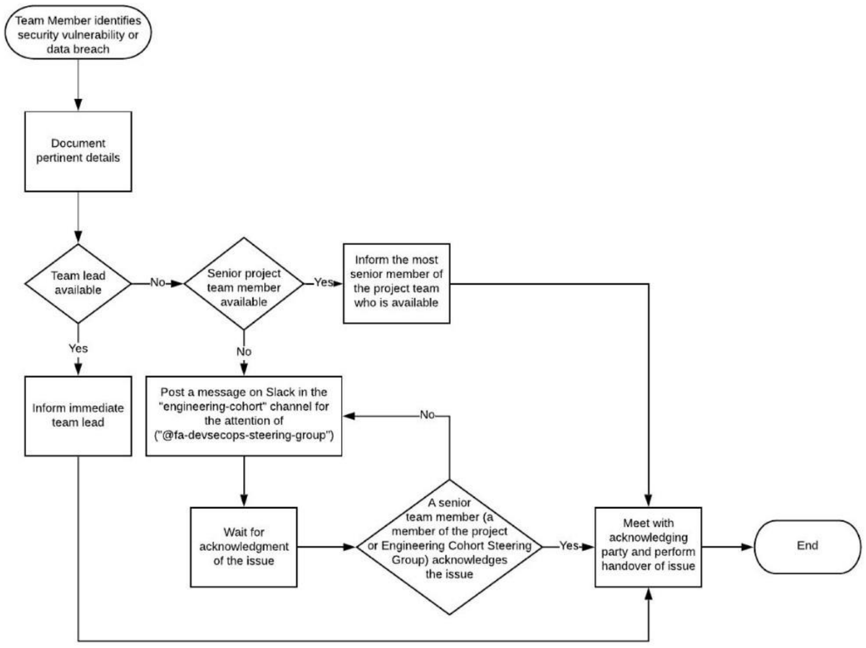

3.2. Issue Assessment Process

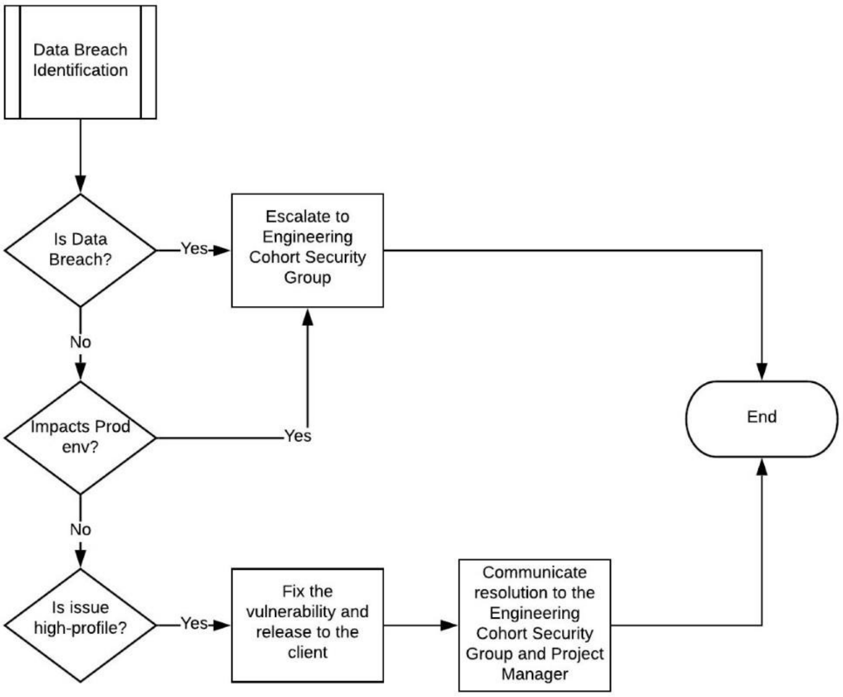

3.3. Identification Vectors

Team members can learn about security vulnerabilities and data breaches in many ways. The sub-sections below list some examples.

3.3.1. Security Vulnerabilities

- Third-party email communications
- Source-code review
- Failed Unit/Integration Tests
- Linters
- Television
- Podcasts
- Newspaper articles

2486

- Internet forums
- Meet-Ups
- Vulnerabilities scanners

3.3.2. Data Breach

- Third-party email communications
- Television
- Podcasts
- Newspaper articles
- Internet forums
- Meet-Ups
- Written confession

4. Communication Strategy

The primary mechanisms for communicating incidents within the Institute include Slack, email, and Google Drive.

4.1. Slack

Slack will be used to report actual or potential data breaches and security vulnerabilities.
Team members should use the following channels throughout the incident management lifecycle:

- #engineering-cohort
  - When a team member is unable to initially report a data breach or security vulnerability to a senior team member in person, they should do so in this channel.
- #ec-steering-group
  - The Engineering Cohort Steering Group (STG) should conduct general discussions about a security incident in this channel.

Team members should include the following user group when an incident is reported or mentioned in one of the relevant Slack channels to ensure members of the Engineering Cohort Security Group receive a Slack notification:

- @fa-devsecops-steering-group
  - This user group includes members of the Engineering Cohort Security Group.

4.2. Email

The incident escalation process requires email communication. The Response Team will formally notify a Co-Director via email when:

- An incident involves a data breach.
- A security vulnerability has a high profile.
- The likelihood of a data breach is imminent.

2487

2488

The "Escalation Email Template" (see appendix) can be used as a guide when creating this email.

If the Response Team has determined that Deakin ICT needs to be informed about an incident, the General Manager Software Engineering or a Co-Director can do so via email.

The "Escalation Email Template" (see appendix) can be used as a guide when creating this email.

4.3. Google Drive

The Security Incident Log is hosted on Google Drive (here).

The Security Incident Report (SIR) associated with an incident investigation should be created using the "A^2I^2 - Template - Security Incident Report" Google Docs template.

All SIRs and related incident investigation artefacts belong in a folder on the "A^2I^2" Shared Drive.

5. Response

5.1. Escalation Procedure

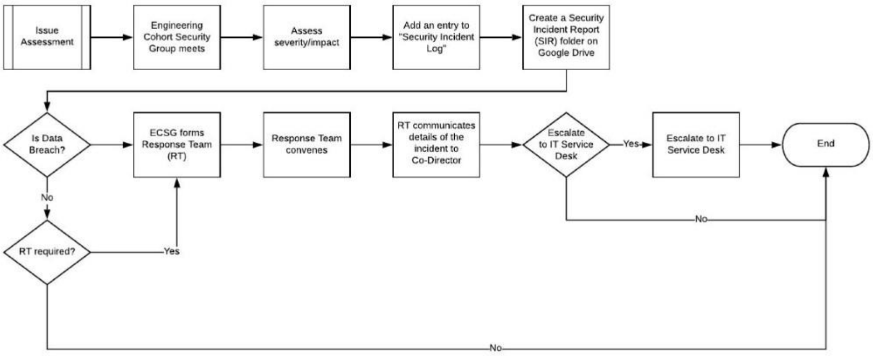

- If the Response Team agrees that it is required the incident will be escalated to the Director, ICT Infrastructure Services or nominee via the IT Service Desk as per the Deakin University "INFORMATION AND COMMUNICATIONS TECHNOLOGY SECURITY PROCEDURE"/"INFORMATION AND COMMUNICATIONS TECHNOLOGY SECURITY POLICY".

2489

Commercial in Confidence

5.2. Incident Response

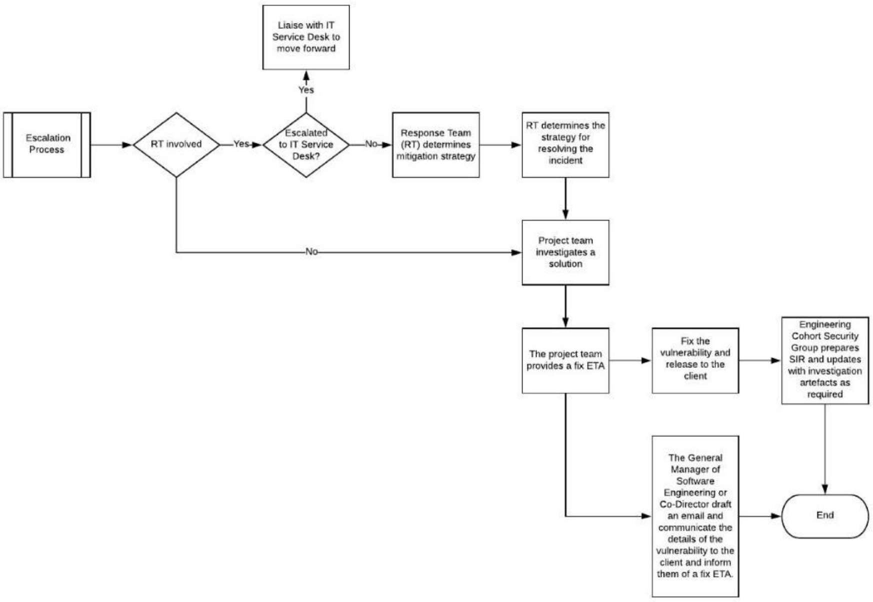

6. Roles and Responsibilities

6.1. Roles

6.1.1. Engineering Cohort Steering Group (STG)

"The Engineering Cohort is led by a steering group, which is a blend of people (experience and role) across the organisation that represent a range of interests relevant to the Engineering Cohort. The group will be composed predominantly of senior members of the group who will be responsible for decision-making, but other group members are expected to have a voice and be treated with the respect of a participating member through direct involvement in the activities of the steering committee."[5]

[5] https://docs.google.com/document/d/11dzEuEEcfppppnGf_jeTMphFMQRNEzA7VVReaV0vsb8/edit#

6.1.2. Engineering Cohort Security Group (ECSG)

The Engineering Cohort Security Group (also known as FA-DevSecOps) is a group of individuals from the Engineering Cohort that focus their attention on the betterment of cybersecurity capability and education across the Institute. The "General Manager Software Engineering" is an automatic member.

The group members can be reached on Slack using the "@fa-devsecops-steering-group" user group.

6.1.3. Response Team

The data breach Response Team is responsible for carrying out actions that can reduce the potential impact of a data breach.

The Baseline Response Team consists of the mandatory individuals required to respond to a security incident. The Extended Response Team includes all members of the Baseline Response Team as well as additional parties that may be necessary depending on the circumstances/severity of a data breach.

The Baseline Response Team will include the following groups and individuals:

- Representatives from the Engineering Cohort Security Group
- General Manager Software Engineering
- The Product Manager of the software affected
- The team member who discovered the issue

The Extended Response Team may also include the following additional groups and individuals:

- External experts, E.g. Deakin ICT
- Legal support — to identify legal obligations and provide advice
- Risk management support — to assess the risks from the breach
- Human resources (HR) support — if the breach was due to the actions of a staff member
- External data forensics experts
- University Media Consultants

6.1.4. General Manager Software Engineering

The A^2I^2 General Manager Software Engineering is responsible for the management and allocation of Institute software engineering oversight and team members.

The current General Manager is Mr Allan Jones.

6.1.5. Co-Director

There are two A^2I^2 Co-Directors. The Co-Directors are the leaders of the Institute.

The current Co-Directors are Professor Svetha Venkatesh and Professor Kon Mouzakis.

6.1.6. A^2I^2 Team Members

Any A^2I^2 software engineer can report possible data breaches or security vulnerabilities. Other institute staff are also encouraged to communicate news of potential threats they have learned about through media or other sources.

6.2. Responsibilities Matrix

Role	Responsibilities
Engineering Cohort Steering Group (STG)	• Respond to reports of security incidents and escalate to a member of the Engineering Cohort Security Group (ECSG).<br>• Own the security incident management process.<br>• Work with the ECSG to resolve security incidents.
Engineering Cohort Security Group (ECSG)<br>AKA<br>FA-DevSecOps	• Maintain the Data Breach Response Plan.<br>• Own incident management process until a Response Team is commissioned.<br>• Maintain the Security Incident Log.<br>• Write Security Incident Reports (SIR) and maintain the Google Drive SIR folder.<br>• Conduct incident assessments.<br>• Escalate incidents within the Institute.<br>• Lead incident response until a Response Team is commissioned.<br>• Determine when a Response Team is required and work with the General Manager Software Engineering to assemble it.<br>• Work with Product Managers and development teams to implement fixes and mitigate the damage of incidents.<br>• Conduct post-incident reviews and include findings in SIR.<br>• Advise when to escalate incidents to Deakin ICT.
Response Team	• Consider what information needs to be reported to senior management and at what point.

	• For a high-profile vulnerability, identify risks that could make a breach more likely to occur.<br>• Manage the incident management lifecycle when a security incident involves a data breach or high-profile vulnerability.<br>• Coordinate the development team and provide support to its members.<br>• Assess the risks from the breach.<br>• Establish the cause and impact of a data breach that involved ICT systems.<br>• Review any security and monitoring controls related to the breach.<br>• Work with Deakin ICT to resolve the incident.
General Manager Software Engineering	• Provide leadership to the Engineering Cohort Security Group.<br>• Manage client relations with Product Managers and Co-Director.<br>• Work with the ECSG to determine when the Co-Director should be informed about an incident.<br>• Liaise with Deakin ICT during the incident lifecycle.<br>• Acts as a driving member of any assembled Response Team.
Co-Director	• Manage client relations throughout the incident management lifecycle when incidents involve a data breach or high-profile vulnerability.<br>• Keep the client informed of the findings.<br>• Escalate formally to Deakin ICT when required.
A^2I^2 Team Members	• Report security incidents to their project lead, the Engineering Cohort Steering Group, or the Engineering Cohort Security Group under the procedures outlined in the Data Breach Response Plan.<br>• Develop and test fixes for security vulnerabilities.<br>• Investigate Data Breach attack vectors.

7. Documentation

7.1. Security Incident Log

The Security Incident Log maintains an audit trail of all past security incidents. It captures meta-data about an incident and provides links to the corresponding Security Incident Report (SIR) which is a detailed journal of the steps taken to resolve the incident.

Each security incident must have an entry in the Security Incident Log.

Click here to access the Security Incident Log.

7.2. Security Incident Report

A Security Incident Report (SIR) is a historical log of the events surrounding a security incident captured in the Security Incident Log.

The SIR describes a security incident and the steps taken to investigate and resolve it. The document also includes the post-incident review findings.

A member of the Engineering Cohort Security Group will create a SIR for each security incident contained in the Security Incident Log.

Instructions for Engineering Cohort Security Group team members:

- One report per incident
    - Stored on Google Drive
    - Create a folder for each Incident (based on the ID from the Security Incident Log)
        - Create as sub-folder of Security Incident Reports
    - Create a report in the folder (create from Google Docs template: "A^2I^2 - Template - Security Incident Report")
        - The title should be "SIR-[ID]"
    - Add any investigation artefacts to the folder

8. Post-Incident Review

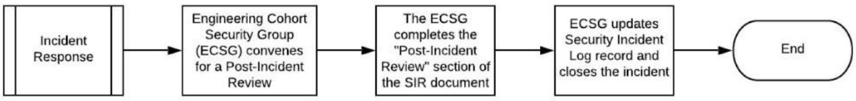

9. Appendix

9.1. Escalation Email Template

This template is merely a guide and can be modified as desired.

Title
[Provide a brief title that describes the security incident.]

Type of incident
[Is it a Data Breach or Security Vulnerability?]

Incident Identification Date
[When was the incident identified?]

What happened?
[Provide a detailed description of the security incident and any data breach that has occurred.]

Impact
[See Security Incident Log>"Data" tab>"Security Incident Impact" for options.]

Status
[What is the state of the investigation? See Security Incident Log>"Data" tab>"Status" for options.]

If the Institute is yet to resolve the incident, include the following sections.

Incident Resolution ETA
[Provide an approximate ETA (do not be too specific).]

Next steps
[What do we need to do? Explain the future actions involved in the incident investigation.]

If the Institute has a resolution for the incident, include the following sections.

Incident Resolution Date
[When was it resolved?]

Resolution Steps
[Provide a brief description of the steps taken to resolve the incident.]

2502

10. Terminology

10.1. Cyber security events

A cyber security event is an occurrence of a system, service or network state indicating a possible breach of security policy, failure of safeguards or a previously unknown situation that may be relevant to security.

10.2. Cyber security incidents

A cyber security incident is an unwanted or unexpected cyber security event, or a series of such events, that have a significant probability of compromising business operations.

11. References

1. Australian Government Information Security Manual

2503

Appendix 32 Case Report Form

CONFIDENTIAL

**Participant Id**	

Vibe Up Case Report Form

Version dated: 06 Apr 2021

Basic eligibility (ELY) - Screening

Id	Question	Response
ELY1	How old are you?	
ELY2a	Are you **currently registered** as a **student** at an Australian university, TAFE or other higher education institution?	
ELY2b	Which of the following best describes you?	
ELY2c	Which academic institution are you **currently** registered at?<br><br>If you are registered at multiple institutions, pick the one where you spend most of your time.	
ELY3a	Where do you currently live?	
ELY3b	In the **next 2 months**, are you planning to travel outside Australia?	
ELY4	How do you rate your English ability?	
ELY5a	Do you have your own smartphone with an active mobile number and internet access?	
ELY5b	Does your smartphone run **Android 5.0** (Lollipop) or higher?<br><br>If you bought your smartphone new within the past 2 years, you can assume it does.	
ELY5b	Do you have an **iPhone 6S or higher**?	
ELY6a	Have you previously registered to take part in this study?	
ELY6b	In the **next 2 months**, do you expect any major events or disruptions coming up that might make it difficult to take part (or continue taking part) in this study?	
ELY7	If you were allocated to the physical activity treatment, is there anything that would prevent you from safely taking part?<br><br>One of the brief treatments for distress in this study involves exercise. This is self-guided so you can control the intensity and type of activity involved.<br><br>If you have a heart or lung problem that interferes with exercise, you should talk to your GP or specialist before agreeing to take part in this study.	

Recruitment pathway (RTP) - Screening

Id	Question	Response
RTP1a	How did you hear about this study?	

Page 1 of 44

HC Number: HC200466 **Version dated:** 06 Apr 2021

	This information will help us get the word out to more students.	
RTP1b	Please specify:	

Contact details (CTD) - Screening

Id	Question	Response
CTD1	What is your preferred name?	
CTD2a	What is your mobile number?<br><br>Your mobile number will be your login for the study app. We'll also send instructions via SMS.	
CTD2b	Confirm your mobile number by typing it again.	
CTD3	What is your email address?	

Physical and mental health (MED) - Screening

Id	Question	Response
MED1	Have you ever been diagnosed by a professional (e.g., a doctor) with a **chronic physical health condition**?<br><br>Chronic physical health conditions are things like asthma, diabetes, cerebral palsy or sickle cell anaemia that can be managed but not cured.	
MED2	Have you ever been diagnosed by a professional (e.g., a doctor or psychologist) with a **mental health** condition?	
MED3	Which of the following condition(s) have you **ever been** diagnosed with? Pick all that apply.	
MED5	When were you **first diagnosed** with a mental health condition?	
MED4	Is your mental health condition currently **active**?<br><br>By active we mean that you are experiencing symptoms, receiving treatment and/or seeing a healthcare professional regularly for this condition.	
MED6	Are you **currently** taking any medications prescribed by a professional for your mental health?	
MED7	Are you **currently** having any therapy sessions with a professional for your mental health?	
MED8	Are you **currently** using any apps or online programmes for your mental health?	

Kessler Psychological Distress Scale, 10-item version (KTEN) - Screening

Id	Question	Response
KTEN1	About how often did you feel tired out for no good reason?	
KTEN2	About how often did you feel nervous?	

CONFIDENTIAL

Participant Id	

KTEN3	About how often did you feel so nervous that nothing could calm you down?	
KTEN4	About how often did you feel hopeless?	
KTEN5	About how often did you feel restless or fidgety?	
KTEN6	About how often did you feel so restless you could not sit still?	
KTEN7	About how often did you feel depressed?	
KTEN8	About how often did you feel that everything was an effort?	
KTEN9	About how often did you feel so sad that nothing could cheer you up?	
KTEN10	About how often did you feel worthless?	

Extended Suicidal Ideation Attributes Scale (SIDAS) - Screening

Id	Question	Response
SIDAS1	In the **past month**, how often have you had thoughts about suicide?	
SIDAS2	In the **past month**, how much control have you had over these thoughts?	
SIDAS3	In the **past month**, how close have you come to making a suicide attempt?	
SIDAS4	In the **past month**, to what extent have you felt tormented by thoughts about suicide?	
SIDAS5	In the **past month**, how much have thoughts about suicide interfered with your ability to carry out daily activities, such as school, chores or social activities?	
SAT	Have you ever made a suicide attempt?	

Demographic details (DEM) - Screening

Id	Question	Response
DEM1a	How do you describe your gender?<br><br>This information will help our research understand if different groups of people find different treatments more useful.	
DEM1b	Please specify:	
DEM2a	What was your sex recorded at birth?	
DEM2b	Please specify:	
DEM3a	How do you describe your sexual orientation?<br><br>This information will help our research understand if different groups of people find different treatments more useful.	
DEM3b	Please specify:	
DEM4	Are you of Aboriginal or Torres Strait Islander origin?<br><br>If you are of both Aboriginal and Torres Strait Islander origin, mark both 'Yes' boxes.	

CONFIDENTIAL

Participant Id	

DEM5	What is your ancestry? Pick up to two options.	
DEM6	What is the language that you mostly speak at home?	

Study and employment (WRK) - Screening

Id	Question	Response
WRK2a	What is your Weighted Average Mark (WAM) for your **current** degree or qualification?	
WRK2a	What is your Grade Point Average (GPA – 4 point scale) for your **current** degree or qualification?	
WRK2a	What is your Grade Point Average (GPA – 7 point scale) for your **current** degree or qualification?	
WRK3a	In addition to studying, do you **currently** have a paid job?	
**Id**	**Question**	**Response**
WRK1	Are you studying in Australia as an international student?	
WRK2a	What is your Weighted Average Mark (WAM) or Grade Point Average (GPA) for your **current** degree or qualification?<br><br>The WAM or GPA provides an indication of your overall academic performance. Please select the option that your institution uses.	
**Id**	**Question**	**Response**
WRK3b	**On average**, how many hours a week does your job take up?<br><br>If you have more than one job, add up all the time taken each week.	

Subjective Socioeconomic Status (SES) - Screening

Id	Question	Response
SES	Think of this ladder as representing where people stand in Australia.<br>At the top are the people who are the best off – they have the most money, the most education and the most respected jobs.	

	At the bottom are the people who are the worst off – who have the least money, least education and the least respected jobs.<br>**Where would you place yourself on this ladder?**	

Abridged EQ-5D-5L (EQV) - Screening

Id	Question	Response
EQV	We would like to know how good or bad your health is **today.**<br>100 means the **best** health you can imagine.<br>0 means the **worst** health you can imagine.<br>Mark the scale to indicate how your health is **today**.	

Abridged NIDA-Modified ASSIST Drug Screening Tool (AOD) - Screening

Id	Question	Response
AOD1	Five or more alcoholic drinks in a day.	
AOD1	Four or more alcoholic drinks in a day.	
AOD2	Tobacco products.	
AOD3	Prescription drugs for non-medical reasons.	
AOD4	Illegal drugs.	

Short Warwick Edinburgh Mental Wellbeing Scale (WBS) - Screening

Id	Question	Response
WBS1	I've been feeling optimistic about the future.	
WBS2	I've been feeling useful.	
WBS3	I've been feeling relaxed.	
WBS4	I've been dealing with problems well.	
WBS5	I've been thinking clearly.	
WBS6	I've been feeling close to other people.	
WBS7	I've been able to make up my own mind about things.	

Multidimensional Scale of Perceived Social Support (PSS) - Screening

Id	Question	Response
PSS1	There is a special person who is around when I am in need.	
PSS2	There is a special person with whom I can share joys and sorrows.	
PSS3	My family really tries to help me.	
PSS4	I get the emotional help and support I need from my family.	
PSS5	I have a special person who is a real source of comfort to me.	

CONFIDENTIAL

Participant Id	

PSS6	My friends really try to help me.	
PSS7	I can count on my friends when things go wrong.	
PSS8	I can talk about my problems with my family.	
PSS9	I have friends with whom I can share my joys and sorrows.	
PSS10	There is a special person in my life who cares about my feelings.	
PSS11	My family is willing to help me make decisions.	
PSS12	I can talk about my problems with my friends.	

Depression, Anxiety and Stress Scale, 21-item version (DASS) - Baseline

Id	Question	Response
DASS1	I found it hard to wind down.	
DASS2	I was aware of dryness of my mouth.	
DASS3	I couldn't seem to experience any positive feeling at all.	
DASS4	I experienced breathing difficulty (e.g., excessively rapid breathing, breathlessness in the absence of physical exertion.)	
DASS5	I found it difficult to work up the initiative to do things.	
DASS6	I tended to over-react to situations.	
DASS7	I experienced trembling (e.g., in the hands.)	
DASS8	I felt that I was using a lot of nervous energy.	
DASS9	I was worried about situations in which I might panic and make a fool of myself.	
DASS10	I felt that I had nothing to look forward to.	
DASS11	I found myself getting agitated.	
DASS12	I found it difficult to relax.	
DASS13	I felt downhearted and blue.	
DASS14	I was intolerant of anything that kept me from getting on with what I was doing.	
DASS15	I felt I was close to panic.	
DASS16	I was unable to become enthusiastic about anything.	
DASS17	I felt I wasn't worth much as a person.	
DASS18	I felt that I was rather touchy.	
DASS19	I was aware of the action of my heart in the absence of physical exertion (e.g., sense of heart rate increase, heart missing a beat.)	
DASS20	I felt scared without any good reason.	
DASS21	I felt that life was meaningless.	

Depression, Anxiety and Stress Scale, 21-item version (DASS) - Mid

Id	Question	Response
DASS1	I found it hard to wind down.	
DASS2	I was aware of dryness of my mouth.	
DASS3	I couldn't seem to experience any positive feeling at all.	

2516

CONFIDENTIAL

Participant Id	

DASS4	I experienced breathing difficulty (e.g., excessively rapid breathing, breathlessness in the absence of physical exertion.)	
DASS5	I found it difficult to work up the initiative to do things.	
DASS6	I tended to over-react to situations.	
DASS7	I experienced trembling (e.g., in the hands.)	
DASS8	I felt that I was using a lot of nervous energy.	
DASS9	I was worried about situations in which I might panic and make a fool of myself.	
DASS10	I felt that I had nothing to look forward to.	
DASS11	I found myself getting agitated.	
DASS12	I found it difficult to relax.	
DASS13	I felt downhearted and blue.	
DASS14	I was intolerant of anything that kept me from getting on with what I was doing.	
DASS15	I felt I was close to panic.	
DASS16	I was unable to become enthusiastic about anything.	
DASS17	I felt I wasn't worth much as a person.	
DASS18	I felt that I was rather touchy.	
DASS19	I was aware of the action of my heart in the absence of physical exertion (e.g., sense of heart rate increase, heart missing a beat.)	
DASS20	I felt scared without any good reason.	
DASS21	I felt that life was meaningless.	

Depression, Anxiety and Stress Scale, 21-item version (DASS) - Post

Id	Question	Response
DASS1	I found it hard to wind down.	
DASS2	I was aware of dryness of my mouth.	
DASS3	I couldn't seem to experience any positive feeling at all.	
DASS4	I experienced breathing difficulty (e.g., excessively rapid breathing, breathlessness in the absence of physical exertion.)	
DASS5	I found it difficult to work up the initiative to do things.	
DASS6	I tended to over-react to situations.	
DASS7	I experienced trembling (e.g., in the hands.)	
DASS8	I felt that I was using a lot of nervous energy.	
DASS9	I was worried about situations in which I might panic and make a fool of myself.	
DASS10	I felt that I had nothing to look forward to.	
DASS11	I found myself getting agitated.	
DASS12	I found it difficult to relax.	
DASS13	I felt downhearted and blue.	
DASS14	I was intolerant of anything that kept me from getting on with what I was doing.	
DASS15	I felt I was close to panic.	

2517

CONFIDENTIAL

Participant Id	

DASS16	I was unable to become enthusiastic about anything.	
DASS17	I felt I wasn't worth much as a person.	
DASS18	I felt that I was rather touchy.	
DASS19	I was aware of the action of my heart in the absence of physical exertion (e.g., sense of heart rate increase, heart missing a beat.)	
DASS20	I felt scared without any good reason.	
DASS21	I felt that life was meaningless.	

Depression, Anxiety and Stress Scale, 21-item version (DASS) - Follow-up

Id	Question	Response
DASS1	I found it hard to wind down.	
DASS2	I was aware of dryness of my mouth.	
DASS3	I couldn't seem to experience any positive feeling at all.	
DASS4	I experienced breathing difficulty (e.g., excessively rapid breathing, breathlessness in the absence of physical exertion.)	
DASS5	I found it difficult to work up the initiative to do things.	
DASS6	I tended to over-react to situations.	
DASS7	I experienced trembling (e.g., in the hands.)	
DASS8	I felt that I was using a lot of nervous energy.	
DASS9	I was worried about situations in which I might panic and make a fool of myself.	
DASS10	I felt that I had nothing to look forward to.	
DASS11	I found myself getting agitated.	
DASS12	I found it difficult to relax.	
DASS13	I felt downhearted and blue.	
DASS14	I was intolerant of anything that kept me from getting on with what I was doing.	
DASS15	I felt I was close to panic.	
DASS16	I was unable to become enthusiastic about anything.	
DASS17	I felt I wasn't worth much as a person.	
DASS18	I felt that I was rather touchy.	
DASS19	I was aware of the action of my heart in the absence of physical exertion (e.g., sense of heart rate increase, heart missing a beat.)	
DASS20	I felt scared without any good reason.	
DASS21	I felt that life was meaningless.	

Modified Physical Activity Vital Sign (PAVS) - Baseline

Id	Question	Response
PAVS1	In the past **week**, on how many **days** did you engage in moderate to strenuous exercise?	

	Moderate exercise means that you are breathing hard enough that, while you might be able to talk, singing would not be possible.<br><br>Strenuous exercise means being out of breath enough that you can't talk.	
PAVS2	On average, how many **minutes** did each session of moderate to strenuous exercise last for?	

Modified Physical Activity Vital Sign (PAVS) - Mid

Id	Question	Response
PAVS1	In the past **week**, on how many **days** did you engage in moderate to strenuous exercise?<br><br>Moderate exercise means that you are breathing hard enough that, while you might be able to talk, singing would not be possible.<br><br>Strenuous exercise means being out of breath enough that you can't talk.	
PAVS2	On average, how many **minutes** did each session of moderate to strenuous exercise last for?	

Modified Physical Activity Vital Sign (PAVS) - Post

Id	Question	Response
PAVS1	In the past **week**, on how many **days** did you engage in moderate to strenuous exercise?<br><br>Moderate exercise means that you are breathing hard enough that, while you might be able to talk, singing would not be possible.<br><br>Strenuous exercise means being out of breath enough that you can't talk.	
PAVS2	On average, how many **minutes** did each session of moderate to strenuous exercise last for?	

Modified Physical Activity Vital Sign (PAVS) - Follow-up

Id	Question	Response
PAVS1	In the past **week**, on how many **days** did you engage in moderate to strenuous exercise?<br><br>Moderate exercise means that you are breathing hard enough that, while you might be able to talk, singing would not be possible.<br><br>Strenuous exercise means being out of breath enough that you can't talk.	

2522

CONFIDENTIAL

Participant Id	

PAVS2	On average, how many **minutes** did each session of moderate to strenuous exercise last for?	

Abridged Pittsburgh Sleep Quality Index (PSQI) - Baseline

Id	Question	Response
PSQI6	During the past **week**, how would you rate your sleep quality overall?	

Abridged Pittsburgh Sleep Quality Index (PSQI) - Mid

Id	Question	Response
PSQI6	During the past **week**, how would you rate your sleep quality overall?	

Abridged Pittsburgh Sleep Quality Index (PSQI) - Post

Id	Question	Response
PSQI6	During the past **week**, how would you rate your sleep quality overall?	

Abridged Pittsburgh Sleep Quality Index (PSQI) - Follow-up

Id	Question	Response
PSQI6	During the past **week**, how would you rate your sleep quality overall?	

Mindfulness single item questionnaire (MIND) - Baseline

Id	Question	Response
MIND1	Mindfulness is a practice where you intentionally focus your attention on what you're experiencing in the present moment, with an attitude of openness and non-judgment.<br>During the past **week**, how mindful have you been?	

Mindfulness single item questionnaire (MIND) - Mid

Id	Question	Response
MIND1	Mindfulness is a practice where you intentionally focus your attention on what you're experiencing in the present moment, with an attitude of openness and non-judgment.<br>During the past **week**, how mindful have you been?	

2523

2524

CONFIDENTIAL

Participant Id	

Mindfulness single item questionnaire (MIND) - Post

Id	Question	Response
MIND1	Mindfulness is a practice where you intentionally focus your attention on what you're experiencing in the present moment, with an attitude of openness and non-judgment.<br>During the past **week**, how mindful have you been?	

Mindfulness single item questionnaire (MIND) - Follow-up

Id	Question	Response
MIND1	Mindfulness is a practice where you intentionally focus your attention on what you're experiencing in the present moment, with an attitude of openness and non-judgment.<br>During the past **week**, how mindful have you been?	

Modified Positive and Negative Affect Schedule, Short Form (PANAS) - EMA - Day 1, Signal 1

Id	Question	Response
PANAS0	How do you feel **right now**? (Please select all that apply)	
PANAS1	To what extent do you feel **upset** right now?	
PANAS2	To what extent do you feel **hostile** right now?	
PANAS3	To what extent do you feel **alert** right now?	
PANAS4	To what extent do you feel **ashamed** right now?	
PANAS5	To what extent do you feel **inspired** right now?	
PANAS6	To what extent do you feel **nervous** right now?	
PANAS7	To what extent do you feel **determined** right now?	
PANAS8	To what extent do you feel **attentive** right now?	
PANAS9	To what extent do you feel **afraid** right now?	
PANAS10	To what extent do you feel **active** right now?	
PANAS11	To what extent do you feel **hopeless** right now?	
PANAS12	To what extent do you feel **calm** right now?	

Modified Positive and Negative Affect Schedule, Short Form (PANAS) - EMA - Day 1, Signal 2

Id	Question	Response
PANAS0	How do you feel **right now**? (Please select all that apply)	
PANAS1	To what extent do you feel **upset** right now?	
PANAS2	To what extent do you feel **hostile** right now?	
PANAS3	To what extent do you feel **alert** right now?	
PANAS4	To what extent do you feel **ashamed** right now?	

2525

CONFIDENTIAL

Participant Id	

PANAS5	To what extent do you feel **inspired** right now?	
PANAS6	To what extent do you feel **nervous** right now?	
PANAS7	To what extent do you feel **determined** right now?	
PANAS8	To what extent do you feel **attentive** right now?	
PANAS9	To what extent do you feel **afraid** right now?	
PANAS10	To what extent do you feel **active** right now?	
PANAS11	To what extent do you feel **hopeless** right now?	
PANAS12	To what extent do you feel **calm** right now?	

Modified Positive and Negative Affect Schedule, Short Form (PANAS) - EMA - Day 2, Signal 1

Id	Question	Response
PANAS0	How do you feel **right now**? (Please select all that apply)	
PANAS1	To what extent do you feel **upset** right now?	
PANAS2	To what extent do you feel **hostile** right now?	
PANAS3	To what extent do you feel **alert** right now?	
PANAS4	To what extent do you feel **ashamed** right now?	
PANAS5	To what extent do you feel **inspired** right now?	
PANAS6	To what extent do you feel **nervous** right now?	
PANAS7	To what extent do you feel **determined** right now?	
PANAS8	To what extent do you feel **attentive** right now?	
PANAS9	To what extent do you feel **afraid** right now?	
PANAS10	To what extent do you feel **active** right now?	
PANAS11	To what extent do you feel **hopeless** right now?	
PANAS12	To what extent do you feel **calm** right now?	

Modified Positive and Negative Affect Schedule, Short Form (PANAS) - EMA - Day 2, Signal 2

Id	Question	Response
PANAS0	How do you feel **right now**? (Please select all that apply)	
PANAS1	To what extent do you feel **upset** right now?	
PANAS2	To what extent do you feel **hostile** right now?	
PANAS3	To what extent do you feel **alert** right now?	
PANAS4	To what extent do you feel **ashamed** right now?	
PANAS5	To what extent do you feel **inspired** right now?	
PANAS6	To what extent do you feel **nervous** right now?	
PANAS7	To what extent do you feel **determined** right now?	
PANAS8	To what extent do you feel **attentive** right now?	
PANAS9	To what extent do you feel **afraid** right now?	
PANAS10	To what extent do you feel **active** right now?	
PANAS11	To what extent do you feel **hopeless** right now?	
PANAS12	To what extent do you feel **calm** right now?	

2528

CONFIDENTIAL

Participant Id	

Modified Positive and Negative Affect Schedule, Short Form (PANAS) - EMA - Day 3, Signal 1

Id	Question	Response
PANAS0	How do you feel **right now**? (Please select all that apply)	
PANAS1	To what extent do you feel **upset** right now?	
PANAS2	To what extent do you feel **hostile** right now?	
PANAS3	To what extent do you feel **alert** right now?	
PANAS4	To what extent do you feel **ashamed** right now?	
PANAS5	To what extent do you feel **inspired** right now?	
PANAS6	To what extent do you feel **nervous** right now?	
PANAS7	To what extent do you feel **determined** right now?	
PANAS8	To what extent do you feel **attentive** right now?	
PANAS9	To what extent do you feel **afraid** right now?	
PANAS10	To what extent do you feel **active** right now?	
PANAS11	To what extent do you feel **hopeless** right now?	
PANAS12	To what extent do you feel **calm** right now?	

Modified Positive and Negative Affect Schedule, Short Form (PANAS) - EMA - Day 3, Signal 2

Id	Question	Response
PANAS0	How do you feel **right now**? (Please select all that apply)	
PANAS1	To what extent do you feel **upset** right now?	
PANAS2	To what extent do you feel **hostile** right now?	
PANAS3	To what extent do you feel **alert** right now?	
PANAS4	To what extent do you feel **ashamed** right now?	
PANAS5	To what extent do you feel **inspired** right now?	
PANAS6	To what extent do you feel **nervous** right now?	
PANAS7	To what extent do you feel **determined** right now?	
PANAS8	To what extent do you feel **attentive** right now?	
PANAS9	To what extent do you feel **afraid** right now?	
PANAS10	To what extent do you feel **active** right now?	
PANAS11	To what extent do you feel **hopeless** right now?	
PANAS12	To what extent do you feel **calm** right now?	

Modified Positive and Negative Affect Schedule, Short Form (PANAS) - EMA - Day 4, Signal 1

Id	Question	Response
PANAS0	How do you feel **right now**? (Please select all that apply)	
PANAS1	To what extent do you feel **upset** right now?	
PANAS2	To what extent do you feel **hostile** right now?	
PANAS3	To what extent do you feel **alert** right now?	
PANAS4	To what extent do you feel **ashamed** right now?	
PANAS5	To what extent do you feel **inspired** right now?	

2529

2530

CONFIDENTIAL

Participant Id	

PANAS6	To what extent do you feel **nervous** right now?	
PANAS7	To what extent do you feel **determined** right now?	
PANAS8	To what extent do you feel **attentive** right now?	
PANAS9	To what extent do you feel **afraid** right now?	
PANAS10	To what extent do you feel **active** right now?	
PANAS11	To what extent do you feel **hopeless** right now?	
PANAS12	To what extent do you feel **calm** right now?	

Modified Positive and Negative Affect Schedule, Short Form (PANAS) - EMA - Day 4, Signal 2

Id	Question	Response
PANAS0	How do you feel **right now**? (Please select all that apply)	
PANAS1	To what extent do you feel **upset** right now?	
PANAS2	To what extent do you feel **hostile** right now?	
PANAS3	To what extent do you feel **alert** right now?	
PANAS4	To what extent do you feel **ashamed** right now?	
PANAS5	To what extent do you feel **inspired** right now?	
PANAS6	To what extent do you feel **nervous** right now?	
PANAS7	To what extent do you feel **determined** right now?	
PANAS8	To what extent do you feel **attentive** right now?	
PANAS9	To what extent do you feel **afraid** right now?	
PANAS10	To what extent do you feel **active** right now?	
PANAS11	To what extent do you feel **hopeless** right now?	
PANAS12	To what extent do you feel **calm** right now?	

Modified Positive and Negative Affect Schedule, Short Form (PANAS) - EMA - Day 5, Signal 1

Id	Question	Response
PANAS0	How do you feel **right now**? (Please select all that apply)	
PANAS1	To what extent do you feel **upset** right now?	
PANAS2	To what extent do you feel **hostile** right now?	
PANAS3	To what extent do you feel **alert** right now?	
PANAS4	To what extent do you feel **ashamed** right now?	
PANAS5	To what extent do you feel **inspired** right now?	
PANAS6	To what extent do you feel **nervous** right now?	
PANAS7	To what extent do you feel **determined** right now?	
PANAS8	To what extent do you feel **attentive** right now?	
PANAS9	To what extent do you feel **afraid** right now?	
PANAS10	To what extent do you feel **active** right now?	
PANAS11	To what extent do you feel **hopeless** right now?	
PANAS12	To what extent do you feel **calm** right now?	

Page 14 of 44

HC Number: HC200466 **Version dated:** 06 Apr 2021

2531

CONFIDENTIAL

Participant Id	

Modified Positive and Negative Affect Schedule, Short Form (PANAS) - EMA - Day 5, Signal 2

Id	Question	Response
PANAS0	How do you feel **right now**? (Please select all that apply)	
PANAS1	To what extent do you feel **upset** right now?	
PANAS2	To what extent do you feel **hostile** right now?	
PANAS3	To what extent do you feel **alert** right now?	
PANAS4	To what extent do you feel **ashamed** right now?	
PANAS5	To what extent do you feel **inspired** right now?	
PANAS6	To what extent do you feel **nervous** right now?	
PANAS7	To what extent do you feel **determined** right now?	
PANAS8	To what extent do you feel **attentive** right now?	
PANAS9	To what extent do you feel **afraid** right now?	
PANAS10	To what extent do you feel **active** right now?	
PANAS11	To what extent do you feel **hopeless** right now?	
PANAS12	To what extent do you feel **calm** right now?	

Modified Positive and Negative Affect Schedule, Short Form (PANAS) - EMA - Day 6, Signal 1

Id	Question	Response
PANAS0	How do you feel **right now**? (Please select all that apply)	
PANAS1	To what extent do you feel **upset** right now?	
PANAS2	To what extent do you feel **hostile** right now?	
PANAS3	To what extent do you feel **alert** right now?	
PANAS4	To what extent do you feel **ashamed** right now?	
PANAS5	To what extent do you feel **inspired** right now?	
PANAS6	To what extent do you feel **nervous** right now?	
PANAS7	To what extent do you feel **determined** right now?	
PANAS8	To what extent do you feel **attentive** right now?	
PANAS9	To what extent do you feel **afraid** right now?	
PANAS10	To what extent do you feel **active** right now?	
PANAS11	To what extent do you feel **hopeless** right now?	
PANAS12	To what extent do you feel **calm** right now?	

Modified Positive and Negative Affect Schedule, Short Form (PANAS) - EMA - Day 6, Signal 2

Id	Question	Response
PANAS0	How do you feel **right now**? (Please select all that apply)	
PANAS1	To what extent do you feel **upset** right now?	
PANAS2	To what extent do you feel **hostile** right now?	
PANAS3	To what extent do you feel **alert** right now?	
PANAS4	To what extent do you feel **ashamed** right now?	
PANAS5	To what extent do you feel **inspired** right now?	

CONFIDENTIAL

Participant Id	

PANAS6	To what extent do you feel **nervous** right now?	
PANAS7	To what extent do you feel **determined** right now?	
PANAS8	To what extent do you feel **attentive** right now?	
PANAS9	To what extent do you feel **afraid** right now?	
PANAS10	To what extent do you feel **active** right now?	
PANAS11	To what extent do you feel **hopeless** right now?	
PANAS12	To what extent do you feel **calm** right now?	

Modified Positive and Negative Affect Schedule, Short Form (PANAS) - EMA - Day 7, Signal 1

Id	Question	Response
PANAS0	How do you feel **right now**? (Please select all that apply)	
PANAS1	To what extent do you feel **upset** right now?	
PANAS2	To what extent do you feel **hostile** right now?	
PANAS3	To what extent do you feel **alert** right now?	
PANAS4	To what extent do you feel **ashamed** right now?	
PANAS5	To what extent do you feel **inspired** right now?	
PANAS6	To what extent do you feel **nervous** right now?	
PANAS7	To what extent do you feel **determined** right now?	
PANAS8	To what extent do you feel **attentive** right now?	
PANAS9	To what extent do you feel **afraid** right now?	
PANAS10	To what extent do you feel **active** right now?	
PANAS11	To what extent do you feel **hopeless** right now?	
PANAS12	To what extent do you feel **calm** right now?	

Modified Positive and Negative Affect Schedule, Short Form (PANAS) - EMA - Day 7, Signal 2

Id	Question	Response
PANAS0	How do you feel **right now**? (Please select all that apply)	
PANAS1	To what extent do you feel **upset** right now?	
PANAS2	To what extent do you feel **hostile** right now?	
PANAS3	To what extent do you feel **alert** right now?	
PANAS4	To what extent do you feel **ashamed** right now?	
PANAS5	To what extent do you feel **inspired** right now?	
PANAS6	To what extent do you feel **nervous** right now?	
PANAS7	To what extent do you feel **determined** right now?	
PANAS8	To what extent do you feel **attentive** right now?	
PANAS9	To what extent do you feel **afraid** right now?	
PANAS10	To what extent do you feel **active** right now?	
PANAS11	To what extent do you feel **hopeless** right now?	
PANAS12	To what extent do you feel **calm** right now?	

2536

CONFIDENTIAL

Participant Id	

Modified Positive and Negative Affect Schedule, Short Form (PANAS) - EMA - Day 8, Signal 1

Id	Question	Response
PANAS0	How do you feel **right now**? (Please select all that apply)	
PANAS1	To what extent do you feel **upset** right now?	
PANAS2	To what extent do you feel **hostile** right now?	
PANAS3	To what extent do you feel **alert** right now?	
PANAS4	To what extent do you feel **ashamed** right now?	
PANAS5	To what extent do you feel **inspired** right now?	
PANAS6	To what extent do you feel **nervous** right now?	
PANAS7	To what extent do you feel **determined** right now?	
PANAS8	To what extent do you feel **attentive** right now?	
PANAS9	To what extent do you feel **afraid** right now?	
PANAS10	To what extent do you feel **active** right now?	
PANAS11	To what extent do you feel **hopeless** right now?	
PANAS12	To what extent do you feel **calm** right now?	

Modified Positive and Negative Affect Schedule, Short Form (PANAS) - EMA - Day 8, Signal 2

Id	Question	Response
PANAS0	How do you feel **right now**? (Please select all that apply)	
PANAS1	To what extent do you feel **upset** right now?	
PANAS2	To what extent do you feel **hostile** right now?	
PANAS3	To what extent do you feel **alert** right now?	
PANAS4	To what extent do you feel **ashamed** right now?	
PANAS5	To what extent do you feel **inspired** right now?	
PANAS6	To what extent do you feel **nervous** right now?	
PANAS7	To what extent do you feel **determined** right now?	
PANAS8	To what extent do you feel **attentive** right now?	
PANAS9	To what extent do you feel **afraid** right now?	
PANAS10	To what extent do you feel **active** right now?	
PANAS11	To what extent do you feel **hopeless** right now?	
PANAS12	To what extent do you feel **calm** right now?	

Modified Positive and Negative Affect Schedule, Short Form (PANAS) - EMA - Day 9, Signal 1

Id	Question	Response
PANAS0	How do you feel **right now**? (Please select all that apply)	
PANAS1	To what extent do you feel **upset** right now?	
PANAS2	To what extent do you feel **hostile** right now?	
PANAS3	To what extent do you feel **alert** right now?	
PANAS4	To what extent do you feel **ashamed** right now?	
PANAS5	To what extent do you feel **inspired** right now?	

2537

PANAS6	To what extent do you feel **nervous** right now?	
PANAS7	To what extent do you feel **determined** right now?	
PANAS8	To what extent do you feel **attentive** right now?	
PANAS9	To what extent do you feel **afraid** right now?	
PANAS10	To what extent do you feel **active** right now?	
PANAS11	To what extent do you feel **hopeless** right now?	
PANAS12	To what extent do you feel **calm** right now?	

Modified Positive and Negative Affect Schedule, Short Form (PANAS) - EMA - Day 9, Signal 2

Id	Question	Response
PANAS0	How do you feel **right now**? (Please select all that apply)	
PANAS1	To what extent do you feel **upset** right now?	
PANAS2	To what extent do you feel **hostile** right now?	
PANAS3	To what extent do you feel **alert** right now?	
PANAS4	To what extent do you feel **ashamed** right now?	
PANAS5	To what extent do you feel **inspired** right now?	
PANAS6	To what extent do you feel **nervous** right now?	
PANAS7	To what extent do you feel **determined** right now?	
PANAS8	To what extent do you feel **attentive** right now?	
PANAS9	To what extent do you feel **afraid** right now?	
PANAS10	To what extent do you feel **active** right now?	
PANAS11	To what extent do you feel **hopeless** right now?	
PANAS12	To what extent do you feel **calm** right now?	

Modified Positive and Negative Affect Schedule, Short Form (PANAS) - EMA - Day 10, Signal 1 (Control arm)

Id	Question	Response
PANAS0	How do you feel **right now**? (Please select all that apply)	
PANAS1	To what extent do you feel **upset** right now?	
PANAS2	To what extent do you feel **hostile** right now?	
PANAS3	To what extent do you feel **alert** right now?	
PANAS4	To what extent do you feel **ashamed** right now?	
PANAS5	To what extent do you feel **inspired** right now?	
PANAS6	To what extent do you feel **nervous** right now?	
PANAS7	To what extent do you feel **determined** right now?	
PANAS8	To what extent do you feel **attentive** right now?	
PANAS9	To what extent do you feel **afraid** right now?	
PANAS10	To what extent do you feel **active** right now?	
PANAS11	To what extent do you feel **hopeless** right now?	
PANAS12	To what extent do you feel **calm** right now?	

2540

CONFIDENTIAL

Participant Id	

Modified Positive and Negative Affect Schedule, Short Form (PANAS) - EMA - Day 10, Signal 2 (Control arm)

Id	Question	Response
PANAS0	How do you feel **right now**? (Please select all that apply)	
PANAS1	To what extent do you feel **upset** right now?	
PANAS2	To what extent do you feel **hostile** right now?	
PANAS3	To what extent do you feel **alert** right now?	
PANAS4	To what extent do you feel **ashamed** right now?	
PANAS5	To what extent do you feel **inspired** right now?	
PANAS6	To what extent do you feel **nervous** right now?	
PANAS7	To what extent do you feel **determined** right now?	
PANAS8	To what extent do you feel **attentive** right now?	
PANAS9	To what extent do you feel **afraid** right now?	
PANAS10	To what extent do you feel **active** right now?	
PANAS11	To what extent do you feel **hopeless** right now?	
PANAS12	To what extent do you feel **calm** right now?	

Modified Positive and Negative Affect Schedule, Short Form (PANAS) - EMA - Day 11, Signal 1 (Control arm)

Id	Question	Response
PANAS0	How do you feel **right now**? (Please select all that apply)	
PANAS1	To what extent do you feel **upset** right now?	
PANAS2	To what extent do you feel **hostile** right now?	
PANAS3	To what extent do you feel **alert** right now?	
PANAS4	To what extent do you feel **ashamed** right now?	
PANAS5	To what extent do you feel **inspired** right now?	
PANAS6	To what extent do you feel **nervous** right now?	
PANAS7	To what extent do you feel **determined** right now?	
PANAS8	To what extent do you feel **attentive** right now?	
PANAS9	To what extent do you feel **afraid** right now?	
PANAS10	To what extent do you feel **active** right now?	
PANAS11	To what extent do you feel **hopeless** right now?	
PANAS12	To what extent do you feel **calm** right now?	

Modified Positive and Negative Affect Schedule, Short Form (PANAS) - EMA - Day 11, Signal 2 (Control arm)

Id	Question	Response
PANAS0	How do you feel **right now**? (Please select all that apply)	
PANAS1	To what extent do you feel **upset** right now?	
PANAS2	To what extent do you feel **hostile** right now?	
PANAS3	To what extent do you feel **alert** right now?	
PANAS4	To what extent do you feel **ashamed** right now?	
PANAS5	To what extent do you feel **inspired** right now?	

2541

2542

CONFIDENTIAL

Participant Id	

PANAS6	To what extent do you feel **nervous** right now?	
PANAS7	To what extent do you feel **determined** right now?	
PANAS8	To what extent do you feel **attentive** right now?	
PANAS9	To what extent do you feel **afraid** right now?	
PANAS10	To what extent do you feel **active** right now?	
PANAS11	To what extent do you feel **hopeless** right now?	
PANAS12	To what extent do you feel **calm** right now?	

Modified Positive and Negative Affect Schedule, Short Form (PANAS) - EMA - Day 12, Signal 1 (Control arm)

Id	Question	Response
PANAS0	How do you feel **right now**? (Please select all that apply)	
PANAS1	To what extent do you feel **upset** right now?	
PANAS2	To what extent do you feel **hostile** right now?	
PANAS3	To what extent do you feel **alert** right now?	
PANAS4	To what extent do you feel **ashamed** right now?	
PANAS5	To what extent do you feel **inspired** right now?	
PANAS6	To what extent do you feel **nervous** right now?	
PANAS7	To what extent do you feel **determined** right now?	
PANAS8	To what extent do you feel **attentive** right now?	
PANAS9	To what extent do you feel **afraid** right now?	
PANAS10	To what extent do you feel **active** right now?	
PANAS11	To what extent do you feel **hopeless** right now?	
PANAS12	To what extent do you feel **calm** right now?	

Modified Positive and Negative Affect Schedule, Short Form (PANAS) - EMA - Day 12, Signal 2 (Control arm)

Id	Question	Response
PANAS0	How do you feel **right now**? (Please select all that apply)	
PANAS1	To what extent do you feel **upset** right now?	
PANAS2	To what extent do you feel **hostile** right now?	
PANAS3	To what extent do you feel **alert** right now?	
PANAS4	To what extent do you feel **ashamed** right now?	
PANAS5	To what extent do you feel **inspired** right now?	
PANAS6	To what extent do you feel **nervous** right now?	
PANAS7	To what extent do you feel **determined** right now?	
PANAS8	To what extent do you feel **attentive** right now?	
PANAS9	To what extent do you feel **afraid** right now?	
PANAS10	To what extent do you feel **active** right now?	
PANAS11	To what extent do you feel **hopeless** right now?	
PANAS12	To what extent do you feel **calm** right now?	

2543

2544

CONFIDENTIAL

Participant Id	

Modified Positive and Negative Affect Schedule, Short Form (PANAS) - EMA - Day 13, Signal 1 (Control arm)

Id	Question	Response
PANAS0	How do you feel **right now**? (Please select all that apply)	
PANAS1	To what extent do you feel **upset** right now?	
PANAS2	To what extent do you feel **hostile** right now?	
PANAS3	To what extent do you feel **alert** right now?	
PANAS4	To what extent do you feel **ashamed** right now?	
PANAS5	To what extent do you feel **inspired** right now?	
PANAS6	To what extent do you feel **nervous** right now?	
PANAS7	To what extent do you feel **determined** right now?	
PANAS8	To what extent do you feel **attentive** right now?	
PANAS9	To what extent do you feel **afraid** right now?	
PANAS10	To what extent do you feel **active** right now?	
PANAS11	To what extent do you feel **hopeless** right now?	
PANAS12	To what extent do you feel **calm** right now?	

Modified Positive and Negative Affect Schedule, Short Form (PANAS) - EMA - Day 13, Signal 2 (Control arm)

Id	Question	Response
PANAS0	How do you feel **right now**? (Please select all that apply)	
PANAS1	To what extent do you feel **upset** right now?	
PANAS2	To what extent do you feel **hostile** right now?	
PANAS3	To what extent do you feel **alert** right now?	
PANAS4	To what extent do you feel **ashamed** right now?	
PANAS5	To what extent do you feel **inspired** right now?	
PANAS6	To what extent do you feel **nervous** right now?	
PANAS7	To what extent do you feel **determined** right now?	
PANAS8	To what extent do you feel **attentive** right now?	
PANAS9	To what extent do you feel **afraid** right now?	
PANAS10	To what extent do you feel **active** right now?	
PANAS11	To what extent do you feel **hopeless** right now?	
PANAS12	To what extent do you feel **calm** right now?	

Modified Positive and Negative Affect Schedule, Short Form (PANAS) - EMA - Day 14, Signal 1 (Control arm)

Id	Question	Response
PANAS0	How do you feel **right now**? (Please select all that apply)	
PANAS1	To what extent do you feel **upset** right now?	
PANAS2	To what extent do you feel **hostile** right now?	
PANAS3	To what extent do you feel **alert** right now?	
PANAS4	To what extent do you feel **ashamed** right now?	
PANAS5	To what extent do you feel **inspired** right now?	

2545

2546

CONFIDENTIAL

Participant Id	

PANAS6	To what extent do you feel **nervous** right now?	
PANAS7	To what extent do you feel **determined** right now?	
PANAS8	To what extent do you feel **attentive** right now?	
PANAS9	To what extent do you feel **afraid** right now?	
PANAS10	To what extent do you feel **active** right now?	
PANAS11	To what extent do you feel **hopeless** right now?	
PANAS12	To what extent do you feel **calm** right now?	

Modified Positive and Negative Affect Schedule, Short Form (PANAS) - EMA - Day 14, Signal 2 (Control arm)

Id	Question	Response
PANAS0	How do you feel **right now**? (Please select all that apply)	
PANAS1	To what extent do you feel **upset** right now?	
PANAS2	To what extent do you feel **hostile** right now?	
PANAS3	To what extent do you feel **alert** right now?	
PANAS4	To what extent do you feel **ashamed** right now?	
PANAS5	To what extent do you feel **inspired** right now?	
PANAS6	To what extent do you feel **nervous** right now?	
PANAS7	To what extent do you feel **determined** right now?	
PANAS8	To what extent do you feel **attentive** right now?	
PANAS9	To what extent do you feel **afraid** right now?	
PANAS10	To what extent do you feel **active** right now?	
PANAS11	To what extent do you feel **hopeless** right now?	
PANAS12	To what extent do you feel **calm** right now?	

Modified Positive and Negative Affect Schedule, Short Form (PANAS) - EMA - Day 15, Signal 1 (Control arm)

Id	Question	Response
PANAS0	How do you feel **right now**? (Please select all that apply)	
PANAS1	To what extent do you feel **upset** right now?	
PANAS2	To what extent do you feel **hostile** right now?	
PANAS3	To what extent do you feel **alert** right now?	
PANAS4	To what extent do you feel **ashamed** right now?	
PANAS5	To what extent do you feel **inspired** right now?	
PANAS6	To what extent do you feel **nervous** right now?	
PANAS7	To what extent do you feel **determined** right now?	
PANAS8	To what extent do you feel **attentive** right now?	
PANAS9	To what extent do you feel **afraid** right now?	
PANAS10	To what extent do you feel **active** right now?	
PANAS11	To what extent do you feel **hopeless** right now?	
PANAS12	To what extent do you feel **calm** right now?	

2547

CONFIDENTIAL

Participant Id	

Modified Positive and Negative Affect Schedule, Short Form (PANAS) - EMA - Day 15, Signal 2 (Control arm)

Id	Question	Response
PANAS0	How do you feel **right now**? (Please select all that apply)	
PANAS1	To what extent do you feel **upset** right now?	
PANAS2	To what extent do you feel **hostile** right now?	
PANAS3	To what extent do you feel **alert** right now?	
PANAS4	To what extent do you feel **ashamed** right now?	
PANAS5	To what extent do you feel **inspired** right now?	
PANAS6	To what extent do you feel **nervous** right now?	
PANAS7	To what extent do you feel **determined** right now?	
PANAS8	To what extent do you feel **attentive** right now?	
PANAS9	To what extent do you feel **afraid** right now?	
PANAS10	To what extent do you feel **active** right now?	
PANAS11	To what extent do you feel **hopeless** right now?	
PANAS12	To what extent do you feel **calm** right now?	

Modified Positive and Negative Affect Schedule, Short Form (PANAS) - EMA - Day 16, Signal 1 (Control arm)

Id	Question	Response
PANAS0	How do you feel **right now**? (Please select all that apply)	
PANAS1	To what extent do you feel **upset** right now?	
PANAS2	To what extent do you feel **hostile** right now?	
PANAS3	To what extent do you feel **alert** right now?	
PANAS4	To what extent do you feel **ashamed** right now?	
PANAS5	To what extent do you feel **inspired** right now?	
PANAS6	To what extent do you feel **nervous** right now?	
PANAS7	To what extent do you feel **determined** right now?	
PANAS8	To what extent do you feel **attentive** right now?	
PANAS9	To what extent do you feel **afraid** right now?	
PANAS10	To what extent do you feel **active** right now?	
PANAS11	To what extent do you feel **hopeless** right now?	
PANAS12	To what extent do you feel **calm** right now?	

Modified Positive and Negative Affect Schedule, Short Form (PANAS) - EMA - Day 16, Signal 2 (Control arm)

Id	Question	Response
PANAS0	How do you feel **right now**? (Please select all that apply)	
PANAS1	To what extent do you feel **upset** right now?	
PANAS2	To what extent do you feel **hostile** right now?	
PANAS3	To what extent do you feel **alert** right now?	
PANAS4	To what extent do you feel **ashamed** right now?	
PANAS5	To what extent do you feel **inspired** right now?	

2550

CONFIDENTIAL

Participant Id	

PANAS6	To what extent do you feel **nervous** right now?	
PANAS7	To what extent do you feel **determined** right now?	
PANAS8	To what extent do you feel **attentive** right now?	
PANAS9	To what extent do you feel **afraid** right now?	
PANAS10	To what extent do you feel **active** right now?	
PANAS11	To what extent do you feel **hopeless** right now?	
PANAS12	To what extent do you feel **calm** right now?	

Modified Positive and Negative Affect Schedule, Short Form (PANAS) - EMA - Day 17, Signal 1 (Control arm)

Id	Question	Response
PANAS0	How do you feel **right now**? (Please select all that apply)	
PANAS1	To what extent do you feel **upset** right now?	
PANAS2	To what extent do you feel **hostile** right now?	
PANAS3	To what extent do you feel **alert** right now?	
PANAS4	To what extent do you feel **ashamed** right now?	
PANAS5	To what extent do you feel **inspired** right now?	
PANAS6	To what extent do you feel **nervous** right now?	
PANAS7	To what extent do you feel **determined** right now?	
PANAS8	To what extent do you feel **attentive** right now?	
PANAS9	To what extent do you feel **afraid** right now?	
PANAS10	To what extent do you feel **active** right now?	
PANAS11	To what extent do you feel **hopeless** right now?	
PANAS12	To what extent do you feel **calm** right now?	

Modified Positive and Negative Affect Schedule, Short Form (PANAS) - EMA - Day 17, Signal 2 (Control arm)

Id	Question	Response
PANAS0	How do you feel **right now**? (Please select all that apply)	
PANAS1	To what extent do you feel **upset** right now?	
PANAS2	To what extent do you feel **hostile** right now?	
PANAS3	To what extent do you feel **alert** right now?	
PANAS4	To what extent do you feel **ashamed** right now?	
PANAS5	To what extent do you feel **inspired** right now?	
PANAS6	To what extent do you feel **nervous** right now?	
PANAS7	To what extent do you feel **determined** right now?	
PANAS8	To what extent do you feel **attentive** right now?	
PANAS9	To what extent do you feel **afraid** right now?	
PANAS10	To what extent do you feel **active** right now?	
PANAS11	To what extent do you feel **hopeless** right now?	
PANAS12	To what extent do you feel **calm** right now?	

2551

2552

CONFIDENTIAL

Participant Id	

Modified Positive and Negative Affect Schedule, Short Form (PANAS) - EMA - Day 18, Signal 1 (Control arm)

Id	Question	Response
PANAS0	How do you feel **right now**? (Please select all that apply)	
PANAS1	To what extent do you feel **upset** right now?	
PANAS2	To what extent do you feel **hostile** right now?	
PANAS3	To what extent do you feel **alert** right now?	
PANAS4	To what extent do you feel **ashamed** right now?	
PANAS5	To what extent do you feel **inspired** right now?	
PANAS6	To what extent do you feel **nervous** right now?	
PANAS7	To what extent do you feel **determined** right now?	
PANAS8	To what extent do you feel **attentive** right now?	
PANAS9	To what extent do you feel **afraid** right now?	
PANAS10	To what extent do you feel **active** right now?	
PANAS11	To what extent do you feel **hopeless** right now?	
PANAS12	To what extent do you feel **calm** right now?	

Modified Positive and Negative Affect Schedule, Short Form (PANAS) - EMA - Day 18, Signal 2 (Control arm)

Id	Question	Response
PANAS0	How do you feel **right now**? (Please select all that apply)	
PANAS1	To what extent do you feel **upset** right now?	
PANAS2	To what extent do you feel **hostile** right now?	
PANAS3	To what extent do you feel **alert** right now?	
PANAS4	To what extent do you feel **ashamed** right now?	
PANAS5	To what extent do you feel **inspired** right now?	
PANAS6	To what extent do you feel **nervous** right now?	
PANAS7	To what extent do you feel **determined** right now?	
PANAS8	To what extent do you feel **attentive** right now?	
PANAS9	To what extent do you feel **afraid** right now?	
PANAS10	To what extent do you feel **active** right now?	
PANAS11	To what extent do you feel **hopeless** right now?	
PANAS12	To what extent do you feel **calm** right now?	

Modified Positive and Negative Affect Schedule, Short Form (PANAS) - EMA - Day 19, Signal 1 (Control arm)

Id	Question	Response
PANAS0	How do you feel **right now**? (Please select all that apply)	
PANAS1	To what extent do you feel **upset** right now?	
PANAS2	To what extent do you feel **hostile** right now?	
PANAS3	To what extent do you feel **alert** right now?	
PANAS4	To what extent do you feel **ashamed** right now?	
PANAS5	To what extent do you feel **inspired** right now?	

2553

2554

CONFIDENTIAL

Participant Id	

PANAS6	To what extent do you feel **nervous** right now?	
PANAS7	To what extent do you feel **determined** right now?	
PANAS8	To what extent do you feel **attentive** right now?	
PANAS9	To what extent do you feel **afraid** right now?	
PANAS10	To what extent do you feel **active** right now?	
PANAS11	To what extent do you feel **hopeless** right now?	
PANAS12	To what extent do you feel **calm** right now?	

Modified Positive and Negative Affect Schedule, Short Form (PANAS) - EMA - Day 19, Signal 2 (Control arm)

Id	Question	Response
PANAS0	How do you feel **right now**? (Please select all that apply)	
PANAS1	To what extent do you feel **upset** right now?	
PANAS2	To what extent do you feel **hostile** right now?	
PANAS3	To what extent do you feel **alert** right now?	
PANAS4	To what extent do you feel **ashamed** right now?	
PANAS5	To what extent do you feel **inspired** right now?	
PANAS6	To what extent do you feel **nervous** right now?	
PANAS7	To what extent do you feel **determined** right now?	
PANAS8	To what extent do you feel **attentive** right now?	
PANAS9	To what extent do you feel **afraid** right now?	
PANAS10	To what extent do you feel **active** right now?	
PANAS11	To what extent do you feel **hopeless** right now?	
PANAS12	To what extent do you feel **calm** right now?	

Modified Positive and Negative Affect Schedule, Short Form (PANAS) - EMA - Day 20, Signal 1 (Control arm)

Id	Question	Response
PANAS0	How do you feel **right now**? (Please select all that apply)	
PANAS1	To what extent do you feel **upset** right now?	
PANAS2	To what extent do you feel **hostile** right now?	
PANAS3	To what extent do you feel **alert** right now?	
PANAS4	To what extent do you feel **ashamed** right now?	
PANAS5	To what extent do you feel **inspired** right now?	
PANAS6	To what extent do you feel **nervous** right now?	
PANAS7	To what extent do you feel **determined** right now?	
PANAS8	To what extent do you feel **attentive** right now?	
PANAS9	To what extent do you feel **afraid** right now?	
PANAS10	To what extent do you feel **active** right now?	
PANAS11	To what extent do you feel **hopeless** right now?	
PANAS12	To what extent do you feel **calm** right now?	

2555

2556

CONFIDENTIAL

Participant Id	

Modified Positive and Negative Affect Schedule, Short Form (PANAS) - EMA - Day 20, Signal 2 (Control arm)

Id	Question	Response
PANAS0	How do you feel **right now**? (Please select all that apply)	
PANAS1	To what extent do you feel **upset** right now?	
PANAS2	To what extent do you feel **hostile** right now?	
PANAS3	To what extent do you feel **alert** right now?	
PANAS4	To what extent do you feel **ashamed** right now?	
PANAS5	To what extent do you feel **inspired** right now?	
PANAS6	To what extent do you feel **nervous** right now?	
PANAS7	To what extent do you feel **determined** right now?	
PANAS8	To what extent do you feel **attentive** right now?	
PANAS9	To what extent do you feel **afraid** right now?	
PANAS10	To what extent do you feel **active** right now?	
PANAS11	To what extent do you feel **hopeless** right now?	
PANAS12	To what extent do you feel **calm** right now?	

Modified Positive and Negative Affect Schedule, Short Form (PANAS) - EMA - Day 21, Signal 1 (Control arm)

Id	Question	Response
PANAS0	How do you feel **right now**? (Please select all that apply)	
PANAS1	To what extent do you feel **upset** right now?	
PANAS2	To what extent do you feel **hostile** right now?	
PANAS3	To what extent do you feel **alert** right now?	
PANAS4	To what extent do you feel **ashamed** right now?	
PANAS5	To what extent do you feel **inspired** right now?	
PANAS6	To what extent do you feel **nervous** right now?	
PANAS7	To what extent do you feel **determined** right now?	
PANAS8	To what extent do you feel **attentive** right now?	
PANAS9	To what extent do you feel **afraid** right now?	
PANAS10	To what extent do you feel **active** right now?	
PANAS11	To what extent do you feel **hopeless** right now?	
PANAS12	To what extent do you feel **calm** right now?	

Modified Positive and Negative Affect Schedule, Short Form (PANAS) - EMA - Day 21, Signal 2 (Control arm)

Id	Question	Response
PANAS0	How do you feel **right now**? (Please select all that apply)	
PANAS1	To what extent do you feel **upset** right now?	
PANAS2	To what extent do you feel **hostile** right now?	
PANAS3	To what extent do you feel **alert** right now?	
PANAS4	To what extent do you feel **ashamed** right now?	
PANAS5	To what extent do you feel **inspired** right now?	

2557

2558

CONFIDENTIAL

Participant Id	

PANAS6	To what extent do you feel **nervous** right now?	
PANAS7	To what extent do you feel **determined** right now?	
PANAS8	To what extent do you feel **attentive** right now?	
PANAS9	To what extent do you feel **afraid** right now?	
PANAS10	To what extent do you feel **active** right now?	
PANAS11	To what extent do you feel **hopeless** right now?	
PANAS12	To what extent do you feel **calm** right now?	

Modified Positive and Negative Affect Schedule, Short Form (PANAS) - EMA - Day 22, Signal 1 (Control arm)

Id	Question	Response
PANAS0	How do you feel **right now**? (Please select all that apply)	
PANAS1	To what extent do you feel **upset** right now?	
PANAS2	To what extent do you feel **hostile** right now?	
PANAS3	To what extent do you feel **alert** right now?	
PANAS4	To what extent do you feel **ashamed** right now?	
PANAS5	To what extent do you feel **inspired** right now?	
PANAS6	To what extent do you feel **nervous** right now?	
PANAS7	To what extent do you feel **determined** right now?	
PANAS8	To what extent do you feel **attentive** right now?	
PANAS9	To what extent do you feel **afraid** right now?	
PANAS10	To what extent do you feel **active** right now?	
PANAS11	To what extent do you feel **hopeless** right now?	
PANAS12	To what extent do you feel **calm** right now?	

Modified Positive and Negative Affect Schedule, Short Form (PANAS) - EMA - Day 22, Signal 2 (Control arm)

Id	Question	Response
PANAS0	How do you feel **right now**? (Please select all that apply)	
PANAS1	To what extent do you feel **upset** right now?	
PANAS2	To what extent do you feel **hostile** right now?	
PANAS3	To what extent do you feel **alert** right now?	
PANAS4	To what extent do you feel **ashamed** right now?	
PANAS5	To what extent do you feel **inspired** right now?	
PANAS6	To what extent do you feel **nervous** right now?	
PANAS7	To what extent do you feel **determined** right now?	
PANAS8	To what extent do you feel **attentive** right now?	
PANAS9	To what extent do you feel **afraid** right now?	
PANAS10	To what extent do you feel **active** right now?	
PANAS11	To what extent do you feel **hopeless** right now?	
PANAS12	To what extent do you feel **calm** right now?	

2559

CONFIDENTIAL

Participant Id	

Modified Positive and Negative Affect Schedule, Short Form (PANAS) - EMA - Day 23, Signal 1 (Control arm)

Id	Question	Response
PANAS0	How do you feel **right now**? (Please select all that apply)	
PANAS1	To what extent do you feel **upset** right now?	
PANAS2	To what extent do you feel **hostile** right now?	
PANAS3	To what extent do you feel **alert** right now?	
PANAS4	To what extent do you feel **ashamed** right now?	
PANAS5	To what extent do you feel **inspired** right now?	
PANAS6	To what extent do you feel **nervous** right now?	
PANAS7	To what extent do you feel **determined** right now?	
PANAS8	To what extent do you feel **attentive** right now?	
PANAS9	To what extent do you feel **afraid** right now?	
PANAS10	To what extent do you feel **active** right now?	
PANAS11	To what extent do you feel **hopeless** right now?	
PANAS12	To what extent do you feel **calm** right now?	

Modified Positive and Negative Affect Schedule, Short Form (PANAS) - EMA - Day 23, Signal 2 (Control arm)

Id	Question	Response
PANAS0	How do you feel **right now**? (Please select all that apply)	
PANAS1	To what extent do you feel **upset** right now?	
PANAS2	To what extent do you feel **hostile** right now?	
PANAS3	To what extent do you feel **alert** right now?	
PANAS4	To what extent do you feel **ashamed** right now?	
PANAS5	To what extent do you feel **inspired** right now?	
PANAS6	To what extent do you feel **nervous** right now?	
PANAS7	To what extent do you feel **determined** right now?	
PANAS8	To what extent do you feel **attentive** right now?	
PANAS9	To what extent do you feel **afraid** right now?	
PANAS10	To what extent do you feel **active** right now?	
PANAS11	To what extent do you feel **hopeless** right now?	
PANAS12	To what extent do you feel **calm** right now?	

Modified Positive and Negative Affect Schedule, Short Form (PANAS) - EMA - Day 24, Signal 1 (Control arm)

Id	Question	Response
PANAS0	How do you feel **right now**? (Please select all that apply)	
PANAS1	To what extent do you feel **upset** right now?	
PANAS2	To what extent do you feel **hostile** right now?	
PANAS3	To what extent do you feel **alert** right now?	
PANAS4	To what extent do you feel **ashamed** right now?	
PANAS5	To what extent do you feel **inspired** right now?	

PANAS6	To what extent do you feel **nervous** right now?	
PANAS7	To what extent do you feel **determined** right now?	
PANAS8	To what extent do you feel **attentive** right now?	
PANAS9	To what extent do you feel **afraid** right now?	
PANAS10	To what extent do you feel **active** right now?	
PANAS11	To what extent do you feel **hopeless** right now?	
PANAS12	To what extent do you feel **calm** right now?	

Modified Positive and Negative Affect Schedule, Short Form (PANAS) - EMA - Day 24, Signal 2 (Control arm)

Id	Question	Response
PANAS0	How do you feel **right now**? (Please select all that apply)	
PANAS1	To what extent do you feel **upset** right now?	
PANAS2	To what extent do you feel **hostile** right now?	
PANAS3	To what extent do you feel **alert** right now?	
PANAS4	To what extent do you feel **ashamed** right now?	
PANAS5	To what extent do you feel **inspired** right now?	
PANAS6	To what extent do you feel **nervous** right now?	
PANAS7	To what extent do you feel **determined** right now?	
PANAS8	To what extent do you feel **attentive** right now?	
PANAS9	To what extent do you feel **afraid** right now?	
PANAS10	To what extent do you feel **active** right now?	
PANAS11	To what extent do you feel **hopeless** right now?	
PANAS12	To what extent do you feel **calm** right now?	

Modified Positive and Negative Affect Schedule, Short Form (PANAS) - EMA - Day 25, Signal 1 (Control arm)

Id	Question	Response
PANAS0	How do you feel **right now**? (Please select all that apply)	
PANAS1	To what extent do you feel **upset** right now?	
PANAS2	To what extent do you feel **hostile** right now?	
PANAS3	To what extent do you feel **alert** right now?	
PANAS4	To what extent do you feel **ashamed** right now?	
PANAS5	To what extent do you feel **inspired** right now?	
PANAS6	To what extent do you feel **nervous** right now?	
PANAS7	To what extent do you feel **determined** right now?	
PANAS8	To what extent do you feel **attentive** right now?	
PANAS9	To what extent do you feel **afraid** right now?	
PANAS10	To what extent do you feel **active** right now?	
PANAS11	To what extent do you feel **hopeless** right now?	
PANAS12	To what extent do you feel **calm** right now?	

CONFIDENTIAL

Participant Id	

Modified Positive and Negative Affect Schedule, Short Form (PANAS) - EMA - Day 25, Signal 2 (Control arm)

Id	Question	Response
PANAS0	How do you feel **right now**? (Please select all that apply)	
PANAS1	To what extent do you feel **upset** right now?	
PANAS2	To what extent do you feel **hostile** right now?	
PANAS3	To what extent do you feel **alert** right now?	
PANAS4	To what extent do you feel **ashamed** right now?	
PANAS5	To what extent do you feel **inspired** right now?	
PANAS6	To what extent do you feel **nervous** right now?	
PANAS7	To what extent do you feel **determined** right now?	
PANAS8	To what extent do you feel **attentive** right now?	
PANAS9	To what extent do you feel **afraid** right now?	
PANAS10	To what extent do you feel **active** right now?	
PANAS11	To what extent do you feel **hopeless** right now?	
PANAS12	To what extent do you feel **calm** right now?	

Modified Positive and Negative Affect Schedule, Short Form (PANAS) - EMA - Day 26, Signal 1 (Control arm)

Id	Question	Response
PANAS0	How do you feel **right now**? (Please select all that apply)	
PANAS1	To what extent do you feel **upset** right now?	
PANAS2	To what extent do you feel **hostile** right now?	
PANAS3	To what extent do you feel **alert** right now?	
PANAS4	To what extent do you feel **ashamed** right now?	
PANAS5	To what extent do you feel **inspired** right now?	
PANAS6	To what extent do you feel **nervous** right now?	
PANAS7	To what extent do you feel **determined** right now?	
PANAS8	To what extent do you feel **attentive** right now?	
PANAS9	To what extent do you feel **afraid** right now?	
PANAS10	To what extent do you feel **active** right now?	
PANAS11	To what extent do you feel **hopeless** right now?	
PANAS12	To what extent do you feel **calm** right now?	

Modified Positive and Negative Affect Schedule, Short Form (PANAS) - EMA - Day 26, Signal 2 (Control arm)

Id	Question	Response
PANAS0	How do you feel **right now**? (Please select all that apply)	
PANAS1	To what extent do you feel **upset** right now?	
PANAS2	To what extent do you feel **hostile** right now?	
PANAS3	To what extent do you feel **alert** right now?	
PANAS4	To what extent do you feel **ashamed** right now?	
PANAS5	To what extent do you feel **inspired** right now?	

CONFIDENTIAL

Participant Id	

PANAS6	To what extent do you feel **nervous** right now?	
PANAS7	To what extent do you feel **determined** right now?	
PANAS8	To what extent do you feel **attentive** right now?	
PANAS9	To what extent do you feel **afraid** right now?	
PANAS10	To what extent do you feel **active** right now?	
PANAS11	To what extent do you feel **hopeless** right now?	
PANAS12	To what extent do you feel **calm** right now?	

Behavioural intentions (BHV) - EMA - Day 1, Signal 1

Id	Question	Response
BHV1	How likely are you to do something specifically as a result of how you feel **right now**?	
BHV2	What is it that are you likely to do?<br>We don't actively monitor responses to this question, but help is always available if you need it. [Link to support options]	

Behavioural intentions (BHV) - EMA - Day 1, Signal 2

Id	Question	Response
BHV1	How likely are you to do something specifically as a result of how you feel **right now**?	
BHV2	What is it that are you likely to do?<br>We don't actively monitor responses to this question, but help is always available if you need it. [Link to support options]	

Behavioural intentions (BHV) - EMA - Day 2, Signal 1

Id	Question	Response
BHV1	How likely are you to do something specifically as a result of how you feel **right now**?	
BHV2	What is it that are you likely to do?<br>We don't actively monitor responses to this question, but help is always available if you need it. [Link to support options]	

Behavioural intentions (BHV) - EMA - Day 2, Signal 2

Id	Question	Response
BHV1	How likely are you to do something specifically as a result of how you feel **right now**?	
BHV2	What is it that are you likely to do?<br>We don't actively monitor responses to this question, but help is always available if you need it. [Link to support options]	

CONFIDENTIAL

Participant Id	

Behavioural intentions (BHV) - EMA - Day 3, Signal 1

Id	Question	Response
BHV1	How likely are you to do something specifically as a result of how you feel **right now**?	
BHV2	What is it that are you likely to do?<br>We don't actively monitor responses to this question, but help is always available if you need it. [Link to support options]	

Behavioural intentions (BHV) - EMA - Day 3, Signal 2

Id	Question	Response
BHV1	How likely are you to do something specifically as a result of how you feel **right now**?	
BHV2	What is it that are you likely to do?<br>We don't actively monitor responses to this question, but help is always available if you need it. [Link to support options]	

Behavioural intentions (BHV) - EMA - Day 4, Signal 1

Id	Question	Response
BHV1	How likely are you to do something specifically as a result of how you feel **right now**?	
BHV2	What is it that are you likely to do?<br>We don't actively monitor responses to this question, but help is always available if you need it. [Link to support options]	

Behavioural intentions (BHV) - EMA - Day 4, Signal 2

Id	Question	Response
BHV1	How likely are you to do something specifically as a result of how you feel **right now**?	
BHV2	What is it that are you likely to do?<br>We don't actively monitor responses to this question, but help is always available if you need it. [Link to support options]	

Behavioural intentions (BHV) - EMA - Day 5, Signal 1

Id	Question	Response
BHV1	How likely are you to do something specifically as a result of how you feel **right now**?	
BHV2	What is it that are you likely to do?<br>We don't actively monitor responses to this question, but help is always available if you need it. [Link to support options]	

CONFIDENTIAL

Participant Id	

Behavioural intentions (BHV) - EMA - Day 5, Signal 2

Id	Question	Response
BHV1	How likely are you to do something specifically as a result of how you feel **right now**?	
BHV2	What is it that are you likely to do?<br>We don't actively monitor responses to this question, but help is always available if you need it. [Link to support options]	

Behavioural intentions (BHV) - EMA - Day 6, Signal 1

Id	Question	Response
BHV1	How likely are you to do something specifically as a result of how you feel **right now**?	
BHV2	What is it that are you likely to do?<br>We don't actively monitor responses to this question, but help is always available if you need it. [Link to support options]	

Behavioural intentions (BHV) - EMA - Day 6, Signal 2

Id	Question	Response
BHV1	How likely are you to do something specifically as a result of how you feel **right now**?	
BHV2	What is it that are you likely to do?<br>We don't actively monitor responses to this question, but help is always available if you need it. [Link to support options]	

Behavioural intentions (BHV) - EMA - Day 7, Signal 1

Id	Question	Response
BHV1	How likely are you to do something specifically as a result of how you feel **right now**?	
BHV2	What is it that are you likely to do?<br>We don't actively monitor responses to this question, but help is always available if you need it. [Link to support options]	

Behavioural intentions (BHV) - EMA - Day 7, Signal 2

Id	Question	Response
BHV1	How likely are you to do something specifically as a result of how you feel **right now**?	

2572

CONFIDENTIAL

Participant Id	

BHV2	What is it that are you likely to do?<br>We don't actively monitor responses to this question, but help is always available if you need it. [Link to support options]	

Behavioural intentions (BHV) - EMA - Day 8, Signal 1

Id	Question	Response
BHV1	How likely are you to do something specifically as a result of how you feel **right now**?	
BHV2	What is it that are you likely to do?<br>We don't actively monitor responses to this question, but help is always available if you need it. [Link to support options]	

Behavioural intentions (BHV) - EMA - Day 8, Signal 2

Id	Question	Response
BHV1	How likely are you to do something specifically as a result of how you feel **right now**?	
BHV2	What is it that are you likely to do?<br>We don't actively monitor responses to this question, but help is always available if you need it. [Link to support options]	

Behavioural intentions (BHV) - EMA - Day 9, Signal 1

Id	Question	Response
BHV1	How likely are you to do something specifically as a result of how you feel **right now**?	
BHV2	What is it that are you likely to do?<br>We don't actively monitor responses to this question, but help is always available if you need it. [Link to support options]	

Behavioural intentions (BHV) - EMA - Day 9, Signal 2

Id	Question	Response
BHV1	How likely are you to do something specifically as a result of how you feel **right now**?	
BHV2	What is it that are you likely to do?<br>We don't actively monitor responses to this question, but help is always available if you need it. [Link to support options]	

2573

2574

CONFIDENTIAL

Participant Id	

Behavioural intentions (BHV) - EMA - Day 10, Signal 1 (Control arm)

Id	Question	Response
BHV1	How likely are you to do something specifically as a result of how you feel **right now**?	
BHV2	What is it that are you likely to do?<br>We don't actively monitor responses to this question, but help is always available if you need it. [Link to support options]	

Behavioural intentions (BHV) - EMA - Day 10, Signal 2 (Control arm)

Id	Question	Response
BHV1	How likely are you to do something specifically as a result of how you feel **right now**?	
BHV2	What is it that are you likely to do?<br>We don't actively monitor responses to this question, but help is always available if you need it. [Link to support options]	

Behavioural intentions (BHV) - EMA - Day 11, Signal 1 (Control arm)

Id	Question	Response
BHV1	How likely are you to do something specifically as a result of how you feel **right now**?	
BHV2	What is it that are you likely to do?<br>We don't actively monitor responses to this question, but help is always available if you need it. [Link to support options]	

Behavioural intentions (BHV) - EMA - Day 11, Signal 2 (Control arm)

Id	Question	Response
BHV1	How likely are you to do something specifically as a result of how you feel **right now**?	
BHV2	What is it that are you likely to do?<br>We don't actively monitor responses to this question, but help is always available if you need it. [Link to support options]	

Behavioural intentions (BHV) - EMA - Day 12, Signal 1 (Control arm)

Id	Question	Response
BHV1	How likely are you to do something specifically as a result of how you feel **right now**?	
BHV2	What is it that are you likely to do?<br>We don't actively monitor responses to this question, but help is always available if you need it. [Link to support options]	

2575

Behavioural intentions (BHV) - EMA - Day 12, Signal 2 (Control arm)

Id	Question	Response
BHV1	How likely are you to do something specifically as a result of how you feel **right now**?	
BHV2	What is it that are you likely to do?<br>We don't actively monitor responses to this question, but help is always available if you need it. [Link to support options]	

Behavioural intentions (BHV) - EMA - Day 13, Signal 1 (Control arm)

Id	Question	Response
BHV1	How likely are you to do something specifically as a result of how you feel **right now**?	
BHV2	What is it that are you likely to do?<br>We don't actively monitor responses to this question, but help is always available if you need it. [Link to support options]	

Behavioural intentions (BHV) - EMA - Day 13, Signal 2 (Control arm)

Id	Question	Response
BHV1	How likely are you to do something specifically as a result of how you feel **right now**?	
BHV2	What is it that are you likely to do?<br>We don't actively monitor responses to this question, but help is always available if you need it. [Link to support options]	

Behavioural intentions (BHV) - EMA - Day 14, Signal 1 (Control arm)

Id	Question	Response
BHV1	How likely are you to do something specifically as a result of how you feel **right now**?	
BHV2	What is it that are you likely to do?<br>We don't actively monitor responses to this question, but help is always available if you need it. [Link to support options]	

Behavioural intentions (BHV) - EMA - Day 14, Signal 2 (Control arm)

Id	Question	Response
BHV1	How likely are you to do something specifically as a result of how you feel **right now**?	

BHV2	What is it that are you likely to do?<br><br>We don't actively monitor responses to this question, but help is always available if you need it. [Link to support options]	

Behavioural intentions (BHV) - EMA - Day 15, Signal 1 (Control arm)

Id	Question	Response
BHV1	How likely are you to do something specifically as a result of how you feel **right now**?	
BHV2	What is it that are you likely to do?<br><br>We don't actively monitor responses to this question, but help is always available if you need it. [Link to support options]	

Behavioural intentions (BHV) - EMA - Day 15, Signal 2 (Control arm)

Id	Question	Response
BHV1	How likely are you to do something specifically as a result of how you feel **right now**?	
BHV2	What is it that are you likely to do?<br><br>We don't actively monitor responses to this question, but help is always available if you need it. [Link to support options]	

Behavioural intentions (BHV) - EMA - Day 16, Signal 1 (Control arm)

Id	Question	Response
BHV1	How likely are you to do something specifically as a result of how you feel **right now**?	
BHV2	What is it that are you likely to do?<br><br>We don't actively monitor responses to this question, but help is always available if you need it. [Link to support options]	

Behavioural intentions (BHV) - EMA - Day 16, Signal 2 (Control arm)

Id	Question	Response
BHV1	How likely are you to do something specifically as a result of how you feel **right now**?	
BHV2	What is it that are you likely to do?<br><br>We don't actively monitor responses to this question, but help is always available if you need it. [Link to support options]	

CONFIDENTIAL

Participant Id	

Behavioural intentions (BHV) - EMA - Day 17, Signal 1 (Control arm)

Id	Question	Response
BHV1	How likely are you to do something specifically as a result of how you feel **right now**?	
BHV2	What is it that are you likely to do?<br>We don't actively monitor responses to this question, but help is always available if you need it. [Link to support options]	

Behavioural intentions (BHV) - EMA - Day 17, Signal 2 (Control arm)

Id	Question	Response
BHV1	How likely are you to do something specifically as a result of how you feel **right now**?	
BHV2	What is it that are you likely to do?<br>We don't actively monitor responses to this question, but help is always available if you need it. [Link to support options]	

Behavioural intentions (BHV) - EMA - Day 18, Signal 1 (Control arm)

Id	Question	Response
BHV1	How likely are you to do something specifically as a result of how you feel **right now**?	
BHV2	What is it that are you likely to do?<br>We don't actively monitor responses to this question, but help is always available if you need it. [Link to support options]	

Behavioural intentions (BHV) - EMA - Day 18, Signal 2 (Control arm)

Id	Question	Response
BHV1	How likely are you to do something specifically as a result of how you feel **right now**?	
BHV2	What is it that are you likely to do?<br>We don't actively monitor responses to this question, but help is always available if you need it. [Link to support options]	

Behavioural intentions (BHV) - EMA - Day 19, Signal 1 (Control arm)

Id	Question	Response
BHV1	How likely are you to do something specifically as a result of how you feel **right now**?	
BHV2	What is it that are you likely to do?<br>We don't actively monitor responses to this question, but help is always available if you need it. [Link to support options]	

CONFIDENTIAL

Participant Id	

Behavioural intentions (BHV) - EMA - Day 19, Signal 2 (Control arm)

Id	Question	Response
BHV1	How likely are you to do something specifically as a result of how you feel **right now**?	
BHV2	What is it that are you likely to do?<br>We don't actively monitor responses to this question, but help is always available if you need it. [Link to support options]	

Behavioural intentions (BHV) - EMA - Day 20, Signal 1 (Control arm)

Id	Question	Response
BHV1	How likely are you to do something specifically as a result of how you feel **right now**?	
BHV2	What is it that are you likely to do?<br>We don't actively monitor responses to this question, but help is always available if you need it. [Link to support options]	

Behavioural intentions (BHV) - EMA - Day 20, Signal 2 (Control arm)

Id	Question	Response
BHV1	How likely are you to do something specifically as a result of how you feel **right now**?	
BHV2	What is it that are you likely to do?<br>We don't actively monitor responses to this question, but help is always available if you need it. [Link to support options]	

Behavioural intentions (BHV) - EMA - Day 21, Signal 1 (Control arm)

Id	Question	Response
BHV1	How likely are you to do something specifically as a result of how you feel **right now**?	
BHV2	What is it that are you likely to do?<br>We don't actively monitor responses to this question, but help is always available if you need it. [Link to support options]	

Behavioural intentions (BHV) - EMA - Day 21, Signal 2 (Control arm)

Id	Question	Response
BHV1	How likely are you to do something specifically as a result of how you feel **right now**?	

BHV2	What is it that are you likely to do?<br>We don't actively monitor responses to this question, but help is always available if you need it. [Link to support options]	

Behavioural intentions (BHV) - EMA - Day 22, Signal 1 (Control arm)

Id	Question	Response
BHV1	How likely are you to do something specifically as a result of how you feel **right now**?	
BHV2	What is it that are you likely to do?<br>We don't actively monitor responses to this question, but help is always available if you need it. [Link to support options]	

Behavioural intentions (BHV) - EMA - Day 22, Signal 2 (Control arm)

Id	Question	Response
BHV1	How likely are you to do something specifically as a result of how you feel **right now**?	
BHV2	What is it that are you likely to do?<br>We don't actively monitor responses to this question, but help is always available if you need it. [Link to support options]	

Behavioural intentions (BHV) - EMA - Day 23, Signal 1 (Control arm)

Id	Question	Response
BHV1	How likely are you to do something specifically as a result of how you feel **right now**?	
BHV2	What is it that are you likely to do?<br>We don't actively monitor responses to this question, but help is always available if you need it. [Link to support options]	

Behavioural intentions (BHV) - EMA - Day 23, Signal 2 (Control arm)

Id	Question	Response
BHV1	How likely are you to do something specifically as a result of how you feel **right now**?	
BHV2	What is it that are you likely to do?<br>We don't actively monitor responses to this question, but help is always available if you need it. [Link to support options]	

CONFIDENTIAL

Participant Id	

Behavioural intentions (BHV) - EMA - Day 24, Signal 1 (Control arm)

Id	Question	Response
BHV1	How likely are you to do something specifically as a result of how you feel **right now**?	
BHV2	What is it that are you likely to do?<br>We don't actively monitor responses to this question, but help is always available if you need it. [Link to support options]	

Behavioural intentions (BHV) - EMA - Day 24, Signal 2 (Control arm)

Id	Question	Response
BHV1	How likely are you to do something specifically as a result of how you feel **right now**?	
BHV2	What is it that are you likely to do?<br>We don't actively monitor responses to this question, but help is always available if you need it. [Link to support options]	

Behavioural intentions (BHV) - EMA - Day 25, Signal 1 (Control arm)

Id	Question	Response
BHV1	How likely are you to do something specifically as a result of how you feel **right now**?	
BHV2	What is it that are you likely to do?<br>We don't actively monitor responses to this question, but help is always available if you need it. [Link to support options]	

Behavioural intentions (BHV) - EMA - Day 25, Signal 2 (Control arm)

Id	Question	Response
BHV1	How likely are you to do something specifically as a result of how you feel **right now**?	
BHV2	What is it that are you likely to do?<br>We don't actively monitor responses to this question, but help is always available if you need it. [Link to support options]	

Behavioural intentions (BHV) - EMA - Day 26, Signal 1 (Control arm)

Id	Question	Response
BHV1	How likely are you to do something specifically as a result of how you feel **right now**?	
BHV2	What is it that are you likely to do?<br>We don't actively monitor responses to this question, but help is always available if you need it. [Link to support options]	

2588

CONFIDENTIAL

Participant Id	

Behavioural intentions (BHV) - EMA - Day 26, Signal 2 (Control arm)

Id	Question	Response
BHV1	How likely are you to do something specifically as a result of how you feel **right now**?	
BHV2	What is it that are you likely to do?<br>We don't actively monitor responses to this question, but help is always available if you need it. [Link to support options]	

Abridged Credibility and Expectancy Questionnaire (CEQ) - Mid

Id	Question	Response
CEQ1	Right now, how logical does the idea of using this app to improve your distress seem to you?	
CEQ6	Close your eyes for a few moments and try to identify what you really **feel** about this app and its likely success.<br>By the end of the study, how much improvement in your distress do you really **feel** will occur?	

Abridged Revised University of Rhode Island Change Assessment Scale (URC) - Mid

Id	Question	Response
URC19	I wish I had more ideas on how to solve my problems.	
URC24	I hope that this app will contain some good advice for me.	
URC25	Anyone can talk about changing; I'm actually doing something about it.	
URC26	All this talk about psychology is boring. Why can't people just forget their problems?	
URC29	I have worries but so does the next person. Why spend time thinking about them?	
URC30	I am actively working on my problem.	

Daily log of engagement with intervention (LOG) - Intervention

Id	Question	Response
LOGa	How much time did you spend practicing mindfulness yesterday?	
LOGb	How much time did you spend being physically active yesterday?	

Page 43 of 44

HC Number: HC200466 **Version dated:** 06 Apr 2021

2589

CONFIDENTIAL

Participant Id	

	Please include any changes you made to increase your physical activity throughout the day (not just exercise workouts).	
LOGc	How much sleep did you get last night?	

Within-study exposures questionnaire (EXP) - Post

Id	Question	Response
EXP1	In the past **two weeks**, my life or routine was disrupted for some reason (ignoring anything to do with this app.)	
EXP2a	In the past **two weeks**, something **negatively** affected my mental health (ignoring anything to do with this app.)	
EXP2b	In the past **two weeks**, something **positively** affected my mental health (ignoring anything to do with this app.)	
EXP3	In the past **two weeks**, something interfered with my ability to use this app.	

UX questionnaire (UX) - Post

Id	Question	Response
UX1	I found the app easy to use.	
UX2	I found the app useful for my mental health.	
UX3	Overall, I am satisfied with the app.	
UX4a	I had no problems using the app.	
UX4b	Can you tell us what problem(s) you encountered?<br><br>Letting us know will help us improve the experience for others. Thank you!	
UX5	I trusted the information given by the app.	
UX6	Activities or actions suggested by the app **were new** for me.	
UX7	**In the past two weeks**, I have put into practice activities or actions suggested by the app.	
UX8	I **intend in the future** to put into practice activities or actions suggested by the app.	

Page 44 of 44

HC Number: HC200466 **Version dated:** 06 Apr 2021

Appendix 33 Research Partner Collaborative Research Agreement

Research Collaboration Agreement

PARTIES	
**UNSW**	The University of New South Wales ABN 57 195 873 179, a body corporate established pursuant to the *University of New South Wales Act 1989* (NSW) of UNSW Sydney NSW 2052
**Collaborators and their first-named Chief Investigator(s) (Key Personnel)**	see below

Collaborator	CI Name
Deakin University	Professor Svetha Venkatesh
Macquarie University	Professor Henry Cutler
The Australian Psychological Society Limited	Ms Ros Knight
Australian Medical Association Limited	Dr Martin Laverty

DETAILS	
**KEY DATES**	
**Project Start Date** (clause 3.2)	30 JUN 2020
**Project Completion Date** (clause 3.2)	30 JUL 2023
**CONTACT DETAILS**	
**UNSW**	Address for Notices:<br>Attention: Director, Research Grants and Contracts<br>Address: UNSW Research Grants and Contracts, The University of New South Wales, UNSW Sydney NSW 2052<br>Email: rgc@unsw.edu.au<br><br>Address for invoices:<br>As above
**Deakin University**	Address for Notices:<br>Attention: Senior Grants Officer, Deakin Research<br>Address: Deakin University, Geelong Waurn Ponds Campus, Locked Bag 20000, Geelong VIC 3220<br>Email: research-contracts@deakin.edu.au<br>Phone: 03 5227 2985<br><br>Address for invoices:<br>Susan Rose; Melissa Hale<br>Senior Management Accountant (Research), Research Finance<br>dr-finance@deakin.edu.au
**Macquarie University**	Address for Notices:

2594

	Attention: Manager, Post Award and Reporting<br>Address: C5C-17 Wally's Walk L3, Macquarie University, NSW 2109<br>Email: research.postaward@mq.edu.au<br>Phone: 02 9850 4454<br><br>Address for invoices:<br>As above
**The Australian Psychological Society Limited**	Address for Notices:<br>Attention: Chief Executive Officer<br>Address: PO Box 38 Flinders Lane VIC 8009<br>Email: ceo@psychology.org.au<br>Phone: 03 8662 3318<br><br>Address for invoices:<br>As above
**Australian Medical Association Limited**	Address for Notices:<br>Attention: Secretary General<br>Address: PO Box 6090 Kingston ACT 2600<br>Email: mlaverty@ama.com.au<br>Phone: 02 6270 5400<br><br>Address for invoices:<br>As above

2595

PAYMENTS

Payment (clause 4)

Funding Distribution Table

Institutions	30-June-2020 to 29-June-2021	30-June-2021 to 29-June-2022	30-June-2022 to 29-June-2023	Total
**UNSW**	$622,466.00	$658,586.39	$653,371.31	$1,934,424.30
**Deakin**	$941,467.85	$979,897.94	$1,019,643.90	$2,941,009.70
**Macquarie University**	$40,000.00	$40,000.00	$40,000.00	$120,000.00
**Total**	**$1,603,934.45**	**$1,678,484.33**	**$1,713,015.21**	**$4,995,434.00**

PROJECT

**Project**	Optimising treatments in mental health using AI
**Head Agreement** (clause 2 and Schedule 1)	Commonwealth Standard Grant Agreement MRFAI000028 between the Commonwealth Department of Health and UNSW fully executed on the 25 June 2020
**Key Personnel** (clause 3.4(b)	**UNSW** – Prof Helen Christensen, A/Prof Sarah Kummerfeld, Dr Warren Kaplan, Dr Jin Han, Dr Joanne Beames, Ms Jo Riley, Dr Kit Huckvale, A/Prof Jill Newby, Dr Aliza Werner-Seidler<br>**Deakin University –** Prof Svetha Venkatesh, Prof Kon Mouzakis, Prof Rajesh Vasa, A/Prof Sunil Gupta, A/Prof Santu Rana, A/Prof Truyen Tran, Dr Thomas Quinn<br>**Macquarie University –** Prof Henry Cutler, A/Prof Liz Schroeder, Dr Megan Gu<br>**The Australian Psychological Society Limited –** Ms Ros Knight<br>**Australian Medical Association Limited –** Dr Martin Laverty
**Activities** (clause 3)	As set out in Application MRFAI000028 submitted by UNSW to the Commonwealth Department of Health on the 3 March 2020 for the program "MRFF Applied Artificial Intelligence Research in Health"

EXECUTED AS AN AGREEMENT

Executed for and on behalf of The University of New South Wales:

....
Signature of Authorised Representative

....
Print Name

....
Position

....
Date of Signing

Executed for and on behalf of Deakin University

Signature of Authorised Representative

Print Name

Position

Date of Signing

Executed for and on behalf of Macquarie University

Signature of Authorised Representative

Print Name

Position

Date of Signing

Executed for and on behalf of The Australian Psychological Society Limited

Signature of Authorised Representative

Print Name

Position

Date of Signing

Executed for and on behalf of Australian Medical Association Limited

Signature of Authorised Representative

Print Name

Position<br><br>Date of Signing

Recitals

1. UNSW has entered into a Commonwealth Standard Grant Agreement between the Commonwealth Department of Health and UNSW fully executed on 25 June 2020 to undertake a study: “Optimising treatments in mental health using AI”. That Commonwealth Standard Grant Agreement is referred to as the “Head Agreement” throughout this Research Collaboration Agreement, and a copy appears as Schedule 1 hereto.

2. UNSW is collaborating with the Collaborators to undertake the Project under the terms and conditions of this Research Collaboration Agreement.

Terms

1. Definitions and interpretation

1.1 All defined terms in the Head Agreement will have the same meaning in this Research Collaboration Agreement unless otherwise specified in this Research Collaboration Agreement.

1.2 In this Research Collaboration Agreement the following additional definitions will apply except where the context otherwise requires:

Activities means the program of work to be performed by the Collaborators referred to in the **Details**;

Background Intellectual Property means Intellectual Property Rights owned by or licensed to a Party as at the Project Start Date, or acquired or developed by a Party during the course of the Project independently of the Project, which that Party has the right to license to third Parties and which are necessary for the performance of the Project, including those specified in Schedule 2 or as subsequently notified in writing by the Parties;

Confidential Information means any information which the Parties agree in writing is confidential and that is by its nature confidential but does not include information which:

(a) is or becomes part of the public domain unless it came into the public domain by a breach of confidentiality;
(b) is obtained lawfully from a third party without any breach of confidentiality;
(c) is already known by the recipient Party (as shown by its written record) before the date of disclosure to it;
(d) is independently developed by an employee of the recipient Party who has no knowledge of the disclosure under this Agreement.

Details means the matters set out in the table on the front page(s) of this Research Collaboration Agreement;

Grant means the funds provided to UNSW for the Project in accordance with the Head Agreement;

GST has the same meaning as in the GST Law;

GST Law has the same meaning as in the *A New Tax System (Goods and Services Tax) Act* 1999 (Cth) as amended or replaced from time to time;

2602

Head Agreement means the agreement specified in the Details a copy of which is attached as Schedule 1;

Intellectual Property Rights or **Intellectual Property** means all intellectual property rights, including without limitation:

(a) patents, copyright, rights in circuit layouts, registered designs, plant varieties, trade marks (including service marks), all other rights resulting from intellectual activity in the industrial, scientific, literary or artistic fields and the right to have confidential information kept confidential; and

(b) any application or right to apply for registration of any of those rights;

Key Personnel means the people specified as such in the Details that the Collaborators will use to perform the Activities;

Non-Severable means any improvement that cannot be exploited or used separately to the Background Intellectual Property;

Payment means the amounts specified in the Details which UNSW will pay to the Collaborators for performing the Activities;

Parties means the parties to this Research Collaboration Agreement and their respective successors and permitted assigns, and **"Party"** means any one of them;

Project means the program of research and development set out in the **Details**;

Project Intellectual Property means all Intellectual Property Rights created or developed as part of performing the Project, but does not include Intellectual Property Rights in Background Intellectual Property;

Project Material means all material including but not limited to:

(a) documents, computer software, equipment and data stored by any means; and

(b) all material and subject matter in which the rights referred to in paragraphs (a) to (g) of the definition of "Intellectual Property Rights" in this clause subsist,

which is created or developed for the sole purpose of undertaking the Project.

Student means a student who is enrolled at UNSW or with a Collaborator and involved in the Project;

Tax Invoice has the same meaning as in the GST Law;

Taxable Supply has the same meaning as in the GST Law; and

Term means the term of this Research Collaboration Agreement provided in clause 2.

1.3 In the event of any discrepancy or inconsistency between this Research Collaboration Agreement and the Head Agreement the following order of precedence will apply to resolve the issue:

(c) the Head Agreement;

(d) this Research Collaboration Agreement; and

(e) the schedules and attachments forming part of this Research Collaboration Agreement.

2603

2604

1.4 Headings in this Research Collaboration Agreement are for convenience only and do not alter interpretation.

2. Term

2.1 This Research Collaboration Agreement will be effective for the term of the Head Agreement.

3. Collaborator obligations

3.1 The parties agree that any and all terms, conditions, obligations and indemnities imposed on UNSW under the Head Agreement are incorporated by reference into this Research Collaboration Agreement and will apply to the Collaborator(s) as if they were UNSW under the Head Agreement.

3.2 The Collaborators:

(a) agree to do all things necessary to enable UNSW to comply with its obligations under the Research Collaboration Agreement; and

(b) must not do anything that may cause UNSW to breach its obligations, or prevent or impede UNSW from complying with its obligations, under the Head Agreement.

3.3 The Collaborators will:

(a) start the Activities on the Project Start Date;

(b) use reasonable endeavours to complete the Project on or near the Project Completion Date;

(c) provide their contributions to the Project in accordance with the Head Agreement unless otherwise specified in this Research Collaboration Agreement;

(d) provide the reports required to be submitted by UNSW under the Head Agreement to the extent that their Activities relate to those reports; and

(e) ensure that their expenditure of the Grant is in accordance with all relevant conditions of the Head Agreement.

3.4 The Collaborators will perform the Activities:

(a) in accordance with the Details and the Head Agreement;

(b) using Key Personnel (if any);

(c) with due skill and care in accordance with generally accepted professional, scientific and ethical principles and standards applicable to the Project; and

(d) in compliance with all applicable laws and regulations.

3.5 The Collaborators will:

(a) notify UNSW if any of its Key Personnel (other than a Student) becomes unable to perform the Activities; and

(b) use reasonable endeavours to replace its Key Personnel (other than a Student) with other suitable personnel acceptable to UNSW.

2605

4. Payment

4.1 UNSW will make Payment(s) to the Collaborators in accordance with the Details provided that UNSW receives its payment of the Grant under the Head Agreement and subject to UNSW receiving a satisfactory tax invoice in respect of the Payment(s) from the Collaborators.

4.2 Unless specified otherwise, all monetary amounts expressed in this Research Collaboration Agreement are exclusive of GST.

4.3 Payments will be due by the date specified on each Tax Invoice or if no due date is specified, within 30 days after the Tax Invoice is issued.

4.4 If any supply under this Research Collaboration Agreement is a Taxable Supply, the party making the supply may, in addition to any payment for the supply, recover the amount of the GST applicable to the supply.

4.5 Any amount of GST payable for a supply will be payable at the same time as the payment for the supply to which it relates.

4.6 UNSW will be entitled to defer part or full payment to a Collaborator of any instalment under clause 4.1 until such time as that Collaborator has performed to the reasonable satisfaction of UNSW that part of the Activities to which the instalment relates.

4.7 Notwithstanding clause 4.6, UNSW will not be entitled to defer payment of an instalment where non-performance of part of the Activities by the Collaborator is a result of a failure by UNSW to perform its obligations under this Research Collaboration Agreement or under the Head Agreement.

5. Intellectual Property

5.1 The Parties agree that the ownership of Background Intellectual Property is not affected by this Research Collaboration Agreement and that all Background Intellectual Property remains the property of the Party that makes it available for the purpose of carrying out the Project.

5.2 The parties agree that any Non-Severable adaptations, modifications or improvements made to a party's Background Intellectual Property Rights will be owned by the owner of the Background Intellectual Property Rights. In the case where two parties jointly own the Background Intellectual Property Rights, such Non-Severable adaptations, modifications or improvements will be owned by them in equal shares. To the extent necessary, the other parties hereby assign any right, title or interest they may have in those Non-Severable improvements to any Background Intellectual Property Rights to the owner(s) of the relevant Background Intellectual Property Rights upon creation.

5.3 Each Party grants to each other Party a royalty-free, non-exclusive, non-transferrable licence to use its Background Intellectual Property to the extent necessary to carry out the Project.

5.4 Each Collaborator grants a permanent, irrevocable, free, world-wide, non-exclusive licence (including the right to sub-licence) to UNSW in respect of their relevant Background Intellectual Property to satisfy clause 17.3 of the Head Agreement but for no other purpose.

5.5 No representations or warranties are made or given in relation to Background Intellectual Property, however each Party making available Background Intellectual Property acknowledges that to the best of its knowledge, such Background Intellectual Property when used in accordance with this Research Collaboration Agreement will not infringe any third party Intellectual Property rights.

5.6 The Parties agree that all rights, title and interest in the Project Intellectual Property (except for copyright in a Student thesis) will be owned by Deakin University and UNSW in equal shares as tenants in common.

5.7 The Parties agree that copyright in a Student thesis will be owned by the Student but the Party where the Student is enrolled will ensure that the Student enters into written arrangements which are consistent with and enable that Party to give effect to the terms of clause 5 of this Research Collaboration Agreement before the Student commences any Activities on the Project.

5.8 The administration and management of the Project Intellectual Property will comply with the *National Principles of Intellectual Property Management for Publicly Funded Research*.

5.9 All Parties are committed to appropriate recognition of contributions to invention and exploitation of Intellectual Property for the benefit of the Australian community.

5.10 The Parties each agree to ensure that their respective staff and Students working on the Project promptly provide to UNSW written notice (within a reasonable time) of any Project Intellectual Property that may have potential commercial value if and when such staff and Students become aware of such Project Intellectual Property.

5.11 The Parties who own Project Intellectual Property (as determined in accordance with clause 5.5) will consult and decide what (if any) measures should be taken to protect the Project Intellectual Property and negotiate in good faith and using all best endeavours to agree the terms of any program of commercialisation arising from the Project Intellectual Property so as to fairly share in any commercial return associated with the Project and the Project Intellectual Property.

5.12 Having regard to any requirements to protect potentially commercially valuable Project Intellectual Property, each Party grants to each other Party a non-exclusive, non-transferable, perpetual, royalty free, worldwide licence to use the Project Intellectual Property they own for:

(a) non-commercial research, education and training purposes; and

(b) publication purposes (subject to clause 6 of this Research Collaboration Agreement).

5.13 Deakin University grants a permanent, irrevocable, free, world-wide, non-exclusive licence (including the right to sub-license) to UNSW in respect of its interest in the Project Intellectual Property to satisfy Supplementary Term 3.4 and clause 17.3 of the Head Agreement but for no other purpose.

6. Publication

6.1 Subject to clause 7.1 each Collaborator agrees to provide UNSW with any publications resulting from an Activity and its related data in order for UNSW to comply with the obligations under the Head Agreement in relation to dissemination of research findings for the purposes of the MRFF Grant and of the *Lifting Clinical Trials and Registries Capacity Grant Program*.

6.2 The Parties are entitled to publish the results of the Project subject to clause 6.4.

6.3 The Parties acknowledge that a Student may include the results of the Project in whole or in part in the Student's thesis, in which case the non-enrolling institution(s) whose Confidential Information and/or Intellectual Property will be prejudiced if it is published in the Student's thesis may reasonably request that the thesis be submitted to examiners in confidence and that the thesis be held in restricted confidential storage in accordance with the enrolling institution's applicable regulations, by-laws and procedures. Each party will endeavour to keep any period of restriction for a PhD thesis to a minimum.

6.4 The publishing Party will provide a copy of the proposed publication to each other Party within 28 days in advance of submitting for publication. The other Parties may provide comments and/or reasonable amendments to the publication to protect their Confidential Information and/or Intellectual Property provided they are given to the publishing Party in writing no later than 14 days before the publication is made. If no such comments or amendments are provided

within the 14 day period the publishing Party can make the publication subject to any applicable requirements under the Head Agreement.

7. Confidentiality

7.1 Each Party acknowledges that all Confidential Information disclosed by one Party to the other, whether existing prior to the commencement of the Project or created in the course of the Project, will be kept confidential and shall not be disclosed to any third party without the prior written consent of the disclosing party, such consent not to be unreasonably withheld or delayed.

7.2 Notwithstanding clause 7.1, UNSW may disclose Confidential Information, including the terms of this Agreement, if required by the Commonwealth Department of Health under the terms of the Head Agreement and the Parties may disclose Confidential Information to their employees, contractors and Students involved in the Project, or their related entities, on a need to know basis as may be necessary for the purposes of this Research Collaboration Agreement provided that each such recipient is made aware of the confidential nature of the information and bound to keep the information in confidence. Each Party may disclose its Confidential Information and any other Party's Confidential Information if required by law but, if possible, it must inform the relevant other Parties first and use reasonable endeavours to limit the terms of that disclosure as reasonably requested.

7.3 The Parties acknowledge the obligations of each other Party under their respective statutes to deposit in the library a copy of a Student's completed thesis or work submitted for a higher degree. Nothing in this Agreement affects the operation of those statutes or creates any obligations contrary to those statutes.

8. Conflict of Interest

8.1. The Parties warrant that to the best of their knowledge, as at the date of this Research Collaboration Agreement, there is no Conflict of Interest which will affect their Key Personnel's conduct of the Activity.

8.2. If a Party becomes aware of a Conflict of Interest during the term of this Research Collaboration Agreement, that Party will notify UNSW immediately, who will notify the Commonwealth Department of Health in writing of the full details of that Conflict of Interest if required and the steps UNSW proposes to resolve or otherwise deal with the Conflict of Interest. The Parties agree to cooperate with each other to comply with any steps reasonably required by the Commonwealth Department of Health to resolve or otherwise deal with that Conflict of Interest.

9. Termination and Reduction

9.1. UNSW may terminate this Research Collaboration Agreement if:

(a) the Commonwealth Department of Health ceases to provide Funding for the Project or the Head Agreement is terminated for any reason, in which case UNSW will notify the Collaborators and the Parties will arrange to discuss available options regarding the Project and this Research Collaboration Agreement; or

(b) a Collaborator breaches a material term of this Research Collaboration Agreement and such breach is not remedied within 30 days of written notice of the breach by UNSW to the Collaborator.

9.2. This Research Collaboration Agreement may be terminated:

(a) at any time by the Parties mutual written agreement; or

(b) if the Project is wholly terminated.

9.3. If UNSW receives notice that a Collaborator wishes to withdraw its involvement in the Project UNSW will seek, in accordance with the Head Agreement, the remaining Parties' consent to terminate this Research Collaboration Agreement, or continue the Project with the remaining Collaborator. Where the Parties elect to proceed with the Project they will do all things necessary to amend this Research Collaboration Agreement to reflect the new arrangements.

9.4. If the Commonwealth Department of Health reduces the scope of the Head Agreement, the Project or the Funding, UNSW will notify the Collaborators in writing and the Parties agree that this Research Collaboration Agreement will be similarly reduced.

9.5. Upon termination or reduction, the Parties will stop or reduce performance of the Project, take all reasonable steps to minimise loss resulting from the termination or reduction, continue to perform any Project obligations which are not affected by the reduction, and each Collaborator will reasonably assist UNSW to comply with a request from the Commonwealth Department of Health for the Funding to be repaid if that request arises from the Collaborator's conduct. The Parties will also return all Confidential Information and property belonging to the other Parties within 14 days of the termination date.

9.6. No Party will be liable to the other upon termination of this Research Collaboration Agreement for any compensation for loss of prospective opportunities or benefits that would have been conferred on another Party but for the termination or reduction in scope of this Research Collaboration Agreement.

10. Indemnity and Insurance

10.1. Each Collaborator indemnifies (and agrees to keep indemnified) UNSW and UNSW's personnel ("those indemnified") from and against any:

(a) cost or liability incurred by those indemnified;

(b) loss of or damage to property of those indemnified;

(c) loss or expense incurred by those indemnified in dealing with any claim against them, including legal costs and expenses on a solicitor/own client basis and the cost of time spent, resources used, or disbursement paid by those indemnified

arising from any claim that the Commonwealth Department of Health makes against UNSW under the Head Agreement to the extent such loss or damage was contributed to by the Collaborator.

10.2 In this clause 10 'fault' means any negligent or unlawful act or omission or wilful misconduct.

10.3 The Collaborators' liability to indemnify those indemnified under this clause 10 will reduce proportionately to the extent that any act or omission involving fault on the part of those indemnified contributed to the relevant, liability, loss or damage or loss or expense.

10.4 The right of those indemnified to be indemnified under this clause 10 is in addition to and not exclusive of, any other right , power or remedy provided by law , but those indemnified are not entitled to be compensated in excess of the amount of the relevant cost, liability, loss, damage or expense.

10.5 The Parties will have the equivalent insurance required of UNSW under the Head Agreement.

11. Dispute resolution

11.1 If any dispute or difference arises in connection with this Research Collaboration Agreement then the Parties will negotiate in good faith to resolve it.

2614

12. Notices

12.1 Any notice to be given by either Party will be in writing and forwarded to the other Party either by email, post or facsimile transmission. The address for notices is as set out in the Details or as last notified by the recipient in writing.

13. Miscellaneous

13.1 This Research Collaboration Agreement is governed by the laws of the State of New South Wales and the Parties submit to the non-exclusive jurisdiction of the courts of New South Wales.

13.2 This Research Collaboration Agreement may only be amended in writing by the Parties.

13.3 The Collaborators may not assign the rights and obligations arising under this Research Collaboration Agreement without the prior written consent of UNSW.

13.4 The Parties are independent contracting parties, and nothing in this Research Collaboration Agreement makes a Party the agent or legal representative of any other Party for any purpose or grants a Party any authority to assume or to create any obligation on behalf of or in the name of any other Party.

13.5 This Research Collaboration Agreement constitutes the entire agreement and understanding between the Parties with respect to the subject matter of this Research Collaboration Agreement.

2615

Schedule 1 – Head Agreement

(See **Appendix 33 - Funding Agreement**)

Appendix 34 Commonwealth Standard Grant Agreement

Australian Government

Department of Health

Commonwealth Standard Grant Agreement

between the Commonwealth represented by

Department of Health

and

UNIVERSITY OF NEW SOUTH WALES

MRFF Applied Artificial Intelligence Research in Health
Standard Grant Agreement February 2020 Page 1 of 41

Contents

Grant Agreement MRFAI000028 3
Parties to this Agreement 3
Scope of this Agreement 4
Grant Details MRFAI000028 5
A. Purpose of the Grant 5
B. Activity 5
C. Duration of the Grant 8
D. Payment of the Grant 10
E. Reporting 11
F. Party representatives and address for notices 12
G. Activity Material 12
Supplementary Terms 13
Schedule 1: Commonwealth Standard Grant Conditions 24
Signatures 33
Commonwealth 33
Grantee 33
Schedule 2 Reporting requirements 34

Grant Agreement MRFAI000028

Once completed, this document, together with the Grant Details and the Commonwealth Standard Grant Conditions (Schedule 1), forms an Agreement between the Commonwealth and the Grantee.

Parties to this Agreement

The Grantee

Full legal name of Grantee	UNIVERSITY OF NEW SOUTH WALES
Legal entity type (e.g. individual, incorporated association, company, partnership, etc)	Other Incorporated Entity
Trading or business name	UNSW
Any relevant licence, registration or provider number	Not applicable
Australian Business Number (ABN) or other entity identifiers	57195873179
Australian Company Number (ACN)	
Registered for Goods and Services Tax (GST)?	Yes
Date from which GST registration was effective?	23/01/2000
Registered office address	CHANCELLERY UNSW<br>HIGH ST<br>KENSINGTON NSW 2033<br>Australia
Relevant business place	384-392 Victoria St<br>DARLINGHURST NSW 2010<br>Australia

The Commonwealth

The Commonwealth of Australia represented by the
Department of Health
of Sirius Building, Furzer Street, Woden Town Centre ACT 2606
ABN 83 605 426 759

The Department of Industry, Science, Energy and Resources will manage the Agreement on behalf of the Department of Health.

Background

The Commonwealth has agreed to enter this Agreement under which the Commonwealth will provide the Grantee with a Grant for the purpose of assisting the Grantee to undertake the associated Activity.

The Grantee agrees to use the Grant and undertake the Activity in accordance with this Agreement and the relevant Grant Details.

Scope of this Agreement

This Agreement comprises:

(a) this document;

(b) the Supplementary Terms (if any);

(c) the Standard Grant Conditions (Schedule 1);

(d) the Grant Details;

(e) any other document referenced or incorporated in the Grant Details.

If there is any ambiguity or inconsistency between the documents comprising this Agreement in relation to a Grant, the document appearing higher in the list will have precedence to the extent of the ambiguity or inconsistency.

This Agreement represents the Parties' entire agreement in relation to the Grant provided under it and the relevant Activity and supersedes all prior representations, communications, agreements, statements and understandings, whether oral or in writing.

Certain information contained in or provided under this Agreement may be used for public reporting purposes.

Grant Details MRFAI000028

A. Purpose of the Grant

The Grant is being provided as part of the MRFF Applied Artificial Intelligence Research in Health grant opportunity.

The objectives of the grant opportunity are to:
• translate or implement innovative AI technologies into health applications that benefit multiple health disciplines/areas
• involve consumers in the research journey to ensure the research is applicable to the needs of the Australian community
• increase AI workforce capacity and capability, particularly in relation to health, through cross-sector and interdisciplinary collaboration.

The intended outcome of the grant opportunity is:
• to promote the application of novel AI technologies and methodologies to cross-sectoral and interdisciplinary health research that will transform health care and outcomes through improved preventive, diagnostic and treatment approaches.

The MRFF, established under the *Medical Research Future Fund Act 2015* (MRFF Act), provides grants of financial assistance to support health and medical research and innovation to improve the health and wellbeing of Australians. It operates as an endowment fund with the capital preserved in perpetuity. At maturity, the MRFF will reach $20 billion. The MRFF provides a long-term sustainable source of funding for endeavours that aim to improve health outcomes, quality of life and health system sustainability.

This MRFF investment is guided by the Australian Medical Research and Innovation Strategy 2016–2021 (the Strategy) and related set of Australian Medical Research and Innovation Priorities 2018–2020 (the Priorities), developed by the independent and expert Australian Medical Research Advisory Board following extensive national public consultation.

In 2019-20 Budget, the Government announced its continued commitment to support lifesaving medical research with a $5 billion 10-year investment plan for the MRFF. The Investment Plan will place Australia at the leading edge of research in areas like genomics and will support the search for cures and treatments, including for rare cancers. The plan is underpinned by four key themes – patients, researchers, translation and missions.

The MRFF Applied Artificial Intelligence Research in Health forms part of the $20 billion Medical Research Future Fund.

B. Activity

The Activity is made up of the Grantee's project and all eligible project activities as specified in these Grant Details.

In undertaking the Activity, the Grantee must comply with the requirements of the grant opportunity guidelines (as in force at the time of application).

The Grantee must notify the Commonwealth about events relating to the project and provide an opportunity for the Minister or their representative to attend.

2628

The Grantee must ensure that the grant from the MRFF is appropriately badged and acknowledged in any correspondence, public announcement and publicly available materials including: reporting of key findings; presentations; and publications relating to this activity.

Project title

Optimising treatments in mental health using AI

Project scope and description

Project description:
Our primary project is to address the extensive psychological distress present in University students in Australia prior to their university examinations. Distress, anxiety and depression lead to drop out rates that are particularly concerning to university administrators, their students and families. In Australia one survey of 5,000 students reported that 25% were experiencing severe levels of psychological distress. Psychological distress is linked to poorer outcomes, including attrition and impaired academic performance, higher rates of alcohol intake, cigarette smoking and suicidal tendencies, and the development of more serious mental health disorders.

We aim to use AI-driven adaptive trial methods to determine which of a series of therapeutic interventions lead to best outcomes in this group; critically evaluate the value of using AI adaptive methods in a mental health context (e.g. in terms of resource impacts); explore whether these therapies benefit some groups more than others (e.g. first episode vs chronic/multiple episodes); and identify specific student characteristics that are associated with better response to therapy. We will evaluate a set of digitally delivered, evidence-based therapies based on mindfulness, cognitive behaviour and physical activity.

The project will be conducted over 36 months, recruiting 48 University students every 2 weeks. Four interventions will be offered: sleep hygiene, mindfulness, physical activity and a no treatment control. AI and software will be developed in Phase 1 to determine which treatments ameliorate symptoms quickly. In Phase 2, we will then examine which treatments work best for particular subgroups of students by expanding and developing AI further. We will also examine which personal characteristics of which groups are most effective. The project will build AI that can be useful in other areas of psychiatry (i.e. determining which of many medications is best), neuroscience and addiction, in all areas where treatment type is uncertain. The new AI will quickly offer personalised treatments to patients. The project, which builds on our successful AI/Digital Phenotyping platform, adds new AI to allow the optimisation of treatments across a range of disciplines.

Activities:
Governance and translational plan activities will be commenced and put in place within three months of project kick-off. This will include MOUs with our clinical partners (the AMA, Australian Psychological Society, Macquarie University Centre for Health Economics, the Lived Experience Panels at the Black Dog Institute, and Garvan Institute). Consumers will be involved at all levels: reviewing the interventions, providing feedback on the research protocol, commenting on initial results, success factors, data, and dissemination practices, and our ethics, through our Knowledge Translation Research Plan and our Risk Management Plan.
Students will be recruited through targeting social media and through promotion via Deakin University's student portal and via UNSW university channels, and clinic/counselling centre or departments. Each student will be offered one of four arms: Sleep Hygiene, Mindfulness, Physical Activity, or a no treatment control condition, each delivered through our app and platform.

2629

Participants will be sent questions/surveys, and smartphone data collected. The Primary Outcome Measure is the Depression Anxiety Stress Scale (DASS-21) (total score).
The development of efficient intervention design at a population level will require software development activities during the 0-18 month period, to identify the most promising intervention as quickly as possible maximising statistical power, the development of design frameworks and platform for the delivery of the adaptive trials; and the development of predictive markers from the data streams arising from volume of individual data (phenotype data) collected from the mobile phone technology.
The translational plan activity over 36 months will deliver the development of clinical practice guidelines, webinars, conferences and forums, digital toolkits, to improve clinical practice, based on the findings of the study; a plan to transfer the optimisation AI into other disciplines, a training program in mental health AI, the pilot linkage of genetic material to smartphone data and the development of a commercialisation pathway for the software.

Project outcomes

The expected project outcomes are consistent with the objectives of the grant:

Health applications that benefit multiple health disciplines
• The project will yield significant knowledge about how to improve distress, anxiety and depression at speed in students at University, for use by counsellors, psychologists, and students.
• These benefits can be extended to adolescent, and older adult populations.
• The AI can be used and applied to all behavioural disorders where choice of treatment is uncertain. The AI developed through the project will offer new techniques and platforms that will be available to other AI/Mental Health/Psychiatry/Neuroscience groups nationally. The new AI offered by the project includes: Protocols to deliver AI based adaptive trials for health domains (applicable to other treatment trials); Software and architectures for digital phenotyping that can collect sensor data and signals; Validated AI methods for accelerated experimental design in the health setting; and Advanced machine learning methods providing summary statistics at individual level turning raw sensor data into useful clinical insights that can be integrated into digital interventions.

Research outcomes that are relevant to needs and priorities of consumers
• The project delivers outcomes that are a priority for the Australian public. In Australia, there is intense interest in better treatment and services for mental health conditions. The Victorian Commission on Mental Health Services, and the Productivity Commission have both addressed the need to improve the quality of treatments. The Wellcome Trust in the UK has dedicated £200M to develop better treatments. The AI developed in this project, could well be of benefit to the Trust, and used globally.

Increase in AI capability in Australia
• The project will establish Australia's experimental trials at the forefront of international clinical practice and research.
• The project will consolidate and extend our research teams capacity for interdisciplinary collaboration, across Deakin and UNSW, and partner organisations.
• The project enhances the capacity and capability of AI translational research by bringing together young medical and AI researchers to work together, and to build a translational research program in AI/Mental Health. This group will serve as the vanguard of this capacity in Australia.

Partnerships
The project will also establish and consolidate partnerships with clinicians, and consumers. It will

directly involve, through the Advisory Board of the project, the peak national medical and psychological organisations in Australia. It will engage and foster consumer-led research. Through its translational plan, it will influence policy and practice in treatment services across primary care, university counselling services, psychological and psychiatric practice.

Dissemination of Research Findings

The Grantee will ensure that appropriate safeguards are in place to protect patient privacy, Intellectual property and commercially confidential information for all Activities funded under this Project.

The Grantee is encouraged to ensure that research activities are published and translated into clinical practice. Types of research dissemination may include, making lay summaries available for research participants, and presenting at specific forums.

The Grantee is also encouraged to publish de-identified research data in an open access repository and in accordance with best practice

C. Duration of the Grant

The Activity starts on 30 June 2020 and ends on 29 June 2023, which is the **Activity Completion Date**.

The Agreement ends on 06 December 2023 which is the **Agreement End Date**.

Activity Schedule

In undertaking the Activity, the Grantee will meet the following milestones by the due dates.

Milestone number	Milestone name and desciption	Due date
001	Milestone 1 Performance Indicators<br>• Management Committee, and 5 subcommittees established (G1)<br>• Recruitment of key personnel (G1)<br>• Development of a Knowledge Translation Research Plan (see Details, page 11) (G1)<br>• Project and Risk Management Plan put into place and review schedule commenced (G1)<br>• Data Monitoring and Safety Board Established (G1)<br>• Ethics completed (D1)<br>• Development of the trial and recruitment protocol completed, and student recruitment started (D1)<br>• Preliminary AI and platform software systems completed (S1)<br>• Registration of the Trial completed; Research Protocol Paper in submission (O1)<br>• Translation Plan in progress; draft report (O1)<br>• Summary report describing progress across the project in readiness for next milestones (O1)	31/10/2020

2634

Milestone number	Milestone name and desciption	Due date
002	Milestone 2 Performance Indicators<br>• Recruitment of key personnel (G2)<br>• Phase 1, Stage 1 trial started (D2)<br>• AI algorithm and platform updated systems delivered (S2)<br>• Phase 1, Stage 1 started, recruitment continues (T1)<br>• Phase 1, Stage 1 trial ends and preliminary results of intervention efficacy prepared (T1)<br>• Summary Report on outcomes, approved by Management and disseminated to Advisory (O2)<br>• Brief prepared for Health Economics Partner (02)	28/02/2021
003	Milestone 3a Performance Indicators<br>• MOU and agreements across the organisations participating in next milestone (G3)<br>• Phase 1, Stage 2 trial started, recruitment continues (D3)<br>• Software platform complete, capable of delivery of large-scale population trials (S3)<br>• Methods for selecting optimal strategy across population in health settings established (S3)<br>• Validated AI optimisation algorithms refined and completed (S3)<br>• Preliminary AI and software systems to deliver adaptive interventions in sub-groups delivered (S4)<br>• Validated ML algorithms for extracting patterns from data streams delivered (S4)<br>• Initial sub-group analysis delivered (S4)	31/12/2021
004	Milestone 3b Performance Indicators<br>• Phase 1 Stage 2 trial starts (T2)<br>• Optimal interventions identified across the population at end of Trial (T2)<br>• Research paper on the AI and software system design for population based adaptive trials (O3)<br>• Research paper on results of the optimal intervention design in mental health (O3)<br>• Training manuals/toolkits for carers/clinicians/descriptions of model for potential users (O3)<br>• Public report on outcomes generated published and disseminated to relevant partners/clinicians (O3)<br>• Discussions initiated with MPT connect, venture capitalists and others around commercialisation (O3)	31/12/2021

2635

Milestone number	Milestone name and desciption	Due date
005	Milestone 4 Performance Indicators<br>• Phase 2, Stage 1 trial started, recruitment continues (D4), genetic material taken from 100 participants<br>• Updated AI integrated with Software platform delivered (S5)<br>• Early results for optimal interventions across sub-groups established (T3)<br>• Public Report on outcomes generated published and disseminated (O4)<br>• Papers in preparation (O4)	30/06/2022
006	Milestone 5 Performance Indicators<br>• Phase 2, Stage 2 trial starts, recruitment continues (D5)<br>• Updated AI integrated with Software platform delivered (S6)<br>• Delivered Software and platform for sub-group discriminated trials (S6)<br>• Delivered validated AI/ML algorithms to accelerate experimental design incorporating context (S6)<br>• Optimal interventions across sub-groups established (T4)<br>• Research paper - protocols and frameworks for delivering AI-driven trials for sub-populations (O5)<br>• Research paper - methods for selecting optimal strategy in a sub-population for health settings (O5)<br>• Review of clinician's views of service model (O5)<br>• Continued discussions with partners, funders, etc (G5)<br>• Commercialisation pipeline report and final report (O5)	29/06/2023
007	Milestone 6 Performance Indicator<br>• Final report delivered (O5)	29/06/2023

D. Payment of the Grant

The total amount of the Grant is $4,995,434 (plus GST if applicable).

The Grant will be provided at up to 100.00 per cent of eligible expenditure as defined in the grant opportunity guidelines subject to availability of Program funds.

The Grant will be paid in accordance with clause ST2.

The Grant will be paid according to the following schedule. Payments are subject to satisfactory progress on the project and compliance by the Grantee with its obligations under this Agreement.

Payment event	Anticipated payment date	Payment amount (GST excl)
Initial payment	30/06/2020	$1,981,470.01
Progress payment	28/02/2021	$1,289,605.99
Progress payment	28/02/2022	$1,724,358

MRFF Applied Artificial Intelligence Research in Health
Standard Grant Agreement February 2020 Page 10 of 41

2638

Total	$4,995,434

Invoicing

The Grantee agrees to allow the Commonwealth to issue it with a Recipient Created Tax Invoice (RCTI) for any taxable supplies it makes in relation to the Activity.

E. Reporting

The Grantee agrees to provide the following reports to the Commonwealth representative in accordance with the reporting requirements (Schedule 2).

Report type	Period start date	Period end date	Agreed evidence	Due date
First progress report	30/06/2020	31/12/2020	Satisfactory completion of first progress report	31/01/2021
Compliance with working with children obligations	30/06/2020	30/06/2021	Working with Children Annual Statement of Compliance	30/07/2021
Second progress report	01/01/2021	31/12/2021	Satisfactory completion of second progress report	31/01/2022
Compliance with working with children obligations	30/06/2021	30/06/2022	Working with Children Annual Statement of Compliance	30/07/2022
Third progress report	01/01/2022	31/12/2022	Satisfactory completion of third progress report	31/01/2023
End of project report	30/06/2020	29/06/2023	Satisfactory completion of end of project report	29/07/2023
Independent Audit report	30/06/2020	29/06/2023	Satisfactory report completed by independent auditor	29/07/2023
Compliance with working with children obligations	30/06/2022	29/06/2023	Working with Children Annual Statement of Compliance	30/07/2023

2639

2640

During the Agreement period, the Commonwealth may ask the Grantee for ad-hoc reports on the project. The Grantee must provide these reports in the timeframes notified by the Commonwealth.

The Grantee may also be asked to participate and provide information about the Activity as part of the overarching evaluation of the MRFF.

F. Party representatives and address for notices

Grantee's representative and address

Grantee's representative name	Ms Debbie Docherty
Position	Director, Research Grants and Contracts
Address	Anzac Pde<br>Kensington NSW 2052
Business hours telephone	+61 2 9385 7230
Mobile	
Email	rgc@unsw.edu.au

Commonwealth representative and address

Name of representative	Simon Williams
Position	
Postal address	GPO Box 2013<br>CANBERRA ACT 2601
Physical address	10 Binara Street<br>CANBERRA ACT 2600
Business hours telephone	03 9268 7551
Email	

The Parties' representatives will be responsible for liaison and the day-to-day management of the Grant, as well as accepting and issuing any written notices in relation to the Grant.

G. Activity Material

Not applicable

2641

Supplementary Terms

ST1. Other Contributions

Not applicable

ST2. Activity Budget

ST2.1 In this Agreement, Appropriation means money drawn from the Consolidated Revenue Fund.

ST2.2 The Grantee agrees to use the Grant and any Other Contributions and undertake the Activity consistently with the Activity Budget in the following table:

Financial year 2019/20

Head of expenditure	Breakdown of expenditure	Agreed project cost
Project expenditure	Contract	$0
Project expenditure	Equipment (up to $80,000 only)	$0
Project expenditure	Labour costs	$0
Project expenditure	Labour on-costs (Inclusive of additional 30% allowance)	$0
Project expenditure	Other eligible expenditure	$0
Project expenditure	Travel and Overseas Expenditure	$0
Financial year total		$0

Financial year 2020/21

Head of expenditure	Breakdown of expenditure	Agreed project cost
Project expenditure	Contract	$67,373
Project expenditure	Equipment (up to $80,000 only)	$0
Project expenditure	Labour costs	$1,123,110
Project expenditure	Labour on-costs (Inclusive of additional 30% allowance)	$293,181
Project expenditure	Other eligible expenditure	$110,170
Project expenditure	Travel and Overseas Expenditure	$17,936
Financial year total		$1,611,770

Financial year 2021/22

Head of expenditure	Breakdown of expenditure	Agreed project cost
Project expenditure	Contract	$81,373

Project expenditure	Equipment (up to $80,000 only)	$0
Project expenditure	Labour costs	$1,170,491
Project expenditure	Labour on-costs (Inclusive of additional 30% allowance)	$306,039
Project expenditure	Other eligible expenditure	$100,530
Project expenditure	Travel and Overseas Expenditure	$17,936
Financial year total		$1,676,369

Financial year 2022/23

Head of expenditure	Breakdown of expenditure	Agreed project cost
Project expenditure	Contract	$57,373
Project expenditure	Equipment (up to $80,000 only)	$0
Project expenditure	Labour costs	$1,216,253
Project expenditure	Labour on-costs (Inclusive of additional 30% allowance)	$315,203
Project expenditure	Other eligible expenditure	$100,530
Project expenditure	Travel and Overseas Expenditure	$17,936
Financial year total		$1,707,295

All financial years

Head of expenditure	Breakdown of expenditure	Agreed project cost
Project expenditure	Contract	$206,119
Project expenditure	Equipment (up to $80,000 only)	$0
Project expenditure	Labour costs	$3,509,854
Project expenditure	Labour on-costs (Inclusive of additional 30% allowance)	$914,423
Project expenditure	Other eligible expenditure	$311,230
Project expenditure	Travel and Overseas Expenditure	$53,808
All financial years total		$4,995,434

Figures in the above table are GST inclusive amounts less GST credits that can be claimed in relation to the expenditure.

ST2.3 Subject to sufficient appropriation being available, the Grant will be paid up to the Annual Capped Amounts over the financial years specified in the following table.

2646

Annual Capped Amounts

Financial Year	Annual capped amount (GST excl)
2019/20	$1,981,470.01
2020/21	$1,289,605.99
2021/22	$1,724,358
Total	$4,995,434

ST2.4 The Commonwealth is not required to make a payment if it would result in the amount paid in a financial year exceeding the Annual Capped Amount for that financial year specified in the table under clause ST2.3.

ST2.5 In accordance with the Activity Budget under clause ST2.2, the Annual Capped Amounts may not be exceeded unless the Commonwealth specifically approves an increase of that amount under clause ST2.8.

ST2.6 Subject to this clause, the Grantee may reallocate expenditure in respect of categories of expenditure in the Activity Budget, provided it does not materially change the Activity, any Milestone(s) set out in this Agreement, or cause the Grantee to be in breach of any of its obligations under this Agreement.

ST2.7 The Grantee must give the Commonwealth:

(a) at any time the Grantee wishes to request a variation to any one or more of the Annual Capped Amounts; or

(b) if otherwise requested by the Commonwealth,

a revised Activity Budget in a form acceptable to the Commonwealth. The revised Activity Budget must clearly identify any proposed changes, including of any proposed changes to the Annual Capped Amounts, and explain the reasons for the proposed changes.

ST2.8 The Commonwealth may, at its discretion, approve or reject a revised Activity Budget provided under clause ST2.7 and/or any proposed changes to the Annual Capped Amounts. The Commonwealth's approval may be granted subject to conditions.

ST2.9 If a revised Activity Budget and any proposed changes to the Annual Capped Amounts are approved by the Commonwealth, then it will become the Activity Budget and, if relevant, the Annual Capped Amounts will be adjusted accordingly.

ST3. Intellectual property in Activity Material

ST3.1 In this Agreement:

Application means the application form and all supporting documentation submitted by the Grantee in respect of which the Grant has been awarded.

National (IPPF Research) Principles means the *National Principles of Intellectual Property for Publicly Funded Research* (available at: http://www.arc.gov.au/policy) and any successor document.

ST3.2 The Commonwealth agrees not to claim ownership of any Intellectual Property Rights in an Application or in any research arising from the Activity.

ST3.3 The Grantee agrees to:

2647

(a) comply with an intellectual property policy which:

(i) is approved by the Grantee's governing body;

(ii) has as one of its aims, the maximisation of benefits to Australia arising from publicly funded research; and

(iii) complies with the National (IPPF Research) Principles;

(b) ensure that the Grantee and all applicants for participation in the Activity are familiar with the current intellectual property and patent landscape for the research areas included in the Application.

ST3.4 The Grantee provides the Commonwealth a permanent, non-exclusive, irrevocable, royalty-free licence (including a right to sub-licence) to use, modify, communicate, reproduce, publish and adapt the Activity Material.

ST4. Access/monitoring/inspection

ST4.1 The Grantee agrees to give the Commonwealth, or any persons authorised in writing by the Commonwealth:

(a) access to premises where the Activity is being performed and/or where Material relating to the Activity is kept within the time period specified in a Commonwealth notice; and

(b) permission to inspect and take copies of any Material relevant to the Activity.

ST4.2 The Auditor-General and any Information Officer under the *Australian Information Commissioner Act 2010* (Cth) (including their delegates) are persons authorised for the purposes of clause ST4.1.

ST4.3 This clause ST4 does not detract from the statutory powers of the Auditor-General or an Information Officer (including their delegates).

ST5. Equipment and Assets

Not applicable

ST6. Specified Personnel

Not applicable

ST7. Relevant qualifications, licences, permits, approvals or skills

Not applicable

ST8. Vulnerable Persons

ST8.1 In this Agreement

**Criminal or Court Record**	means any record of any Other Offence;

Other Offence means, in relation to a person, a conviction, finding of guilt, on-the-spot fine for, or court order relating to:

(a) an apprehended violence or protection order made against the person;

(b) the consumption, dealing in, possession or handling of alcohol, a prohibited drug, narcotic or other prohibited substance;

(c) violence against another person or the injury, but excluding the death, of another person; or

(d) an attempt to commit a crime or offence, or to engage in any conduct or activity, described in paragraphs (a) to (c);

Police Check means a formal inquiry made to the relevant police authority in each State or Territory and designed to obtain details of an individual's criminal conviction or a finding of guilt in all places (within and outside Australia) that the Grantee knows the person has resided in;

Serious Offence means:

(a) a crime or offence involving the death of a person;

(b) a sex-related offence or a crime, including sexual assault (whether against an adult or child); child pornography, or an indecent act involving a child;

(c) fraud, money laundering, insider dealing or any other financial offence or crime, including those under legislation relating to companies, banking, insurance or other financial services; or

(d) an attempt to commit a crime or offence described in (a) to (c);

Serious Record means a conviction or any finding of guilt regarding a Serious Offence; and

Vulnerable Person means an individual aged 18 years and above who is or may be unable to take care of themselves, or is unable to protect themselves against harm or exploitation for any reason, including age, physical or mental illness, trauma or disability, pregnancy, the influence, or past or existing use, of alcohol, drugs or substances or any other reason.

ST8.2 Before any person commences performing work on any part of the Activity that involves working or contact with a Vulnerable Person, the Grantee must:

(a) obtain a Police Check for that person;

(b) confirm that the person is not prohibited by any law from being engaged in a capacity where they may have contact with a Vulnerable Person;

(c) comply with all State, Territory or Commonwealth laws relating the employment or engagement of persons in any capacity where they may have contact with a Vulnerable Person; and

(d) ensure that the person holds all licences or permits for the capacity in which they are to be engaged, including any specified in the Grant Details, and the Grantee must

ensure that Police Checks and any licences or permits obtained in accordance with this clause ST8.2 remain current for the duration of their involvement in the Activity.

ST8.3 The Grantee must ensure that a person does not perform work on any part of the Activity that involves working or contact with a Vulnerable Person if a Police Check indicates that the person at any time has:

(a) a Serious Record; or

(b) a Criminal or Court Record and the Grantee has not conducted a risk assessment and determined that any risk is acceptable.

ST8.4 In undertaking a risk assessment under clause ST8.3, the Grantee must have regard to

(a) the nature and circumstances of the offence(s) on the person's Criminal or Court Record and whether the charge or conviction involved Vulnerable Persons;

(b) whether the person's Criminal or Court Record is directly relevant to, or reasonably likely to impair the person's ability to perform, the role that the person will, or is likely to, perform in relation to the Activity;

(c) the length of time that has passed since the person's charge or conviction and his or her record since that time;

(d) the circumstances in which the person will, or is likely to, have contact with a Vulnerable Person as part of the Activity;

(e) any other relevant matter,

and must ensure it fully documents the conduct and outcome of the risk assessment.

ST8.5 The Grantee agrees to notify the Commonwealth of any risk assessment it conducts under this clause and agrees to provide the Commonwealth with copies of any relevant documentation on request.

ST8.6 If during the term a person involved in performing work on any part of the Activity that involves working or contact with a Vulnerable Person is:

(a) charged with a Serious Offence or Other Offence, the Grantee must immediately notify the Commonwealth; or

(b) convicted of a Serious Offence, the Grantee must immediately notify the Commonwealth and ensure that that person does not, from the date of the conviction, perform any work or role relating to the Activity.

ST9. Child safety

ST9.1 In this Agreement

Child means an individual(s) under the age of 18 years and **Children** has a similar meaning;

Child-Related Personnel means officers, employees, contractors (including subcontractors), agents and volunteers of the Grantee involved with the Activity who as part of that involvement may interact with Children;

Legislation means a provision of a statute or subordinate legislation of the Commonwealth, or of a State, Territory or local authority;

**National Principles for Child Safe Organisations**	means the National Principles for Child Safe Organisations, which have been endorsed in draft form by the Commonwealth Government (available at: https://www.humanrights.gov.au/national-principles-child-safe-organisations) and subsequently, from the time of their endorsement by the Council of Australian Governments, the final National Principles for Child Safe Organisations as published by the Australian Government;
**Relevant Legislation**	means Legislation in force in any jurisdiction where any part of the Activity may be carried out;
**Working With Children Check or WWCC**	means the process in place pursuant to Relevant Legislation to screen an individual for fitness to work with Children.

Relevant checks and authority

ST9.2 The Grantee must

(a) comply with all Relevant Legislation relating to the employment or engagement of Child-Related Personnel in relation to the Activity, including all necessary Working With Children Checks however described; and

(b) ensure that Working With Children Checks obtained in accordance with this clause ST9.2 remain current and that all Child-Related Personnel continue to comply with all Relevant Legislation for the duration of their involvement in the Activity.

National Principles for Child Safe Organisations and other action for the safety of Children

ST9.3 The Grantee agrees in relation to the Activity to:

(a) implement the National Principles for Child Safe Organisations;

(b) ensure that all Child-Related Personnel implement the National Principles for Child Safe Organisations;

(c) complete and update, at least annually, a risk assessment to identify the level of responsibility for Children and the level of risk of harm or abuse to Children;

(d) put into place and update, at least annually, an appropriate risk management strategy to manage risks identified through the risk assessment required by this clause ST9.3;

(e) provide training and establish a compliance regime to ensure that all Child Related Personnel are aware of, and comply with

(i) the National Principles for Child Safe Organisations;

(ii) the Grantee's risk management strategy required by this clause ST9.3;

(iii) Relevant Legislation relating to requirements for working with Children, including Working With Children Checks;

(iv) Relevant Legislation relating to mandatory reporting of suspected child abuse or neglect, however described; and

(f) provide the Commonwealth with an annual statement of compliance with clauses ST9.2 and ST9.3, in such form as may be specified by the Commonwealth.

ST9.4 With reasonable notice to the Grantee, the Commonwealth may conduct a review of the Grantee's compliance with this clause ST9.

2656

ST9.5 The Grantee agrees to:

(a) notify the Commonwealth of any failure to comply with this clause ST9;

(b) co-operate with the Commonwealth in any review conducted by the Commonwealth of the Grantee's implementation of the National Principles for Child Safe Organisations or compliance with this clause ST9; and

(c) promptly, and at the Grantee's cost, take such action as is necessary to rectify, to the Commonwealth's satisfaction, any failure to implement the National Principles for Child Safe Organisations or any other failure to comply with this clause ST9.

ST10. Commonwealth Material, facilities and assistance

Not applicable

ST11. Jurisdiction

ST11.1 This Agreement is governed by the law of the Australian Capital Territory.

ST12. Grantee trustee of trust (if applicable)

ST12.1 In this Agreement, **Trust** means the trust specified in the Parties to the Agreement section of this Agreement.

ST12.2 The Grantee warrants that:

(a) it is the sole trustee of the Trust; and

(b) it has full and valid power and authority to enter into this Agreement and perform the obligations under it on behalf of the Trust; and

(c) it has entered into this Agreement for the proper administration of the Trust; and

(d) all necessary resolutions, consents, approvals and procedures have been obtained or duly satisfied to enter into this Agreement and perform the obligations under it; and

(e) it has the right to be indemnified out of the assets of the Trust for all liabilities incurred by it under this Agreement.

ST13. Fraud

ST13.1 In this Agreement, Fraud means dishonestly obtaining a benefit, or causing a loss, by deception or other means, and includes alleged, attempted, suspected or detected fraud.

ST13.2 The Grantee must ensure its personnel and subcontractors do not engage in any Fraud in relation to the Activity.

ST13.3 If the Grantee becomes aware of:

(a) any Fraud in relation to the performance of the Activity; or

(b) any other Fraud that has had or may have an effect on the performance of the Activity;

then it must within 5 business days report the matter to the Commonwealth and all appropriate law enforcement and regulatory agencies.

ST13.4 The Grantee must, at its own cost, investigate any Fraud referred to in clause ST11.3 in accordance with the Australian Government Investigations Standards available at www.ag.gov.au.

2657

2658

ST13.5 The Commonwealth may, at its discretion, investigate any Fraud in relation to the Activity. The Grantee agrees to co-operate and provide all reasonable assistance at its own cost with any such investigation.

ST13.6 This clause survives the termination or expiry of the Agreement.

ST14. Prohibited dealings

Not applicable

ST15. Anti-corruption

ST15.1 In this Agreement:

Illegal or Corrupt Practice means directly or indirectly:

(a) making or causing to be made, any offer, gift, payment, consideration or benefit of any kind to any party, or

(b) receiving or seeking to receive, any offer, gift, payment, consideration or benefit of any kind from any party, as an inducement or reward in relation to the performance of the Activity, which would or could be construed as an illegal or corrupt practice;

ST15.2 The Grantee warrants that the Grantee, its officers, employees, contractors, agents and any other individual or entity involved in carrying out the Activity have not, engaged in an Illegal or Corrupt Practice.

ST15.3 The Grantee agrees not to, and to take all reasonable steps to ensure that its officers, employees, contractors, agents and any other individual or entity involved in carrying out the Activity do not:

(a) engage in an Illegal or Corrupt Practice; or

(b) engage in any practice that could constitute the offence of bribing a foreign public official contained in section 70.2 of the Criminal Code Act 1995 (Cth).

ST15.4 The Grantee agrees to inform the Commonwealth within five business days if the Grantee becomes aware of any activity as described in ST15.3 in relation to the performance of the Activity.

ST16. Step-in rights

Not applicable

ST17. Grant administrator

Not applicable

ST18. Management Adviser

Not applicable

ST19. Indemnities

ST19.1 The Grantee indemnifies the Commonwealth, its officers, employees and contractors against any claim, loss or damage arising in connection with the Activity.

ST19.2 The Grantee's obligation to indemnify the Commonwealth will reduce proportionally to the extent any act or omission involving fault on the part of the Commonwealth contributed to the claim, loss or damage.

2659

ST20. Compliance with Legislation and Policies

ST20.1 In this Agreement:

Legislation means a provision of a statute or subordinate legislation of the Commonwealth, or of a State, Territory or local authority.

ST20.2 The Grantee agrees to comply with all Legislation applicable to its performance of this Agreement.

ST20.3 The Grantee agrees, in carrying out its obligations under this Agreement, to comply with any of the Commonwealth's policies as notified, referred or made available by the Commonwealth to the Grantee (including by reference to an internet site).

ST20.4 In carrying out the Activity, the Grantee must comply with the following applicable policies/laws:

(a) *Medical Research Future Fund Act 2015*

(b) Ethics and research practices:

(i) NHMRC/ARC/UA Australian Code for the Responsible Conduct of Research (2018)

(ii) NHMRC/ARC/UA National Statement on Ethical Conduct in Human Research (2007, updated 2018)

(iii) Ethical Conduct in Research with Aboriginal and Torres Strait Islander Peoples and communities (2018)

ST21. Work health and safety

ST21.1 The Grantee agrees to ensure that it complies at all times with all applicable work health and safety legislative and regulatory requirements and any additional work health and safety requirements set out in the Grant Details.

ST21.2 If requested by the Commonwealth, the Grantee agrees to provide copies of its work health and safety management plans and processes and such other details of the arrangements it has in place to meet the requirements referred to in clause ST21.1.

ST21.3 When using the Commonwealth's premises or facilities, the Grantee agrees to comply with all reasonable directions and procedures relating to work health and safety and security in effect at those premises or those facilities, as notified by the Commonwealth or as might reasonably be inferred from the use to which the premises or facilities are being put.

ST22. Transition

Not applicable

ST23. Corporate Governance

ST23.1 In this Agreement:

Constitution means (depending on the context):

(a) a company's, body corporate's or incorporated association's constitution, or equivalent documents, which (where relevant) includes rules and any amendments that are part of the constitution;

(b) in relation to any other kind of body:

y

(i) the body's charter or memorandum; or

(ii) any instrument or law constituting or defining the constitution of the body or governing the activities of the body or its members.

ST23.2 The Grantee warrants that nothing in its constitution conflicts with its obligations under this Agreement.

ST23.3 The Grantee agrees to provide a copy of its constitution to the Commonwealth upon request and inform the Commonwealth whenever there is a change in the Grantee's constitution, structure or management.

ST24. Counterparts

ST24.1 This Agreement may be executed in any number of counterparts. All counterparts, taken together, constitute one instrument. A Party may execute this Agreement by signing any counterpart.

ST25. Secret and Sacred Indigenous Material

Not applicable

Schedule 1: Commonwealth Standard Grant Conditions

1. Undertaking the Activity

1.1 The Grantee agrees to undertake the Activity for the purpose of the Grant in accordance with this Agreement.

1.2 The Grantee is fully responsible for the Activity and for ensuring the performance of all its obligations under this Agreement in accordance with all relevant laws. The Grantee will not be relieved of that responsibility because of:

(a) the grant or withholding of any approval or the exercise or non-exercise of any right by the Commonwealth; or

(b) any payment to, or withholding of any payment from, the Grantee under this Agreement.

2. Payment of the Grant

2.1 The Commonwealth agrees to pay the Grant to the Grantee in accordance with the Grant Details.

2.2 Notwithstanding any other provision of this Agreement, the Commonwealth may by notice withhold payment of any amount of the Grant and/or take any other action specified in the Supplementary Terms if it reasonably believes that:

(a) the Grantee has not complied with this Agreement;

(b) the Grantee is unlikely to be able to perform the Activity or manage the Grant in accordance with this Agreement; or

(c) there is a serious concern relating to this Agreement that requires investigation.

2.3 A notice under clause 2.2 will contain the reasons any action taken under clause 2.2 and, where relevant, the steps the Grantee can take to address those reasons.

2.4 The Commonwealth will only be obliged to pay the withheld amount once the Grantee has addressed the reasons contained in a notice under clause 2.2 to the Commonwealth's reasonable satisfaction.

3. Acknowledgements

3.1 The Grantee agrees not to make any public announcement, including by social media, in connection with the awarding of the Grant without the Commonwealth's prior written approval.

3.2 The Grantee agrees to acknowledge the Commonwealth's support in all Material, publications and promotional and advertising materials published in connection with this Agreement. The Commonwealth may notify the Grantee of the form of acknowledgement that the Grantee is to use.

4. Notices

4.1 Each Party agrees to promptly notify the other Party of anything reasonably likely to adversely affect the undertaking of the Activity, management of the Grant or its performance of any of its other requirements under this Agreement.

4.2 A notice given by a Party under this Agreement must be in writing and addressed to the other Party's representative as set out in the Grant Details or as most recently updated by notice given in accordance with this clause.

5. Relationship between the Parties

5.1 A Party is not by virtue of this Agreement the employee, agent or partner of the other Party and is not authorised to bind or represent the other Party.

6. Subcontracting

6.1 The Grantee is responsible for the performance of its obligations under this Agreement, including in relation to any tasks undertaken by subcontractors.

6.2 The Grantee agrees to make available to the Commonwealth the details of any of its subcontractors engaged to perform any tasks in relation to this Agreement upon request.

7. Conflict of interest

7.1 Other than those which have already been disclosed to the Commonwealth, the Grantee warrants that, to the best of its knowledge, at the date of this Agreement, neither it nor its officers have any actual, perceived or potential conflicts of interest in relation to the Activity.

7.2 If during the term of the Agreement, any actual, perceived or potential conflict arises or there is any material change to a previously disclosed conflict of interest, the Grantee agrees to:

(a) notify the Commonwealth promptly and make full disclosure of all relevant information relating to the conflict; and

(b) take any steps the Commonwealth reasonably requires to resolve or otherwise deal with that conflict.

8. Variation, assignment and waiver

8.1 This Agreement may be varied in writing only, signed by both Parties.

8.2 The Grantee cannot assign its obligations, and agrees not to assign its rights, under this Agreement without the Commonwealth's prior approval.

8.3 The Grantee agrees not to enter into negotiations with any other person for the purposes of entering into an arrangement that will require novation of, or involve any assignment of rights under, this Agreement without first consulting the Commonwealth.

8.4 A waiver by a Party of any of its rights under this Agreement is only effective if it is in a signed written notice to the other Party and then only to the extent specified in that notice.

9. Taxes, duties and government charges

9.1 The Grantee agrees to pay all taxes, duties and government charges imposed or levied in Australia or overseas in connection with the performance of this Agreement, except as provided by this Agreement.

9.2 If Goods and Services Tax (GST) is payable by a supplier on any supply made under this Agreement, the recipient of the supply will pay to the supplier an amount equal to the GST payable on the supply, in addition to and at the same time that the consideration for the supply is to be provided under this Agreement.

9.3 The Parties acknowledge and agree that they each:

(a) are registered for GST purposes;

(b) have quoted their Australian Business Number to the other; and

(c) must notify the other of any changes to the matters covered by this clause.

9.4 The Grantee agrees that the Commonwealth will issue it with a recipient created tax invoices for any taxable supplies it makes under this Agreement.

9.5 The Grantee agrees not to issue tax invoices in respect of any taxable supplies.

9.6 If the Grantee is not, or not required to be, registered for GST, then:

(a) clauses 9.3(a), 9.4 and 9.5 do not apply; and

(b) the Grantee agrees to notify the Commonwealth in writing within 7 days of becoming registered for GST if during the term of the Agreement it becomes, or is required to become, registered for GST.

10. Spending the Grant

10.1 The Grantee agrees to spend the Grant for the purpose of performing the Activity and otherwise in accordance with this Agreement.

10.2 Within 90 days after the Activity Completion Date, the Grantee agrees to provide the Commonwealth with an independently audited financial acquittal report verifying that the Grant has been spent in accordance with this Agreement.

10.3 The reports under clause 10.2 must be audited by:

(a) a Registered Company Auditor registered under the *Corporations Act 2001* (Cth); or

(b) a certified Practising Accountant; or

(c) a member of the Institute of Public Accountants; or

(d) a member of Chartered Accountants Australia and New Zealand;

who is not a principal member, shareholder, officer or employee of the Grantee or a related body corporate.

11. Repayment

11.1 If any amount of the Grant:

(a) has been spent other than in accordance with this Agreement; or

(b) is additional to the requirements of the Activity

then the Commonwealth may, by written notice:

(c) require the Grantee to repay that amount to the Commonwealth;

(d) require the Grantee to deal with that amount as directed by the Commonwealth; or

(e) deduct the amount from subsequent payments of the Grant or amounts payable under another agreement between the Grantee and the Commonwealth.

11.2 If the Commonwealth issues a notice under this Agreement requiring the Grantee to repay a Grant amount:

(a) the Grantee must do so within the time period specified in the notice;

(b) the Grantee must pay interest on any part of the amount that is outstanding at the end of the time period specified in the notice until the outstanding amount is repaid in full; and

(c) the Commonwealth may recover the amount and any interest under this Agreement as a debt due to the Commonwealth without further proof of the debt being required.

12. Record keeping

12.1 The Grantee agrees to keep financial accounts and other records that:

(a) detail and document the conduct and management of the Activity;

(b) identify the receipt and expenditure of the Grant and any Other Contributions separately within the Grantee's accounts and records so that at all times the Grant is identifiable;

(c) enable all receipts and payments related to the Activity to be identified and reported.

12.2 The Grantee agrees to keep the records for five years after the Activity Completion Date or such other time specified in the Grant Details and provide copies of the records to the Commonwealth upon request.

13. Reporting and liaison

13.1 The Grantee agrees to provide the Reporting Material specified in the Grant Details to the Commonwealth.

13.2 In addition to the obligations in clause 13.1, the Grantee agrees to:

(a) liaise with and provide information to the Commonwealth as reasonably required by the Commonwealth; and

(b) comply with the Commonwealth's reasonable requests, directions, or monitoring requirements,

in relation to the Activity.

13.3 If the Commonwealth acting reasonably has concerns regarding the performance of the Activity or the management of the Grant, the Commonwealth may by written notice require the Grantee to provide one or more additional reports, containing the information and by the date(s) specified in the notice.

13.4 The Grantee acknowledges that the giving of false or misleading information to the Commonwealth is a serious offence under the *Criminal Code Act 1995* (Cth).

14. Privacy

14.1 When dealing with Personal Information in carrying out the Activity, the Grantee agrees:

(a) to comply with the requirements of the *Privacy Act 1988* (Cth);

(b) not to do anything which, if done by the Commonwealth, would be a breach of an Australian Privacy Principle;

(c) to ensure that any of the Grantee's subcontractors or personnel who deal with Personal Information for the purposes of this Agreement are aware of the requirements of the *Privacy Act 1988* (Cth) and the Grantee's obligations under this clause;

2716

(d) to immediately notify the Commonwealth if the Grantee becomes aware of an actual or possible breach of this clause by the Grantee or any of the Grantee's subcontractors or personnel.

14.2 In carrying out the Activity, the Grantee agrees not to send any Personal Information outside of Australia without the Commonwealth's prior written approval. The Commonwealth may impose any conditions it considers appropriate when giving its approval.

15. Confidentiality

15.1 The Parties agree not to disclose each other's confidential information without the other Party's prior written consent unless required or authorised by law or Parliament to disclose.

15.2 The Commonwealth may disclose the Grantee's confidential information where;

(a) the Commonwealth is providing information about the Activity or Grant in accordance with Commonwealth accountability and reporting requirements;

(b) the Commonwealth is disclosing the information to a Minister of the Australian Government, a House or Committee of the Commonwealth Parliament; or

(c) the Commonwealth is disclosing the information to its personnel or another Commonwealth agency where this serves the Commonwealth's legitimate interests.

16. Insurance

16.1 The Grantee agrees to maintain adequate insurance for as long as any obligations remain in connection with this Agreement and provide proof of insurance to the Commonwealth upon request.

17. Intellectual property

17.1 Subject to clause 17.2 the Grantee owns the Intellectual Property Rights in Activity Material created and Reporting Material.

17.2 This Agreement does not affect the ownership of Intellectual Property Rights in Existing Material.

17.3 The Grantee provides the Commonwealth a permanent, non-exclusive, irrevocable, royalty-free licence to use, modify, communicate, reproduce, publish, adapt and sub-license the Reporting Material for Commonwealth Purposes.

17.4 The licence in clause 17.3 does not apply to Activity Material.

18. Dispute resolution

18.1 The Parties agree not to initiate legal proceedings in relation to a dispute arising under this Agreement unless they have first tried and failed to resolve the dispute by negotiation.

18.2 Unless clause 18.3 applies, the Parties agree to continue to perform their respective obligations under this Agreement when a dispute exists.

18.3 The Parties may agree to suspend performance of the Agreement pending resolution of the dispute.

18.4 Failing settlement by negotiation in accordance with clause 18.1, the Parties may agree to refer the dispute to an independent third person with power to intervene and direct some form of

2717

resolution, in which case the Parties will be bound by that resolution. If the Parties do not agree to refer the dispute to an independent third person, either Party may initiate legal proceedings.

18.5 Each Party will bear their own costs in complying with this clause 18, and the Parties will share equally the cost of any third person engaged under clause 18.4.

18.6 The procedure for dispute resolution under this clause does not apply to any action relating to termination, cancellation or urgent interlocutory relief.

19. Reduction, Suspension and Termination

19.1 Reduction in scope of agreement for fault

19.1.1 If the Grantee does not comply with an obligation under this Agreement and the Commonwealth believes that the non-compliance is incapable of remedy, or if the Grantee has failed to comply with a notice to remedy, the Commonwealth may by written notice reduce the scope of the Agreement.

19.1.2 The Grantee agrees, on receipt of the notice of reduction, to:

(a) stop or reduce the performance of the Grantee's obligations as specified in the notice;

(b) take all available steps to minimise loss resulting from the reduction;

(c) continue performing any part of the Activity or the Agreement not affected by the notice if requested to do so by the Commonwealth;

(d) report on, and return any part of the Grant to the Commonwealth, or otherwise deal with the Grant, as directed by the Commonwealth.

19.1.3 In the event of reduction under clause 19.1.1, the amount of the Grant will be reduced in proportion to the reduction in the scope of the Agreement.

19.2 Suspension

19.2.1 If:

(a) the Grantee does not comply with an obligation under this Agreement and the Commonwealth believes that the non-compliance is capable of remedy;

(b) the Commonwealth reasonably believes that the Grantee is unlikely to be able to perform the Activity or manage the Grant in accordance with this Agreement; or

(c) the Commonwealth reasonably believes that there is a serious concern relating to this Agreement that requires investigation;

the Commonwealth may by written notice:

(d) immediately suspend the Grantee from further performance of the Agreement (including expenditure of the Grant); and/or

(e) require that the non-compliance or inability be remedied, or the investigation be completed, within the time specified in the notice.

19.2.2 If the Grantee:

(a) remedies the non-compliance or inability specified in the notice to the Commonwealth's reasonable satisfaction, or the Commonwealth reasonably concludes that the concern is unsubstantiated, the Commonwealth may direct the Grantee to recommence performing the Activity; or

(b) fails to remedy the non-compliance or inability within the time specified, or the Commonwealth reasonably concludes that the concern is likely to be substantiated, the Commonwealth may reduce the scope of the Agreement in accordance with clause 19.1 or terminate the Agreement immediately by giving a second notice in accordance with clause 19.3.

19.3 Termination for fault

19.3.1 The Commonwealth may terminate this Agreement by notice where the Grantee has:

(a) failed to comply with an obligation under this Agreement and the Commonwealth believes that the non-compliance is incapable of remedy or where clause 19.2.2(b) applies;

(b) provided false or misleading statements in relation to the Grant; or

(c) become bankrupt or insolvent, entered into a scheme of arrangement with creditors, or come under any form of external administration.

19.3.2 The Grantee agrees, on receipt of the notice of termination, to:

(a) stop the performance of the Grantee's obligations;

(b) take all available steps to minimise loss resulting from the termination; and

(c) report on, and return any part of the Grant to the Commonwealth, or otherwise deal with the Grant, as directed by the Commonwealth.

20. Cancellation or reduction for convenience

20.1 The Commonwealth may cancel or reduce the scope of this Agreement by notice, due to:

(a) a change in government policy; or

(b) a Change in the Control of the Grantee which the Commonwealth reasonably believes will negatively affect the Grantee's ability to comply with this Agreement.

20.2 On receipt of a notice of reduction or cancellation under this clause, the Grantee agrees to:

(a) stop or reduce the performance of the Grantee's obligations as specified in the notice; and

(b) take all available steps to minimise loss resulting from that reduction or cancellation; and

(c) continue performing any part of the Activity or the Agreement not affected by the notice if requested to do so by the Commonwealth;

(d) report on, and return any part of the Grant to the Commonwealth, or otherwise deal with the Grant, as directed by the Commonwealth.

20.3 In the event of reduction or cancellation under this clause, the Commonwealth will be liable only to:

(a) pay any part of the Grant due and owing to the Grantee under this Agreement at the date of the notice; and

(b) reimburse any reasonable and substantiated expenses the Grantee unavoidably incurs that relate directly and entirely to the reduction in scope or cancellation of the Agreement.

20.4 In the event of reduction, the amount of the Grant will be reduced in proportion to the reduction in the scope of the Agreement.

20.5 The Commonwealth's liability to pay any amount under this clause is:

(a) subject to the Grantee's compliance with this Agreement; and

(b) limited to an amount that when added to all other amounts already paid under the Agreement will not exceed the total amount of the Grant.

20.6 The Grantee will not be entitled to compensation for loss of prospective profits or benefits that would have been conferred on the Grantee but for the cancellation or reduction in scope of the Agreement under clause 20.1.

20.7 The Commonwealth will act reasonably in exercising its rights under this clause.

21. Survival

21.1 The following clauses survive termination, cancellation or expiry of this Agreement:

- clause 10 (Spending the Grant);
- clause 11 (Repayment);
- clause 12 (Record keeping);
- clause 13 (Reporting);
- clause 14 (Privacy);
- clause 15 (Confidentiality);
- clause 16 (Insurance)
- clause 17 (Intellectual property);
- clause 19 (Reduction, Suspension and Termination);
- clause 21 (Survival);
- clause 22 (Definitions);
- ST3 (Intellectual property in Activity Material)
- ST4 (Access/monitoring/inspection);
- ST19 (Indemnities);and
- any other clause which expressly or by implication from its nature is meant to survive.

22. Definitions

22.1 In this Agreement, unless the contrary appears:

- **Activity** means the activities described in the Grant Details and includes the provisions of the Reporting Material.
- **Activity Completion Date** means the date or event specified in the Grant Details.
- **Activity Material** means any Material, other than Reporting Material, created or developed by the Grantee as a result of the Activity and includes any Existing Material that is incorporated in or supplied with the Activity Material.
- **Agreement** means the Grant Details, Supplementary Terms (if any), the Commonwealth Standard Grant Conditions and any other document referenced or incorporated in the Grant Details.
- **Agreement End Date** means the date or event specified in the Grant Details.

2724

- **Australian Privacy Principle** has the same meaning as in the *Privacy Act 1988*
- **Change in the Control** means any change in any person(s) who directly exercise effective control over the Grantee.
- **Commonwealth** means the Commonwealth of Australia as represented by the Commonwealth entity specified in the Agreement and includes, where relevant, its officers, employees, contractors and agents.
- **Commonwealth Purposes** includes the following:

  (a) the Commonwealth verifying and assessing grant proposals, including a grant application;

  (b) the Commonwealth administering, monitoring, reporting on, auditing, publicising and evaluating a grant program or exercising its rights under this Agreement;

  (c) the Commonwealth preparing, managing, reporting on, auditing and evaluating agreements, including this Agreement; and

  (d) the Commonwealth developing and publishing policies, programs, guidelines and reports, including Commonwealth annual reports;

  but in all cases:

  (e) excludes the commercialisation (being for-profit use) of the Material by the Commonwealth.
- **Commonwealth Standard Grant Conditions** means this document.
- **Existing Material** means Material developed independently of this Agreement that is incorporated in or supplied as part of Reporting Material or Activity Material.
- **Grant** means the money, or any part of it, payable by the Commonwealth to the Grantee for the Activity as specified in the Grant Details and includes any interest earned by the Grantee on that money once the Grant has been paid to the Grantee.
- **Grantee** means the legal entity other than the Commonwealth specified in the Agreement and includes, where relevant, its officers, employees, contractors and agents.
- **Grant Details** means the document titled Grant Details that forms part of this Agreement.
- **Intellectual Property Rights** means all copyright, patents, registered and unregistered trademarks (including service marks), registered designs, and other rights resulting from intellectual activity (other than moral rights under the *Copyright Act 1968*).
- **Material** includes documents, equipment, software (including source code and object code versions), goods, information and data stored by any means including all copies and extracts of them.
- **Party** means the Grantee or the Commonwealth.
- **Personal Information** has the same meaning as in the *Privacy Act 1988*.
- **Records** includes documents, information and data stored by any means and all copies and extracts of the same.
- **Reporting Material** means all Material which the Grantee is required to provide to the Commonwealth for reporting purposes as specified in the Grant Details, and includes any Existing Material that is incorporated in or supplied with the Reporting Material.

2725

Signatures

Executed as an agreement:

Commonwealth

Signed for and on behalf of the Commonwealth of Australia as represented by the Department of Health.

Name (print)	Masha Somi
Position (print)	Chief Executive Officer, Health and Medical Research Office
Signature	Digitally signed by Masha Somi Date: 2020.06.23 22:11:50 +10'00'
Date	
Witness name (print)	Digitally signed by Mikayla Burke Date: 2020.06.25 14:52:35 +10'00'
Signature	
Date	

Grantee

Full legal name of the Grantee	UNIVERSITY OF NEW SOUTH WALES<br>ABN: 57195873179
Name (print)	Debbie Docherty
Signature	Debbie Docherty Digitally signed by Debbie Docherty Date: 2020.06.19 12:22:28 +10'00'
Date	19 Jun 2020
Witness name (print)	Thomas Thong
Signature	
Date	19 Jun 2020

MRFF Applied Artificial Intelligence Research in Health Standard Grant Agreement February 2020 Page 33 of 41

2728

Schedule 2 Reporting requirements

Appendix 1

2019 Applied Artificial Intelligence Research in Health Grant Opportunity - progress report requirements

You will need to provide the following information in your progress reports. The Commonwealth reserves the right to amend or adjust the requirements.

You must complete and submit your report on the portal. You can enter the required information in stages and submit when it is complete.

Project progress

a. Complete the following table, updating for all milestones shown in the Activity Schedule of your grant agreement.

Milestone	Agreed end date	Actual/ anticipated end date	Current % complete	Progress comments – Include progress towards completion of agreed project activites including risks arising, how they are being managed to ensure planned project outcomes are met and any delays in achieving Milestones

b. Where applicable, describe any project activities completed during the reporting period that are not captured in the table above.

c. Is the overall project proceeding in line with your grant agreement?

If no, identify any changes or anticipated issues. Comment on any impacts on project timing and outcomes and how you expect to manage these.

d. Are there any planned events relating to the project that you are required to notify us about in accordance with your agreement?

If yes, provide details of the event including date, time, purpose of the event and key stakeholders expected to attend.

e. Have you complied with all legislation applicable to the delivery of the activity as outlined in ST20 of this Agreement. If not, please provide an explanation why.

2729

Project outcomes

a. Outline the extent to which you have met the project outcomes to date, as per the objectives in Section 1.3 of the Grant Opportunity Guidelines.

b. Outline the extent to which you have met the project objectives to date, as per Section 1.3 of the Grant Opportunity Guidelines

c. Are you on track to deliver these project outcomes and objectives?

d. Provide information about your progress against your project milestones:

- Describe the extent to which your progress supports your achievement of key project activities.

e. What is the most important finding from your research to date?

- Have you found new and/or unexpected findings or outcomes through the process?

f. What is your strategy for disseminating that knowledge and supporting its path to full implementation?

- Have you identified enablers and/or barriers to the translation/implementation of your research findings?

Project expenditure

Provide the following information about your eligible project expenditure. Eligible expenditure is divided into the same categories as the budget in your application.

All expenditure should be GST inclusive, less GST credits you can claim. We may ask you to provide evidence of costs incurred.

Refer to the grant opportunity guidelines or contact us if you have any questions about eligible expenditure.

a. What is the eligible expenditure you have incurred in this reporting period?

b. What is the estimated eligible expenditure for the next reporting period?

c. What is the estimated eligible expenditure for remaining reporting periods in current financial year (if applicable)?

d. What is the estimated total eligible expenditure for future financial years?

e. What is the estimated total eligible expenditure for the project?

f. Briefly explain the reason for any changes between the forecast and actual expenditure for the current reporting period, and any significant changes to the forecast budget for the remainder of the project.

g. Is the project expenditure broadly in line with the activity budget in the grant agreement?

If no, explain the reasons.

Attachments

a. Attach any agreed evidence required with this report to demonstrate project progress.

b. Attach copies of any published reports and promotional material, relating to the project.

2732

Certification

You must ensure an authorised person completes the report and can certify the following:

- The information in this report is accurate, complete and not misleading and that I understand the giving of false or misleading information is a serious offence under the *Criminal Code 1995* (Cth).
- The activities undertaken and the expenditure incurred is in accordance with the grant agreement.
- I am aware of the grantee's obligations under their grant agreement.
- I am aware that the grant agreement empowers the Commonwealth to terminate the grant agreement and to request repayment of funds paid to the grantee where the grantee is in breach of the grant agreement.

2733

2734

Appendix 2

2019 Applied Artificial Intelligence Research in Health Grant Opportunity - end of project report requirements

You will need to provide the following information in your progress reports. The Commonwealth reserves the right to amend or adjust the requirements.

You must complete and submit your report on the portal. You can enter the required information in stages and submit when it is complete.

Project achievements

a. Complete the following table, updating for all milestones shown in the Activity Schedule of your grant agreement.

Milestone	Agreed end date	Actual/ anticipated end date	Current % complete	Outcome comments – work undertaken and impact of any delay

b. Where applicable, describe any project activities completed during the reporting period that are not captured in the table above.

Project outcomes

a. Outline the extent to which you have met the project objectives at the project end date as per the objectives in Section 1.3 of the Grant Opportunity Guidelines.

b. Outline the extent to which you have met the project outcomes at the project end date as per outcomes in Section 1.3 of the Grant Opportunity Guidelines.

c. Did you deliver all your project milestones?

If you have not fully met the project objectives and outcomes, explain why.

d. Are there any planned events relating to the project that you are required to notify us about in accordance with your agreement?

If yes, provide details of the event including date, time, purpose of the event and key stakeholders expected to attend.

e. What is the most important finding from your research at project end date?

- Have you found new and/or unexpected findings and outcomes through the process?

f. Describe the extent to which your strategy for disseminating knowledge gained from the project has supported/is supporting implementation of research findings.

2735

- Have you identified enablers and/or barriers to the translation/implementation of your research findings?

g. Reflecting on the overall project, what are the two or three key learnings that could inform the design and conduct of similar research projects in the future?

Project benefits

a. What benefits has the project achieved?

b. What ongoing impact will the project have?

c. Did the project result in any unexpected benefits?

If yes, explain why.

d. Is there any other information you wish to provide about your project?

If yes, provide details.

Total eligible project expenditure

a. Indicate the total eligible project expenditure incurred. Eligible expenditure is divided into the same categories as the budget in your application.

All expenditure should be GST inclusive, less GST credits you can claim. We may ask you to provide evidence of costs incurred.

Refer to the grant opportunity guidelines or contact us if you have any questions about eligible expenditure.

b. Provide any comments you may have to clarify any figures.

c. Was the expenditure incurred in accordance with the activity budget in the grant agreement?

If no, explain the reason for a project underspend or overspend, or any other significant changes to the budget.

Updated business indicators

a. Provide the following financial data for your organisation for your latest complete financial year.

These fields are mandatory and entering $0 is acceptable if applicable.

- Financial year completed
- Sales revenue (turnover)
- Export revenue
- R&D expenditure
- Taxable income
- Number of employees including working proprietors and salaried directors (headcount)
- Number of independent contractors (headcount)

Attachments

a. Attach any agreed evidence required with this report to demonstrate progress or successful completion of your project.

b. Attach copies of any published reports and promotional material, relating to the project.

Certification

You must ensure an authorised person completes the report and can certify the following:

- The information in this report is accurate, complete and not misleading and that I understand the giving of false or misleading information is a serious offence under the *Criminal Code 1995* (Cth).
- The activities undertaken and the expenditure incurred is in accordance with the grant agreement.
- I am aware of the grantee's obligations under their grant agreement.
- I am aware that the grant agreement empowers the Commonwealth to terminate the grant agreement and to request repayment of funds paid to the grantee where the grantee is in breach of the grant agreement.

MRFF Applied Artificial Intelligence Research in Health Standard Grant Agreement February 2020 Page 39 of 41

Appendix 3

2019 Applied Artificial Intelligence Research in Health Grant Opportunity -
Compliance with working with children obligations

Where applicable, you will need to answer the following questions in your annual statement of compliance. The Commonwealth reserves the right to amend or adjust the requirements.

You must submit your annual statement of compliance as you would a report on the portal.

Statement of compliance

1 Is the organisation, and persons working with children on behalf of the organisation in relation to the Activity, compliant with Commonwealth, state or territory legislation?

2 Has the organisation completed a risk assessment in relation to the Activity and all persons who may engage with children in association with the Activity?

3 Has the organisation put in place an appropriate strategy to manage risks identified through the risk assessment?

4 Has the organisation delivered training and established a compliance regime to ensure that all persons who may engage with children are aware of, and comply with:

- the National Principles for Child Safe Organisations
- the risk management strategy in item 3 above
- relevant legislation relating to requirements for working with children, including working with children checks
- relevant legislation relating to requirements for working with vulnerable people, including working with vulnerable people checks; and
- relevant legislation relating to mandatory reporting of suspected child abuse or neglect however described?

Certification

You must ensure an authorised person completes the report and can certify the following:

- The information in this report is accurate, complete and not misleading and that I understand the giving of false or misleading information is a serious offence under the *Criminal Code 1995* (Cth).
- The activities undertaken and the expenditure incurred is in accordance with the grant agreement.
- I am aware of the grantee's obligations under their grant agreement.
- I am aware that the grant agreement empowers the Commonwealth to terminate the grant agreement and to request repayment of funds paid to the grantee where the grantee is in breach of the grant agreement.

Appendix 4

Independent audit report

A template and related guidance for this report will be available on business.gov.au and GrantsConnect.

References (ICP GCP 6.2.7)

1. Cavanagh, K., et al., *Can mindfulness and acceptance be learnt by self-help?: A systematic review and meta-analysis of mindfulness and acceptance-based self-help interventions.* Clinical Psychology Review, 2014. **34**(2): p. 118-129.
2. Tang, Y.-Y., B.K. Hölzel, and M.I. Posner, *The neuroscience of mindfulness meditation.* Nature Reviews Neuroscience, 2015. **16**(4): p. 213-225.
3. Sevinc, G., et al., *Common and Dissociable Neural Activity After Mindfulness-Based Stress Reduction and Relaxation Response Programs.* Psychosom Med, 2018. **80**(5): p. 439-451.
4. Dawson, A.F., et al., *Mindfulness-Based Interventions for University Students: A Systematic Review and Meta-Analysis of Randomised Controlled Trials.* Appl Psychol Health Well Being, 2019. **n/a**(n/a).
5. Bamber, M.D. and E. Morpeth, *Effects of Mindfulness Meditation on College Student Anxiety: a Meta-Analysis.* Mindfulness, 2019. **10**(2): p. 203-214.
6. Blanck, P., et al., *Effects of mindfulness exercises as stand-alone intervention on symptoms of anxiety and depression: Systematic review and meta-analysis.* Behav Res Ther, 2018. **102**: p. 25-35.
7. Lyzwinski, L.N., et al., *A Systematic Review of Electronic Mindfulness-Based Therapeutic Interventions for Weight, Weight-Related Behaviors, and Psychological Stress.* Telemedicine and e-Health, 2017. **24**(3): p. 173-184.
8. Huberty, J., et al., *Efficacy of the Mindfulness Meditation Mobile App "Calm" to Reduce Stress Among College Students: Randomized Controlled Trial.* JMIR Mhealth Uhealth, 2019. **7**(6): p. e14273.
9. Rebar, A.L., et al., *A meta-meta-analysis of the effect of physical activity on depression and anxiety in non-clinical adult populations.* Health Psychology Review, 2015. **9**(3): p. 366-378.
10. Larun, L., et al., *Exercise in prevention and treatment of anxiety and depression among children and young people.* Cochrane Database Syst Rev, 2006(3): p. Cd004691.
11. San Román-Mata, S., et al., *Benefits of Physical Activity and Its Associations with Resilience, Emotional Intelligence, and Psychological Distress in University Students from Southern Spain.* Int J Environ Res Public Health, 2020. **17**(12).
12. Chan, J.S.Y., et al., *Special Issue – Therapeutic Benefits of Physical Activity for Mood: A Systematic Review on the Effects of Exercise Intensity, Duration, and Modality.* The Journal of Psychology, 2019. **153**(1): p. 102-125.
13. Cebolla, A., et al., *Unwanted effects: Is there a negative side of meditation? A multicentre survey.* PLOS ONE, 2017. **12**(9): p. e0183137.
14. Klika, B. and C. Jordan, *HIGH-INTENSITY CIRCUIT TRAINING USING BODY WEIGHT: Maximum Results With Minimal Investment.* ACSM's Health & Fitness Journal, 2013. **17**(3).
15. Schmidt, D., et al., *The effect of high-intensity circuit training on physical fitness.* The Journal of sports medicine and physical fitness, 2015. **56**.
16. Engel, F., et al., *Classroom-based micro-sessions of functional high-intensity circuit training enhances functional strength but not cardiorespiratory fitness in school children–a feasibility study.* Frontiers in Public Health, 2019. **7**: p. 291.
17. Miller, M.B., et al., *The Effect of a Short-Term High-Intensity Circuit Training Program on Work Capacity, Body Composition, and Blood Profiles in Sedentary Obese Men: A Pilot Study.* BioMed Research International, 2014. **2014**: p. 191797.
18. Ludin, A.F.M., et al., *High intensity circuit training on body composition, cardiovascular risk factors and physical fitness status among overweight and obese female students.* J Phys Activity, Sports Exerc, 2015. **3**: p. 40-48.
19. Ware Jr, J.E., *SF-36 Health Survey*, in *The use of psychological testing for treatment planning and outcomes assessment, 2nd ed.* 1999, Lawrence Erlbaum Associates Publishers: Mahwah, NJ, US. p. 1227-1246.
20. Sperlich, B., et al., *A 4-Week Intervention Involving Mobile-Based Daily 6-Minute Micro-Sessions of Functional High-Intensity Circuit Training Improves Strength and Quality of Life, but Not Cardio-Respiratory Fitness of Young Untrained Adults.* Frontiers in Physiology, 2018. **9**(423).
21. Romeo, A., et al., *Can Smartphone Apps Increase Physical Activity? Systematic Review and Meta-Analysis.* J Med Internet Res, 2019. **21**(3): p. e12053.
22. American Academy of Sleep Medicine, *International classification of sleep disorders*. 2014.

23. Chung, K.F., et al., *Sleep hygiene education as a treatment of insomnia: a systematic review and meta-analysis.* Fam Pract, 2018. **35**(4): p. 365-375.
24. Morin, C.M. and N.H. Azrin, *Stimulus control and imagery training in treating sleep-maintenance insomnia.* Journal of Consulting and Clinical Psychology, 1987. **55**(2): p. 260-262.
25. Bélanger, L., et al., *Meta-analysis of sleep changes in control groups of insomnia treatment trials.* J Sleep Res, 2007. **16**(1): p. 77-84.
26. Peach, H., J.F. Gaultney, and D.D. Gray, *Sleep hygiene and sleep quality as predictors of positive and negative dimensions of mental health in college students.* Cogent Psychology, 2016. **3**(1).
27. Galambos, N.L., A.L. Howard, and J.L. Maggs, *Rise and Fall of Sleep Quantity and Quality With Student Experiences Across the First Year of University.* Journal of Research on Adolescence, 2011. **21**(2): p. 342-349.
28. Jansson-Fröjmark, M., J. Evander, and S. Alfonsson, *Are sleep hygiene practices related to the incidence, persistence and remission of insomnia? Findings from a prospective community study.* Journal of Behavioral Medicine, 2019. **42**(1): p. 128-138.
29. Zhang, J., et al., *Sleep Habits, Sleep Problems, Sleep Hygiene, and Their Associations With Mental Health Problems Among Adolescents.* J Am Psychiatr Nurses Assoc, 2018. **24**(3): p. 223-234.
30. Goodman, R., *The Strengths and Difficulties Questionnaire: a research note.* Journal of child psychology and psychiatry, 1997. **38**(5): p. 581-586.
31. Friedrich, A. and A.A. Schlarb, *Let's talk about sleep: a systematic review of psychological interventions to improve sleep in college students.* J Sleep Res, 2018. **27**(1): p. 4-22.
32. Hertenstein, E., et al., *Insomnia as a predictor of mental disorders: A systematic review and meta-analysis.* Sleep Med Rev, 2019. **43**: p. 96-105.
33. Buysse, D.J., et al., *The Pittsburgh Sleep Quality Index: a new instrument for psychiatric practice and research.* Psychiatry Res, 1989. **28**(2): p. 193-213.
34. Grigsby-Toussaint, D.S., et al., *Sleep apps and behavioral constructs: A content analysis.* Preventive Medicine Reports, 2017. **6**: p. 126-129.
35. Gerow, M. and P.J. Bruner, *Exercise Induced Asthma.* Treasure Island, FL: StatPearls, 2020.
36. Nicholl, J., P. Coleman, and B. Williams, *The epidemiology of sports and exercise related injury in the United Kingdom.* British journal of sports medicine, 1995. **29**(4): p. 232-238.
37. Franklin, B.A. and S. Billecke, *Putting the Benefits and Risks of Aerobic Exercise in Perspective.* Current Sports Medicine Reports, 2012. **11**(4): p. 201-208.
38. Antony, M.M., et al., *Psychometric properties of the 42-item and 21-item versions of the Depression Anxiety Stress Scales in clinical groups and a community sample.* Psychological assessment, 1998. **10**(2): p. 176.
39. Lovibond, S.H. and P.F. Lovibond, *Manual for the depression anxiety stress scales (2nd edition)*. 1995, Psychology Foundation: Sydney, NSW, Australia.
40. Villar, S.S., J. Bowden, and J. Wason, *Multi-armed Bandit Models for the Optimal Design of Clinical Trials: Benefits and Challenges.* Stat Sci, 2015. **30**(2): p. 199-215.
41. Kessler, R. and D. Mroczek, *An update of the development of mental health screening scales for the US National Health Interview Study.* Ann Arbor: University of Michigan, Survey Research Center of the Institute for Social Research, 1992.
42. Australian Bureau of Statistics, *Information Paper: Use of the Kessler Psychological Distress Scale in ABS Health Surveys, Australia, 2007-08*. 2012, Commonwealth of Australia: Canberra, ACT, Australia.
43. van Spijker, B.A.J., et al., *The suicidal ideation attributes scale (SIDAS): Community-based validation study of a new scale for the measurement of suicidal ideation.* Suicide and Life-Threatening Behavior, 2014. **44**(4): p. 408-419.
44. Dorsey, E.R., B. Kluger, and C.H. Lipset, *The New Normal in Clinical Trials: Decentralized Studies.* Annals of Neurology, 2020. **88**(5): p. 863-866.
45. Greenwood, J.L., E.A. Joy, and J.B. Stanford, *The Physical Activity Vital Sign: a primary care tool to guide counseling for obesity.* Journal of Physical Activity and Health, 2010. **7**(5): p. 571-576.
46. Buysse, D.J., et al., *The Pittsburgh Sleep Quality Index: a new instrument for psychiatric practice and research.* Psychiatry research, 1989. **28**(2): p. 193-213.
47. UNSW Research Ethics & Compliance Support. *Clinical Trials Research Governance*. 2020 01 Dec 2020]; Available from: https://research.unsw.edu.au/clinical-trials-research-governance.
48. Bhatt, A., *Protocol deviation and violation.* Perspect Clin Res, 2012. **3**(3): p. 117.
49. Benjamini, Y. and Y. Hochberg, *Controlling the False Discovery Rate: A Practical and Powerful Approach to Multiple Testing.* Journal of the Royal Statistical Society. Series B (Methodological), 1995. **57**(1): p. 289-300.

50. Althunian, T.A., et al., *Defining the noninferiority margin and analysing noninferiority: An overview.* Br J Clin Pharmacol, 2017. **83**(8): p. 1636-1642.
51. Thompson, E.R., *Development and Validation of an Internationally Reliable Short-Form of the Positive and Negative Affect Schedule (PANAS).* Journal of Cross-Cultural Psychology, 2007. **38**(2): p. 227-242.
52. UNSW Research Infrastructure. *Data Storage and Tools*. 2020 01 Dec 2020]; Available from: https://research.unsw.edu.au/data-storage-and-tools.
53. Kessler, R.C., et al., *Short screening scales to monitor population prevalences and trends in non-specific psychological distress.* Psychol Med, 2002. **32**(6): p. 959-76.
54. Australian Bureau of Statistics. *Indigenous Status Standard*. 2014; Available from: https://www.abs.gov.au/statistics/standards/indigenous-status-standard/latest-release#definition-of-variable.
55. Australian Bureau of Statistics. *Ancestry Standard*. 2014; Available from: https://www.abs.gov.au/statistics/standards/ancestry-standard/latest-release.
56. Bouwmans, C., et al., *The iMTA Productivity Cost Questionnaire: A Standardized Instrument for Measuring and Valuing Health-Related Productivity Losses.* Value Health, 2015. **18**(6): p. 753-8.
57. Burgess, P.M., et al., *Service Use for Mental Health Problems: Findings from the 2007 National Survey of Mental Health and Wellbeing.* Australian & New Zealand Journal of Psychiatry, 2009. **43**(7): p. 615-623.
58. Herdman, M., et al., *Development and preliminary testing of the new five-level version of EQ-5D (EQ-5D-5L).* Qual Life Res, 2011. **20**(10): p. 1727-36.
59. Adler, N.E., et al., *Relationship of subjective and objective social status with psychological and physiological functioning: preliminary data in healthy white women.* Health Psychol, 2000. **19**(6): p. 586-92.
60. National Institute on Drug Abuse, *NIDA Drug Screening Tool, NIDA-Modified ASSIST (NM ASSIST)*. 2020, National Institutes of Health: Bethseda, MA, USA.
61. Stewart-Brown, S., et al., *Internal construct validity of the Warwick-Edinburgh mental well-being scale (WEMWBS): A rasch analysis using data from the Scottish health education population survey.* Health Qual Life Outcomes, 2009. **7**: p. 15.
62. Zimet, G.D., et al., *The multidimensional scale of perceived social support.* Journal of personality assessment, 1988. **52**(1): p. 30-41.
63. Devilly, G.J. and T.D. Borkovec, *Psychometric properties of the credibility/expectancy questionnaire.* J Behav Ther Exp Psychiatry, 2000. **31**(2): p. 73-86.
64. Tambling, R.B. and S.A. Ketring, *The R-URICA: A Confirmatory Factor Analysis and a Revision to the URICA.* Contemporary Family Therapy, 2014. **36**(1): p. 108-119.
65. Brooke, J., *SUS: a "quick and dirty" usability scale*, in *Usability evaluation in industry*, P.W. Jordan, et al., Editors. 1996, Taylor & Francis Ltd.: London, UK. p. 189.
66. Zhou, L., et al., *The mHealth App Usability Questionnaire (MAUQ): Development and Validation Study.* JMIR Mhealth Uhealth, 2019. **7**(4): p. e11500.
