# Supplementary material for: Brief Digital Interventions for Psychological Distress: An AI-Enhanced Response-Adaptive Randomized Clinical Trial
Source: JAMA Netw Open. 2025 Oct 31;8(10):e2540502. doi: 10.1001/jamanetworkopen.2025.40502 (PMC12579342; doi:10.1001/jamanetworkopen.2025.40502)
Supplement: Supplement 2. — eFigure 1. Trial Schematic eMethods 1. Method of Calculating Severity Scores eMethods 2. Contextual Multi-Arm Bandit Algorithme Figure 2. Overview of the Contextual Multi-Arm Bandit (MAB) Algorithm Framework eFigure 3. Illustration of How the Contextual MAB Algorithm Allocates Participants to an Interventione Table 1. Intervention Content eMethods 3. Statistical Analysis Plan eFigure 4. Hypothesis Testing Procedure and Processing of DASS-21 Scores Including Bias Adjustment and Log Transformation eTable 2. Maximum P Value for Each Distress Severity Group at Each Interim Analysis and Final Analysis eTable 3. Detailed Breakdown Used to Calculate Final Maximum P Values for Each Distress Severity Group eTable 4. Number of Participants Per Distress Severity Group Included in the Interim Analyses and Final Analysis eTable 5. Interim Hypothesis Tests After Mini-Trials 4 and 8 for DASS-21 Total Scores eResults. eFigure 5. Number of Participants in the Mild, Moderate, and Severely Distressed Subgroups With Scores in the Normal/Mild, Moderate, and Severe/Extremely Severe Ranges on the DASS-21 Depression, Anxiety and Stress Subscales at Baseline eFigure 6. Mean Change in DASS-21 Total Score From Pre- to Post-Intervention (Top), and the Number of Participants (Bottom) Allocated Across Each of the 12 Mini-Trials, for Mild (a), Moderate (b), and Severe (c) Distress Groups eTable 6. Observed DASS-21 Total Scores for the Mild Distress Group by Intervention Group Across Each of the Twelve Mini-Trials eTable 7. Observed DASS-21 Total Scores for the Moderate Distress Group by Intervention Group Across Each of the Twelve Mini-Trials eTable 8. Observed DASS-21 Total Scores for the Severe Distress Group by Intervention Group Across Each of the Twelve Mini-Trials eTable 9. Unweighted Observed Means and Standard Deviations for DASS-21 Depression Subscale Scores Pre- and Post-Intervention, and Change by Severity and Intervention Group eTable 10. Unweighted Observed Means and Standard Deviati [file jamanetwopen-e2540502-s002.pdf]

Supplementary Online Content

Newby J, Gupta S, Hoon L, et al. Brief digital interventions for psychological distress: an AI-enhanced response-adaptive randomized clinical trial. *JAMA Netw Open.* 2025;8(10):e2540502. doi:10.1001/jamanetworkopen.2025.40502

eFigure 1. Trial Schematic
eMethods 1. Method of Calculating Severity Scores
eMethods 2. Contextual Multi-Arm Bandit Algorithm
eFigure 2. Overview of the Contextual Multi-Arm Bandit (MAB) Algorithm Framework
eFigure 3. Illustration of How the Contextual MAB Algorithm Allocates Participants to an Intervention
eTable 1. Intervention Content
eMethods 3. Statistical Analysis Plan
eFigure 4. Hypothesis Testing Procedure and Processing of DASS-21 Scores Including Bias Adjustment and Log Transformation
eTable 2. Maximum *P* Value for Each Distress Severity Group at Each Interim Analysis and Final Analysis
eTable 3. Detailed Breakdown Used to Calculate Final Maximum *P* Values for Each Distress Severity Group
eTable 4. Number of Participants Per Distress Severity Group Included in the Interim Analyses and Final Analysis
eTable 5. Interim Hypothesis Tests After Mini-Trials 4 and 8 for DASS-21 Total Scores
eResults.
eFigure 5. Number of Participants in the Mild, Moderate, and Severely Distressed Subgroups With Scores in the Normal/Mild, Moderate, and Severe/Extremely Severe Ranges on the DASS-21 Depression, Anxiety and Stress Subscales at Baseline
eFigure 6. Mean Change in DASS-21 Total Score From Pre- to Post-Intervention (Top), and the Number of Participants (Bottom) Allocated Across Each of the 12 Mini-Trials, for Mild (a), Moderate (b), and Severe (c) Distress Groups
eTable 6. Observed DASS-21 Total Scores for the Mild Distress Group by Intervention Group Across Each of the Twelve Mini-Trials
eTable 7. Observed DASS-21 Total Scores for the Moderate Distress Group by Intervention Group Across Each of the Twelve Mini-Trials
eTable 8. Observed DASS-21 Total Scores for the Severe Distress Group by Intervention Group Across Each of the Twelve Mini-Trials
eTable 9. Unweighted Observed Means and Standard Deviations for DASS-21 Depression Subscale Scores Pre- and Post-Intervention, and Change by Severity and Intervention Group
eTable 10. Unweighted Observed Means and Standard Deviations for DASS-21 Anxiety Subscale Scores Pre- and Post-Intervention, and Change by Severity and Intervention Group

© 2025 Newby J et al. *JAMA Network Open.*

eTable 11. Unweighted Observed Means and Standard Deviations for DASS-21 Stress Subscale Scores Pre- and Post-Intervention, and Change by Severity and Intervention Group
eTable 12. Group Comparisons of the Pre- to Post-Changes in Bias Corrected and Log Transformed DASS-21 Depression Subscale Scores
eTable 13. Group Comparisons of the Pre- to Post-Changes in Bias Corrected and Log Transformed DASS-21 Anxiety Subscale Scores
eTable 14. Group Comparisons of the Pre- to Post-Changes in Bias Corrected and Log Transformed for DASS-21 Stress Subscale Scores
eTable 15. Unweighted Observed Means and Standard Deviations for Sleep Quality Scores Pre- and Post-Intervention, and Change by Severity and Intervention Group
eTable 16. Unweighted Observed Means and Standard Deviations for Mindfulness Scores Pre- and Post-Intervention, and Change by Severity and Intervention Group
eTable 17. Unweighted Observed Means and Standard Deviations for Physical Activity Vital Sign Scores Pre- and Post-Intervention, and Change by Severity and Intervention Group
eTable 18. Group Comparisons of the Pre- to Post-Changes in Bias Corrected and Log Transformed Sleep Quality Scores
eTable 19. Group Comparisons of the Pre- to Post-Changes in Bias Corrected and Log Transformed Mindfulness Scores
eTable 20. Group Comparisons of the Pre- to Post-Changes in Bias Corrected and Log Transformed Physical Activity Vital Sign Scores
eTable 21. Descriptive Statistics for the App User Engagement Metrics Across Groups
eReferences.

This supplementary material has been provided by the authors to give readers additional information about their work.

eFigure 1. Trial Schematic

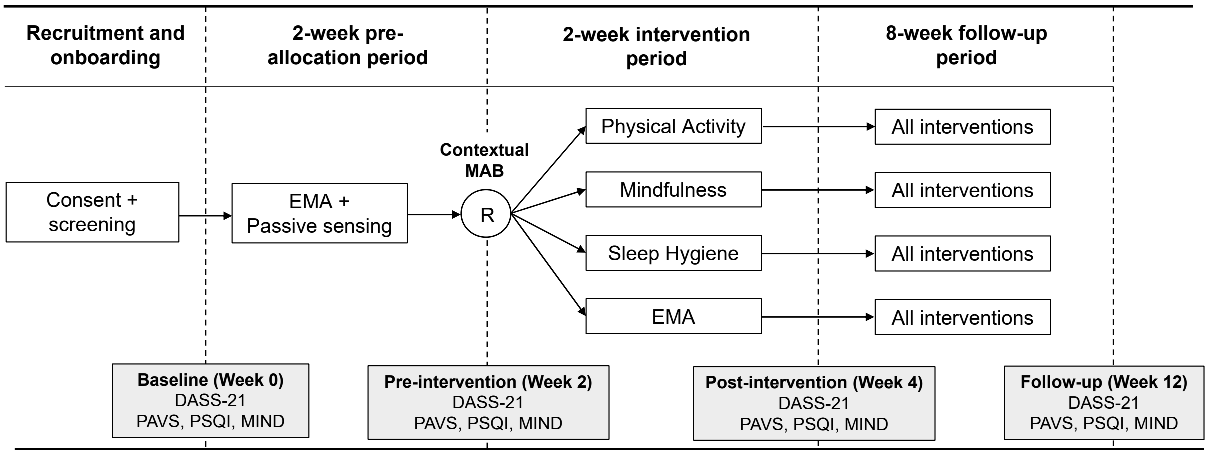

eFigure1 describes the design of each of the 12 mini-trials. Interim analyses were conducted after mini-trial 4 and mini-trial 8, and final testing after mini-trial 12.

© 2025 Newby J et al. *JAMA Network Open.*

eMethods 1. Method of Calculating Severity Scores

The participants were classified into mild, moderate, and severe distress categories based on their normalised baseline DASS-21 scores. Firstly, we computed the total scores for the Depression, Anxiety, and Stress subscales by summing the scores for each item on the relevant subscale, multiplying the subscale score by two, and then standardised each subscale score individually using the instructions in the DASS-21 scoring manual [1]. For the Depression subscale, we employed a normalisation mean of 6.35 and a standard deviation of 6.85. Similarly, for the Anxiety subscale, the normalisation parameters were a mean of 4.77 and a standard deviation of 4.79, while for the Stress subscale, they were a mean of 11.19 and a standard deviation of 8.25. Following normalisation, we averaged the standardised scores of the three subscales to generate a composite statistic, which we utilised to assign participants to severity groups. Participants with a statistic less than 1.0 were placed in the "Mild" group. Those with a statistic greater than or equal to 1.0 but less than 2.0 were assigned to the "Moderate" group, while participants with a statistic greater than or equal to 2.0 were categorised into the "Severe" group.

The normalised total DASS-21 score for a given participant was calculated as follows:

$$S = \left(\frac{DASS_{depression} - 6.35}{6.85} + \frac{DASS_{anxiety} - 4.77}{4.79} + \frac{DASS_{stress} - 11.19}{8.25} \right) \div 3 \qquad (1)$$

Equation 1

Where $DASS_{depression}$, $DASS_{anxiety}$, and $DASS_{stress}$ are a participant's DASS-21 depression, anxiety and stress subscale scores respectively. These values were normalised and averaged to calculate the normalised DASS-21 total score as per Equation 1[1]. The result (S) is a single numerical value for each assessment time point.

The *score* used to measure the impact of an intervention was the difference between the normalised pre- and post- intervention DASS-21 total score, S_{pre} and S_{post}, respectively, as defined as:

$$score = S_{pre} - S_{post} \qquad (2)$$

Equation 2

© 2025 Newby J et al. *JAMA Network Open.*

eMethods 2. Contextual Multi-Arm Bandit Algorithm

The algorithm applied in the study was a Multi-Arm Bandit (MAB) algorithm with contextual information. The code will be made available upon reasonable request. The MAB algorithm was used to allocate participants enrolled in Mini-trials 2 – 12 (inclusive). MABs are a sample efficient method of determining the best possible intervention to prescribe to a participant. In contextual MABs, a participant's specific characteristics (e.g., age, gender, distress level) – referred to as their context features – are used to incorporate more information about an individual to recommend an intervention [2]. In this trial, the context feature was a participants' normalised DASS-21 total score at the baseline assessment.

© 2025 Newby J et al. *JAMA Network Open.*

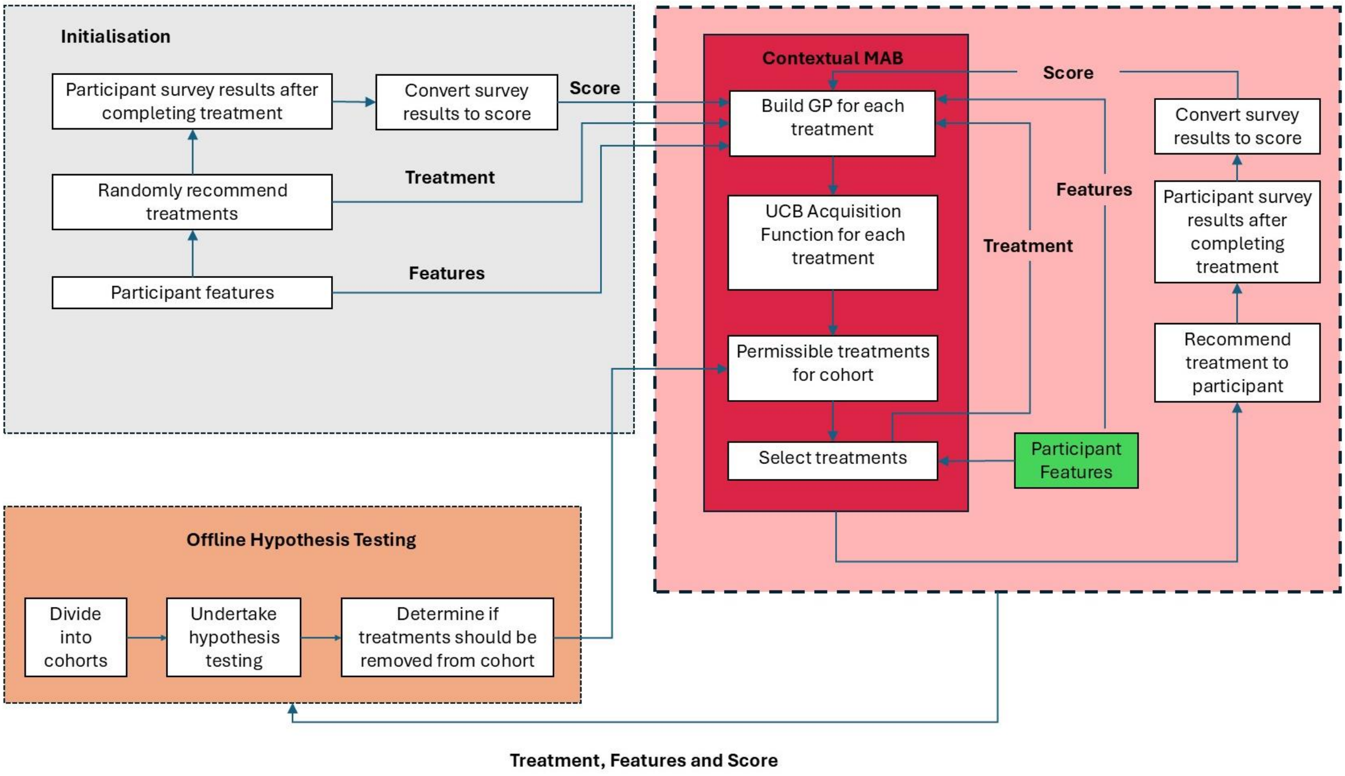

eFigure 2. Overview of the Contextual Multi-Arm Bandit (MAB) Algorithm Framework. GP = Gaussian Process, UCB = Upper Confidence Bound.

In the first mini-trial, participants were randomly assigned to interventions. The results of these participants corresponding to their pre-to post-treatment change scores on the DASS-21 survey were converted to a score which was then fed into the Contextual MAB algorithm. The Contextual MAB provided recommended interventions based on participant features. At a high level, the algorithm operated as follows:

For mini-trial 1:

(1) Participants who completed the baseline DASS-21 were randomly allocated to interventions with the ratio 1:1:1:1. In order to determine allocation in the first mini trial, the interventions (mindfulness, physical activity, sleep hygiene, ecological momentary assessment) were randomised through a computer generated random number generator. Then interventions were allocated in a round-robin fashion, in a 1:1:1:1 ratio to each participant once they had completed their baseline assessment surveys. Participants completed two additional DASS surveys; a pre-intervention and post-treatment (post-) survey. Participants were told their intervention allocation within the app immediately after completing the pre-intervention survey. For each survey (baseline, pre-intervention, and post-intervention), a normalised DASS-21 total score was calculated as per Equation 1.

© 2025 Newby J et al. *JAMA Network Open.*

(2) The difference between a participant's normalised pre- and post-intervention DASS-21 total scores was used as a measure of the impact of the intervention (referred to as the '*score'* variable in eFigure2) and calculated as per Equation 2. A positive value indicated an improvement between pre- and post-assessment. The normalised baseline DASS-21 total score is referred to as the contextual *feature*. The *score, feature* and allocated *intervention* were fed into the Contextual MAB algorithm. Only data of participants who had completed both the pre-intervention and post-intervention DASS-21 surveys were used in the Contextual MAB.

(3) The *score*, *feature* and *intervention* information were used to fit a Gaussian Process (GP) model for each intervention. For example, only data from participants allocated to the mindfulness intervention were used to fit the mindfulness Gaussian Process model. Details on the fitting of the Gaussian Process model is discussed in the Gaussian Process Model section. The Contextual MAB is made up of a Gaussian Process for each intervention.

For mini-trials 2 – 12:

(4) For mini-trials 2-12, a participant's normalised baseline DASS-21 total score (*feature)* was calculated and used to classify them into a distress severity group as per our published protocol [1].

(5) This *feature* was used to select and recommend the best intervention for a given individual via the Contextual MAB. Only interventions that were still active for allocation[1] were available to be selected from. Participants' intervention recommendation was revealed to them after they completed the pre-intervention DASS-21 survey. They had two weeks' access to the intervention (or control) before their post-intervention DASS-21 total survey scores. The participant's pre- and post- intervention DASS-21 total survey results for the intervention (or control) period were then converted into a *score*, as per Equation 1 and Equation 2.

(6) After each mini-trial, participants' *scores, features* and *interventions* were fed back into the Gaussian Process model and used to refit the model. Only data of participants who completed both pre- and post-intervention DASS-21 surveys were used to fit the Gaussian Process. All participant data that had been accumulated from the prior mini-trials was used to fit the Gaussian Process. For example, after mini-trial 4, the Gaussian Process model was fit with participant data from mini-trials 1, 2, 3 and 4.

(7) This process returned to step (4) and continued until mini-trial 12 was completed.

Gaussian Process Model

The Contextual MAB works by building a probabilistic model for each of the interventions relating the participants' *features* with *scores*. A commonly used probabilistic model is a Gaussian Process (GP) which is fully defined by a mean and covariance function [3]. Prior to fitting the GP, the normalised baseline DASS-21 total score (*feature)* was scaled to range between 0 and 1, and the change in the

[1] We performed interim hypothesis tests at mini trial 4 and 8 and as a result, active interventions (I) were those that had not been removed following an interim hypothesis test.

© 2025 Newby J et al. *JAMA Network Open.*

normalised pre- and post-intervention DASS-21 total score (*score)* was standardised (mean of 0 and standard deviation of 1). Equation 3 and Equation 4 show the formulation of the mean and variance of the GP model, where y is the standardised *score* and x is the normalised *feature*. The smoothness of the model is defined through the covariance function, that is, how varied scores are for nearby context features. As x is a single continuous variable in our study, a squared exponential kernel was used, parameterised by a length scale (l), the form of which is shown in Equation 5. The length scale was tuned each time a GP was fitted (as long as the number of data points within the GP was higher than 50). If the number of data points was above 50, tuning was done by searching for a length scale value within a vector of values [0.05, 0.1, 0.2, 0.3], to find the one which maximised the log likelihood [3]. If there were not yet 50 data points for a given intervention GP, the length scale was set to a default value of 0.1. Additionally, noise was added to the kernel to capture the variance in the scores. The noise was set to $\sigma_n = 0.1$. For participants who had both the same *feature* and allocated the same intervention, their scores were averaged before being used to fit the GP to avoid errors due to the matrix ill-conditioning.

$$\mu_i(x_{t+1}) = \boldsymbol{k}^T(\boldsymbol{K} + \sigma_n^2\boldsymbol{I})^{-1}y_{\boldsymbol{1:t}} \qquad (3)$$

Equation 3

$$\sigma_i{}^2(x_{t+1}) = k_i(x_{t+1}, x_{t+1}) - \boldsymbol{k}^T(\boldsymbol{K})^{-1}\boldsymbol{k} \qquad (4)$$

Equation 4

Where i is the intervention and t is the total number of data points collected so far for that intervention and

$$k(x_i, x_j) = \exp\left(-\frac{1}{2l^2}\left\|x_i - x_j\right\|^2\right)$$

Equation 5

$$(5)$$

$$\boldsymbol{K} = \begin{bmatrix} k(x_1, x_1) & \cdots & k(x_1, x_t) \\ \vdots & \ddots & \vdots \\ k(x_t, x_1) & \cdots & k(x_t, x_t) \end{bmatrix}$$

$$\boldsymbol{k} = [k(x_{t+1}, x_1) \quad \ldots \quad k(x_{t+1}, x_t)].$$

Acquisition Function

Once fitted, a GP was used to produce a predictive distribution, i.e. a mean and standard deviation prediction for how a new participant (who had not yet undertaken an intervention) would respond to each active intervention or control. This information was used to recommend an intervention (or control) that would be most likely to be beneficial for that participant. This proceeded as follows. When a new participant completed their baseline DASS-21 survey they were categorised into one of three (mild, moderate, severe) distress severity groups (c). Their baseline DASS-21 survey score was then converted into a *feature* (x_p). Using this feature, a

© 2025 Newby J et al. *JAMA Network Open.*

prediction of likely treatment effects could be made for each intervention (I_c) GPs. The mean $\mu_i(x_p)$ and standard deviation $\sigma_i(x_p)$ prediction from each GP (indexed by i) for the participant with features x_p was used to construct an Upper Confidence Bound (UCB) acquisition function [4], the form of which is shown in Equation 6.

$$UCB(x_p) = \mu_i(x_p) + \sqrt{\beta_t}\sigma_i(x_p) \tag{6}$$

Equation 6

Where β_t takes the form given by [4].

The UCB acquisition function balances the trade-off between acting exploitatively (recommending interventions which are known to produce positive outcomes given the participants' features) and exploratively (recommending interventions which have not been tested for the participants' features and have a high level of uncertainty). The intervention (i) from the set of possible available interventions (I_c) with the highest UCB value is recommended to the participant in the next iteration as described in Equation 7.

$$i = \underset{i \in I_c}{\text{argmax}}\left(UCB(x_p)\right) \tag{7}$$

Equation 7

© 2025 Newby J et al. *JAMA Network Open.*

eFigure 3. Illustration of How the Contextual MAB Algorithm Allocates Participants to an Intervention

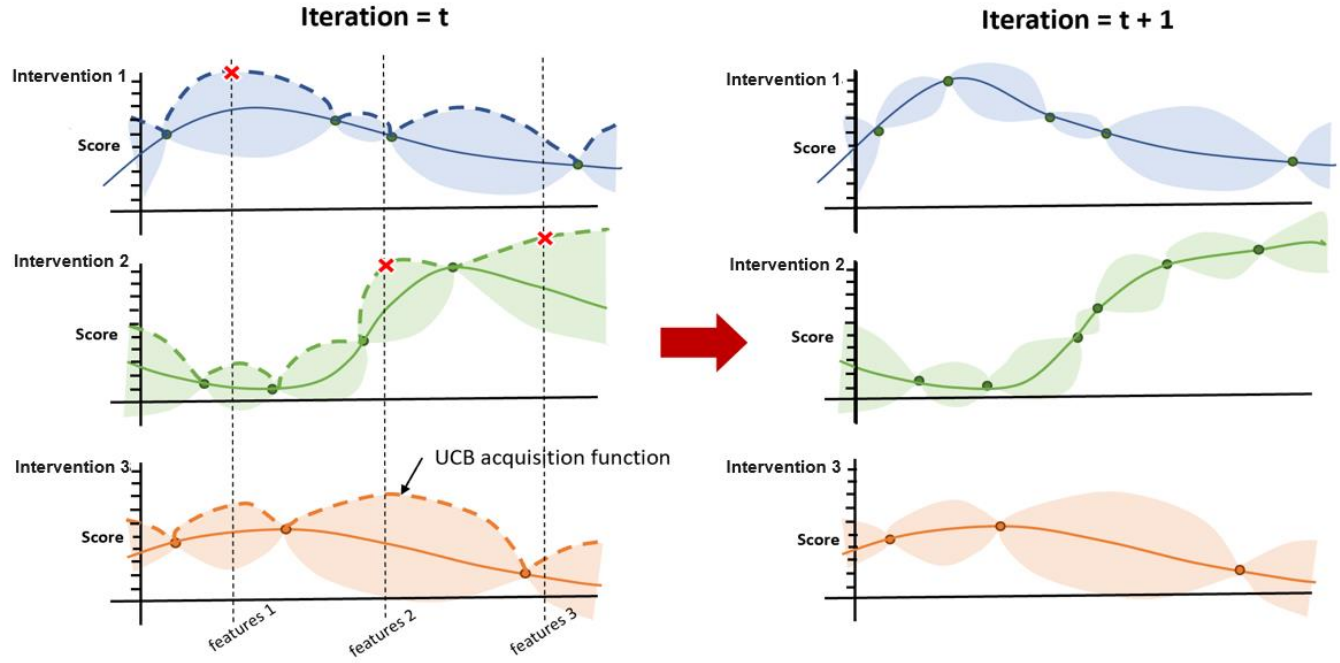

eFigure 3. An iteration of the Contextual MAB algorithm. Left plot uses example fictional participants to illustrate the process by which new participants were recommended an intervention. In this diagram, for participant 1 (feature 1), intervention 1 returned the highest UCB function value, whilst for participant 2 (feature 2) and participant 3 (feature 3), intervention 2 returned the highest value. After the intervention recommendation was given to the participant, and when their completed pre- and post- intervention results were returned, converted to a *score* and standardised, these were used to refit the necessary GPs, along with all previous data (as shown in the right plot). This process continued until mini-trial 12.

In iteration = t, participant scores are fit to the individual GPs. The UCB acquisition function is derived for each intervention GP (dashed line) using the mean (solid line) and the standard deviation (shaded area). For each participant, their features (e.g. feature 1 = features for participant 1) are used to select the intervention for which the UCB function, at that feature value, returns the highest value (marked by red cross). This intervention is then recommended to the participant and the resulting pre-to-post intervention scores are used to update the GPs, as shown in iteration = t + 1

© 2025 Newby J et al. *JAMA Network Open.*

Interventions

Screenshots of the Vibe Up smartphone app

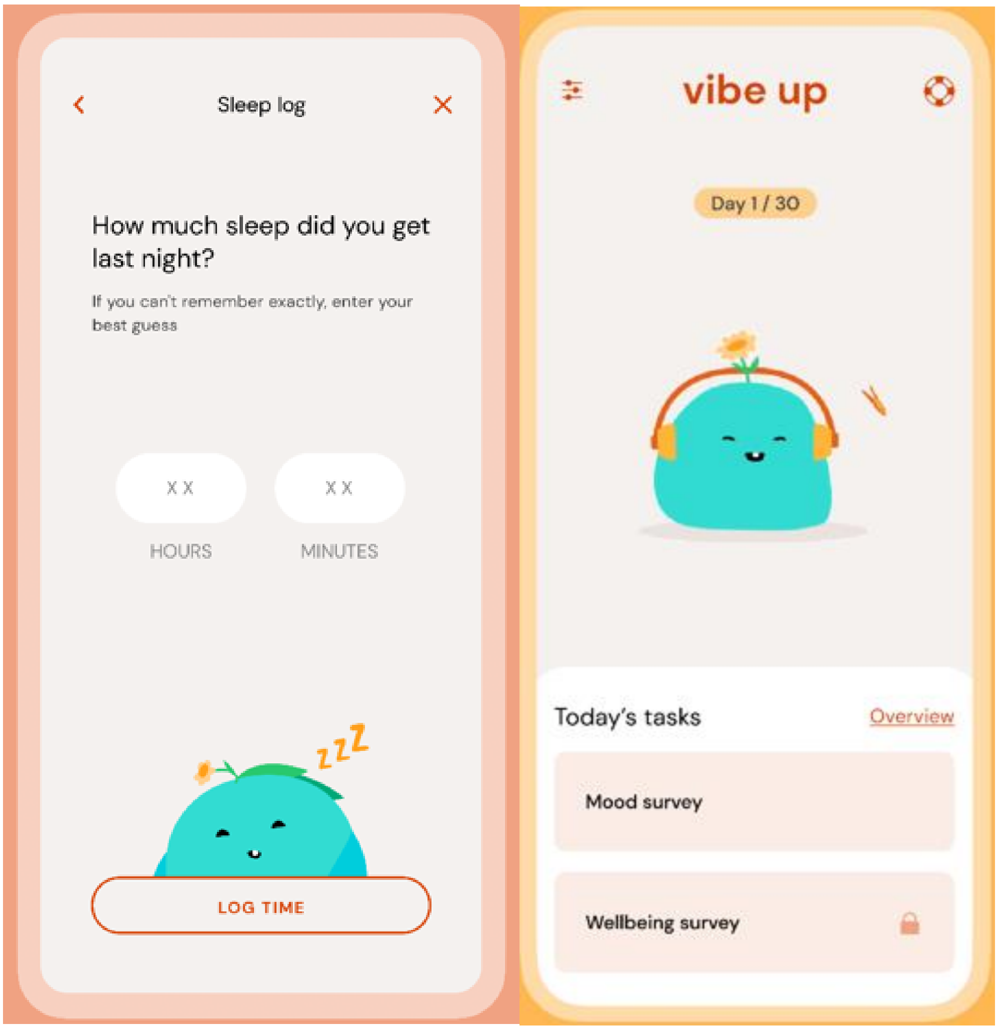

© 2025 Newby J et al. *JAMA Network Open*.

eTable 1. Intervention Content

Module Number	Module release Day	Name	Summary of content	Format	Length
**Mindfulness Intervention**					
Introduction	Day 1 of the intervention period	Introduction	• Explanation of mindfulness and its benefits for college students.<br>• Explanation of the difference between formal and informal mindfulness practice.<br>• Tuning into the five senses to improve mindful awareness of daily activities.<br>• Reducing judgment and increasing self-compassion.	Video	3 minutes
1	Day 1 of the intervention period, or after completion of the introductory video	Mindful breathing	Guided mindfulness practice involving mindful awareness of breathing.	Audio, with choice of male or female voice	4 minutes
2	Day 2 of the intervention period, or one day after the release of Mindful	Unhooking from thoughts	Guided mindfulness practice involving noticing, awareness and acceptance of thoughts, a non-judgmental attitude towards thoughts, including ‘leaves on a stream’ imagery.	Audio, with choice of male or female voice.	5 minutes

© 2025 Newby J et al. *JAMA Network Open.*

	breathing				
3	Day 3 of the intervention period, or one day after the release of unhooking from thoughts	Body scan	Guided mindfulness practice based on mindful awareness of body sensations, to encourage noticing, awareness and acceptance of bodily sensations.	Audio, with choice of male or female voice.	5 minutes
4	Day 4 of the intervention period, or one day after the release of Body scan	Mindful eating	Guided mindfulness practice encouraging the use of all five senses to bring curiosity to eating.	Audio, with choice of male or female voice.	4 minutes
5	Day 5 of the intervention period, or one day after the release of Mindful eating	Mindful walking	Guided mindfulness practice blending awareness of surrounding environment and bodily sensations.	Audio, with choice of male or female voice.	4 minutes
**Physical Activity Intervention**					
**Module Number**	**Module release Day**	**Name**	**Summary of content**	**Format**	**Length**
1	Day 1 of the intervention period	Introduction	• Benefits of physical activity for cognition, mental health and physical health in college students.<br>• Australian guidelines for physical activity.<br>• Setting realistic goals and benefits of small changes in	Infographic	Reading time: <5 minutes

© 2025 Newby J et al. *JAMA Network Open.*

			physical activity.<br>• Tips to increase physical activity:<br>○ Choosing enjoyable activities<br>○ Setting goals and tracking progress<br>○ Being social<br>Increasing step count		
2	Available immediately after a participant has completed the introductory infographic	Daily goal setting	Participants choose from the following options:<br>• Increasing step count<br>• 7-minute HIIT workout<br>• Other activity (social sport, gardening, yoga, bike riding, dancing, etc)<br>Rest day	Interactive app cards, with informational text tailored to the option selected.	<3 minutes
3	Available immediately after a participant has completed the introductory infographic	High Intensity Interval Training (HIIT) workout	HIIT protocol consisting of the following 12 exercises (each performed for 30 seconds, with 10 second interim rest periods):<br>1. Jumping jacks<br>2. Wall sit<br>3. Push up<br>4. Abdominal crunch<br>5. Step up onto a chair<br>6. Squat<br>7. Triceps dip on chair<br>8. Plank<br>9. High knees<br>10. Lunge<br>11. Push-up and rotation<br>12. Side plank	Video	7 minutes

© 2025 Newby J et al. *JAMA Network Open.*

**Sleep Hygiene Intervention**					
**Module Number**	**Module release day**	**Name**	**Content**	**Format**	**Length**
1	Available to participants immediately after allocation to the intervention	Why sleep?	• Recommended hours of sleep per night.<br>• Impact of lack of sleep on cognition and emotion.<br>• Benefit of sleep for mental health and physical health.<br>Introduction to sleep hygiene.	Infographic	Reading time: <5 minutes
2	Available immediately after a participant has completed Module 1	Sleep habits	• Establishing a regular bedtime and wake time.<br>• Eliminating (or limiting) naps.<br>Establishing a wind down routine.	Infographic	Reading time: <5 minutes
3	Available two days after Module 2 is made available (regardless of participant engagement)	Sleep environment	• Reducing light, noise and temperature disturbance, and ensuring bedding is comfortable.<br>• Limiting use of bed to sleep and sex.<br>• The impact of electronic devices on sleep; avoiding screens/blue light prior to bedtime, and not sleeping with a phone.<br>Get out of bed when unable to sleep for 20 minutes or more, do something relaxing and return to bed when sleepy.	Infographic	Reading time: <5 minutes

© 2025 Newby J et al. *JAMA Network Open.*

4	Available two days after Module 3 is made available (regardless of participant engagement)	Daily activities for sleep	• Reducing caffeine, alcohol, and nicotine intake; especially close to bedtime.<br>• Eating a healthy diet and considering timing of food/fluid intake.<br>• Increasing physical activity and avoiding exercise close to bedtime.	Infographic	Reading time: <5 minutes

Note. Table adapted from [5]

Ecological Momentary Assessment Description

Participants in this trial arm had the option of completing two signal contingent EMA surveys per day, as well as event-contingent EMA. For the signal-contingent EMA, the Vibe Up Smartphone app generated two daily prompts, within two separate windows of time: morning (08:00-10:00) and evening (19:00-21:00) according to the participants' local time. The prompts were sent at a random time within each window. Participants were sent a notification from the Vibe Up app, and had up to 60 minutes to respond to the prompt, with a reminder sent after 30 minutes for participants who had not completed the surveys.

In addition, participants could also complete event-contingent EMA. That is, they could log an EMA survey at any time during the day in response to a self-identified stressor. If a participant initiated an event-contingent EMA survey within the morning (08:00-10:00) or evening (19:00-21:00) windows of time, then it stopped the Vibe Up app from sending a signal-contingent EMA notification reminder. This occurred regardless of whether the participant had completed the event-contingent EMA survey or not.

Each EMA survey contained questions about the participant's current positive and negative affect, and how likely they were to respond to the emotions they were currently experiencing. Participants in this group were informed that the EMA survey responses were not actively monitored, but were provided support options via a "Get Help" link in the app.

© 2025 Newby J et al. *JAMA Network Open.*

eMethods 3. Statistical Analysis Plan

Analysis Methodology

The goal of the optimisation was to identify the best performing intervention, defined as the intervention that yielded the greatest improvement in DASS-21 total scores between pre- and post- intervention. Assessment of whether the optimisation goal was satisfied was made through interim analyses conducted after mini-trials 4, 8 and the final analysis after mini-trial 12 using all data available up to that point in time. At the start of the trial, all interventions and the control were part of the available intervention set (I). If, for a given distress severity group, an intervention was found to be performing significantly better than all other available interventions and control, then this intervention was removed from the available intervention set. Having been removed, this best performing intervention is no longer available for recommendation to that distress severity group in further mini-trials. An intention-to-treat approach was used for the interim and final analyses, with the assumption that missing data was at random. To be included in the intent-to-treat sample, participants must have completed the baseline DASS-21 survey (to qualify being allocated to an intervention) and the pre-intervention DASS-21 survey. The interim hypothesis testing was completed within each distress severity group.

Interim Analyses

Following mini-trial 4, and 8, an interim analysis was conducted. The hypothesis test was conducted for each distress severity group in order to determine if any intervention was significantly outperforming all other interventions, within that severity group. If so, this intervention was to be removed from the list of active interventions and no longer available for allocation in future mini-trials for that distress severity group.

Hypothesis Testing Procedure

Detailed below are the results of the interim analyses conducted after mini-trial 4 and 8, as well as the final study results from the final analysis conducted after mini-trial 12. Results for both the primary (DASS-21 total score) and secondary outcomes (DASS-21 Depression, Anxiety and Stress subscales, Physical Activity Vital Sign, Mindfulness and Sleep quality) are provided. Treatment effect sizes for the three active interventions compared to the EMA control condition, calculated as the Standardised Mean Difference (SMD), are also provided.

Illustrated in eFigure 4 is the procedure by which data from the DASS-21 surveys, for each distress severity group, is extracted, transformed, and used to fit a mixed model repeated measures analysis of variance (MMRM) model in preparation for the hypothesis testing. A MMRM model was used as it is designed to model the response measured at two fixed time points (pre- and post- intervention) and can handle missing data (allowing the intention-to-treat modelling) [6]. From the MMRM model, planned contrasts between each intervention were conducted to compare the

© 2025 Newby J et al. *JAMA Network Open.*

difference in pre-intervention and post-intervention DASS-21 total scores, or other secondary outcome measures.

© 2025 Newby J et al. *JAMA Network Open.*

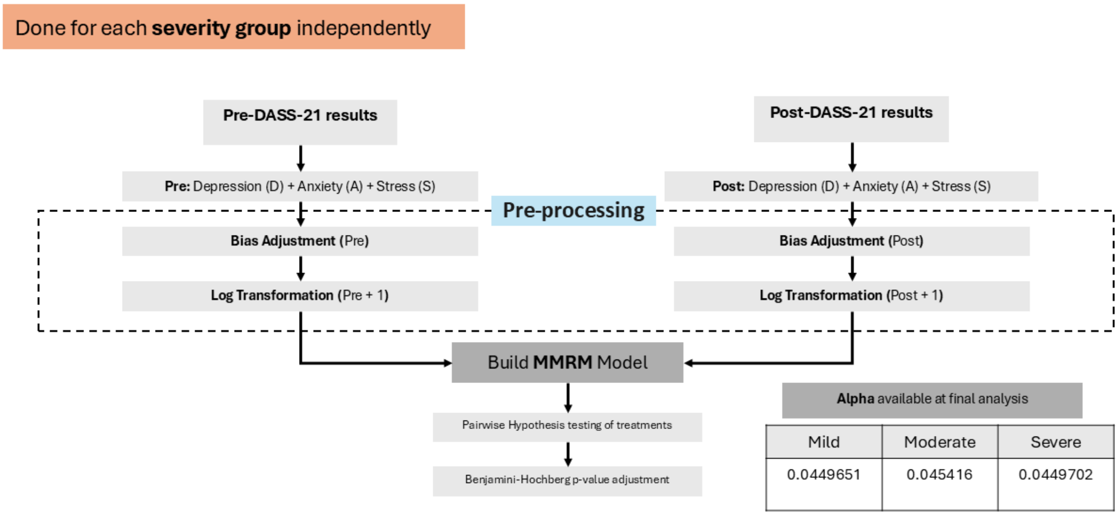

eFigure 4. Hypothesis Testing Procedure and Processing of DASS-21 Scores Including Bias Adjustment and Log Transformation

For each distress severity group:

(1) The Depression (D), Anxiety (A) and Stress (S) scores from the pre-intervention and post-intervention surveys are summated, respectively. These come to represent the score before intervention (pre) and after intervention (post). These scores are also called reward-pre and reward-post in the Contextual Multi-Arm Bandit (MAB) community, but will be referred to as pre-intervention and post-intervention for consistency.

(2) The pre and post values undergo an adjustment to account for the sampling bias induced by the sampling method of the Contextual Multi-Armed Bandit (MAB) algorithm employed during the trial for intervention allocation to participants. Details on this Bias Adjustment are detailed below.

(3) Due to an observed skewing in the data, a log transformation was applied to map to a normal distribution for analysis.

(4) Following this pre-processing, the data is used to fit an MMRM model from which pairwise hypothesis testing is conducted between each pair of available interventions (or control) for that distress severity group. To avoid the inflation of Type 1 errors from the multiple comparisons, a Benjamini-Hochberg Correction in the critical p-values values and an O'Brien-Fleming alpha spending procedure was employed. Details of the Benjamini-Hochberg Procedure and the alpha spending values per interim hypothesis test are detailed below.

(5) The results of the hypothesis testing are used to determine whether any interventions should be removed from the following mini-trials, and the available intervention set be updated. The criteria by which an intervention is removed is if it was found to be, for a given distress severity group,

© 2025 Newby J et al. *JAMA Network Open.*

significantly better than all other interventions. For example, if for the moderate distress group, the mindfulness intervention was found to be performing significantly better than all other interventions (and control) in a pair-wise comparison for that group, then mindfulness would be removed from the set of available interventions such that the updated available intervention set would become $I_{moderate} = \{Sleep\ Hygiene, Control, Physical\ Activity\}$.

Bias Adjustment

Due to the sampling bias induced by the Contextual MAB intervention allocation (compared to a Randomised Control Trial (RCT), bias adjustment was required. A Horvitz-Thompson (HT) Estimator with Inverse Propensity Weighting (IPW) adjustment was applied to the pre and post values [7].

Benjamini-Hochberg Procedure

The Benjamini-Hochberg procedure was applied to adjust the required critical p-value for statistical significance [8]. Given a maximum p-value available for a given interim hypothesis test (this value determined via the alpha-spending available), this p-value was weighted across each comparison, such that the lowest critical p-value was assigned to the comparison with the lowest p-value calculated, and the largest critical p-value for the comparison with the largest p-value calculated.

α-Spending

As the trial involved 3 hypothesis testing points (interim analyses after mini-trial 4, 8 and the final analysis after mini-trial 12), it was important that the α spent across all the analyses, for each distress severity group, totalled 0.05. The interim hypothesis tests 1 (after mini-trial 4) and 2 (after mini-trial 8) followed O'Brien & Fleming type α-spending [9] where the maximum p-value available (to then be split according to the Benjamini-Hochberg procedure) was 0.0006869 and 0.0161445, respectively, for each distress severity group. For the final hypothesis test, Rpact [10] was used to determine the final p-value for each distress severity group (to ensure the total α spend of 0.05 was met). This calculation was done retrospectively by examining the percentage of participants at each interim test. eTable2 details these values. eTable3 outlines the breakdown of participant numbers and α spent so far input into Rpact to calculate the maximum p-value used in the final hypothesis test.

© 2025 Newby J et al. *JAMA Network Open.*

eTable 2. Maximum *P* Value for Each Distress Severity Group at Each Interim Analysis and Final Analysis

Interim Hypothesis Test: Critical p-value			
	**1**	**2**	**3**
	**Interim Analysis**	**Interim Analysis**	**Final analysis**
	**Mini-trial 4**	**Mini-trial 8**	**Mini-trial 12**
**Mild**	0.0006869	0.0161445	0.0449651
**Moderate**	0.0006869	0.0161445	0.045416
**Severe**	0.0006869	0.0161445	0.0449702

© 2025 Newby J et al. *JAMA Network Open.*

eTable 3. Detailed Breakdown Used to Calculate Final Maximum *P* Values for Each Distress Severity Group

Mild Distress			
	Hypothesis Test 1: Mini-trial 4	Hypothesis Test 2: Mini-trial 8	Hypothesis Test 3: Mini-trial 12
Data points	131	328	494
%of Data points for severity group	26.52%	66.40%	100.00%
p-value	0.0006869	0.0161445	0.0449651
α-spent	0.0006869	0.0163747	0.05

Moderate Distress			
	Hypothesis Test 1: Mini-trial 4	Hypothesis Test 2: Mini-trial 8	Hypothesis Test 3: Mini-trial 12
Data points	140	302	439
%of Data points for severity group	31.82%	68.64%	100.00%
p-value	0.0006869	0.0161445	0.045416
α-spent	0.0006869	0.0163747	0.05

Severe Distress			
	Hypothesis Test 1: Mini-trial 4	Hypothesis Test 2: Mini-trial 8	Hypothesis Test 3: Mini-trial 12
Data points	107	232	349
%of Data points for severity group	30.57%	66.29%	100.00%
p-value	0.0006869	0.0161445	0.0449702
α-spent	0.0006869	0.0163747	0.05

eTable3 provides the participant numbers after each interim hypothesis testing point, broken down by their severity group. The last of these tests indicate the final total number of participants per cohort used for the analysis. Of note, there were two participants whose data were included in the interim analyses 1 and 2, who were not included in the final analysis because it became apparent they had duplicated data at the interim analyses.

© 2025 Newby J et al. *JAMA Network Open.*

eTable 4. Number of Participants Per Distress Severity Group Included in the Interim Analyses and Final Analysis

**Number of participants in each analysis**			
	**1**	**2**	**3**
	**Interim Analysis**	**Interim Analysis**	**Final analysis**
	**Mini-trial 4**	**Mini-trial 8**	**Mini-trial 12**
**Mild**	131	328	494
**Moderate**	139	301	439
**Severe**	106	231	349

© 2025 Newby J et al. *JAMA Network Open.*

eTable 5. Interim Hypothesis Tests After Mini-Trials 4 and 8 for DASS-21 Total Scores

	Interim Analysis 1 (after mini-trial 4)						Interim Analysis 2 (after mini-trial 8)					
	Mild		Moderate		Severe		Mild		Moderate		Severe	
Comparison	*p* value	BH	*p* value	BH	*p* value	BH	*p* value	BH	*p* value	BH	*p* value	BH
Mindfulness > Control	0.06	0.000	0.17	0.000	0.002	0.000	0.05	0.008	0.02	0.003	**0.0025***	0.003
Physical Activity > Control	0.01	0.000	0.19	0.000	0.01	0.000	0.005	0.003	0.06	0.008	0.02	0.005
Sleep Hygiene > Control	0.02	0.000	0.03	0.000	0.13	0.001	0.007	0.005	0.04	0.005	0.14	0.01
Physical Activity> Mindfulness	0.36	0.001	0.45	0.001	0.21	0.001	0.20	0.01	0.33	0.01	0.27	0.02
Sleep Hygiene > Mindfulness	0.31	0.000	0.20	0.001	0.02	0.000	0.26	0.01	0.25	0.01	0.03	0.008
Physical Activity > Sleep Hygiene	0.42	0.001	0.15	0.000	0.11	0.000	0.37	0.02	0.48	0.02	0.14	0.01

Note. * *p* value lower than Benjamini-Hochberg (BH) adjusted critical *p* value, which was used to control for inflation of Type 1 error rates.

© 2025 Newby J et al. *JAMA Network Open.*

eResults.

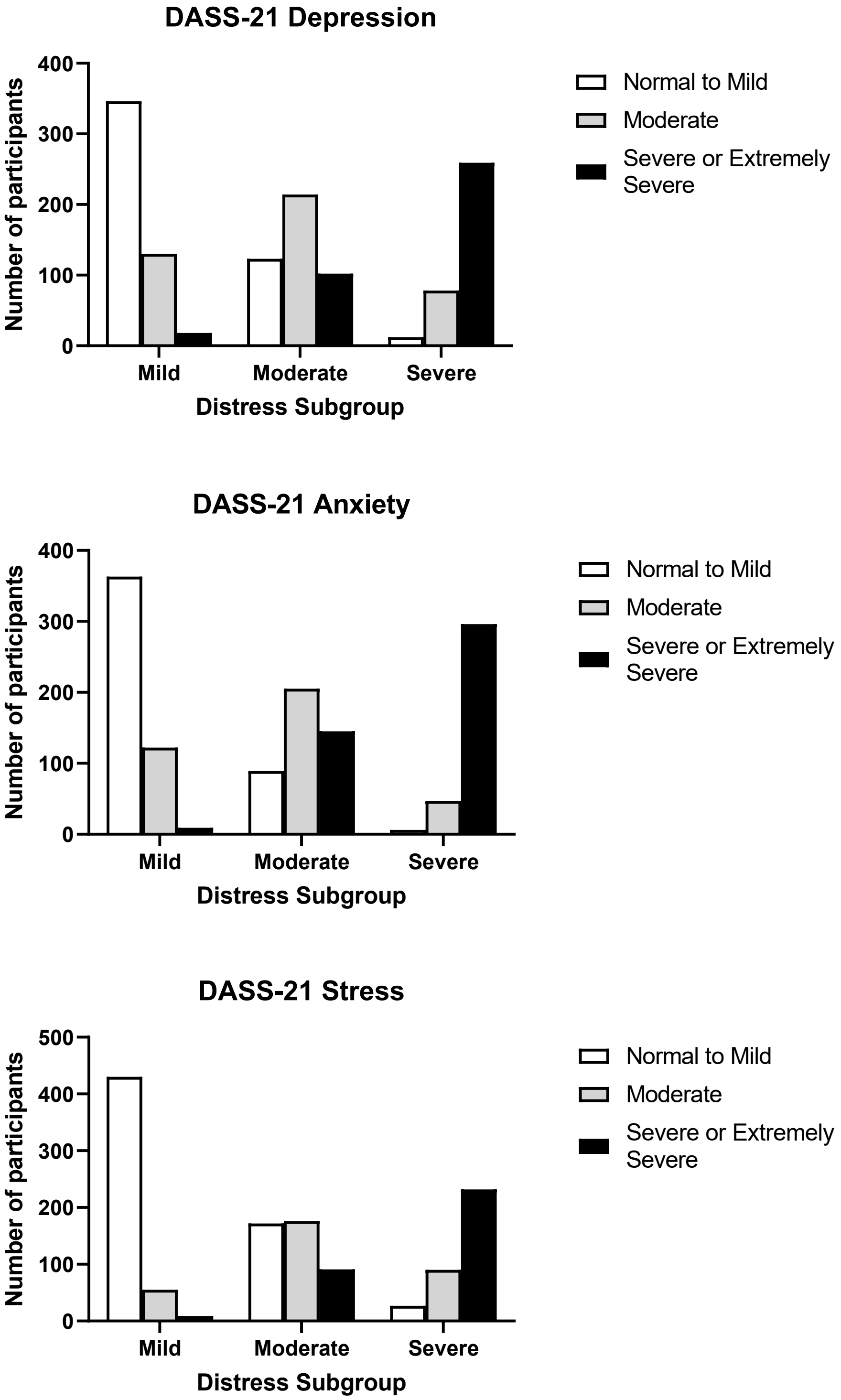

eFigure 5. Number of Participants in the Mild, Moderate, and Severely Distressed Subgroups With Scores in the Normal/Mild, Moderate, and Severe/Extremely Severe Ranges on the DASS-21 Depression, Anxiety and Stress Subscales at Baseline

© 2025 Newby J et al. *JAMA Network Open.*

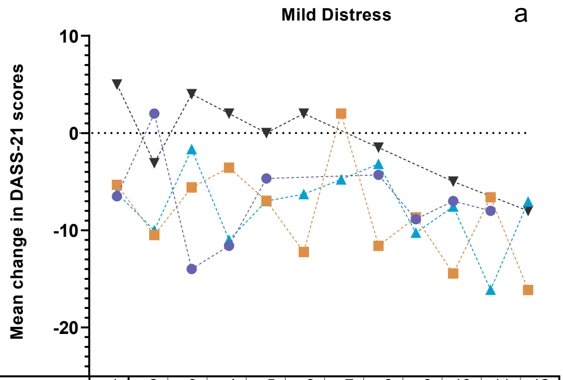

Mini Trial	1	2	3	4	5	6	7	8	9	10	11	12
Mindfulness	8	4	2	5	15	0	0	13	7	4	3	0
Physical Activity	9	8	20	9	23	8	6	5	9	9	23	14
Sleep Hygiene	11	5	6	4	14	35	31	22	23	23	17	17
Ecological Momentary Assessment	4	11	4	1	5	1	0	4	0	2	0	1

Number of participants per mini trial

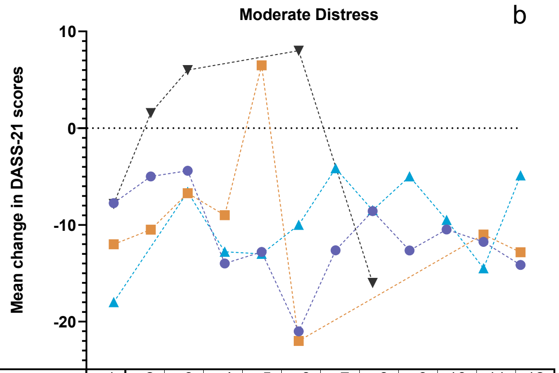

Mini Trial	1	2	3	4	5	6	7	8	9	10	11	12
Mindfulness	8	10	5	2	20	18	19	14	22	21	17	13
Physical Activity	5	4	11	8	4	9	0	0	0	0	2	12
Sleep Hygiene	10	0	17	23	16	13	13	12	8	4	4	9
Ecological Momentary Assessment	12	9	2	0	0	1	0	1	0	0	0	0

Number of participants per mini trial

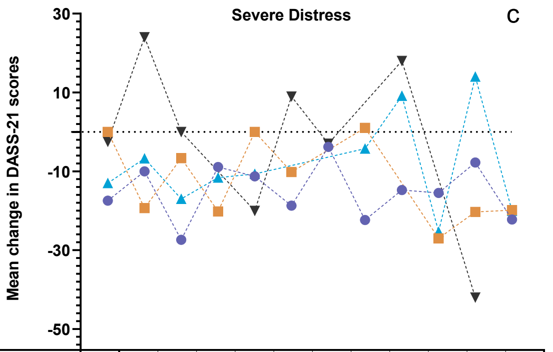

Mini Trial	1	2	3	4	5	6	7	8	9	10	11	12
Mindfulness	7	2	13	9	22	17	19	6	16	19	9	9
Physical Activity	4	6	3	11	1	11	0	2	0	6	13	15
Sleep Hygiene	4	11	2	5	12	0	0	8	7	3	1	2
Ecological Momentary Assessment	8	1	5	0	3	2	6	0	1	0	2	0

Number of participants per mini trial

© 2025 Newby J et al. *JAMA Network Open.*

eFigure 6. Mean Change in DASS-21 Total Score From Pre- to Post-Intervention (Top), and the Number of Participants (Bottom) Allocated Across Each of the 12 Mini-Trials, for Mild (a), Moderate (b), and Severe (c) Distress Groups

© 2025 Newby J et al. *JAMA Network Open.*

eTable 6. Observed DASS-21 Total Scores for the Mild Distress Group by Intervention Group Across Each of the Twelve Mini-Trials

	**Mindfulness**				**Physical Activity**				**Sleep Hygiene**				**EMA**			
Mini-trial	Baseline	Pre	Post	Mean change	Baseline	Pre	Post	Mean change	Baseline	Pre	Post	Mean change	Baseline	Pre	Post	Mean change
	N M(SD)	N M(SD)	N M(SD)	N M(SD)	N M(SD)	N M(SD)	N M(SD)	N M(SD)	N M(SD)	N M(SD)	N M(SD)	N M(SD)	N M(SD)	N M(SD)	N M(SD)	N M(SD)
1	8	8	8	8	12	12	9	9	12	12	11	11	4	4	4	4
	29.0 (5.1)	31.0 (14.14)	24.5 (8.65)	-6.5 (14.48)	31.0 (6.24)	39.33 (13.7)	28.0 (10.54)	-5.33 (7.66)	31.33 (6.55)	25.67 (11.77)	18.0 (10.75)	-6.18 (8.55)	29.0 (3.61)	29.0 (10.05)	34.0 (11.22)	5.0 (3.0)
2	6	6	4	4	10	10	8	8	6	6	5	5	13	13	11	11
	29.0 (5.39)	31.0 (7.9)	28.5 (14.31)	2.0 (13.93)	29.0 (1.84)	30.2 (6.66)	21.25 (6.78)	-10.5 (12.11)	19.67 (2.69)	24.67 (5.96)	16.4 (4.08)	-10.0 (6.07)	36.62 (1.44)	37.54 (10.59)	34.73 (9.58)	-3.09 (8.67)
3	4	4	2	2	22	22	20	20	6	6	6	6	5	5	4	4
	30.5 (5.17)	38.5 (12.36)	24.0 (0.0)	-14.0 (4.0)	28.91 (9.0)	38.82 (18.34)	30.0 (15.11)	-5.6 (16.54)	32.33 (2.92)	31.0 (14.69)	29.33 (7.8)	-1.67 (15.98)	20.8 (2.71)	24.0 (12.13)	29.0 (16.16)	4.0 (6.16)
4	6	6	5	5	11	11	9	9	4	4	4	4	2	2	1	1
	30.0 (5.03)	32.67 (15.82)	20.8 (12.04)	-11.6 (8.52)	32.55 (4.98)	33.09 (10.56)	26.44 (13.85)	-3.56 (16.27)	25.5 (5.55)	34.0 (1.41)	23.0 (13.45)	-11.0 (13.0)	16.0 (2.0)	17.0 (7.0)	12.0 (0.0)	2.0 (0.0)
5	17	17	15	15	23	23	23	23	15	15	14	14	5	5	5	5
	24.59 (11.01)	30.71 (14.32)	24.53 (15.65)	-4.67 (10.52)	35.83 (5.62)	33.3 (12.18)	26.35 (16.45)	-6.96 (12.54)	32.13 (9.56)	33.07 (13.62)	26.71 (16.66)	-7.0 (13.13)	24.0 (1.26)	28.0 (13.39)	28.0 (11.45)	0.0 (6.81)
6	0	0	0	0	9	9	8	8	40	40	35	35	1	1	1	1
	- (-)	- (-)	- (-)	- (-)	34.22 (5.45)	34.0 (8.64)	19.0 (6.78)	-12.25 (8.8)	29.6 (6.17)	29.65 (13.53)	23.83 (12.69)	-6.29 (14.64)	8.0 (0.0)	8.0 (0.0)	10.0 (0.0)	2.0 (0.0)
7	0	0	0	0	7	7	6	6	33	33	31	31	0	0	0	0
	- (-)	- (-)	- (-)	- (-)	28.0 (2.39)	22.57 (7.15)	25.0 (11.47)	2.0 (16.73)	29.82 (9.35)	29.88 (13.64)	24.19 (17.5)	-4.77 (15.85)	- (-)	- (-)	- (-)	- (-)
8	15	15	13	13	6	6	5	5	22	22	22	22	4	4	4	4
	26.13 (6.17)	26.93 (9.18)	22.31 (5.48)	-4.31 (12.1)	35.33 (7.36)	39.0 (6.61)	28.0 (8.49)	-11.6 (8.24)	34.09 (6.45)	30.18 (7.43)	27.0 (14.57)	-3.18 (15.39)	13.5 (1.66)	12.5 (2.96)	11.0 (7.14)	-1.5 (7.4)
9	8	8	7	7	12	12	9	9	23	23	23	23	0	0	0	0
	28.5 (3.84)	29.0 (11.87)	21.71 (8.17)	-8.86 (13.52)	28.0 (2.45)	31.67 (11.01)	22.89 (11.04)	-8.67 (8.43)	34.7 (7.82)	36.87 (14.64)	26.61 (12.38)	-10.26 (16.23)	- (-)	- (-)	- (-)	- (-)
10	4	4	4	4	9	9	9	9	24	24	23	23	2	2	2	2
	29.5 (4.33)	38.5 (12.03)	31.5 (17.57)	-7.0 (11.18)	28.22 (2.74)	31.56 (8.15)	17.11 (11.51)	-14.44 (12.25)	33.0 (10.49)	32.33 (17.49)	25.74 (12.99)	-7.57 (12.13)	15.0 (1.0)	18.0 (12.0)	13.0 (11.0)	-5.0 (1.0)
11	4	4	3	3	24	24	23	23	20	20	17	17	0	0	0	0
	26.0 (7.87)	18.5 (4.97)	12.67 (0.94)	-8.0 (2.83)	30.42 (3.7)	31.58 (8.96)	24.52 (13.43)	-6.61 (12.16)	35.5 (9.1)	34.3 (12.49)	19.76 (9.99)	-16.12 (15.38)	- (-)	- (-)	- (-)	- (-)
12	0	0	0	0	16	16	14	14	19	19	17	17	1	1	1	1
	- (-)	- (-)	- (-)	- (-)	33.62 (5.49)	32.75 (13.76)	19.71 (10.55)	-16.14 (11.84)	34.32 (7.0)	36.11 (14.19)	31.18 (16.07)	-7.06 (12.18)	16.0 (0.0)	20.0 (0.0)	12.0 (0.0)	-8.0 (0.0)
Overall[a] N	72	72	61	61	161	161	143	143	224	224	208	208	37	37	33	33

© 2025 Newby J et al. *JAMA Network Open.*

M(SD)	27.33 (7.48)	30.14 (12.89)	23.54 (11.96)	-6.07 (12.08)	31.39 (6.16)	33.55 (12.55)	24.53 (13.34)	-8.15 (13.42)	31.88 (8.49)	31.72 (13.71)	24.86 (14.22)	-7.27 (14.63)	25.84 (9.43)	27.35 (13.79)	26.61 (14.23)	-0.55 (7.50)

Note. Pre = pre-intervention, Post = post-intervention, mean change = change in scores from pre- to post-intervention, M=mean, SD = standard deviation, N= number of participants. [a] Averaged across mini-trials. EMA = Ecological Momentary Assessment.

© 2025 Newby J et al. *JAMA Network Open.*

eTable 7. Observed DASS-21 Total Scores for the Moderate Distress Group by Intervention Group Across Each of the Twelve Mini-Trials

	Mindfulness				Physical Activity				Sleep Hygiene				EMA			
Mini-trial	Baseline	Pre	Post	Mean change	Baseline	Pre	Post	Mean change	Baseline	Pre	Post	Mean change	Baseline	Pre	Post	Mean change
	N M(SD)	N M(SD)	N M(SD)	N M(SD)	N M(SD)	N M(SD)	N M(SD)	N M(SD)	N M(SD)	N M(SD)	N M(SD)	N M(SD)	N M(SD)	N M(SD)	N M(SD)	N M(SD)
1	9	9	8	8	5	5	5	5	10	10	10	10	13	13	12	12
	54.22 (7.45)	51.78 (12.24)	43.5 (18.27)	-7.75 (24.09)	50.8 (3.92)	41.6 (11.2)	29.6 (5.12)	-12.0 (15.59)	53.2 (3.92)	51.4 (13.3)	33.4 (14.31)	-18.0 (13.11)	50.62 (6.72)	46.77 (14.11)	40.0 (13.09)	-7.83 (11.65)
2	13	13	10	10	4	4	4	4	1	1	0	0	9	9	9	9
	46.62 (3.79)	36.0 (8.38)	29.2 (13.42)	-5.0 (9.0)	56.0 (2.45)	46.5 (16.99)	36.0 (21.4)	-10.5 (12.6)	50.0 (0.0)	56.0 (0.0)	- (-)	- (-)	50.44 (6.09)	44.67 (8.27)	46.22 (23.75)	1.56 (23.62)
3	5	5	5	5	11	11	11	11	18	18	17	17	2	2	2	2
	54.4 (1.5)	57.6 (15.2)	53.2 (31.86)	-4.4 (24.7)	45.64 (1.87)	44.18 (13.31)	37.45 (10.38)	-6.73 (10.63)	49.33 (3.4)	43.44 (10.11)	36.71 (11.92)	-6.59 (10.22)	57.0 (1.0)	49.0 (7.0)	55.0 (1.0)	6.0 (6.0)
4	2	2	2	2	11	11	8	8	26	26	23	23	0	0	0	0
	50.0 (2.0)	43.0 (3.0)	29.0 (13.0)	-14.0 (16.0)	48.18 (3.46)	52.0 (9.98)	42.0 (13.3)	-9.0 (14.18)	51.85 (7.21)	52.0 (13.21)	39.3 (17.01)	-12.78 (15.6)	- (-)	- (-)	- (-)	- (-)
5	22	22	20	20	6	6	4	4	19	19	16	16	0	0	0	0
	50.82 (2.06)	49.18 (10.95)	37.6 (14.47)	-12.8 (18.0)	56.0 (3.65)	38.33 (9.34)	45.0 (13.45)	6.5 (12.11)	47.26 (5.99)	46.84 (13.82)	36.62 (12.66)	-13.0 (13.1)	- (-)	- (-)	- (-)	- (-)
6	18	18	18	18	10	10	9	9	15	15	13	13	2	2	1	1
	55.44 (3.04)	54.78 (13.25)	33.78 (13.1)	-21.0 (14.35)	48.2 (4.24)	47.6 (7.58)	24.67 (13.92)	-22.0 (16.65)	46.13 (3.9)	45.07 (14.86)	36.62 (14.6)	-10.0 (17.66)	60.0 (0.0)	53.0 (5.0)	56.0 (0.0)	8.0 (0.0)
7	24	24	19	19	0	0	0	0	16	16	13	13	0	0	0	0
	54.25 (3.76)	50.08 (10.64)	39.05 (12.47)	-12.63 (12.38)	- (-)	- (-)	- (-)	- (-)	44.38 (3.33)	37.62 (8.19)	34.62 (15.87)	-4.15 (16.5)	- (-)	- (-)	- (-)	- (-)
8	16	16	14	14	0	0	0	0	13	13	12	12	1	1	1	1
	51.12 (3.24)	53.75 (11.09)	45.14 (20.46)	-8.57 (24.12)	- (-)	- (-)	- (-)	- (-)	50.62 (8.24)	50.77 (9.66)	40.33 (14.14)	-8.5 (12.89)	52.0 (0.0)	62.0 (0.0)	46.0 (0.0)	-16.0 (0.0)
9	30	30	22	22	0	0	0	0	9	9	8	8	0	0	0	0
	52.73 (4.08)	52.53 (12.56)	40.09 (15.32)	-12.64 (18.64)	- (-)	- (-)	- (-)	- (-)	42.89 (1.37)	40.22 (12.27)	36.25 (19.76)	-5.0 (16.55)	- (-)	- (-)	- (-)	- (-)
10	24	24	21	21	1	1	0	0	5	5	4	4	0	0	0	0
	51.83 (4.36)	51.17 (14.87)	41.71 (13.16)	-10.48 (17.03)	48.0 (0.0)	36.0 (0.0)	- (-)	- (-)	46.4 (6.86)	41.2 (5.46)	33.0 (15.33)	-9.5 (14.99)	- (-)	- (-)	- (-)	- (-)
11	23	23	17	17	3	3	2	2	4	4	4	4	0	0	0	0
	52.17 (4.82)	49.22 (11.7)	40.82 (16.64)	-11.76 (16.14)	53.33 (8.06)	41.33 (15.43)	23.0 (11.0)	-11.0 (3.0)	45.0 (3.0)	34.0 (5.1)	19.5 (2.96)	-14.5 (3.84)	- (-)	- (-)	- (-)	- (-)
12	15	15	13	13	14	14	12	12	10	10	9	9	0	0	0	0
	53.73 (2.29)	45.73 (15.58)	31.38 (15.87)	-14.15 (13.82)	54.71 (4.51)	49.71 (8.78)	35.67 (16.55)	-12.83 (17.27)	44.0 (1.55)	39.8 (8.83)	35.78 (15.3)	-4.89 (11.93)	- (-)	- (-)	- (-)	- (-)
Overall[a] N	201	201	169	169	65	65	55	55	146	146	129	129	27	27	25	25
M(SD)	52.41 (4.41)	50.05 (13.17)	38.8 (16.7)	-12.06 (17.81)	50.8 (5.49)	46.37 (11.75)	34.84 (15.27)	-10.84 (15.94)	48.04 (6.13)	45.44 (12.81)	36.28 (15.26)	-9.77 (14.73)	51.78 (6.52)	47.26 (11.71)	44.32 (17.62)	-3.04 (17.48)

Note. Pre = pre-intervention, Post = post-intervention, mean change = change in scores from pre- to post-intervention, M=mean, SD = standard deviation, N= number of participants. [a] Averaged across mini-trials. EMA = Ecological Momentary Assessment.

© 2025 Newby J et al. *JAMA Network Open.*

eTable 8. Observed DASS-21 Total Scores for the Severe Distress Group by Intervention Group Across Each of the Twelve Mini-Trials

	Mindfulness				Physical Activity				Sleep Hygiene				EMA			
Mini-trial	Baseline	Pre	Post	Mean change	Baseline	Pre	Post	Mean change	Baseline	Pre	Post	Mean change	Baseline	Pre	Post	Mean change
	N M(SD)	N M(SD)	N M(SD)	N M(SD)	N M(SD)	N M(SD)	N M(SD)	N M(SD)	N M(SD)	N M(SD)	N M(SD)	N M(SD)	N M(SD)	N M(SD)	N M(SD)	N M(SD)
1	9	9	7	7	5	5	4	4	6	6	4	4	8	8	8	8
	79.78 (12.16)	68.89 (11.36)	49.43 (19.99)	-17.43 (12.9)	72.0 (10.35)	59.2 (8.35)	60.5 (36.2)	0.0 (34.15)	82.33 (11.97)	71.33 (10.69)	54.0 (16.91)	-13.0 (18.68)	71.75 (4.94)	56.0 (16.88)	53.5 (16.9)	-2.5 (17.49)
2	4	4	2	2	6	6	6	6	12	12	11	11	1	1	1	1
	74.0 (2.45)	70.5 (10.14)	70.0 (12.0)	-10.0 (16.0)	73.33 (6.7)	59.33 (8.99)	40.0 (11.72)	-19.33 (9.36)	69.67 (6.62)	63.17 (18.47)	55.64 (20.8)	-6.73 (17.73)	100.0 (0.0)	80.0 (0.0)	104.0 (0.0)	24.0 (0.0)
3	14	14	13	13	3	3	3	3	2	2	2	2	6	6	5	5
	64.86 (3.68)	63.14 (11.08)	35.23 (16.8)	-27.38 (21.86)	79.33 (1.89)	63.33 (6.6)	56.67 (11.81)	-6.67 (8.22)	79.0 (5.0)	53.0 (7.0)	36.0 (2.0)	-17.0 (9.0)	82.67 (8.3)	67.67 (11.74)	69.6 (17.32)	0.0 (17.93)
4	11	11	9	9	13	13	11	11	6	6	5	5	0	0	0	0
	69.09 (10.87)	58.91 (17.69)	51.78 (4.47)	-8.89 (16.76)	83.54 (15.19)	73.23 (18.17)	55.27 (24.14)	-20.18 (24.46)	78.0 (2.83)	79.67 (17.72)	67.2 (15.93)	-11.6 (15.87)	- (-)	- (-)	- (-)	- (-)
5	23	23	22	22	1	1	1	1	12	12	12	12	3	3	3	3
	69.65 (10.59)	63.48 (18.48)	52.55 (18.06)	-11.27 (25.57)	90.0 (0.0)	84.0 (0.0)	84.0 (0.0)	0.0 (0.0)	77.5 (6.89)	73.17 (16.66)	62.5 (21.48)	-10.67 (18.43)	86.67 (2.49)	72.67 (8.06)	52.67 (20.15)	-20.0 (12.33)
6	23	23	17	17	13	13	11	11	0	0	0	0	2	2	2	2
	70.43 (10.57)	60.96 (18.59)	44.24 (22.49)	-18.71 (13.84)	78.77 (13.91)	70.46 (18.32)	61.09 (29.99)	-10.18 (20.48)	- (-)	- (-)	- (-)	- (-)	92.0 (2.0)	71.0 (13.0)	80.0 (20.0)	9.0 (7.0)
7	24	24	19	19	0	0	0	0	0	0	0	0	6	6	6	6
	70.08 (8.11)	56.5 (17.11)	53.05 (25.32)	-3.79 (22.14)	- (-)	- (-)	- (-)	- (-)	- (-)	- (-)	- (-)	- (-)	83.67 (3.14)	73.0 (21.28)	70.0 (17.32)	-3.0 (22.88)
8	7	7	6	6	2	2	2	2	9	9	8	8	0	0	0	0
	73.71 (12.12)	69.43 (18.57)	50.33 (24.09)	-22.33 (19.44)	62.0 (2.0)	52.0 (2.0)	53.0 (7.0)	1.0 (5.0)	77.56 (10.23)	64.89 (19.82)	56.75 (20.3)	-4.25 (15.47)	- (-)	- (-)	- (-)	- (-)
9	20	20	16	16	0	0	0	0	7	7	7	7	1	1	1	1
	73.3 (10.01)	68.8 (15.99)	51.12 (21.64)	-14.75 (19.41)	- (-)	- (-)	- (-)	- (-)	72.0 (3.55)	44.0 (24.31)	53.14 (27.75)	9.14 (23.25)	76.0 (0.0)	70.0 (0.0)	88.0 (0.0)	18.0 (0.0)
10	20	20	19	19	6	6	6	6	3	3	3	3	0	0	0	0
	81.1 (15.07)	69.5 (19.12)	53.16 (18.28)	-15.47 (18.85)	69.0 (5.97)	74.33 (16.39)	47.33 (15.61)	-27.0 (13.89)	72.67 (3.4)	70.67 (10.5)	45.33 (4.11)	-25.33 (6.6)	- (-)	- (-)	- (-)	- (-)
11	12	12	9	9	14	14	13	13	2	2	1	1	2	2	2	2
	86.0 (12.78)	68.33 (18.13)	60.67 (25.25)	-7.78 (13.64)	74.57 (10.29)	58.0 (20.51)	37.23 (25.29)	-20.31 (24.24)	75.0 (1.0)	58.0 (2.0)	70.0 (0.0)	14.0 (0.0)	108.0 (0.0)	105.0 (1.0)	63.0 (29.0)	-42.0 (28.0)
12	13	13	9	9	16	16	15	15	2	2	2	2	0	0	0	0
	66.0 (3.76)	49.69 (10.43)	26.22 (14.95)	-22.22 (12.98)	77.12 (10.1)	68.12 (20.16)	49.2 (17.77)	-19.87 (24.13)	82.0 (4.0)	70.0 (0.0)	50.0 (18.0)	-20.0 (18.0)	- (-)	- (-)	- (-)	- (-)
Overall[a] N	180	180	148	148	79	79	72	72	61	61	55	55	29	29	28	28
M(SD)	72.67 (11.87)	63.17 (17.65)	48.91 (21.85)	-14.53 (20.5)	76.63 (12.1)	66.41 (18.55)	50.39 (24.6)	-16.56 (23.0)	75.74 (8.28)	65.7 (19.86)	56.69 (21.19)	-7.6 (19.58)	83.03 (10.99)	69.38 (19.1)	65.43 (22.01)	-4.36 (22.9)

© 2025 Newby J et al. *JAMA Network Open.*

Note. Pre = pre-intervention, Post = post-intervention, mean change = change in scores from pre- to post-intervention, M=mean, SD = standard deviation, N= number of participants. [a] Averaged across mini-trials. EMA = Ecological Momentary Assessment.

© 2025 Newby J et al. *JAMA Network Open.*

eTable 9. Unweighted Observed Means and Standard Deviations for DASS-21 Depression Subscale Scores Pre- and Post-Intervention, and Change by Severity and Intervention Group

	**Pre-intervention**	**Post-intervention**	**Pre-Post Change**
	Observed Mean (SD)	Observed Mean (SD)	Observed Mean (SD)
**Severity Group: Mild**			
Physical Activity	11.43 (7.11)	8.31 (6.23)	-2.76 (6.35)
Mindfulness	10.5 (6.09)	7.8 (5.67)	-2.30 (5.52)
Sleep Hygiene	10.88 (7.00)	8.08 (6.48)	-3.06 (6.70)
EMA	8.81 (6.17)	7.82 (6.75)	-0.85 (4.58)
**Severity Group: Moderate**			
Physical Activity	14.4 (6.37)	10.62 (6.92)	-3.53 (7.15)
Mindfulness	17 (8.30)	13.56 (7.81)	-3.68 (7.68)
Sleep Hygiene	15.88 (8.35)	12.56 (7.64)	-3.40 (7.60)
EMA	16.52 (7.18)	15.44 (8.8)	-0.80 (6.95)
**Severity Group: Severe**			
Physical Activity	22.51 (8.81)	17.06 (10.41)	-5.86 (9.54)
Mindfulness	22.62 (9.06)	17.05 (10.18)	-5.53 (8.61)
Sleep Hygiene	24.3 (10.18)	21.31 (10.16)	-2.55 (7.81)
EMA	23.24 (11.2)	22.07 (10.81)	-1.36 (10.47)

Note. EMA = Ecological Momentary Assessment.

© 2025 Newby J et al. *JAMA Network Open.*

eTable 10. Unweighted Observed Means and Standard Deviations for DASS-21 Anxiety Subscale Scores Pre- and Post-Intervention, and Change by Severity and Intervention Group

	**Pre-intervention**	**Post-intervention**	**Pre-Post Change**
	Observed Mean (SD)	Observed Mean (SD)	Observed Mean (SD)
**Severity Group: Mild**			
Physical Activity	6.81 (4.88)	5.01 (4.64)	-1.45 (4.44)
Mindfulness	6.06 (4.68)	5.48 (4.76)	-0.23 (4.73)
Sleep Hygiene	6.73 (5.04)	5.10 (4.61)	-1.66 (4.98)
EMA	6.70 (5.80)	6.55 (5.25)	-0.06 (3.76)
**Severity Group: Moderate**			
Physical Activity	12.18 (5.60)	8.95 (5.36)	-3.20 (6.09)
Mindfulness	12.25 (5.46)	9.21 (6.00)	-3.40 (6.42)
Sleep Hygiene	11.10 (5.76)	8.20 (4.89)	-3.22 (5.17)
EMA	11.41 (4.50)	10.80 (5.60)	-0.48 (6.25)
**Severity Group: Severe**			
Physical Activity	18.23 (7.05)	14.31 (7.56)	-3.86 (8.09)
Mindfulness	17.00 (7.33)	13.04 (7.51)	-4.09 (7.15)
Sleep Hygiene	16.95 (7.58)	14.65 (7.97)	-2.11 (7.73)
EMA	19.31 (6.87)	19.14 (8.37)	-0.14 (7.89)

Note. EMA = Ecological Momentary Assessment.

© 2025 Newby J et al. *JAMA Network Open.*

eTable 11. Unweighted Observed Means and Standard Deviations for DASS-21 Stress Subscale Scores Pre- and Post-Intervention, and Change by Severity and Intervention Group

	Pre-intervention	Post-intervention	Pre-Post Change
	Observed Mean (SD)	Observed Mean (SD)	Observed Mean (SD)
**Severity Group: Mild**			
Physical Activity	15.32 (6.03)	11.22 (6.07)	-3.94 (6.47)
Mindfulness	13.58 (6.58)	10.26 (6.41)	-3.54 (6.24)
Sleep Hygiene	14.11 (6.36)	11.68 (6.62)	-2.55 (6.65)
EMA	11.84 (6.17)	12.24 (6.34)	0.36 (4.37)
**Severity Group: Moderate**			
Physical Activity	19.78 (6.02)	15.27 (7.02)	-4.11 (7.55)
Mindfulness	20.80 (6.27)	16.04 (6.96)	-4.98 (7.52)
Sleep Hygiene	18.47 (5.95)	15.52 (6.86)	-3.15 (6.41)
EMA	19.33 (6.49)	18.08 (6.79)	-1.76 (6.98)
**Severity Group: Severe**			
Physical Activity	25.67 (7.05)	19.03 (9.49)	-6.83 (8.62)
Mindfulness	23.54 (6.78)	18.81 (8.15)	-4.91 (8.28)
Sleep Hygiene	24.46 (7.89)	20.73 (7.84)	-2.95 (7.71)
EMA	26.83 (8.01)	24.21 (8.28)	-2.86 (8.65)

Note. EMA = Ecological Momentary Assessment.

© 2025 Newby J et al. *JAMA Network Open.*

eTable 12. Group Comparisons of the Pre- to Post-Changes in Bias Corrected and Log Transformed DASS-21 Depression Subscale Scores

	Mild			Moderate			Severe		
Comparison	SMD (95%CI)	p value	BH	SMD (95%CI)	p value	BH	SMD (95%CI)	p value	BH
Mindfulness > Control	0.19(-0.17-0.55)	0.19	0.03	0.22(-0.15-0.59)	0.15	0.02	**0.61(0.23-1.00)***	0.002	0.02
Physical Activity > Control	0.31(0.01-0.61)	0.06	0.02	0.37(-0.08-0.81)	0.06	0.008	**0.61(0.18-1.04)***	0.003	0.03
Sleep Hygiene > Control	0.36(0.08-0.64)	0.03	0.008	0.21(-0.17-0.60)	0.16	0.02	0.11(-0.30-0.52)	0.31	0.04
Physical Activity> Mindfulness	0.12(-0.20-0.44)	0.22	0.04	0.15(-0.17-0.47)	0.17	0.04	0(-0.31-0.30)	0.49	0.05
Sleep Hygiene > Mindfulness	0.17(-0.12-0.46)	0.12	0.02	-0.01(-0.24-0.23)	0.48	0.05	**-0.50(-0.78--0.22)***	0.001	0.008
Physical Activity > Sleep Hygiene	-0.05(-0.28-0.17)	0.31	0.05	0.15(-0.17-0.46)	0.17	0.03	**0.50(0.15-0.85)***	0.003	0.02

Note. SMD = standardised mean difference. * *p* value lower than Benjamini-Hochberg (BH) adjusted critical *p* value.

© 2025 Newby J et al. *JAMA Network Open.*

eTable 13. Group Comparisons of the Pre- to Post-Changes in Bias Corrected and Log Transformed DASS-21 Anxiety Subscale Scores

	Mild			Moderate			Severe		
Comparison	SMD (95%CI)	p value	BH	SMD (95%CI)	p value	BH	SMD (95%CI)	p value	BH
Mindfulness > Control	0.11(-0.25-0.46)	0.31	0.05	0.46(0.10-0.81)	0.02	0.008	0.47(0.14-0.81)	0.01	0.008
Physical Activity > Control	0.46(0.16-0.75)	0.009	0.008	0.40(-0.04-0.81)	0.05	0.02	0.41(0.04-0.77)	0.03	0.02
Sleep Hygiene > Control	0.37(0.09-0.65)	0.02	0.02	0.39(0.04-0.74)	0.04	0.02	0.23(-0.18-0.64)	0.16	0.03
Physical Activity> Mindfulness	**0.35 (0.04-0.66)***	0.01	0.02	-0.06(-0.30-0.17)	0.29	0.03	-0.07(-0.35-0.22)	0.32	0.05
Sleep Hygiene > Mindfulness	0.27(-0.03-0.57)	0.03	0.03	-0.07(-0.38-0.24)	0.34	0.04	-0.24(-0.57-0.09)	0.06	0.02
Physical Activity > Sleep Hygiene	0.08(-0.13-0.30)	0.22	0.04	0.00(-0.32-0.31)	0.50	0.05	0.17(-0.20-0.55)	0.17	0.04

Note. SMD = standardised mean difference. * *p* value lower than Benjamini-Hochberg (BH) adjusted critical *p* value.

© 2025 Newby J et al. *JAMA Network Open.*

eTable 14. Group Comparisons of the Pre- to Post-Changes in Bias Corrected and Log Transformed for DASS-21 Stress Subscale Scores

	Mild			Moderate			Severe		
Comparison	SMD (95%CI)	p value	BH	SMD (95%CI)	p value	BH	SMD (95%CI)	p value	BH
Mindfulness > Control	**0.57(0.23-0.90)***	0.004	0.02	0.43(0.08-0.79)	0.02	0.008	0.33(-0.00-0.67)	0.05	0.04
Physical Activity > Control	**0.65(0.36-0.93)***	0.000	0.008	0.37(-0.05-0.78)	0.06	0.02	**0.61(0.23-1.00)***	0.003	0.02
Sleep Hygiene > Control	**0.47(0.20-0.74)***	0.006	0.02	0.28(-0.06-0.63)	0.10	0.02	0.07(-0.31-0.43)	0.39	0.05
Physical Activity> Mindfulness	0.08(-0.22-0.37)	0.30	0.05	-0.07(-0.38-0.24)	0.33	0.05	0.28(-0.03-0.59)	0.03	0.02
Sleep Hygiene > Mindfulness	-0.09(-0.38-0.19)	0.26	0.04	-0.15(-0.38-0.08)	0.10	0.03	-0.27(-0.54-0.00)	0.05	0.03
Physical Activity > Sleep Hygiene	0.17(-0.05-0.39)	0.06	0.03	0.08(-0.23-0.40)	0.31	0.04	**0.55(0.23-0.87)***	0.001	0.008

Note. SMD = standardised mean difference. * *p* value lower than Benjamini-Hochberg (BH) adjusted critical *p* value.

© 2025 Newby J et al. *JAMA Network Open.*

eTable 15. Unweighted Observed Means and Standard Deviations for Sleep Quality Scores Pre- and Post-Intervention, and Change by Severity and Intervention Group

	Pre-intervention	Post-intervention	Pre-Post Change
	Observed Mean (SD)	Observed Mean (SD)	Observed Mean (SD)
**Severity Group: Mild**			
Physical Activity	1.56 (0.61)	1.70 (0.67)	0.13 (0.67)
Mindfulness	1.59 (0.65)	1.74 (0.68)	0.15 (0.70)
Sleep Hygiene	1.50 (0.65)	1.75 (0.64)	0.24 (0.69)
EMA	1.49 (0.70)	1.73 (0.52)	0.18 (0.46)
**Severity Group: Moderate**			
Physical Activity	1.43 (0.73)	1.67 (0.58)	0.22 (0.74)
Mindfulness	1.33 (0.64)	1.59 (0.65)	0.29 (0.72)
Sleep Hygiene	1.43 (0.68)	1.64 (0.66)	0.22 (0.77)
EMA	1.44 (0.70)	1.44 (0.71)	0.04 (0.45)
**Severity Group: Severe**			
Physical Activity	1.25 (0.61)	1.51 (0.69)	0.25 (0.58)
Mindfulness	1.30 (0.70)	1.45 (0.72)	0.19 (0.82)
Sleep Hygiene	1.21 (0.64)	1.58 (0.69)	0.36 (0.78)
EMA	1.28 (0.88)	1.46 (0.64)	0.21 (0.88)

Note. EMA = Ecological Momentary Assessment.

© 2025 Newby J et al. *JAMA Network Open.*

eTable 16. Unweighted Observed Means and Standard Deviations for Mindfulness Scores Pre- and Post-Intervention, and Change by Severity and Intervention Group

	Pre-intervention	Post-intervention	Pre-Post Change
	Observed Mean (SD)	Observed Mean (SD)	Observed Mean (SD)
**Severity Group: Mild**			
Physical Activity	1.25 (1.04)	1.52 (1.02)	0.24 (0.99)
Mindfulness	1.23 (1.00)	2.20 (1.03)	0.93 (1.22)
Sleep Hygiene	1.13 (1.03)	1.49 (1.07)	0.34 (0.95)
EMA	1.40 (1.06)	1.45 (1.06)	0.00 (1.00)
**Severity Group: Moderate**			
Physical Activity	1.08 (1.02)	1.31 (1.06)	0.31 (1.06)
Mindfulness	1.01 (0.89)	2.06 (0.94)	1.08 (1.11)
Sleep Hygiene	1.01 (0.93)	1.37 (0.90)	0.34 (1.02)
EMA	1.26 (0.90)	1.52 (1.19)	0.28 (0.94)
**Severity Group: Severe**			
Physical Activity	1.15 (1.01)	1.50 (1.09)	0.36 (1.10)
Mindfulness	1.06 (0.96)	2.12 (0.98)	1.04 (1.26)
Sleep Hygiene	1.07 (1.05)	1.31 (0.92)	0.27 (1.13)
EMA	1.34 (0.94)	1.54 (1.04)	0.21 (0.88)

Note. EMA = Ecological Momentary Assessment.

© 2025 Newby J et al. *JAMA Network Open.*

eTable 17. Unweighted Observed Means and Standard Deviations for Physical Activity Vital Sign Scores Pre- and Post-Intervention, and Change by Severity and Intervention Group

	Pre-intervention	Post-intervention	Pre-Post Change
	Observed Mean (SD)	Observed Mean (SD)	Observed Mean (SD)
**Severity Group: Mild**			
Physical Activity	116.16 (157.35)	153.02 (163.91)	42.20 (161.39)
Mindfulness	102.93 (113.52)	120.33 (163.22)	5.98 (126.88)
Sleep Hygiene	98.61 (119.52)	121.51 (135.22)	21.07 (109.45)
EMA	89.14 (79.55)	83.52 (86.07)	-11.03 (89.50)
**Severity Group: Moderate**			
Physical Activity	111.31 (136.28)	138.70 (155.47)	29.81 (100.82)
Mindfulness	98.51 (135.61)	106.26 (131.53)	16.21 (123.49)
Sleep Hygiene	88.34 (103.58)	104.81 (138.76)	19.11 (128.65)
EMA	87.41 (183.19)	104.88 (203.05)	20.08 (88.04)
**Severity Group: Severe**			
Physical Activity	110.30 (214.55)	153.26 (139.77)	62.24 (116.69)
Mindfulness	89.67 (112.67)	111.54 (151.20)	21.12 (150.11)
Sleep Hygiene	109.75 (151.04)	111.09 (151.24)	2.45 (108.79)
EMA	106.62 (121.76)	169.64 (208.02)	59.57 (156.20)

Note. EMA = Ecological Momentary Assessment.

© 2025 Newby J et al. *JAMA Network Open.*

eTable 18. Group Comparisons of the Pre- to Post-Changes in Bias Corrected and Log Transformed Sleep Quality Scores

	Mild			Moderate			Severe		
Comparison	SMD (95%CI)	p value	BH	SMD (95%CI)	p value	BH	SMD (95%CI)	p value	BH
Mindfulness > Control	-0.18(-0.60-0.24)	0.20	0.03	0.22(-0.11-0.54)	0.15	0.008	-0.09(-0.50-0.32)	0.33	0.02
Physical Activity > Control	-0.29(-0.66-0.09)	0.07	0.02	0.21(-0.16-0.58)	0.19	0.02	-0.05(-0.34-045)	0.40	0.04
Sleep Hygiene > Control	-0.18(-0.60-0.24)	0.32	0.05	0.17(- 0.17-0.50)	0.22	0.03	-0.01(-0.44-0.46)	0.48	0.05
Physical Activity> Mindfulness	-0.11(-0.39-0.18)	0.24	0.03	-0.01(-0.29-0.28)	0.48	0.05	0.03(-0.21-0.28)	0.40	0.03
Sleep Hygiene > Mindfulness	0.09(-0.19-0.37)	0.26	0.04	-0.05(-0.28-018)	0.33	0.03	0.08(-0.25-0.41)	0.31	0.008
Physical Activity > Sleep Hygiene	-0.2(-0.41-0.01)	0.03	0.008	0.04(-0.25-0.34)	0.39	0.04	-0.04(-0.35-0.27)	0.40	0.03

Note. SMD = standardised mean difference. * *p* value lower than Benjamini-Hochberg (BH) adjusted critical *p* value.

© 2025 Newby J et al. *JAMA Network Open.*

eTable 19. Group Comparisons of the Pre- to Post-Changes in Bias Corrected and Log Transformed Mindfulness Scores

	Mild			Moderate			Severe		
Comparison	SMD (95%CI)	p value	BH	SMD (95%CI)	p value	BH	SMD (95%CI)	p value	BH
Mindfulness > Control	**0.87 (0.44-1.29)***	0.000	0.008	**0.87(0.53-1.21)***	0.000	0.02	**0.72(0.45-0.99)***	0.000	0.03
Physical Activity > Control	0.25(-0.11-0.62)	0.09	0.04	0.17(-0.23-0.56)	0.24	0.04	0.17(-0.13-0.48)	0.21	0.03
Sleep Hygiene > Control	**0.38 (0.02-0.73)***	0.02	0.03	0.26(-0.07-0.59)	0.12	0.03	0.13(-0.21-0.47)	0.28	0.04
Physical Activity> Mindfulness	**-0.61 (-0.93--0.29)***	0.000	0.02	**-0.70(-1.03--0.38)***	0.000	0.03	**-0.55(-0.82--0.27)***	0.000	0.02
Sleep Hygiene > Mindfulness	-0.49(-0.80--0.18)	0.000	0.03	**-0.61(-0.84--0.39)***	0.000	0.008	**-0.59(-0.90--0.27)***	0.000	0.008
Physical Activity > Sleep Hygiene	-0.12(-0.32-0.08)	0.13	0.05	-0.09(-0.42-0.23)	0.28	0.05	0.04(-0.30-0.39)	0.41	0.05

Note. SMD = standardised mean difference. * *p* value lower than Benjamini-Hochberg (BH) adjusted critical *p* value.

© 2025 Newby J et al. *JAMA Network Open.*

eTable 20. Group Comparisons of the Pre- to Post-Changes in Bias Corrected and Log Transformed Physical Activity Vital Sign Scores

	Mild			Moderate			Severe		
Comparison	SMD (95%CI)	p value	BH	SMD (95%CI)	p value	BH	SMD (95%CI)	p value	BH
Mindfulness > Control	0.27(- 0.24-0.77)	0.11	0.04	0.04(-0.42-0.49)	0.43	0.05	-0.13(-0.51-0.24)	0.26	0.05
Physical Activity > Control	**0.53 (0.06-1.00)***	0.003	0.008	0.35(-0.15-0.86)	0.07	0.03	0.19(-0.20-0.58)	0.20	0.04
Sleep Hygiene > Control	0.38(-0.07-0.82)	0.02	0.02	0.11(- 0.37-0.59)	0.31	0.04	-0.37(-0.84-0.09)	0.05	0.03
Physical Activity> Mindfulness	0.27(-0.04-0.57)	0.04	0.03	0.32(0.03-0.60)	0.02	0.008	**0.32 (0.07-0.57)***	0.01	0.02
Sleep Hygiene > Mindfulness	0.11(-0.17-0.39)	0.22	0.05	0.07(-0.16-0.30)	0.26	0.03	-0.24(-0.60-0.13)	0.06	0.03
Physical Activity > Sleep Hygiene	0.15(-0.07-0.38)	0.07	0.03	0.24(-0.08-0.57)	0.06	0.02	**0.56(0.20-0.92)***	0.001	0.008

Note. SMD = standardised mean difference. * *p* value lower than Benjamini-Hochberg (BH) adjusted critical *p* value.

© 2025 Newby J et al. *JAMA Network Open.*

eTable 21. Descriptive Statistics for the App User Engagement Metrics Across Groups

	Total Sample		Mindfulness		Physical Activity		Sleep Hygiene		EMA	
Metric	N	Statistics	N	Statistics	N	Statistics	N	Statistics	N	Statistics
Module Access	1282		453		305		431		93	
Accessed any content, n (%)		1066 (83.15)		380 (83.89)		268 (87.87)		330 (76.57)		88 (94.62)
Accessed half of the content, n (%)		791 (61.70)		280 (61.81)		211 (69.18)		252 (58.47)		48 (51.61)
Accessed all content, n (%)		375 (29.25)		163 (35.98)		40 (13.11)		172 (39.91)		0 (0.00)
Log Data	1189		453		305		431			
Logged any data, n (%)		1056 (88.81)		366 (80.79)		280 (91.80)		410 (95.13)		
Number of logs (0-14), mean (SD)		6.71 (4.94)		4.87 (4.24)		7.45 (4.44)		9.56 (4.26)		
Met requirements*, n (%)		818 (68.80)		290 (64.02)		238 (78.03)		290 (67.29)		
Time logged (minutes)*, mean (SD)		199.21 (210.38)		13.99 (18.02)		68.46 (70.59)		453.84 (55.48)		
Self-report questionnaires										
Credibility Expectancy Questionnaire items (CEQ), mean (SD)	1220		421		293		417		89	
Pre-intervention credibility (1-9)		6.02 (1.61)		5.95 (1.62)		6.08 (1.56)		6.14 (1.61)		5.51 (1.65)
Pre-intervention helpfulness expectancy (0-100%)		38.71 (19.28)		39.10 (19.05)		40.24 (19.57)		38.20 (19.05)		34.27 (20.05)
Usability Questionnaire (UX; 1-5[a]), mean (SD)			377		269		395		84	
Easy to use	1125	4.41 (0.72)		4.44 (0.66)		4.39 (0.70)		4.41 (0.76)		4.31 (0.84)
App useful for my mental health	1125	3.54 (0.96)		3.83 (0.89)		3.51 (0.89)		3.40 (0.97)		3.00 (1.09)
Satisfied with app	1125	3.83 (0.89)		3.97 (0.89)		3.86 (0.84)		3.77 (0.87)		3.46 (1.05)
Had no problems using the app	1125	4.16 (0.88)		4.20 (0.85)		4.07 (0.96)		4.20 (0.84)		4.04 (0.94)
Trusted information in the app	1041	4.37 (0.59)		4.41 (0.58)		4.28 (0.63)		4.40 (0.57)		
Activities/actions were new	1041	2.92 (1.17)		3.11 (1.20)		2.76 (1.13)		2.85 (1.16)		
Put activities/actions in the app into practice (past 2 weeks)	1041	3.77 (0.89)		3.75 (0.96)		3.75 (0.88)		3.81 (0.82)		
Intend to put activities/actions from the app into practice in future	1041	3.91 (0.79)		3.92 (0.84)		3.80 (0.77)		3.96 (0.74)		

Note. CEQ = Abridged Credibility/Expectancy Questionnaire, EMA = ecological momentary assessment (active control), UX = App Usability Questionnaire.
* Mindfulness = length of time engaged in mindfulness (requirement = 3 minutes 38 seconds each day on average – the length of the shortest mindfulness video). Physical Activity = length of time engaged in physical activity (requirement = 150 minutes per week, i.e., average daily exercise of 21.43 minutes), Sleep Hygiene = length of time spent asleep (requirement = 7-9 hours each day on average).
[a] 1=strongly disagree, 2=disagree, 3=undecided, 4=agree, 5= strongly agree.

© 2025 Newby J et al. *JAMA Network Open.*

eReferences.

1. Lovibond SH, Lovibond PF. *Manual for the depression anxiety stress scales (2nd edition)*. 1995.
2. Lu T, Pal D, Pal M. Contextual Multi-Armed Bandits. presented at: Proceedings of the Thirteenth International Conference on Artificial Intelligence and Statistics; 2010; Proceedings of Machine Learning Research. https://proceedings.mlr.press/v9/lu10a.html
3. Rasmussen CE, Williams CK. *Gaussian processes for machine learning* vol 1. MIT Press; 2006.
4. Srinivas N, Krause A, Kakade S, Seeger M. Gaussian Process Optimization in the Bandit Setting. . 2010:
5. Huckvale K, Hoon L, Stech E, et al. Protocol for a bandit-based response adaptive trial to evaluate the effectiveness of brief self-guided digital interventions for reducing psychological distress in university students: the Vibe Up study. *BMJ Open*. Apr 28 2023;13(4):e066249. doi:10.1136/bmjopen-2022-066249
6. Davis S. Mixed models for repeated measures using categorical time effects (MMRM). . *linical trials with missing data: a guide for practitioners*. 2014:130-184.
7. Horvitz DG, Thompson DJ. A Generalization of Sampling Without Replacement from a Finite Universe. *Journal of the American Statistical Association*. 1952/12/01 1952;47(260):663-685. doi:10.1080/01621459.1952.10483446
8. Chen SY, Feng Z, Yi X. A general introduction to adjustment for multiple comparisons. *Journal of thoracic disease*. Jun 2017;9(6):1725-1729. doi:10.21037/jtd.2017.05.34
9. O'Brien PC, Fleming TR. A multiple testing procedure for clinical trials. *Biometrics*. Sep 1979;35(3):549-56.
10. https://www.rpact.org

© 2025 Newby J et al. *JAMA Network Open.*
