# Supplementary material for: Brief Digital Interventions for Psychological Distress: An AI-Enhanced Response-Adaptive Randomized Clinical Trial
Source: JAMA Netw Open. 2025 Oct 31;8(10):e2540502. doi: 10.1001/jamanetworkopen.2025.40502 (PMC12579342; doi:10.1001/jamanetworkopen.2025.40502)
Supplement: Supplement 3. — Data Sharing Statement [file jamanetwopen-e2540502-s003.pdf]

Data Sharing Statement

Newby. Brief Digital Interventions for Psychological Distress. *JAMA Netw Open*. Published October 31, 2025. doi:10.1001/jamanetworkopen.2025.40502

Data
Additional Information: Australian New Zealand Clinical Trials Registry Identifier, ACTRN12621001223820.
Data available: Yes
Data types: Deidentified participant data
How to access data: To request access to de-identified data, please contact Professor Jill Newby, via j.newby@unsw.edu.au
When available: With publication

Supporting Documents
Document types: None

Additional Information
Who can access the data: Researchers whose proposed use of the data has been approved.
Types of analyses: For pre-planned analyses, meta-analyses and individual patient meta-analyses (if applicable).
Mechanisms of data availability: After approval of a proposal by the investigator team, with a signed data access agreement, and appropriate ethical approvals in place.
